# Genome-Wide Analyses of MADS-Box Genes Reveal Their Involvement in Seed Development and Oil Accumulation of Tea-Oil Tree (*Camellia oleifera*)

**DOI:** 10.1155/2024/3375173

**Published:** 2024-07-29

**Authors:** Xianzhi Zhang, Wenliang He, Xinyi Wang, Yongliang Duan, Yongjuan Li, Yi Wang, Qingbin Jiang, Boyong Liao, Sheng Zhou, Yongquan Li

**Affiliations:** ^1^ College of Horticulture and Landscape Architecture Zhongkai University of Agriculture and Engineering, Guangzhou 510225, China; ^2^ Heyuan Branch Center Guangdong Laboratory for Lingnan Modern Agriculture, Heyuan 517500, China; ^3^ School of Mechanic and Electronic Engineering Zhongkai University of Agriculture and Engineering, Guangzhou 510225, China; ^4^ Research Institute of Tropical Forestry Chinese Academy of Forestry, Guangzhou 510520, China

**Keywords:** *Camellia oleifera*, expression pattern, genome-wide analysis, MADS-box gene, oil accumulation, seed development

## Abstract

The seeds of *Camellia oleifera* produce high amount of oil, which can be broadly used in the fields of food, industry, and medicine. However, the molecular regulation mechanisms of seed development and oil accumulation in *C. oleifera* are unclear. In this study, evolutionary and expression analyses of the MADS-box gene family were performed across the *C. oleifera* genome for the first time. A total of 86 MADS-box genes (*ColMADS*) were identified, including 60 M-type and 26 MIKC members. More gene duplication events occurred in M-type subfamily (6) than that in MIKC subfamily (2), and *SEP-like* genes were lost from the MIKC^C^ clade. Furthermore, 8, 15, and 17 differentially expressed *ColMADS* genes (DEGs) were detected between three developmental stages of seed (S1/S2, S2/S3, and S1/S3), respectively. Among these DEGs, the *STK-like ColMADS12* and *TT16-like ColMADS17* were highly expressed during the seed formation (S1 and S2), agreeing with their predicted functions to positively regulate the seed organogenesis and oil accumulation. While *ColMADS57* and *ColMADS07* showed increasing expression level with the seed maturation (S2 and S3), conforming to their potential roles in promoting the seed ripening. In all, these results revealed a critical role of MADS-box genes in the *C. oleifera* seed development and oil accumulation, which will contribute to the future molecular breeding of *C. oleifera*.

## 1. Introduction

*Camellia oleifera* Abel. is one of the world-famous woody oil species for its seeds producing high amount of edible oil [1, 2]. The fatty acid content of tea-tree oil is comparable to that of olive oil, earning it the nickname “Oriental olive oil” [3, 4]. *C. oleifera* generally blooms in November. Nearly 150 days after pollination (DAP), that is, March of the next year, the ovule begins to develop [5]. By 250 DAP (July), the heart embryo is transformed into the torpedo-shaped embryo and cotyledons emerge. Until 365 DAP (November), fully developed seed is observed and accumulates high oil content (Figure S1). Its seed development period surpassed that of *C. chekiangoleosa* (about 180 days) [6], oil palm (150 days) [7], castor seed (60 days) [8], and certainly other herbaceous oil-seed plants (rape and sunflower). Up to now, key genes underpinning *C. oleifera* seed development and oil accumulation are not clear [3–5, 9].

MADS-box genes encode a kind of transcription factors (TFs) that play pivotal roles in seed development [10, 11]. The MADS-box TFs are characterized by the presence of 58-60 conserved amino acids known as MADS domain and are able to bind to the CArG-box *cis*-elements to regulate the expression of certain genes [12]. The name “MADS” was initially derived from the first letter of minichromosome maintenance 1 (MCM1) of yeast, AGAMOUS (AG) of *Arabidopsis*, DEFICIENS (DEF) of *Antirrhinum*, and serum response factor (SRF) of humans [13]. The MADS-box TF family is usually classified into two subfamilies: Type I (M-type) and Type II (MIKC). The M-type subfamily generally contains an SRF domain, including three groups: M*α*, M*β*, and M*γ*. The MIKC subfamily typically possesses SRF or MEF2 domains and contains two lineages: MIKC^∗^ and MIKC^C^ (superscript C denotes classic) [14]. MIKC^C^ genes have been widely studied in plants and subdivided into 13 groups, that is, *AG*/*SHP*, *AGL6*, *AGL12*, *AGL15*, *ANR1*, *AP1*, *AP3*/*PI*, *FLC*, *SEP*, *SOC1*, *SVP*, *TM8*, and *TT16* [15, 16].

In general, the seed development is primarily comprised of two phases: seed organogenesis and maturation [17, 18]. According to the classic ABCDE model, ovule identity is determined by C+D+E genes [19, 20]. In the model plant *A. thaliana*, *AGAMOUS* (*AG*) is C-class gene, and *SEEDSTICK* (*STK*), *SHATTERPROOF1* (*SHP1*), and *SHATTERPROOF2* (*SHP2*) are D-class genes, while E-class genes consist of *SEPALLATA1* (*SEP1*), *SEP2*, *SEP3*, and *SEP4* [19]. All these C/D/E-class genes belong to the MADS-box family and are coexpressed during the seed organogenesis [21–23]. In tomato, the MADS-box gene *AGL6* regulated the seed formation [24]. Recently, heterologous expression of *C. sinensis CsAGL9* in wild-type *Arabidopsis* resulted in early flowering and significantly reduced the number of seeds per pod [25].

It is worth noting that MADS-box genes are likely involved in seed oil accumulation too [26]. In *Arabidopsis*, oil accumulation begins at the embryonic cotyledon stage and continues until seed maturity [27]. Overexpression of the MADS-box gene *STK* promotes lipid synthesis, while *SEP3* had an antagonistic effect on *STK* to reduce the oil content of the seed [28]. Similarly, in canola, the MADS-box gene *Transparent Testa16* (*Bntt16*) takes part in the embryo development and lipid synthesis [29]. Silencing of *Bntt16* reduced the oil content and altered fatty acid composition of rape seed.

In this study, we firstly performed genome-wide analyses of the *C. oleifera* MADS-box (*ColMADS*) gene family. Subsequently, the expression patterns of *ColMADS* genes were compared between the three development stages of the seed (Figure S1). Finally, the key *ColMADS* genes participating in seed development and oil accumulation were identified based on comprehensive analyses of gene expression and function prediction. The obtained results will facilitate to understand the molecular regulatory mechanisms of seed development and oil accumulation in *C. oleifera*.

## 2. Results

### 2.1. Genome-Wide Identification and Phylogenetic Classification of ColMADS Genes

A total of 86 MADS-box genes (*ColMADS01*-*86*) were identified in *C. oleifera* genome based on BLASTP and HMMER analyses. The ColMADS TFs were sized from 66 aa (ColMADS64) to 492 aa (ColMADS62), with an average of 209 aa (Table S1). Their isoelectric points (PI) were between 4.53 (ColMADS49) and 11.03 (ColMADS48). Molecular weight (MW) varied from 7.3 kDa (ColMADS61) to 56.5 kDa (ColMADS62). The instability index (II) of 64 ColMADS TFs (74.41%) exceeded 40, indicating that most ColMADS TFs are susceptible to destabilization or degradation under certain conditions. Moreover, the grand average of hydropathicity (GRAVY) for 76 ColMADS TFs (88.37%) exhibited negative values, implying a higher degree of water solubility for most ColMADS TFs. Subcellular localization prediction revealed that 52 ColMADS TFs (60.47%) are localized in the nucleus, 17 in chloroplasts, 12 in the cytoplasm, 4 in mitochondria, and 1 in the peroxisome.

**Figure 1:**
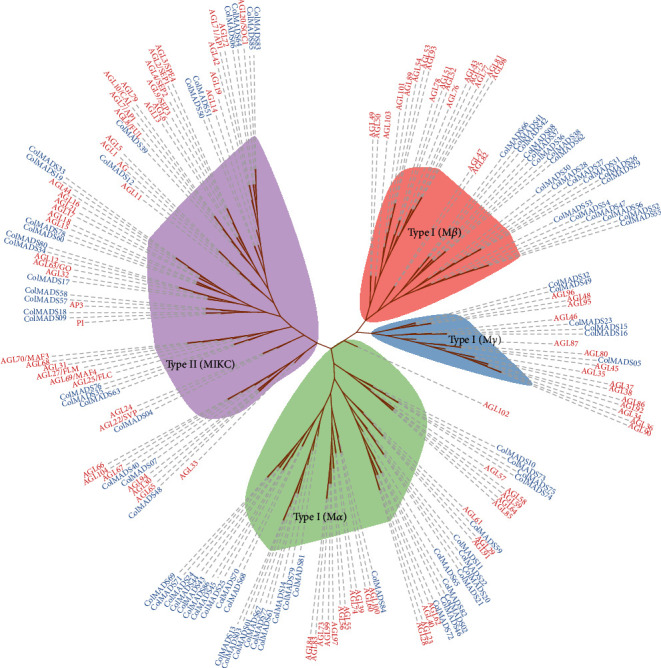
Maximum likelihood (ML) tree of MADS-box proteins in *C. oleifera* (blue) and *A. thaliana* (red). The tree reveals two subfamilies of the MADS-box gene family (i.e., Type I (M-type) and Type II (MIKC)). In M-type subfamily, branches of M*α*, M*β*, and M*γ* groups are shadowed in green, orange, and blue, respectively. MIKC subfamily branch is in purple.

**Figure 2:**
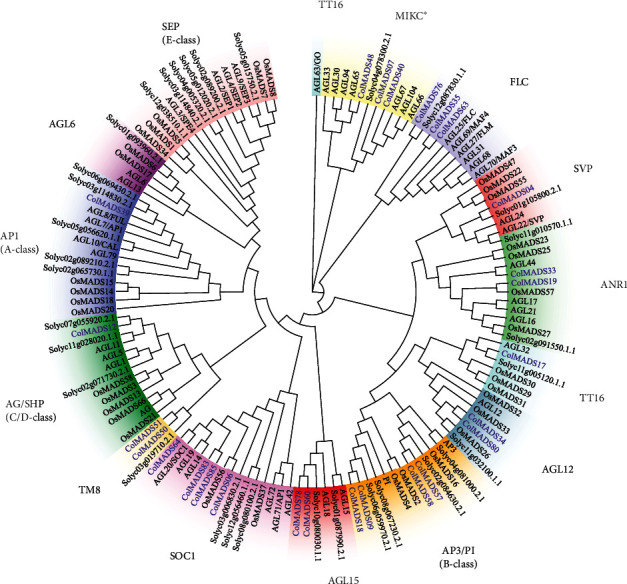
Maximum likelihood (ML) tree of MIKC MADS-box proteins in *C. oleifera*, *A. thaliana*, *Solanum lycopersicum*, and *Oryza sativa*. MIKC^∗^ clade and 13 known MIKC^C^ groups are highlighted in different colors.

To classify the 86 *ColMADS* genes, the phylogenetic relationship among *ColMADS* and *A. thaliana* MADS-box (*AtMADS*) (*A. thaliana*, 103) was analyzed. The maximum likelihood (ML) tree showed that 60 *ColMADS* genes were clustered in Type I (M-type) subfamily, while the rest 26 belonged to Type II (MIKC) subfamily (Figure 1). In the M-type subfamily, 34, 20, and 16 *ColMADS* genes were classified into the M*α*, M*β*, and M*γ* lineage, respectively. MIKC MADS-box TFs of tomato (MIKC-SolyMADS, 32), rice (MIKC-OsMADS, 34), and *Arabidopsis* (MIKC-AtMADS, 46) were further used to classify MIKC-ColMADS TFs (26). The phylogenetic tree was branched into 14 groups, corresponding to the 13 known MIKC^C^ groups and 1 MIKC^∗^ group (Figure 2). There were 1 A-class (*ColMADS39*), 4 B-class (*ColMADS09*, *18*, *57*, and *58*), and 1 C/D-class (*ColMADS12*) members, respectively. No E-class MADS-box genes and *AGL6-like* genes were detected. Three MIKC-ColMADS (*ColMADS07*, *40*, and *48*) were in the MIKC^∗^ group.

### 2.2. Genomic Synteny Analysis and Chromosome Mapping of *ColMADS* Genes

**Figure 3:**
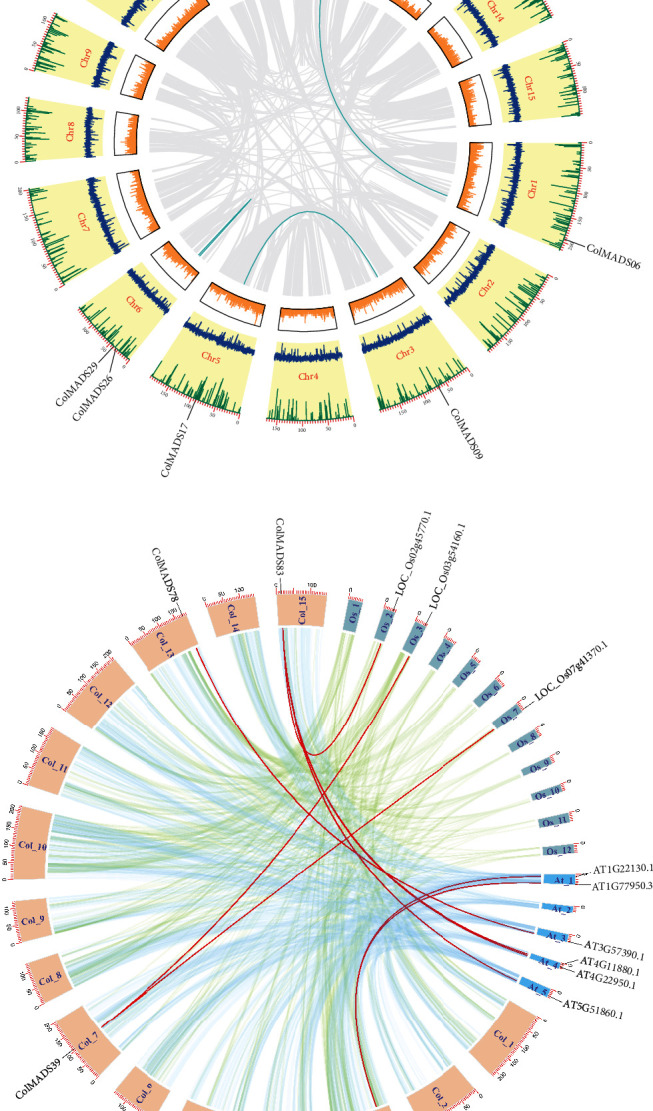
Synteny analysis of MADS-box genes within *C. oleifera* and between species. (a) Duplication events of *ColMADS* genes in *C. oleifera* genome. (b) Collinear relationships of MADS-box genes between *C. oleifera* and the other two species (*Arabidopsis* and rice). Note: (a) From the inner to outer, gray lines: synteny blocks; cyan lines: duplicated *ColMADS* gene pairs; heatmap: gene density profile; blue line plot: GC ratio; green line plot: *N* ratio. (b) Light blue and green lines: syntenic blocks between *C. oleifera*/*Arabidopsis* and *C. oleifera*/rice, respectively; red lines: collinear *ColMADS* gene pairs.

Gene duplication events among *ColMADS* genes were identified based on MCScanX analysis in TBtools software, revealing 3 pairs of segmental duplicated genes (Figure 3(a)). To ascertain the selection pressure on duplicated *ColMADS* genes, the Ka/Ks ratio of each pair of paralogous genes was estimated. It is noteworthy that the Ka/Ks value of all the 3 pairs of segmental duplicated genes was less than 1 (Table S2), implying strong purification selection on these duplicated genes. In addition, the estimated divergence time for the segmental duplicated gene pairs ranges from 1.15 to 1.45 million years ago (Mya). Furthermore, synteny analyses of *C. oleifera* versus two representatives (*A. thaliana* and rice) showed that 3 pairs of MADS-box orthologous genes between *C. oleifera*/rice and 6 pairs between *C. oleifera*/*A. thaliana* (Figure 3(b)).

**Figure 4:**
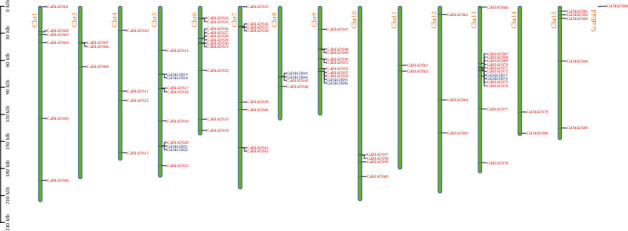
Chromosomal distribution of *ColMADS* genes. Each green bar represents one assembled chromosome, while the scaffold denotes the unassembled free fragments. Genes indicated by the blue are tandem duplication genes.

Chromosomal locations of 86 *ColMADS* genes displayed a highly uneven distribution of *ColMADS* genes across the genome (Figure 4). Chromosome 13 (Chr13) harbored the maximum number of *ColMADS* genes (13, 15.1%), followed by Chr6 (11, 12.8%), and Chr5 and Chr9 (both contain 10, 11.6%). One *ColMADS* gene (*ColMADS86*) was not located on any assembled 15 chromosomes, and none *ColMADS* genes were seen on Chr2. Based on the physical location (< 200 kb) and homology rate (> 80%) between genes in the same chromosome, five pairs of tandem duplicated genes were found (Table S2). Notably, all the tandem duplicated genes were the M-type members. With the exception of ColMADS55/56, the Ka/Ks value of these tandem duplicated genes is less than 1, and the divergence time is ranging from 0.21 to 3.43 Mya.

### 2.3. Gene and Protein Structure Analysis

**Figure 5:**
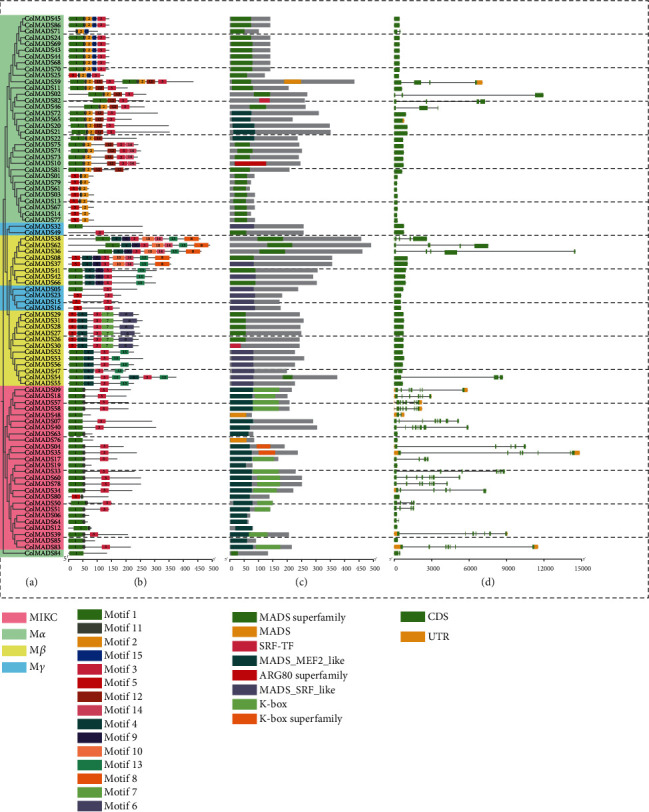
Protein domain and gene structure of *ColMADS* genes. (a) Phylogenetic relationship of *ColMADS* gene family. Three clades of Type I (M-type) and Type II (MIKC) are color-shadowed differently. (b) Protein motif composition of *ColMADS* genes. (c) Conserved protein domains of *ColMADS* genes. Seven domains and 15 motifs are boxed in different colors. (d) Exon-intron organization of *ColMADS* genes. CDS, UTR, and intron are, respectively, represented by green, yellow boxes, and black lines.

The phylogenetic tree of 86 ColMADS TFs was constructed based on protein sequences, and it was found that M-type and MIKC genes were obviously separated (Figure 5(a)). Fifteen conserved motifs (Motifs 1−15) were identified (Figure 5(b), Table S3) in all the 86 ColMADS TFs, among which Motifs 1, 3, and 5 were the most conserved. Motif 2 was specific to M*α*-ColMADS members. In addition, seven types of conserved domains, that is, MADS-MEF2*-like*, MADS superfamily, MADS-SRF*-like*, MADS, SRF-TF, K-box, and K-box superfamily, were detected (Figure 5(c)). The length of *ColMADS* genes ranged from 212 bp (*ColMADS61*) to 14,850 bp (*ColMADS35*). Exon numbers vary from 1 to 8 (Figure 5(d)), with the MIKC members containing more exons (average 4.5) than M-type members (average 1.4).

**Figure 6:**
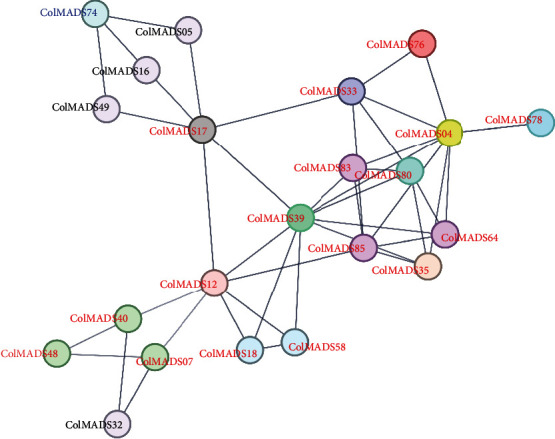
Putative interactions between *ColMADS* proteins. MIKC, M*γ*, and M*α* members are, respectively, indicated in red, black, and blue fonts.

A total of 1831 *cis*-elements were identified in *ColMADS* gene promotors, the most of which were light responsive elements (891), followed by phytohormone responsive elements (437) (Table S4). Protein-interactive analysis predicted that 22 out of 86 *ColMADS* TFs can interact with each other (Figure 6). *ColMADS12*, *39*, *17*, and *04* could interact with more than 6 *ColMADS* members, respectively. In particular, C/D-class *ColMADS*12 can interact with 7 MIKC-*ColMADS* TFs (*ColMADS*0*7*, *17*, *18*, *39*, *40*, *58*, and *85*).

### 2.4. Expression and Function of *ColMADS* Genes During Seed Development

**Figure 7:**
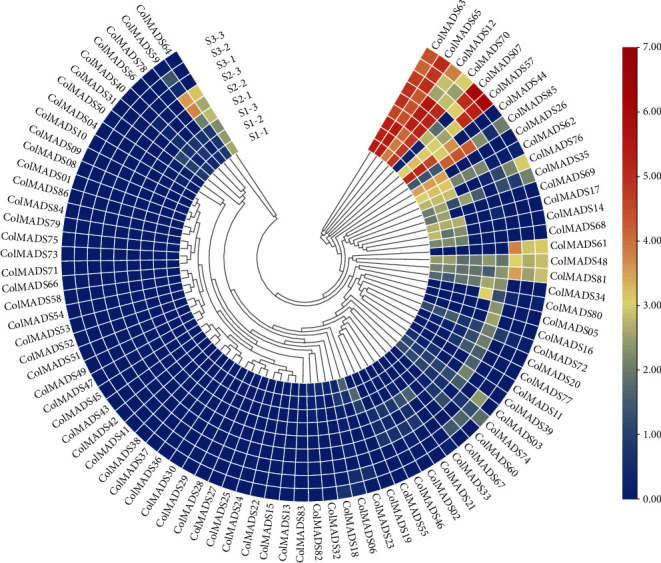
Expression profile of *ColMADS* genes in *C. oleifera* seed. FPKM values of 86 *ColMADS* genes were calculated using transcriptome data with normalization. Log2 (FPKM value) is displayed from low (blue) to high (red), indicating expression level. S1, S2, and S3 correspond, respectively, to three developmental stages (i.e., 150 DAP (March), 250 DAP (July), and 365 DAP (November)), with three biological replicates at each stage.

To investigate the function of MADS-box genes in *C. oleifera* seed development, expression patterns of all the 86 *ColMADS* genes were examined based on transcriptome data of three seed development stages (Figure S1), that is, March (S1-150 DAP), July (S2-250 DAP), and November (S3-365 DAP). More than half of *ColMADS* genes (52/86, 60.5%) were expressed in seed, but the rest 34 genes were not active at any of the three stages (Figure 7). Among these 34 genes that were not expressed, 6 were duplicated genes (*ColMADS09*, *15*, *22*, *29*, *43*, and *73*). *ColMADS22* and *ColMADS73* were truncated compared to their paralogous genes (Figure 5). In addition, genes belonging to the AP1 (*ColMADS39*) and SVP (*ColMADS04*) group were silenced in seeds.

According to the criteria of log2 (fold change value) *≥* 1 or ≤ −1 and adjusted *p* value (*p*adj) < 0.05, a total of 8, 15, and 17 differentially expressed genes (DEGs) were detected, respectively, between S1/S2, S2/S3, and S1/S3 (Table S5). Five genes (*ColMADS07*, *12*, *34*, *55*, and *60*) were upregulated, while 3 (*ColMADS39*, *65*, and *76*) were downregulated between S1/S2. Whereas 7 genes (*ColMADS07*, *48*, *57*, *61*, *65*, *76*, and *81*) were upregulated, 8 (*ColMADS12*, *17*, *34*, *39*, *40*, *62*, *64*, and *70*) were downregulated between S2/S3. In terms of DGEs between S1/S3, 5 genes (*ColMADS07*, *48*, *57*, *76*, and *81*) were upregulated, 12 genes (*ColMADS12*, *17*, *31*, *34*, *39*, *40*, *44*, *62*, *64*, *65*, *69*, and *70*) were downregulated.

The edible oil is gradually accumulated from S2 to S3 and reaches to the highest level at S3 in *C. oleifera* seed (Figure S1). In order to explore the potential functions of the 15 DEGs between S2/S3, they were annotated by blast to *Arabidopsis* on the TAIR website (http://www.arabidopsis.org/). The results showed that *ColMADS12* was D-class gene (*STK-like*) and essential to initiate seed organogenesis (Table 1). Similar expression pattern was seen in another 7 DEGs between S2/S3 (i.e., *ColMADS17*, *34*, *39*, *40*, *62*, *64*, and *70*), all showing lower expression level at S3 compared to that of S1 and S2. *ColMADS17* (*TT16-like*) may be involved in the ovule development. Both of *ColMADS62* (*AGL47-like*) and *ColMADS70* (*AGL62-like*) likely participated in the endosperm development. In addition, the function prediction of upregulated genes between S2/S3 suggested that *ColMADS07* (*AtAGL67-like*) modulated lipid accumulation and seed germination. *ColMADS57* showed homology with *AtAP3*, which could affect fruit ripening and seed maturation. *ColMADS61* was homologous to *AtAGL23* and played a role in regulating chloroplasts development of seeds.

### 2.5. Quantitative real-time PCR (qRT-PCR) Validation of Transcriptome Analysis

**Figure 8:**
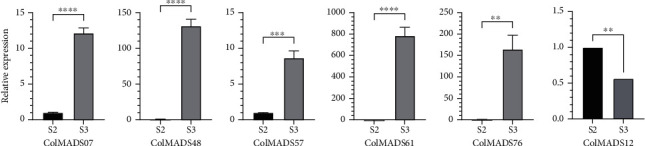
qRT-PCR analyses of differently expressed *ColMADS* genes at S2 and S3. Error bars indicate standard deviation of three biological replicates. Significant differences are shown with ^∗∗^*p* ≤ 0.01, ^∗∗∗^*p* ≤ 0.001, and ^∗∗∗∗^*p* ≤ 0.0001.

The DEGs identified between S2 (July) and S3 (November) were further validated by qRT-PCR. Since the FPKM values of the 15 DEGs were low (FPKM < 50) in S2/S3, to obtain reliable result, 6 DEGs with FPKM values > 10 (i.e., *ColMADS07*, 12, *48*, *57*, *61*, and and *76*) were selected for qRT-PCR analysis. *Actin* and *CoEF1α* were used separately as the reference gene for dual verification. The results from two control genes were consistent and revealed that all these 6 genes were DEGs between S2/S3 (Figure 8). As expected, the expression levels of the 5 genes (*ColMADS07*, *48*, *57*, *61*, and *76*) were found to be higher at S3 compared to S2, while *ColMADS12* was expressed at lower levels in S3 than S2, which is consistent with the transcription data.

## 3. Discussion

### 3.1. Genome-Wide Characterization of MADS-Box Genes in *C. oleifera*

In this study, we identified a total of 86 MADS-box genes across the *C. oleifera* genome for the first time (Table S1). Uneven chromosome distribution of *ColMADS* genes was observed (Figure 4), just as shown in *Eucommia* [14]. The size of *C. oleifera* MADS-box family (86) is comparable to its congeneric species *C. sinensis* (83) and *C*. *chekiangoleosa* (89) [30, 31]. It has been suggested that the genome size is related to the number of MADS-box genes [14]. The similar genome size of *C. oleifera* (2.95 Gb) [9], *C. sinensis* (3.26 Gb) [32], and *C*. *chekiangoleosa* (2.73 Gb) [33] may account for their approximate number of MADS-box genes. But we should keep in mind that complex historical events (e.g., gene duplication and loss) can affect the MADS-box gene family size too. In *C. oleifera*, eight gene duplicated events were detected and most of duplication events (6/8) occurred in the M-type subfamily (Figure 3, Table S2). Interestingly, the proportion of Type I MADS-box genes in *C. oleifera* (60/86, 69.8%) was also significantly higher than that in its closely related species (i.e., *C. sinensis* (47/83, 56.6%) [30] and *C. chekiangoleosa* (38/89, 42.7%)) [31]. The higher duplication rate (or lower gene loss rate) may lead to the expansion of M-type subfamily in *C. oleifera* (Figure 1).

The MIKC genes contained much more exons (average 4.5) than the M-type members (average 1.4, Figure 5), as revealed in *Eucommia* [14]. MIKC^C^ MADS-box genes have been widely studied and revealed conserved functions in plants [11–13]. Phylogenetic analysis showed that 23 MIKC^C^*ColMADS* genes were categorized into 11 groups (Figure 2). However, we failed to find the *SEP-like* genes in *C. oleifera*. It is worth noting that there is one *SEP-like* gene (*CsMADS64*) in *C. sinensis* [30] and five *SEP-like* genes (*CchMADS11*, *16*, *45*, *62*, and *82*) in *C. chekiangoleosa* [31]. *SEP-like* genes are E-class genes of the ABCDE model and are essential for fruit and seed development [13, 34]. Recent study on *C. sinensis* also showed that the *SEP-like* gene *CsMADS64* (named *CsAGL9*) played key roles in seed setting [25]. It is very common that seed abortion in *C. oleifera* [2, 3], but rarely seen in *C. chekiangoleosa* and *C. sinensis*. Missing *SEP-like* genes may partly explain the low rate of seed setting in *C. oleifera* in the wild.

### 3.2. Potential Roles of MADS-Box Genes in *C. oleifera* Seed Development

The expression of 86 *ColMADS* genes was analyzed in details between three developmental stages of seed. We found that a few genes (e.g., *ColMADS07*) had expression differences in the different biological replications (Figure 7). The underlying genetic and physiological variations may lead to the changes of fruit development rate in different individuals, resulting in different gene expressions [2, 3]. A total of eight *ColMADS* genes (i.e., *ColMADS12*, *17*, *34*, *39*, *40*, *62*, *64*, and *70*) were highly expressed in ovule and significantly reduced the expression level with seed development (Table S5). Among them, there was a homologue of *AtSTK*, the D-class gene of the ABCDE model in *Arabidopsis* [12, 13]. D-class genes determined the identity of ovule and regulated the seed organogenesis [19, 20]. Previous studies have revealed that the *STK-like* genes controlled seed setting in tomato, Chinese chestnut, *Jatropha curcas*, and so on [35–37]. It is, thus, reasonable to speculate that *ColMADS12* acts as the D-class *STK-like* gene to regulate the seed organogenesis of *C. oleifera*.

Moreover, *ColMADS17* was clustered in the *TT16* group and homologous to *AtTT16* (Figure 3, Table 1). *TT16-like* genes have been reported to control the seed formation of tomato [38]. In rice, *TT16-like* genes participated in the seed organogenesis and the proanthocyanidin biosynthesis of the seed coat [39]. The downregulation of *Bntt16* gene by RNA interference (RNAi) in canola also caused dwarf phenotypes with a decrease in the number of seeds [29]. Therefore, it is also possible for the *TT16-like* gene *ColMADS17* to function in the seed formation of *C. oleifera*. Further functional verification of these *STK-like* and *TT16-like* genes (*ColMADS12* and *17*) is required urgently to understand their roles in the seed setting of *C. oleifera*.

The seed maturation lasted from 250 DAP (S2, July) to 365 DAP (S3, November) in *C. oleifera* (Figure S1). Between S2 and S3, seven upregulated genes (*ColMADS07*, *48*, *57*, *61*, *65*, *76*, and *81*) were uncovered (Figures 7 and 8, Table S5). It was previously reported that *CchMADS39* and *CchMADS55* were highly expressed in *C. chekiangoleosa* seeds [31]. We blasted the protein sequences of these two genes with those of 86 MADS-box genes in *C. Oleifera*. The result showed that *CchMADS39* and *CchMADS55* were both homologous to *ColMADS07.* As expected, *ColMADS07* displayed the highest expression in mature *C. oleifera* seeds, which was consistent with the finding in *C. chekiangoleosa* [31]. *ColMADS07* was the homology of *AtAGL67* (Table 1) and could interact with the *STK-like* gene *ColMADS12* (Figure 6). Ectopic expression of the grape *AGL67-like* gene (*VvMADS45*) in tomato significantly increased the seed size [40]. In addition, *ColMADS57* was homologous to *Solyc02g084630* (*TM6*) of tomato (Figure 2). The *TM6* gene is highly expressed in seed and plays a crucial role in fruit ripening in tomato [41]. An increased expression level of the *TM6-like* gene (*VcMADS58*) was also observed during blueberry ripening [42]. Collectively, these two genes (*ColMADS57* and *07*) likely participated in the seed maturation of *C. oleifera*.

### 3.3. The Relationship Between MADS-Box Genes and Tea-Tree Oil Accumulation

In *C. oleifera*, the oil accumulation is accompanied by seed ripening [2–4], which is similar to that of *A. thaliana* [27]. The tea-tree oil is composed of palmitic acid (C16:0), stearic acid (C18:0), oleic acid (C18:1), linoleic acid (C18:2), and linolenic acid (C18:3), among which the oleic acid content increased the most during seed maturation [26]. The *Bntt16* gene was highly expressed in the embryo of canola, and *tt16* RNAi transgenic seeds had fewer oil bodies than the wild-type plants [29]. Similarly, the *TT16-like* gene *ColMADS17* of *C. oleifera* was highly expressed in the stage of ovule formation (Figure 7), implying a putative conservative function of *TT16*-*like* genes in canola and *C. oleifera*.

The latest study by He et al. revealed the gene regulatory network antagonistically orchestrated by *STK* and *SEP3* to govern the seed oil accumulation in *Arabidopsis* [28]. *STK* was highly expressed in the developing embryo and promoted the oil accumulation, especially the oleic acid content in the seed. But SEP3 could interact with STK and weakened the binding ability of STK to downstream genes (e.g., *MYB5* and *SFAR4*), thus inhibiting seed oil accumulation [28]. Notably, the *STK-like* gene *ColMADS12* of *C. oleifera* displayed the highest level of expression at the seed formation stage (Figure 7, Table S5), consistent with the expression pattern of *STK* in *Arabidopsis* [28]. This suggested that *ColMADS12* might play a double role in positively regulating the seed development and oil accumulation of *C. oleifera*. Meanwhile, the loss of *SEP*-*like* genes from *C. oleifera* (Figure 2) seemed to be a natural *sep3* mutant of *Arabidopsis* [28], which liberated the STK for downstream lipid synthesis genes to accumulate high amount of oil in the *C. oleifera* seed.

## 4. Materials and Methods

### 4.1. Identification and Classification of MADS-Box Genes in *C. oleifera*

We downloaded the *C. oleifera* genome data from the NCBI database (https://www.ncbi.nlm.nih.gov/datasets/genome/GCA_022316695.1/) on March 8, 2023. Meanwhile, nucleotide and amino acid sequences of MADS-box genes in *A. thaliana* and *Oryza sativa* were separately downloaded from http://rice.plantbiology.msu.edu and https://www.arabidopsis.org/ (Additional Data 1). ColMADS genes were identified by two independent methods. Firstly, all the protein sequences of *C. oleifera* were reciprocally blasted [43] with *A. thaliana* and *O. sativa* MADS-box proteins. The best hits with a score value ≥ 100 and an *e* value ≤ 1e^−10^ were determined as candidate *ColMADS* genes [14]. Secondly, we downloaded the conserved MEF2 (PF09047) and SRF (PF00319) domains from Pfam [44]. HMMER v.3.0 was used to identify candidate ColMADS proteins by hidden Markov model (HMM) search [14]. Finally, the candidate sequences of *C. oleifera* shared by the two aforementioned methods were selected as nonredundant ColMADS genes. Then, these sequences were further verified in the Conserved Domain Database of NCBI (CDD) (http://www.ncbi.nlm.nih.gov/cdd/) and Simple Modular Architecture Research Tool (SMART) (http://smart.embl-heidelberg.de/) [45]. In addition, the MWs, PI, II, protein length, and GRAVY of ColMADS TFs were calculated using the ExPasy website (https://web.expasy.org/protparam/). The subcellular location of ColMADS TFs was also predicted by WoLF PSORT (https://www.genscript.com/wolf-psort.html?src=leftbar).

To classify the MADS-box TF family of *C. oleifera*, *AtMADS* proteins (Table S6) were aligned with ColMADS proteins by the ClustalW program [46]. An unrooted ML phylogenetic tree was constructed in MEGA11.0 [47], with 1000 bootstrap replicates under the J+G+I+F model. The resulted ML tree was visualized in Chiplot (https://www.chiplot.online/tvbot.html). Moreover, MIKC protein sequences of *O. sativa* (MIKC-OsMADS) and *Solanum lycopersicum* (MIKC-SolyMADS) were added (Table S6) for ML tree construction to categorize Type II (MIKC) MADS-box TFs of *C. oleifera.* The names of ColMADS phylogenetic groups were referred to the MADS-box classification system of *Arabidopsis*/*Solanum* [15, 16].

### 4.2. Genome Synteny Analysis and Chromosome Localization

The genome annotation file of *C. oleifera* was retrieved from the NCBI database (https://www.ncbi.nlm.nih.gov/datasets/genome/GCA_022316695.1/) (accessed on March 8, 2023). The MCScanX software [48] was used to perform intraspecies synteny analysis among the 86 *ColMADS* genes, with default parameters. The tandem and segment duplication events of *ColMADS* genes were detected by using TBtools [49]. Tandem duplication genes were verified based on the following criteria: (1) on the same chromosome, (2) the homology between genes greater than 80%, and (3) the physical distance less than 200 kb [50]. In the same way, the interspecies synteny analysis between *C. oleifera* and two representative plants (i.e., *A. thaliana* and *Oryza sativa*) was also conducted with MCScanX [48]. The homology of MADS-box genes among *C. oleifera*, *A. thaliana*, and *O. sativa* was visualized in TBtools [49]. Moreover, chromosomal locations of all *ColMADS* genes were retrieved from the GFF3 annotation file [9]. The physical positions of 86 *ColMADS* genes were then mapped on the assembled 15 chromosomes of *C. oleifera* by TBtools [49].

### 4.3. Gene Structure, Cis-Element, and Protein-Interaction Prediction of *ColMADS* Genes

The *ColMADS* gene structures (i.e., exon–intron organizations) were analyzed by comparing the coding (CDS) and genomic sequences in the TBtools [49]. Conserved motifs of the ColMADS TFs were identified through the online website MEME (https://meme-suite.org/). InterPro online tool (https://www.ebi.ac.uk/interpro/) was further used to explore the potential functions of motifs. Conserved protein domains of the ColMADS TFs were also confirmed in the NCBI-CDD website (https://www.ncbi.nlm.nih.gov/Structure/bwrpsb/bwrpsb.cgi) (accessed on May 27, 2023).

The promoter sequences of 2000 bp upstream from each *ColMADS* CDS were obtained by TBtools [49] from the genome data. The PlantCARE online program (http://bioinformatics.psb.ugent.be/webtools/plantcare/html/) (accessed on May 20, 2023) was used to search for putative *cis*-acting elements. The *cis*-elements related to plant growth and development, phytohormone responsive, stress responsive, and light responsiveness were analyzed for each *ColMADS* gene. Moreover, to reveal the ColMADS TFs interaction network, protein interaction analysis was performed by submitting ColMADS TFs to the STRING website (https://cn.string-db.org/) (accessed on May 29, 2023).

### 4.4. Expression Analysis of ColMADS Genes by RNA-Seq

To reveal the expression patterns of MADS-box genes in consecutive seed development stage of *C. oleifera*, we collected seed samples from three different time points (Figure S1: March-S1, 150 DAP; July-S2, 250 DAP; November-S3, 365 DAP). At each stage, three biological replicates were sampled from Xiaokeng Forest Farm (24°42′N, 113°48′E) in Guangdong, China in 2021–2022. Seeds from 15-year-old *C. oleifera* were collected and frozen in liquid nitrogen immediately and then stored at −80°C until used.

RNeasy Plant Mini Kit (74904, Qiagen, German) was used to extract total RNA. RNA-seq library was constructed for sequencing by following the manuals at the Illumina platform of BGI Technologies Corporation (Shenzhen, China). About 6 Gb of clean data were produced for each sample. The expression level of each *ColMADS* gene was quantified by these transcriptome reads in RSEM software [51]. FPKM (fragments per kilobase of transcript per million mapped) values of *ColMADS* genes were calculated by the uniquely mapped reads [52]. For each development stage (S1, S2, ad S3), reads from three individuals (biological replicates) were batched into one dataset. Then, TBtools [49] software was used to draw heat maps based on FPKM data. Differential expression analyses were performed between S1/S2, S2/S3, and S1/S3, respectively, by using DESeq R package v1.10.1 [53]. Genes with Log2 (fold change value) ≥ 1 or ≤ −1, and *p*adj < 0.05 were considered as DEGs [14]. The newly generated seed RNA-seq data herein were deposited in the SRA database (accession number: PRJNA1002891).

### 4.5. qRT-PCR Validation of Transcriptome Data

The six DEGs (*ColMADS07*, *12*, *48*, *57*, *61*, and *76*) between S2/S3 were selected for qRT-PCR validation. SuperScript™ IV VILO™ Master Mix (Thermo Fisher, USA) was used for the cDNA synthesis according to the manufacturer's instructions. Primers were designed by Primer3 (http://bioinfo.ut.ee/primer3-0.4.0/primer3/) based on CDS sequences and synthesized by Sangon Biotech Co. Ltd. (Shanghai, China) (Table S7). The specificity of primers was evaluated using the *C. oleifera* genome data in TBtools [49].

qRT-PCR analysis was conducted according to the manual of Hieff qRT-PCR SYBR Green Master Mix (YEASEN, Shanghai, China) on a LightCycler 480 II Real-Time PCR Platform (Roche, Germany). Each reaction mixture (20 *μ*L) contained 1.0 *μ*L cDNA, 0.4 *μ*L forward primer, 0.4 *μ*L reverse primer, 10.0 *μ*L of qRT-PCR Mix, and 8.2 *μ*L of ddH_2_O. The relative expression levels of each gene were calculated by the 2^−ΔΔCT^ method [54, 55], and the mean ± standard deviation (SD) values were estimated from three independent biological replicates. *Actin* and *CoEF1α* were independently used as the inner reference genes for data normalization following the study of Bao [56] and Wu et al. [57]. Analysis of variance (ANOVA) and Student's *t*-test were performed in R software for measuring the difference of gene expression level [58, 59]. Subsequently, qRT-PCR analyses of differently expressed *ColMADS* genes were visualized using GraphPad Prism v9.1 software (https://www.graphpad.com).

## 5. Conclusions

In this study, a total of 86 MADS-box genes were identified in *C. oleifera*, which can be divided into M*α* (34), M*β* (20), M*γ* (6), MIKC^∗^ (3), and MIKC^C^ (23) lineages. Six gene duplication events occurred in the Type I subfamily (M-type), while the *SEP* clade has been lost from the MIKC^C^ lineage. Further comparative transcriptome analyses from three development stages of seed revealed 8, 15, and 17 differentially expressed *ColMADS* genes between S1/S2, S2/S3, and S1/S3, respectively. The expression patterns of *ColMADS12* (*STK-like*), *ColMADS17* (*TT16-like*), *ColMADS57* (*TM6-like*), and *ColMADS07* (*AGL67-like*) were consistent with their predicted functions in regulating the seed development. In addition, *ColMADS12* and *ColMADS17* may also positively control the seed oil accumulation. Collectively, this study laid an important foundation for future in-depth study to uncover the molecular regulation mechanisms of seed development and oil accumulation in *C. oleifera*.

## Figures and Tables

**Table 1:** Function annotation of *ColMADS* genes involved in seed development.

**Gene name**	**Ortholog in *Arabidopsis***	**Other names**	**Putative function**
ColMADS07	AT1G77950	AGL67	Modulate lipid accumulation and seed germination
ColMADS12	AT4G09960	STK	Control seed size and cell cycle progression
ColMADS17	AT5G23260	TT16	Control ovule development
ColMADS57	AT3G54340	AP3	Affect fruit ripening and seed maturation
ColMADS61	AT1G65360	AGL23	Regulating chloroplasts development of seeds
ColMADS62	AT5G55690	AGL47	Involved in early endosperm development
ColMADS70	AT5G60440	AGL62	Promote endosperm development

## Data Availability

Transcriptome data derived from seeds of *Camellia oleifera* are deposited in the Sequence Read Archive (accession number: PRJNA1002891). The datasets supporting the conclusions are included in the article/Supplementary Materials.

## Supporting Information

Supporting Information 1 Figure S1. A schematic of seed development in *Camellia oleifera*.

Supporting Information 2 Table S1. Detailed information of the MADS-box genes in *Camellia oleifera*.

Supporting Information 3 Table S2. Gene duplication events in the MADS-box gene family of *Camellia oleifera*.

Supporting Information 4 Table S3. Conserved motifs in ColMADS proteins.

Supporting Information 5 Table S4. The *cis*-elements in promoter regions of *ColMADS* genes.

Supporting Information 6 Table S5. Differentially expressed *ColMADS* genes between S1/S2 (a), S2/S3 (b) and S1/S3 (c).

Supporting Information 7 Table S6. Name and ID of MADS-box genes in *Arabidopsis thaliana* (a), *Oryza sativa* (b), and *Solanum lycopersicum* (c) used in this study.

Supporting Information 8 Table S7. Primers used in this study for qRT-PCR analysis.

Supporting Information 9 Additional Data 1: the nucleotide and amino-acid sequences of MADS-box genes in *Camellia oleifera*, *Arabidopsis thaliana*, *Oryza sativa*, and *Solanum lycopersicum*.

## References

[1] Quan W., Wang A., Gao C., Li C. (2022). Applications of Chinese *Camellia oleifera* and its by-products: a review. *Frontiers in Chemistry*.

[2] Ye Z., Wu Y., Muhammad Z. U. H. (2020). Complementary transcriptome and proteome profiling in the mature seeds of *Camellia oleifera* from Hainan Island. *PLoS One*.

[3] Ji K., Song Q., Yu X. (2022). Hormone analysis and candidate genes identification associated with seed size in *Camellia oleifera*. *Royal Society Open Science*.

[4] Gong W., Song Q., Ji K. (2020). Full-length transcriptome from *Camellia oleifera* seed provides insight into the transcript variants involved in oil biosynthesis. *Journal of Agricultural and Food Chemistry*.

[5] Liao T., Yuan D. Y., Zou F. (2014). Self-sterility in *Camellia oleifera* may be due to the prezygotic late-acting self-incompatibility. *PLoS One*.

[6] Wang Z., Huang B., Ye J. (2022). Comparative transcriptomic analysis reveals genes related to the rapid accumulation of oleic acid in *Camellia chekiangoleosa*, an oil tea plant with early maturity and large fruit. *Plant Physiology and Biochemistry*.

[7] Cao J. Q., Wang W., Xu X., Li S. Y., Zheng Y. S., Li D. D. (2023). Identification and analysis of MADS-box genes expressed in the mesocarp of oil palm fruit (*Elaeis guineensis* Jacq.). *Biochemical Genetics*.

[8] Huang F., Peng M., Chen X. (2018). cDNA-AFLP analysis of transcript derived fragments during seed development in castor bean (*Ricinus communis* L.). *Biotechnology & Biotechnological Equipment*.

[9] Lin P., Wang K., Wang Y. (2022). The genome of oil-camellia and population genomics analysis provide insights into seed oil domestication. *Genome Biology*.

[10] Theissen G., Ruempler F., Gramzow L. (2018). Array of MADS-box genes: facilitator for rapid adaptation?. *Trends in Plant Science*.

[11] Kappel S., Ruempler F., Theissen G. (2023). Cracking the floral quartet code: how do multimers of MIKC^C^-type MADS-domain transcription factors recognize their target genes?. *International Journal of Molecular Sciences*.

[12] Ng M., Yanofsky M. F. (2001). Function and evolution of the plant MADS-box gene family. *Nature Reviews. Genetics*.

[13] Parenicova L., de Folter S., Kieffer M. (2003). Molecular and phylogenetic analyses of the complete MADS-box transcription factor family in *Arabidopsis*: new openings to the MADS world. *Plant Cell*.

[14] Zhang X., Wang X., Pan L., Guo W., Li Y., Wang W. (2023). Genome-wide identification and expression analysis of MADS-box transcription factors reveal their involvement in sex determination of hardy rubber tree (*Eucommia ulmoides* oliv.). *Frontiers in Genetics*.

[15] Ren Z., Yu D., Yang Z. (2017). Genome-wide identification of the MIKC-type MADS-box gene family in *Gossypium hirsutum* L. unravels their roles in flowering. *Frontiers in Plant Science*.

[16] Wu Y., Zhang C., Wu W., Li W., Lyu L. (2021). Genome-wide identification and analysis of the MADS-box gene family and its potential role in fruit ripening in black raspberry (*Rubus occidentalis* L.). *Journal of Berry Research*.

[17] Savadi S. (2018). Molecular regulation of seed development and strategies for engineering seed size in crop plants. *Plant Growth Regulation*.

[18] Su L., Wan S., Zhou J., Shao Q. S., Xing B. (2021). Transcriptional regulation of plant seed development. *Physiologia Plantarum*.

[19] Zhang J., Wang Y., Naeem M. (2019). An AGAMOUS MADS-box protein, SlMBP3, regulates the speed of placenta liquefaction and controls seed formation in tomato. *Journal of Experimental Botany*.

[20] Zhang S., Yao J., Wang L. (2022). Role of grapevineSEPALLATA‐relatedMADS‐box geneVvMADS39in flower and ovule development. *The Plant Journal*.

[21] Gonzalez-Morales S. I., Chavez-Montes R. A., Hayano-Kanashiro C. (2016). Regulatory network analysis reveals novel regulators of seed desiccation tolerance in *Arabidopsis thaliana*. *Proceedings of the National Academy of Sciences of the United States of America*.

[22] Kang I.-H., Steffen J. G., Portereiko M. F., Lloyd A., Drews G. N. (2008). The AGL62 MADS domain protein regulates cellularization during endosperm development in *Arabidopsis*. *Plant Cell*.

[23] Masiero S., Colombo L., Grini P. E., Schnittger A., Kater M. M. (2011). The emerging importance of type I MADS box transcription factors for plant reproduction. *Plant Cell*.

[24] Molesini B., Dusi V., Pennisi F., Pandolfini T. (2020). How hormones and MADS-box transcription factors are involved in controlling fruit set and parthenocarpy in tomato. *Genes*.

[25] Wang L., Qian Y., Wu L., Wei K., Wang L. (2024). The MADS-box transcription factor CsAGL9 plays essential roles in seed setting in *Camellia sinensis*. *Plant Physiology and Biochemistry*.

[26] Yang J., Chen B., Manan S. (2022). Critical metabolic pathways and SAD/FADs, WRI1s, and DGATs cooperate for high-oleic acid oil production in developing oil tea (*Camellia oleifera*) seeds. *Horticulture Research*.

[27] Kazaz S., Barthole G., Domergue F. (2020). Differential activation of partially redundant *Δ*9 stearoyl-ACP desaturase genes is critical for omega-9 monounsaturated fatty acid biosynthesis during seed development in *Arabidopsis*. *Plant Cell*.

[28] He S., Min Y., Liu Z. (2024). Antagonistic MADS-box transcription factors SEEDSTICK and SEPALLATA3 form a transcriptional regulatory network that regulates seed oil accumulation. *Journal of Integrative Plant Biology*.

[29] Deng W., Chen G., Peng F., Truksa M., Snyder C. L., Weselake R. J. (2012). Transparent Testa16 plays multiple roles in plant development and is involved in lipid synthesis and embryo development in canola. *Plant Physiology*.

[30] Zhang Z. B., Jin Y. J., Wan H. H., Cheng L., Feng Z. G. (2021). Genome-wide identification and expression analysis of the MADS-box transcription factor family in *Camellia sinensis*. *Journal of Applied Genetics*.

[31] Zhou P., Qu Y., Wang Z. (2023). Gene structural specificity and expression of MADS-box gene family in *Camellia chekiangoleosa*. *International Journal of Molecular Sciences*.

[32] Wang X., Feng H., Chang Y. (2020). Population sequencing enhances understanding of tea plant evolution. *Nature Communications*.

[33] Shen T. F., Huang B., Xu M. (2022). The reference genome of *Camellia chekiangoleosa* provides insights into *Camellia* evolution and tea oil biosynthesis. *Horticulture Research*.

[34] Cheng Y., He P., Jiang L., Liu S., Zhou Y. (2019). Identification and characterization of a SEPALLATA-like MADS-box gene from cucumber (*Cucumis sativus* L.). *Notulae Botanicae Horti Agrobotanici Cluj-Napoca*.

[35] Mizzotti C., Ezquer I., Paolo D. (2014). SEEDSTICK is a master regulator of development and metabolism in the *Arabidopsis* seed coat. *PLoS Genetics*.

[36] Gao Y. R., Sun J. C., Sun Z. L. (2020). The MADS-box transcription factor CmAGL11 modulates somatic embryogenesis in Chinese chestnut (*Castanea mollissima* Blume). *Journal of Integrative Agriculture*.

[37] Xu G., Huang J., Lei S. K., Sun X. G., Li X. (2019). Comparative gene expression profile analysis of ovules provides insights into *Jatropha curcas* L. ovule development. *Scientific Reports*.

[38] de Martino G., Pan I., Emmanuel E., Levy A., Irish V. F. (2006). Functional analyses of two tomato *APETALA3* genes demonstrate diversification in their roles in regulating floral development. *Plant Cell*.

[39] Yang X., Wu F., Lin X. (2012). Live and let die - the B_sister_ MADS-box gene *OsMADS29* controls the degeneration of cells in maternal tissues during seed development of rice (*Oryza sativa*). *PLoS One*.

[40] Sun X. M., Zhang S. L., Li X. M. (2020). A MADS-box transcription factor from grapevine, VvMADS45, influences seed development. *Plant Cell, Tissue and Organ Culture (PCTOC)*.

[41] Cao X., Liu X., Wang X. (2019). B-class MADS-box *TM6* is a candidate gene for tomato male sterile-15(26). *Theoretical and Applied Genetics*.

[42] Wang X., Huang Q., Shen Z. (2023). Genome-wide identification and analysis of the MADS-box transcription factor genes in blueberry (*Vaccinium* spp.) and their expression pattern during fruit ripening. *Plants-Basel*.

[43] Camacho C., Coulouris G., Avagyan V. (2009). BLAST+: architecture and applications. *BMC Bioinformatics*.

[44] Mistry J., Chuguransky S., Williams L. (2021). Pfam: the protein families database in 2021. *Nucleic Acids Research*.

[45] Letunic I., Doerks T., Bork P. (2012). SMART 7: recent updates to the protein domain annotation resource. *Nucleic Acids Research*.

[46] Thompson J. D., Gibson T. J., Higgins D. G. (2003). Multiple sequence alignment using ClustalW and ClustalX. *Current Protocols in Bioinformatics*.

[47] Tamura K., Stecher G., Kumar S. (2021). MEGA11 molecular evolutionary genetics analysis version 11. *Molecular Biology and Evolution*.

[48] Wang Y. P., Tang H. B., DeBarry J. D. (2012). MCScanX: a toolkit for detection and evolutionary analysis of gene synteny and collinearity. *Nucleic Acids Research*.

[49] Chen C., Chen H., Zhang Y. (2020). TBtools: an integrative toolkit developed for interactive analyses of big biological data. *Molecular Plant*.

[50] Gu Z., Cavalcanti A., Chen F., Bouman P., Li W. (2002). Extent of gene duplication in the genomes of Drosophila, nematode, and yeast. *Molecular Biology and Evolution*.

[51] Li B., Dewey C. N. (2011). RSEM: accurate transcript quantification from RNA-Seq data with or without a reference genome. *BMC Bioinformatics*.

[52] Zhao Y. D., Li M. C., Konate M. M. (2021). TPM, FPKM, or normalized counts? A comparative study of quantification measures for the analysis of RNA-seq data from the NCI patient-derived models repository. *Journal of Translational Medicine*.

[53] Li D., Zand M. S., Dye T. D., Goniewicz M. L., Rahman I., Xie Z. (2022). An evaluation of RNA-seq differential analysis methods. *PLoS One*.

[54] Livak K. J., Schmittgen T. D. (2001). Analysis of relative gene expression data using real-time quantitative PCR and the 2^-*ΔΔ*CT^ method. *Methods*.

[55] Fan C. M., Wang X., Wang Y. W. (2013). Genome-wide expression analysis of soybean MADS genes showing potential function in the seed development. *PLoS One*.

[56] Bao M. R. (2010). *Isolation, cloning, and functional study of the ripening-regulated protein gene from Camellia oleifera seeds, [Ph.D. thesis]*.

[57] Wu B., Ruan C., Han P. (2019). Comparative transcriptomic analysis of high- and low-oil *Camellia oleifera* reveals a coordinated mechanism for the regulation of upstream and downstream multigenes for high oleic acid accumulation. *3 Biotech*.

[58] Wu Y., Ke Y., Wen J. (2018). Evolution and expression analyses of the MADS-box gene family in *Brassica napus*. *PLoS One*.

[59] Zhong S., Yang H., Guan J. (2022). Characterization of the MADS-box gene family in *Akebia trifoliata* and their evolutionary events in angiosperms. *Genes*.

