# Supplementary material for: Genome-Wide Analyses of MADS-Box Genes Reveal Their Involvement in Seed Development and Oil Accumulation of Tea-Oil Tree (Camellia oleifera)
Source: Int J Genomics. 2024 Jul 29;2024:3375173. doi: 10.1155/2024/3375173 (PMC11300058; doi:10.1155/2024/3375173)
Supplement: Supporting Information 1 — Figure S1. A schematic of seed development in Camellia oleifera. [file 3375173.f1.pdf]

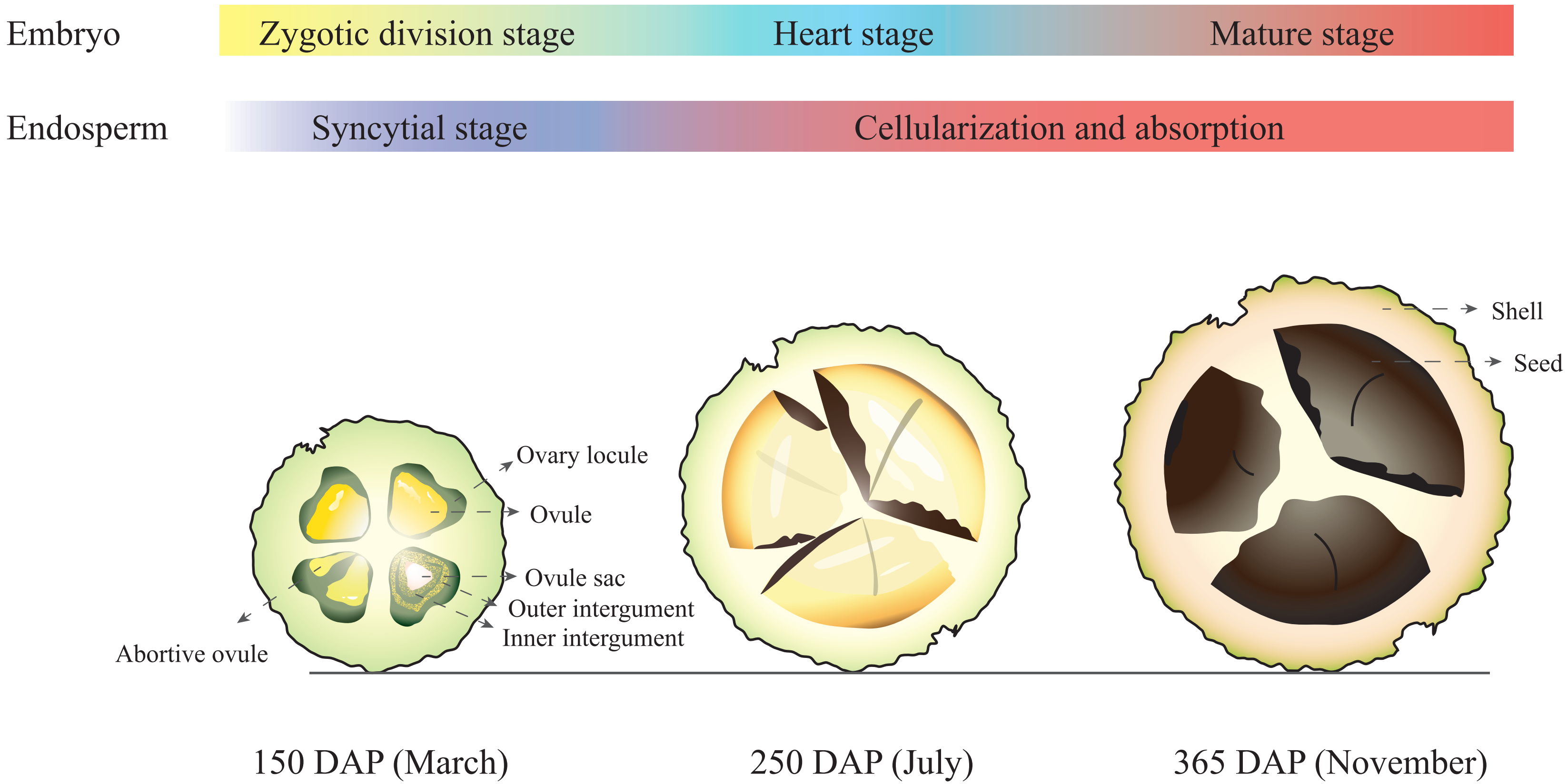
Embryo
Zygotic division stage
Heart stage
Mature stage
Endosperm
Syncytial stage
Cellularization and absorption
Ovary locule
Ovule
Ovule sac
Outer intergument
Inner intergument
Abortive ovule
Shell
Seed
150 DAP (March)
250 DAP (July)
365 DAP (November)
