# Supplementary material for: Genome-Wide Analyses of MADS-Box Genes Reveal Their Involvement in Seed Development and Oil Accumulation of Tea-Oil Tree (Camellia oleifera)
Source: Int J Genomics. 2024 Jul 29;2024:3375173. doi: 10.1155/2024/3375173 (PMC11300058; doi:10.1155/2024/3375173)
Supplement: Supporting Information 2 — Table S1. Detailed information of the MADS-box genes in Camellia oleifera. [file 3375173.f2.docx]

| **Table S1. Detailed information of the MADS-box genes in *Camellia oleifera*.** | | | | | | | | | | |
| --- | --- | --- | --- | --- | --- | --- | --- | --- | --- | --- |
| **Gene name** | **Gene ID** | **Gene length** | **Number of Amino Acid** | **Molecular Weight** | **Theoretical pI** | **Instability Index** | **Aliphatic Index** | **Grand Average of Hydropathicity** | **Subcellular location** | **Group** |
| *ColMADS01* | augustus_masked-HiC_scaffold_1-processed-gene-6.31 | 260 | 86 | 9227.52 | 8.57 | 26.07 | 76.05 | 0.213 | Chloroplasts | α |
| *ColMADS02* | maker-HiC_scaffold_1-snap-gene-286.25 | 11939 | 270 | 31097.79 | 9.8 | 53.72 | 78.33 | -0.629 | Cytoplasm | α |
| *ColMADS03* | augustus_masked-HiC_scaffold_1-processed-gene-312.25 | 266 | 88 | 9558.04 | 8.59 | 12.29 | 83.18 | 0.439 | Chloroplasts | α |
| *ColMADS04* | maker-HiC_scaffold_1-snap-gene-403.29 | 10523 | 191 | 21760.87 | 9.24 | 51.98 | 88.8 | -0.626 | Nucleus | SVP |
| *ColMADS05* | snap_masked-HiC_scaffold_1-processed-gene-1244.25 | 716 | 238 | 27562.23 | 9.7 | 44.58 | 67.61 | -0.716 | Nucleus | γ |
| *ColMADS06* | augustus_masked-HiC_scaffold_1-processed-gene-1933.87 | 215 | 71 | 8365.79 | 9.8 | 48.76 | 87.89 | -0.199 | Nucleus | SOC1 |
| *ColMADS07* | maker-HiC_scaffold_3-snap-gene-405.23 | 5157 | 290 | 32887.83 | 5.12 | 58.47 | 71.28 | -0.61 | Nucleus | MIKC* |
| *ColMADS08* | snap_masked-HiC_scaffold_3-processed-gene-409.30 | 1070 | 356 | 41182.88 | 5.91 | 55.79 | 75.62 | -0.615 | Nucleus | β |
| *ColMADS09* | maker-HiC_scaffold_3-snap-gene-670.36 | 6069 | 216 | 25395.08 | 6.52 | 62.2 | 77.64 | -0.792 | Nucleus | PI |
| *ColMADS10* | augustus_masked-HiC_scaffold_4-processed-gene-267.23 | 740 | 246 | 26747.17 | 6.1 | 32.85 | 74.51 | -0.407 | Cytoplasm | α |
| *ColMADS11* | augustus_masked-HiC_scaffold_4-processed-gene-942.10 | 617 | 205 | 23503.54 | 8.8 | 43.75 | 73.22 | -0.82 | Nucleus | α |
| *ColMADS12* | augustus_masked-HiC_scaffold_4-processed-gene-1043.69 | 242 | 80 | 9434.91 | 10.01 | 51.29 | 76.75 | -0.684 | Nucleus | AG/SHP |
| *ColMADS13* | augustus_masked-HiC_scaffold_4-processed-gene-1626.4 | 254 | 84 | 9057.31 | 6.51 | 19.45 | 77.86 | 0.287 | Chloroplasts | α |
| *ColMADS14* | snap_masked-HiC_scaffold_5-processed-gene-488.9 | 224 | 74 | 7920.93 | 4.86 | 22.29 | 75.14 | 0.335 | Chloroplasts | α |
| *ColMADS15* | snap_masked-HiC_scaffold_5-processed-gene-753.24 | 521 | 173 | 19888.91 | 9.08 | 43.92 | 80.06 | -0.638 | Cytoplasm | γ |
| *ColMADS16* | snap_masked-HiC_scaffold_5-processed-gene-754.9 | 533 | 177 | 20330.31 | 8.91 | 44.14 | 76.61 | -0.724 | Nucleus | γ |
| *ColMADS17* | maker-HiC_scaffold_5-snap-gene-908.18 | 2728 | 169 | 19738.45 | 8.53 | 46.58 | 85.98 | -0.766 | Cytoplasm | TT16 |
| *ColMADS18* | maker-HiC_scaffold_5-snap-gene-917.1 | 2968 | 201 | 23500.08 | 9.56 | 73.52 | 77.56 | -0.805 | Nucleus | PI |
| *ColMADS19* | augustus_masked-HiC_scaffold_5-processed-gene-1272.5 | 239 | 79 | 9001.5 | 9.93 | 68.38 | 85.06 | 0.067 | Mitochondria | ANR1 |
| *ColMADS20* | augustus_masked-HiC_scaffold_5-processed-gene-1549.24 | 1046 | 348 | 37535.3 | 5.6 | 42.46 | 57.82 | -0.302 | Nucleus | α |
| *ColMADS21* | augustus_masked-HiC_scaffold_5-processed-gene-1551.13 | 1055 | 351 | 37758.61 | 5.49 | 43.18 | 57.04 | -0.27 | Nucleus | α |
| *ColMADS22* | augustus_masked-HiC_scaffold_5-processed-gene-1552.5 | 710 | 236 | 25704.25 | 6.91 | 46.52 | 67.84 | -0.348 | Nucleus | α |
| *ColMADS23* | augustus_masked-HiC_scaffold_5-processed-gene-1767.57 | 548 | 182 | 21178.14 | 9.57 | 56.15 | 79.84 | -0.841 | Cytoplasm | γ |
| *ColMADS24* | augustus_masked-HiC_scaffold_6-processed-gene-132.3 | 425 | 141 | 15699.92 | 7.87 | 57.25 | 88.65 | -0.381 | Nucleus | α |
| *ColMADS25* | augustus_masked-HiC_scaffold_6-processed-gene-137.7 | 368 | 122 | 13522.18 | 4.86 | 47.36 | 87.3 | -0.244 | Mitochondria | α |
| *ColMADS26* | augustus_masked-HiC_scaffold_6-processed-gene-354.22 | 731 | 243 | 27900 | 5.29 | 57.35 | 83.05 | -0.426 | Cytoplasm | β |
| *ColMADS27* | augustus_masked-HiC_scaffold_6-processed-gene-354.24 | 749 | 249 | 28642.99 | 5.62 | 59.12 | 85.78 | -0.321 | Nucleus | β |
| *ColMADS28* | augustus_masked-HiC_scaffold_6-processed-gene-357.5 | 740 | 246 | 28050.24 | 5.57 | 51.7 | 83.25 | -0.384 | Cytoplasm | β |
| *ColMADS29* | augustus_masked-HiC_scaffold_6-processed-gene-409.21 | 734 | 244 | 27891.11 | 5.28 | 61.14 | 83.52 | -0.352 | Cytoplasm | β |
| *ColMADS30* | augustus_masked-HiC_scaffold_6-processed-gene-409.22 | 731 | 243 | 27733.79 | 5.05 | 58.11 | 84.65 | -0.387 | Nucleus | β |
| *ColMADS31* | augustus_masked-HiC_scaffold_6-processed-gene-411.77 | 773 | 257 | 29093.42 | 5.44 | 58.21 | 83.11 | -0.352 | Nucleus | β |
| *ColMADS32* | augustus_masked-HiC_scaffold_6-processed-gene-709.20 | 773 | 257 | 29311.84 | 6.15 | 42.31 | 81.56 | -0.458 | Chloroplasts | γ |
| *ColMADS33* | maker-HiC_scaffold_6-snap-gene-1253.21 | 8848 | 229 | 26314.61 | 7.11 | 43.69 | 85.11 | -0.801 | Nucleus | ANR1 |
| *ColMADS34* | maker-HiC_scaffold_6-snap-gene-1375.22 | 7347 | 221 | 25145.63 | 9.71 | 40.44 | 87.38 | -0.493 | Peroxisome | AGL12 |
| *ColMADS35* | maker-HiC_scaffold_7-snap-gene-5.0 | 14850 | 237 | 27268.79 | 6.26 | 45.62 | 75.27 | -0.788 | Nucleus | FLC |
| *ColMADS36* | maker-HiC_scaffold_7-snap-gene-222.11 | 14504 | 462 | 53056.03 | 6.02 | 47.07 | 69.2 | -0.687 | Nucleus | β |
| *ColMADS37* | augustus_masked-HiC_scaffold_7-processed-gene-233.11 | 1070 | 356 | 41277.84 | 5.14 | 46.1 | 70.37 | -0.626 | Nucleus | β |
| *ColMADS38* | snap_masked-HiC_scaffold_7-processed-gene-234.4 | 2616 | 458 | 52532.88 | 8.65 | 49.4 | 70.07 | -0.714 | Chloroplasts | β |
| *ColMADS39* | maker-HiC_scaffold_7-snap-gene-1063.2 | 9064 | 206 | 23707.06 | 8.77 | 54.35 | 90.92 | -0.729 | Nucleus | AP1 |
| *ColMADS40* | maker-HiC_scaffold_7-snap-gene-1146.26 | 5932 | 304 | 33921.74 | 4.93 | 58.43 | 69.64 | -0.562 | Nucleus | MIKC* |
| *ColMADS41* | augustus_masked-HiC_scaffold_7-processed-gene-1568.6 | 920 | 306 | 35467.29 | 6.96 | 34.4 | 67.84 | -0.762 | Nucleus | β |
| *ColMADS42* | augustus_masked-HiC_scaffold_7-processed-gene-1570.38 | 872 | 290 | 33466.86 | 8.15 | 35.54 | 64.21 | -0.776 | Nucleus | β |
| *ColMADS43* | augustus_masked-HiC_scaffold_8-processed-gene-781.14 | 425 | 141 | 15600.78 | 6.32 | 54.75 | 90.07 | -0.344 | Nucleus | α |
| *ColMADS44* | augustus_masked-HiC_scaffold_8-processed-gene-782.13 | 425 | 141 | 15600.78 | 6.32 | 54.75 | 90.07 | -0.344 | Nucleus | α |
| *ColMADS45* | snap_masked-HiC_scaffold_8-processed-gene-782.3 | 425 | 141 | 15567.76 | 5.93 | 47.43 | 91.42 | -0.237 | Mitochondria | α |
| *ColMADS46* | snap_masked-HiC_scaffold_8-processed-gene-888.0 | 3512 | 264 | 29762.2 | 9.11 | 42.38 | 84.89 | -0.346 | Cytoplasm | α |
| *ColMADS47* | maker-HiC_scaffold_9-snap-gene-256.27 | 651 | 199 | 22583.06 | 5.21 | 32.2 | 77.84 | -0.519 | Cytoplasm | β |
| *ColMADS48* | maker-HiC_scaffold_9-snap-gene-474.1 | 798 | 77 | 8992.75 | 11.03 | 61.3 | 101.3 | -0.201 | Chloroplasts | MIKC* |
| *ColMADS49* | augustus_masked-HiC_scaffold_9-processed-gene-482.0 | 773 | 257 | 29885.39 | 4.54 | 59.02 | 82.65 | -0.543 | Cytoplasm | γ |
| *ColMADS50* | maker-HiC_scaffold_9-snap-gene-586.18 | 1615 | 150 | 17686.38 | 10.05 | 59.9 | 72.13 | -0.884 | Nucleus | TM8 |
| *ColMADS51* | maker-HiC_scaffold_9-snap-gene-588.25 | 1575 | 141 | 16508.06 | 9.94 | 60.88 | 76.74 | -0.714 | Nucleus | TM8 |
| *ColMADS52* | augustus_masked-HiC_scaffold_9-processed-gene-693.0 | 683 | 227 | 25839.82 | 5.73 | 35.99 | 73.83 | -0.5 | Nucleus | β |
| *ColMADS53* | augustus_masked-HiC_scaffold_9-processed-gene-693.10 | 779 | 259 | 29533.98 | 6.05 | 45.14 | 71.08 | -0.588 | Nucleus | β |
| *ColMADS54* | maker-HiC_scaffold_9-snap-gene-723.52 | 8696 | 374 | 42621.7 | 8.05 | 35.06 | 81.28 | -0.374 | Cytoplasm | β |
| *ColMADS55* | snap_masked-HiC_scaffold_9-processed-gene-724.7 | 683 | 227 | 25722.71 | 6.64 | 33.18 | 73.83 | -0.492 | Nucleus | β |
| *ColMADS56* | augustus_masked-HiC_scaffold_9-processed-gene-725.0 | 683 | 227 | 25725.73 | 6.06 | 35.11 | 73.83 | -0.466 | Nucleus | β |
| *ColMADS57* | maker-HiC_scaffold_10-snap-gene-1650.15 | 2045 | 208 | 24272.73 | 9.42 | 42.33 | 73.61 | -0.835 | Nucleus | AP3 |
| *ColMADS58* | maker-HiC_scaffold_10-snap-gene-1651.6 | 2218 | 208 | 24272.73 | 9.42 | 42.33 | 73.61 | -0.835 | Nucleus | AP3 |
| *ColMADS59* | maker-HiC_scaffold_10-snap-gene-1726.0 | 7062 | 434 | 48061.98 | 9.31 | 52.65 | 88.34 | -0.292 | Chloroplasts | α |
| *ColMADS60* | maker-HiC_scaffold_10-snap-gene-1890.34 | 5264 | 251 | 28405.42 | 8.23 | 52.82 | 83.86 | -0.701 | Nucleus | AGL15 |
| *ColMADS61* | augustus_masked-HiC_scaffold_11-processed-gene-655.3 | 212 | 70 | 7349.3 | 5.97 | 26.28 | 72.57 | 0.213 | Chloroplasts | α |
| *ColMADS62* | maker-HiC_scaffold_11-snap-gene-720.33 | 7520 | 492 | 56508.88 | 5.8 | 52.03 | 65.59 | -0.67 | Nucleus | β |
| *ColMADS63* | augustus_masked-HiC_scaffold_12-processed-gene-90.39 | 248 | 82 | 9449.1 | 9.98 | 53.16 | 77.2 | -0.1 | Nucleus | FLC |
| *ColMADS64* | snap_masked-HiC_scaffold_12-processed-gene-1039.17 | 361 | 66 | 7605.01 | 10.97 | 43.9 | 90.15 | -0.205 | Nucleus | SOC1 |
| *ColMADS65* | snap_masked-HiC_scaffold_12-processed-gene-1405.7 | 778 | 219 | 24235.77 | 9.33 | 41.41 | 72.6 | -0.448 | Chloroplasts | α |
| *ColMADS66* | augustus_masked-HiC_scaffold_13-processed-gene-9.35 | 911 | 303 | 35035.73 | 6.45 | 36.2 | 61.75 | -0.764 | Nucleus | β |
| *ColMADS67* | augustus_masked-HiC_scaffold_13-processed-gene-635.12 | 263 | 87 | 9383.75 | 7.73 | 18.52 | 79.66 | 0.299 | Chloroplasts | α |
| *ColMADS68* | augustus_masked-HiC_scaffold_13-processed-gene-679.5 | 425 | 141 | 15683.88 | 7.79 | 54.98 | 92.84 | -0.333 | Nucleus | α |
| *ColMADS69* | snap_masked-HiC_scaffold_13-processed-gene-683.22 | 425 | 141 | 15668.81 | 6.32 | 51.19 | 86.6 | -0.414 | Nucleus | α |
| *ColMADS70* | augustus_masked-HiC_scaffold_13-processed-gene-684.26 | 425 | 141 | 15637.78 | 6.16 | 55.67 | 88.01 | -0.379 | Nucleus | α |
| *ColMADS71* | maker-HiC_scaffold_13-snap-gene-684.19 | 484 | 101 | 11005.29 | 4.92 | 39.84 | 88.22 | -0.23 | Chloroplasts | α |
| *ColMADS72* | augustus_masked-HiC_scaffold_13-processed-gene-700.0 | 932 | 310 | 32024.78 | 8.51 | 39.6 | 59.52 | -0.304 | Nucleus | α |
| *ColMADS73* | augustus_masked-HiC_scaffold_13-processed-gene-715.1 | 722 | 240 | 26113.5 | 6.25 | 39.36 | 76.79 | -0.365 | Nucleus | α |
| *ColMADS74* | snap_masked-HiC_scaffold_13-processed-gene-715.33 | 755 | 251 | 27408 | 7.11 | 39.66 | 73.03 | -0.447 | Nucleus | α |
| *ColMADS75* | augustus_masked-HiC_scaffold_13-processed-gene-715.6 | 728 | 242 | 26335.73 | 6.71 | 38.19 | 75.74 | -0.389 | Nucleus | α |
| *ColMADS76* | augustus_masked-HiC_scaffold_13-processed-gene-773.4 | 257 | 85 | 9788.45 | 10.93 | 64.89 | 91.76 | -0.309 | Nucleus | FLC |
| *ColMADS77* | augustus_masked-HiC_scaffold_13-processed-gene-1139.36 | 266 | 88 | 9523.84 | 6.51 | 28.94 | 80.91 | 0.256 | Chloroplasts | α |
| *ColMADS78* | maker-HiC_scaffold_13-snap-gene-1736.47 | 4271 | 251 | 28424.42 | 8.75 | 51.29 | 82.31 | -0.722 | Nucleus | AGL15 |
| *ColMADS79* | augustus_masked-HiC_scaffold_14-processed-gene-1172.38 | 224 | 74 | 7913.04 | 8.77 | 21.54 | 77.84 | 0.27 | Chloroplasts | α |
| *ColMADS80* | augustus_masked-HiC_scaffold_14-processed-gene-1408.16 | 416 | 138 | 15167.57 | 9.52 | 46.05 | 78.55 | -0.362 | Nucleus | AGL12 |
| *ColMADS81* | snap_masked-HiC_scaffold_15-processed-gene-54.28 | 626 | 208 | 23010.81 | 5.05 | 41.16 | 76.92 | -0.642 | Chloroplasts | α |
| *ColMADS82* | maker-HiC_scaffold_15-snap-gene-95.17 | 7266 | 261 | 30492.21 | 9.66 | 54.26 | 77.28 | -0.572 | Chloroplasts | α |
| *ColMADS83* | maker-HiC_scaffold_15-snap-gene-134.31 | 11520 | 216 | 25085.87 | 8.21 | 70.36 | 89.4 | -0.703 | Nucleus | SOC1 |
| *ColMADS84* | maker-HiC_scaffold_15-snap-gene-607.39 | 462 | 133 | 14432.35 | 10.41 | 78.75 | 71.2 | -0.742 | Nucleus | α |
| *ColMADS85* | augustus_masked-HiC_scaffold_15-processed-gene-1351.0 | 275 | 91 | 10309.29 | 9.97 | 56.49 | 101.87 | 0.165 | Chloroplasts | SOC1 |
| *ColMADS86* | genemark-scaffold_2313_fragment_2-processed-gene-0.3 | 425 | 141 | 15567.76 | 5.93 | 47.43 | 91.42 | -0.237 | Mitochondria | α |
