# Supplementary material for: Genome-Wide Analyses of MADS-Box Genes Reveal Their Involvement in Seed Development and Oil Accumulation of Tea-Oil Tree (Camellia oleifera)
Source: Int J Genomics. 2024 Jul 29;2024:3375173. doi: 10.1155/2024/3375173 (PMC11300058; doi:10.1155/2024/3375173)
Supplement: Supporting Information 3 — Table S2. Gene duplication events in the MADS-box gene family of Camellia oleifera. [file 3375173.f3.docx]

| **Table S2. Gene duplication events in the MADS-box gene family of *Camellia oleifera*.** | | | | | | | |
| --- | --- | --- | --- | --- | --- | --- | --- |
| **Gene1** | **Gene2** |  | **Duplication type** | **Ka** | **Ks** | **Ka_Ks** | **Dulication date(MY)** |
| augustus_masked-HiC_scaffold_5-processed-gene-1551.13 | augustus_masked-HiC_scaffold_5-processed-gene-1552.5 | ColMADS21/22 | Tandem | 0.02353 | 0.041152 | 0.571787 | 1.37 |
| snap_masked-HiC_scaffold_5-processed-gene-753.24 | snap_masked-HiC_scaffold_5-processed-gene-754.9 | ColMADS15/16 | Tandem | 0.04368 | 0.073891 | 0.59115 | 2.46 |
| augustus_masked-HiC_scaffold_8-processed-gene-781.14 | augustus_masked-HiC_scaffold_8-processed-gene-782.13 | ColMADS43/44 | Tandem | 0 | 0.010292 | 0 | 3.43 |
| snap_masked-HiC_scaffold_9-processed-gene-724.7 | augustus_masked-HiC_scaffold_9-processed-gene-725.0 | ColMADS55/56 | Tandem | 0.001845 | 0 | - | 0 |
| augustus_masked-HiC_scaffold_13-processed-gene-715.1 | snap_masked-HiC_scaffold_13-processed-gene-715.33 | ColMADS73/74 | Tandem | 0.00534 | 0.006431 | 0.830316 | 0.21 |
| maker-HiC_scaffold_3-snap-gene-670.36 | maker-HiC_scaffold_5-snap-gene-908.18 | ColMADS09/17 | Segmental | 0.5333 | NaN | NaN | - |
| augustus_masked-HiC_scaffold_6-processed-gene-354.22 | augustus_masked-HiC_scaffold_6-processed-gene-409.21 | ColMADS26/29 | Segmental | 0.012144 | 0.034588 | 0.351109 | 1.15 |
| augustus_masked-HiC_scaffold_1-processed-gene-1933.87 | snap_masked-HiC_scaffold_12-processed-gene-1039.17 | ColMADS06/64 | Segmental | 0.061433 | 0.435356 | 0.14111 | 1.45 |
