# Supplementary material for: Genome-Wide Analyses of MADS-Box Genes Reveal Their Involvement in Seed Development and Oil Accumulation of Tea-Oil Tree (Camellia oleifera)
Source: Int J Genomics. 2024 Jul 29;2024:3375173. doi: 10.1155/2024/3375173 (PMC11300058; doi:10.1155/2024/3375173)
Supplement: Supporting Information 4 — Table S3. Conserved motifs in ColMADS proteins. [file 3375173.f4.docx]

| **Table S3. Conserved motifs in ColMADS proteins.** | |  |  |  |  |
| --- | --- | --- | --- | --- | --- |
| **Motif** | **Sequences** | **Go terms** | **Domain (From InterPro)** | **Tomtom (List the first 3)** | **Predict tomtom** |
| 1 | MGRGKIEIKRIENKSNRQVTFSKRRNGLFKKASELSVLCDAEVAJJVFSP | Protein dimerization activity; DNA binding | IPR002100 | PS00350, PS00256, PS00413 | MADS box 1 |
| 2 | NPSVDSVVDRYLSTTSASEKAS | — | — | PS01280, PS00417, PS00974 | Glucose inhibited division protein |
| 3 | DEWLDGLSJEELEQLEKLLEEKLKKVKAR | — | — | PS00029, PS00400, PS00223 | Leucine zipper pattern |
| 4 | NNEVETWPZNPNEVKTLIBTYKNQSKEDRLRRTLE | — | — | PS00688, PS00664, PS01220 | Vinculin repeated domain |
| 5 | TYFKRKAGLFKKTMELCILCGTEAAVITF | Protein dimerization activity; DNA binding | IPR002100 | PS00350, PS00568, PS00159 | MADS box 1 |
| 6 | TLNAMNNCDINQMCLQFASPLNELLFASMYTMNPLQITEPQLGEETISSS | — | — | PS00979, PS01084, PS00628 | G-protein coupled receptors family |
| 7 | IELLMFSKMHYQYQPLIEPPMFVPLWPLNCYGVPGSSGGGG | — | — | PS00721, PS01288, PS00429 | Formate--tetrahydrofolate ligase |
| 8 | HHPSPSMGYYGGSSMQTMSPYMQYLKMASGPSQVHAWQMEEYYKANEFQM | — | — | PS00324, PS00810, PS00876 | Aspartokinase or ADP-glucose pyrophosphorylase |
| 9 | NFFEBRNQKIEDALVKLRKKNDKALYSTW | — | — | PS00967, PS00007, PS00965 | Tyrosine kinase phosphorylation site |
| 10 | LNDMEQTTQQNHSNYFMQGLFGTTSLQMGIIHEKTPISSLN | — | — | PS00970, PS00405, PS01014 | Nuclear transition protein |
| 11 | AGKLYEFG | — | — | PS00576, PS01104, PS01058 | General diffusion Gram-negative porins signature. |
| 12 | VRSVQELNLKYTKVLGELEAEKRRGEALDE | — | — | PS01088, PS00446, PS01203 | - |
| 13 | SSSVLVMGKPLEEAIYYDPTMLJPNFENV | — | — | PS00485, PS01183, PS00007 | Adenosine and AMP deaminase |
| 14 | YEIDVIALHYPFBQLDHQGIVELDAKSMA | — | — | PS00763, PS00847, PS00387 | Glutathione peroxidases |
| 15 | GCEAAAANHHRLSSW | — | — | PS00458, PS00291, PS01266 | - |
