# Supplementary material for: Genome-Wide Analyses of MADS-Box Genes Reveal Their Involvement in Seed Development and Oil Accumulation of Tea-Oil Tree (Camellia oleifera)
Source: Int J Genomics. 2024 Jul 29;2024:3375173. doi: 10.1155/2024/3375173 (PMC11300058; doi:10.1155/2024/3375173)
Supplement: Supporting Information 6 — Table S5. Differentially expressed ColMADS genes between S1/S2 (a), S2/S3 (b) and S1/S3 (c). [file 3375173.f6.docx]

| **Table S5. Differentially expressed ColMADS genes between S1/S2 (A), S2/S3 (B) and S1/S3 (C).** | | | | | | | |
| --- | --- | --- | --- | --- | --- | --- | --- |
| **(A) Differential expressed genes between S1/S2** | | | |  |  |  |  |
| **Gene ID** | **Gene name** | **baseMean** | **Log2FoldChange** | **IfcSE** | **Stat** | **Pvalue** | **padj** |
| maker-HiC_scaffold_3-snap-gene-405.23 | *ColMADS07* | 2185.6932 | 4.2882011 | 1.1181624 | 3.8350433 | 0.0001255 | 0.000871 |
| augustus_masked-HiC_scaffold_4-processed-gene-1043.69 | *ColMADS12* | 8.4333008 | 6.5706062 | 2.3879684 | 2.7515466 | 0.0059315 | 0.0129472 |
| maker-HiC_scaffold_6-snap-gene-1375.22 | *ColMADS34* | 26.587919 | 1.0894159 | 0.3299538 | 3.3017223 | 0.0009609 | 0.0051458 |
| maker-HiC_scaffold_7-snap-gene-1063.2 | *ColMADS39* | 77.265886 | -3.54355 | 1.3608294 | -2.603964 | 0.0092153 | 0.0351761 |
| snap_masked-HiC_scaffold_9-processed-gene-724.7 | *ColMADS55* | 177.55287 | 1.1052583 | 0.2290501 | 4.8254003 | 1.40E-06 | 1.54E-05 |
| maker-HiC_scaffold_10-snap-gene-1890.34 | *ColMADS60* | 2.9147776 | 3.0089572 | 1.0956702 | 2.7462252 | 0.0060285 | 0.0247256 |
| snap_masked-HiC_scaffold_12-processed-gene-1405.7 | *ColMADS65* | 11.805192 | -1.983581 | 0.8229505 | -2.410329 | 0.0159381 | 0.0313953 |
| augustus_masked-HiC_scaffold_13-processed-gene-773.4 | *ColMADS76* | 36.973232 | -4.589176 | 0.9830873 | -4.668126 | 3.04E-06 | 3.12E-05 |
|  |  |  |  |  |  |  |  |
| **(B) Differential expressed genes between S2/S3** | | | |  |  |  |  |
| **Gene ID** | **Gene name** | **baseMean** | **Log2FoldChange** | **IfcSE** | **Stat** | **Pvalue** | **padj** |
| maker-HiC_scaffold_3-snap-gene-405.23 | *ColMADS07* | 2185.6932 | 4.759945 | 1.1057136 | 4.3048625 | 1.67E-05 | 5.69E-05 |
| augustus_masked-HiC_scaffold_4-processed-gene-1043.69 | *ColMADS12* | 219.20477 | -5.925291 | 0.6097224 | -9.718013 | 2.53E-22 | 4.38E-21 |
| maker-HiC_scaffold_5-snap-gene-908.18 | *ColMADS17* | 31.487668 | -6.686233 | 1.2559274 | -5.323742 | 1.02E-07 | 4.65E-07 |
| maker-HiC_scaffold_6-snap-gene-1375.22 | *ColMADS34* | 26.587919 | -4.749219 | 0.8906878 | -5.33208 | 9.71E-08 | 4.45E-07 |
| maker-HiC_scaffold_7-snap-gene-1063.2 | *ColMADS39* | 77.265886 | -5.663823 | 1.799408 | -3.147603 | 0.0016461 | 0.0040139 |
| maker-HiC_scaffold_7-snap-gene-1146.26 | *ColMADS40* | 11.805192 | -1.983581 | 0.8229505 | -2.410329 | 0.0159381 | 0.0313953 |
| maker-HiC_scaffold_9-snap-gene-474.1 | *ColMADS48* | 129.1496 | 1.451207 | 0.305539 | 4.7496623 | 2.04E-06 | 7.87E-06 |
| maker-HiC_scaffold_10-snap-gene-1650.15 | *ColMADS57* | 1049.7522 | 1.8249446 | 0.1328913 | 13.732614 | 6.48E-43 | 3.98E-41 |
| augustus_masked-HiC_scaffold_11-processed-gene-655.3 | *ColMADS61* | 138.8457 | 1.0036426 | 0.2720237 | 3.6895415 | 0.0002247 | 0.0006402 |
| maker-HiC_scaffold_11-snap-gene-720.33 | *ColMADS62* | 90.326353 | -2.60739 | 0.3313732 | -7.868439 | 3.59E-15 | 3.54E-14 |
| snap_masked-HiC_scaffold_12-processed-gene-1039.17 | *ColMADS64* | 12.873627 | -5.692135 | 1.2814241 | -4.442038 | 8.91E-06 | 3.14E-05 |
| snap_masked-HiC_scaffold_12-processed-gene-1405.7 | *ColMADS65* | 1049.7522 | 1.2325859 | 0.1290325 | 9.5525239 | 1.27E-21 | 1.95E-20 |
| augustus_masked-HiC_scaffold_13-processed-gene-684.26 | *ColMADS70* | 33.561636 | -1.10214 | 0.3995884 | -2.758188 | 0.0058123 | 0.0127097 |
| augustus_masked-HiC_scaffold_13-processed-gene-773.4 | *ColMADS76* | 35.780422 | 6.0768122 | 0.9897821 | 6.1395458 | 8.28E-10 | 4.84E-09 |
| snap_masked-HiC_scaffold_15-processed-gene-54.28 | *ColMADS81* | 7.6597693 | 2.5095588 | 1.0236572 | 2.4515616 | 0.0142238 | 0.0283902 |
|  |  |  |  |  |  |  |  |
| **(C) Differential expressed genes between S1/S3** | | | |  |  |  |  |
| **Gene ID** | **Gene name** | **baseMean** | **Log2FoldChange** | **IfcSE** | **Stat** | **Pvalue** | **padj** |
| maker-HiC_scaffold_3-snap-gene-405.23 | *ColMADS07* | 2185.6932 | 9.0481461 | 1.1168184 | 8.1017167 | 5.42E-16 | 5.34E-15 |
| augustus_masked-HiC_scaffold_4-processed-gene-1043.69 | *ColMADS12* | 219.20477 | -6.19795 | 0.6084478 | -10.18649 | 2.28E-24 | 4.27E-23 |
| maker-HiC_scaffold_5-snap-gene-908.18 | *ColMADS17* | 31.487668 | -7.308312 | 1.2499837 | -5.846726 | 5.01E-09 | 2.50E-08 |
| augustus_masked-HiC_scaffold_6-processed-gene-411.77 | *ColMADS31* | 3.6418184 | -4.782295 | 1.6913481 | -2.827505 | 0.0046912 | 0.0104629 |
| maker-HiC_scaffold_6-snap-gene-1375.22 | *ColMADS34* | 26.587919 | -3.659803 | 0.8903079 | -4.110716 | 3.94E-05 | 0.0001201 |
| maker-HiC_scaffold_7-snap-gene-1063.2 | *ColMADS39* | 77.265886 | -9.207373 | 1.7883365 | -5.148568 | 2.62E-07 | 1.06E-06 |
| maker-HiC_scaffold_7-snap-gene-1146.26 | *ColMADS40* | 11.805192 | -2.0466 | 0.8061751 | -2.538654 | 0.011128 | 0.0218833 |
| augustus_masked-HiC_scaffold_8-processed-gene-782.13 | *ColMADS44* | 4.8688819 | -4.051896 | 1.4233948 | -2.846642 | 0.0044183 | 0.0095039 |
| maker-HiC_scaffold_9-snap-gene-474.1 | *ColMADS48* | 138.8457 | 1.0430528 | 0.2644257 | 3.9445964 | 7.99E-05 | 0.000232 |
| maker-HiC_scaffold_10-snap-gene-1650.15 | *ColMADS57* | 245.15539 | 1.4059617 | 0.3917082 | 3.5893089 | 0.0003316 | 0.0008727 |
| maker-HiC_scaffold_11-snap-gene-720.33 | *ColMADS62* | 90.326353 | -2.76133 | 0.3258641 | -8.47387 | 2.37E-17 | 2.62E-16 |
| snap_masked-HiC_scaffold_12-processed-gene-1039.17 | *ColMADS64* | 12.873627 | -5.795174 | 1.2722765 | -4.554964 | 5.24E-06 | 1.80E-05 |
| snap_masked-HiC_scaffold_12-processed-gene-1405.7 | *ColMADS65* | 1.6997726 | -4.531337 | 1.8369574 | -2.466762 | 0.0136341 | 0.048455 |
| snap_masked-HiC_scaffold_13-processed-gene-683.22 | *ColMADS69* | 7.7968959 | -4.045029 | 1.2805224 | -3.158889 | 0.0015837 | 0.0037044 |
| augustus_masked-HiC_scaffold_13-processed-gene-684.26 | *ColMADS70* | 33.561636 | -1.268046 | 0.3844413 | -3.298412 | 0.0009723 | 0.0023626 |
| augustus_masked-HiC_scaffold_13-processed-gene-773.4 | *ColMADS76* | 36.081125 | 1.4876363 | 0.6575462 | 2.2624057 | 0.0236723 | 0.0430559 |
| snap_masked-HiC_scaffold_15-processed-gene-54.28 | *ColMADS81* | 79.630578 | 1.0548763 | 0.3673758 | 2.8713817 | 0.0040868 | 0.0088434 |
