# Supplementary material for: Genome-Wide Analyses of MADS-Box Genes Reveal Their Involvement in Seed Development and Oil Accumulation of Tea-Oil Tree (Camellia oleifera)
Source: Int J Genomics. 2024 Jul 29;2024:3375173. doi: 10.1155/2024/3375173 (PMC11300058; doi:10.1155/2024/3375173)
Supplement: Supporting Information 7 — Table S6. Name and ID of MADS-box genes in Arabidopsis thaliana (a), Oryza sativa (b), and Solanum lycopersicum (c) used in this study. [file 3375173.f7.docx]

| **Table S6. Name and ID of MADS-box genes in *Arabidopsis thaliana* (A), *Oryza sativa* (B), and *Solanum lycopersicum* (C) used in this study** | | | | | | | |
| --- | --- | --- | --- | --- | --- | --- | --- |
| **(A) *Arabidopsis thaliana*** | |  | **(B) *Oryza sativa*** |  |  | **(C) *Solanum lycopersicum*** | |
| **Gene Name** | **Gene ID** |  | **Gene Name** | **Gene ID** |  | **Gene Name** | **Gene ID** |
| *AG* | AT4G18960.1 |  | *OsMADS1* | LOC_Os03g11614.1 |  | *Solyc01g087990.2.1* | Solyc01g087990.2.1 |
| *AGL100* | AT1G17310.1 |  | *OsMADS13* | LOC_Os12g10540.1 |  | *Solyc01g093960.2.1* | Solyc01g093960.2.1 |
| *AGL101* | AT5G27050.1 |  | *OsMADS14* | LOC_Os03g54160.1 |  | *Solyc01g105800.2.1* | Solyc01g105800.2.1 |
| *AGL102* | AT1G47760.1 |  | *OsMADS15* | LOC_Os07g01820.1 |  | *Solyc02g065730.1.1* | Solyc02g065730.1.1 |
| *AGL103* | AT3G18650.1 |  | *OsMADS16* | LOC_Os06g49840.1 |  | *Solyc02g071730.2.1* | Solyc02g071730.2.1 |
| *AGL104* | AT1G22130.1 |  | *OsMADS17* | LOC_Os04g49150.1 |  | *Solyc02g084630.2.1* | Solyc02g084630.2.1 |
| *AGL12* | AT1G71692.1 |  | *OsMADS18* | LOC_Os07g41370.1 |  | *Solyc02g089200.2.1* | Solyc02g089200.2.1 |
| *AGL13* | AT3G61120.1 |  | *OsMADS2* | LOC_Os01g66030.1 |  | *Solyc02g089210.2.1* | Solyc02g089210.2.1 |
| *AGL14* | AT4G11880.1 |  | *OsMADS20* | LOC_Os12g31748.1 |  | *Solyc02g091550.1.1* | Solyc02g091550.1.1 |
| *AGL15* | AT5G13790.2 |  | *OsMADS21* | LOC_Os01g66290.1 |  | *Solyc03g006830.2.1* | Solyc03g006830.2.1 |
| *AGL16* | AT3G57230.1 |  | *OsMADS22* | LOC_Os02g52340.1 |  | *Solyc03g019710.2.1* | Solyc03g019710.2.1 |
| *AGL17* | AT2G22630.2 |  | *OsMADS23* | LOC_Os08g33488.1 |  | *Solyc03g114830.2.1* | Solyc03g114830.2.1 |
| *AGL18* | AT3G57390.1 |  | *OsMADS25* | LOC_Os04g23910.1 |  | *Solyc03g114840.2.1* | Solyc03g114840.2.1 |
| *AGL19* | AT4G22950.1 |  | *OsMADS26* | LOC_Os08g02070.1 |  | *Solyc04g005320.2.1* | Solyc04g005320.2.1 |
| *AGL21* | AT4G37940.1 |  | *OsMADS27* | LOC_Os02g36924.1 |  | *Solyc04g078300.2.1* | Solyc04g078300.2.1 |
| *AGL23* | AT1G65360.1 |  | *OsMADS29* | LOC_Os02g07430.1 |  | *Solyc04g081000.2.1* | Solyc04g081000.2.1 |
| *AGL24* | AT4G24540.1 |  | *OsMADS3* | LOC_Os01g10504.1 |  | *Solyc05g012020.2.1* | Solyc05g012020.2.1 |
| *AGL28* | AT1G01530.1 |  | *OsMADS30* | LOC_Os06g45650.1 |  | *Solyc05g015750.2.1* | Solyc05g015750.2.1 |
| *AGL29* | AT2G34440.1 |  | *OsMADS31* | LOC_Os04g52410.1 |  | *Solyc05g056620.1.1* | Solyc05g056620.1.1 |
| *AGL30* | AT2G03060.2 |  | *OsMADS32* | LOC_Os01g52680.1 |  | *Solyc06g059970.2.1* | Solyc06g059970.2.1 |
| *AGL32* | AT5G23260.4 |  | *OsMADS33* | LOC_Os12g10520.1 |  | *Solyc06g069430.2.1* | Solyc06g069430.2.1 |
| *AGL33* | AT2G26320.1 |  | *OsMADS34* | LOC_Os03g54170.1 |  | *Solyc07g055920.2.1* | Solyc07g055920.2.1 |
| *AGL35* | AT5G26630.1 |  | *OsMADS37* | LOC_Os08g41960.1 |  | *Solyc08g067230.2.1* | Solyc08g067230.2.1 |
| *AGL36* | AT5G26650.1 |  | *OsMADS4* | LOC_Os05g34940.1 |  | *Solyc08g080100.2.1* | Solyc08g080100.2.1 |
| *AGL39* | AT5G27130.1 |  | *OsMADS47* | LOC_Os03g08754.1 |  | *Solyc10g080030.1.1* | Solyc10g080030.1.1 |
| *AGL40* | AT4G36590.1 |  | *OsMADS5* | LOC_Os06g06750.1 |  | *Solyc11g010570.1.1* | Solyc11g010570.1.1 |
| *AGL41* | AT2G26880.1 |  | *OsMADS55* | LOC_Os06g11330.1 |  | *Solyc11g028020.1.1* | Solyc11g028020.1.1 |
| *AGL42* | AT5G62165.2 |  | *OsMADS56* | LOC_Os10g39130.1 |  | *Solyc11g032100.1.1* | Solyc11g032100.1.1 |
| *AGL43* | AT5G40220.1 |  | *OsMADS57* | LOC_Os02g49840.1 |  | *Solyc12g005210.1.1* | Solyc12g005210.1.1 |
| *AGL45* | AT3G05860.1 |  | *OsMADS58* | LOC_Os05g11414.1 |  | *Solyc12g038510.1.1* | Solyc12g038510.1.1 |
| *AGL46* | AT2G28700.1 |  | *OsMADS6* | LOC_Os02g45770.1 |  | *Solyc12g056460.1.1* | Solyc12g056460.1.1 |
| *AGL47* | AT5G55690.1 |  | *OsMADS66* | LOC_Os05g11380.1 |  | *Solyc12g087830.1.1* | Solyc12g087830.1.1 |
| *AGL48* | AT2G40210.1 |  | *OsMADS7* | LOC_Os08g41950.1 |  |  |  |
| *AGL49* | AT1G60040.1 |  | *OsMADS8* | LOC_Os09g32948.1 |  |  |  |
| *AGL50* | AT1G59810.1 |  |  |  |  |  |  |
| *AGL52* | AT4G11250.1 |  |  |  |  |  |  |
| *AGL53* | AT5G27070.1 |  |  |  |  |  |  |
| *AGL54* | AT5G27090.1 |  |  |  |  |  |  |
| *AGL55* | AT1G60920.1 |  |  |  |  |  |  |
| *AGL56* | AT1G60880.1 |  |  |  |  |  |  |
| *AGL57* | AT3G04100.1 |  |  |  |  |  |  |
| *AGL58* | AT1G28450.1 |  |  |  |  |  |  |
| *AGL59* | AT1G28460.1 |  |  |  |  |  |  |
| *AGL6* | AT2G45650.1 |  |  |  |  |  |  |
| *AGL60* | AT1G72350.1 |  |  |  |  |  |  |
| *AGL61* | AT2G24840.1 |  |  |  |  |  |  |
| *AGL62* | AT5G60440.1 |  |  |  |  |  |  |
| *AGL65* | AT1G18750.3 |  |  |  |  |  |  |
| *AGL66* | AT1G77980.1 |  |  |  |  |  |  |
| *AGL67* | AT1G77950.4 |  |  |  |  |  |  |
| *AGL71* | AT5G51870.3 |  |  |  |  |  |  |
| *AGL72* | AT5G51860.1 |  |  |  |  |  |  |
| *AGL73* | AT5G38620.1 |  |  |  |  |  |  |
| *AGL74* | AT1G48150.1 |  |  |  |  |  |  |
| *AGL75* | AT5G41200.1 |  |  |  |  |  |  |
| *AGL76* | AT5G40120.1 |  |  |  |  |  |  |
| *AGL77* | AT5G38740.1 |  |  |  |  |  |  |
| *AGL78* | AT5G65330.1 |  |  |  |  |  |  |
| *AGL79* | AT3G30260.1 |  |  |  |  |  |  |
| *AGL80* | AT5G48670.1 |  |  |  |  |  |  |
| *AGL81* | AT5G39750.1 |  |  |  |  |  |  |
| *AGL82* | AT5G58890.1 |  |  |  |  |  |  |
| *AGL83* | AT5G49490.1 |  |  |  |  |  |  |
| *AGL84* | AT5G49420.1 |  |  |  |  |  |  |
| *AGL85* | AT1G54760.1 |  |  |  |  |  |  |
| *AGL86* | AT1G31630.1 |  |  |  |  |  |  |
| *AGL87* | AT1G22590.2 |  |  |  |  |  |  |
| *AGL89* | AT5G27580.1 |  |  |  |  |  |  |
| *AGL90* | AT5G27960.1 |  |  |  |  |  |  |
| *AGL91* | AT3G66656.1 |  |  |  |  |  |  |
| *AGL92* | AT1G31640.1 |  |  |  |  |  |  |
| *AGL93* | AT5G26950.1 |  |  |  |  |  |  |
| *AGL94* | AT1G69540.2 |  |  |  |  |  |  |
| *AGL95* | AT2G15660.1 |  |  |  |  |  |  |
| *AGL96* | AT5G06500.1 |  |  |  |  |  |  |
| *AGL97* | AT1G46408.1 |  |  |  |  |  |  |
| *AGL98* | AT5G39810.1 |  |  |  |  |  |  |
| *AGL99* | AT5G04640.1 |  |  |  |  |  |  |
| *ANR1* | AT2G14210.2 |  |  |  |  |  |  |
| *AP1* | AT1G69120.1 |  |  |  |  |  |  |
| *AP3* | AT3G54340.1 |  |  |  |  |  |  |
| *CAL* | AT1G26310.1 |  |  |  |  |  |  |
| *FLC* | AT5G10140.1 |  |  |  |  |  |  |
| *FUL* | AT5G60910.1 |  |  |  |  |  |  |
| *GOA* | AT1G31140.2 |  |  |  |  |  |  |
| *MADS14* | AT1G33070.1 |  |  |  |  |  |  |
| *MAF1* | AT1G77080.8 |  |  |  |  |  |  |
| *MAF2* | AT5G65050.3 |  |  |  |  |  |  |
| *MAF3* | AT5G65060.1 |  |  |  |  |  |  |
| *MAF4* | AT5G65070.1 |  |  |  |  |  |  |
| *MAF5* | AT5G65080.2 |  |  |  |  |  |  |
| *PHE1* | AT1G65330.1 |  |  |  |  |  |  |
| *PHE2* | AT1G65300.1 |  |  |  |  |  |  |
| *PI* | AT5G20240.1 |  |  |  |  |  |  |
| *SEP1* | AT5G15800.2 |  |  |  |  |  |  |
| *SEP2* | AT3G02310.1 |  |  |  |  |  |  |
| *SEP3* | AT1G24260.2 |  |  |  |  |  |  |
| *SEP4* | AT2G03710.1 |  |  |  |  |  |  |
| *SHP1* | AT3G58780.4 |  |  |  |  |  |  |
| *SHP2* | AT2G42830.2 |  |  |  |  |  |  |
| *SOC1* | AT2G45660.1 |  |  |  |  |  |  |
| *STK* | AT4G09960.4 |  |  |  |  |  |  |
| *SVP* | AT2G22540.1 |  |  |  |  |  |  |
