# Supplementary material for: Genome-Wide Analyses of MADS-Box Genes Reveal Their Involvement in Seed Development and Oil Accumulation of Tea-Oil Tree (Camellia oleifera)
Source: Int J Genomics. 2024 Jul 29;2024:3375173. doi: 10.1155/2024/3375173 (PMC11300058; doi:10.1155/2024/3375173)
Supplement: Supporting Information 8 — Table S7. Primers used in this study for qRT-PCR analysis. [file 3375173.f8.docx]

| **Table S7. Primers used in this study for qRT-PCR analysis.** | |  |
| --- | --- | --- |
| **Gene name** | **Forward primer sequences** | **Reverse primer sequences** |
| *ColMADS07* | CAATCTTGTATGCAGGAGATGCT | GACCCTTGCAACATTGGCTC |
| *ColMADS12* | TGGATGTCTCTCGTCTCTCC | ACAATAAGGGCAACCTCAGCA |
| *ColMADS48* | GCACTAGCAATCAACAAGTGACC | CAATGTTGCTGCGCTCTCTG |
| *ColMADS57* | TAAGGAGAGAGATCGGGCAGA | TCCATGCCTTTCCTCCATGC |
| *ColMADS61* | CTTCCCTTCCACAGATGGCCT | TTGCGGAGGCTTTGGAGGAT |
| *ColMADS76* | CGCCGACAAGGACTGATGAA | TGTCGGAAAATCGAACATTAAGGA |
| *Actin* | CTTGCTGGCCGTGATCTAAC | CACCCTTTGAAATCCACATC |
| *CoEF1α* | CATGATCACTGGTACCTCACAG | CATGATCACTGGTACCTCACAG |
