# Supplementary material for: Genome-Wide Analyses of MADS-Box Genes Reveal Their Involvement in Seed Development and Oil Accumulation of Tea-Oil Tree (Camellia oleifera)
Source: Int J Genomics. 2024 Jul 29;2024:3375173. doi: 10.1155/2024/3375173 (PMC11300058; doi:10.1155/2024/3375173)
Supplement: Supporting Information 9 — Additional Data 1: the nucleotide and amino-acid sequences of MADS-box genes in Camellia oleifera, Arabidopsis thaliana, Oryza sativa, and Solanum lycopersicum. [file 3375173.f9.docx]

**#nucleotide sequences of MADS-box genes in *Camellia oleifera***

>ColMADS01

ATGACTATTATACCCACTCTCTTCCCCACTCTCTTTCTTTCCACATATGGCCTCTTCAAGAAGGCCGGCGAGCTTTGCAACTTGTGCGGCACCGAAGCCGCCGTCATCACCTTCTCCAACGCCGGCAACACCTTCGCCTTCGGCAACCCCTCCGTCGACTCTGTCCTCGACCGTTACCTCTCCATGACTTCATCATCCTCCAGAGCCTCCGCAAGCTTCTTTCAACTCAAATCTGTGCACAATTCCTGCAAATCGCAGTAA

>ColMADS02

ATGTATGTGCTCCCTCTGTGCTTTGACTATGGTGACCAAATATTCCATAATATTCTGTGTGACCCAGTGAAAGAAAGGGAAACCATGCATCGGCGAAGGCGGCATGGACAAAAGAAGATGGTGAAGGAGAAAGAGAATGGCGAAAGAGGACGACGAATATTCAAGAAGAGTTCCTCAATAGTTTTAGCCATGGCAAAGAAACCAAGCATGGGTCGCCAAAAGATCAAAATTGAGAAAATAGAGATCAAAAATCACTTGCAAGTCACCTTCTCAAAACGCCGATCTGGGCTTTTCAAGAAGGCAAGCGAACTTTGCACGCTTTGTGGCATAGAGATTGCTATCATAGTTTTCTCTCCGGCTGGCAAAGTGTTTTCTTTTGGCCACCCTAACGTGGATTCTATCGTTGATCAATTCCTAACTCGAAACCCTCCACCTAACACCACCACACGCCACCTTATTGAGGCTCATCGTAGTGCTAGCGTGCGCGAGCTCAACTTACAACTTACTCAAGTTCTCAATGAATTGGAAGTCGAGAAAAGACATGGTGAAACCTTAGATCACACGAGAAAGGCTAGTCAAAGACAATACTGGTGGGAAGCTCCAATAGACAAGCTTGGCTTGCATGAGCTTGAACAACTTAGGAATTCAATGGTGGAGCTCAAGAAGAACGTAACCAATCAGGCAAACAAAATTCAATTCGAGGGCACCACAAACCCTTCACCATTTTTCTTAGCAAATGGCACCAGAATGGTTGATCACTTTGAGAGTAAAACATCTCATATCAATGCTTCTTCTTCAATCACTCCTAAT

>ColMADS03

ATGACTATTATACCCACTCTCTTCCCCATTTTCTTCCTTTCCACATATGGCCTCTTCAAGAAGACCGGCGAGCTCTGCAACTTGTGCGGCACCGAAGCCGCCGTCATCACCTTCTCCAACGCCGGCAACACCTTGGCCTTCGGCAACCCCTCCGTCGACTTCGTCCTCGACCGTTACCTCTCCATGACTTCATCATCCTTCAAAGCCTCCGCAAGCTTCTCTTCCCTCTTTCGACATAGGGTTTCAACTCAAATCTGTGCACCATGA

>ColMADS04

ATGGTGAGACAGAGAATTCAGATCAAGAAAATCGACAACGTGACGGCGAGGCAGGTGACCTTTTCGAAGAGAAGAAGAGGGCTTTTCAAGAAAGCTCATGAACTCTCAACACTCTGTGATGCCGAGATTGCTCTTATTGTCTTCTCCGCTACTGGCAGACTCTTTGAGTACGCCAGCTCTAGTATGAAGCAGGTAATTGAGAGGCATAATCTACAACCACAGAATCTTGTGCACCTAAACCAACCATCATTGGAGCTGCAGCTTGAAAATAGCACACGTGCCATGTTGAGCAAGGAAGTTGAAGAGAGGACTCTTGAATTGAGGCAACTCAGGGGAGAAGAGCTCCATGAACTTGGTTTCGAAGAATTAAAGAAACTAGAAAAATCACTGGAAGGAGGATTGAGCCGTGTCTTAAAGACAAAGCCTTTGAGAGGCAAGTTAAATTTTCAGATGCAGATAGTGAATATGGGCCAACCACAAGAACAAGGGCAGTCTTCAGAATCTATCACCAACAATGGCAGCACAGTTGCTCCTCCCCAAGACTATGACAGCTCTGATACTTCCCTCAAGTTG

>ColMADS05

ATGACTAGAAAGAAGGTTAAGCTTGCATTCATCACCAACGACTCAGCAAGAAAAGCAACATTCAATAAAAGGAAGAAGGGTCTGATGAAGAAGGCGAGCGAGCTAAGCACTCTATGTGGGATTGATGCATGCGTGATTATACATAGTCCATACAAGTCTCAACCTGAGGTTTGGCCAAACAACTTGGGAGTCCAACGCGTGCTGGCAAAGTTTGAGAGAATGCCCAAGATGGAACAAAGTAAGAAGATGGAGACCCAAGAAAGCTTCATCAGACAAAGGATAGCGAAAGCGAATGAACAACTCAAGAAAAAACTCAAGGATAATCGAGAGAAGGAGATGACAGAAATCATGTACCAGTGTTTGAGTGGGAGAGGGCTCCAAAACTTGTCCATGGTGGATCAATGCGATGTTAATTGGTTGATAGATAAAAATTTGAAGGAAATTGGAAAGAAGATTGAATCGTTGAAGAAAACTCCGCAACAAGTTGCACCGGTGGCATTGAAGAATACCAAAGAAATGCTTGGCAACGGAATGCATAGAAAGAAAGAGAAGTTATTGGGTGTTGATTTGGCCATGGATGCTATGAACAGGTCACACGGGTTCAATGAATGGATGAACAATCCACATTATGAGAATATGGGTTTTGATGGGGATAAAATGATGGTGCCATTTGGAGACAACCACAATCTTATGTGGTCTAGTTTTTTTTTCCCATGA

>ColMADS06

ATGGTGAGAGGGAAGACTCAGATGAGGCGTATAGAGAACGCTACAAGCAGGCAAGTCACCTTCTCCAAAAGGAGAAATGGGCTTCTGAAGAAGGCCTTTGAGCTCTCGGTTCTTTGCGACGCCGAGGTCGCGCTCATCGTCTTTTCTCCGAGAGGAAAGCTCTATGAATTTGCCAGTTCTAGGTACGATTTGTGTATTTATTATCTATATTATTGA

>ColMADS07

ATGGGTCGGGTGAAATTAGAAATAAAGAGAATAGAAAACAACACAAATAAACAAGTCACGTTTTCGAAGCGTAGGAATGGACTCATCAAGAAGGCCTATGAACTATCTGTGCTGTGTGACATCGATGTTGCTCTTATTATGTTCTCTCCTTCTGCTCGTCTCACTCACTTCTCCTCCCCTAAAACAAGGCTTGAAGATGTGTTTACTCGTTTCATAAATCTTTCTGATCGAGAAATGTCGAGCACACTGAATCAACTCTGGATTGAAAATGAAGTTGCTCTTCATCAAACCAATATTTTCAAGCCTGATCCATCAAATTTGACGTCTCTGGAGGAGCTTGAGTCATGCGAGAAAAAACTTGAACACATCTTGACTACCGTCACAGAGAAAAAGGAACATTTGTTAAACAACCAAGCATCATCTTCTTCTTATAATCAATCTTGTATGCAGGAGATGCTGCAGCAGCAGCAACAGACGCCAATATTTTGTGAGAACACCGTCGTTGACAGTTTGTTACCAGATAATGGCCAGAACAACCATGCCCAAATGTTCGATGCATCTGTTCCTTTTATTCCCCTCAGAGATGCTTCAAATACAGTGTATGAGCCAATGTTGCAAGGGTCAAGCTCACAAATGGATCCACAAGGCACGTGGGGGTGCCGCGTTGACCAATCAGATATTGAGAACCTTCCAGCATGGCACCATGCCTATGCTTCAACAGACCCCAACTCTATGCCACCACCACACCTATTTTCTCCAATTCAGCATGGGATGGAGGAGCCAGTCATGATGGCGACACGAAGTGATCAGGTAGAGACTGCATCGAACAGCTCACTTGAACAACCACATACCACAGCCACTAACTATGAG

>ColMADS08

ATGGAACTCATAAGAAATGAAAAGGCTCGTTATGTAACCTGTCAAAAGAGAAAGAAGGGCTTGAAAAAGAAGACTTGTGAACTAGAAACTCTATGTGATGTCCAAGTTTGTCTGATCATCTATGGACCTAAGTTGGATGATCATTCCACTGAGGTAGAAATCTGGCCCCAAAATCCCAATGATATTCAACGCTTGATTGACTCTTACAGAAACCAATCTATTGAAGATCGTCATAAGAGGACGCTTGATTTATCCAACTTTTTTGAGAACCGAAACCAAAAGATTGAAGATGCGCTTATCAAACTACGTAAGAAGAATGACAAGGCTTTGTACTCTACGTGGGATGATCGTTACAATGACTTGTCAGAGAGGCAGCTGAGAGCCTTTGAAGGTATGTTGGAGGGTAAACTTGCAGATGTGAAGGCTAGGGTTGAATTCATGAAGGGAACTCTAGCATTAGCAAGTCTTAACGATTTGGAACAAACCTCTCAACAAAATCATTCGAATTATTTCATGCAAGGCTTGTTTGGTACTAGAAGTTTGCAAATGGGGATTATCCATGAGGAGACGCATATTTCTTCGTTGAATCCGCTGTATGAAATTGACAACATCTCTCTTCATTACCCTTTTGATCAGATTGATCACCAGGGAATTGTTGAGCTTGATGCAAAATCCATGGCAATTAGTCCAATGATGATGCCAATGAACAACATTAATGACACTCATTACACTCAATTTGGTGGTATGTCTAGTAGCAACTTGCAGTGCATTGATCCACTAGAGAAATCAATTTATTACGATCCAATAATGTTGTTGCCTTCTTCAGAGCCCGTTTTAATAGAAGCCTCGTGCATTGAGTCGTCTTTTTCAATGTCTAGGATGATAGAGAACGCAGTTGGTAAAAATCATCATCCTTCGCCCTCTATGGGTTATCGTGGCGGTTCAAGCATGCAAACAATGTCGCCATATATGCAGTACCTGAAGATGACAAGTGGTCCTTCTCAAGTTCATGCATGGCAGATAGAGGAGTACTATAAAGCCATTGAGTTTCAAATGAAGAATCAGAAGTGA

>ColMADS09

ATGGGGAGAGGAAAGATAGAGATAAAAAGGATAGAAAACTCAAGCAACAGGCAAGTAACTTACTCAAAGAGGAGGAATGGGATCATGAAGAAAGCTAAAGAGATCACTGTTCTTTGTGATGCTCAGGTCTCTCTTGTCATCTTTGCCAGCTCTGGGAAGATGCATGAGTACTGTAGCCCTTCCACTACGTTGGTTGACATCTTGGATAAGTATCACAAGCAGTCTGGGGAGAGGTTGTGGGATGCTAAGCATGAGAATCTCAGCAACGAACTTGATAGAATCAAGAAAGAGAATGATAGCATGCAGATTGAGCTGAGGCACCTGAAAGGAGAAGACATAACATCTTTGCACCACAAAGAGCTCATGGCCATAGAGGAAGCCCTCGAAAATGGGCTAGGGAGTGTTCGCGAGAAACAGGCAAGTCTGATGATATTGCTAATGGAGTACATTGACATGATGGAGAAAAATAAAAAGATGTTGGAGGAGGAGAACAATCACCTCAATTTTATGTTGCATCAGCAAGAGATGAACATGGAGAGCAGCAGAGAGATGGAAAATGGATATCATCAGCGAGTGCGAGATTTCCAGTCCCAGATGCCTTTTGCCTTCCGCGTGCAGCCAATTCAGCCAAATTTGCAAGAGAGAATCTAG

>ColMADS10

ATGGGCCGCCAAAAGATTGAAATCAAGAAGATAGAGAAGAAAAGCCAACTCCAAGTCACCTTCTCCAAGCGCCGTGCCGGAATATTCAAAAAAGCCGGCGAACTTGGCGTCTTGTGCGGCGCCTACGTCGCCGTCATCGTCACCTCCCCTGCCGGCAAGGTCTTCGCCTTTGGCAACCCCTCTGTTGACTCCGTCATTGATCGCTTTCTCTCCAAAAACCCTAATCCTAACCCTAATTCCCATGGTTTCGACACGTGTCAGGATGACGAGCTCCACCAAGTGCATCAACAAAGTATTAACCCTAACCCTAATCCTAACTCTCAGTGCTCGGAGCTCCATCGAGTGAGTAAGAATCCTAACCCTAACCCTAATTCTCAGTGTTTGGACATGTGTCACGAGGAGGAGGTCCGGCAAGTGCAGCAATTGAGTAAGAAGTATGTGAAGGCGGTGGGAAAGTTGGAGGCGGAGAAGGAGAGAGGAAAGGCAGTGGCCGAGTTGAACAGCGGCGGAGGAGGAGGTGGGTTTTGGTGGGATGAGAGTGTTGAGGGATTAGAGTTGCATGAGCTTGAGCAGTATGTGGCTGCATTGGAAGTTCTTAAGAGTAACTTATTGGCTAGGGCTGATGAGATGGCGGTGGCAGCGAGCGGTTTTCCAGCGAATTTCTTGGCTTCCAACGGTGTTGTGGTGGCTGATGCTTTTGGTAGTGCTGAGTGTTTTGGTTTTGATTGTAAGCCTTTCTAA

>ColMADS11

ATGGAGAGGAAGCAAACCCAAGGCCGAAAGAAGATTGAAATGAAGATGATAGCCGATGAAAACGCAAGAAGGATTACCTTCTCAAAGCGTAGACATGGGCTTTTCAAGAAAGCAAGCGAGCTGAGTACCTTATGTGGCGTAGATATGGCCATTGTCCTCTTCTCCATGGGAGGCAAGGCTTTCTCTTTTGGAAAACCTAATGTTGACTCGGTTGTAGACCGCTTTCTGAACCAAAATGCGCAGCCAAGTGAAGGCGTCTCCAGCCATGCCTCGCGGCACGATGATGCCACTGTGCACCAACTCAACCAACAATTCCATGAGCTGAGCAAGCGATTGAAAGCCGAGAAAAAGAAAGAGAAAATTGTTCAAAACATGAGTACCAACAACTATGGCTTGCTAAGTTGTAGGTTTGATGCATATGTTAATCAGCTTGACTTGCAACAGCTTGAACAATTAAAAAGGTCTATGGTGGAGCTTAAGAAAAACGTGGCTCAGAGAGTTGATGAATTGAATTCGGAGGGTTCTTCCAAGCATGCACAAGAAGAAGAGCAAGTGGAAAACACTGAAATAAATGACAATGATGTTTCTACTATTCCCCATGATTGGTTAAGGTTGTGA

>ColMADS12

ATGGAGTTTCAGAATCAATCAATGGATGTCTCTCGTCTCTCCCCCCAGAGAAAAATGGGTAGGGGAAAGATCGAGATCAAACGGATCGAAAACACAACCAATCGGCAAGTCACCTTCTGCAAGCGCCGCAATGGTTTGCTCAAGAAAGCCTATGAGTTGTCTGTTCTATGTGATGCTGAGGTTGCCCTTATTGTTTTCTCGACCCGTGGCCGGCTTTACGAGTATGCCAACAACAGGTATTGA

>ColMADS13

ATGACTATTATACCCACTCTCTTCCTTTCCACATATGGCCTCTTCAAGAAGACCGACGAGCTCTGCAACTTGTGTGGCACCGAAGCCGCCGTCATCACCTTCTCCAACGCCGGCAACACCTTCGCTTTCGGCAACTCCTTCGTCGACTCCGTCATTGACCGTTACCTCTCTATGACTTCACCATCCTCTAAAGCCTCCGCAAGCTTCTCTTCTCTCTTTCAACATAGGGTTTCAACTCAAATCTGTGCACCATGA

>ColMADS14

ATGACTATTATATCCACTCTCTTCCCCACTCTCTTCCTTTCCACAGATGGCCTCTTCAAGAAGACCGGCGAGTTTTGCAACTTGTGCGGCACCGAAGCCGCCGTCATTACCTTCTCCAACGCCAGCAACACCTTCGCCTTCAGCAACCCCTCCGTCGACTCCGTCCTCGACCGTTACCTCTCTATGACTTCATCATCTTCCAGAGCCTCCGTAAGCTTCTCTTAG

>ColMADS15

ATGAAGTACGAGTTCATTGCTGATGAACGTGTTCGGAAGCAGACATTTCGAAGAAGAAAGGCTGGATTATTGAAAAAGGTAAGTGTGCTTGAAACTCTATGTTCTGTTGATGCATGTGCTATAATATATCCTGCTACTACAACAGGATCTGTTGCTCAACCAGATGTTTGGCCTTCTCCAAATGAAGCATCCCATTTGCTCCGAAGGTTTGAAAACTTGCCTAGCATGAAAAAAACAGCCAACATGATGGACCTAGAAAATTTTCTTAAGGAAAACATAACGAGAATGAGTAAGTATTTGGACAAGGAAAAAAAGAAAAATCGGGGGGTTGAAATTGGACAACAAATGACCAAATTCTTGTTTGAAAAAAATTTGCATGATTTTAGCAACTTGGATGATCTGAGAGATGTTGCTTCCCTTTTGGATGAGAAGATTAATTTGATGAACGATAAGCTTGAAAACATCGCAAACGAGATTGGGAGTGGATCAAGGTGTGGCGAAAAGAAGCAACAATCCATTTGA

>ColMADS16

ATGGGTCGAACAGTGAAGTACGAGTTTATTGCTGATGAACGTGTTCGGAAGCAGACATTTCGAAAAAGAAAGGCTGGATTATTGAAAAAGGTAAGTGAGCTGAAAACTCTATGTTCTGTTGATGCATGTGCTATAATATACCCTACTACTGCAACAGGATCTGTTGCTCAACCAGATGTTTGGCCTTCTGCAAATGAAGCATCCCATTTGCTCCAAAGATTTGAAAACTTGCCTAGCATGAAACAAACAGCTAACATGATGGACCAAGAAAAATTTCTTAAGCGCAACATAACGAGAATGAGTAAGAATTTGGACAAGGAAAAAAAGAAAAATCGGGGGGTTGAAATTGAACAACAAATGACCAAATTCTTGTTTGAAAAAAATTTGCATGATTTTAGCAACTTGGATGATCTGAGAGATGTTGCTTCCCTTTTGGATGAGAAGATTGATTTGATGAACGACAAGCTTGAAAACATCGCGAACGAGATTGGGAGTGGATCAAGGTGTGGCGAAGAGAAGCAACAATCCATTTGA

>ColMADS17

ATGGGTAGAGGAAAGATTGAGGTTAAGAGGATCGAAAACAACACTAGCAGACAAGTCACCTTCTCAAAACGCCGAACAGGGCTGCTCAAGAAGACCCATGAACTTTCTGTCCTCTGCGATGCGCAGATCGGACTCATCGTCTTCTCCAGCAAAGGGAAGCTCTTTGAGTACTGCACTCACCCTCTGAGCATGGGTGAAATGATAGGAAGATATCTGGATGCTACAGGGATTCGTATACCAGAGCACGATGACCGGGAACAAATATTCAATGAGTTGACAAGAATCAGGAATGAAACTCATAATCTTCAACTGAGTTTGCAGCGGTACAAAGGTGAGGACTTGAGTTCTGCACGGTACGAGGATTTGGATGAGCTTGAACGACAGCTTGAATCCTCAGTTAACAAGGTCCGAGCTCGGAAGATAAAATTTGAAATAGAAGAAGATAATTTGAGAGAGAAAGAACCAGCTAGGCCTAGCCTAATTGACCATCATGCCTTCCTTAGGGCTTAG

>ColMADS18

ATGGGGAGAGGAAAGATAGAGATAAAGAGGATAGAGAACACAAGCAACAGGCAAGTGACTTACTCAAAGAGAAGAAATGGGATCTTGAAGAAAGCTAAGGAGATCACTGTTCTATGTGATGCTCAGGTCTCTCTTGTTGTCTTTGGCAGTTCTGGCAAGATGCATGAGTTCTGTAGCCCTTCCACTACGTTGGTTGACATCTTGGAGAAGTATCACAAACAGTCTGGGAAGAGAATCTCAGCAATGAATTGGATAGAATCAAGAAAGAAAATGACAAGCTTGCAGATTGAGCTCAGGCACTTGAAAGGAGAAGACATAACATCTTTGCAGCCCAGGGATCTCATGGCCATAGAGAACGCCCTCGAAACTGGGCTTGAAAGTGTTCGCTACAAACAGTCGGAGATTCACAGGATGATGAAGAAAAATGGAAAGATGCTGGAGGAGGATAACAAGCAGCTCAATCTGATACTGCATCAGCAAGAGATGGAGAAGGGAAGTAGAGAATTTGAGAATGGATATCATCGGCAAGTGAATGACTACCAGCCCCAGATGCCTTTCACCTTCCGGGTGCAGCCCATCCAGCCAAATTTGCATGAGAGAATTTAG

>ColMADS19

ATGGGGAGGGGGAAGATCGTGATAAGGAGGATCGACAATTCTACAAGCAGGCAAGTGACGTTTTCCAAGAGGAGGAACGGATTGCTGAAGAAGGCGAAGGAGCTGGCGATCCTGTGCGACGCGGAGGTAGGAGTTATGGTGTTCTCCAGCACCGGCAAGCTCTACGATTTCTCCAGCTCCAGGTCCTCTCTCTCTCTCTTCTTCTATGTTTTTTTTTTCTCTCCCTATATTGAATGTTAA

>ColMADS20

ATGAACACCACCAAGAAAACCACCCAAGGCCGCCAGAAGATCGAAATCAAGAAGATTGAGAGCGTAAGCAATCGCCGAGTCACCTTCTCGAAACGCCGTGCCGGCCTCTTCAAGAAGGCCAGTCAACTCTGCATCCTCAGCGGCGCTGAGATCGCCATTGTCGTCAAATCTCCCGGAAAGCGCACCTTCGCTTTCGGCCAACCCAGCGTCGACGCCGTGATCGATCGATACCTCACCAGAACCTCCGCCGCCGCCGAGCAGAACAACCGCCCTTCGGTGCTCGATTTCAACGAGAAATACGCCCAAGTTTCGAAGGAATTGGAGGCGGAGAAGAGGCAGAGAGCTGTGATTGAGGAGACAAAGAAGGCGGCGAATGACGGCGGATTTTGGTGGGATGAGGCGGTTGATGATCTAGGATTAGAGGAGCTGGAGCAGTACGTGGCGGCGTTGGAGGAGTTGAAGAAGATTGTGAGTATGAAAGCTGATGAGTTGATGATAATGAAAGCGAATTCTTCAATGATGTTTGGTATGAATGAAAGTTCGAATCATGGTGGGCTGGGTTTAATGAGTGATTGTGCTACTTCTACGGTTCCTCATGGGTTTGATTGTGCGAATTCTTCATTGATGTTTGGTATGAATGAAAGTTCGAATCATGGTGGGCTGAGTTTGATGAGTGATTGTGCTACTTCTACGGTTCCTCATGGGTTTGATTGTGCGAATTCTTCAATGATGTTTGGTATGAATGAAAGTGCGAATCATGGCGGACTGGGTTTTATGAGTGATTGTGCTACTTCTACGGTTCCTCATGGGTTTGATTGTGCGAATTCTTCAATGATGTTTGGTATGAATGAAAGTTCGAATCATGGTGGGCTGAGTTTGATGAGTGATTGTGCTACTTCTACGGTTCCTCATGGGTTTGATTGTGCGAATTCTTCAATGATGTTTGGTATGAATGAAAGTGCGAATCATGGCGGACTGGGTTTTATGAGTGATTGTGCTACTTCTATGATTCCTCATGGGTTTGATTTTGGACGTGGCCAGTTCTGA

>ColMADS21

ATGGTCACCATGAACACCACCAAGAAAACCACCCAAGGCCGCCAGAAGATCGAAATCAAGAAGATTGAGAGCGTAAGCAATCGCCGAGTCACCTTCTCGAAACGCCGTGCCGGCCTCTTCAAGAAGGCCAGTCAACTCTGCATCCTCAGCGGCGCTGAGATCGCCATTGTCGTCAAATCTCCCGGAAAGCGCACCTTCGCTTTCGGCCAACCCAGCGTCGACGCCGTGATCGATCGATACCTCACTGGAACCTCCGCCGCCGCCGAGCAGAACAACCGCCCTTCGGTGCTCGATTTCAACGAGAAATACGCCCAAGTTTCGAAGGAATTGGAGGCGGAGAAGAGGCAGAGAGCTGTGATTGAGGAGACAAAGAAGGCGGCGAATGACGGCGGATTTTGGTGGGATGAGGCGGTTGATGATCTAGGATTAGAGGAGCTGGAGCAGTACGTGGCGGCGTTGGAGGAGTTGAAGAAGATTGTGAGTATGAAAGCTGATGAGTTGATGATAATGAAAGCGAATTCTTCAATGATGTTTGGTATGAATGAAAGTTCGAATCATGGTGGGCTGGGTTTAATGAGTGATTGTGCTACTTCTACGGTTCCTCATGGGTTTGATTGTGCGAATTCTTCAATGATGTTTGGTATGAGTGAAAGTTCGAATCATGGTGGGCTGAGTTTGATGAGTGATTGTGCTACTTCTACGGTTCCTCATGGGTTTGATTGTGCGAATTCTTCAATGATGTTTGGTATGAATGAAAGTGCGAATCATGGCGGACTGGGTTTTATGAGTGATTGTGCTACTTCTACGGTTCCTCATGGGTTTGATTGTGCGAATTCTTCAATGATGTTTGGTATGAATGAAAGTTCGAATCATGGTGGGCTGAGTTTGATGAGTGATTGTGCTACTTCTACGGTTCCTCACGGGTTTGATTGTGCGAATTCTTCAATGATGTTTGGTATGAATGAAAGTGCGAATCATGGCGGACTGGGTTTTATGAGTGATTGTGCTACTTCTATGATTCCTCATGGGTTTGATTTCGGACGTGGCCAGTTCTGA

>ColMADS22

ATGAACACCACCAAGAAAACCACCCAAGGCCGCCAGAAGATCGAAATCAAGAAGATTGAGAGCGTAAGCAATCGCCGAGTCACCTTCTCGAAACGCCGTGCCGGCCTCTTCAAGAAGGCCAGTCAACTCTGCATCCTCAGCGGCGCTGAGATCGCCATTGTCGTCAAATCTCCCGGAAAGCGCACCTTCGCTTTCGGCCAACCCAGCGTCGACGCCGTGATCGATCGATACCTCACCGGAACCTCCGCCGCCGCCGAGCAGAACAACCGCCCTTCGGTGCTCGATTTCAACGAGAAATACGCCCAAGTTTCGAAGGAATTGGAGGCGGAGAAGAGGCGGAGAGCTGTGATTGATGAGGCGGTTGATGATCTAGGATTAGAGGAGCTGGAGCAGTACGTGGCGGCGTTGGAGGAGTTGAAGAAGAATGTGAGTATGAAAGCTGGTGAGTTGATGATAATGAAAGCGAATTCTTCAATGATGTTTGGTATGAATGAAAGTTCGAATCATGGTGGGCTGGGTTTGATGAGTGATTGTGCTACTTCTACGGTTCCTCACGGGTTTGATTGTGCGAATTCTTCAATGATGTTTGGTATGAATGAAAGTGCGAATCATGGCGGACTGGGTTTGATGAGTGATTGTGCTACTTCTACGGTTCCTCACGGGTTTGATTGTGCGAATTCTTCAATGATGTTTGGTAGGTTTGATGAGTGA

>ColMADS23

ATGGGTCGAATGGTGGATTACGAGTTTATTGCTGATGAACGTGTTCGGAGGCAAACGTTTCGGAAAAGAAAGGCTGGATTGTTGAAGAAGCTGAGCGAGCTTAAAGCTCTATGTTCTGTTGATGCATTTGCTATAATATATCCTACTACTACAACTGGATCTGTTGCTCAACCAGATGTTTGGCCTTCTCCATCTGAAGCAAACCATTTGCTGCAAAGGTTTAAAAACTTGCCTAGTCCGCAACAAGCAGCTAACATGATGAACCAAGAAAAATTCCTTAGGCAAAACATATTGAGAGTGAGTAAGAATTTCGACAAGGAAAAGCAGAAAAATCGGCGGGTTGACATTGAACAACAAATGACCAAATTATTGTTTGAAAAAAATTTGCATGATTTTAGCAACTTGGATGATCTGAGAGATGTTGCTTCCCTTTTGGATGAGAAGATTAATCTGGTGAACGATAAGATTGAAAACATCACGCGTGATGAGAATGGGAACGAGATTAGGAGTGGATCAAGGCGCGGCAAAGAGAAACAACAATCCATTTGA

>ColMADS24

ATGGGAAGCAGAAGAAAGAAAATTGAGATTAAAAGAGTAGAGAAGGAAGGGCAGAGAATGGTTACATTCTCCAAGAGACGCCATGGTCTCTTCAACAAAGCCCGACAACTCCGATCCCTCACCGGCGCTGACATCGCTATCCTCACCTTCTCCCCCGCTGGCCGTCCCTACACTCACGGTGAACCCTCCTTCGATGCCCTTGTCGACCGTTACCTCAATACCGTCGCCGCCGGAGAGAAGGCAGAAGAGGGTTGTGAGGCTGCCGCCGCGACCCACCACCGGTTGAGTTCGTGGTTGGATGCTCTGCAATTCGATGCAAGTGATAGCATTGAAGATTTGGAAATCTTGAAGAAAGGGCTTGAAGAGATTGCAGCGAAAGTGACTAAGAAAATTGATGACGTTTTTGTTGATTCTTTGCTCGTTTGA

>ColMADS25

ATGGTTACATTCTCCAAGAGACGTCAGGGTCTCTTCAACAAAGCCCGACAACTCCGATTCCTCACCGGCGCCGACATTGCCATCCTCACCTTCTCCCCCGCTGGCCGTCCCTACACTCACGGCGAACCCTCCTTCGACGCCCTTGTCGACCGTTACCTCAACACCGCCGCCGCTGGAGAGAAGGCAGAAGAGGGTTGTGAGGCTGCCAGCACGAACCACCACCAGTTGAGTTCGTGGTTGGATGCTCTGCAATTCGATGCGAGTGATAGCATTGAAGATTTGGAAATCTTGAAAAAAGGGCTCGAAGAGATCACAACAAAAGTGGCTGAGAAAGTTGATGACCTTTTTGTTGATTCTTTGCTCTTTTGA

>ColMADS26

ATGTACAAGAAGAGAAAAGCCTGTATCATCAAGAAAACAATGGAGCTCTCGATATTGTGCGACATCAATGCCTTCACCATCTGTTTTGGGCCCAACAATGAGGTTGAGACATGGCCTGAAAACCCAACCCACGTCAAAACTCTCATCAACGCGTACAAGAATAACCGCCGAGGCAAACAACTGGTTCAAGCCCAACAGCAACAACCCTCTGATCATCATCGTGGCTTAGATGATAAAATCAGGGGTTTATTGGGTTGGAATGATGGTTGGCTTTGTGGGTTGTCAAGAGAACAGTTGATGAGTTTATGGAAATTGATGAGATTGAAATTACAGGAATTAACAAGAAGAATTGAGTTGTTGATGTTCAGTAAGATGCACTACCAGTACCAACCATTAATTGAGCCACCGATGTTCGTGCCGCTTTGGCCATTGAATTGCTATGGAGTTCCTGGAAGTAGTGGTGGTGATGGTGGTGAGTTGAGTGTGCCATTGACATGGGAGTTTAATGAAACATTGGAGGAGACTACTCTTAATGCCATGAACAACTCTGATATTAATCAGATGTGTTTGCAATTTTCTTCGCCTCTCAATGAACTTCTATTTGCGTCAATGTATACGATGAATCCGCTGCAAATCACTGAGCCTCAGTTGGGTGAAGAAACAATCAACAGCAGTGACACTGACTCTCAAGCCAATCCCAGTCTCAATGTGAACATGGATTGGATTGTTTAG

>ColMADS27

ATGTACAAGAAGAGAAAAGCCTGTATCATCAAGAAAACAATGGAGCTCTCGATATTGTGCGACATCAATGCCTTCACCATCTGTTTTGGGCCCAACAATGAGGTTGAGACATGGCCTGAAAACCCAACCCACGTCAAAACTCTCATCAACGCGTACAAGAATAACCGCCGAGGCAAACAACTGGTTCAAGCCCAACAGCAACAACCCTCTGATCATCATCGTGGCTTAGATGATAAAATCAGGGGTTTATTGGGTTGGAATGATGGTTGGCTTTGTGGGTTGTCAAGAGAACAGTTGATTTGTTTATGGAAATTGATGAGATTGAAATTACAGGAATTAACAAGAAGAATTGAGTTGTTGATGTTCAGTAAGATGCACTACCAGTACCAACTATTAATTGAGCCACCGATGTTCGTGCCGCTTTGGCCATTGAGTTGCTATGGAGTTCCTGGAAGTAGTGGTGGTGGTGCGTTGAGTGTGCCATTGACATGGGAGTTTAATGAAACATTGGAGGAGACTACTCTTAATGCCATGAACAACTGTGATATTAATGCCATGAACAACTATGATATTAATCAGATGTGTCTGCAATTTGCTTCGCCTCTCAATGAATTTCTGTTTGCATCAATGTATACGATGAATCCGCTGCAAATCACTGAGCCTCAGTTGAGTGAAGAAACAATCAGCAGCAGTAACACTGACTCTCAAGCCAATCCCAGTCTCAATGTGAACATGGATTGGATTATTTAG

>ColMADS28

ATGTACAAGAAGAGAAAAGCCTGTATCATCAAGAAAACAATGGAGCTCTCGATATTGTGCGACATCAATGCCTTCACCATCTGTTTTGGGCCCAACAATGAGGTTGAGACATGGCCTGAAAACCCAACCCACGTCAAAACTCTCATCAACGCGTACAAGAATAACCACCGAGGCAAACAACTGGTTCAAGCCCAACAGCAACAACCCTCTGATCATCATCGTGGCTTAGATGATAAAATCAGGGGTTTATTGGGTTGGAATGATGGTTGGCTTTGTGGGTTGTCAAGAGAACAGTTGATGAGTTTATGGAAATTGATGAAATTGAAATTACAGGAATTAACAAGAAGAATTGAGTTGTTGATGTTCAGTAAGATGCACTACCAGTACCAACCATTAATTGAGCCACCGATGTTCGTGCCGCTTTGGCCATTGAATTGCTATGGAGTTCCTGGAAGTAGTGGTGCTGATGGTGGTGCTGGTGGTGAGTTGAGTGTGCCATTGACATGGGAGTTTAATGAAACATTGGAGGAGACTACTCTTAATGCCATGAACAACTGTGATATTAATCAGATGTGTCTGCAATTTTCTTCGCCTCTCAATGAACTTCTGTTTGCGTCAATGTATACGATGAATCCGCTGCAAAACACTGAGCCTCAGTTGGGTGAAGAAACAATCATCAGCAATGACACTGGCTCTCAAGCCAATCCCAGTCTCAATGTGAACATGAATTGGATTATTTAA

>ColMADS29

ATGTACAAGAAGAGAAAAGCCTGTATCATCAAGAAAACAATGGAGCTCTCGATATTGTGCGACATCAATGCCTTCACCATCTGTTTTGGGCCCAACAATGAGGTTGAGACATGGCCTGAAAACCCAACCCACGTCAAAACTCTCATCAACGCGTACAAGAATAACCGCCGAGGCAAACAACTGGTTCAAGCCCAACAGCAACAACCCTCTGATCATCATCGTGGCTTAGATGATAAAATCATGGGTTTATTGGGTTGGAATGATGGTTGGCTTTGTGGGTTGTCAAGAGAACAGTTGATGAGTTTATGGAAATTGATGAGATTGAAATTACAGGAATTAACAAGAAGAATTGAGTTGTTGATGTTCAGTAAGATGCACTACCAGTACCAACCATTAATTGAGCCACCGATGTTCGTGCCGCTTTGGCCATTGAATTGCTATGGAGTTCCTGGAAGTAGTGGTGGTGGTGGTGGTGGTGAGTTGAGTGTGCCATTGACATGGGAGTTTAATGAAACATTGGAGGAGACTACTCTTAATGCCATGAACAACTGTGATATTAATCAGATGTGTCTGCAATTTGCTTCGCCTCTCAATGAACTTCTGTTTGCATCAATGTATACGATGAATCCGCTGCAAATCACTGAGCCTCAGTTGAGTGAAGAAACAATCAGCAGTAGTGATACTGACTCTCAAGCCAATCCCAGTCTCAATGTGAACATGGATTGGATTATTTAG

>ColMADS30

ATGTACAAGAAAAGAAAAGCCTGTATCATCAAGAAAACAATGGAGCTCTCGATCTTGTGCGACATCAATGCCTTCACCATCTTTTTTGGGCCCAACAATGAGGTTGAGACATGGCCTGAAAACCCAACCCACGTCAAAACTCTCATAAACACGTACAAGAATAACCGCCAAGGCAAACAACTGGTTCAAGCACAACAGCAACAACCCTCTGATCATCGTGGCTTAGATGATAAAATCAGGGGTTTATTGGGTTGGAATGATGGTTGGCTTTGTGGGTTGTCAAGAGAACAGTTGATGAGTTTATGGAAATTGATGAAATTGAAATTACAGGAATTAACAAGAAGAATTGAGTTGTTGATGTTCAGTAAGATGCACTACCAGTACCAACTATTAATCGAGCCACCGATGTTCGTGCCGCTTTGGCCATTGAATTGCGATGGAGTTCCTGGAAGTAGTGGTGGTGGTGGTGGTGGTGAGTTGAGTGTGCCATTGACATGGGAGTTTAATGAAACATTGGAGGAGACTACTCTTAATGCCATGAACAACTGTGATATTAATCAGATGTGCCTGCAATTTTCTTCGCCTCTCAATGAACTTCTGTTTGCGTCAATATATACGATGAATCCGTTGCAAAACACTGAGCCTCAATTGGGTGAAGAAACAATCAGCAGCAGTGACACTGACTCTCAAGCCAATCCCAGTCTCAATGTGAACATGGATTGGATTATTTAG

>ColMADS31

ATGTACAAGAAGAGAAAAGCCTGTATCATCAAGAAAACAATGGAGCTCTCGATATTGTGCGACATCAATGCCTTCACCATCTGTTTTGGGCCCAACAATGAGGTTGAGACATGGCCTGAAAACCCAACCCACGTCAAAACTCTCATCAACGCGTACAAGAATAACCGCCGAGGCAAACAACTGGTTCAAGCCCAACAGCAACAACCCTCTGATCATCATCGTGGCTTAGATGATAAAATCAGGGGTTTATTGGGTTGGAATGATGGTTGGCTTTGTGGGTTGTCAAGAGAACAGTTGATGAGTTTATGGAAATTGATGAGATTGAAATTACAGGAATTAACAAGAAGAATTGAGTTGTTGATGTTCAGTAAGATGCACTACCAGTACCAACTATTAATTGAGCCACCGATGTTCGTGCCGCTTTGGCCATTGAATTGCTATGGAGTTCCTGGAAGTAGTGGTGGTGATGGTGGTGGTGGTGGTGGTGGTGGTGCGTTGAGTGTGCCATTGACATGGGAGTTTAATGAAACATTGGAGGAGACTACTCTTAACGCCATGAACAACTGTGATATTAATGCCATGAACAACTGTGATATTAATCAGATGTGTCTGAAATTTGCTTCGCCTCTCAATGAACTTCTGTTTGCATCAATGTATACGATGAATCCGCTGCAAATCACTGAGCCTCAGTTGAGTGAAGAAACAATCAGCAGCAGTGACACTGACTCTCAAGCCAATCCCAGTCTCAATGTGAACATGGATTGGATTATTTAG

>ColMADS32

ATGACAAGAAATAAGATTAAACTTGCATGGATAGTCGATAAGTCTTCGAGGAAAGCCACTCTCAAAAGAAGAAGAGCTAGCATATTCAAGAAGGCCGGAGAACTGTCTGTCCTATGCGATGTGGAAGTTGGTGTTATCGTTTACAGTCACAAGGAAGCTGATCTTGCTGTTTGGCCATCTTACGGGCACATGAAACAAATGTTTGAAAGGTTCCTGAGTATCCCAATAGTAGAACGAAACCAGAAAATGATGACAAATGAAGGCTTCCTCACACAAAGGGTCACGCAGGAGACCAATAAGAACAATATGGAAAAGAAGAAGAATGATAAGAAAGAAATTCAGGAAATCATGAATCAAGTCTTTGAAGGTAGCAATCTATATCAACTTAACATCATGAGACTAAATTATTTGTCATTGTTGACAGTTGATAAACTGAAGCAGCTTGAAGAAAGAGAAAGAGCACGAGGACAACAAGCTCCTCCTCTATTGCCACTTCTCATTCCTGCTCCTGCTCTTGGTCCAGCTCCTGGAATGATGGAAGTGAATGAAGTTTCCCAAGCTCACACCAGTATTGAAGCAACATCGATAGAGCAGTTGATGAATGACAACTGGTTCATGGAAACAATGCCTCTTAACTGGGACATGCTAGGTCCAAGTGGGGTGAGTGACATGGGGATGCCATCAGCGCCTGTTGTTGGCAGAATGAATGGTGATAATGAAGAGGATTTGCCAGATGACCTAAATGCATTCTTTCCTCACATTTACTCCCCTTAA

>ColMADS33

ATGGGAAGAGGAAAAATTGTCATCAGAAGAATTGACAATTCGACAAGCAGGCAAGTGACTTTCTCAAAGCGCAAAAATGGATTGCTCAAGAAAGCCAAGGAACTATCAATTCTTTGTGATGCCGAAGTTGGAGTGATTGTCATCTCCAGTACTGGGAGGCTCTATGAATTTGCAAGCCCTAGCATGAGATCAATATTTGAACGATACAACAATGAGCAGGAGAATCATCAGCTTTTGAGTCCTACTTCAGAAGCCAAGCTTTGGCAAAGGGAGGCAGAAAGCTTAAGGCAACAATTGAACTACTTACAAGAAAGCCACAGAAAATTGACGGGAGAAGAACTTTCGGATTTGAGTGTGGAAGATCTGGAAAATCTGGAAGGCCAATTGGAAACAAGCTTAAAGGGTATCCGAACAAAAAAGCACCAAATTTTAACTGATGAAATACAAGAATTAAACCACCAGGGAGTTATTAGTCATCAAGAAAATATGAAATTGTATACGGAGGTTAAAGTCCTGCGACGAGAAAATGCAGAATTACATAAAAAGGTTTATGGAGCAAACGACATAAACAGAAGTTCCTTCAAACCATATGGTTGCAGCAATGGATATGACTTACATGTACCTATACAACTACAGCTAAGCCAACCGCAACAAACACACAATGAAACACCAGAAACGCAAGGAATCTAG

>ColMADS34

ATGGCTAGAGGAAAGGTTCAGATGAAGAGGATCGAAAACCCGGTGCACAGACAAGTCACCTTCTGCAAGCGCCGAGCTGGGCTTCTTAAGAAGGCTAAGGAGCTCTCTGTGCTTTGTGATGCTGAAATTGGTGTTCTCATTTTCTCTGCCAATGGCAAGCTCTACGAACTCGCCACTAAAGGAACCATGCAAGGGCTTGTTGGGAGGTACATGAAATCAACCGGAGACACTCAGGCCGACCACGATGAAGAAAAGCAAGTTATGGACTCAAAAGAGGAGATCAACATGCTGAAAAATGAAATTGAATTTCTGAAGAAGGGACTCAGGTTTATGTCTGGAGGGGGAGTTGAAGCAATGACACTAGACGAGTTACACATGCTTGAAAAACATCTTGAAATTTGGATTTATCATATCCGCTCAGTAAAGATGGACATCATGTCTCAAGAGATCCAATTATTGAAGACTAAGAAGCACATGAAGCAAAATGACCAAGTTACTGCTGCTAAAGCTGAGGCCATTGTTAAGGTCGTTGAAAAGAAAGCTGCCAAAGTTGAGAAGAAACTAACAGAGGCACTAACCAACAAGGAGCAGACGTCAACGCCTATCAAGAAAGCCAAGACTCTGTCCAAGATCAATATAAGGTTCAAGTCAACATCTCGAATATAA

>ColMADS35

ATGGGAAGAGGGAAGGTTCAGTTGAAGCGAATCGAAGATAAGAACAGTCGGCAAGTGACATTCTCGAAGCGACGTACCGGATTGATCAAAAAAGCTCACGAACTCTCCATACTCTGCGATGTCGAGATCGCTCTCATCGTCTTCTCCGCTAGAGGCAAGCTCTACCAGTTCTGCACTGGCGACAGTTTAAGAAAAGTCCTAGAGCGCTATCAGATTCACAAAGATGCAGAAGTTGCTGGCAGTAGTGTTCAAGAATCCAAGAAGCTTACGGAGGGATATATGGATTTCAGTAGAGGCACCAACCTACTTCAAATGGTTCAAAGGCACTTTGAAGAGCAAAAGATTGAGCAGCTAGATGTGGCAGAGCTCACTCAAGTAGAACACCAATTAGATGCTATACTCCGCCAAACCAGAATAAAAAAGTCACAGCTAATGATGAAAGCTGTCACGGCTCTCCATGAAAAGGAGGAACAGCCAAGAGAGGGAAGGCAATTAATGGAAAAGGAGATCACAGCAATGATCAATGAGGCCACCATGGACGACGATTGCCGCCACCAGCAGCAGCAGACGCAGCAGGGGGACCCAGATATGGACCTGGAGCTGTATGGCTACACTAACAATACAAACAACAACAACAACAGTAGTACAGGTAGCGGCGGTGGTGGTGTGTATCATCATCTCCAACAAGAAGAAAGTATGTTTTATTTGCTTTGA

>ColMADS36

AATGATGATGATGAAGGATTTGAGTTCGGTGTGGAGATCAAGGTTGGTAAAGGATACGACGACAGGTTCGTTGTCTTCTTCGTTTTTGAGATCATCGTGCTTGAGCAAAACTCCTTTGAGAGAGCCAAATCGAAGAGCAGAGATAGTCTAGTTCTTGTCAAATCCAACTGCCCAGAAGAAATATGCACCGAGGCCATTCGCTCGAGCGAAGTTCACCTTGTTGCTGATCGATTCCGTCAAACAAAGGAACGAAACAGCAGCAGACAAACAGAGCAGAGCACACGGACGGAGCAGCAGCAAGCACAGATGGGTCGAGCTAAACAAAGAATGGAACTCATAAGAAACGAAAAGGCTCGCTATGTAACCTATCAAAAGAGAAAGAAGGGCTTGAAGAAGAAGACTTGTGAACTGGAAACTCTATGTGATGTCCAAGTTTGCCTAATCATCTATGGACCTAAGTTGGATGATCATTCCTCTGAGGTAGAAATCTGGCCCCAAAATCCCAATGATATTCAGCGCTTGATTGACACCTATAGAAACCAATCTATTGAAGATCGTCATAGGAGGATGCTTGATTTATCCAACTTTTTTGAGAACCGAAGCCAAAAGATTGAAGACGCGCTTGTCAAACTACGTAAGAAGAATGACAAGGCTTTGTACTCTACGTGGGATGATCGTTACAATGACTTGCCGGAGGGGCAGCTGAGAGCCTTTGAAGGTATGTTTGAGGGTAAGCTTGCAGATGTGAAGGCCAAGGTTGAATTCATGAAGGGAACTCAAGCATTGGCAAGTCTTAACGATATGGAACAAACCACTCAACAAAACCATTCGAATTATTTCATGCAAGGCTTGTTTGGTACAACAAGTTTGCAAATGGGGATTATCCATGAGAAGACGCCTATTTCTTCGTTGAATCCGCTGTATGAAATTCATGGCATCTCTCTTCATTACCCTCTTGGTCAGACTGATCACCAGGGAATTGTTGAGCTTGATGCAAAATCCATGGCAATTAGTCCAATGATGATGCCAATGAACATTGACAATCATTACACTCAACTTGGCGGTATGTCTAGCAACAACTTGCAGTGCATTGATCCACTAGAGAAGTCAATAAATTATGATCCAATAATGTTGTTGCCCTCTCCAGAGTCTGTTTTAACAGGAGTCTCATGCACTGAGTCGTCCTTTTCAATGTCTAGGATGATGGAGAACGAGGTTGGTAAAAAGCATCATCCTTCGCCCTCTGTGGGTTACTATGGCGGTTCAAGCATGCAGACAATGTCTATAGCGACCCCGTACCTGAAGATGGCAAGTGGTCCTTCTCAAGTTCATGCATGGCAGATGAAGGAGTACTATAAAGCCAATGAGTTTGAGACGAAGAACCAG

>ColMADS37

ATGGAACTCATAAGAAACGAAAAGGCTCGCTATGTAACCTATCAAAAGAGAAAAAATGCCTTGAAGAAGAAGACTTGTGAACTAGAAACTCTATGTGATGTCCAAGTCTGCCTGATCATCTATGGACCTGAGTTGGATGATTATTCCACCGAGGTAGAAATCTGGCCCCAAAATCCCAATGATATTCAACGCTTGATTGACTCCTACAGAAACCAATCTATTGAAGATCGTCATAGGAGGACGCTTGATTTATTCAACTTTTTTGAGAACCGAAACCAAGAGATTGAAGACGCGCTTGTCAAACTGCGTAAGAAGAATGACAAGTCTTTGTACTCTATGTGGGATGATCGTTACAATGACTTGCCAGAGGGGCAGCTGAGAGCCTTTGACGGTATGTTGGAGGGTAAGCTTGTAGATGTGAAGGCTAGGGTTGAATTCATGAAGGGAACTCGAGCATTGGCAAGTCTTAACGATATGGAACAAACCACTCAACAAAACCATTCGAATTATTTCATGCAAGGTTTGTTTGGTACTACAAGTTTGCAAATGGGGATTGTGCATGAGGAGACGCCTATTTCTTTGTTGAATCCGCTGTATGAAATTGATGACGTCTCTCTTCATTACCCTTTTGATCAGACTGATCACCAAGGAATTGTTGAGCTTGATGCAAAATCCATGGCAATTAGTCCAATGATGATGCCAATGAACAACATTAATGACACTCATTACACTCAACTTGGCGGTATGTCTAGCAGTAGCTTGCAGTGCATTGATCCACTAAAGACGTCGATATATTATGATCCAATAATGTTGTTGCCCTCTCCAGAGCCTGTTTTAACAGGAGTCTCATACACTGAGTCGTCCTTTTCAATGTCTAGGATGATGGAGAACGAGGTTGGTAAAAATCATCATCCTTCACCCTCTATGGGTTACTGTGGCACTTCAAGCATGCAAACAATGTTGCCATATATGCAATACCTGAAGATGGCAAGTGGTCCTTCTCAAGTTCATGCATGGCAGATGGAGGAGTACTATAAAGCCAATGAGTTTCAGATGAAGAACCAGAAGTGA

>ColMADS38

ATGTTACGCAACAAGAAATATGCACCGAGGCCATTCGCTCGAGCGAAGTTCACCTTGTTGCTGATCGATTCCGTGTCATCATACCCAATCCAACTGATTCCTGCATATGAGTACGTGGACAAGCAAACAAAGGAACGAAATAGCAGCACACAAACAGAGCAGAGCACACGGACGGAGCAGCAGCAAGCACAGACCCCCATTTTAATGTTCGCCTTGGGCCCTGAAAAACTCAAGGCCAGCCCTGGTCACAACCCCACTAACAGGGCCCTAACCAATGAGAATGATATGGGTCGAGCTAAACAAAGAATGGAACTCATAAGAAACAAAAAGGCTCGCTATGTAACCTATCAAAAGAGAAAGAAAGGCTTGAAGAAGAAGACTTGTGAACTGGAAACTCTATGTGATGTCCAAGTTTGCCTAATCATCTATGGACCTAAGTTGGATGATCATTCCACCGAGGTAGAAATCTGGCCCCAAAATCCCAATGATATTCAGCGCTTGATTGACACCTATAGAAACCAATCTATTGAAGATCGTCATAGGAGGACGCTTGATTTATCCAAATTTTTTGAGAACCGAAACCAAAAGATTGAAGACGCACTTGTCAAACTACGTAAGAAGAATGACAAGGCTTTGTACTCTACGTGGGATGATCACTACAATGACTTGCCGGAGGGGCAGCTAAGAGCCTTTGAAGGTATGTTGGAGGGTAAGCTTGTAGATGTTAAGGCCAAGGTTGAATTCATGAAGGGAACTCGAGCATTGGCAAGTCTTAACAATATGGAACAAACCACTCAACAAAACCATTCGAATTATTTCATGCAAGGCTTGTTTGGTACAACAAGTTTGCAAATGGGGATTATCCATGAGAAGACGCCTATTTCTTCGTTGAATCCGCTGTATGAAATTGATGGCATCTCTCTTCATTACCCTTTTGGTCAGACTGATCACCAGGGAATTGTTGAGCTTGATGCAAAATCCATGGCAATTAGTCCAATGATAATGCCAATGAACAACATTAATGACAGTCATTACACTCAACTTGGCGGTATGTCTAGCAGCAACTTGCAGTGCATTGATCCACTAGAGAAGTCGATATATTATGATCCAATAATGTTGTTGCCCTCTCCAGAGTCTGTTTTAACAGGAGTCTCATGCACTGAGTCGTCCTTGTCAATGTCTAGGATGATGGAGAACGAAGTTGGTAAAAATCATCATCCTTCGCCCTCTGTGGGTTACTATGGCGGTTCAAGCATGCAGACAATGTCACCATATATGCAGTACCTGAAGATGGCAAGTGGTCCTTCTCAAGTTCATGCATGGCAGATGAAGGAGTACTATAAAGCCAATGAGTTTGAGATGAAGAACCAGAAGTGA

>ColMADS39

ATGGGGAGAGGAAGAGTCCAGCTGAAGAGGATCGAGAACAAGATCAACAGACAAGTTACTTTCTCAAAACGCCGTTCTGGACTTCTCAAAAAAGCTCACGAGATCTCAATCCTCTGCGATGCTGAGATCGCTCTCATCATCTTCTCCACCAAAGGAAAGCTCTTCGAATACTCCACGGATTCCTGCATGGAAAGGATTCTTGAGCGATATGAGAGGCATTTGATGGGTGAAGACCTCGATACCTTGAATCTTAAAGAGCTTCAGAATTTGGAGCACCAGCTCGACTCTGCTCTTAAACATATAAGGACAAGAAAGAACCAACTAATGTACGAATCCATCTCTGAGCTTCAAAAAAAGGATAAGGCATTGCAGGAGCAAAACAACTTGCTTACAAAGCAGATTAAGGAAAAGGAGAAGAAGGAGAAGGAACAGCAGGCTCAACTGGAGCAGCACCAAAACCATGATCTGAATTCATCCTCTGTTGTTATCTCACAGTCATTGCACTCCTTGAACATTGGTGGTGCTTACCAGGCAGCTGTTGGAGATGTAGAAGGAGCTCCGCATCAAATTCAAAACAATGCAGTAATGCCACCATGGATGATTAGTCACATCAATGGATAA

>ColMADS40

ATGGGTCGTGTTAAACTTCAAATTAAGAGAATAGAAAACAACACAAACCGGCAAGTTACTTTCTCCAAACGTAGAAATGGACTCATCAAGAAGGCTTATGAATTATCAGTTCTTTGTGACATTGATATAGCTCTCATCATGTTTTCTCCCTCTGGTCGTCTCAGTCATTTCTCTGGCAAGCGAAGAATTGAAGATGCACTTAGCCGTTACGATTTCCAACAAGAAATCAACAATTTGCAACAACAGCTCCAGATGGCTGAGGATCAACTAAGTATATTTGAGCCGCACGTATTAGGGTTTACATCTATGGAGGAACTTGAGTCATGTGAAAAGAACCTCTTGGAGTCAATGAACCGCGTCATGCAAAGAAAGAAATATTTAATGGGCGATCATTTGTCCACATTTGATATGCAGATGAATTCAATGGGGCAGATGTTCTTTGATGCACAAGAGGAGGGGATGCCAACAGCCTCATTCCAGAATGAATTAGTTTGTTGGCTGCCAGAGAATAACAATGGAGGAGACAATCAAAACCATATTTTTGGTGCTTCTACTTCTGATCATTCTTGTATTCGTCTAGGGAGCAATTCGGCAGCAACAATTTACAATGAAATACCGAATAATGCATCAGCAGGGGGTGTAAATGGGGAGGCAAACAGCATCGGAAGTGGAGGTTACCCAAGTGATGATCACAACAGCCTTTCACACCAGTGGCACCATGCCTCTACTTCAACTCATGATCTCTTCTCATCACTCATTCCACCAACCTCCTTCCCTCAAAATGACATGGCGAGTCCAAACGTAGGAGCAGTGGAGCAACTTCAACAGGTAGAGGCCACGCAGAGCTGCCCAGGCCCCCCGGAGGTGGAGTTGGAGGTGGAGCTGCCATCTATTGAACAAGATGCAAAATTTTGA

>ColMADS41

ATGGGTAGAGCTAAACAAAACATGGAACTCATACGCGATGACAAGACTCGATATGTGACCTTTCAAAAGAGAAAGAAGGGGTTGAAGAAGAAGACTTACGAATTACAAACCCTCTGTGATGTTGAAGTGTGTTGGATTATTTATGGTCCTAAGATCAATGACCGTCCTACAGATGATCAAGCAGAAATCTGGCCCCCAAATCCCGACGTAATTCAGCACTTGATCGAGACCTACAAAAACCTATCTAGTGAAGATCGCACGAGGAGAACCCTTGGTTTGCCCAACTTTTTTGAGGATAGGAATCAAAAGATTGAAGAAATGCTTGTGAAACTACAAAAGAAAAATGATGAGGCTAGGTACTCTACGTGGGATGATCGATACAATGGTTTGTCGAAAGAACAACTCAAAGAATTTGAAAGTGTTTTGGAGGGAAAGCTTCAAGATGTGAAGGCTAAGGTTGCATTCATGAAGGGTAGTGATCAGAAATTGTTAGTAGCATGTATCGATGACAATTCTCACACTCAAACCATCCAAAACCAATCTTATTTCATGCATGGAGGCATGTTTGGGATAGAAGTTGACAATGACATCGCTAATTTACAGTACTATTTCAATCAGGCTCAGGTTGATGCAAATTCCAAAGCGAATCCAATGATGATGACGACGATGATGCCGACGATGATGCTGAGGAACAATAATCAATATTACAATAAACTTGGCGGCGTGTCTAGCAGTAACTTTATGTGCAATCCACTAGATAGGCCAATTTATTATGATCCGATGATGTTGGAGAATGCTATTGTTAACAATCATTTGCCACCTGTGGGCTATTGCAATCTGAGCATACAGACAACGTCACCGTCATCTAGTGTGCATGCTTCTCAGTTGGACAAGGACTATAAGCCAGGGAATTTCAAATAA

>ColMADS42

ATGGGTAAAGCTAAACAAAACATGGAACTCATACGCAACGACAAGACTCGATGTGTGACCTTTCAAAAGAGAAACAAGGGATTGAAGAAGAAGACTTATGAATTAAAAACCCTCTGTGATGTTGAAGTTTGTTTGATTATTTATGGTCCTAAGATCAATGACCGTCCTACGGATGATCAAGCAGAAATCTGGCCCCCAAATCCCAATGTAATTCAGCACCTGATCAACATCTACAAAAACCAATCTAGTGAAGATCGCATGAGGAGAACCCTTGGTTTGTCCAACTTTTTTGAGGATAGGAATCAAAAGATTGAAGACATGCTTGTGAAACTACGAAAGAAAAATGATGAGGCTAGGTACTCTACTTGGGATGATCGGTACAATGGTTTGTCGAAAGAACAATTGAGAGAATTTGAAAGTGTTTTGGAGGGAAAGCTTCAAGATGTGAAGGCTAGGGTTGCATTCATGAAGGGTATTGATCAGAAACTATCAGTAGCATGTATCAACGACAATTCTCACACTCAAACCACCCAAAACCAATCTTATTTCATGCATGGAGGCATGTTTGGGACAGAAGTTGACAACGACATCGCTAATTTACAGTACTCTTTCAATCAGGCTCAAGTTGATGCAAATTCCAAGGCGAATCCAACGATGATGATGACGATGATGCCAAGGAACAATAATCAGTATTACAATCAACTTGGCGGCATGTCTAGCAGTAACTTTATGTGCAATCCACTAGATTGGCCAATTTATTATGATCCGATGATGTTGGAGAATATGATTGTTAATAATCCTTCGCTGCCTGTGGGCTACTACAATCCGAGCATACACACAACGTCGTCGTCATCTAGTGCGCATGCTTCTTAG

>ColMADS43

ATGGGAAATGGAAAAAAAAAAATTGAGATTAAGAGAGTGGAGAAGGAAGGGCAGAGAATGGTTACATTCTCCAAGAGGCGTCGGGGTCTCTTCAACAAAGCCCGACAACTCCGATCCCTCACCGGCGCCGACATCGCAATCCTCACCTTCTCCCCCGCTGGCCGTCCCTTCACTCACGGCGAGCCCTCCTTCGATGCCCTCGTCGACCGTTACCTCAACACCGCCGCCGCCGGAGAGAAGGCAGAAGAGGGTTGTGAGGCTGCCGCCGCGAACCACCACCGGTTGAGTTCGTGGTTGGATGCTCTGCAATTCGATGCGAGTGATAGCATTGAAGATTTGGAAATCTTGAAGAAAGGGCTGGAAGAGATCGCAGTGAAAGTGGCTGAGAAAATCGATGACATTTTTGTTGATTCTTTGCTCGTTTGA

>ColMADS44

ATGGGAAATGGAAAAAAAAAAATTGAGATTAAGAGAGTGGAGAAGGAAGGGCAGAGAATGGTTACATTCTCCAAGAGGCGTCGGGGTCTCTTCAACAAAGCCCGACAACTCCGATCCCTCACCGGCGCCGACATCGCAATCCTCACCTTCTCCCCCGCTGGCCGTCCCTTCACTCACGGCGAGCCCTCCTTCGACGCCCTCGTCGACCGTTACCTCAACACCGCCGCCGCCGGAGAGAAGGCAGAAGAGGGTTGTGAGGCTGCCGCCGCGAACCACCACCGGTTGAGTTCGTGGTTGGATGCTCTGCAATTCGATGCGAGTGATAGCATTGAAGATTTGGAAATCTTGAAGAAAGGGCTGGAAGAGATCGCAGTGAAAGTGGCTGAGAAAATCGATGACATTTTTGTTGATTCTTTGCTCGTTTGA

>ColMADS45

ATGGGAAGTGGAAAAAGGAAAATTGAGATTAAAAGAGTGGAGAAGGAAGGACAGAGAATGGTTACATTCTCCAAGAGATGTCAGGGTCTCTTCAACAAAGCCCGACAACTCCGATCCCTCACCGGCGCCGACATCGCAATCCTCACCTTCTCCCCCGCTGGCCGTCCCTACACTCACGGCGAGCCCTTCTTCGACGCCCTTGTCGACCGTTACCTCAACACCGCCGTCGCCGGAGAGAAGGCAGAAGAGGGTTGTGAGGCTGCCGCCGCGAACCACCATCAGTTGAGTTCGTGGTTGGATGCTCTGCAATTCAATGCGAGTGATAGCATTGAAGATTTGGAAATCTTGAAGAAAGGGCTTGAAGAGATCGCAACGAAAGTGGCTTTGAAAATCGATGACGTTTTTGTTGATTCTTTGCTCGTTTGA

>ColMADS46

ATGGCTACTCCTAATCGTTGTATTGCTACTCCTAAAATATCACGATTTGTGAATGAGGTTGAAGGTCTATTTTTCTTTGTAGTCCCAACTTTTATACTTGTTGGTGTTGAACGGTGGGAAGAGAAGAGTTCTTCAATAGTTTTAGCCATGGCAAAGAAACCAAGCATGGGTCGCCAAAAGATCAAAATTGCGAAAATAGAGATCAAAAATCGCTTGCAAGTCACCTTCTCAAAACGCCGATCTGGGCTTTTCAAGATGGCAAGTGAACTTTGCACGCTTTGTGGTATAGAGATGGCTATCATAGTTTTCTCTCCAACTGGTAAAGTCTTTTCTTTCGGCCACCCTAACGTGGATTCTATAGTTGATCAATTCCTGACTCGAAACCCTCCACCTGACACCACCGCACGCCACCTTATTGAGGCTCATCGTGATGCTAGTGTGCACGAGCTCAACTTACAACTTACTCAAGTTCTCAATGAATTGGAAGTCAAGAAAAGACATGGTGAAACCTTTGATCACACGAGAAAAGCTAGTGAAAGACAATATTGGTGGGAAGCTCCAATAGACAAGCTTGGCTTGCATGAGCTTGAACAACTTAGGAATTCAATGGTGGAGCTCAAGAAGAACGTAACCAATCAGGCAAACAAAATTCAAGTCGAGGGCAACACAAATCCTTTGCCATTTTTCTTGGTAAATGGCAGTGGAATCGTTGATCCCTTTGAGAGTAAAACATCTCATATCAATGCTTCTTCTTCAACCGGTCCTAATCTTTCACAGCAACCAAACGAACCCTAA

>ColMADS47

ATGGAGGATGATGAAAATGGTAAGAAACCCATGAAGCGAATTGACAACAAGAAAGCTTTGAGAGTTACTTACATAAAGAGAAAGGACTGTGTTATCAAGAAGACAATGGAGCTCTCGATTCTCTGTGGTATCAATGTCTTCACACTCTGTTTCGGCCCCAATGGAGAAGTCGATACGTGGCCCCAAAACCCAAACGAAGTGAAAGCTCTGATCAAGATGTACAAAGAGTGCCAAGAAAAGCCCGTTGCCAAGAAAGTTTTGGTGGGGAATGAGGATGGTTATGGTTGGCTTGATGGGTTATCGGCAGAGTCGGCGATGAGTTTCTTGGGAAAGTTGGAGTCAAAATTGGAGGTTTTGAAAGCAAGAAACGAGTTCTTGAAGATGGTGAAGGAGCAGAAAAACAGAGGGGTAGTGGTAATGGGGAAAGAAACAGAGAAAACTTCTTGTTGGTTTGATGATACGAATGCGATCGACAATTTGCAGAAAAACAGAGGAGTAATCGTAATGGGGAAAGAAACAGAGGAAGCTTGTTGGTTTGATGATACAAGTGCGATCGGCAATTTTGAGGAGGACGATCTTGCAATGTTGCTTGAGCCT

>ColMADS48

ATGGGGAGGGTTAAGTTAAAGATAAAGAGATTAGAGAGCACTAGCAATCAACAAGTGACCTATTTGAGAAGAAGGAATGGAATCTTCAAGAAAGCTAAAGAATTATTCATATTATGTGACATTCACATTGTCCTTCTCATGTTTTCTCCAACTGGAAAGGCAACATTATTCCGCAGAGAGCGCAGCAACATTGCAGAGGTTATTACAAAATTCGCTCAACTGACACCACAATAA>ColMADS49ATGTTCAAGAAAATCGACGACCTCTCAACACTATGCGACGTCAAAGCCGGCACCGTCATTTACAACCTAGGAGAGGCTGAACCGATGGTGTGGCCATCATACGAGTACATGAAACAAATGTTTGAGAAGTTCTTGAGCATGTCAATAATAGACAGAAGTCAAAAGATGGTGACATATGAGCGCTACCTCATACAAATGATCACCAAGGAGAGTGAGAAAAATAATAGAGAAAAGAAAAATAATGATAAAAAAGAAATTCAAGAAATCATGAATCAAATGTTTGAAGGAAATGATCTAAATGAACTTAATATGATGAAGTTGAATTTATTGTCTTTGTTGGCAGATGATAAGTTGAAGCAACTAAAGAATAGGCAAAGTAGGCCCCGTGGTCAACAGATGGCTCTTTTTCCTCCCCCGCCTCCTCTACCTTTGTCGCCAGTTCCAGCTCCAAAAATCATAGAAGTAGAAGAACCAGTTCCAGCTCCTGAAATGATTGAAGTGGAAGGACCGATTCCAGCTCCTGAAATGATAGAAGTGGAAAGACAAGTTCCAGCTCCTGAAAGGATAGAAGTGGAAAGAGTAGTTGAAGATGGAGGAAGCTTGATTCCGATGATAATTCAGGAGTTGATGAACGACCAGTGTTTCATGGAAACAACGGCGGAACGTATGGAGCAGTTACTATCAGAGGATGTTGATGGAACAAGTTACAGTGAAAATAAAGAAGACCTGTGGGAGGACTTAGATGAAGTGTTCCATCATATTTACTTCCCATGA

>ColMADS50

GGTGAAAGAGAAAGAAAGAGAGAGAAGATGCGTAGAGGCAAAGTAGAGTTAAAGAGGATTGAGAACCCATCGAGCAGGCAAGTGACCTTTTCTAAGAGGAGAAATGGGCTTCTAAAGAAGGCTTTTGAGCTGTCCATCCTCTGTGATGCTGAGGTTGCCCTTCTCATGTTCTCTCCCTCTGGAAAGGCTTATCAGTTCTGCAGTCATGACATGGATAGGACCATTTCAAGGTACCGGAGTGAAGTGCGAATTTCTCAATTTAATGACCAAGGACTAAGAACCATGGAGGTTCGGAGGAGTGAAATGGATGAATTAAAGAGCACAATAGAAACCTTGGAAGCAAGACAAAAGCACTTTGCCGGAGAAGATCTATCAATGTTGAGCATGAAAGAATTGAAGCTAATAGAGCGCCAGTTGAGGAATGGAGTTGAACGCGTCCGCTCTAAAACG

>ColMADS51

ATGCGTAGAGGCAAAGTAGAGTTAAAGAGGATTGAGAACCCATCGAGCAGGCAAGTGACCTTTTCTAAGAGGAGAAATGGGCTTCTAAAGAAGGCTTTTGAGCTGTCCATCCTCTGTGATGCTGAGGTTGCCCTTCTCATGTTCTCTCCCTCTGGAAAGGCTTATCAGTTCTGCAGTCATGACATGGATAGGACCATTTCAAGGTACCGGAGTGAAGTGCGAATTTCTCAATTTAATGACCAAGGACTAAGAACCATGGAGGTTCGGAGGAGTGAAATGGATGAATTAAAGAGCACAATAGAAACCTTGGAAGCAAGACAAAAGCAGTTTGCCGGAGAAGATCTATCAATGTTGAGCATGAAAGAATTGAAGCTAATAGAGCGCCAGTTGAGGAATGGAGTTGAACGCGTCCGCTCTAAAACG

>ColMADS52

ATGAAGGACGATGAAAAGGGTAAGAAACCCATGAAGCGAATTGAGAACAAGAAAGCTCTGAGAGTTACTTACATGAAGAGAAAGGACTGTGTTATCAAGAAAACAATGGAGCTCTCGATTCTCTGCGGTATCAATGTCTTCACACTCTGTTTCGGCCCTAATGGAGAGGTCGACACGTGGCCTCAAAACCCAAACGAAGTGAAAGCTCTGATCAAGATGTACAAAGAGTCTGCCAAGAAAAGCCCGGTTAGGAAAACTTGTGAATCTTCATTTTCTGATTGTTGTTTGGATGCTGAGAAAGTTGCCAAGAAAGTTTGGGTGGGGAATGAGGATGGTTATGGTTGGCTTGATGGGTTATCGGCAGAGTCGGCGATGAGTTTCTTGGGAAAGTTGGAGTCAAAATTGGAGGTTTTGAAAGCAAGAATCGAGTTCTTGAAGATGGTGAAGGAGCAGAAAAACAGAGGGGCAGTGGTAATGGGGAAAGAAACAGAGGAAACTTCTTGTTGGTTTGATGATACGAATGTGATCGACAATTTGCAGAAAAACAGAGGAGTAATCGTAATGGGGAAAGAAACAGAGGAAGCTTGTTGGTTTGATGATACGAGTGCGATCGACAATTTTGAGGGGGACGACCTTGCAATGTTGCTAGACCTTTATCACCAGTACCAACCTGGCATTTTCTGA

>ColMADS53

ATGGAGGATGATGAAAAGGGTAAGAAAACCATGAAGCGAATTGAGAACAAGAAAGCTCTGAGAGTTACTTACAAGAAGAGAAAGGACTGTGTTATCAAGAAGACAATGGAGCTCTCGATTCTCTGCGGTATCAATGTCTTCACACTCTGTTTCGGCCCCAATGGAGAGGTCAACACGTGGCCCCAAAACCCAAACGAAGTGAAGGCTCTGATCAGGATGTACAAAGAGTCTGCCAAGAAAAGCCCGGTTAGTGAAACTTGTGAATCTTCTTTTTCTGATTGCTGTTTGGGTGCTGAGAAAGTTGCCAAGAAAGTTTTGGGGGGGAATGAGGATGGTTATGGTTGGCTTGATGGGTTATCAGCAGAGTCGGTGATGAGTTTCTTGGGAAAGTTGGATTCAAAATTGGAGGTTTTGAAAGCAAGAATTGAGTTCTTGAAGATGGTGAATGAGCAGAAAAACAGAGTAGTGGTAATGGGAAAAGAAACAGAGGAAACTTGTTGGTTTGATGATACAAATGCGATCGACAATTTGCAGAAAAACAGAGGAGTAATCGTAATGGGGAAACAAACAGAGCCTTGTTACCAACCACAACCACAACCACAACCACAACAACAACAACAAGCTATTCAACTGAATTTTGAGATGACCAATGCTTCAATGTTGATGGAGCCTTGTTACCAACCACAACAACAGCAACAAGATACTCAACTGAATTTTGTGATGGACAACCTTTCAATGTTGCTTGACCTTTGTCACCAGTACCAATCTGGCATTTTCTGA

>ColMADS54

ATGGAGGATGATAAAAAGGGAAAGAAACCCATGAAGCGAATTGAGAACAAGAAAGCTCTGAGAGTTACTTACATGAAGAGAAAGGACTGTGTTATCAAGAAGACAATGGAGCTCTCGATTCTCTGCGGTATCAATGTCTTTACACTCTGTTTCGGCCCCAATGGAGAGGTCGACACGTGGCCCCAAAACCCAAACGAAGTGAAGGCTCTGATCAGGATGTACAAAGAGTCTGCCAAGGAAAGCCCGGTTAGTAAAACTTGTGAATCTTCCTTTTCTGATTGTTGTTTGGGTGCTGAGAAAGTTGCCAAGAAAGTTTTGGTGGGGAATGAGGATGGTTATGGTTGGCTTGATGGGTTATCAGCAGAGTCGGTGCTGAGTTTCTTGGGAAAGTTGGATTCAAAATTGGAGGTTTTGAAAGCAAGAATTGAGTTCTTGAAGATGGTGAATGAGCAGAAAAACAGAGGAGTAATCGTAATGGGGAAACAAACAGAGGAAGCTTGTTGGTTTGATGATACAAGTGCGATAGACAATTTTAAGGAGGACTGCCTTGCAAGAAAGGACTGTGTTATCAAGAAGACAATGGCGCTCTCGATTCTCTGCGGGATCAATGTCTTCACACTCTGTTTCGGCCCTAATGGAGAGGTCGACACATGGCCCCACAACCTAAATGAAGTGAAAGCTCTAATCAAGATGTACAAAGAGTCTACTGAGAAAAGCCCGATTAGGAAAACTTGTGAATCTTTTAAAGTTGCTAAGAAAGTTTTGTTGGGAAATGAGGATGGTTATGGTTGGCTTGATGGGTTATCGACAGAGTCGGTGATGAGTTTCTTGGAAAAGTTGGAGTCAAAATTGGAAGTTTTGAAAGCAAGAATTGAGTTCTTGAATATGTGCGGAAAGAAACATAAGAAGCTTGTTGGTTTAATGATACGAATGCGATTGACAATTTGCAAAAAAAACAGAGAAGTAATGGTAATGGGGAAAGAAACAGAGGAAGCTTGTTGGCTTGATGATACGAATGCAATCAACAATTTTGAGGACTATCTTGCAATGTTGCTTGAGCCTTATTACCAGCCACAGCCACAACAACAAACAAGTCAATTGGATTTTGAGATGAACAATTTTTGA

>ColMADS55

ATGAAGGACGATGAAAAGGGTAAGAAACCCATGAAGCGAATTGAGAACAAGAAAGCTCTGAGAGTTACTTACAAGAAGAGAAAGGACTGTGTTATCAAGAAGACAATGGAGCTCTCGATTCTCTGCGGTATCAATGTCTTCACACTCTGTTTCGGCCCTAATGGAGAGGTCGACACGTGGCCTCAAAACCCAAACGAAGTGAAAGCTCTGATCAAGATGTACAAAGAGTCTGCCAAGAAAAGCCCGGTTAGGAAAACTTGTGAATCTTCTTTTTCTGATTGTTGTTTGGATGCTGAGAAAGTTGCCAAGAAAGTTTGGGTGGGGAATGAGGATGGTTATGGTTGGCTTGATGGGTTATCGGCAGAGTCGGCGATGAGTTTCTTGGGAAAGTTGGAGTCAAAATTGGAGGTTTTGAAAGCAAGAATCGAGTTCTTGAAGATGGTGAAGGAGCAGAAAAACAGAGGGGCAGTGGTAATGGGGAAAGAAACAGAGGAAACTTCTTGTTGGTTTGGTGATACGAATGTGATCGACAATTTGCAGAAAAACAGAGGAGTAATCGTAATGGGGAAAGAAACAGAGGAAGCTTGTTGGTTTGATGATACGAGTGCGATCGACAATTTTGAGGGGGACGACCTTGCAATGTTGCTAGACCTTTGTCACCAGTACCAAACTGGCATTTTCTGA

>ColMADS56

ATGAAGGACGATGAAAAGGGTAAGAAACCCATGAAGCGAATTGAGAACAAGAAAGCTCTGAGAGTTACTTACATGAAGAGAAAGGACTGTGTTATCAAGAAGACAATGGAGCTCTCGATTCTCTGCGGTATCAATGTCTTCACACTCTGTTTCGGCCCTAATGGAGAGGTCGACACGTGGCCTCAAAACCCAAACGAAGTGAAAGCTCTGATCAAGATGTACAAAGAGTCTGCCAAGAAAAGCCCGGTTAGGAAAACTTGTGAATCTTCTTTTTCTGATTGTTGTTTGGATGCTGAGAAAGTTGCCAAGAAAGTTTGGGTGGGGAATGAGGATGGTTATGGTTGGCTTGATGGGTTATCGGCAGAGTCGGCGATGAGTTTCTTGGGAAAGTTGGAGTCAAAATTGGAGGTTTTGAAAGCAAGAATCGAGTTCTTGAAGATGGTGAAGGAGCAGAAAAACAGAGGGGCAGTGGTAATGGGGAAAGAAACAGAGGAAACTTCTTGTTGGTTTGGTGATACGAATGTGATCGACAATTTGCAGAAAAACAGAGGAGTAATCGTAATGGGGAAAGAAACAGAGGAAGCTTGTTGGTTTGATGATACGAGTGCGATCGACAATTTTGAGGGGGACGACCTTGCAATGTTGCTAGACCTTTGTCACCAGTACCAAACTGGCATTTTCTGA

>ColMADS57

ATGGGGCGTGGAAAGATAGAGATAAAGAGGATAGAGAATGCAACGAACAGGCAGGTGACTTACTCGAAGAGAAGGAATGGTATAATGAAGAAAGCACAAGAGCTCACAGTTCTGTGCGATGCCAAGGTCTCCCTCATCATGTTCTCCAACACCGGCAAATTTCATGAGTACACCACTCCCAACATCACGACGAAAAAGATCTACGATCAGTACCAGAAGACTCTTGGGATCGACCTATGGAGCACACATTACGAGAGAATGCAAGGACACTTGAAAAAACTCAAGGAGATCAACAATAAACTAAGGAGAGAGATCGGGCAGAGAGTGGGTGGAGAGGACTTGAACGATCTAAGCATCCAGGAACTGTGCGGTCTTGAGCAAAAGATGGCTGCTTCTTTGACTGATGTTCGCCAACGGAAGTATCATGTGCTCAAAACTCAGACAGAGACCTACAAGAAGAAGGTGAGAAGCATGGAGGAAAGGCATGGAAATCTCCTACTGAACTTTGAGGCAAGATGCGAAGATCCTCAGTTTGGAACATTATTTGAGAATGACCGCGACTATGACTATGCTTTCTGCCTTCACCGTGGAGGAGGTTTTGGATCCAATGAACTGCGGCTTGCTTAA

>ColMADS58

ATGGGGCGTGGAAAGATAGAGATAAAGAGGATAGAGAATGCAACGAACAGGCAGGTGACTTACTCGAAGAGAAGGAATGGTATAATGAAGAAAGCACAAGAGCTCACAGTTCTGTGCGATGCCAAGGTCTCCCTCATCATGTTCTCCAACACCGGCAAATTTCATGAGTACACCACTCCCAACATCACGACGAAAAAGATCTACGATCAGTACCAGAAGACTCTTGGGATCGACCTATGGAGCACACACTACGAGAGAATGCAAGGACACCTAAAAAAACTCAAGGAGATCAACAATAAACTAAGGAGAGAGATCGGGCAGAGAGTGGGTGGAGAGGACTTGAACGATCTAAGCATCCAGGAACTGTGCGGTCTTGAGCAAAAGATGGCTGCTTCTTTGACTGATGTTCGCCAACGGAAGTATCATGTGCTCAAAACTCAGACAGAGACCTACAAGAAGAAGGTGAGAAGCATGGAGGAAAGGCATGGAAATCTCCTACTGAACTTTGAGGCAAGATGCGAAGATCCTCAGTTTGGAACATTATTTGAGAATGACCGCGACTATGACTATGCTTTCTGCCTTCACCGTGGAGGAGGTTTTGGATCCAATGAACTGCGCCTTGCTTAA

>ColMADS59

ATGGCGAGGAAGATGGCCGGAGGTCGCAGAAAGATTGAAATGAAGTTGATTCCTTCAAAATCTGCTCGCCAAGTTGCCTTATCAAAGCGCCGATTAGGCATTTTCAAGAAGGCAAATGAGCTATGTATCTTGACTGGTTGTGAGATTGGTATTGTTGTCTTCTCACCTAGTGGGAAAGCCTTCTCATTTGGCCATCCTTCTGTTGACACAATAGTGCAGAGGTCCCTTTACGAAAGCCCCATGCCCGCTGCTATAGGTCAGGATTCTCATGGATCTATTGTTCCTGCACTCAGCCAAGAGTACACTAAGATGTGTAGACAACTAGGAGCAGAGAAGAGGCGAGGAAAGGAGCTTAAGGAGAGTGCGATGAAGTGCCAAAGGCCCTACTGGTTGGATGCCCCAATTCATGAGCTCAACTTGGATCAAGTACTGGATTCCAAGAAATGTATGGAAGAACTTAGGGCTAAAATAGCCAAGAGAGTCAATGAACTTTCAGTTGAGGGATCTGCTTCAAATTTAGCTTCTTCTGCAAAATTTGTTCGAGGCATTGATCTCAATATGGCCAGAGGTTGCAGAAAGATTGAAATGAAGTTGATTCCTTCAAAATCTGCTCGCCAAGTTGCCTTTTCAAAGCGCCGATTAGGCATTTTCAAGAAGGCAAATGAGCTATGTATCTTGACGGGTTGTGAGATTGGTATTGTTGTCTTCTCACCTAGTGGGAACGCCTTCTCATTTGGCCATCCTTCTGTTGACACAATAGTACAGAGGTCCCTTTACGAAAGCCCCATGCCCGCTGCTATAGATCAGGATTCTCATGGATCTATTGTTCCTGCACTCTGCCAAGAGTACACTGAGATGTGTAGACAACTAGGAGTAGAGAAGAGGCGAGGAAAGGAACTTAAGGAGAGTGCGATGAAGTGCCAAAGGCCCTACTGGTTGGATGCCCCAATTCATGAGCTCAACTTGGATCAAGTACTGGATTCCAAGAAATGTATGGAAGAACTTAGGGCTAAAATAGCCAAGAGAGTCCATGAACTTTCAGTTGAGGGATCTGCTTCAAATTTAGCTTCTTCTGCAAAACTTGTTCGAGACATTGATCTCAATGGTCCTTTGGGCCCTCCCCAAGAGCGTGAGCCCGAGAATCAAGTGACTCCCCCTAATCGCCTGATCCGGGCAGGGATTCTATCTTATGTTTTGCAAGGTGGGCTTCAAACTTTTTTTTGTACTGATACATTGAGGCCGATTGTCCCCCCGGGAAGGCTACTCTCGCCTTGCAAACTGCTTCTCGAATGCTCTGATGAATGA

>ColMADS60

ATGGGTAGGGGGAAAATTGAGATAAAGAAGATAGAGAATGTTAATAGCCGGCAAGTCACCTTCTCTAAGCGAAAAGCCGGTCTGCTCAAGAAGGCCAAGGAGTTGTCAATTTTATGTGATGCCGAGGTTGGTCTTATAATCTTCAACAACTCAGGCAAGCTCTATGAATTCGCCAGTCCCCGCATGGAACACATTCTTGCAAGATACAACAATGGCCTTGAATCTTCTACACTTACAAATCCTTCAACTGAGAAAGCAGTATTAGAGCTTGAAAAGCAGCCCCCCGAGCTAGATGCTTTAAGAGGTGAAGTTGCAAAACTACAAAAGGGGTATAGACGAAAAATGGGTAAGGATTTGGAGGGAATGAGTTTCAAGGAACTTCAACAATTGGAGCATCAATTGAATGAAGGGATATTGTCCGTCAAGGATAGAAAGGAACAAGTATTGTTGGAGCAGCTTGAGAAATCAAAATTGCAGGAGCAAAAGGTCAAGCTAGAGAATGAAACTCTGCGCGAGCAGATTGAGGAGCTTGGGCGACCAAGCACAACACCTACGCCTACTCATGAATTCCATTGTCTTGGAAGAAAGCCATCTGTTCTAAGTTCAAATATAGTTTGTGATGGTTACTTTGACACAGAAAGAGACTCAGAGACTTCTTTGCGCTTGGGGTTGTCAAGTGAAGTTTTTCATAAGAGAAAAGTATGCAAGATTGAATCGGTTTCTAATGATGACTCTGGCAGCCAAATGGCTTCA

>ColMADS61

ATGACTATTATACCCACTCTCTTCCCTTCCACAGATGGCCTCTTCAAGAAGACCAGCGAGCTCTGCAACTTGTGCGGCACCGAAGCCGCTATCATCACCTTCTCCAACGCCGGCAACACCTTCGCCTTCGGCAACCCCTCCGTCGACTCCGTCATCAACCGTTACCTCTCCATGACTTCATCATCCTCCAAAGCCTCCGCAAGCTTCTCTTAG

>ColMADS62

AATGATGATGATGAAGGATTCGAGTTCGGTGTGGAGATCAAGGTCTGTAAAGGATACGACGACGGGTTTGTTGTCTTCTTTGTTTTCGAGATCATCTTCTTGAGCAAAACTCCTTCGAGAGAGCCAAATCGAAGAGAGATAGTCCAGTTCTTGTCAAATCCAACTGCCCAGAAGAAATATCCACCGAGGCCATTCGCTGGAGCAAAGTTCACCTTGTTGCTGATCGATTCCGTGTCATCATACCCAATCCAATCGGTTCCTGCATATGAGTATGTGGACACGGTCTCCTCATCAAACACAACAGAGGCATTCCCCAAGCCAACAAAGCAACGAAGCAGCAGCACACAAACAGAGCAGAGCACGCGGACGGAGCAGCAGCAAGCACAGATGGGTCGAGCTAAACAAAGAATGGAACTCATAAGAAACGAAAAGGCTCGCTATGTAACCTATCAAAAGAGAAAGAAGGGCTTGAAGAAGAAGACTTGTGAACTAGAAACTCTATGTGATGTCCAAGTCTGCCTGATCATCTATGGACCTAAGTTAGATGATAATTCCACAGAGGTAGAAATCTGGCCCCAAAATCCCAATGATATTCAACGCTTGATTCACACCTATAGAAACCAATCTATTGAAGATCGTCATAGGAGGACGCTTGATTTATCCAACTTTTTTGAGAACCGAAACCAAGAGATTGAAGACGCACTTGTCAAACTACGTAAGAAGAATGACAAGGCTTTGTACTCTACGTGGGATGATCGTTACAATGACTTGTCGGAGGGGCAGCTGAGAGCCTTTGAAGGTATGTTGGAGGGTAAGCTTGCGGATGTGAAGGCCAGGGTTGAATTCATGAAGGGAACTCGAGCATTGGCAAGTCTTAATGATATGGAACAAACCACTCAACAAAACCATTCAAATTATTTCATGCAAGGCTTGTTTGGTACTACAAGTTTGCAAATGGGGATTATCCATGAGAAGATGCCTATTTCTTCGTTGAATTCGCTGTATGAAATTGATGGCATCCCTCTTCATTACCCTTTTGATCAGACTGATCACCAGGGAATTGTTGAGCTTGATGCAAAATCCATGGCAATTAGTCCAATGATGATGCCAATGAACAACATTAATGACACTCATTACACTCAACTTGGCGGTATGTCTAGCAGCAACTTGCAGTGTATTGATCCACTAGAGAAGTCGATATATTATGATCCAATAATGTTGTTGCCCTCTCCAGAGTCTGTTTTAACAGGAGTCTCGTGCACTGAGTCGTCCTTTTCAATGTCTAGGATGATGGAGAACGAGGTTGGTAAAAATCATCATCCTTCACCCTCTATGGCTTACTATGGCGGTTCAAGCATGCAGACAATGTCACCATATATGCAGTACCTGAAGATGGCAAGTGGTCCTTCTCAAGTTCATGCATGGCAGATGGAGGAGTACTATAAAGCCAATGAGTTTCAAATGAAGAACCAGAAG

>ColMADS63

ATGGGGCGAAAGAAGGTGGAAATGAAGCGAATCGAATCGAAGAGTAGTCGACAAGTGACATTCTCGAAGAGGAGAGGTGGATTGGTCAAGAAAGCTCGCGACCTCTCCGTCCTCTGCGACGTCGACGTCGCTCTCCTCGTCTTCTCCAGCCGCGGCAAGCTCTATCAATTCTGCAGCGCCAACAACAGGTCTCTCTCTCTCTTCGATTTTTGTTTGTTTTCATGTTTTTGGAATCACTGTTTTGCGTGA

>ColMADS64

ATGGTGAGAGGGAAGACTCAGATGAGGAGGATAGAGAACGCTACAAGCAGGCAAGTCACCTTTTCCAAAAGGAGAAATGGGCTTCTGAAGAAGGCATTTGAGCTCTCGGTTCTCTGCGATGCTGAGGTTGCTCTCATCATCTTCTCTCCTAGGGGAAAGCTCTATGAATTTGCTAGTCCAAGATTGAAATTGTTTAGCTAA

>ColMADS65

ATGGAAAGGAAGAGCAAAGGCCGTCAGAAGATCGCGATGACAAGAATCTCAAATGCAAGCAACCGAAACGTTACCTTCTCAAAGCGTCGTTCTGGGCTATTCAAGAAAGCAAGCGAACTCTGCACCCTATGTGGTGCTGAAATTGCCATCATCGTTTTCTCTCCTGGCAAAAAGGCCTTCTCTTTTGGCCACCCTTGTGTTGACACTGTTGTAGATTGTTTTCTCTCTCGAAACTCTCTACCCAATTCAGGCTCGCTCCAACTTGTCGAGGCTCACTGCAATGCGAATGTTAGCAAGCTCAACTTGCAACTTAATGAAGCACTTGATATGTTGGAAGCAGAGAAGAAAAGAGGTGAAGAGCTTAACAAGATGAGGAAAGCTAGCCGAGACAGGTGTTGGTGGGAAGCACCGGTTAGCGAGCTTGGACTTCAGCAACTCGAGCAATTGAAGGTTGCAATGGAGGATCTAAAGAAGAATGTTGCGAAGCAAAGCGAGAAGATTCAGATGGAGGAATCGAACCCCTCGAGGTTTTTTGAAGCTGGGTCAAGTTCCATGGGAGGGCGTGTTGGAGGTGCTTCTGATGGTGTTAAGGTTTCAGGGCTACGTCTGTCCATGACTCCATATGGATATACCCTCGACTACGGACATGGATTTTTTTGA

>ColMADS66

ATGGGTAGAGCTAAACAAAACATGGAACTCATACAGAATGACAAGACTCGATATGTGGCCTTTCAAAAGAGAAAGAAGGGGTTGAAAAAGAAGACTTACGAATTAAAAACCCTCTGTGATGTTGAAGTGTGCTTGATTATTTATGGTCCTAAGATCAATGACCGTCCTACGGATGATCAAGCAGAAATCTGGCCCCCAAATCCCGACGTAATTCAGCACTTGATCGACACCTACAAAAACCAATCTAGTGAAGATCGCACGAGAAGAACCCTTGGTTTGTCTGACTTTTTTGAGGATAGGAATCAAAAGATTGAAGACACGCTTGTGAAACTACGAAAGAAAAATGATGAGGCTAGGTACTCTACGTGGGATGATCGGTACAATGATTTGTCAAAAGAACAACTCAGAGAATTTGAAAGTGTTTTGGAGGGAAAGCTTCAAGATGTGAAGGCTAGGGTTGCATTCATGAAGGGTATTGATCAGAAACTGTCAGTAGCATGTATGAACGACAATTCTCACACTCAAACCCAAAACCAATCTTATTTCATGCATGGATGCATGTTTGGGACTGAAGTTGACAACGGCTTCGCTAATTTACAGTACTCTTTCAATCAGGCTCAGGTTGATGCAAATTCCAAGGCGAATCCAATGATGATGATGACGATGATGCCAATGAACAATAATCAGTATTACAATCAACTTGGCGGCGTGTCTAGTAGTAACTTTATGTGCAACCCACTAGATAGGCCAATTTATTATGATCCGATGATGTTGGAGAATGTGATTGTTAACAATCCTTCACAGCCTGTAGGCTACTGCAATCCGAGCATACAAACAATGTCGCCGTATATGCAGTGTGTGAAGATGGAATGTGCTTCATCTAGTGTGCATGCTTCTCAGTTGGACAAGTAA

>ColMADS67

ATGACTATTAAACCCACTCTCTTCCCCACTCTCTTCCTTTCCACAGATGGCCTCTTCAAGAAGACCGGCGAGCTCTGCAACTTGTGCGGCACCGAAGCCGCCGTTATCACCTTCTCCAACGCCGGCAACACCTTCGCCTTCAACCCCTCCGTCGACTCCGTCCTCGACCGTTACCTCTCCATGACTTCATCATCCTCCAGAGCCTCCGCAAGCTTCTCTTTCCTCTTTCAACATAAGGTTTCAACTCAAATCTGTGCACTATGA

>ColMADS68

ATGGGAAGCGGAAGAAGAAAAATTGAGATTAAAAGGGTAGTGAAGGAAGGGCAGAGAATCGTTACATTTTCCAAGAGACGTCGTGGTCTCTTCAACAAAGCCCGACAACTCCGATGCCTCACCGGCGCCGACATTGCAATCCTCACCTTCTCCCCCGCTGGCCGTCCATACACTCACGGCGAACCCTCCTTCGACGCCCTTGTCGACCGTTACCTCAACACCGCCGCCGCCAGAGAGAAGGCAGAAGACGGTTGTGAGGCTGCCGCCGCGAACCACCACCGGTTGAATTCGTGGTTGGATGCTCTGCAATTCGATGCGAGTGATAGCATTGAAGATTTGGAAATCTTGAAGAAAGGGCTTGAAGAGATCGCAGCGAAAGTGGCTGAGAAAATTGATGACGTTTTTGTTGATTCTTTGCTCGTTTGA

>ColMADS69

ATGGGAAATGAAAAAAAGAAAATTGAGACTAAAAGAGTGGAGAAGGAAGGGCAGAGAATGGTTACATTCTCCAAGAGACGTCATGGTCTCTTCAACAAAGCCCGACAACTCCGATCCCTCACCGGCGCCGACATCGCAATCCTCACCTTCTCCCCCGCTGGCCGTCCCTACACTTACGGCGAGCCCTCCTTCGACGCCCTTGTCGACCGTTACCTCAACACCGCCGCCGCTGGAGAAAAGGCAGAAGAGGGTTGTGAGGCTGCCGCCGTGAACCACCACCGGTTGAGTTCGTGGTTGGATGCTCTGCAATTCGATGCGAGTGATAGCATTGAAGATTTGGAAATCTTGAAGAAAGGGCTTGAAGAGATTGCAGCGAAAGTGGCTAAGAAAATCGATGACGTTTTTGTTGATTCTTTGCTCGTTTGA

>ColMADS70

ATGGGAAGCGGAAGAAGAAAAATTGAGATTAAAAGGGTAGAGAAGGAAGGGCAGAGAATGGTTACATTCTCCAAGAGACGTCGTGGTCTCTTCAACAAAGCCCGACAACTCCGATGCCTCACCGGCGCCGACATTGCAATCCTCACCTTCTCCCCCGCTGGCCGTCCATACACTCACGGCGAACCCTCCTTCGACGCCCTTGTCGACCGTTACCTCAACACCGCCGCCGCCGGAGAGAAGGCAGAAGAGGGTTGTGAGGCTGCCGCCGCGAACCAGCACCGGTTGAATTCGTGGTTGGATGCTCTGCAATTCGATGCGAGTGATAGCATTGAAGATTTGGAAATCTTGAAGAAAGGGCTTGAAGAGATCGCAGCGAAAGTGGCTGAGAAAATTGATGACGTTTTCGTTGATTCTTTGCTCGTTTGA

>ColMADS71

AGTGAAACATTCAGCAAAGCCCGACAACTCCGATCCCTTACAGGCGCCGACATCGCAATCCTCACCTTCTCCCCCGCTGGCCGTCCCTACACTCACGGCGAACCCTCCTTCGACGCCCTTGTCGACCGTTACCTCAACACCGCCGCCGCCGGAGAGAAGGCAGAAGAGGGTTGTGAGGCTGCCGCCGCGAAGCACCACCGGTTGAACTCGTGGTTGGATGCTCTGCAATTCGATGCGAGGCTTGAAGAGATCGCAGCGAAAGTAGCTGAGAAAATCGATGACATTTTTGTTGATTCTTTGCTA

>ColMADS72

ATGGAGAGAAAGGGCAAAGGCCGCCAAAAGATCACCATGACCAAAATGGAGAACGAGAGCAACCTCCAAGTCACCTTTTCAAAACGGCGATCTGGTCTCTTCAAGAAAGCCAGCGAGCTTTCAACCCTGTGTGGTGCCGAGGTGGGAATCATTGTATTCTCTCCTGGCAAAAAGGCCTACTCTTTTGGCCACCCCACTGTGGACATGATCATCGACAGGTTTCTGTCACGGGACGCTCCTCTGAACAGTGGGACTCACCAGCTGGTGGAGGCCCACAGGAGCGCCTGTGTCCGGGATCTTAACCTGCAGTTGACTCATGTCCAGGCCCTTCTGGACGTGGAGAAGCAGCGCGGCACAGCCCTAGATCAGCTCAAGATGGCCGAGGGTCAGGGTGAACATCGCTGGTGGGAGGGTCCGGTAGAGGAGATGAATCTGCCACAGCTTCATCAGTTTAAGGCTACTATGATGGAACTCAGCACTAAGATTGGAATCCGCGCTGATGGGATTGTGCATGAACAGCCTGCCAATCCCTTTGGATTTGGGGTGGGCCCGACTTCTTTTGGTGGTGGTGCTGGAGGTGCTGGATTTCATCAAGGGCCGAGTTCTTTTGGAGGTGCTGGATTTCTTGCTGGGCCGAGTTCTTTTGGTGGTGGTGGTGGTACTTCTTTTGGTGGTGGTGGTGGTGGATTTCTTGATGGGCAGAGTTCTTTTGGTGGTGGTGTTGGTGGCTCGTCATTTCAGGCGGGCCCGAGTTTTGCTGGAGGTGGTGGCATTGGTGCTTTACCATTTGGTGCAAGGCCGGGTTCTTTTGGAGCAGGTGATGGTGGCAGCGACAACAATGGCATTGTTCCATTTGATGGTAGGGTTCTAGACGCCAGTGGTTCGAAGGCTCCTTATGGATTTAACCATGGCTATGGTCGTGGTTTTTACTAA

>ColMADS73

ATGGGCCGCCAAAAGATTGAAATCAAGAAGATAGAGAAGAAAAGCCAGCTCCAAGTCACCTTCTCCAAGCGCCGTGCCGGAATATTCAAAAAAGCCGGCGAACTCGGCGTCTTGTGCGGCGCCCACGTCGCCGTCATCGTCACCTCCCCTGCCGGCAAGGTCTTCGCCTTTGGCAACCCCTCTGTTGATTCCGTCATTGATCGCTTTCTCTCCAAAAACCCTAATCCCCATGGTTTCGACTCGTGTCATGATGACGAGCTCCACCAAGTGCACCAGCAAAGTATTAATCCTAACTCTCAGTGCTCGGAGCCCCATCGAGTGAGTAAGAATCCTAACCTTAACCCTAATTCTCAGTGTTTGGACATGTGTCATGAGGAGGAGGTCCGGCAAGTGCAGCAATTGAGTAAGAAGTATGTGAAGGCGGTGGGGAAGTTGGAGGCAGAGAAGGAGAGAGGAAAGGCGTTGGCCGAGTTGAACAGCGGCGGAGGAGGAGGTGGGTTTTGGTGGGAGGAGAGTGTTGAGGGATTAGAGTTGCATGAGCTTGAGCAGTATGTGGCTGCATTGGAAGTTCTTAAGAGTAACTTATTGGCTAGGGCTGATGAGATGGCGGTGGCAGCGAGCGGTTTTCCAGCGAATTTCTTGGCTTCCAACGGTGTTGTGGTGGCTGATGCTTTTGGTAGTGCTGAGTGCAGTGGTTTTGGTTTTGATTATAAGCCTTTCTAA

>ColMADS74

ATGGAAAACAAGCAGAAAAAACACAGCATGGGCCGCCAAAAGATTGAAATCAAGAAGATAGAGAAGAAAAGCCAGCTCCAAGTCACCTTCTCCAAGCGCCGTGCCGGAATATTCAAAAAAGCTGGCGAACTCGGCGTCTTGTGCGGCGCCCACGTCGCCGTCATCGTCACCTCCCCTGCCGGCAAGGTCTTCGCCTTTGGCAACCCCTCTGTTGATTCCGTCATTGATCGCTTTCTCTCCAAAAACCCTAATCCCCATGGTTTCGACTCGTGTCATGATGACGAGCTCCACCAAGTGCACCAGCAAAGTATTAATCCTAACTCTCAGTGCTCGGAGCCCCATCGAGTGAGTAAGAATCCTAACCTTAACCCTAATTCTCAGTGTTTGGACATGTGTCATGAGGAGGAGGTCCGGCAAGTGCAGCAATTGAGTAAGAAGTATGTGAAGGCGGTGGGGAAGTTGGAGGCAGAGAAGGAGAGAGGAAAGGCGGTGGCCGAGTTGAACAGCGGCGGAGGAGGAGGTGGGTTTTGGTGGGAGGAGAGTGTTGAGGGATTAGAGTTGCATGAGCTTGAGCAGTATGTGGCTGCATTGGAAGTTCTTAAGAGTAACTTATTGGCTAGGGCTGATGAGATGGCGGTGGCAGCGAGCGGTTTTCCAGCGAATTTCTTGGCTTCCAACGGTGTTGTGGTGGCTGATGCTTTTGGTAGTGCTGAGTGCAGTGGTTTTGGTTTTGGTTTTGATCGTAAGCCTTTCTAA

>ColMADS75

ATGGGCCGCCAAAAGATTGAAATCAAGAAGATAGAGAAGAAAAGCCAGCTCCAAGTCACCTTCTCCAAGCGCCGTGCCGGAATATTCAAAAAAGCCGGCGAACTCGGCGTCTTGTGCGGCGCCCACGTCGCCGTCATCGTCACCTCCCCTGCCGGCAAGGTCTTCGCCTTTGGCAACCCCTCTGTTGATTCCGTCATTGATCGCTTTCTCTCCAAAAACCCTAATCCCCATGGTTTCGACTCGTGTCATGATGACGAGCTCCACCAAGTGCACCAGCAAAGTATTAATCCTAACTCTCAGTGCTCGGAGCCCCATCGAGTGAGAAAGAATCCTAACCTTAACCCTAATTCTCAGTGTTTGGACACGTGTCATGAGGAGGAGGTCCGGCAAGTGCAGCAATTGAGTAAGAAGTATGTGAAGGCGGTGGGGAAGTTGGAGGCAGAGAAGGAGAGAGGAAAGGCGGTGGCCGAGTTGAACAGCGGCGGAGGAGGAGGTGGGTTTTGGTGGGAGGAGAGTGTTGAGGGATTAGAGTTGCATGAGCTTGAGCAGTATGTGGCTGCATTGGAAGTTCTTAAGAGTAACTTATTGGCTAGGGCTGATGAGATGGCGGTGGCAGCGAGCGGTTTTCCAGCGAATTTCTTGGCTTCCAACGGTGTTGTGGTGGCTGATGCTTTTGGTAGTGCTGAGTGCAGTGGTTTTGGTTTTGGTTTTGATCGTAAGCCTTTCTAA

>ColMADS76

ATGGGACGGCGCAGGGTGGAGCTGAGGCCAATCCAGGACAAGCATAAACGACAAATCACCTTCTCGAAGCGCCGACAAGGACTGATGAAGAAAGCTCAAGAACTCTCCGTCCTCTGCGACGTCGACGTCGCCCTCGCCGTCTTTACCAGCGGTTGCCGTGCTTATAAGTTCTCCGGCGGCAACAGGTACGCGCTCGCTCTCTCTTTCTCTCTCATCAATATTCAAATCCTTAATGTTCGATTTTCCGACAATCGATGA

>ColMADS77

ATGACTATTATACCCACTCTCTTCCCCACTCTCTTCCTTTCCACAGATGGCCTCTTCAAGAAGACCAGCGAGCTCTGCAACTTATGCGGCACCGAAGCCGCCGTAATCACCTTCTCCAACGCCAGCAACACCTTCGCCTTCGACAACCCCTCCGTCGACTCCGTCCTCGACCGTTACCTCTCCATGACTTCATCATCCTCCAAAGCCTCCGTAAGCTTTTCTTCCCTCTTTCGACATAGGGTTTCAACTCAAATCTGTGCACCATGA

>ColMADS78

ATGGGTAGGGGGAAAATTGAGATAAAGAAGATAGAGAATGTTAATAGCCGGCAAGTCACCTTCTCTAAGCGAAAAGCCGGTCTGCTCAAGAAGGCCAAGGAGTTGTCAATTTTATGTGATGCCGAGGTTGGTCTTATAATCTTCAACAACTCAGGCAAGCTCTATGAATTCGCCAGTCCCCGCATGGAACACATTCTTGCAAGATACAACAATGGTCTTGAATCTTCTACACTTACAAATCCTTCAACTGAGAAAGCAGTATTAGAGCTTGAAAAGCAGCCCCCCGAGCTAGATGCTTTAAGAGGTGAAGTTGCAAAACTACAAAAGGGGTATAGACGAACGATGGGTAAGGATTTGGAGGGAATGAGTTTCAAGGAACTTCAACAATTGGAGCATCAATTGAATGAAGGGATATTGTCCGTTAAGGATAGAAAGGAACAAGTATTGTTGGAGCAGCTTGAGAAATCAAAATTGCAGGAGAAAAAGGTCAAGCTAGAGAATGAAACTCTGCGCGAGCAGATTGAGGAGCTTGGGCGACCAAGCACAACACCTACGCCTACTCATCAATTCCATTGTCTTGGAAGAAAGCGATCTGTTCTAAGTTCAAATACAGTTTGTGATGGTTACTTTGACACAGAAAGAGACTCAGAGACTTCTTTGCGCTTGGGGTTGTCAAGTGAAGTTTTTCATAAGAGAAAAGTATGCAAGATTGAATCGGTTTCTAATGATGACTCTGGCAGCCAAATGGCTTCATAG

>ColMADS79

ATGACTATTATAGCTACTCTCTTCCCCACTCTCTTCCTTTCCAAAGATAGCCTCTTCAAGAAGACCGGCAAGCTCTGCAACTTGTGCAGCACTGAAGCCGCCGTCATCACCTTCTCCAACGCCGGCAACACCTTCACCTTCGGCAACCCCTCCGTCGACTCCGTCCTCGACCGTTACCTCTCCATGACTGCATCATCCTCCAGAGCCTCCACAAGCTTCTCTTAG

>ColMADS80

ATGAAGAGGATCGAAAACCCGGTGCACAGACAAGTCACCTTCTGCAAGCGCCGAGCTGGGCTTCTTAAGAAGGCTAAGGAGCTCTCTGTGCTTTGTGATGCTAAAATTGGTGTTCTCATTTTCTCTACCAATGGCAAGCTCTACGAACTCGCCACTAAAGGAACCATGCAAGGGCTTGTTGGGAGGTACATGAAATCAACCGGCGACACTCAGGCCGACCATGATGAAGAAAAGCAAGTTATGGACTCAAAAGAGGAGATCAATATGCTGAAAAATGAAATTGAATTTCTGAAGAAAGGACTCAGGTTTATGTCTGGAGCCTCTTTTGCCGTGGCAGGATCAGCTACTGCACTATACCACCTGTCCAACCATGCTCCGGCCTCGCACAAGACTGCCTCACCAGCATTGCCGTCGTGA

>ColMADS81

ATGGGGCGTAGAAGAGTCGAAATCAAGAGAATCGAAGACAAAGCCAGAAGACAAACCACCTTCACAAAGCGCCGTGACGGCCTCTTCAAGAAGACCGGCGAGCTCTGCAACTTGTGCGGCACCGAAGCCGCCGTCATCACCTTCTCCAACGCCGGCAACACCTTCGCGTTCGGCAACCCCTCCGTCGACTCCGTCCTCGACCGTTACCTCTCCATGACCTCATCATCCTCCAGAGCCTCCGTGGACGGCGGCGAGGTGGCGGCGGCTGAACGAGTGAGAGAGAGGGAAGAGAAGCTTGCGGAGGCTCTGGCTAGGTTGGAGGTCGAGAAGAAGAGGGGAGAGGCGATCGATGAGGCTTTGAGTATGTTGAAATTGAATGAGGATGTCGTTGGTGATTCGAAAGTTACCGTTTCGGAGGAGATGGGGGAGATTGGGAGTGCGGTGGAGATGGCTCAATTGGAAGCGGAGAAGAAGCATGATGAGATTGTTGATGAGATGTTGGCGATGTTGAATGCGAAGGCTGAAGCTTCGAGATTGAAAGAAGTTGAACAGGAGGTTGGGAAGGATGTGGATGAGGAGACGACTGAATTGCAAATCGGAAACAAAAGCCATAGAGGCGATCGATGA

>ColMADS82

CGTACCTGCCGGCATATATGTTTACGGCGTTGCAATGTCGATATCGGTGTAACCCTAGAGTGGAATGGGTATGAGAATATGTATGTGCTCCCTCTGTGCTTTGACTATGGTGATCAAATATTCTATAATATTCTGTGTGACACAGTGAAGGAAAGGGAAACCATGCATCGGCGAAGGCGGCACGGACAAAGGAAGATGGCGAAGGAGAATGGCGAAAGAGGACGACGAATATTCACCATGGCAAAGAAACCAAGCATGGGTCACCAAAAGATCAAAATTGCGAAAATAGAGATAAAAAATCACTTGCAAGTCACCTTCTCAAAACGCCGATCTGGGCTTTTCAAGAAGGCAAGCGAACTTTGCACGCTTTGTGCCATAGAGATTGCTATCATAGTTTTCTCTCCGGCTAGCAAAGTTGTGCGCGAGCTCAACTTACAACTTACTCAAGTTCTCAATGAATTGGAAGCCGAGAAAAGACGTGGTGAAACCTTAGATCACATGAGAAAAGCTAGTCAAAGACAATACTGGTGGGAAGCTCCAATAGACAAGCTTGGCTTGCATGAGCTTGAACAACTTAGGAATTCAATGGTGGAGCTCAAGAAGAAGGTAACGAATCAAGTGAACAAAATTCAAGTCGAGGGCACCACAAACCCTTCGCCATTTTTCTTAGTAAATGGCACCAGAATGGTTGATCACTTTGAGAGTAAAACATCTCATATCAATGCTTCTTCTTCAACCACTCCTAATGTGCACTACTTTGGCTATTATGGTCATGGATTTTTTTAA

>ColMADS83

ATGGTGAGAGGGAAAACTCAGATGAAAAGGATAGAAAATGCTACAAGTAGGCAAGTGACTTTTTCTAAACGCAGAAGTGGGCTTCTCAAGAAGGCCTTTGAGTTATCAGTCCTTTGTGATGCTGAAGTTGCACTCATCATCTTCTCTCCAAAAGGGAGGCTCTACGAGTTCTCAAATTCCAGGTGCCTCAGTATTAGCAAGACAATAGAACGGTATCAGAGAAATACCAAGGAACAAGAAGGAAGTGGCAGAAAAGAAGCTGAAGAAAATTTGCAGCATTTGAAGGAAGAAGCTGTTTCCATGTCACAGAAGATTGAACTTATTGAAGCCTCTAAACGAAAGCTCTTGGGTGATTGTTTGGAGTCTTGCTCTATCAATGAGCTACAACTAATAGAAGAACAGTTGGAGAAAAGTTTACACAACATTAGGGCAAGAAAGACTCTTTTATATAAGGAGAAGATCGAGCAATTGAAGGAAGAGGAGAAAATCCTAACGGAAGAAAATCAAAAATTACGGGAGAAGTGTGAGATGCGATCGCCACATCTGCCAATTCCGCTGCTAGTAGCTCCCGAGCCTAATCGTGAATTTTCAGATGTTGAGACGGAATTGTTCATAGGACTACCCGAACAACGAACTACTCATTCTCTCTAG

>ColMADS84

AAAACCACCCAAGGCCATCAGAAGATCGAAATCAAGAAGATTGAGAGTGTAAGCAGTCGCCAAGTCACCTTCTCCAAACGCCGCGTCAGGCCTCTTCAAGAAGGCCAGCGAGCTCTGCATCCTCAGCTGGCGCTAAGATTGCCATTGTCGTCAAATCTCCCAGAAAATGCACTTTCACTTTCGCATGCCCCAGCGTCGACGCCGTCATCGATCGATACCTCACCGGAACCACCGCCGTTGAAGAGTAGAACAACCGCCCTTGGGTGCGCGATTTCAAAGAGCGATACGCCCAAGTTTCCAAGGAATTGGAGGCGGAGAAGAGACGGAGCGCGGGTTAGAGGAGCTGGAGCAGTATGTGGCAGCATTGGAGGAATTGAAGAAGAATGTGGCAATGAAAGCTAA

>ColMADS85

ATGGTGAGAGGGAAAACTCAGATGAAAAGGATAGAAAAGGCTACAAGAAGGCAAGTGACTTTATCTAAACGCAGAAGTGGGCTTCTCAAGAAGGCCTTTGAGTTATCAGTCCTTTGTGATGCTGAAGTTGCACTCATCATCTTCTCTCCAAAAGGGAGGCTCTATGAGTTCTCAAATTCCAGGGCGGCTGCTATGGGTGGTTCTACTGTTTTATGGGATATTTTCTGCTGTAATCTGCTGATAGCATTATGGCATTATCTCTGTGCTCAGGTCTGA

>ColMADS86

ATGGGAAGTGGAAAAAGGAAAATTGAGATTAAAAGAGTGGAGAAGGAAGGACAGAGAATGGTTACATTCTCCAAGAGATGTCAGGGTCTCTTCAACAAAGCCCGACAACTCCGATCCCTCACCGGCGCCGACATCGCAATCCTCACCTTCTCCCCCGCTGGCCGTCCCTACACTCACGCGAGCCCTTCTTCGACGCCCTTGTCGACCGTTACCTCAACACCGCCGTCGCCGGAGAGAAGGCAGAAGAGGGTTGTGAGGCTGCCGCCGCGAACCACCATCAGTTGAGTTCGTGGTTGGATGCTCTGCAATTCAATGCGAGTGATAGCATTGAAGATTTGGAAATCTTGAAGAAAGGGCTTGAAGAGATCGCAACGAAAGTGGCTTTGAAAATCGATGACGTTTTTGTTGATTCTTTGCTCGTTTGA

**#amino acid sequences of MADS-box genes in *Camellia oleifera***

>ColMADS01

MTIIPTLFPTLFLSTYGLFKKAGELCNLCGTEAAVITFSNAGNTFAFGNPSVDSVLDRYLSMTSSSSRASASFFQLKSVHNSCKSQ

>ColMADS02

MYVLPLCFDYGDQIFHNILCDPVKERETMHRRRRHGQKKMVKEKENGERGRRIFKKSSSIVLAMAKKPSMGRQKIKIEKIEIKNHLQVTFSKRRSGLFKKASELCTLCGIEIAIIVFSPAGKVFSFGHPNVDSIVDQFLTRNPPPNTTTRHLIEAHRSASVRELNLQLTQVLNELEVEKRHGETLDHTRKASQRQYWWEAPIDKLGLHELEQLRNSMVELKKNVTNQANKIQFEGTTNPSPFFLANGTRMVDHFESKTSHINASSSITPN

>ColMADS03

MTIIPTLFPIFFLSTYGLFKKTGELCNLCGTEAAVITFSNAGNTLAFGNPSVDFVLDRYLSMTSSSFKASASFSSLFRHRVSTQICAP

>ColMADS04

MVRQRIQIKKIDNVTARQVTFSKRRRGLFKKAHELSTLCDAEIALIVFSATGRLFEYASSSMKQVIERHNLQPQNLVHLNQPSLELQLENSTRAMLSKEVEERTLELRQLRGEELHELGFEELKKLEKSLEGGLSRVLKTKPLRGKLNFQMQIVNMGQPQEQGQSSESITNNGSTVAPPQDYDSSDTSLKL

>ColMADS05

MTRKKVKLAFITNDSARKATFNKRKKGLMKKASELSTLCGIDACVIIHSPYKSQPEVWPNNLGVQRVLAKFERMPKMEQSKKMETQESFIRQRIAKANEQLKKKLKDNREKEMTEIMYQCLSGRGLQNLSMVDQCDVNWLIDKNLKEIGKKIESLKKTPQQVAPVALKNTKEMLGNGMHRKKEKLLGVDLAMDAMNRSHGFNEWMNNPHYENMGFDGDKMMVPFGDNHNLMWSSFFFP

>ColMADS06

MVRGKTQMRRIENATSRQVTFSKRRNGLLKKAFELSVLCDAEVALIVFSPRGKLYEFASSRYDLCIYYLYY

>ColMADS07

MGRVKLEIKRIENNTNKQVTFSKRRNGLIKKAYELSVLCDIDVALIMFSPSARLTHFSSPKTRLEDVFTRFINLSDREMSSTLNQLWIENEVALHQTNIFKPDPSNLTSLEELESCEKKLEHILTTVTEKKEHLLNNQASSSSYNQSCMQEMLQQQQQTPIFCENTVVDSLLPDNGQNNHAQMFDASVPFIPLRDASNTVYEPMLQGSSSQMDPQGTWGCRVDQSDIENLPAWHHAYASTDPNSMPPPHLFSPIQHGMEEPVMMATRSDQVETASNSSLEQPHTTATNYE

>ColMADS08

MELIRNEKARYVTCQKRKKGLKKKTCELETLCDVQVCLIIYGPKLDDHSTEVEIWPQNPNDIQRLIDSYRNQSIEDRHKRTLDLSNFFENRNQKIEDALIKLRKKNDKALYSTWDDRYNDLSERQLRAFEGMLEGKLADVKARVEFMKGTLALASLNDLEQTSQQNHSNYFMQGLFGTRSLQMGIIHEETHISSLNPLYEIDNISLHYPFDQIDHQGIVELDAKSMAISPMMMPMNNINDTHYTQFGGMSSSNLQCIDPLEKSIYYDPIMLLPSSEPVLIEASCIESSFSMSRMIENAVGKNHHPSPSMGYRGGSSMQTMSPYMQYLKMTSGPSQVHAWQIEEYYKAIEFQMKNQK

>ColMADS09

MGRGKIEIKRIENSSNRQVTYSKRRNGIMKKAKEITVLCDAQVSLVIFASSGKMHEYCSPSTTLVDILDKYHKQSGERLWDAKHENLSNELDRIKKENDSMQIELRHLKGEDITSLHHKELMAIEEALENGLGSVREKQASLMILLMEYIDMMEKNKKMLEEENNHLNFMLHQQEMNMESSREMENGYHQRVRDFQSQMPFAFRVQPIQPNLQERI

>ColMADS10

MGRQKIEIKKIEKKSQLQVTFSKRRAGIFKKAGELGVLCGAYVAVIVTSPAGKVFAFGNPSVDSVIDRFLSKNPNPNPNSHGFDTCQDDELHQVHQQSINPNPNPNSQCSELHRVSKNPNPNPNSQCLDMCHEEEVRQVQQLSKKYVKAVGKLEAEKERGKAVAELNSGGGGGGFWWDESVEGLELHELEQYVAALEVLKSNLLARADEMAVAASGFPANFLASNGVVVADAFGSAECFGFDCKPF

>ColMADS11

MERKQTQGRKKIEMKMIADENARRITFSKRRHGLFKKASELSTLCGVDMAIVLFSMGGKAFSFGKPNVDSVVDRFLNQNAQPSEGVSSHASRHDDATVHQLNQQFHELSKRLKAEKKKEKIVQNMSTNNYGLLSCRFDAYVNQLDLQQLEQLKRSMVELKKNVAQRVDELNSEGSSKHAQEEEQVENTEINDNDVSTIPHDWLRL

>ColMADS12

MEFQNQSMDVSRLSPQRKMGRGKIEIKRIENTTNRQVTFCKRRNGLLKKAYELSVLCDAEVALIVFSTRGRLYEYANNRY

>ColMADS13

MTIIPTLFLSTYGLFKKTDELCNLCGTEAAVITFSNAGNTFAFGNSFVDSVIDRYLSMTSPSSKASASFSSLFQHRVSTQICAP

>ColMADS14

MTIISTLFPTLFLSTDGLFKKTGEFCNLCGTEAAVITFSNASNTFAFSNPSVDSVLDRYLSMTSSSSRASVSFS

>ColMADS15

MKYEFIADERVRKQTFRRRKAGLLKKVSVLETLCSVDACAIIYPATTTGSVAQPDVWPSPNEASHLLRRFENLPSMKKTANMMDLENFLKENITRMSKYLDKEKKKNRGVEIGQQMTKFLFEKNLHDFSNLDDLRDVASLLDEKINLMNDKLENIANEIGSGSRCGEKKQQSI

>ColMADS16

MGRTVKYEFIADERVRKQTFRKRKAGLLKKVSELKTLCSVDACAIIYPTTATGSVAQPDVWPSANEASHLLQRFENLPSMKQTANMMDQEKFLKRNITRMSKNLDKEKKKNRGVEIEQQMTKFLFEKNLHDFSNLDDLRDVASLLDEKIDLMNDKLENIANEIGSGSRCGEEKQQSI

>ColMADS17

MGRGKIEVKRIENNTSRQVTFSKRRTGLLKKTHELSVLCDAQIGLIVFSSKGKLFEYCTHPLSMGEMIGRYLDATGIRIPEHDDREQIFNELTRIRNETHNLQLSLQRYKGEDLSSARYEDLDELERQLESSVNKVRARKIKFEIEEDNLREKEPARPSLIDHHAFLRA

>ColMADS18

MGRGKIEIKRIENTSNRQVTYSKRRNGILKKAKEITVLCDAQVSLVVFGSSGKMHEFCSPSTTLVDILEKYHKQSGKRISAMNWIESRKKMTSLQIELRHLKGEDITSLQPRDLMAIENALETGLESVRYKQSEIHRMMKKNGKMLEEDNKQLNLILHQQEMEKGSREFENGYHRQVNDYQPQMPFTFRVQPIQPNLHERI

>ColMADS19

MGRGKIVIRRIDNSTSRQVTFSKRRNGLLKKAKELAILCDAEVGVMVFSSTGKLYDFSSSRSSLSLFFYVFFFSPYIEC

>ColMADS20

MNTTKKTTQGRQKIEIKKIESVSNRRVTFSKRRAGLFKKASQLCILSGAEIAIVVKSPGKRTFAFGQPSVDAVIDRYLTRTSAAAEQNNRPSVLDFNEKYAQVSKELEAEKRQRAVIEETKKAANDGGFWWDEAVDDLGLEELEQYVAALEELKKIVSMKADELMIMKANSSMMFGMNESSNHGGLGLMSDCATSTVPHGFDCANSSLMFGMNESSNHGGLSLMSDCATSTVPHGFDCANSSMMFGMNESANHGGLGFMSDCATSTVPHGFDCANSSMMFGMNESSNHGGLSLMSDCATSTVPHGFDCANSSMMFGMNESANHGGLGFMSDCATSMIPHGFDFGRGQF

>ColMADS21

MVTMNTTKKTTQGRQKIEIKKIESVSNRRVTFSKRRAGLFKKASQLCILSGAEIAIVVKSPGKRTFAFGQPSVDAVIDRYLTGTSAAAEQNNRPSVLDFNEKYAQVSKELEAEKRQRAVIEETKKAANDGGFWWDEAVDDLGLEELEQYVAALEELKKIVSMKADELMIMKANSSMMFGMNESSNHGGLGLMSDCATSTVPHGFDCANSSMMFGMSESSNHGGLSLMSDCATSTVPHGFDCANSSMMFGMNESANHGGLGFMSDCATSTVPHGFDCANSSMMFGMNESSNHGGLSLMSDCATSTVPHGFDCANSSMMFGMNESANHGGLGFMSDCATSMIPHGFDFGRGQF

>ColMADS22

MNTTKKTTQGRQKIEIKKIESVSNRRVTFSKRRAGLFKKASQLCILSGAEIAIVVKSPGKRTFAFGQPSVDAVIDRYLTGTSAAAEQNNRPSVLDFNEKYAQVSKELEAEKRRRAVIDEAVDDLGLEELEQYVAALEELKKNVSMKAGELMIMKANSSMMFGMNESSNHGGLGLMSDCATSTVPHGFDCANSSMMFGMNESANHGGLGLMSDCATSTVPHGFDCANSSMMFGRFDE

>ColMADS23

MGRMVDYEFIADERVRRQTFRKRKAGLLKKLSELKALCSVDAFAIIYPTTTTGSVAQPDVWPSPSEANHLLQRFKNLPSPQQAANMMNQEKFLRQNILRVSKNFDKEKQKNRRVDIEQQMTKLLFEKNLHDFSNLDDLRDVASLLDEKINLVNDKIENITRDENGNEIRSGSRRGKEKQQSI

>ColMADS24

MGSRRKKIEIKRVEKEGQRMVTFSKRRHGLFNKARQLRSLTGADIAILTFSPAGRPYTHGEPSFDALVDRYLNTVAAGEKAEEGCEAAAATHHRLSSWLDALQFDASDSIEDLEILKKGLEEIAAKVTKKIDDVFVDSLLV

>ColMADS25

MVTFSKRRQGLFNKARQLRFLTGADIAILTFSPAGRPYTHGEPSFDALVDRYLNTAAAGEKAEEGCEAASTNHHQLSSWLDALQFDASDSIEDLEILKKGLEEITTKVAEKVDDLFVDSLLF>ColMADS26

MYKKRKACIIKKTMELSILCDINAFTICFGPNNEVETWPENPTHVKTLINAYKNNRRGKQLVQAQQQQPSDHHRGLDDKIRGLLGWNDGWLCGLSREQLMSLWKLMRLKLQELTRRIELLMFSKMHYQYQPLIEPPMFVPLWPLNCYGVPGSSGGDGGELSVPLTWEFNETLEETTLNAMNNSDINQMCLQFSSPLNELLFASMYTMNPLQITEPQLGEETINSSDTDSQANPSLNVNMDWIV

>ColMADS27

MYKKRKACIIKKTMELSILCDINAFTICFGPNNEVETWPENPTHVKTLINAYKNNRRGKQLVQAQQQQPSDHHRGLDDKIRGLLGWNDGWLCGLSREQLICLWKLMRLKLQELTRRIELLMFSKMHYQYQLLIEPPMFVPLWPLSCYGVPGSSGGGALSVPLTWEFNETLEETTLNAMNNCDINAMNNYDINQMCLQFASPLNEFLFASMYTMNPLQITEPQLSEETISSSNTDSQANPSLNVNMDWII

>ColMADS28

MYKKRKACIIKKTMELSILCDINAFTICFGPNNEVETWPENPTHVKTLINAYKNNHRGKQLVQAQQQQPSDHHRGLDDKIRGLLGWNDGWLCGLSREQLMSLWKLMKLKLQELTRRIELLMFSKMHYQYQPLIEPPMFVPLWPLNCYGVPGSSGADGGAGGELSVPLTWEFNETLEETTLNAMNNCDINQMCLQFSSPLNELLFASMYTMNPLQNTEPQLGEETIISNDTGSQANPSLNVNMNWII

>ColMADS29

MYKKRKACIIKKTMELSILCDINAFTICFGPNNEVETWPENPTHVKTLINAYKNNRRGKQLVQAQQQQPSDHHRGLDDKIMGLLGWNDGWLCGLSREQLMSLWKLMRLKLQELTRRIELLMFSKMHYQYQPLIEPPMFVPLWPLNCYGVPGSSGGGGGGELSVPLTWEFNETLEETTLNAMNNCDINQMCLQFASPLNELLFASMYTMNPLQITEPQLSEETISSSDTDSQANPSLNVNMDWII

>ColMADS30

MYKKRKACIIKKTMELSILCDINAFTIFFGPNNEVETWPENPTHVKTLINTYKNNRQGKQLVQAQQQQPSDHRGLDDKIRGLLGWNDGWLCGLSREQLMSLWKLMKLKLQELTRRIELLMFSKMHYQYQLLIEPPMFVPLWPLNCDGVPGSSGGGGGGELSVPLTWEFNETLEETTLNAMNNCDINQMCLQFSSPLNELLFASIYTMNPLQNTEPQLGEETISSSDTDSQANPSLNVNMDWII

>ColMADS31

MYKKRKACIIKKTMELSILCDINAFTICFGPNNEVETWPENPTHVKTLINAYKNNRRGKQLVQAQQQQPSDHHRGLDDKIRGLLGWNDGWLCGLSREQLMSLWKLMRLKLQELTRRIELLMFSKMHYQYQLLIEPPMFVPLWPLNCYGVPGSSGGDGGGGGGGGALSVPLTWEFNETLEETTLNAMNNCDINAMNNCDINQMCLKFASPLNELLFASMYTMNPLQITEPQLSEETISSSDTDSQANPSLNVNMDWII

>ColMADS32

MTRNKIKLAWIVDKSSRKATLKRRRASIFKKAGELSVLCDVEVGVIVYSHKEADLAVWPSYGHMKQMFERFLSIPIVERNQKMMTNEGFLTQRVTQETNKNNMEKKKNDKKEIQEIMNQVFEGSNLYQLNIMRLNYLSLLTVDKLKQLEERERARGQQAPPLLPLLIPAPALGPAPGMMEVNEVSQAHTSIEATSIEQLMNDNWFMETMPLNWDMLGPSGVSDMGMPSAPVVGRMNGDNEEDLPDDLNAFFPHIYSP

>ColMADS33

MGRGKIVIRRIDNSTSRQVTFSKRKNGLLKKAKELSILCDAEVGVIVISSTGRLYEFASPSMRSIFERYNNEQENHQLLSPTSEAKLWQREAESLRQQLNYLQESHRKLTGEELSDLSVEDLENLEGQLETSLKGIRTKKHQILTDEIQELNHQGVISHQENMKLYTEVKVLRRENAELHKKVYGANDINRSSFKPYGCSNGYDLHVPIQLQLSQPQQTHNETPETQGI

>ColMADS34

MARGKVQMKRIENPVHRQVTFCKRRAGLLKKAKELSVLCDAEIGVLIFSANGKLYELATKGTMQGLVGRYMKSTGDTQADHDEEKQVMDSKEEINMLKNEIEFLKKGLRFMSGGGVEAMTLDELHMLEKHLEIWIYHIRSVKMDIMSQEIQLLKTKKHMKQNDQVTAAKAEAIVKVVEKKAAKVEKKLTEALTNKEQTSTPIKKAKTLSKINIRFKSTSRI

>ColMADS35

MGRGKVQLKRIEDKNSRQVTFSKRRTGLIKKAHELSILCDVEIALIVFSARGKLYQFCTGDSLRKVLERYQIHKDAEVAGSSVQESKKLTEGYMDFSRGTNLLQMVQRHFEEQKIEQLDVAELTQVEHQLDAILRQTRIKKSQLMMKAVTALHEKEEQPREGRQLMEKEITAMINEATMDDDCRHQQQQTQQGDPDMDLELYGYTNNTNNNNNSSTGSGGGGVYHHLQQEESMFYLL

>ColMADS36

NDDDEGFEFGVEIKVGKGYDDRFVVFFVFEIIVLEQNSFERAKSKSRDSLVLVKSNCPEEICTEAIRSSEVHLVADRFRQTKERNSSRQTEQSTRTEQQQAQMGRAKQRMELIRNEKARYVTYQKRKKGLKKKTCELETLCDVQVCLIIYGPKLDDHSSEVEIWPQNPNDIQRLIDTYRNQSIEDRHRRMLDLSNFFENRSQKIEDALVKLRKKNDKALYSTWDDRYNDLPEGQLRAFEGMFEGKLADVKAKVEFMKGTQALASLNDMEQTTQQNHSNYFMQGLFGTTSLQMGIIHEKTPISSLNPLYEIHGISLHYPLGQTDHQGIVELDAKSMAISPMMMPMNIDNHYTQLGGMSSNNLQCIDPLEKSINYDPIMLLPSPESVLTGVSCTESSFSMSRMMENEVGKKHHPSPSVGYYGGSSMQTMSIATPYLKMASGPSQVHAWQMKEYYKANEFETKNQ

>ColMADS37

MELIRNEKARYVTYQKRKNALKKKTCELETLCDVQVCLIIYGPELDDYSTEVEIWPQNPNDIQRLIDSYRNQSIEDRHRRTLDLFNFFENRNQEIEDALVKLRKKNDKSLYSMWDDRYNDLPEGQLRAFDGMLEGKLVDVKARVEFMKGTRALASLNDMEQTTQQNHSNYFMQGLFGTTSLQMGIVHEETPISLLNPLYEIDDVSLHYPFDQTDHQGIVELDAKSMAISPMMMPMNNINDTHYTQLGGMSSSSLQCIDPLKTSIYYDPIMLLPSPEPVLTGVSYTESSFSMSRMMENEVGKNHHPSPSMGYCGTSSMQTMLPYMQYLKMASGPSQVHAWQMEEYYKANEFQMKNQK

>ColMADS38

MLRNKKYAPRPFARAKFTLLLIDSVSSYPIQLIPAYEYVDKQTKERNSSTQTEQSTRTEQQQAQTPILMFALGPEKLKASPGHNPTNRALTNENDMGRAKQRMELIRNKKARYVTYQKRKKGLKKKTCELETLCDVQVCLIIYGPKLDDHSTEVEIWPQNPNDIQRLIDTYRNQSIEDRHRRTLDLSKFFENRNQKIEDALVKLRKKNDKALYSTWDDHYNDLPEGQLRAFEGMLEGKLVDVKAKVEFMKGTRALASLNNMEQTTQQNHSNYFMQGLFGTTSLQMGIIHEKTPISSLNPLYEIDGISLHYPFGQTDHQGIVELDAKSMAISPMIMPMNNINDSHYTQLGGMSSSNLQCIDPLEKSIYYDPIMLLPSPESVLTGVSCTESSLSMSRMMENEVGKNHHPSPSVGYYGGSSMQTMSPYMQYLKMASGPSQVHAWQMKEYYKANEFEMKNQK

>ColMADS39

MGRGRVQLKRIENKINRQVTFSKRRSGLLKKAHEISILCDAEIALIIFSTKGKLFEYSTDSCMERILERYERHLMGEDLDTLNLKELQNLEHQLDSALKHIRTRKNQLMYESISELQKKDKALQEQNNLLTKQIKEKEKKEKEQQAQLEQHQNHDLNSSSVVISQSLHSLNIGGAYQAAVGDVEGAPHQIQNNAVMPPWMISHING

>ColMADS40

MGRVKLQIKRIENNTNRQVTFSKRRNGLIKKAYELSVLCDIDIALIMFSPSGRLSHFSGKRRIEDALSRYDFQQEINNLQQQLQMAEDQLSIFEPHVLGFTSMEELESCEKNLLESMNRVMQRKKYLMGDHLSTFDMQMNSMGQMFFDAQEEGMPTASFQNELVCWLPENNNGGDNQNHIFGASTSDHSCIRLGSNSAATIYNEIPNNASAGGVNGEANSIGSGGYPSDDHNSLSHQWHHASTSTHDLFSSLIPPTSFPQNDMASPNVGAVEQLQQVEATQSCPGPPEVELEVELPSIEQDAKF

>ColMADS41

MGRAKQNMELIRDDKTRYVTFQKRKKGLKKKTYELQTLCDVEVCWIIYGPKINDRPTDDQAEIWPPNPDVIQHLIETYKNLSSEDRTRRTLGLPNFFEDRNQKIEEMLVKLQKKNDEARYSTWDDRYNGLSKEQLKEFESVLEGKLQDVKAKVAFMKGSDQKLLVACIDDNSHTQTIQNQSYFMHGGMFGIEVDNDIANLQYYFNQAQVDANSKANPMMMTTMMPTMMLRNNNQYYNKLGGVSSSNFMCNPLDRPIYYDPMMLENAIVNNHLPPVGYCNLSIQTTSPSSSVHASQLDKDYKPGNFK

>ColMADS42

MGKAKQNMELIRNDKTRCVTFQKRNKGLKKKTYELKTLCDVEVCLIIYGPKINDRPTDDQAEIWPPNPNVIQHLINIYKNQSSEDRMRRTLGLSNFFEDRNQKIEDMLVKLRKKNDEARYSTWDDRYNGLSKEQLREFESVLEGKLQDVKARVAFMKGIDQKLSVACINDNSHTQTTQNQSYFMHGGMFGTEVDNDIANLQYSFNQAQVDANSKANPTMMMTMMPRNNNQYYNQLGGMSSSNFMCNPLDWPIYYDPMMLENMIVNNPSLPVGYYNPSIHTTSSSSSAHAS

>ColMADS43

MGNGKKKIEIKRVEKEGQRMVTFSKRRRGLFNKARQLRSLTGADIAILTFSPAGRPFTHGEPSFDALVDRYLNTAAAGEKAEEGCEAAAANHHRLSSWLDALQFDASDSIEDLEILKKGLEEIAVKVAEKIDDIFVDSLLV

>ColMADS44

MGNGKKKIEIKRVEKEGQRMVTFSKRRRGLFNKARQLRSLTGADIAILTFSPAGRPFTHGEPSFDALVDRYLNTAAAGEKAEEGCEAAAANHHRLSSWLDALQFDASDSIEDLEILKKGLEEIAVKVAEKIDDIFVDSLLV

>ColMADS45

MGSGKRKIEIKRVEKEGQRMVTFSKRCQGLFNKARQLRSLTGADIAILTFSPAGRPYTHGEPFFDALVDRYLNTAVAGEKAEEGCEAAAANHHQLSSWLDALQFNASDSIEDLEILKKGLEEIATKVALKIDDVFVDSLLV

>ColMADS46

MATPNRCIATPKISRFVNEVEGLFFFVVPTFILVGVERWEEKSSSIVLAMAKKPSMGRQKIKIAKIEIKNRLQVTFSKRRSGLFKMASELCTLCGIEMAIIVFSPTGKVFSFGHPNVDSIVDQFLTRNPPPDTTARHLIEAHRDASVHELNLQLTQVLNELEVKKRHGETFDHTRKASERQYWWEAPIDKLGLHELEQLRNSMVELKKNVTNQANKIQVEGNTNPLPFFLVNGSGIVDPFESKTSHINASSSTGPNLSQQPNEP

>ColMADS47

MEDDENGKKPMKRIDNKKALRVTYIKRKDCVIKKTMELSILCGINVFTLCFGPNGEVDTWPQNPNEVKALIKMYKECQEKPVAKKVLVGNEDGYGWLDGLSAESAMSFLGKLESKLEVLKARNEFLKMVKEQKNRGVVVMGKETEKTSCWFDDTNAIDNLQKNRGVIVMGKETEEACWFDDTSAIGNFEEDDLAMLLEP

>ColMADS48

MGRVKLKIKRLESTSNQQVTYLRRRNGIFKKAKELFILCDIHIVLLMFSPTGKATLFRRERSNIAEVITKFAQLTPQ

>ColMADS49

MFKKIDDLSTLCDVKAGTVIYNLGEAEPMVWPSYEYMKQMFEKFLSMSIIDRSQKMVTYERYLIQMITKESEKNNREKKNNDKKEIQEIMNQMFEGNDLNELNMMKLNLLSLLADDKLKQLKNRQSRPRGQQMALFPPPPPLPLSPVPAPKIIEVEEPVPAPEMIEVEGPIPAPEMIEVERQVPAPERIEVERVVEDGGSLIPMIIQELMNDQCFMETTAERMEQLLSEDVDGTSYSENKEDLWEDLDEVFHHIYFP

>ColMADS50

GERERKREKMRRGKVELKRIENPSSRQVTFSKRRNGLLKKAFELSILCDAEVALLMFSPSGKAYQFCSHDMDRTISRYRSEVRISQFNDQGLRTMEVRRSEMDELKSTIETLEARQKHFAGEDLSMLSMKELKLIERQLRNGVERVRSKT

>ColMADS51

MRRGKVELKRIENPSSRQVTFSKRRNGLLKKAFELSILCDAEVALLMFSPSGKAYQFCSHDMDRTISRYRSEVRISQFNDQGLRTMEVRRSEMDELKSTIETLEARQKQFAGEDLSMLSMKELKLIERQLRNGVERVRSKT

>ColMADS52

MKDDEKGKKPMKRIENKKALRVTYMKRKDCVIKKTMELSILCGINVFTLCFGPNGEVDTWPQNPNEVKALIKMYKESAKKSPVRKTCESSFSDCCLDAEKVAKKVWVGNEDGYGWLDGLSAESAMSFLGKLESKLEVLKARIEFLKMVKEQKNRGAVVMGKETEETSCWFDDTNVIDNLQKNRGVIVMGKETEEACWFDDTSAIDNFEGDDLAMLLDLYHQYQPGIF

>ColMADS53

MEDDEKGKKTMKRIENKKALRVTYKKRKDCVIKKTMELSILCGINVFTLCFGPNGEVNTWPQNPNEVKALIRMYKESAKKSPVSETCESSFSDCCLGAEKVAKKVLGGNEDGYGWLDGLSAESVMSFLGKLDSKLEVLKARIEFLKMVNEQKNRVVVMGKETEETCWFDDTNAIDNLQKNRGVIVMGKQTEPCYQPQPQPQPQQQQQAIQLNFEMTNASMLMEPCYQPQQQQQDTQLNFVMDNLSMLLDLCHQYQSGIF

>ColMADS54

MEDDKKGKKPMKRIENKKALRVTYMKRKDCVIKKTMELSILCGINVFTLCFGPNGEVDTWPQNPNEVKALIRMYKESAKESPVSKTCESSFSDCCLGAEKVAKKVLVGNEDGYGWLDGLSAESVLSFLGKLDSKLEVLKARIEFLKMVNEQKNRGVIVMGKQTEEACWFDDTSAIDNFKEDCLARKDCVIKKTMALSILCGINVFTLCFGPNGEVDTWPHNLNEVKALIKMYKESTEKSPIRKTCESFKVAKKVLLGNEDGYGWLDGLSTESVMSFLEKLESKLEVLKARIEFLNMCGKKHKKLVGLMIRMRLTICKKNREVMVMGKETEEACWLDDTNAINNFEDYLAMLLEPYYQPQPQQQTSQLDFEMNNF

>ColMADS55

MKDDEKGKKPMKRIENKKALRVTYKKRKDCVIKKTMELSILCGINVFTLCFGPNGEVDTWPQNPNEVKALIKMYKESAKKSPVRKTCESSFSDCCLDAEKVAKKVWVGNEDGYGWLDGLSAESAMSFLGKLESKLEVLKARIEFLKMVKEQKNRGAVVMGKETEETSCWFGDTNVIDNLQKNRGVIVMGKETEEACWFDDTSAIDNFEGDDLAMLLDLCHQYQTGIF

>ColMADS56

MKDDEKGKKPMKRIENKKALRVTYMKRKDCVIKKTMELSILCGINVFTLCFGPNGEVDTWPQNPNEVKALIKMYKESAKKSPVRKTCESSFSDCCLDAEKVAKKVWVGNEDGYGWLDGLSAESAMSFLGKLESKLEVLKARIEFLKMVKEQKNRGAVVMGKETEETSCWFGDTNVIDNLQKNRGVIVMGKETEEACWFDDTSAIDNFEGDDLAMLLDLCHQYQTGIF

>ColMADS57

MGRGKIEIKRIENATNRQVTYSKRRNGIMKKAQELTVLCDAKVSLIMFSNTGKFHEYTTPNITTKKIYDQYQKTLGIDLWSTHYERMQGHLKKLKEINNKLRREIGQRVGGEDLNDLSIQELCGLEQKMAASLTDVRQRKYHVLKTQTETYKKKVRSMEERHGNLLLNFEARCEDPQFGTLFENDRDYDYAFCLHRGGGFGSNELRLA

>ColMADS58

MGRGKIEIKRIENATNRQVTYSKRRNGIMKKAQELTVLCDAKVSLIMFSNTGKFHEYTTPNITTKKIYDQYQKTLGIDLWSTHYERMQGHLKKLKEINNKLRREIGQRVGGEDLNDLSIQELCGLEQKMAASLTDVRQRKYHVLKTQTETYKKKVRSMEERHGNLLLNFEARCEDPQFGTLFENDRDYDYAFCLHRGGGFGSNELRLA

>ColMADS59

MARKMAGGRRKIEMKLIPSKSARQVALSKRRLGIFKKANELCILTGCEIGIVVFSPSGKAFSFGHPSVDTIVQRSLYESPMPAAIGQDSHGSIVPALSQEYTKMCRQLGAEKRRGKELKESAMKCQRPYWLDAPIHELNLDQVLDSKKCMEELRAKIAKRVNELSVEGSASNLASSAKFVRGIDLNMARGCRKIEMKLIPSKSARQVAFSKRRLGIFKKANELCILTGCEIGIVVFSPSGNAFSFGHPSVDTIVQRSLYESPMPAAIDQDSHGSIVPALCQEYTEMCRQLGVEKRRGKELKESAMKCQRPYWLDAPIHELNLDQVLDSKKCMEELRAKIAKRVHELSVEGSASNLASSAKLVRDIDLNGPLGPPQEREPENQVTPPNRLIRAGILSYVLQGGLQTFFCTDTLRPIVPPGRLLSPCKLLLECSDE

>ColMADS60

MGRGKIEIKKIENVNSRQVTFSKRKAGLLKKAKELSILCDAEVGLIIFNNSGKLYEFASPRMEHILARYNNGLESSTLTNPSTEKAVLELEKQPPELDALRGEVAKLQKGYRRKMGKDLEGMSFKELQQLEHQLNEGILSVKDRKEQVLLEQLEKSKLQEQKVKLENETLREQIEELGRPSTTPTPTHEFHCLGRKPSVLSSNIVCDGYFDTERDSETSLRLGLSSEVFHKRKVCKIESVSNDDSGSQMAS

>ColMADS61

MTIIPTLFPSTDGLFKKTSELCNLCGTEAAIITFSNAGNTFAFGNPSVDSVINRYLSMTSSSSKASASFS

>ColMADS62

NDDDEGFEFGVEIKVCKGYDDGFVVFFVFEIIFLSKTPSREPNRREIVQFLSNPTAQKKYPPRPFAGAKFTLLLIDSVSSYPIQSVPAYEYVDTVSSSNTTEAFPKPTKQRSSSTQTEQSTRTEQQQAQMGRAKQRMELIRNEKARYVTYQKRKKGLKKKTCELETLCDVQVCLIIYGPKLDDNSTEVEIWPQNPNDIQRLIHTYRNQSIEDRHRRTLDLSNFFENRNQEIEDALVKLRKKNDKALYSTWDDRYNDLSEGQLRAFEGMLEGKLADVKARVEFMKGTRALASLNDMEQTTQQNHSNYFMQGLFGTTSLQMGIIHEKMPISSLNSLYEIDGIPLHYPFDQTDHQGIVELDAKSMAISPMMMPMNNINDTHYTQLGGMSSSNLQCIDPLEKSIYYDPIMLLPSPESVLTGVSCTESSFSMSRMMENEVGKNHHPSPSMAYYGGSSMQTMSPYMQYLKMASGPSQVHAWQMEEYYKANEFQMKNQK

>ColMADS63

MGRKKVEMKRIESKSSRQVTFSKRRGGLVKKARDLSVLCDVDVALLVFSSRGKLYQFCSANNRSLSLFDFCLFSCFWNHCFA

>ColMADS64

MVRGKTQMRRIENATSRQVTFSKRRNGLLKKAFELSVLCDAEVALIIFSPRGKLYEFASPRLKLFS

>ColMADS65

MERKSKGRQKIAMTRISNASNRNVTFSKRRSGLFKKASELCTLCGAEIAIIVFSPGKKAFSFGHPCVDTVVDCFLSRNSLPNSGSLQLVEAHCNANVSKLNLQLNEALDMLEAEKKRGEELNKMRKASRDRCWWEAPVSELGLQQLEQLKVAMEDLKKNVAKQSEKIQMEESNPSRFFEAGSSSMGGRVGGASDGVKVSGLRLSMTPYGYTLDYGHGFF

>ColMADS66

MGRAKQNMELIQNDKTRYVAFQKRKKGLKKKTYELKTLCDVEVCLIIYGPKINDRPTDDQAEIWPPNPDVIQHLIDTYKNQSSEDRTRRTLGLSDFFEDRNQKIEDTLVKLRKKNDEARYSTWDDRYNDLSKEQLREFESVLEGKLQDVKARVAFMKGIDQKLSVACMNDNSHTQTQNQSYFMHGCMFGTEVDNGFANLQYSFNQAQVDANSKANPMMMMTMMPMNNNQYYNQLGGVSSSNFMCNPLDRPIYYDPMMLENVIVNNPSQPVGYCNPSIQTMSPYMQCVKMECASSSVHASQLDK

>ColMADS67

MTIKPTLFPTLFLSTDGLFKKTGELCNLCGTEAAVITFSNAGNTFAFNPSVDSVLDRYLSMTSSSSRASASFSFLFQHKVSTQICAL

>ColMADS68

MGSGRRKIEIKRVVKEGQRIVTFSKRRRGLFNKARQLRCLTGADIAILTFSPAGRPYTHGEPSFDALVDRYLNTAAAREKAEDGCEAAAANHHRLNSWLDALQFDASDSIEDLEILKKGLEEIAAKVAEKIDDVFVDSLLV

>ColMADS69

MGNEKKKIETKRVEKEGQRMVTFSKRRHGLFNKARQLRSLTGADIAILTFSPAGRPYTYGEPSFDALVDRYLNTAAAGEKAEEGCEAAAVNHHRLSSWLDALQFDASDSIEDLEILKKGLEEIAAKVAKKIDDVFVDSLLV

>ColMADS70

MGSGRRKIEIKRVEKEGQRMVTFSKRRRGLFNKARQLRCLTGADIAILTFSPAGRPYTHGEPSFDALVDRYLNTAAAGEKAEEGCEAAAANQHRLNSWLDALQFDASDSIEDLEILKKGLEEIAAKVAEKIDDVFVDSLLV

>ColMADS71

SETFSKARQLRSLTGADIAILTFSPAGRPYTHGEPSFDALVDRYLNTAAAGEKAEEGCEAAAAKHHRLNSWLDALQFDARLEEIAAKVAEKIDDIFVDSLL

>ColMADS72

MERKGKGRQKITMTKMENESNLQVTFSKRRSGLFKKASELSTLCGAEVGIIVFSPGKKAYSFGHPTVDMIIDRFLSRDAPLNSGTHQLVEAHRSACVRDLNLQLTHVQALLDVEKQRGTALDQLKMAEGQGEHRWWEGPVEEMNLPQLHQFKATMMELSTKIGIRADGIVHEQPANPFGFGVGPTSFGGGAGGAGFHQGPSSFGGAGFLAGPSSFGGGGGTSFGGGGGGFLDGQSSFGGGVGGSSFQAGPSFAGGGGIGALPFGARPGSFGAGDGGSDNNGIVPFDGRVLDASGSKAPYGFNHGYGRGFY

>ColMADS73

MGRQKIEIKKIEKKSQLQVTFSKRRAGIFKKAGELGVLCGAHVAVIVTSPAGKVFAFGNPSVDSVIDRFLSKNPNPHGFDSCHDDELHQVHQQSINPNSQCSEPHRVSKNPNLNPNSQCLDMCHEEEVRQVQQLSKKYVKAVGKLEAEKERGKALAELNSGGGGGGFWWEESVEGLELHELEQYVAALEVLKSNLLARADEMAVAASGFPANFLASNGVVVADAFGSAECSGFGFDYKPF

>ColMADS74

MENKQKKHSMGRQKIEIKKIEKKSQLQVTFSKRRAGIFKKAGELGVLCGAHVAVIVTSPAGKVFAFGNPSVDSVIDRFLSKNPNPHGFDSCHDDELHQVHQQSINPNSQCSEPHRVSKNPNLNPNSQCLDMCHEEEVRQVQQLSKKYVKAVGKLEAEKERGKAVAELNSGGGGGGFWWEESVEGLELHELEQYVAALEVLKSNLLARADEMAVAASGFPANFLASNGVVVADAFGSAECSGFGFGFDRKPF

>ColMADS75

MGRQKIEIKKIEKKSQLQVTFSKRRAGIFKKAGELGVLCGAHVAVIVTSPAGKVFAFGNPSVDSVIDRFLSKNPNPHGFDSCHDDELHQVHQQSINPNSQCSEPHRVRKNPNLNPNSQCLDTCHEEEVRQVQQLSKKYVKAVGKLEAEKERGKAVAELNSGGGGGGFWWEESVEGLELHELEQYVAALEVLKSNLLARADEMAVAASGFPANFLASNGVVVADAFGSAECSGFGFGFDRKPF

>ColMADS76

MGRRRVELRPIQDKHKRQITFSKRRQGLMKKAQELSVLCDVDVALAVFTSGCRAYKFSGGNRYALALSFSLINIQILNVRFSDNR

>ColMADS77

MTIIPTLFPTLFLSTDGLFKKTSELCNLCGTEAAVITFSNASNTFAFDNPSVDSVLDRYLSMTSSSSKASVSFSSLFRHRVSTQICAP

>ColMADS78

MGRGKIEIKKIENVNSRQVTFSKRKAGLLKKAKELSILCDAEVGLIIFNNSGKLYEFASPRMEHILARYNNGLESSTLTNPSTEKAVLELEKQPPELDALRGEVAKLQKGYRRTMGKDLEGMSFKELQQLEHQLNEGILSVKDRKEQVLLEQLEKSKLQEKKVKLENETLREQIEELGRPSTTPTPTHQFHCLGRKRSVLSSNTVCDGYFDTERDSETSLRLGLSSEVFHKRKVCKIESVSNDDSGSQMAS

>ColMADS79

MTIIATLFPTLFLSKDSLFKKTGKLCNLCSTEAAVITFSNAGNTFTFGNPSVDSVLDRYLSMTASSSRASTSFS

>ColMADS80

MKRIENPVHRQVTFCKRRAGLLKKAKELSVLCDAKIGVLIFSTNGKLYELATKGTMQGLVGRYMKSTGDTQADHDEEKQVMDSKEEINMLKNEIEFLKKGLRFMSGASFAVAGSATALYHLSNHAPASHKTASPALPS

>ColMADS81

MGRRRVEIKRIEDKARRQTTFTKRRDGLFKKTGELCNLCGTEAAVITFSNAGNTFAFGNPSVDSVLDRYLSMTSSSSRASVDGGEVAAAERVREREEKLAEALARLEVEKKRGEAIDEALSMLKLNEDVVGDSKVTVSEEMGEIGSAVEMAQLEAEKKHDEIVDEMLAMLNAKAEASRLKEVEQEVGKDVDEETTELQIGNKSHRGDR

>ColMADS82

RTCRHICLRRCNVDIGVTLEWNGYENMYVLPLCFDYGDQIFYNILCDTVKERETMHRRRRHGQRKMAKENGERGRRIFTMAKKPSMGHQKIKIAKIEIKNHLQVTFSKRRSGLFKKASELCTLCAIEIAIIVFSPASKVVRELNLQLTQVLNELEAEKRRGETLDHMRKASQRQYWWEAPIDKLGLHELEQLRNSMVELKKKVTNQVNKIQVEGTTNPSPFFLVNGTRMVDHFESKTSHINASSSTTPNVHYFGYYGHGFF

>ColMADS83

MVRGKTQMKRIENATSRQVTFSKRRSGLLKKAFELSVLCDAEVALIIFSPKGRLYEFSNSRCLSISKTIERYQRNTKEQEGSGRKEAEENLQHLKEEAVSMSQKIELIEASKRKLLGDCLESCSINELQLIEEQLEKSLHNIRARKTLLYKEKIEQLKEEEKILTEENQKLREKCEMRSPHLPIPLLVAPEPNREFSDVETELFIGLPEQRTTHSL

>ColMADS84

KTTQGHQKIEIKKIESVSSRQVTFSKRRVRPLQEGQRALHPQLALRLPLSSNLPENALSLSHAPASTPSSIDTSPEPPPLKSRTTALGCAISKSDTPKFPRNWRRRRDGARVRGAGAVCGSIGGIEEECGNES

>ColMADS85

MVRGKTQMKRIEKATRRQVTLSKRRSGLLKKAFELSVLCDAEVALIIFSPKGRLYEFSNSRAAAMGGSTVLWDIFCCNLLIALWHYLCAQV

>ColMADS86

MGSGKRKIEIKRVEKEGQRMVTFSKRCQGLFNKARQLRSLTGADIAILTFSPAGRPYTHGEPFFDALVDRYLNTAVAGEKAEEGCEAAAANHHQLSSWLDALQFNASDSIEDLEILKKGLEEIATKVALKIDDVFVDSLLV

**#nucleotide sequences of MADS-box genes in *Arabidopsis thaliana***

>AT1G01530.1 | Symbols: AGL28 | AGAMOUS-like 28 | chr1:192640-193662 REVERSE LENGTH=744

ATGGCGAGAAAGAATCTTGGTCGTAGAAAAATAGAGTTGGTAAAAATGACCAACGAATCAAACCTTCAAGTTACGTTTTCAAAAAGAAGATCTGGTCTTTTCAAAAAAGGCAGTGAACTTTGCACCTTATGTGACGCGGAAATTGCGATAATCGTGTTTTCACCCTCGGGAAAAGCATACTCTTTTGGTCATCCAAATGTTAACAAGTTACTTGACCACTCTTTAGGGCGTGTTATAAGACACAACAACACAAACTTTGCTGAAAGCCGCACAAAGCTCCGTATTCAAATGCTCAATGAGTCTTTAACTGAGGTGATGGCTGAAAAAGAAAAAGAACAAGAGACCAAGCAGTCAATAGTCCAAAATGAAAGAGAGAACAAGGACGCTGAGAAGTGGTGGAGGAACTCTCCAACAGAACTCAACTTAGCTCAATCAACTTCCATGAAATGTGATCTTGAAGCTTTGAAGAAGGAAGTTGATGAAAAAGTCGCTCAACTTCATCATAGAAACCTAAACTTCTATGTTGGAAGTTCTAGCAATGTTGCTGCTCCAGCAGCTGTTAGTGGTGGTAATATCTCCACAAACCATGGTTTCTTTGATCAAAACGGAAACTCTACTTCTGCTCCAACACTGCCGTTTGGATTTAATGTTATGAATCGCACACCAGCTGGGTACAACAGTTACCAACTCCAAAACCAGGAGGTTAAACAAGTCCATCCTCAGTATTGGGCTCGTTACTATTAG

>AT1G17310.1 | Symbols: AGL100 | agamous-like 100 | chr1:5928014-5928667 REVERSE LENGTH=654

ATGAAAGATTTGTTCATGGAAGGAGAAAGAGAAACTTCATCGATGACTTGCTTGACGCCCAAGGATTCTGTTCAAAGCCCTAACATGTTGGTTAGACAACCAAAAAAGGAGACAACAACACAAACCCCTAAAACGACACGAGGAAGACAGAAGATAGAGATCAAGAAAATCGAGGAAGAGACCAAGAGACAAGTAACGTTCTCGAAACGCCGTCGTGGGCTGTTCAAGAAATCCGCTGAATTGAGCGTTCTCACGGGCGCAAAAATCGCCGTCATAACGTTCTCTAAATGCGATAGGATCTACAGATTTGGCCATGTAGACGCATTGATAGACAAGTATCTTCGTAAGAGTCCGGTGAAGTTGGAGGGATATTCCGGTGATAACGCGGCGGATGAGGAAAGTAGGAGACCGTGGTGGGAGCGTCCGGTGGAGAGTGTGCCAGAAGAGGAGCTTGAGGAGTACATGGCAGCATTGAGTATGTTGAGAGAAAATATAGGGAAAAAGATTGTGGCGATGGGTAACGATCGGACGGTTGATATGGTTCCGGCATGGCCAATCAATGTGATGGGATGGAAACCAACGATGGATATGCAAAAATTGGAAAATCTGACGGATGGGGTTAATCGATGCCGTGTAGGTCAAAACGGTGATTGA

>AT1G18750.1 | Symbols: AGL65 | AGAMOUS-like 65 | chr1:6467266-6469640 FORWARD LENGTH=1170

ATGGGAAGGGTTAAGTTGAAGATTAAAAGACTTGAGAGCACAAGCAACAGGCAAGTTACATACACGAAGAGAAAAAATGGGATTTTGAAGAAAGCCAAAGAGTTATCGATTTTGTGTGATATTGATATTGTCCTTCTTATGTTTTCCCCTACCGGAAGAGCTACTGCTTTCCATGGAGAACACAGTTGCATTGAAGAGGTTATTTCCAAGTTTGCGCAATTAACTCCACAAGAAAGGACAAAAAGGAAACTGGAGAGCCTTGAAGCATTGAAGAAAACTTTTAAGAAACTGGATCATGATGTAAATATACATGACTTTTTAGGAGCAAGGAATCAAACTATTGAGGGTCTAAGTAACCAAGTAGCCATTTACCAAGCTCAGCTAATGGAGTGTCATAGGAGGTTGAGTTGTTGGACGAACATCGATAGAATAGAAAACACTGAGCACCTCGATTTATTGGAAGAATCATTGAGGAAATCCATTGAAAGAATCCAGATTCACAAGGAACATTACAGAAAGAACCAACTCTTGCCAATAGAATGTGCAACAACACAGTTTCACAGCGGGATACAGTTGCCTATGGCGATGGGAGGTAATAGTAGTATGCAAGAAGCTCACTCCATGTCTTGGCTTCCTGATAATGATCACCAGCAAACAATCTTACCTGGTGATTCCAGTTTTCTTCCCCATAGAGAGATGGATGGTTCGATTCCCGTTTACTCAAGCTGCTTCTTTGAGTCTACGAAACCAGAAGATCAGATATGCAGCAACCCGGGACAACAGTTTGAGCAGTTAGAACAACAAGGAAACGGTTGTTTGGGGTTACAACAACTTGGAGAGGAATATTCATATCCTACACCGTTTGGTACTACTTTGGGAATGGAAGAAGATCAAGAGAAAAAGATAAAATCTGAAATGGAATTGAACAACTTGCAACAACAGCAACAGCAACAACAACAACAACAACAACAAGATCCTTCAATGTATGATCCCATGGCTAATAATAATGGTGGCTGCTTTCAGATTCCTCATGATCAGTCCATGTTTGTCAATGATCATCATCATCATCATCACCACCATCATCAAAATTGGGTTCCAGATTCAATGTTTGGTCAGACTTCTTACAACCAGGTTTGTGTGTTCACACCTCCATTGGAACTATCTAGGTAG

>AT1G22130.1 | Symbols: AGL104 | AGAMOUS-like 104 | chr1:7812387-7814259 REVERSE LENGTH=1008

ATGGGTCGGGTGAAATTAGAAATCAAGAGAATAGAGAACACAACGAATCGACAAGTTACGTTTTCAAAACGTAGAAATGGTTTGATTAAGAAAGCTTATGAATTGTCGATTCTTTGTGATATCGACATTGCTCTTATCATGTTCTCTCCTTCTGATCGCCTTAGCCTTTTCTCCGGAAAAACTCGGATTGAAGACGTTTTTTCAAGATTCATCAATCTTCCTAAACAAGAACGAGAGAGTGCTCTATACTTTCCTGACCAGAATAGACGCCCAGATATTCAAAACAAAGAGTGTCTACTAAGGATTCTGCAGCAACTCAAGACTGAAAATGACATCGCTCTTCAAGTCACGAACCCTGCAGCTATCAACTCTGACGTCGAGGAACTTGAGCATGAAGTTTGTAGGTTACAACAACAACTCCAAATGGCAGAAGAAGAACTAAGGAGATACGAACCGGATCCAATAAGATTCACGACCATGGAGGAATACGAAGTTTCTGAGAAGCAACTTCTCGACACTCTAACACATGTTGTCCAACGGCGAGACCATCTCATGAGCAACCATTTATCTTCGTATGAAGCATCTACTATGCAACCAAACATTGGTGGTCCTTTTGTAAATGACGTCGTTGAAGGTTGGCTACCTGAAAATGGGACCAATCAAACCCATTTGTTTGATGCATCGGCCCATTCTAACCAACTCAGAGAATTATCATCGGCAATGTATGAACCATTGTTGCAAGGAAGTAGCTCAAGCTCGAACCAAAACAACATGAGTGAATGCCACGTGACGAACCACAATGGTGAAATGTTCCCTGAGTGGGCTCAGGCGTATTCATCCTCGGCCTTATTCGCTTCTATGCAGCAACAGCATGAAGGTGTGGGTCCCAGTATAGAAGAGATGATGCCAGCTCAGCAGAGTGATATTCCGGGAGTGACGGCGGAGACACAAGTTGATCATGAAGTCTCCGACTATGAAACAAAGGTTCCTCAACTCAGTAGCCAATAA

>AT1G22590.2 | Symbols: AGL87 | AGAMOUS-like 87 | chr1:7983511-7984002 FORWARD LENGTH=492

ATGGGAAGAAGAAAAGTTACACACCAATTGATTTCTGACAACGCCACTCGCCGAGTTACATTCAGAAAACGCAAAGATGGTTTATTGAAGAAGATCTATGAACTGACCGTTCTATGTGGTTTACCCGCTTGTGCCATCATCTACAGCGAATACAAAGATGGCCCTGAGCTCTGGCCAAACCTTAACGAGGTTCGTTCTATTCTTAACAGGTTAAGCGAACTTCCGGTAGAGAAGCAGACCAAATACATGATGGACCAGAAGGATCTCATGAACAAGATGATCCAAGACGCAGAGAAGAAGTTGGAGAAGGAGAAGATGCATACTCGTGCGATGAAACTTGGGTTAATGGCTGGGTCTAATGATCTAATCACCGATACTGATTGTTCGGAGGAACTGGCCAGAGCAGCTGATGTGGTTGATAAGAAGCTCAAAGCCATCAGAGAGAGAATCAAGGCTGTTGAAGCAGGAGCACCAATCATCAAGAGAGACTAG

>AT1G24260.1 | Symbols: AGL9, SEP3 | SEPALLATA3, AGAMOUS-like 9 | chr1:8593790-8595862 REVERSE LENGTH=753

ATGGGAAGAGGGAGAGTAGAATTGAAGAGGATAGAGAACAAGATCAATAGGCAAGTGACGTTTGCAAAGAGAAGGAATGGTCTTTTGAAGAAAGCATACGAGCTTTCAGTTCTATGTGATGCAGAAGTTGCTCTCATCATCTTCTCAAATAGAGGAAAGCTGTACGAGTTTTGCAGTAGTTCGAGCATGCTTCGGACACTGGAGAGGTACCAAAAGTGTAACTATGGAGCACCAGAACCCAATGTGCCTTCAAGAGAGGCCTTAGCAGAACTTAGTAGCCAGCAGGAGTATCTCAAGCTTAAGGAGCGTTATGACGCCTTACAGAGAACCCAAAGGAATCTGTTGGGAGAAGATCTTGGACCTCTAAGTACAAAGGAGCTTGAGTCACTTGAGAGACAGCTTGATTCTTCCTTGAAGCAGATCAGAGCTCTCAGGACACAGTTTATGCTTGACCAGCTCAACGATCTTCAGAGTAAGGAACGCATGCTGACTGAGACAAATAAAACTCTAAGACTAAGGTTAGCTGATGGGTATCAGATGCCACTCCAGCTGAACCCTAACCAAGAAGAGGTTGATCACTACGGTCGTCATCATCATCAACAACAACAACACTCCCAAGCTTTCTTCCAGCCTTTGGAATGTGAACCCATTCTTCAGATCGGGTATCAGGGGCAACAAGATGGAATGGGAGCAGGACCAAGTGTGAATAATTACATGTTGGGTTGGTTACCTTATGACACCAACTCTATTTGA

>AT1G26310.1 | Symbols: CAL1, AGL10, CAL | AGAMOUS-like 10, CAULIFLOWER | chr1:9100330-9103510 REVERSE LENGTH=768

ATGGGAAGGGGTAGGGTTGAATTGAAGAGGATAGAGAACAAGATCAATAGACAAGTGACATTCTCGAAAAGAAGAACTGGTCTTTTGAAGAAAGCTCAGGAGATCTCTGTTCTTTGTGATGCCGAGGTTTCCCTTATTGTCTTCTCCCATAAGGGCAAATTGTTCGAGTACTCCTCTGAATCTTGCATGGAGAAGGTACTAGAACGCTACGAGAGGTATTCTTACGCCGAGAGACAGCTGATTGCACCTGACTCTCACGTTAATGCACAGACGAACTGGTCAATGGAGTATAGCAGGCTTAAGGCCAAGATTGAGCTTTTGGAGAGAAACCAAAGGCATTATCTGGGAGAAGAGTTGGAACCAATGAGCCTCAAGGATCTCCAAAATCTGGAGCAGCAGCTTGAGACTGCTCTTAAGCACATTCGCTCCAGAAAAAATCAACTCATGAATGAGTCCCTCAACCACCTCCAAAGAAAGGAGAAGGAGATACAGGAGGAAAACAGCATGCTTACCAAACAGATAAAGGAGAGGGAAAACATCCTAAGGACAAAACAAACCCAATGTGAGCAGCTGAACCGCAGCGTCGACGATGTACCACAGCCACAACCATTTCAACACCCCCATCTTTACATGATCGCTCATCAGACTTCTCCTTTCCTAAATATGGGTGGTTTGTACCAAGAAGAAGACCAAACGGCGATGAGGAGGAACAATCTGGATCTGACTCTTGAACCCATTTACAATTACCTTGGCTGTTACGCCGCTTGA

>AT1G28450.1 | Symbols: AGL58 | AGAMOUS-like 58 | chr1:10003966-10004523 FORWARD LENGTH=558

ATGAATCCCAAGAAAACCAAAGGAAAACAAAAGATTAACATCAAGAAAATTGAAAAAGACGAAGACAGATCGGTCACATTGTCTAAGCGTCTAAATGCTATCTACACTATGATCATTGAGCTTTCCATTCTCTGTGGTGTTGAAGTTGCGTTTATCGGGTATTCTTGCTCTGGAAAACCATACACATTCGGCAGTCCGTCCTTCCAAGCCGTGGTGGAGCGGTTTCTCAACGGCGAGGCCTCGTCGTCTTCTTCATCATCGTTGCAACGATCGGTCAAGAATGCTCACAAGCAAGCGAAGATTCAAGAGCTTTGCAAAAGATACAATAGATTGGTGGAGGAGTTAAAGGTGGACGAAGTCAAAGTCAAGAAGGCAGCTGCATTGGCGGAGACGAGGGCCGTGAATAAGGATGCGTGGTGGAAAGCAGATCCAAATGACGTGAAGGATCACGAAAAGGCGAAGAAGATGATGGAGAAGTATCAAGAGCTCAAAGAGAAACTGCGTGAGGAAGTTGCTTTAAGGATCAAGAGAGGACATGATGAGAATAATAACAAATGA

>AT1G28460.1 | Symbols: AGL59 | AGAMOUS-like 59 | chr1:10006230-10006778 FORWARD LENGTH=549

ATGAATCCCAAGAAAACCAAAGGAAAACAAAAGATTAACATCAAGAAAATTGAAAAAGATGAAGGCAGATCGGTCACATTCTCTAAGCGTCTAAATGGTATCTACACTAAAATCAGTGAGCTTTCTATTCTCTGTGGTGTTGAAGTTGCGTTTATCGGGTATTCTTGCTCTGGAAAACCATACACTTTTGGCAGTCCATCCTTCCAAGCCGTGGCGGAGCGGTTTCTCAACGGCGATGCCTCGTCGTCTTCTTCATCATCATTGGTCATGAATGCTCACAAGCAAGCGAAGATTCAAGAGCTTTGCAAAAAATACAATAGATTGGTGGAGGAGTTAAAGGTGGACGAAGTCAAAGTCAAGAAGGCAGCTGCATTGGCGGAGACGAGGGTCGTGAATAAGGATGTGTGGTGGAAAGTGGATCCAAATGACGTGAAGGATCACGAAAAGGCGAAGAAGATGATGGAGAAGTATCAAGAGCTCTATGATAAACTGTGTGAGCAAGCTGCTTCAAGGATCAAGAGAGGACATGATGAGAATAATAACAAATGA

>AT1G29962.1 | Symbols: AGL64 | AGAMOUS-like 64 | chr1:10496730-10497287 FORWARD LENGTH=558

ATGAAACCCAAGAAAACCAAAGGAAAACAAAGGATTAACATCAAGAAAATTGAAAAAGACGAAGACAGATTGGTCACATTGTCTAAGCGTCGAAATGGTATCTACACTAAACTCAGTGAGCTTTCCATTCTCTGTGGTGCTGAAGTTGCGTTTCTCGGGTATTCTTGCTCTGGAAAACCATACACATTCGGCAGTCCGTCCTTCCAAGCCGTGGCAGAGCGGTTTCTCAACGGCGAGGCCTCGTCGTCTTCTTCATCATCGTTGCAACGATCGGTCATGAATGCTCATCAGCAAGCGAAGATTCAAGAGCTTTGCAAAGTATACAATAGATTGGTGGAGGAGATAACGGTGGAAGAAGTCAAATTAAAGAAGACGGCTGCATTGGCGGAGATGATGCCCATGAATGAGGATGCGTGGTGGAAAGTGGATCCAAATGACGTGAAGGATCGCGAAGAGGTGAAGAAGATGATGGAGAAGCATCAAGAGCTCTATGAGAAACTGTGCGAGGAAGCTGCTTCAAGGATCAAGAGAGGACATGATGAGAATAATAACAAATGA

>AT1G31140.1 | Symbols: GOA, AGL63 | AGAMOUS-like 63, GORDITA | chr1:11118031-11119673 FORWARD LENGTH=642

ATGAGGAAAGGTAAGAGAGTGATAAAAAAGATAGAGGAGAAAATAAAGAGACAAGTGACATTCGCAAAGAGAAAGAAGAGTCTAATCAAGAAGGCATATGAACTCTCTGTTCTCTGCGATGTCCACCTTGGTCTCATCATCTTCTCTCACTCCAACAGGCTCTACGATTTCTGCTCCAACTCTACCAGCATGGAGAATCTCATCATGAGATACCAAAAGGAAAAAGAAGGTCAAACCACTGCAGAACACAGTTTCCACTCGTGTTCAGATTGCGTGAAGACGAAGGAATCAATGATGAGAGAGATAGAGAATCTTAAGCTGAATCTTCAATTGTACGACGGACATGGCTTGAATCTCTTGACCTACGACGAGCTCCTTTCTTTTGAGCTCCATCTCGAATCTTCTCTACAACATGCTCGAGCTCGCAAGTCTGAGTTCATGCATCAGCAGCAGCAGCAACAAACAGATCAAAAGCTTAAGGGAAAAGAAAAGGGTCAAGGAAGCTCTTGGGAGCAGCTGATGTGGCAAGCAGAGAGACAGATGATGACGTGTCAAAGACAAAAAGATCCTGCGCCGGCGAATGAAGGAGGAGTTCCTTTTTTACGGTGGGGAACAACCCACCGACGTTCTTCACCTCCTTAA

>AT1G31630.1 | Symbols: AGL86 | AGAMOUS-like 86 | chr1:11318528-11319547 REVERSE LENGTH=1020

ATGAGGTCGAAAATTAAGTTATCACTCATAGCTAATAAGACCTCAAGGAGAACCACATTCAGGAAGAGGAAGGGAGGGATAACGAACAAACTCCATGAGCTAACAACTCTCTGCGGCGTCAAAGCATGTGCGGTAATCTCCAGTCCGTACGAGAATCCAGTGGTGTGGCCGTCAACCGAAGGTGTTCAAGAGGCGGTTTCCATGTTTATGGAGAGGCCGGCGACAGAACAATCCAAGCTGATGATGAGTCATGAGACCTACTTGCAGGACAAAATTACCAAAGAAACAAAGAAACTGGAGAGTCTACGTCGTGAAAACCGAGAATCTCAGCTTAGGCAATTTATGTTTGATTGTGTTGAAGGTAAGATGAGTGAGCATCAGTATGGTGCAAGGGACCTTCAAGATTTAAGTCTTTATATTGATCACTATATCAATCAGCTTAATTCAAGTGTCATGCTCCTTACAAACAATGGTGCGTCTTCTTCTTCCTTTCCTCCTCCGCTTCATACTTCAGTTGCGGGTGCGGGTGCGGGTGCGGGTGCTGCTCCTCTTGTTGTTGCGGGTGCGGGTGCGGCTCCTCTTGCTGTTGCGGGGGCGGGTGCGAGTCCTCTTGCTGTTGCGGGTGTGGGTGCGGCTCCTCTTGCTGTTGCGGGTGCCGGTCCTCCTATGGCTCAGAATCAGTATGAGCCGATTCAGCCCTATATCCCTACTGCTTTTAGTGATAATATTCAATACCAAGCTCCTGTGGATTTTAATCATCAGATCCAACATGGAATCTATGATAATCTCAGTTTGGATCCAAATCATCAGTATCCGTTTCAAGATGATCCATTCATGGAGATGTTGATGGAATATCCTTATGAACAAGTGGGTTATGCTGCAGAGCATGCACACATCCCTTTCATGAACGGAAACTACTACAACTACCACCAACCACCAACCGTTGGTCTTACTACCACCGGTCACATGCCTTCCAACAACGCCACCACCACCACCACCACCAACACCACCGTTGTGTGA

>AT1G31640.1 | Symbols: AGL92 | AGAMOUS-like 92 | chr1:11322692-11324176 REVERSE LENGTH=1395

ATGAGGACGAAGACTAAGTTAGTACTCATACCTGATAGACACTTTCGGAGAGCCACATTCAGGAAGAGGAATGCAGGGATAAGGAAGAAACTCCACGAGCTGACAACTCTCTGTGACATCAAAGCATGTGCGGTAATCTACAGTCCGTTCGAGAATCCAACGGTGTGGCCGTCAACCGAAGGTGTTCAAGAGGTGATTTCGGAGTTCATGGAGAAGCCGGCGACAGAACGGTCCAAGACGATGATGAGTCATGAGACTTTCTTGCGGGACCAAATCACCAAAGAACAAAACAAACTAGAGAGTCTACGTCGTGAAAACCGAGAAACTCAGCTTAAGCATTTTATGTTTGATTGCGTTGGAGGCAAGATGAGTGAGCAACAGTATGGTGCAAGGGACCTTCAAGATTTAAGTCTTTTTACTGATCAATATCTTAATCAGCTTAATGCCAGGAAGAAGTTCCTTACAGAATATGGTGAGTCTTCTTCTTCTGTTCCTCCTCTGTTTGATGTTGCGGGTGCCAATCCTCCTGTTGTTGCAGATCAAGCTGCGGTAACTGTTCCTCCTTTGTTTGCTGTTGCGGGTGCCAATCTTCCTGTTGTTGCTGATCAAGCTGCGGTAACTGTTCCTCCTCTGTTTGCTGTTGCGGGTGCCAATCTTCCTGTTGTTGCAGATCAAGCTGCGGTTAATGTTCCTACTGGATTTCATAACATGAATGTGAACCAGAATCAGTATGAGCCGGTTCAGCCCTATGTCCCTACTGGTTTTAGTGATCATATTCAATATCAGAATATGAACTTCAATCAAAACCAACAAGAGCCGGTTCATTACCAGGCTCTTGCTGTTGCGGGTGCCGGTCTTCCTATGACTCAGAATCAGTATGAGCCCGTTCACTACCAGAGTCTTGCTGTCGCGGGTGGCGGTCTTCCTATGAGTCAGTTGCAGTATGAGCCGGTTCAGCCTTATATCCCTACTGTTTTTAGTGATAATGTTCAATATCAGCATATGAATTTGTATCAAAATCAACAAGAGCCGGTTCACTACCAAGCTCTTGGTGTTGCAGGTGCCGGTCTTCCTATGAATCAGAATCAGTATGAGCCGGTTCAGCCCTATGTCCCTACTGGTTTTAGTGATCATTTTCAGTTTGAGAATATGAATTTGAATCAAAATCAACAGGAGCCGGTTCAATACCAAGCTCCTGTTGATTTTAATCATCAGATTCAACAAGGAAACTATGATATGAATTTGAACCAGAATATGAAGCATGCACACATCCCTTTCATGGACGGAAACTACTACAACTACCACCAGCCACCAACCGTTGGTCTTACTTCCACCGGTCACATGCCTTCCACCACCACCACCACCACCAACAACAACAACAACAACAATGTGTGA

>AT1G33070.1 | Symbols: no symbol available | no full name available | chr1:11982889-11985299 FORWARD LENGTH=498

ATGCCCATGAGGAAGGAAGGGATAACGAAGAAACTTTATGAGTTGGCAACTCTCTGCGACATCAAAGCATGTGCGGGTAAGATAAGTGAGCATCAGTATGGTGCAAGGGACCTTCAAGATTTAAGTTTTCATATTGATCACTATATCAATCAGCTTAATTCCAGGGTCAAGATCCTTACAAACAATGGTGAGTCTTCTTCTTCCGTTCCTCCTCTACTTCATACTTCAGTTGCGGGTGCGGGTGCAGCTCCTCTTCCTGTTGCGGGTGCCGATCTTCCGATGGATCAGAATCAGTATGAGCCGATTCAGCTCTATATCCCTATTGGTCTTAGTTATCATATTCAATACCAACATGAAATCTATGATAATTTCAAGCATGCGCACATCCTTTTCATGAACGGAAACTACTACAACTACCACCAAACACCAACCGTTGGTCTTACTACCACCGGTCACATGCCTTCCAACAACAACACCACCCCCACCACCGATGTGTGA

>AT1G46408.1 | Symbols: AGL97 | AGAMOUS-like 97 | chr1:17232135-17232935 REVERSE LENGTH=801

ATGGGTGGCGTGAAGAGGAAGATTGCTATAGAGAAGATACAGAACAAAAATCCACGAGCGGTTTCATTCTCCAAACGTCGTAAGGGTCTGTATAGCAAAGCTTCTGAACTTTGTCTTCTCTCGGACGCAGAGATTGCGATCATAGCGACTCCTGTTTCTTCTAATTCCAACGCCGCTTTCTACAGTTTTGGCCACTCCTCTGTTGATAATGTCGTCGCTGCTTTTCTCGCCAATCAGCGTCCTTGTGATGAAAGGTTTTGGTGGGAAGATGAGAGTCTTCTAAAATCAGAGAATCTGGAGGAGTTGAGAGAGGCGATGGATTCAATGTCGACTATGTTGCGAGATCTCAAGGAGTTGGAGAAGCAAAGAGATCATCAAACGCAAACCCTAATTCATCAGCCGTGTTCTGCAAGGGTTTGCATTCAAGATTATGTAACTGTGAATTTCGATGGGTTTAACACGGAAGAGCAAACCCTAGCGGTTTCTGACAATAGTAACAACAACGGTTTACTTGGAAACTTGGATGAGTGCAATGAAGATTTTGATGATCTCGATCAAATCTTCGATACGGTGACAAACTCTGAATTTTTATCGGTGAATTTGGAGATGGATGATGTAACTGTGAATTCTGAAGGGAACACGGAAGAGCAAACCCTAGCGGTTTCTGACAATAGCAACAACAACGGTTTACTTGGAAACTTGGATGAGTGCAATGAAGATTTTGATGATCTCGATCAAATCATTGAGTATTTGACAAGCTCTGAAGCTTTATCCATGAATTTAAAGATGGATGATGTCTGA

>AT1G47760.1 | Symbols: AGL102 | AGAMOUS-like 102 | chr1:17572451-17573159 FORWARD LENGTH=555

ATGGGTCGTAGAAAAATAGAGATAAAATTTATTGAAGATAGCATCGAAAGGAAAGCGACGTTTTCAAGACGCCGAAATGGCATTTTTAAGAAGGCTGATGAACTCGCGAAGTTGTGTAATGTAGAGATTGCTGTCTTGGTCATCTCCCCCACTAATATACCATACACATACGGTTATCCATGCTTCAACGATGTGGTTGAGCGTATTCAAAATCCTAGTGCTTCGTCCAAACTCAGGAGTCTCATGAAAGAATTGGAACAAATCAAAGAGTTTCAGGAGGATTTGAGGAAGAAACAGCAAAGAAACCTTGAAAAGTCCAATATGAAAGAGAATGTAGATCTGAAATTGGAGGACTTAGTTGCTTTCAAGGCGAAACTCGAGGCCTATCAAGCAGGTTTAAAGAGGAAACATGTAGAGATGGAGGATTTGTCTTCTCCATCGATACTCTCAAAGAATACAAAGAATAAGATGATGAGGACCGAGTATTCGTCTGGACAGAGCAAAGGAATGTATGAGTTTCGGGCTTTCGGCCCAGGATTTCTTGGAACTATTTAG

>AT1G48150.1 | Symbols: AGL74 | agamous-like 74 | chr1:17785397-17786368 FORWARD LENGTH=972

ATGCAGTCGTCGAACGTAACTGATTGCACAATGAGGAAAAGAGGAACAAAAAGAAAGATTGAAATTGAGAAACGCATGACTAAACAACAACGATCCGTCGCTTGCAGCAAACGCCGTCCCACACTCTTCTCCAAAGCGGCTGATCTTTGCCTTCTCTCCGGAGCCAACATAGCCGTTTTTGTAACTTCTCCTGACGAGAACTCCGATGTCGTTTATTCATTCTCGGGCTACTCTCATGCCTCTGAAATTGTTGACTGTTACCTCAACAACAAGTCTCCTCCCAAGACTACTATTAACCCTGAATCAGCCAAGTTTTGGTGGGAAGAGCCCGATCTTTACCGTGATTGTGACGATCTGTCTGAGTTAAGAATCATCGAAGATCGTTTGATGAGAACGAAGAAGCATCTCATGGATTATCTTGAGAAGAAAGAAAAATCACACTCTGTTTCTAAATCCGATCAAAACCCTAACAACGACAGTGGGTCTTCTTCTTCGTCTTCTCAAATTGCTTCTGATTTTGGTCAAAACCCTAGTACCTTGTCTCCGTCTTCCTTAAAAATTGTTTCTTTTGATCAGAACTCTTACTCCTCACTTGAGCCTTCGTCACAAGTTACTACTTGTTTTGATCAAAACCCTGTTTTCTCTGTTGGCAGCGAGTCATCATCTGATCAATCTCGTTACTTGGTGAATGAAGATTCTGGATTTGTTGACGGCCTCTTATGTGAAACAGAGGAAGAGAATAACGGAATGCGTCTTCCCCAAGAAACACAAACACAACCGATGTTCACAGAGGAGGATCAGAGCTTTTGGGAAAACCTAGATGTCGACGACGTGTTTGGTCTTTTTAAGGACGACAACAATCTTGAGGTTCCTCTTCAAGATCATTCCTCAACCAACGAAGACGACGAGTTATTGATCGACATAAGTGAATACTTAAGCGAAGAAGCAATGGAATGCCCATGTTTTAGTTAG

>AT1G54760.1 | Symbols: AGL85 | AGAMOUS-like 85 | chr1:20433912-20434397 FORWARD LENGTH=486

ATGAAGACAGATTGGTCACATTATCTAAGCGTCGAAATGGAATCTACAATAAGCAATGAGCTTTCCATTCTCTGTGGTGCTGAAGTTGCGTTTCTCGGATACTCTTGCTCAGGAAAACCATACACATTCGGCAGTCCGTCTTTTCAAGCCGTGGCAGAGCGATTTCTCAATCGTGAGGCCTCATCATCGTTGCAACGATCGGTCATGAATGCTCACCAACAAGCGAAGATTCAAGAGCTTTGCAAAGTATACAATAGAATGGTGGAGGAGGCAAAGACGGAAGAAGCCAAAGTAAAGAAGGCAGCTGCATTAGCGGAGACAATGCCCGTAGATGAGGATGCGTGGTGGAAAGTGGATCCAAAGGAGGTGGAAGATCACGAAGAGGCGAAGAAGATAATGGAAAAGTGTGAAGGGCTCTATGAGAAACTGTGTAATGAAGCTGCTGCAAGGATCCAAAGAGGAGATGCTGAGAATAATAACAAATGA

>AT1G59810.1 | Symbols: AGL50 | AGAMOUS-like 50 | chr1:22008604-22009455 FORWARD LENGTH=852

ATGGCTCCTCGTCAGAAGAAACCTAACAAGTCTGATGATGATGATGATTTGCGTAGGAAGAAACAGAGCTTTTTCAAACAGAGATTTCCAGGCTTTAAGAAGAAAGCCTCTGAGCTCTCTGTTCTCTGCGGCAACTCTGTCGGTTTCATCTGTTACGGTCCCGACAGCGATCTTCATGTTTGGCCTCAGTCTCAAGATCATAACCCACAAGCCCTACATGAGATCGTTGCCAAGTTCAATGCCTTGAGTGATGAGAGGAGGAAGAATCATGCATGTGATCTCAATGACTTCCCTCATCATCTCAAGGGTTTGTCTCGTGAGGAGTTAAGGAAGCATCTCCTTCACCTTGACTCTCAATTACTTGGAGTCAGGGAACAAAAGATAGAAATTCTTAAGAAGACGCTCACGGGTTCTTCCGAAAAAGATGGTGCTAGGGTTTCAGAGAACTCGGCCATCTCCGATCACAAGTTGAAGATAGAGCCTCATTTGAAGGATATATTGTCGGAAGATCATCTAATTAGGGTTTCAGATAAGAAGCTGGGTTCGTGTGATGTATTTGACGAGTTGGCTTACGTGGTCCGCGGGTCAAGGAATTTGAACGAGAATGTTTCCAAGTACGAATCAAAAGATGCAGATAACACGGGACTGGATCATCTTGTAACTTTAGGCGGTGATTATCTTCAGGAGGCGGCAGCAGAACTTTACCAAACTTACAATCTAGGGAATTTTTGTGATGATCATGTTTGGGATCTGGAGTTTGCCTCAAGACTACCACTACTTCATACTTTTAGCGATCCTCTCATGACCACCAATACTTGCCAAACGATGAGCACCGATATGATTTCAATTTGA

>AT1G60040.1 | Symbols: AGL49 | AGAMOUS-like 49 | chr1:22119075-22119929 REVERSE LENGTH=855

ATGGCTCCTCGTCAGAAGAAACCTAACAAGTCCGATGATGATGATGGTGATTTGCATAGGAAGAAACAGAGCTTTTTCAAACAGAGATTTCCAGGCTTTAAGAAGAAAGCCTCAGAGCTCTCTGTTCTCTGCGGCAACTCTGTCGGTTTCATCTGTTACGGTCCCGACAACGATCTCCATGTTTGGCCTCAGTCTCAAGATCATAACCCACAAGCCCTACATGAGATCGTGGCCAAGTTCAATGCCTTGAGTGATGAGAGGAGGAAGAATCATGCATGTGATCTCAATGACTTCCCTCATCATCTCAAGGGTTTGTCTCGTGAGGAGTTAAGGAAGCATCTCCTTCACCTTGACTCTCAATTACTTGGAGTCAGGGAACAAAAGATAGAAATTCTTAAGAAGACGCTCACGGGTTCTTCCGAAAAAGATGGTGCAAGGGTTTCAGAGAACTCGGCCATCTCCGATCACAAGTTGAAGATAGAGCCTAATTTGACGGATATATTGTCAGAAGATCATCTAATTAGGGTTTCAGATAAGAAGCTGGGTTCGTGTGATGTATTTGACGAGTTGGCTTACGTGGTCCGCGGGTCAAGGAATTTGAACGAGAATGTTTCCAATTACGAATCAAAAGATGCAGCTTACACGGGAATGGATCATCTTGGCACTTTCGGCGGTAATTATCTTCAGGAGGCGGCGGCAGAACTTTACCAAACTTACAATCTAGGGAATTTTTGTGATGATCATGTTTGGGATCTGGAGTTTGCCTCAAGACTACCACCACTTCATACATTTAGCGATCCTCTCATGACCACCAATACTTGCCAAACGATGAGCTCCGATATGATTTCAATTTGA

>AT1G60880.1 | Symbols: AGL56 | AGAMOUS-like-56 | chr1:22411575-22412180 FORWARD LENGTH=606

ATGGGAGGCAAAAAAACAAAGATTGAGATTAAGAAGATCATAAACAAACCCGCAAAAACGGTTGCTTTCACAAAACGCAGGGAAGGTCTCTTCCGCAAAGCTTCACAGCTTTGTCTCCTCTCTCCAGCCACTCAAATCGCAATCTTAGCGGCTCCTATGACTTCCAAATCTCACGCTTCTTTCTACTCTTTCGGTCATTCCTCTGTCGATAACGTTGTCTCCTCTCTGCTTTATGACCATCCTCCTCTTACGGCAAACCAAGACAACAGGTCAGGGTTAGGGTTTTGGTGGGAAGACAAACGTTTTGACGTATCGGAGAACGTTGAGGAGTTAAAAGAGGCGGTCGATGCTGTTTCGAGGATGTTGAACAATGTGAGATGCCGATTAAATGATGCCGTGAAGAGTACTCAAAGAGATGGAGGTTTAGAGATTCTTCATCATCAGGAGGAGGAAGTTCTTCAGACTCGCAACGACGAGACAAAGACGAATCAAACTCACGAATTTGAAGGCGGCGAAACTTCTGGTTCTGCAAGTTGGTTAGAGAACGAGGATGATATTCTTCACTTTGATGATGATTTTTATACTGGTATTGATCCTTTGTTTTGA

>AT1G60920.1 | Symbols: AGL55 | AGAMOUS-like 55 | chr1:22429692-22430267 REVERSE LENGTH=576

ATGGGAGGCACAAAAAGAAAGATAGAGATGAAGAGAATCGAAGATAAGAACGTGAGAGCAGTTGCTTTCACAAAACGCAAAAGCGGACTCTTCCACAAAGCTTCAGAGCTCTGTCTCCTCTCTCCGGGGACTCAAATCGCAATCTTAGCAACTCCTCTTTCTTCCCACTCCCATGCTTCTTTCTACTCTTTCGGTCATTCCTCTGTCGATCACGTTGTCTCCTCTCTCCTTCACAATCAGCATCCATCTCTTCCCACAAACCAAGATAACAGATCAGGGTTAGGGTTTTGGTGGGAAGACCAAGCCTTTGACAGATTGGAGAACGTCGACGAGTTGAAAGAGGCAGTCGATGCGGTTTCGAGGATGTTGAACAATGTGAGGCTACGATTAGATGATGCCGTGAAGAGCAATCAAAGAGATGGAAGTTTAGTGATTCATCAGGAGGATGAGGAGGTTCTTCAGCTTGGCTACAAAGACACAAATCAAATTACCAAACTCGAAGGTGAAACTTCCGCTTCTGCAAGTTTGTTAAAGAACGTAGTGGATAATCTACACATTGATGATCGTTATTACTGA

>AT1G65300.1 | Symbols: PHE2, AGL38 | PHERES2, AGAMOUS-like 38 | chr1:24254929-24255765 FORWARD LENGTH=837

ATGAAGAGAAAGATGAAGTTATCGTTAATAGAAAACAGTGTATCGAGGAAAACAACATTCACCAAAAGGAAGAAAGGGATGACGAAGAAACTAACCGAGCTAGTCACTCTATGTGGTGTTGAAGCATGTGCGGTCGTCTATAGTCCGTTCAACTCGATCCCGGAGGCTTGGCCGTCAAGGGAAGGCGTTGAAGACGTGGTGTCGAAATTTATGGAGTTGTCGGTGTTGGACCGGACCAAGAAGATGGTGGATCAAGAGACTTTTATAAGTCAAAGGATCGCCAAAGAAAAAGAGCAGCTGCAGAAGCTACGTGATGAGAACCATAATTCTCAGATTCGGGAGTTAATGTTTGGTTGTCTCAAAGGGGAGACGAATGTGTATAATCTTGATGGAAGGGATCTTCAAGATTTGAGTTTATATATTGATAAGTATCTTAATGGTCTTACTCGCAGGATTGAGATCCTTATTGAGAACGGTGAGTCTTCTTCATCTTTACCTCTTCCTATTGTTGCGAATGCAGCTGCACCAGTCGGATTTGATGGTCCTATGTTTCAATATCATAATCAAAATCAGCAAAAGCCGGTTCAATTCCAATATCAGGCTCTTTATGATTTTTATGATCAGATTCCAAAGAAAATTCATGGTTTTAATATGAATATGAATAAGGATTCGAATCAAAGTATGGTTTTGGATTTGAATCAAAATCTTAATGATGGAGAGGACGAGGGCATTCCTTGCATGGACAACAACAACTACCACCCCGAAATCGATTGTCTCGCTACCGTCACCACTGCCCCCACTGATGTTTGTGCTCCTAACATCACCAATGATCTCTAG

>AT1G65330.1 | Symbols: PHE1, AGL37 | AGAMOUS-like 37, PHERES1 | chr1:24266481-24267320 REVERSE LENGTH=840

ATGAGGGGGAAGATGAAGTTATCGTTCATAGAAAATGATTCAGTGAGGAAAACAACATTCACCAAAAGGAAGAAAGGGATGCTGAAGAAATTCAACGAGCTAGTAACTCTATGTGGTGTTGACGCATGTGCGGTCATCCGTAGCCCGTACAACTCGATCCAGGAGCCTTGGCCATCAAGGGAAGGCGTTGAAGAAGTGATGTCGAAGTTTATGGAGTTTTCGGTGTTGGACCGGACCAAGAAGATGGTGGATCAAGAGACGTTTTTACGTCAAAGGATCGCCAAAGAAACAGAACGTCTCCAGAAGCTACGTGATGAGAACCGTAATTCTCAGATTCGAGATTTAATGTTTGGTTGTCTCAAAGGAGAGGTGGACGTGTCTCATCTTCATGGAAGAGATCTTCTTGATTTGAATGTATTTCTTAACAAGTATCTCAATGGTGTTATTCGTAGGGTTGAGATCCTTAAGGAGAACGGTGAGTCTTCTTCATCTGTACCTCCTCCTATTGGTGTAGCTCCTACTGTTGTGGATGCATCTGTCCCAATCGGTTTTGATGGTCGTATGATTCAAGATCAAAACCAAAATCAGCAAGAGCCGGTTCAATTCCAATACCAGGCTCTTTATGATTTTTATGATCAGATTCCAAAGAAACTTCATGATTTTAACATGAAAATGAATATAGATCCAAATCAGAGTATGAATTTGGATCTTAATGATGGAGAGGACGAGGGCATTCCTTGCATGGACAACAACAACTACCACCCCGAAATCGATTGTCTCGCTACCGTCACCACTGCCCCCACTGATGTTTGTGCTCCTAACATCATCAATGATCTCTAG

>AT1G65360.1 | Symbols: AGL23 | AGAMOUS-like 23 | chr1:24281337-24282151 FORWARD LENGTH=681

ATGGTGAAAAAAACTCTTGGTCGTAGAAAGGTAGAGATAGTGAAAATGACTAAGGAATCAAACCTTCAAGTCACATTTTCCAAGAGAAAAGCTGGTCTTTTTAAGAAGGCTAGTGAATTTTGCACATTATGTGATGCAAAAATTGCGATGATCGTGTTTTCACCAGCTGGAAAAGTATTTTCTTTTGGTCATCCAAATGTTGATGTTCTGCTTGACCACTTTCGAGGGTGTGTTGTAGGACACAACAACACAAACCTTGATGAAAGCTACACAAAGCTTCATGTTCAAATGCTCAACAAATCCTACACTGAGGTGAAGGCGGAAGTAGAAAAAGAACAAAAGAATAAGCAGTCGCGGGCTCAAAATGAAAGAGAAAACGAAAACGCTGAGGAGTGGTGGAGTAAGTCTCCATTAGAACTCAACTTAAGTCAATCAACCTGTATGATACGTGTTCTTAAAGATTTGAAGAAGATAGTTGATGAAAAAGCAATTCAATTAATCCATCAAACAAACCCAAACTTCTATGTTGGAAGTTCTAGCAATGCTGCTGCTCCAGCAACTGTTAGTGGTGGTAATATCTCCACAAACCAGGGGTTCTTTGATCAAAACGGAATGACGACTAATCCTACTCAAACACTTCTGTTTGGATTTGATATTATGAATCGCACACCAGGAGTTTAA

>AT1G69120.1 | Symbols: AP1, AtAP1, AGL7 | AGAMOUS-like 7, APETALA1 | chr1:25982576-25986102 REVERSE LENGTH=771

ATGGGAAGGGGTAGGGTTCAATTGAAGAGGATAGAGAACAAGATCAATAGACAAGTGACATTCTCGAAAAGAAGAGCTGGTCTTTTGAAGAAAGCTCATGAGATCTCTGTTCTCTGTGATGCTGAAGTTGCTCTTGTTGTCTTCTCCCATAAGGGAAAACTCTTCGAATACTCCACTGATTCTTGTATGGAGAAGATACTTGAACGCTATGAGAGGTACTCTTACGCCGAAAGACAGCTTATTGCACCTGAGTCCGACGTCAATACAAACTGGTCGATGGAGTATAACAGGCTTAAGGCTAAGATTGAGCTTTTGGAGAGAAACCAGAGGCATTATCTTGGGGAAGACTTGCAAGCAATGAGCCCTAAAGAGCTTCAGAATCTGGAGCAGCAGCTTGACACTGCTCTTAAGCACATCCGCACTAGAAAAAACCAACTTATGTACGAGTCCATCAATGAGCTCCAAAAAAAGGAGAAGGCCATACAGGAGCAAAACAGCATGCTTTCTAAACAGATCAAGGAGAGGGAAAAAATTCTTAGGGCTCAACAGGAGCAGTGGGATCAGCAGAACCAAGGCCACAATATGCCTCCCCCTCTGCCACCGCAGCAGCACCAAATCCAGCATCCTTACATGCTCTCTCATCAGCCATCTCCTTTTCTCAACATGGGTGGTCTGTATCAAGAAGATGATCCTATGGCAATGAGGAGGAATGATCTCGAACTGACTCTTGAACCCGTTTACAACTGCAACCTTGGCTGCTTCGCCGCATGA

>AT1G69540.1 | Symbols: AGL94 | AGAMOUS-like 94 | chr1:26145306-26147159 REVERSE LENGTH=1035

ATGGGTAGAGTGAAGCTAAAGATAAAGAAACTACAGAACATGAATGGACGTCAATGTACGTATACGAAAAGGAGACATGGGATTATGAAAAAGGCTAAGGAGTTATCGATCTTATGCGACATAGATGTTGTGCTTCTCATGTTTTCTCCCATGGGAAAGGCTTCAATTTGCATAGGCAAACACAGCATTGGAGAAGTCATTGCTAAGTTTGCTCAACTCTCTCCTCAAGAAAGAGCAAAGAGGAAGTTGGAAAACCTTGAAGCCTTGAGGAAAACTTTCATGAAAGCTAACCACGATATAGATATATCAAAGTTTCTAGACAGAATTAGTACACCAACAGTTGAGGTGCTTAGCGAAAAAATCAGGTTTCTGCAAACACAATTATCAGATATACACACACGACTAAGCTACTGGACCGATGTAGATAATATCGACAGCGTAGATGTTTTGCAGCAACTAGAACATTCACTAAGACAATCTCTGGCTCAAATCTATGGTCGTAAGGCGAGCATGCCGCAACGTCAGCAGCAACAACTTATGTCCTCACAATGCAAAAACCAGTTGCAGACTGAAATAGATATAGATTTCGGAATGGAGATGGAGCAACAACTTGAGAATTTCTCATGGGTTCGTACCGATGAAAACATGAATGTTCCTATAGAAGAAGAAGACCCTAATCTGCAGCTTCATCACATGTACAAGGACATAACGTGTTCTGCAAGTTCAGCTCTTGGAAATTACTCAGGACTCTTTAGTAAAAGCTCAGATATCTTACAAAAACTAGAAACCGGTAGCATTCCCGGGACATCGGCTGATCCGAACCAACAATTCAGCAATCTCAGTTTCTTAAATGATCAAAAGCTTAAGCAACTAGCTGAGTGGAATCTATTAGGCAGTCCTGCGGATTACTACGTTAGCCAGATCTTGGAAGCTTCTTATAAGCCTCAGATTGGAGGAAAAAACAACGGCGCTTCTTCTGAAACATTACCTTATGTTGCGGTCTTCGATGATCCTCTATATTTTTGGCCAAACTGA

>AT1G71692.1 | Symbols: XAL1, AGL12 | XAANTAL1, AGAMOUS-like 12 | chr1:26952903-26954939 REVERSE LENGTH=636

ATGGCTCGTGGAAAGATTCAGCTTAAGAGGATTGAGAACCCGGTTCACAGACAAGTGACTTTTTGCAAGAGGAGAACTGGTCTTCTCAAGAAGGCTAAGGAGCTCTCTGTGCTCTGTGATGCCGAGATCGGTGTTGTGATCTTCTCTCCTCAGGGCAAGCTCTTTGAGCTCGCTACTAAAGGAACAATGGAGGGAATGATTGATAAGTACATGAAGTGTACTGGTGGTGGTCGTGGTTCTTCTTCTGCTACTTTTACTGCTCAAGAACAACTTCAACCACCAAATCTTGATCCGAAAGATGAGATCAACGTGCTTAAGCAAGAGATTGAGATGCTTCAGAAAGGGATAAGCTATATGTTTGGAGGAGGAGATGGGGCTATGAATCTTGAAGAACTTCTTTTGCTTGAGAAGCATCTTGAGTATTGGATTTCTCAGATTCGCTCTGCTAAGATGGATGTTATGCTTCAAGAAATTCAGTCATTGAGGAACAAGGAAGGAGTCCTCAAAAACACCAACAAGTATCTCCTCGAAAAGATAGAGGAAAACAACAATAGCATATTAGATGCTAACTTCGCAGTCATGGAGACAAACTATTCCTATCCGCTAACAATGCCAAGTGAAATATTTCAGTTCTAG

>AT1G72350.1 | Symbols: AGL60 | agamous-like 60 | chr1:27239273-27239947 REVERSE LENGTH=675

ATGGAGGATGGGGAAGCTTCAACAATCACTTTCTTACCAACCACGGAACCAAAACCCCTACAAAACCCTAACTTGCTGGCCAAACCAAAAAAAGAGACTAAACAAAAAAAACCTAAAACCACCAAAGGTCGACAGAAGATAGAGATCAAGGAGATCATGCTGGAGACCCGAAGGCAAGTGACGTTTTCCAAACGACGATCCGGGCTTTTCAAAAAAGCGGCAGAATTAAGCGTTCTCTGCGGCGCACAGATTGGTATCATAACGTTTTCACGTTGCGATAGGATCTACTCGTTTGGTAACGTGAACTCACTCATCGATAAATACTTGCGTAAGGCTCCGGTGATGCTGAGGTCACATCCCGGTGGTAACGTGGCAAACGGAGAGGAAGATAACGACGGTTTGATGTGGTGGGAGAGAGCGGTGGAGAGTGTGCCGGAGGAGCATATGGAAGAGTACAAGAATGCCTTGAGTGTGTTAAGGGAGAATTTGTTGACGAGGATCTACCAGATGAGTGGTGATCGGACGGTTGAGAATCTTCCGGCATTTCCAAATGAGATGGCTATGGCTGACTGGAAATTAACGAATGAAAATCTGATGGCTAGGAACGATCGAGGTTATGGAGGTAACAATGGTGATTTGGAGTTTGCGTTTATGCCTCAAAACGGTAGACAGTGA

>AT1G77080.2 | Symbols: FLM, AGL27, MAF1 | FLOWERING LOCUS M, AGAMOUS-like 27, MADS AFFECTING FLOWERING 1 | chr1:28955679-28959845 FORWARD LENGTH=579

ATGGGAAGAAGAAAAATCGAGATCAAGCGAATCGAGAACAAAAGCAGTCGACAAGTCACTTTCTCCAAACGACGCAATGGTCTCATCGACAAAGCTCGACAACTTTCGATTCTCTGTGAATCCTCCGTCGCTGTTGTCGTCGTATCTGCCTCCGGAAAACTCTATGACTCTTCCTCCGGTGACGAGATAGAAGCGCTGTTCAAGCCGGAGAAACCTCAATGTTTTGAACTCGATCTTGAAGAAAAAATTCAGAATTATCTTCCACACAAGGAGTTACTAGAAACAGTCCAAAGCAAGCTTGAAGAACCAAATGTCGATAATGTAAGTGTAGATTCTCTAATTTCTCTGGAGGAACAACTTGAGACTGCTCTGTCCGTAAGTAGAGCTAGGAAGGCAGAACTGATGATGGAGTATATCGAGTCCCTTAAAGAAAAGGAGAAATTGCTGAGAGAAGAGAACCAGGTTCTGGCTAGCCAGATGGGAAAGAATACGTTGCTGGCAACAGATGATGAGAGAGGAATGTTTCCGGGAAGTAGCTCCGGCAACAAAATACCGGAGACTCTCCCGCTGCTCAATTAG

>AT1G77950.1 | Symbols: AGL67 | AGAMOUS-like 67 | chr1:29307029-29309667 FORWARD LENGTH=759

ATGGGTCGGGTTAAATTGGAGTTAAAGCGTATAGAGAAGAGCACGAACCGACAAATTACGTTCTCAAAACGTAAAAAAGGTTTAATAAAAAAGGCTTATGAATTGTCAACACTTTGTGACATCGATCTTGCCCTCCTCATGTTCTCTCCCTCTGATCGACTATGTCTCTTTTCCGGTCAAACAAGGATCGAGGACGTTTTGGCGAGGTACATCAATCTTCCTGATCAAGAAAGAGAAAACGCCATAGTTTTCCCTGATCAGAGCAAGCGCCAAGGTATCCAAAACAAAGAGTATTTGTTAAGGACTTTGGAAAAACTCAAAATTGAGGATGACATGGCTCTCCAAATCAACGAACCTCGTCCAGAAGCCACCAACTCCAATGTCGAGGAACTTGAGCAAGAAGTTTGTAGATTACAACAACAGCTTCAGATATCAGAGGAAGAACTCAGGAAATTCGAACCAGATCCAATGAGGCTAACATCAATGGAGGAGATTGAAGCATGTGAAGCTAACCTCATCAATACGTTGACACGTGTCGTCCAGAGAAGGGAACATTTGTTGAGAAAGTCCTGCGAAGCACAAAGTAACCAACAAAGCATGGACGGGATCCTTCTAAATGACATCGTCGAGGATTGGGGACCTGAGCCCGAGCCCAAACAAGCCCATATGATAGCTAATTCGGCCCATCATTCAAATCAGCCAAGCTATGATTTACTCTTACGCAGGAGTAATTCGAGTTCGAACCAAAATCCAAAATGA

>AT1G77980.1 | Symbols: AGL66 | AGAMOUS-like 66 | chr1:29315212-29317067 REVERSE LENGTH=999

ATGGGTCGAGTGAAATTGGAGATAAAACGAATAGAAAACACAACGAATCGACAAGTTACATTCTCCAAAAGACGAAATGGTCTTATAAAGAAAGCTTATGAATTGTCCATTCTTTGTGACATCGACATTGCTCTCCTCATGTTCTCTCCCTCTGATCGCCTTAGCCTCTTTTCGGGCAAAACAAGGATTGAAGACGTTTTCTCTAGATACATCAATCTTTCTGATCAAGAACGAGAGAATGCTCTAGTATTCCCCGATCAAAGTCGACGCCCAGATTTCCAGAGCAAAGAGTATCTACTCCGGACTTTGCAGCAACTCAAGGCTGAGAATGATATTGCTCTTCAACTTACCAACCCTACAGCTATCAACTCCGACGTCGAGGAACTTGAGCATGAAGTTTATAAGTTACAACAACAACTTCTCATGGCAGAGGAAGAACTAAGGAAATACGAACCAGATCCAATCAGGTTTACAACAATGGAAGAGTACGAAACTTGTGAGAAGCAGCTCATGGACACCTTAACACGTGTCAATCAACGACGGGAACATATATTGAGTCAAGACCAATTATCTTCATATGAAGCATCTGCTTTACAACAACAGCAAAGCATGGGTGGGCCTTTTGGAAACGACGTCGTTGGAGGATGGCTGACTGAAAATGGGCCTAACGAAGCCCATTTATTCGACGCATCGGCACATTCAGCAATGTATGAAACTTTATTGCAAGGAAGTAGCTCAAGCTCGAACCAAAACAACATTATGGGTGAATCCAATGTGTCAAATCATAACGGTGACATGTTTCAAGAATGGGCCCAAGCCTATAATTCTACTACGGCCCATAACCCTTCGACTCTATTTCCTCCTATGCAGCATCAGCATGGACTGGTGGTTGATCCCAATATAGAAGAAATTGAAATACCGGTTATGAAGAAGGATGCACAAGCAGACCACGAGGTCTCCGACTATGATATAAGAATGCCTCAGCTCAGTAGCCAATAA

>AT2G03060.1 | Symbols: AGL30 | AGAMOUS-like 30 | chr2:901614-903639 FORWARD LENGTH=1146

ATGGGAAGGGTAAAATTGAAGATAAAGAAGTTAGAGAACACAAATGGACGCCAATCTACATTTGCTAAAAGGAAAAATGGGATCTTGAAAAAGGCTAATGAGCTATCTATTCTTTGTGACATTGATATTGTTCTTCTTATGTTCTCTCCTACTGGCAAGGCTGCAATATGTTGCGGTACACGAAGTAGCATGGAAGAGGTGATTGCTAAGTTTTCTCAAGTAACACCGCAGGAAAGAACGAAAAGGAAGTTCGAGAGTCTTGAAAACTTGAAGAAAACTTTCCAAAAGTTGGATCACGATGTAAATATACGCGAATTTATAGCCTCAAGTAATTCAACAGTAGAGGACTTGAGTACTCAAGCAAGGATTCTGCAGGCTCGGATTTCTGAGATACATGGAAGATTAAGTTATTGGACGGAACCAGATAAGATTAACAATGTTGAACACTTGGGACAGCTCGAAATTTCGATTAGGCAATCCCTTGATCAATTGCGTGCACACAAGGAACATTTTGGGCAGCAGCAACAGGCAATGCAAATAGAAAACGCAAACTTTGTTAAGGATTGGTCAACATGCTCGATGCAAGATGGGATTCAGATTCCTTTAGAACAACAGCTTCAATCTATGTCATGGATTCTTAATAGCAACACCACCAACATTGTCACCGAGGAACACAATTCAATCCCGCAGAGGGAAGTCGAGTGCTCAGCGAGTTCTTCATTCGGGAGCTATCCAGGCTACTTTGGAACAGGGAAATCTCCTGAAATGACAATTCCGGGTCAAGAAACAAGCTTTCTTGATGAACTAAACACCGGACAGCTGAAACAGGACACAAGCTCGCAGCAGCAGTTCACTAATAATAATAATATCACAGCATACAATCCCAATCTTCACAATGATATGAATCATCACCAAACGTTGCCTCCTCCTCCTCTTCCTCTTACTCTTCCGCATGCTCAGGTGTATATTCCAATGAATCAGAGAGAGTATCATATGAATGGATTCTTTGAAGCACCACCACCTGATTCTTCTGCTTACAACGACAACACCAACCAAACCAGGTTTGGTTCTAGCAGCAGCTCCTTGCCTTGCTCAATCTCAATGTTCGACGAATACTTGTTTTCCCAGATGCAGCAGCCGAACTGA

>AT2G03710.1 | Symbols: SEP4, AGL3 | SEPALLATA 4, AGAMOUS-like 3 | chr2:1129622-1131628 FORWARD LENGTH=777

ATGGGAAGAGGGAAAGTTGAGCTGAAGAGGATAGAGAACAAGATCAATAGACAAGTTACTTTTGCAAAGAGAAGAAATGGTTTGCTCAAGAAGGCTTATGAGCTTTCTGTCCTTTGTGATGCTGAGATTGCTCTTCTCATTTTCTCTAACCGTGGCAAGCTCTACGAATTCTGCAGCAGCCCTAGTGGTATGGCGAGGACGGTTGATAAGTATAGAAAACATAGTTATGCAACAATGGATCCAAATCAATCAGCTAAAGACTTGCAGGATAAGTATCAAGACTACTTGAAGCTTAAATCAAGAGTTGAGATCCTTCAACATTCACAAAGGCATTTGCTAGGTGAAGAGCTATCCGAGATGGATGTGAATGAGCTTGAGCATCTCGAACGCCAAGTAGATGCATCACTAAGACAAATAAGATCTACCAAGGCTCGGTCTATGCTTGATCAACTATCTGACCTCAAAACTAAGGAGGAAATGTTATTGGAAACCAATAGAGATCTTAGGAGAAAGTTGGAGGACAGTGATGCAGCACTTACTCAATCGTTTTGGGGAAGTTCTGCTGCAGAACAACAACAACAACATCAACAACAGCAACAAGGCATGAGCTCTTATCAATCAAACCCTCCAATTCAGGAAGCAGGTTTCTTCAAGCCTCTACAAGGCAATGTAGCATTGCAAATGAGCAGTCATTACAATCACAATCCTGCAAATGCAACCAACTCTGCAACAACATCACAGAATGTTAATGGATTCTTCCCTGGATGGATGGTCTGA

>AT2G14210.1 | Symbols: ANR1, AtANR1, AGL44 | ARABIDOPSIS NITRATE REGULATED 1, AGAMOUS-like 44 | chr2:6018841-6023585 FORWARD LENGTH=705

ATGGGGAGAGGGAAGATAGTTATACGAAGGATCGATAACTCTACAAGTAGACAAGTGACTTTCTCCAAGAGAAGGAGTGGTTTGCTTAAGAAAGCTAAAGAGTTATCGATCCTTTGTGATGCAGAAGTTGGTGTTATCATATTCTCTAGCACCGGAAAGCTCTACGACTACGCAAGCAATTCAAGTATGAAAACAATCATTGAGCGGTACAACAGAGTAAAAGAGGAGCAGCATCAACTTCTGAATCATGCCTCAGAGATAAAGTTTTGGCAAAGAGAGGTTGCAAGTTTGCAGCAGCAGCTCCAATATCTACAAGAATGCCACAGGAAACTAGTGGGAGAGGAACTTTCTGGAATGAATGCTAACGACCTACAAAACCTTGAAGACCAGCTAGTAACAAGTCTAAAAGGTGTTCGTCTCAAAAAGGATCAACTTATGACAAATGAAATCAGAGAACTTAATCGTAAGGGACAAATCATCCAAAAAGAGAATCACGAGCTACAAAATATTGTAGATATAATGCGTAAGGAAAATATTAAATTGCAAAAGAAGGTTCATGGAAGAACAAATGCGATTGAAGGCAATTCAAGTGTAGATCCAATAAGCAATGGAACCACAACATATGCACCACCGCAACTTCAACTCATACAACTACAACCAGCTCCTAGAGAAAAATCAATCAGACTAGGGCTACAACTTTCCTAG

>AT2G22540.1 | Symbols: AGL22, FAQ1, SVP | SHORT VEGETATIVE PHASE, Flowering Arabidopsis QTL1, AGAMOUS-like 22 | chr2:9580417-9583603 FORWARD LENGTH=723

ATGGCGAGAGAAAAGATTCAGATCAGGAAGATCGACAACGCAACGGCGAGACAAGTGACGTTTTCGAAACGAAGAAGAGGGCTTTTCAAGAAAGCTGAAGAACTCTCCGTTCTCTGCGACGCCGATGTCGCTCTCATCATCTTCTCTTCCACCGGAAAACTGTTCGAGTTCTGTAGCTCCAGCATGAAGGAAGTCCTAGAGAGGCATAACTTGCAGTCAAAGAACTTGGAGAAGCTTGATCAGCCATCTCTTGAGTTACAGCTGGTTGAGAACAGTGATCACGCCCGAATGAGTAAAGAAATTGCGGACAAGAGCCACCGACTAAGGCAAATGAGAGGAGAGGAACTTCAAGGACTTGACATTGAAGAGCTTCAGCAGCTAGAGAAGGCCCTTGAAACTGGTTTGACGCGTGTGATTGAAACAAAGAGTGACAAGATTATGAGTGAGATCAGCGAACTTCAGAAAAAGGGAATGCAATTGATGGATGAGAACAAGCGGTTGAGGCAGCAAGGAACGCAACTAACGGAAGAGAACGAGCGACTTGGCATGCAAATATGTAACAATGTGCATGCACACGGTGGTGCTGAATCGGAGAACGCTGCTGTGTACGAGGAAGGACAGTCGTCGGAGTCTATTACTAACGCCGGAAACTCTACCGGAGCGCCTGTTGACTCCGAGAGCTCCGACACTTCCCTTAGGCTCGGCTTACCGTATGGTGGTTAG

>AT2G22630.1 | Symbols: AGL17 | AGAMOUS-like 17 | chr2:9618372-9621641 FORWARD LENGTH=684

ATGGGGAGAGGGAAGATTGTGATCCAGAAGATCGATGATTCCACGAGTAGACAAGTCACTTTCTCCAAAAGAAGAAAGGGTCTCATCAAGAAAGCTAAAGAACTTGCTATTCTCTGCGACGCCGAGGTCTGTCTCATCATTTTCTCCAACACTGACAAGCTCTATGACTTTGCCAGCTCCAGTGTGAAATCTACTATTGAACGATTCAATACGGCTAAGATGGAGGAGCAAGAACTAATGAACCCTGCATCAGAAGTTAAGTTTTGGCAGAGAGAGGCTGAAACTCTAAGGCAAGAATTGCACTCATTGCAAGAAAATTATCGGCAACTAACGGGAGTGGAATTAAATGGTTTGAGCGTTAAGGAGTTACAAAACATAGAGAGTCAACTTGAAATGAGTTTACGTGGAATTCGTATGAAAAGGGAACAAATTTTGACCAATGAAATTAAAGAGCTAACCAGAAAGAGGAATCTTGTTCATCATGAAAACCTCGAATTGTCGAGAAAAGTACAAAGGATTCATCAAGAAAATGTCGAACTATACAAGAAGGCTTATGGAACGTCGAACACAAATGGATTGGGACATCATGAGCTAGTAGATGCAGTTTATGAATCCCATGCACAGGTTAGGCTGCAGCTAAGCCAGCCTGAGCAGTCCCATTATAAGACATCTTCAAACAGCTAA

>AT2G24840.1 | Symbols: DIA, AGL61 | DIANA, AGAMOUS-like 61 | chr2:10581082-10581876 FORWARD LENGTH=795

ATGTATGTTACAAAATATAAAAATAAATTACCAACTTTGCAAGTTGATCTCATAATAATGCCACACCAGACTCAAGCATGTATATATAAACAAACACTTTCTCTCCACCAAATATTCCAACAAAGAAAAGCAACTAAAACCCTCCATACAAAAAAAACTATGATGTCGAAGAAGAAAGAAAGCATCGGACGACAAAAAATTCCAATGGTAAAAATAAAGAAAGAGAGCCACCGGCAAGTCACATTCTCCAAACGCAGAGCCGGTCTCTTCAAGAAAGCTAGTGAGCTTTGCACTTTGTGTGGTGCAGAGATTGGGATCATCGTGTTTTCTCCCGCGAAAAAGCCTTTCTCATTCGGACATCCAAGTGTTGAATCTGTATTGGATCGCTACGTGTCTCGAAACAATATGTCCTTAGCTCAGTCGCAGCAACTGCAAGGAAGCCCTGCAGCGAGCTGCGAACTGAATATGCAGTTAACGCATATTTTGAGCGAGGTAGAAGAAGAGAAGAAGAAGGGTCAAGCGATGGAAGAGATGAGAAAAGAGAGTGTGAGGCGGTCGATGATTAATTGGTGGGAAAAGCCAGTAGAGGAGATGAATATGGTTCAGTTACAGGAAATGAAGTATGCATTGGAGGAGTTGAGGAAGACGGTTGTGACAAACATGGCGTCGTTTAATGAGGCCAAAGACGATGTGTTTGGTTTCTTGGACAACAAAGTGACAGTCCCTCCTTACGTGAACATGCCTTCTGGTCCCTCAAATATTTATAATTTTGCCAATGGAAATGGTTGTTTCTGA

>AT2G26320.1 | Symbols: AGL33 | AGAMOUS-like 33 | chr2:11205389-11206287 REVERSE LENGTH=330

ATGAAGAGAACAATCAAAAATAAAAATAAACAAATAGTTAAAGAAAATATGGGGAGGAAAAAACTAAAATTGAAGAGAATAGAGTCCCTAAAAGAGAGAAGTAGCAAATTTTCAAAACGTAAAAAAGGTCTTTTTAAAAAGGCAGAAGAAGTAGCATTGTTATGCGACTCGGATATAATGTTAATTGTTGTTTCTCCCACCGAGAAGCCTACAGTTTTTAACACTCGTTCTAGATCATTCCATACAATCCTTGAGAGGTTTTGCATGCTTTCATTACAAGAACGGGAAGAGAGGTGTGATCTTTCATATTTTTATATAATTATTACATAA

>AT2G28700.1 | Symbols: AGL46 | AGAMOUS-like 46 | chr2:12317384-12318724 REVERSE LENGTH=990

ATGGCAAGAAAAAAGTTAAATCTAACTTACATTTTCAATGATAGAATGAGGAAAAGATCGTTCAAGCAGAGGAGAGAAGGATTTCTGAAGAAACTCAATGATCTCAAGGTTTTATGCGATGTTAATGCATGTGCGGTCGTCTACAATCCATTCAACTCAAATCCAGATGTGTGGCCATCAAAATCGGAGGTGAATAATATCATCAAGAAATTTGAGATGTTACCGGAGACCCAAAAGAAAGTTAAATCGGTGAACCACGAAGAATTTCTCAATCTATACATCTCAAAAGTCGAAAAGCAAAGTAAGAAATTAATTGTAGAAAATAAGGAGACTTGCTTAAAGGAGGTCATGTTCAAGTGTCTTGGTGGGAACATGGGAGATTTTGTTATGAATGATAATGATCGTCTTGACTTATGTAAGTTTATTGATCACTATCTTAGAAATCTTTATCATCATAAAAATGTAACCCTCAACAATCCAAATTTTGAAATCGGAGAATCTTCTTCATTAATGGATATGGCACCAACCGCTACAACTGGAAACATGGCAACAACTGTAGTAGATGAAGGCATGACACCACTTCTTATTGCTGAAGGGAGTTCCTCCTCATTTTTGAATTCTCCACTCTTCAACTCTCCTCAGCTAACCAACGAGTTGCAGCTTATAGTCTCTCAAAACCATAGATTAGAGAATTCTCTGGCAAGTAATCTCTTTTTCAGTGAAGGGCAAGACATTTGCATTCCAGATATGAATCAGTCCATAATCCCATCAAACCAAGGAGCAGAACATGTGGATTTTCTTGAAAGTAATTTTCTACCTAATAACAATCAAGAAGTTTATATCCCAGTTATGGATCAGGATGAAGTTTACAATCCAAATCAAAATCATTATGAGAACCAACAAGGATTCATTGACGAGATGATGAAATATGCTGAGAAAACAAGTTTTCCTTGGATGGTAGAGAACCACTGTTATAATCACAACCAGTAA

>AT2G34440.1 | Symbols: AGL29 | AGAMOUS-like 29 | chr2:14526950-14527468 FORWARD LENGTH=519

ATGGGTCGGAGAAAGATCAAGATGGAGATGGTTCAGGACATGAACACACGACAGGTTACCTTTTCAAAACGGAGGACTGGTTTGTTCAAGAAGGCGAGCGAGTTAGCCACGCTCTGCAACGCTGAGTTGGGCATCGTTGTCTTTTCACCAGGAGGCAAGCCTTTCTCCTACGGGAAACCGAATCTTGATTCTGTTGCAGAGCGATTCATGAGAGAATATGATGATTCAGACAGTGGCGATGAAGAAAAAAGTGGTAATTACAGGCCTAAACTGAAGAGGCTGAGTGAACGTCTCGATTTGCTCAACCAAGAGGTTGAAGCTGAGAAGGAACGAGGCGAGAAGAGTCAGGAGAAGCTTGAATCTGCTGGGGATGAGAGATTCAAGGAGTCCATTGAGACGCTTACCCTCGATGAACTCAATGAATACAAAGATAGGCTTCAGACAGTCCATGGTAGGATTGAAGGTCAAGTCAATCACTTGCAGGCTTCGTCTTGCCTCATGCTTCTCTCCAGAAAATAG

>AT2G40210.1 | Symbols: AGL48 | AGAMOUS-like 48 | chr2:16793213-16794328 REVERSE LENGTH=1116

ATGACAAGAAAGAAAGTAAAACTTGTATGGATCGAGAATGACAAGTCAAGAGCAACAAGTTTGCAAAAAATGAGGGTAGGATTACTTAAAAAGGTGAAAGAGCTAACCATATTGTGTGCTGTAAGGGCTATTGTAATCATTTTCAGCCCCGATAAGGTTGGACCATTGGTGTGGCCATCTCCTCAGGCGACTCATGGTCTCCTAGACGAATTTTTCGCTTTACCGAAGTCCGTGCAGAAGAAGAAAGAGTCAAATGTCGAATCCTACCTAAAAGAGAAGACACATAAGTTTCAAGAACAATTGAAGAAGAGTAAAAAGAAGAACAAGGAGCATGTCATTGATGAGCTGATGATGCAACTCCAAAGCGGTCGTGAAATTGCTGATCTTAACCAAAGTGAGATGTATGCCTTGTTATCTTTCTCAAGAGATACTATCCTACTTTGTAGGAAGAAACTAGCTTTCATGCAATTTCCTCCTCTTCGCGATCCACCAGTGTTTCCATTTGAAATACAAGTCGAAGAGTTCAAAACCACCACAAATGATGGTTTTGTAGGAGGCGGTCAAGATAATAAAAGAGCTGGAAGGACAGATGAAGCAACAAGATTCATCAACACCGATATATTTAAGCAGAGTAAAAGTTATTACTTTTTTGATGAATGGGTTTTTCCACCTAGTCCACCTAAATATGAAATACCGCAACAAATGGAGAACGGAAATCCAAACCCTAAAAGCTATCGTCTTTATCAAGGAAGCAGTAGCAACGGAAACCCTCATTTGGAAATGGATCCATTTCGTCTCCAAATGATGACTTCCCAAGGTTTGGCTGGGTCAGTCTCCCAACCTTTACAACATCACAGCATGATCAATAATCCAACAATGGCTATGAATCAACCAAGCCAAGATCCTTTTGATTACATGAGAAGTGAGCTGGGAATAAACGAAGGAATCAACATTAACAACTCACAATTTTACATGAGCAACAACACTATAACGGCTAATGATGGTGTTCGCCAAGAACCATATCCTAATGTAACAACCGCTGGAGAAAACAATGGTGATGCGACGACGTCTAATACCAACATGGTGTGGCCGGGTTTTAACAATCATCATTTCTAA

>AT2G42830.1 | Symbols: AGL5, SHP2 | AGAMOUS-like 5, SHATTERPROOF 2 | chr2:17820602-17823806 FORWARD LENGTH=741

ATGGAGGGTGGTGCGAGTAATGAAGTAGCAGAGAGCAGCAAGAAGATAGGGAGAGGGAAGATAGAGATAAAGAGGATAGAGAACACTACGAATCGTCAAGTCACTTTCTGCAAACGACGCAATGGTTTACTCAAGAAAGCTTATGAGCTCTCTGTCTTGTGTGACGCTGAGGTTGCTCTTGTCATCTTCTCCACTCGAGGCCGTCTCTACGAGTACGCCAACAACAGTGTGAGAGGAACAATAGAAAGGTACAAGAAAGCTTGCTCCGACGCCGTTAACCCTCCGACCATCACCGAAGCTAATACTCAGTACTATCAGCAAGAGGCGTCTAAACTCCGGAGACAGATTCGGGACATTCAGAATTTGAACAGACACATTCTTGGTGAATCTCTTGGTTCCTTGAACTTTAAGGAACTCAAGAACCTTGAAAGTAGGCTTGAGAAAGGAATCAGTCGTGTCCGATCCAAGAAGCACGAGATGTTAGTTGCAGAGATTGAATACATGCAAAAAAGGGAAATCGAGCTGCAAAACGATAACATGTATCTCCGCTCCAAGATTACTGAAAGAACAGGTCTACAGCAACAAGAATCGAGTGTGATACATCAAGGGACAGTTTACGAGTCGGGTGTTACTTCTTCTCACCAGTCGGGGCAGTATAACCGGAATTATATTGCGGTTAACCTTCTTGAACCGAATCAGAATTCCTCCAACCAAGACCAACCACCTCTGCAACTTGTTTGA

>AT2G45650.1 | Symbols: AGL6, RSB1 | AGAMOUS-like 6, REDUCED SHOOT BRANCHING 1 | chr2:18804453-18806291 FORWARD LENGTH=759

ATGGGAAGAGGGAGAGTGGAGATGAAGAGGATAGAGAACAAGATTAATAGACAAGTGACCTTCTCAAAAAGAAGAAACGGTTTGCTGAAGAAAGCTTATGAGCTTTCTGTTCTTTGCGATGCCGAAGTTGCTCTCATCATCTTCTCAAGCCGTGGCAAGCTCTACGAGTTTGGTAGTGTTGGAATTGAAAGCACAATCGAACGGTATAATCGTTGTTACAACTGCTCTCTAAGCAATAATAAGCCTGAAGAGACTACACAGAGTTGGTGTCAGGAGGTGACAAAGCTTAAATCCAAATACGAATCTCTTGTTCGTACTAACAGGAATTTGCTTGGAGAAGATCTTGGAGAAATGGGTGTGAAGGAACTGCAAGCGCTCGAGAGGCAGCTCGAAGCCGCTCTTACCGCGACTCGACAGCGCAAGACACAAGTTATGATGGAAGAAATGGAAGACCTTAGGAAAAAGGAGAGGCAACTAGGAGACATAAACAAACAACTCAAGATTAAGTTTGAAACGGAAGGCCATGCTTTCAAAACCTTTCAAGACTTATGGGCAAACTCGGCGGCATCGGTGGCCGGGGATCCAAACAATTCTGAATTTCCGGTAGAGCCTTCTCATCCTAATGTATTGGATTGCAACACCGAACCCTTTTTACAAATAGGGTTTCAACAACATTACTACGTGCAAGGTGAAGGGTCTTCGGTATCAAAGAGTAACGTGGCAGGTGAGACTAATTTCGTCCAAGGTTGGGTTCTTTGA

>AT2G45660.1 | Symbols: AGL20, SOC1, ATSOC1 | SUPPRESSOR OF OVEREXPRESSION OF CO 1, AGAMOUS-like 20 | chr2:18807799-18810193 REVERSE LENGTH=645

ATGGTGAGGGGCAAAACTCAGATGAAGAGAATAGAGAATGCAACAAGCAGACAAGTGACTTTCTCCAAAAGAAGGAATGGTTTGTTGAAGAAAGCCTTTGAGCTCTCAGTGCTTTGTGATGCTGAAGTTTCTCTTATCATCTTCTCTCCTAAAGGCAAACTTTATGAATTCGCCAGCTCCAATATGCAAGATACCATAGATCGTTATCTGAGGCATACTAAGGATCGAGTCAGCACCAAACCGGTTTCTGAAGAAAATATGCAGCATTTGAAATATGAAGCAGCAAACATGATGAAGAAAATTGAACAACTCGAAGCTTCTAAACGTAAACTCTTGGGAGAAGGCATAGGAACATGCTCAATCGAGGAGCTGCAACAGATTGAGCAACAGCTTGAGAAAAGTGTCAAATGTATTCGAGCAAGAAAGACTCAAGTGTTTAAGGAACAAATTGAGCAGCTCAAGCAAAAGGAGAAAGCTCTAGCTGCAGAAAACGAGAAGCTCTCTGAAAAGTGGGGATCTCATGAAAGCGAAGTTTGGTCAAATAAGAATCAAGAAAGTACTGGAAGAGGTGATGAAGAGAGTAGCCCAAGTTCTGAAGTAGAGACGCAATTGTTCATTGGGTTACCTTGTTCTTCAAGAAAGTGA

>AT3G02310.1 | Symbols: AGL4, SEP2 | AGAMOUS-like 4, SEPALLATA 2 | chr3:464554-466687 REVERSE LENGTH=753

ATGGGAAGAGGAAGAGTAGAGCTCAAGAGGATAGAGAACAAAATCAACAGACAAGTGACGTTTGCTAAACGTAGAAATGGTTTGCTGAAAAAAGCTTATGAGCTTTCTGTTCTCTGCGATGCTGAAGTCTCTCTCATCGTCTTCTCCAACCGTGGCAAGCTCTACGAGTTCTGCAGCACCTCCAACATGCTCAAGACACTGGAAAGGTATCAGAAGTGTAGCTATGGCTCCATTGAAGTCAACAACAAACCTGCTAAAGAGCTTGAGAACAGCTACAGAGAGTACTTGAAGCTGAAAGGTAGATATGAAAATCTGCAACGTCAGCAGAGAAATCTTCTTGGAGAGGATCTTGGACCTCTGAATTCAAAGGAGCTAGAGCAGCTTGAGCGTCAACTAGACGGCTCTCTGAAGCAAGTTCGCTGCATCAAGACACAGTATATGCTTGACCAGCTCTCTGATCTTCAAGGTAAGGAGCATATCTTGCTTGATGCCAACAGAGCTTTGTCAATGAAGCTGGAAGATATGATCGGCGTGAGACATCACCATATAGGAGGAGGATGGGAAGGTGGTGATCAACAGAATATTGCCTATGGACATCCTCAGGCTCATTCTCAGGGACTATACCAATCTCTTGAATGTGATCCCACTTTGCAAATTGGATATAGCCATCCAGTGTGCTCAGAGCAAATGGCTGTGACGGTGCAAGGTCAGTCCCAACAAGGAAACGGCTACATCCCTGGCTGGATGCTGTGA

>AT3G04100.1 | Symbols: AGL57 | AGAMOUS-like 57 | chr3:1075299-1075922 FORWARD LENGTH=624

ATGTCATCTACGAAGCAAGCAAAGGGAAGAAAAACAAAGGGGAAGCAAAAGATCGAGATGAAGAAGGTGGAGAACTATGGAGATAGGATGATTACGTTCTCAAAACGTAAAACCGGAATTTTTAAGAAAATGAACGAGCTCGTAGCAATGTGTGACGTTGAAGTGGCTTTCTTGATTTTCTCTCAACCCAAGAAGCCCTATACATTCGCACATCCGTCTATGAAGAAAGTGGCTGACCGGTTAAAGAACCCTTCGAGACAAGAACCATTAGAGAGAGACGATACCAGACCCCTCGTCGAAGCTTATAAGAAACGAAGGCTCCACGACCTCGTAAAAAAAATGGAGGCGCTCGAAGAGGAGCTTGCGATGGATCTAGAGAAGTTGAAACTGTTGAAGGAATCGAGAAATGAAAAGAAGTTAGATAAAATGTGGTGGAACTTTCCTTCGGAAGGTTTGAGCGCGAAGGAGCTGCAGCAAAGGTACCAAGCGATGCTCGAGTTACGTGATAACTTATGCGACAATATGGCTCACTTACGATTGGGAAAAGACTGTGGTGGTTCATCTTCTGTTCGTGTGGGACGTCGAGTTTCTGGTGGTGTTCGTCTGTTCGATCGTGAAGCATGA

>AT3G05860.1 | Symbols: AGL45 | agamous-like 45 | chr3:1751406-1752355 REVERSE LENGTH=783

ATGACGAGGAAGAAGCTAAACCTATCTTACATCACCAATGAGTCAATGAGAAAAGCAACATTCAATAAGAGAAAGAAAGGGCTTGTGAAAAAAATCCACGAGCTTTCCGTTCTCTGCGGAATCGAAGCATGTGCAGTGATTTATAGTCCGTTCAACTCAAACCCTGAAGTCTGGCCATCGAATTCAGAAGTTAAAAATGTAATGGAAAATTTTGAGATGTTGACAAAGTTGGAGCAAGAGAAGAAAATGGTGAGCCACGAAGGTTTTATCAGACAAAACATCTCAAAAACTATGGAGAGTAACAACAAAAAGATGATCGATAATGCGGAGAGGACGATGAAAGAAGCCATGTTCCAACTTCTTAGTGGAAAAGGGGAGAAGCTTAATTTGACTGATAGAAACCGTGAAGATTTGTGTAAGTACATTGATCAATATCTTAAAGAACTTTATCACCACAAAAACAAAACCATAAATCAATCACATATTGAACCTGGAGAATCTTCGGGAGCTACAAATGCCATGACACCAACATCTGTTGTTGAACCTATTATCTCTTCAATCCAGCGACCAAATCAGAATCCAAATTTTAATCATCTTAGTCATAATCAATATCAATATCAACAACAGTTTGGATATCCGATTTTGGTTCAAGATGGAATTTACAATCCGAGTCAAATTCAGAATCAACATGAGGAATGGCTGGATGATCATATGATGAATCATTCTAAGGAAATAAGTCATCCTTTGATGGATGATAACAACTTTTACTACCAACAACCCTAG

>AT3G18650.1 | Symbols: AGL103 | AGAMOUS-like 103 | chr3:6417344-6418504 REVERSE LENGTH=1161

ATGGCTTCTTCTTCGTCCTCTTCTCTTTCGTTTTCAACATCGAAGAAGAACAAAACCTTTTTCAAGAAACCTAATTCTGCTTTTTCTTCTTCGAGGGCCACAAGTTTGATCAAAAGACAACAGACTGTCTTTAAGAAAGCTAAAGAGCTTTCAATTCTCTGTGACATCGATGTCTGCGTCATCTGTTACGGATCAAACGGAGAGCTTAAGACATGGCCTGAAGAGAGAGAGAAGGTCAAAGCCATAGCTCGAAGGTACGGTGAATTAAGCGAGACGAAGCGTCGCAAAGGAAGCGTTGATCTTCACGAGTTCCTCGAGAAGATGAACAAGGATGATCCCGAGAAGGAGGAGAAGAAGAAGATTAAAGTGAGACGTGTACCCAAAGTCAAGTATCCAGTTTGGGACCCTAGGTTTGACAATTACTCTGTGGAGCAACTCATGGGACTGGTTCAGTCCTTGGAACGAAACCTAACCAGGATCCAACATAGGACTTGCGCTGTTGTTGAAGCTCAGGGGCAGAGGAGGGTACAGTACACGAACATGGCTAATCAAGAACTGATGATGGCTAATACGATGAATCAACTTCAACAACACTCAAACCAGGTTTCCATGTATCTGTGGAACCATGGAAACGGTGCTTTCTCACAAATCCCAGTCTCAGCATTGGCTTCAAACCAAACGCAATCTCTAGCTCCGATTCCGCCTGAACTGATGATTTACCCGAATTCGGATGCGGGAAATTACTCGGGGTCTCTCGGGGTACAAGGAACTGGGATCAATGGGCTTCAGAACATGAACATGTTAACCTATAACAACATCAACAGCGTTAATGATTTCTCAAAACAGTTTGACCAGAATTCCAGAGCGGAAAGTTACTCTAGCTTACTTGGGGTACACGAAGACGGAAACAATGAGTTTGAGAACCCTAACATGTCCAGCCGCAACAACTTCAATGTTCAAGACTGTGCAGGGTTACTTGGGATGCAAGGAGCTGGAACTAACGGGTTGCAGAGCATGAACATGCATGACTACAGTAACAACAACAGCATTAACTCTAATGGTCTTTCGCATCAGTATGTTCAATTTCCAACGTACAACAGCCAACATCAGGACCGGGTTTTCAATTTGGATCAAAACGGAAACAATACTAGGTCTCTCTAA

>AT3G30260.1 | Symbols: AGL79 | AGAMOUS-like 79 | chr3:11909119-11912880 FORWARD LENGTH=750

ATGGGAAGAGGAAGGGTTCAGCTACGGCGGATCGAGAATAAGATAAGGAGACAAGTGACATTTTCAAAGCGAAGGACCGGTTTGGTAAAGAAAGCTCAAGAGATCTCAGTGTTATGTGATGCTGAAGTTGCTTTGATTGTTTTCTCCCCCAAAGGCAAACTCTTTGAGTACTCTGCTGGTTCCAGCATGGAGAGAATTCTTGATCGATATGAGAGGTCTGCCTACGCCGGTCAAGATATTCCTACACCAAATTTGGATTCACAGGGAGAGTGCTCAACAGAATGTTCAAAGCTCTTGAGGATGATTGATGTCCTGCAAAGAAGCCTGAGGCACTTAAGAGGAGAAGAGGTGGATGGTCTAAGTATCAGAGACCTTCAAGGTGTGGAGATGCAACTTGATACTGCCCTCAAGAAAACTCGCTCTAGAAAGAACCAGCTCATGGTAGAGTCCATAGCACAGCTTCAGAAAAAGGAGAAGGAACTAAAAGAACTGAAGAAACAGCTAACAAAGAAGGCTGGTGAAAGGGAAGACTTTCAAACGCAAAACCTCAGCCATGACTTAGCCTCCTTGGCAACACCGCCATTTGAATCACCCCATGAGCTTCGCCGGACAATATCTCCTCCTCCTCCTCCTTTATCTTCGGGGGATACATCACAAAGGGATGGAGTTGGAGAAGTAGCTGCCGGAACCCTAATTCGAAGGACGAATGCAACGTTGCCTCATTGGATGCCCCAGCTCACCGGAGAATAG

>AT3G54340.1 | Symbols: AP3, ATAP3 | APETALA 3 | chr3:20119428-20121087 REVERSE LENGTH=699

ATGGCGAGAGGGAAGATCCAGATCAAGAGGATAGAGAACCAGACAAACAGACAAGTGACGTATTCAAAGAGAAGAAATGGTTTATTCAAGAAAGCACATGAGCTCACGGTTTTGTGTGATGCTAGGGTTTCGATTATCATGTTCTCTAGCTCCAACAAGCTTCATGAGTATATCAGCCCTAACACCACAACGAAGGAGATCGTAGATCTGTACCAAACTATTTCTGATGTCGATGTTTGGGCCACTCAATATGAGCGAATGCAAGAAACCAAGAGGAAACTGTTGGAGACAAATAGAAATCTCCGGACTCAGATCAAGCAGAGGCTAGGTGAGTGTTTGGACGAGCTTGACATTCAGGAGCTGCGTCGTCTTGAGGATGAAATGGAAAACACTTTCAAACTCGTTCGCGAGCGCAAGTTCAAATCTCTTGGGAATCAGATCGAGACCACCAAGAAAAAGAACAAAAGTCAACAAGACATACAAAAGAATCTCATACATGAGCTGGAACTAAGAGCTGAAGATCCTCACTATGGACTAGTAGACAATGGAGGAGATTACGACTCAGTTCTTGGATACCAAATCGAAGGGTCACGTGCTTACGCTCTTCGTTTCCACCAGAACCATCACCACTATTACCCCAACCATGGCCTTCATGCACCCTCTGCCTCTGACATCATTACCTTCCATCTTCTTGAATAA

>AT3G57230.1 | Symbols: AGL16 | AGAMOUS-like 16 | chr3:21177710-21180671 FORWARD LENGTH=723

ATGGGAAGGGGCAAGATCGCGATTAAGAGGATCAATAACTCTACGAGCCGTCAGGTTACGTTCTCGAAGCGAAGGAATGGATTGTTGAAGAAAGCTAAGGAGCTTGCGATTCTCTGCGATGCTGAGGTTGGTGTCATCATCTTCTCCAGCACCGGTAGGCTCTACGATTTCTCCAGCTCCAGCATGAAATCGGTCATAGAGAGATACAGCGATGCCAAAGGAGAAACCAGTTCAGAAAATGATCCCGCTTCAGAAATTCAGTTCTGGCAAAAGGAGGCTGCGATTCTAAAGCGTCAGCTACATAACTTGCAAGAAAACCACCGGCAAATGATGGGGGAGGAGCTCTCTGGACTAAGTGTAGAAGCTTTACAGAATTTGGAAAATCAGCTTGAATTGAGCCTTCGTGGCGTTCGAATGAAAAAGGATCAAATGTTAATCGAAGAAATACAAGTACTTAACCGAGAGGGGAATCTCGTTCACCAAGAGAATTTAGACCTCCACAAGAAAGTAAACCTAATGCACCAACAGAACATGGAACTACATGAAAAGGTTTCAGAGGTCGAGGGTGTGAAAATCGCAAACAAGAATTCTCTTCTCACAAATGGTCTAGACATGAGAGATACCTCGAACGAACATGTCCATCTTCAGCTCAGCCAACCGCAGCATGATCATGAGACGCATTCAAAAGCTATCCAACTCAACTATTTTTCCTTCATTGCATAA

>AT3G57390.1 | Symbols: AGL18 | AGAMOUS-like 18 | chr3:21233910-21235735 FORWARD LENGTH=771

ATGGGGAGAGGAAGGATTGAGATTAAGAAGATTGAGAATATCAACAGTCGTCAAGTCACTTTCTCTAAGAGACGAAACGGTTTGATCAAGAAGGCTAAAGAGCTTTCGATTCTCTGTGACGCCGAGGTTGCTCTTATCATCTTCTCCAGCACCGGCAAGATTTACGATTTCTCCAGCGTCTGTATGGAGCAAATTCTTTCTAGATATGGATACACTACTGCGTCCACTGAGCATAAACAACAAAGAGAACACCAACTTCTAATTTGTGCTTCACATGGAAATGAAGCTGTGTTGCGAAATGATGATTCTATGAAGGGGGAACTTGAAAGATTACAGCTTGCAATTGAGAGACTTAAGGGTAAGGAGCTTGAAGGTATGAGTTTCCCGGATCTTATTTCTCTTGAAAACCAGTTGAACGAGAGCTTGCATAGTGTCAAGGATCAAAAGACACAAATCCTGCTCAACCAGATTGAGAGATCCAGGATACAGGAGAAAAAAGCATTGGAAGAAAACCAAATCTTGCGCAAACAGGTTGAGATGTTGGGGAGAGGTTCAGGACCAAAAGTGTTGAATGAAAGGCCTCAAGATTCTAGCCCAGAAGCCGATCCCGAGAGCTCTTCATCAGAAGAGGATGAGAATGACAACGAGGAGCACCATTCCGACACTTCCTTGCAGTTGGGGTTGTCGTCGACGGGGTATTGCACAAAGAGAAAGAAGCCGAAGATCGAACTGGTCTGCGATAACTCTGGGAGTCAAGTGGCTTCTGATTGA

>AT3G58780.1 | Symbols: SHP1, AGL1 | SHATTERPROOF 1, AGAMOUS-like 1 | chr3:21739150-21741766 FORWARD LENGTH=747

ATGGAGGAAGGTGGGAGTAGTCACGACGCAGAGAGTAGCAAGAAACTAGGGAGAGGGAAAATAGAGATAAAGAGGATAGAGAACACAACAAATCGTCAAGTTACTTTCTGCAAACGACGCAATGGTCTTCTCAAGAAAGCTTATGAACTCTCTGTCTTGTGTGATGCCGAAGTTGCCCTCGTCATCTTCTCCACTCGTGGCCGTCTCTATGAGTACGCCAACAACAGTGTGAGGGGTACAATTGAAAGGTACAAGAAAGCTTGTTCCGATGCCGTCAACCCTCCTTCCGTCACCGAAGCTAATACTCAGTACTATCAGCAAGAAGCCTCTAAGCTTCGGAGGCAGATTCGAGATATTCAGAATTCAAATAGGCATATTGTTGGGGAATCACTTGGTTCCTTGAACTTCAAGGAACTCAAAAACCTAGAAGGACGTCTTGAAAAAGGAATCAGCCGTGTCCGCTCCAAAAAGAATGAGCTGTTAGTGGCAGAGATAGAGTATATGCAGAAGAGGGAAATGGAGTTGCAACACAATAACATGTACCTGCGAGCAAAGATAGCCGAAGGCGCCAGATTGAATCCGGACCAGCAGGAATCGAGTGTGATACAAGGGACGACAGTTTACGAATCCGGTGTATCTTCTCATGACCAGTCGCAGCATTATAATCGGAACTATATTCCGGTGAACCTTCTTGAACCGAATCAGCAATTCTCCGGCCAAGACCAACCTCCTCTTCAACTTGTGTAA

>AT3G61120.1 | Symbols: AGL13 | AGAMOUS-like 13 | chr3:22618414-22620466 REVERSE LENGTH=735

ATGGGAAGAGGCAAAGTGGAAGTGAAGAGAATTGAGAACAAGATCACTAGACAAGTAACCTTCTCCAAAAGAAAAAGTGGTTTGCTCAAGAAAGCCTACGAACTCTCTGTTCTTTGTGATGCAGAGGTTTCTCTCATAATCTTCTCCACCGGTGGCAAGCTCTACGAGTTCAGCAATGTCGGAGTTGGCAGAACCATTGAGAGGTACTATCGCTGTAAAGACAATCTTTTGGACAATGATACGCTTGAAGATACTCAGGGCTTGCGTCAAGAGGTGACAAAGCTCAAATGCAAATACGAATCTCTTCTTCGCACTCATAGGAATTTGGTTGGGGAAGATCTTGAAGGGATGAGTATAAAGGAATTGCAGACGCTGGAGAGACAGCTTGAAGGAGCTCTTTCCGCAACCCGAAAACAAAAGACGCAGGTTATGATGGAACAAATGGAAGAGCTTCGGAGAAAGGAGAGGGAGCTCGGAGATATAAACAATAAGCTAAAGCTTGAGACAGAAGATCATGATTTTAAAGGCTTTCAAGACCTACTGCTTAACCCGGTGCTCACCGCCGGTTGTTCCACTGATTTTTCCTTGCAATCAACTCATCAAAATTATATATCGGATTGCAACCTTGGATATTTTTTACAGATTGGGTTTCAACAACACTATGAGCAAGGTGAAGGATCTTCGGTGACAAAGAGTAATGCAAGAAGTGATGCTGAGACCAACTTTGTCCAATGA

>AT3G66656.1 | Symbols: AGL91 | AGAMOUS-like 91 | chr3:2091262-2091798 REVERSE LENGTH=537

ATGGGTAGGAGAAAGATTAAGATGGAGAAAGTGCAAGACACAAACACGAAGCAAGTTACCTTCTCAAAACGTAGGCTGGGTTTGTTCAAGAAAGCGAGCGAGCTTGCGACTCTGTGCAACGCGGAGGTTGGTATTGTTGTCTTTTCTCCAGGAAACAAACCGTATTCCTTCGGGAAACCGAATTTTGATGTGATTGCAGAACGGTTTAAGAATGAATTCGAAGAAGAAGAAGAAGGAGATAGCTGTGAAACATCAGGCTATAGTAGAGGCAATAGAGCTAGACAGGAGAAGAAGATATGTAAACGCCTCAACTCGATTACTGAAGAAGCTGAAGCTGAGAAGAAACATGGTGAAGATCTTCACAAGTGGCTTGAATCTGCTGAACAGGATAAGTTCAATAAGCCCATTGAGGAGCTTACGCTTGAGGAACTCAAGGAATTTGAGGCTAAGATTAAGAAAATAAGCTGTGGGATCCAAAGTAACATTAGTCATATGCAGGCTTCGTCTTCTCTCATGTTTCTCTCTAATGATAATTAG

>AT4G02235.1 | Symbols: AGL51 | AGAMOUS-like 51 | chr4:980955-981711 FORWARD LENGTH=591

ATGAAGCAATCTTCTTTTTCTTCTTCTTCTTCGTCACGTAATTCAACTAGTTTAACAAACAGACTCAAAACCATCTTCAAAAAAGCTGAAGAGCTTTCGATTCTCTGTGCTATTGACGTTTGCGTCATCTATTACGGACCAGACGGTGAACTGAGAACATGGCCTAAGGAGAGAAATACAGTGAAAGACATGGCTTCGAGATATAAAGAGGCCACAAAACGCAAGAAGAAACGGACTCTTTCTACTCTGCAAGAAAGGCTTCGTATTGTTGAGTCGCAGAAACAACAAAACAAAAACTTGGTGCATCAGAGTTTAACACCATCGTATCTGAACCAGATCCAACACTTGAATCCTAGCAACTTCTCGCCGTATATGTACAACCATGGAGATGCTGCTACTCTCTCACAACTCCCACTGTCTGCTTCACTCTCCAATCAACTCCAATTACCTGAATCACTTGATGCGGCATGGTTTTGGTCAGAACATGTGTTTGGACAACATCACCAACAACAACAACTTTCAACATCCTGGCGTGTCAAACACACAAGAATACTCACCGTTTCTTTCGGTACAAGCATCTGCAGTGAATAA

>AT4G09960.1 | Symbols: STK, AGL11 | AGAMOUS-like 11, SEEDSTICK | chr4:6236713-6239409 REVERSE LENGTH=693

ATGGGAAGAGGAAAGATAGAAATAAAGAGGATAGAGAACTCAACAAATCGACAAGTGACGTTTTGCAAAAGAAGAAATGGACTTCTGAAGAAAGCCTATGAGCTTTCGGTCCTTTGCGATGCAGAAGTTGCGCTCATTGTTTTCTCCACTCGTGGCCGTCTCTATGAATACGCCAATAACAACATAAGATCAACCATTGAGAGGTACAAGAAAGCTTGTTCTGATAGCACCAACACTAGCACTGTCCAAGAAATCAATGCCGCGTACTATCAACAAGAATCTGCTAAGCTGAGACAACAGATCCAAACGATTCAAAACTCCAACAGGAATCTGATGGGAGACTCTTTGAGTTCCTTAAGTGTCAAGGAACTAAAACAAGTTGAGAATCGCCTTGAGAAAGCTATCTCTAGGATCAGGTCCAAGAAGCATGAGTTGCTTTTAGTTGAAATCGAAAACGCGCAGAAAAGGGAGATTGAGCTTGACAATGAGAACATCTATCTAAGAACTAAGGTAGCAGAAGTGGAGAGGTATCAACAACACCATCATCAAATGGTTAGTGGTTCAGAGATTAATGCAATTGAAGCTTTAGCCTCACGCAATTACTTTGCTCATAGCATTATGACTGCTGGTTCTGGATCTGGTAATGGAGGTTCTTACTCTGATCCCGACAAGAAAATTCTTCATCTCGGATAA

>AT4G11250.1 | Symbols: AGL52 | AGAMOUS-like 52 | chr4:6849578-6850567 FORWARD LENGTH=990

ATGAAGCAAGCTTCTTCTTCTTCGTCATGTAATCCAACAAGTTTAACAAACAGACTTAAAACAATCTTTAAGAAAGCAGAAGAGCTTTCGATTCTCTGTGCTATTGACGTTTGCGTCATCTATTACGGACCAGACGGTGATCTGAGAACATGGCCTAAGGATAGAGAGACAGTGAAAAATATGGCTTTGAGGTATAAAGAGGACAGAAAACGCAAGAAGTGCCTCAATCTTCATGAGTTCCTCGAGAAAGAGAAGGTGAAGGACAAAGACAAGTATAAGGGGAAGACGAATTATGTGAAGAACCCTAATTGGTATCCAAACTTTGATCATTACTCTCCTCAACAACTCTCTCAATTGATTCAGTCCTTAGAACGAACACTTTCTACTCTCCAAAAAAGGCTTCGTATTGTTGAGTCGCAGAAAAAACAGAACACAAACTTGGTGCATCAGAGTTTAACACCATCGTATCTGAACCAGACACAACACTTGGATCCTAGCAAGTTCTCGCTGTATATGTACAACCATGGAGATGCTACTCTCTCACAACTCCCACTGTCTGCTTCACAATCCAATCAACTCATCAATTACCAGATGCAGCATGGTTTTGGTCAGAACATGTGTTTGGACAACATCACCAACAACAACAACTTTCAACATCCTGGCGTGTCAAACACACAAGACTACTCACCGTTACTTTCGGCGAATAACTACGGGTTGAATAATCACTTGATGCAGCAACAGGATCAGCTTCATGGTTTTGATCAGAACTTGTGTATGATGAGTGAAATCATCAATAACAACAACGGTTTACAACATCCTAATCTCTCAAACACTGTTCCACATGAATTCCCTTATGGTAACACTAGCTTCTCTCAAGATATGTTTTCGAGCTATGATGGGAGCAGTTTGCTACAGACATCTTCTCTGCCACCTCTCCACAACATTCCTAACAGCTATTGTTTTTCTGACAACTCAAGACTTCTCTGCTAA

>AT4G11880.1 | Symbols: AGL14, XAL2 | XAANTAL2, AGAMOUS-like 14 | chr4:7143512-7147108 FORWARD LENGTH=666

ATGGTGAGGGGAAAGACAGAGATGAAGAGGATAGAGAACGCAACGAGCAGGCAAGTGACTTTCTCAAAAAGAAGAAATGGACTTTTGAAGAAAGCTTTTGAATTATCAGTCCTTTGTGATGCTGAAGTTGCCCTAATCATCTTCTCTCCTAGAGGCAAACTCTATGAGTTCTCTAGCTCCTCCAGTATACCTAAAACAGTAGAAAGATATCAAAAGCGAATACAAGATCTCGGGTCTAACCATAAGAGAAATGATAATTCACAGCAATCGAAGGACGAAACCTATGGCTTGGCGAGAAAGATCGAACATTTGGAGATTTCGACACGAAAAATGATGGGAGAAGGACTTGACGCATCTTCTATAGAAGAGTTACAACAATTGGAGAACCAGTTGGACAGAAGCTTAATGAAAATAAGAGCCAAAAAGTACCAGTTATTACGAGAAGAAACTGAGAAATTAAAAGAAAAGGAGAGGAACCTCATTGCAGAAAATAAAATGCTGATGGAGAAGTGTGAGATGCAAGGAAGAGGAATAATAGGAAGAATATCATCATCATCATCAACATCAGAACTGGATATAGATGACAATGAAATGGAAGTGGTGACTGATTTGTTCATTGGACCTCCTGAGACTCGACACTTCAAAAAGTTTCCTCCTTCAAACTAA

>AT4G18960.1 | Symbols: AG | AGAMOUS | chr4:10383917-10388272 FORWARD LENGTH=759

ACGGCGTACCAATCGGAGCTAGGAGGAGATTCCTCTCCCTTGAGGAAATCTGGGAGAGGAAAGATCGAAATCAAACGGATCGAGAACACAACGAATCGTCAAGTCACTTTTTGCAAACGTAGAAATGGTTTGCTCAAGAAAGCTTACGAGCTCTCTGTTCTTTGTGATGCTGAAGTCGCACTCATCGTCTTCTCTAGCCGTGGTCGTCTCTATGAGTACTCTAACAACAGTGTAAAAGGGACTATTGAGAGGTACAAGAAGGCAATATCGGACAATTCTAACACCGGATCGGTGGCAGAAATTAATGCACAGTATTATCAACAAGAATCAGCCAAATTGCGTCAACAAATAATCAGCATACAAAACTCCAACAGGCAATTGATGGGTGAGACGATAGGGTCAATGTCTCCCAAAGAGCTCAGGAACTTGGAAGGCAGATTAGAGAGAAGTATTACCCGAATCCGATCCAAGAAGAATGAGCTCTTATTTTCTGAAATCGACTACATGCAGAAAAGAGAAGTTGATTTGCATAACGATAACCAGATTCTTCGTGCAAAGATAGCTGAAAATGAGAGGAACAATCCGAGTATAAGTCTAATGCCAGGAGGATCTAACTACGAGCAGCTTATGCCACCACCTCAAACGCAATCTCAACCGTTTGATTCACGGAATTATTTCCAAGTCGCGGCATTGCAACCTAACAATCACCATTACTCATCCGCGGGTCGCCAAGACCAAACCGCTCTCCAGTTAGTGTAA

>AT4G22950.1 | Symbols: GL19, AGL19 | AGAMOUS-like 19 | chr4:12023946-12027421 REVERSE LENGTH=660

ATGGTGAGGGGCAAAACGGAGATGAAGAGGATAGAGAACGCAACAAGCAGGCAAGTGACGTTTTCGAAGAGAAGAAATGGACTCTTGAAGAAAGCCTTCGAATTATCGGTCCTTTGTGATGCTGAAGTTGCTTTGGTTATCTTCTCTCCAAGATCCAAACTCTATGAGTTCTCTAGCTCTAGTATAGCAGCAACAATTGAACGCTATCAGAGACGAATAAAAGAAATTGGGAATAATCATAAGAGAAATGATAATTCTCAGCAAGCGAGAGACGAAACATCTGGATTGACAAAAAAGATTGAACAGCTAGAGATATCTAAACGAAAATTGCTTGGAGAAGGCATTGATGCATGTTCCATCGAGGAGCTGCAACAGTTAGAGAATCAGTTGGACCGAAGCTTGAGCAGGATAAGAGCCAAGAAGTACCAATTACTCCGTGAAGAAATTGAGAAGTTGAAGGCAGAGGAGAGGAATCTCGTTAAGGAAAATAAAGATCTGAAGGAGAAGTGGCTTGGAATGGGAACAGCAACAATAGCATCATCACAATCAACGTTATCATCATCAGAAGTGAACATAGATGACAATATGGAAGTGGAGACTGGTTTGTTCATTGGACCTCCTGAGACAAGACAATCCAAAAAATTCCCTCCTCAAAATTAA

>AT4G24540.1 | Symbols: AGL24 | AGAMOUS-like 24 | chr4:12671160-12673645 REVERSE LENGTH=663

ATGGCGAGAGAGAAGATAAGGATAAAGAAGATTGATAACATAACAGCGAGACAAGTTACTTTCTCAAAGAGAAGAAGAGGAATCTTCAAGAAAGCCGATGAACTTTCAGTTCTTTGCGATGCTGATGTTGCTCTCATCATCTTCTCTGCCACCGGAAAGCTCTTCGAGTTCTCCAGCTCAAGAATGAGAGACATATTGGGAAGGTATAGTCTTCATGCAAGTAACATCAACAAATTGATGGATCCACCTTCTACTCATCTCCGGCTTGAGAATTGTAACCTCTCCAGACTAAGTAAGGAAGTCGAAGACAAAACCAAGCAGCTACGGAAACTGAGAGGAGAGGATCTTGATGGATTGAACTTAGAAGAGTTGCAGCGGCTGGAGAAACTACTTGAATCCGGACTTAGCCGTGTGTCTGAAAAGAAGGGCGAGTGTGTGATGAGCCAAATTTTCTCACTTGAGAAACGGGGATCGGAATTGGTGGATGAGAATAAGAGACTGAGGGATAAACTAGAGACGTTGGAAAGGGCAAAACTGACGACGCTTAAAGAGGCTTTGGAGACAGAGTCGGTGACCACAAATGTGTCAAGCTACGACAGTGGAACTCCCCTTGAGGATGACTCCGACACTTCCCTGAAGCTTGGGCTTCCATCTTGGGAATGA

>AT4G36590.1 | Symbols: AGL40 | agamous-like 40 | chr4:17261146-17262189 REVERSE LENGTH=747

ATGGTGAGAAGTACCAAAGGTCGTCAGAAAATAGAGATGAAAAAAATGGAAAACGAAAGCAACCTTCAGGTTACTTTCTCAAAAAGAAGATTCGGTCTTTTCAAAAAAGCTAGTGAACTTTGCACATTAAGTGGTGCAGAGATTCTGTTGATTGTGTTCTCTCCTGGTGGGAAAGTGTTTTCTTTTGGCCATCCAAGTGTTCAAGAACTCATTCATCGCTTTTCGAATCCTAACCATAATTCTGCCATTGTCCATCATCAGAACAACAATCTCCAACTTGTTGAAACCCGTCCGGATAGAAATATCCAATATCTCAACAATATACTCACTGAGGTGCTGGCAAACCAGGAAAAGGAGAAACAGAAGAGAATGGTTTTGGACCTATTGAAAGAATCCAGAGAACAAGTAGGAAACTGGTATGAAAAAGATGTGAAAGATCTCGACATGAATGAAACCAACCAGCTGATATCTGCTCTTCAAGATGTGAAAAAGAAACTGGTAAGAGAAATGTCTCAATATTCTCAAGTAAATGTTTCGCAGAATTACTTTGGTCAAAGTTCTGGCGTGATTGGTGGTGGTAATGTTGGCATTGATCTTTTTGATCAAAGAAGAAATGCATTCAACTATAATCCAAACATGGTGTTTCCCAATCATACACCACCAATGTTTGGATACAACAATGATGGAGTTCTCGTTCCGATATCCAACATGAACTACATGTCAAGTTACAACTTCAACCAGAGCTAG

>AT4G37940.1 | Symbols: AGL21 | AGAMOUS-like 21 | chr4:17835695-17838621 REVERSE LENGTH=687

ATGGGAAGAGGGAAGATTGTGATCCAAAGGATCGATGATTCAACGAGTAGACAAGTCACTTTCTCCAAACGAAGAAAGGGCCTTATCAAGAAAGCCAAAGAGCTAGCTATTCTCTGTGATGCCGAGGTCGGTCTCATCATCTTCTCTAGCACCGGAAAGCTCTATGACTTTGCAAGCTCCAGCATGAAGTCGGTTATTGATAGATACAACAAGAGCAAGATCGAGCAACAACAACTATTGAACCCCGCATCAGAAGTCAAGTTTTGGCAGAGAGAAGCTGCTGTTCTAAGACAAGAACTGCATGCTTTGCAAGAAAATCATCGGCAAATGATGGGAGAACAGCTAAATGGTTTAAGTGTTAACGAGCTAAACAGTCTTGAGAATCAAATTGAGATAAGTTTGCGTGGAATTCGTATGAGAAAGGAACAACTGTTGACTCAAGAAATCCAAGAACTAAGCCAAAAGAGGAATCTTATTCATCAGGAAAACCTCGATTTATCTAGGAAAGTACAACGGATTCATCAAGAAAATGTGGAGCTCTACAAGAAGGCTTATATGGCAAACACAAACGGGTTTACACACCGTGAAGTAGCTGTTGCGGATGATGAATCACACACTCAGATTCGGCTGCAACTAAGCCAGCCTGAACATTCCGATTATGACACTCCACCAAGAGCAAACGAATAA

>AT5G04640.1 | Symbols: AGL99 | AGAMOUS-like 99 | chr5:1332825-1333793 FORWARD LENGTH=969

ATGGGGGGCGTGAAGAGAAAGATTTCTATAGAGCTGATAGAGAAGAAAGATTCAAGAGCTGTTGCTTTCTCAAAACGCAGTAGGGGTCTATATAGCAAAGCCTCTGATCTTTGTCTTCTCTCCGATGCACAAATTGCGATCATAGCCACTCCGGTCTCTTCCAAATCAAACGTTTCTTTCTACACCTTTGGCCATTCCTCTGTTGATAACGTTGTAGCCGCTTTCCTCACGAATCAGCGTCCTCGGGAAGGTCTAGGGTTAGACTATTGGTGGGAAGATGAGAGGCTTTCAAAATCAGAGGACCTGGAGGAACTGAGAGACGCAATGGACTCGATGTCGAAGATGTTGAAAGATCTGAAGGATTTGCAGAATCAACGAGATTGTGAGGAGGACGTGAAGAAGAAGGGTGTTTTACACGGAACTCATCAAAAGCAAACCTTTAATCCTGAATCATGTTCTGTGAATTTCGATGGGTTTAACAAGAACACTGAAGAATTTGATCTTGATGAAATATTTGATTATGTGTCAACAGCTGAAGCTCTATCGATGAACTTGGACATGGACGATGTCTCTGTGGTGACCACGAATCAAAACCCCGTCTCTGCTTCTGAAACTGTTGAAGATAGAGAATTGGTTGTTCACAAAAATATGGATGAGGACAATATCCACGTGTCTGATATGGACGACAAGGATACGATGCTAATGATTTCTGACAAGAACAATGTTTTACCTGAAAACTTGGATGAATTCGATCAAGAGCTAGATCTTGATCAGCTACTTGATTTTGAGACAAACTATGAAAGCCTTTTGAAGAGTTGTGAGATGGAGGATTATGCCTCGATGGTGACTACAAAGCAAAATCTGTGCTCAAATCCTGAAGCTGTTGAAGATGGAGGATTAATGATTCAAAAAGATTTACCGGAGGATAATCTCTGCTTTTCTGATTATTTCAGTGACTTACATTGCTGA

>AT5G06500.1 | Symbols: AGL96 | AGAMOUS-like 96 | chr5:1982444-1983172 FORWARD LENGTH=729

ATGGCTCGGAAAAAAGTACGAGCTGCATGGATCCGTGACGATAGGATGAGGAGGGCAAGCCTCAAGAGGAGGTTAACGGGGCTTATCAAAAAAGTGAATGAACTGTCCATTCTGTGCGATATGCGCGCTAGTGTGGTCGTTTTCAATCGAGAAGAAGAACAATTGACGGCGTGGCCATCTCCCGAGGCAGCTAATTCACTCATCGACAACTTTTATTCTCTAACCGACCATGAAAGGACCATGAAGGCCGTTGATCCGGAGTCATACGTCCAGACCGTTATCGAAAAGATTGAGAAGAAACGAGCCGATACTCGGAAGGTTATCACGGAGTTTGAGATGGACGAACTCATGTTCCAAGTCCAAAATGGTCGTGAACTTGCTGATCTCTCCCCAACCGAGGCTGATAAACTAATACCATATGCGGATAAGAAACTTATGTGGTTGAGTAAACGGATGGGTTCTACGGGGGTCGACGCCCTGAGGGCTTCGAATGTTGCGTCTGGGTCAGGTGGAAATGGGTTAAACATGATGGAAACTGGAAGAAGTTTCTATTATGTTGATAAATGGGTCTTCGTTGATCCACAAGTCCAAAACCCTTGTGATGTTGAGACTCATTTACCCACAATGGTCAGTGGCTTAGACCTAAACATGGAACCATCTGATGAAGACTTGGGGACCTATAAAGGAGAGAGCAGCATGGCCGGTGGTGCTGAAGACGATGCCGAATGA

>AT5G10140.1 | Symbols: FLC, RSB6, AGL25, FLF | AGAMOUS-like 25, FLOWERING LOCUS F, REDUCED STEM BRANCHING 6, FLOWERING LOCUS C | chr5:3173724-3179339 REVERSE LENGTH=591

ATGGGAAGAAAAAAACTAGAAATCAAGCGAATTGAGAACAAAAGTAGCCGACAAGTCACCTTCTCCAAACGTCGCAACGGTCTCATCGAGAAAGCTCGTCAGCTTTCTGTTCTCTGTGACGCATCCGTCGCTCTTCTCGTCGTCTCCGCCTCCGGCAAGCTCTACAGCTTCTCCTCCGGCGATAACCTGGTCAAGATCCTTGATCGATATGGGAAACAGCATGCTGATGATCTTAAAGCCTTGGATCATCAGTCAAAAGCTCTGAACTATGGTTCACACTATGAGCTACTTGAACTTGTGGATAGCAAGCTTGTGGGATCAAATGTCAAAAATGTGAGTATCGATGCTCTTGTTCAACTGGAGGAACACCTTGAGACTGCCCTCTCCGTGACTAGAGCCAAGAAGACCGAACTCATGTTGAAGCTTGTTGAGAATCTTAAAGAAAAGGAGAAAATGCTGAAAGAAGAGAACCAGGTTTTGGCTAGCCAGATGGAGAATAATCATCATGTGGGAGCAGAAGCTGAGATGGAGATGTCACCTGCTGGACAAATCTCCGACAATCTTCCGGTGACTCTCCCACTACTTAATTAG

>AT5G13790.1 | Symbols: AGL15 | AGAMOUS-like 15 | chr5:4449128-4450802 REVERSE LENGTH=807

ATGGGTCGTGGAAAAATCGAGATAAAGAGGATCGAGAATGCGAATAGCAGACAAGTCACTTTTTCCAAGAGGCGTTCTGGGTTACTTAAGAAAGCTCGTGAGCTCTCTGTTCTTTGTGATGCTGAAGTTGCTGTCATCGTCTTCTCTAAGTCTGGCAAGCTCTTCGAGTACTCCAGTACTGGAATGAAGCAAACACTTTCCAGATACGGTAATCACCAGAGTTCTTCAGCTTCTAAAGCAGAGGAGGATTGTGCAGAGGTGGATATTTTAAAGGATCAACTTTCAAAGCTTCAAGAGAAACATTTACAACTGCAGGGCAAGGGCTTGAATCCTCTGACCTTTAAAGAGCTGCAAAGCCTTGAGCAGCAACTATATCATGCATTGATTACTGTCAGAGAGCGAAAGGAACGATTGCTGACTAACCAACTTGAAGAATCACGCCTCAAGGAACAACGAGCAGAGTTGGAAAACGAGACCTTGCGTAGACAGGTTCAAGAACTGAGGAGCTTTCTCCCGTCGTTCACCCACTATGTTCCATCCTACATCAAATGCTTTGCTATAGATCCAAAGAACGCTCTCATAAACCACGACAGTAAATGCAGCCTCCAGAACACCGATTCAGACACAACTTTGCAATTAGGGTTGCCGGGAGAGGCACATGATAGAAGGACGAATGAAGGAGAAAGAGAGAGCCCGTCAAGCGATTCAGTGACAACAAACACGAGCAGCGAAACTGCAGAAAGAGGGGATCAGTCTAGTTTAGCAAATTCTCCACCTGAAGCCAAAAGACAAAGGTTCTCTGTTTAG

>AT5G15800.1 | Symbols: AGL2, SEP1 | SEPALLATA1, AGAMOUS-like 2 | chr5:5151594-5153767 REVERSE LENGTH=756

ATGGGAAGAGGAAGAGTAGAGCTGAAGAGGATAGAGAACAAAATCAACAGACAAGTAACGTTTGCAAAGCGTAGGAACGGTTTGTTGAAGAAAGCTTATGAATTGTCTGTTCTCTGTGATGCTGAAGTTGCTCTCATCATCTTCTCCAACCGTGGAAAGCTCTATGAGTTTTGCAGCTCCTCAAACATGCTCAAGACACTTGATCGGTACCAGAAATGCAGCTATGGATCCATTGAAGTCAACAACAAACCTGCCAAAGAACTTGAGAACAGCTACAGAGAATATCTGAAGCTTAAGGGTAGATATGAGAACCTTCAACGTCAACAGAGAAATCTTCTTGGGGAGGATTTAGGACCTTTGAATTCAAAGGAGTTAGAGCAGCTTGAGCGTCAACTGGACGGCTCTCTCAAGCAAGTTCGGTCCATCAAGACACAGTACATGCTTGACCAGCTCTCGGATCTTCAAAATAAAGAGCAAATGTTGCTTGAAACCAATAGAGCTTTGGCAATGAAGCTGGATGATATGATTGGTGTGAGAAGTCATCATATGGGAGGAGGAGGAGGATGGGAAGGTGGTGAACAGAATGTTACCTACGCGCATCATCAAGCTCAGTCTCAGGGACTATACCAGCCTCTTGAATGCAATCCAACTCTGCAAATGGGGTATGATAATCCGGTATGCTCAGAGCAAATAACTGCGACAACCCAAGCTCAGGCGCAGCAGGGAAACGGTTACATCCCGGGGTGGATGCTCTGA

>AT5G20240.1 | Symbols: PI | PISTILLATA | chr5:6829203-6831208 FORWARD LENGTH=627

ATGGGTAGAGGAAAGATCGAGATAAAGAGGATAGAGAACGCAAACAACAGAGTGGTGACGTTCTCAAAGAGGAGGAATGGATTGGTGAAGAAGGCTAAAGAGATCACAGTTCTTTGTGATGCAAAAGTTGCCCTCATAATCTTTGCAAGTAATGGTAAGATGATTGATTACTGTTGTCCTTCCATGGATCTTGGTGCTATGTTGGACCAATACCAGAAGTTATCTGGCAAGAAACTATGGGATGCTAAGCATGAGAACCTTAGCAATGAGATTGATAGGATCAAGAAAGAGAATGATAGCTTACAACTGGAGCTCAGGCATTTGAAGGGAGAAGATATACAGTCTCTCAACTTGAAAAATCTGATGGCTGTCGAGCACGCCATTGAACATGGCCTCGACAAAGTCCGAGACCACCAGATGGAGATCCTTATATCAAAGAGGAGAAATGAGAAGATGATGGCGGAGGAGCAACGGCAACTCACTTTCCAGCTGCAACAACAGGAGATGGCTATAGCAAGCAACGCAAGAGGAATGATGATGAGAGATCATGATGGGCAGTTTGGATATAGAGTGCAACCGATTCAGCCAAATCTTCAGGAAAAGATTATGTCTTTGGTCATCGATTGA

>AT5G23260.1 | Symbols: TT16, ABS, AGL32 | TRANSPARENT TESTA16, ARABIDOPSIS BSISTER, AGAMOUS-like 32 | chr5:7836294-7838340 FORWARD LENGTH=792

ATGAATATTGAGGAAGAGGGAGCCACTCATAAGAGGAAGAAGAGAGAGATGGGTAGAGGGAAGATAGAGATAAAGAAGATAGAGAATCAGACGGCGAGGCAAGTGACCTTCTCCAAGAGAAGAACTGGTCTTATAAAGAAGACTCGTGAGCTCTCTATTCTCTGTGACGCTCACATCGGTCTCATCGTCTTCTCAGCCACCGGAAAGCTTTCCGAGTTCTGCTCCGAACAGAACAGGATGCCTCAACTCATTGACCGATACTTGCATACCAACGGATTGCGACTTCCTGATCATCATGACGACCAGGAGCAATTGCACCATGAGATGGAACTACTAAGAAGAGAGACATGTAACCTTGAGCTTCGTCTGCGTCCATTCCATGGACATGACTTAGCCTCCATTCCTCCTAATGAGCTTGACGGACTCGAGAGACAGCTAGAACATTCTGTCCTCAAAGTCCGTGAGCGTAAGCAACAGTTGGAGAATCTAAGCAGAAAGAGGAGGATGCTAGAAGAAGATAACAACAACATGTACCGTTGGCTTCATGAGCATCGTGCAGCGATGGAGTTTCAACAAGCTGGGATAGATACCAAACCAGGGGAGTATCAACAGTTTATAGAGCAGCTTCAGTGCTATAAACCAGGGGAGTATCAGCAGTTTCTAGAGCAGCAGCAACAACAACCAAACAGCGTTCTTCAGCTTGCTACACTTCCTTCTGAGATTGATCCTACTTACAATCTCCAGCTTGCTCAGCCTAATCTTCAAAACGATCCAACGGCCCAGAATGATTAA

>AT5G26580.1 | Symbols: AGL34 | AGAMOUS-like-34 | chr5:9393065-9394102 REVERSE LENGTH=1038

ATGGGGATGAAGAAGGTAAAGCTATCTTTGATAGCTAATGAAATATCAAGGGAAACATCCTTCATGAAGAGGAAAAATGGGATAATGAAGAAACTCTACGAGTTGTCAACTCTATGTGGTGTCCAAGCTTGTACTCTCATCTACAGTCCATTCATTCCGGTTCCAGAGTTTCTGGAGATGTCGCCGACAGCCCGAACAAGGAAGATGATGAATCAAGAAACGTATCTAATGGAGAGGATTACCAAAGCAAAAGAGCAACTACAGAACCTGGTTGGTGCGAACCAAGAGTTACAGGTTAGACGATTTATGTTTGATTGTGTTGAAGGCAAAATGTCGCAGTATCGTTATGATGCAAAAGACCTTCAAGATTTGTTATCTTGTATAAATCTATATCTCGATCAGCTTAACGGAAGGATCGAGATCCTTAAAGAACACGGTGACTCGTTGCCTTCCGTCTCTCCTTTTCCTACTAGAATTGGTGTTGAAGAAACTGGTGATGAGTCGTCTTCCGACTCTCCTATTCTTGCTACAACTGGGGTTGTAGATACTCCTAATGCTACAAATCCTCGTGTTCTTGTGGCCGATACGACTCATTTTCTTGATGCGAATGCAACTGCGGTAACTGCTCCCTTTGGATTTTCTAATCATATTCAATATAAGAATATGAATATGAGTCAAGATCTGCATCGACCGTTTCAACACCTTGTTCCTACTAACTTTTGTGATTTTTTTCAAAATCAGAATATGAATCAGGTTCAATACCAGGCTCCTCCTAATGATATGTTTAATCAGATTCAACGAGAGTTCTACAACATTAATTTGAATCAGAAATCAAATCAGTATATGAATCAACAACAACCATTCATGAATCCGATGGTGGAACAACATATGAGTCATGTTGGAGGGCGTGAGAGCATTCCTTTCATGGACGGAAACTACTACAACTACAATCAACTACCAGTCGTTGATCATGGTTCCACAAGTTACATGCCTTCCACCACCGGTGTTTATGATCCTTACTTCAACAATAATCTCTAA

>AT5G26630.1 | Symbols: AGL35 | agamous-like 35 | chr5:9350815-9351471 FORWARD LENGTH=657

ATGACGAGACAGAAAGTGAAAATGACTTTCATAGAAAATGAAACAGCAAGAAAATCAACATTCAAGAAAAGAAAGAAAGGTCTTTTGAAGAAAGCTCAAGAGTTGGGAATTCTTTGTGGCGTCCCGATCTTTGCTGTCGTCAACAGTCCGTACGAGCTGAACCCGGAGGTGTGGCCATCGAGAGAGGCTGCGAACCAGGTGGTGTCCCAATGGAAGACGATGTCGGTGATGGACAAGACCAAGAAGATGGTGAACCAAGAGACCTTTCTCCAACAGAGGATCACCAAAGCAACCGAGTCTTGGAAGAAGTTGCGGAAAGAGAACAAAGAGTTGGAGATGAAGAACATTATGTTTGATTGTCTTAGTGGCAAAACTTTGGTTTCTAGTATCGAAAAAACTGAGCTTCGAGATTTTGGTTATGTCATTGAACAACAACTCAAAGATGTTAATCGTAGGATCGAGATTCTGAAAAGGAACAATGAACCATCTTCCGCCCTCGTTCCTGTTGCTGCCCCTACAACATCTAGTGTCATGCCTGTGGTTGAGATGGGTTCTTCTTCGGTTGGATTTTACGACAAGGTTCGAGATCAAATTCAAATTACTTTGAATATGAAACAGACCACAAATGATTTGGATCTGAACAAGAAACAGTGGTGA

>AT5G26650.1 | Symbols: AGL36 | AGAMOUS-like 36 | chr5:9343785-9344885 FORWARD LENGTH=1101

ATGAAGAAGGTGAAGCTATCTTTGATAGCTAATGAAAGATCAAGGAAAACATCCTTCATAAAGAGGAAAGACGGGATTTTTAAGAAACTCCACGAGTTGTCAACTCTGTGTGGTGTCCAAGCTTGTGCTCTCATCTACAGTCCATTCATACCGGTTCCAGAGTCATGGCCGTCAAGGGAAGGTGCTAAAAAGGTGGCTTCAAGGTTTCTGGAGATGCCGCCGACAGCCCGAACCAAGAAGATGATGGATCAAGAGACTTACCTTATGGAGAGGATTACCAAAGCAAAAGAGCAACTAAAGAACCTGGCTGCTGAGAACCGAGAGTTACAGGTTAGACGATTTATGTTTGATTGTGTTGAAGGCAAAATGTCCCAGTATCATTATGATGCAAAAGACCTTCAAGATTTGCAATCTTGTATAAATCTATATCTCGATCAGCTTAACGGAAGGATCGAGTCCATTAAAGAAAATGGTGAGTCGTTGTTGTCTTCCGTCTCTCCTTTTCCTACTAGAATTGGTGTTGACGAAATTGGTGATGAGTCATTTTCCGACTCTCCTATTCATGCTACAACTGGGGTTGTAGATACTCTTAATGCTACCAATCCTCATGTTCTTACGGGCGATATGACTCCTTTTCTTGATGCGGACGCAACTGCGGTAACTGCTTCCAGTAGATTTTTTGATCATATTCCATATGAAAATATGAATATGAGTCAAAATCTGCATGAACCGTTTCAACACCTTGTTCCTACTAACGTTTGTGATTTTTTTCAAAATCAGAATATGAATCAGGTTCAATACCAGGCTCCTAATAATCTGTTTAATCAGATTCAACGAGAATTCTACAACATAAATTTGAATCTGAATTTGAATCTGAATTCGAATCAGTATCTGAATCAACAACAATCATTCATGAATCCGATGGTGGAACAACATATGAATCATGTTGGAGGGCGTGAAAGCATTCCTTTCGTGGACGGAAACTGCTACAACTACCATCAACTACCATCCAATCAACTACCAGCCGTTGATCATGCTTCCACCAGTTACATGCCTTCCACCACCGGTGTCTATGATCCTTACATCAACAATAATCTCTAA

>AT5G26880.1 | Symbols: AGL26 | AGAMOUS-like 26 | chr5:9457950-9459190 REVERSE LENGTH=792

ATGGAGAGTTGCTGTCGGAGCGTCATAGCTTCAAGGACGTTTCATCTGCGGTCAAGTGGCCGTCTATTTCCCTCTCTTAGCTTAACTCATCTCAAGGGCAAGCTATCTCTCTCCATCAACTCGTTTTCCTCCAAGATTCAGTCTCATGCTCTCCGAGGAGTTGGTATAGGCGAGTCAGATAAGAAAAATCCCCTGCCGCGAGGGGCCGGAGAAGGAGTCAAGGAAGATGCGAGGAGTAAGCTGCTTCATGTAGTTTTGGTCTCTCCTCAGATTCCTGGGAATACAGGTTGCATTGCAAGAACATGTGCTGCCTCAGCTGTTGGTCTGCATTTAGTCGGGCCATTAGGTTTTCAAGTGGATGACGCCAGAGTTAAGCGAGCTGGTTTGGATTATTGGCCCTTTGTGGTTGTCAAAGCGCATAGCTCATGGGCTGAGTTTCAAGAATATTTCAGGCTTCAGGAGGGAGAAAAACGAATGATAGCTTTTACAAAAAGAGGAACAAGGATACATTCAGATTTTTCTTACCGATCAGGCGATTACCTCTTGTTCGGGTCAGAGACAAGCGGTCTACCTCCTGAAGCGCTGTCAGACTGCAATCACGAACCATATGGAGGGGGGACCCTACGTATTCCCATGGTAGAAACGTATGTGAGATGTCTGAATCTTTCGGTGAGTGTAGGGATTGCTCTGTATGAAGCGTCCAGACAACTTAACTATGAGCAAATCGAGTGTGCACCTCAAGGCTGTGTGAATGGTGAAGAACCATTGTTGACAGAGGATATCTTTGCTTGA

>AT5G26950.1 | Symbols: AGL93 | AGAMOUS-like 93 | chr5:9483251-9484120 REVERSE LENGTH=870

ATGGATTCTTCAATGTCGACGAAGAAGAAAACCAAATTGTCTGTGAGAAACCAAACTTGTTTCAAGAAATCGTCCCTGTCTTCTTCTTCCACTGCAAAAAAGACCACGAATTTGTCTATGAGAGAACAGACCATGTTTAAGAAAGCCCTCGAGCTTTCGACTCTGTGCAACATCGATGTCTGTGTCATATACTATGGTCGTGACGGAAAACTCATCAAGACATGGCCGGATGATCAATCAAAAGTTCGAGACATGGCTGAGAGGTTTAGCAGATTACACGAGAGAGAGCGATGCAAGAAAAGAACCAACCTTTCTCTGTTTCTACGTAAGAAGATCCTCGACGACACTAAATTGTCGGAGAAAGTCTTAGAGATGGAGGATTCGTTAGAAAGTGGTCTACGAGTACTACAAGATAAGCTTCTGTTACTCCAACCCGAGAAAAACCAGACCGAGTTTGGTCAGACCCGTGCGGTTTCTTCCACAACGAATCCATTGTCTCCTCCTCCTTCGCTAATTGAGGATCATCGTCATCAACAACGGACAGAACCATTGATGAGTGGTGTGTCAAACACAGAGCAAGACCTATCGACGTCATCATTGAGTCAAAATCAGAGCAAATTTTCTGTCTTTCTCTATAACCATGACAACTGTAGCTTCTATCAAGTACCTGACTCTGTTTCTAGCTTTGACAGTTTGACGAGTACTGGTTTACTTGGGGAACAAGGATCTGGTCTGGGAAGTAGCTTTGATCTTCCCATGGTTTTTCCTCCTCAGATGCAGACACAAACCCCACTTGTCCCCTTTGATCAGTTTGCGCCATGGAATCAAGCACCGTCGTTTGCAGATCCAATGATGTTCCCTTATAATTAG

>AT5G27050.1 | Symbols: AGL101 | AGAMOUS-like 101 | chr5:9520276-9520638 FORWARD LENGTH=363

ATGTTCAAGAAAGCCTTGGAGCTTTCAACTCTGTGCAACATCGAAGTCTGTGTCATATATTATGGTCGTGACGGAGAACTCTTCAAGACATGGCCGGAGGATGAATCCAAGGTTCGAGACATGGCAGAGAGGTTTACCAAACTAAACGAGAGAGAGCGACGGAAGAAAAGAACCAATCTTTCTCTGTTTCTACGTAAGAAGATCCTCGACGACAATAAGCTCTCAGGGAAAGTCTTAGAGATGAAGGATTCGTTAGAAAGGGGTCTACGGGTGCTACAAGATAAGCTTCTGTTACTACAACCCGAGAATCAGACCAAGTCCCTGACTCGTTCTGTTTCTAGTTTAGATTATGTGTTCGTTTAA

>AT5G27070.1 | Symbols: AGL53 | AGAMOUS-like 53 | chr5:9527741-9528604 FORWARD LENGTH=864

ATGGATTCTTCAATGTCGACGAAGAAGAAAACCAAATTGTCTGTGAGAAACCAAACTTGTTTCAAGAAATCGTCCCTGTCTTCTTCTTCTACTGCAAAAAAGACCACGAATTTGTCTATGAGAGAACAGACCATGTTCAAGAAAGCCTTGGAGCTTTCAACTCTGTGCAACATCGATGTCTGTGTCATATACTATGGTCGTGACGGAAAACTCATCAAGACATGGCCGGAGGATCAATCAAAAGTTCGAGACATGGCTGAGAGGTTTAGCAGATTACACGAGAGAGAGCGATGCAAGAAAAGAACCAACCTTTCTCTGTTTCTACGTAAGAAGATCCTCGACGACACTAAATTGTCGGAGAAAGTCTTAGAGATGGAGGATTCGTTAGAAAGTGGTCTACGAGTACTACAAGATAAGCTTCTGTTACTCCAACCCGAGAAAAACCAGACCGAGTTTGGTCAGACCCGTGCGGTTTCTTCCACAACGAATCCATTGTCTCCTCCTCCTTCGCTAATTGAGGATCATCGTCATCAACAATGGACAGAACCATTGATGAGTGGTGTGTCAAACACAGAGCAAGACCTATCGACGTCATCATTGAGTCAAAATCAGAGCAGAATTTCAGTCTTTCTCTATAACCATGACAACCGTAGCTTCTATCAAGTCCCTGACTCAGTTTCTAGCTTTGACCAATCGGCTTTACTTGGGGAACAAGGATCTGGTCTAGGAAGTAACTTTGATCTTCCTCCCATGGTTTTTCCTCCTCAGATGCAGACACAAACCCCACTTGTCCCCTTTGATCAGTTTGCGGCATGGAATCAAGCACCGTCGTTTGCAGATCCAATGATGTTCCCTTATAATTAG

>AT5G27090.1 | Symbols: AGL54 | AGAMOUS-like 54 | chr5:9531845-9532408 FORWARD LENGTH=564

ATGGATTCTTCAACGTCTACGAAGAAGAATACCAAATTGTTTGTAAGAAACCAAACTTGTTTCAAGAAATCGTCTCTGTCTTCTTCCAATGCAAAAAAGACCACGAATTTGTCGATGAGAGAACAGACCATGTTTAAGAAAGCCTTGGAGCTTTCGACTCTGTGCGACATCGAAGTCTGTGTCATATACTATGGTCGTGACGGAAAACTCATCAAGACATGGCCGGAAGATCAATCTAAGGTTCGAGACATGGCAGAGAGGTTTAGCAGATTACACGAGAGAGAACGATGCAAAAAAAGAACCAACCTTTCTCTGTTTCTACGTAAGCAGATCCTCCACGACAAGAAATTGTCGGAGAAAGTCTTAGAGATGGAGGATTCGTTAGAAAGTGGTCTACGGGTACTACAAGATAAGCTTCTGTTACTCCAACCCGAGAAAAACCAGACCGAGTTAGGTCAGAGCTGTGCGGTTTATTCCACAACGTATCCATTGTCTTCTCCTTCGCTGATTGAGGATCATCAACATCAACAACAATGGACAGAACCATTGTCAAACACAGAGTAA

>AT5G27130.1 | Symbols: AGL39 | AGAMOUS-like 39 | chr5:9546633-9547553 FORWARD LENGTH=921

ATGCCTTCATCTGATTCCACGATGATGAAGAAAGGAACAAAGCGCAAAATCGAAATAAAGAAACGAGAAACCAAAGAGCAACGAGCCGTGACTTGCTCTAAACGCCGTCAAACTGTTTTTTCCAAAGCCGCCGATCTCTGTCTTATCTCCGGTGCTAACATCGCCGTCTTCGTAACCTCTCCCTCCGATAGTTCCGATGTTGTCTATTCCTTCTCCGGTTACTCCTCTGCCTACGAAATCGCTGATTGTTACCTAAATCGCAAGCCTCCACCCAAGATTGTTAACCCGGCCGGATCCAAACTAGGATTTTGGTGGGAAGACCCTGATCTCTACCATTCGTGTGATGATCTCTCTGAGTTAAGCATTATCGAGGATCGTTTACAGAGAATGAAGAAGCATGTGATGGCTTGCCTTGAGAAGGAAGAAAAATCTCAACTTGTTTCTAGTTTCGACCAAAACCCTAATAGTACTTGTTCTCTCGACGTCGAGGATTGTGATGGATCTTCTTATTCGCAAATTGCTTCTACTTTTACTCCAAACTCTGTCAACGAGTATTGCAGCGATCAAACCTTTTCTTCTTTCCATGGCGATCAAAACCCTAACCTCTCGTCTCCGTCTTTTGATCAGGACTGTTACTCCTCTCTCTATCAGATCTGTGGAGAATCATCTTCACAAGTTGCTTCTTTTGATCAAAACCCTAGCTCCGAGATCCAAGGATTTGAAACAGAGGAAGAGATTAACCAAATCAATCTACTACTACAAGAAACACAAACAGAGGCCAATGTCAATTTGGATGATGAGATCTGCTTTTGGAATGATCTATCCAACGATGATGTGTTTGGTCTAAACAGCTACTTCGGTCTCGATAACACCAATGCAATGATCAACTTCGGAGATTCCGACTTTAGACGTCATGTCTGA

>AT5G27580.1 | Symbols: AGL89 | AGAMOUS-like 89 | chr5:9736651-9737322 FORWARD LENGTH=672

ATGGATTCTTCAATGTCGACGAAGAAGAAAACCAAATTGTCTGTGAGAAACCAAACTTGTTTCAAGAAATCGTCTCTTTCTTCTTCTTCTACTGCAAAAAAGACCACGAATTTGTCTATGAGAGAAGAGACCATGTTTAAGAAAGCCTTGGAGCTTTCAACTCTGTGCGACATCGAAGTCTGTGTCATATATTATGGTCGTGACGGAGAACTCATCAAGACATGGCCGGAAGATCAATCCAAGGTTCGAGACATGGCTGAGAGGTTTAGCAAACTACACGAGAGAGAGCGACGCAAGAAAAGAACAAACCTTTCTCTGTTTCTACGTAAAAAGATCCTCGACGACAATAAATTGTCGGAGAAAGTCTTAGAGATGAAGGATTCGCTAGAAAGTGGTCTACGGGTATTACAAGATAAGCTTCTGTTACTCCAACCCGAGAACCAGACCGAGCTAGGTCAGAGCCGTGCGGTTTCTTCCACAACGAATCCATTGTCTTCTCCTGAGGATCATCATCATCAACAATGGACAGAACCATTGGTGACTGGTGTGTCAAACACAGAGCAAGACCTATCGACGTCACCATTGAGTAACCATCAGAGCAAATATTCAGTCTTTGTCTATAACCATGACAGCGGAAGCTTCTATCAAGTCCCTGACTCGATCTGTTTCTAG

>AT5G27810.1 | Symbols: no symbol available | no full name available | chr5:9855827-9856186 FORWARD LENGTH=360

ATGAAGAAAGTACATGAACTCTCAACTCTATGTGGCATCACATCGTGTGCGATCATCTACAGTCCGTATGATACCAGTCATGAAGTGTGGCCATCAAATTCTGGTGTGCAAAGAGTGGTTTCCGAATTTAGGACACTCCCGGAAATGGATCAACACAAAAAGATGGTAGATCAAGAAGGTTTTCTCAAACAAAGGATCGCGAAACCGACAGAGAATTTGAGGAGACAAAGAAAGGACAATAAGGAGCTCGAGATGACTGAAGTCATGTTTCGATGCTTAATTGGAAACATGGAGATGCTTAAATCAGAATCACAATCAGAATCAACAACAATGGTTTATGAAAATGATGAACCATCTTGA

>AT5G27944.1 | Symbols: no symbol available | no full name available | chr5:9975918-9976592 REVERSE LENGTH=675

ATGTATTCTTCAATGTCGACGAAGAAGAAAACCAAATTGTCTGTGAGAAACCAAACTTGCTTCAAGAAATCGTCTCTGTCTTCTTCTTCCACTGCAAAAAAGACCACGAATTTGTCTATGAGAGAACAGACCATGTTTAAGAAAGCCTTGGAGCTTTCGACTCTCTGCGACATCGAAGTCTGTGTCATACTTTATGGTCGTGACGGAGAACTCATCAAGACATGGCCGGAAGATCAATCAAAAGTTCGAGACATGGCTGAGAGGTTTAGCAGATTACACGAGAGAGAGCGATGCAAGAAAAGGACAAACCTTTCTCTGTTTCTACGTAAGAAGATCCTCGACGACAATAAATTGTCTGAGAAAGTCTTAGAGATGGAGGATTCGTTAGAAAGTGGTCTACGGGTACTGCAAAATAAGCTTCTGTTACTCCAACCCGAGAAAAACCAGACCAAGTTAGGTCAGAGCCGTGCGGTTTCTTCCACAACGAATCTATTGTCTTCTCCTGAGAATCACCATAATCAACAATGGACAGAACCATTGGAGAATGGTGTGTCAAACACAGAGCAAGAGCTATCGACTTCATCATTGAGTCAACATCAGAGCAAATATTCTGTCTTTCTTTATAACCATGACAACGGAAGCTTCTATCAAGTCCCTGACTCGATCTGTTTCTAG

>AT5G27960.1 | Symbols: AGL90 | AGAMOUS-like 90 | chr5:9991685-9992770 REVERSE LENGTH=963

ATGAAGAAGGTAAAGCTATCTTTGATAGCTAATGAAAGATCAAGGAAAACATCCTTCATGAAGAGGAAAAACGGGATATTCAAGAAACTCCACGAGTTGTCAACTCTATGTGGTGTCCAAGCTTGTGCTCTCATCTATAGTCCATTCATACCGGTTCCAGAGTCATGGCCGTCAAGGGAAGGTGCTAAAAAGGTAGCTTCAAAGTTTCTGGAGATGCCGCGGACAGCCCGAACCAGGAAGATGATGGATCAAGAAACCCATCTTATGGAGAGGATTACCAAAGCAAAAGAGCAACTAAAGAATTTGGCTGCTGAGAACCGAGAATTACAGGTTAGACGATTTATGTTTGATTGTGTTGAAGGCAAAATGTCCCAGTATCGTTATGATGCAAAAGACCTTCAAGATTTGCTATCTTGTATGAATCTATATCTCGATCAGCTTAACGGAAGGATCGAGTCCATTAAAGAAAACGGTGAGTCGTTGTTGTCTTCCGTCTCTCCTTTTCCTACTAGAATTGGTGTTGACGAAATTGGTGATGAGTCGTTTTCCGACTCTCCTATTCATTCTACAACTAGGGTTGTAGATACTCCTAATGCTACCAATCCTCATGTTCTTGCGGGCGATATGACTCCTTTTCTTGATGCGGACGCAAATGCGAATATGAATCAGGTTCAATACCAGGCTCCTAATAATCTGTTTAATCAGATTCAACGAGAATTCTACAACATAAATTTGAATCTGAATTTGAATCTGAATTCAAATCAGTATCTGAATCAACAACAATCATTCATGAATCCGATGGTGGAACAACATATGAATCATGTTGGAGGGCGTGAAAGCATTCCTTTCGTGGACAGAAACTACTACAACTACAATCAACTACCAGCCGTTGATCTTGCTTCCACCAGTTACATGCCTTCAACCACCGATGTTTATGATCCTTACATCAACAACAATCTCTAA

>AT5G37415.1 | Symbols: AGL105 | AGAMOUS-like 105 | chr5:14839047-14840301 REVERSE LENGTH=951

ATGAGAGACCCAGATATTGTACAAAATACGCGATCTTTGATATTGTTGAGAGTCTTGACGCATGAAAAGTTGATTCAAAGTCATCGCTATTTCTTCGTTGGTTGGATTCACAAGAATCTCTCTCGCTTTCGACGGAAGATTCGAAGAAGATTCCTCCATTGCGATTTCTCAGGATTCGGATTTTTTCCGAGAAGATTTCTTTTCGGTTTTGTTCTGGAGAAGATGGACCATCTTCAAGAAAGCTTCAGAGGCTCAGAGCTTTGCATACTCTGTGATATCGAAGCCTGCGTCATCTATTACGGACCGGACGGAGAACTCAAGACATGGCCTAAGGAGCGAGAGAAAGTGGAAGACATTGCTCTCAGGTATAGTCAATTAAACGAAGCCTTGAGAAGGAAGAAAAGTGTTACTCTTTATGACTTCCTGAACAAGAAGAAGGACAAGACGAATTTGGAGAAGAAAGCAATGATAACGGACAATGATGACTTGAAGACTTGTTTGAAGAACGTGAATGTTTTAAAGTCTCCAATCGCTGATCATTACTTTAATGACCAAATTTCTCAACTGATTCAGTCCTTAGAACCTCATGTCTCTAAAGTCCAAGAAAGGATTCGTTTTGTTGAGTCGCAGAAACATAAGGAGACAAAACTGGATCATCAGAGTTTAGCATCAATATATTCTCTGAACCAATCTTTGAACCCTAGCCAGTTCACGCTGTTTTTGTACAACCATGGAGACAATACTATGTCTCAGATCCCAAACATGTTTATGAACAACAACAACTTCCAACATTCTTTTGTCTCAAACACACAAGATTACTCTGCTCTCCAAGAATCGGTGAATAACAACTATGGGTTGATGCCGAATGTTCTTTGTGGTTATGATCAGAACCTGTTCACGAGTGATATTACCAACAACAACCTTCTTATCGACAACTCGATGTATCTCTGA

>AT5G38620.1 | Symbols: AGL73 | agamous-like 73 | chr5:15463858-15464907 REVERSE LENGTH=1050

ATGGTGAAAGGCACGAAGAGAAAGATTGCGATTGAGACGATACAGAAGAGAGATTCCCTTAGGGTTACCTGCACCAAACGCCGTAAAGGTCTCTATAGCAAAGCCTCTCAGCTTTGTCTTCTCTCCGACGCACAAATCGCTATCTTAGCGACTCCTCCTTCTTCTGAATCCGACGTCTCTTTCTACTCTTTCGGTCACTCCTCTGTTGATGCCGTTGTCTCTGCTTTTCTCTCCGGAAAACGTCCTGTTTCGGCTCCGAAGGATAACAAAGAGACGAGGGAGGACGTTGGTATCTGTTTGACTCGCAAGAATCTAGGGTTAGGGTTTTGGTGGAATGACGAGAGCCTTGTGAGATCGGAGAATCCTCAAGAGATTAGTGAAGCGATCGGTTCCATGTGGACTCTGTTGAGTAATCTCAAGGAGTTGCGTGCGGATGAAGCTTGCGTTAACGATCACAAGGACTTGAAGAAGAATGAGAAGAGCGATGTTCACGGAACTCAAGATCAAACCCTAATTTTTCAGTCCGCTTCTGCAGTTTGTTGCATCCCTGAAAATCTCAACGATATTACTCAAGAGCCAAATCAAACCCTAGATATTCAGTCCAGTAGTTCTGCAATCTGTTGCGTCCCTGATAAATCACCTGAAATTTTCAATGAAATCACTGAAGAACAAGATCAGATTCTCTCGATTTGTGAAACTTTTTGTGTTACGGATAATAACAACAACAACAACAACAACGCTGCTTTACCTGAAGTTAACCTATATTACAATCAAGACATGGCTATTGATCAACTCATTGACTTCAACACCCCTTTTGAAAGCTCGATAGATGATTGGTTTTCGGACAACACTACTCATCAGGAGACGACTAGTGCAAGTATTTTGAACGATGTTGGTGTTGATGATCAAGTCTCGGTGGATACAAATCCGTTTTCTTATTTTCAAAGCCTTGAAGATGCAGATTTGGTGTTTCAGAGATGTTTGGATGGAGATAATCTACGATTCTCTGATTGTTTCAATGACTTTGCAAACACTATTGCAGCAGTTTGA

>AT5G38740.1 | Symbols: AGL77 | AGAMOUS-like 77 | chr5:15513025-15514305 REVERSE LENGTH=1281

ATGACGACCATTCGATCTTCACCTTCTTCCTCTCGTTGTTCCAATTCGTCTTCCTCTTCTTCTTATTCACTCGCTTCCACAAGTTTGTCTAACAGACTTGAGACTATATTCAAGAAAGCTTCAGAGCTTTGCACTCTCTGTGATATCGAAGCCTGCGTCATCTATTACGGACCAGACGGAGAACTTAAAACATGGCCTAAGGAGCGAGAGAAAGTGCGAGACATTGCTCTGAGGTTTAATCAATTAAACGAAGCCTTGAGACACAAGAAAAGCGTTAATCTTCATGGGTTCCTGAACAAGAAGAAGAAGAACAAGGGTTTGAAGAATCCGAACAAGAAGAAGAAGACGAGTCTTAAGAACGTGAATGTTTTAAAGTATCCACTCGCTGATCATTACTCTCCCGACCAAGTTTCTCAATTGACTCAGTCCTTAGAACTCAATGTCTCTAAATTCCAAGAAAGGCTTCGATTTCTTGAGTCGCAGAAACAGAATGAGACAAAACCGGATCATCAGAGTTTAACATCAATATCTTCTCTGAACCAATCTTTGAACCCTAGCCAGTTCTCGCTGTTTATGTATAACCATGGATATAATACTCTGTCTCAGATCCCAGTCTCTGCATCAAATTTCAATCAGGATTATATCTCAGCGTTACTTGAACAATCTGAGTTGAAGAGTCAGATAATGAAGCAGGAGGTTTGTGGTTATGAGCAGAACATGTGCATGAGTAACCATGGAGATGCTACGCTCTCTCAAATCCCATTCTCTGCATCAAATTTCAATCAAGATTTTTCAGCCAACAACAACTTCCAACATTCTTTTGTCTCAAACACACAAGATTACTACTCTGTTCAAAAATCTGTGAATAACAACTATGGGTTGAAGAATCAGTTAATGAAGCACGATCTTTGTGGTTATGAGCACAACATGTGCATGAGTAACCATGGAGATGCTACCTTCTCTCAAATCCCACTCTCTGCATCAAATTTCAATCAAGATTTTTCAGTCTCAATACAAGAAGAATCTGGCTTGATGCAGCAGGAGCTTTGTGGTTATGATCAGAATCAGAACATGAGCATGGGTGACATCACAAACAACAATTTTCAAGTTACTTGCGCCTCAGTACTAGAATCTGTGAACAACTTTGGGTTGAATCAGTTGATGCACAAGGAGTTTTATGGTTGTCATCAAAACATGTCTATGGGTAACATCAATAACAACAGCTTTCAACATCCTTGGGTCTCAAACGCAGACCATACTCGGCGGTACAAGAATCTGTGA

>AT5G39750.1 | Symbols: EMB3008, AGL81 | EMBRYO DEFECTIVE 3008, AGAMOUS-like 81 | chr5:15906875-15907942 FORWARD LENGTH=1068

ATGGCCATTCGATCTTTACCTTCTTCCTCTCGCTGTTCCTCTTCTTCTTCTTCTTCTTCTTATTCACTCGCTTCAACGAGTCTAAGCAATAGACTTGAGACCATCTTCAAGAAAGCTTCAGAGCTTTGCACTCTCTGTGATATCGAAGCATGCGTCATCTATTACGGACCAGACGGAGAACTCAAGACATGGCCTCCGGAGCGAGAGAAAGTGGAAGACATCGCTCTCAGGTATAGTCAATTAAACGAAGCCTTGAGAAGGAAGAAAAGTGTTACTCTTTATGACTTCCTGAACAAGAAGAAGGACAAGACGAACTTGGAGAAGAAAGCAAAGATAACGGACAATGATGACTTGAAGACTTGTCTGAAGAACGTGAATATTTTAAAGTATCCACTCGCTGATCATTACTCTCCCGACCAAGTTTCTCAACTGATTCAGTCCTTAGAACCTCATGTCTCTAAAGTCCGAGAAAGGATTCGTTTTGTTGAGTCGCAGAAACACAAGGAGACAAAACCGGATCATCAGAGTTTAGCATCATCCTCTCTGAATCATCAGACCCAATCTTTGAACCCTAGCCAGTTTTCGCTGTTTATGTATAACCATGGAGACAATACTCTGTCTCAGATCCCAGTCTCTGCATCAAATTTCAATCAGGATTATTTCTCAGCGTTACTTGAACAATCTGAGTTGAAGAGTCAGATAATGAAGCAGGATCTTTGTGGTTATGAGCAGAACATGTGCATGAGTAACCATGGAGATGCTACGCTCTCTCAAATCCCACTCTCTGCATCAAATCTCAATCAAGATTTCTCAGCCTTACTACAAGACGAATCTGGCTTGATGCAACAGGAGCTTTGTGGTTATGATCAGAACATGTTTATGAACAACAACAACTTTCAACATTCTTTTGTCTCAAACACACAAGATCACTCTGCTCCTGTGGTACAAGAATCTGTGAACAACAACTATGGGTTGATGCCGCATGTTCCTTGTGGTTATGATCAGAACCTGTTCACGAGTGATATTACCAACAACAACCTTCTTATCAACAACTCGATGTTTCTCTGA

>AT5G39810.1 | Symbols: AGL98 | AGAMOUS-like 98 | chr5:15937278-15938344 REVERSE LENGTH=990

ATGGCCATTCGATCTTTACCTTCTTCCTCAGGCTGTTCCAATTCCTCTTCGTCTTCTTCTTATTCACTCGCTTCAACAAGTTTGAGCAATAGACTTGAGACCATCTTCAGGAAAGCTTCAGAGCTTTGCACTCTCTGTGATATCGAAGCATGCGTCATCTATTACGGACCAGACGGAGAACTCAAGACATGGCCTCCGGAGCGAGAGAAAGTGGAAGACATTGCTCTCAGGTATAGTCAACTAAACGAAGCCTTGAGACGCAAGAAGAGCGTTACTCTTTATGACTTCCTGAACAAGAAGAAGAACAAGACGAACTTGGAGAAGAAAGCAAAGATAAAGGACAACGACTTGAAAAGACTTTCCTTAGAACCTCATGTCTCTAAAGTCCGAGAAAGGATTCGTTTTGTTGAGTCGCAGAAACATAAGGAGACAAAACCGGATCATCAGAGTTTAGCATCATCCTCTCTGAATAATCAGACCCAATCTTTGAACCCTAGCCAGTTTTCGCTGTTTATGTATAACCATGGAGATAATATTCTGTCTCAGATCCCAGTCTCTGCATCAAATTTCAATCAGGATTATTTCTCAGCGTTACTTGAACAATCTGAGTTGAAGAGTCAGATAATGAAGCAGGAGGTTTGTGGTTATGAGCAGAACATGTGCATGAGTAACAATGGAGATGCTACGCTCTCTCAAATCCCACTCTCTGCATCAAATTTCAATCAAGAGTTCTCAGCCTTACTACAAGAAGAATCTGGCTTGATGCAACAGGAGCTTTGTAATTATGATCAGAACATGTTTATGAACAACAACAACTTCCAACATTCTTTTGTCTCAAACACACAAGATCACTCTGCTCCTGCGGTACAAGAATCTGTGAACAACAACTATGGGTTGATGCCGCATGTTCCTTGTGGTTATGATCAGAACCTGTTTACGAGTGATATTACCAACAACAACCTTCTTATCGACAACTCGATGTTTCTCTGA

>AT5G40120.1 | Symbols: AGL76 | AGAMOUS-like 76 | chr5:16051879-16053036 FORWARD LENGTH=1158

ATGACTATGCGATCATTACCTTTTTCCTCTTCTTCTTATTCACTCGCTTCCACAAGTTTGAGTAACAGACTTGAGACTATATTCAAGAAAGCTTCAGAGCTTTGCACTCTCTGTGATATCGAAGCGTGCGTCATCTATTACGGACCAGACGGAGAACTTAAGACATGGCCTAAGGAGAAAGAGAAAGTGAGAGACATTGCTCTGAGGTATAGTCAATTAAACGAAGCCTTGAGAAGCAAGAAAAGCGTTAATCTTCATGGGTTCCTGAACAAGAAGAAGAAGAAGAAGAAGAAGGGTTTGAAGAATCCGAACAATAAGAGGAAGACTTGTCTTAAGAAGAACGTGAATGTTTTAAAGTATCCACTCGCTGATCATTACCCTCCCGACCAAGTTTCTCAACTGACTCAGTCCTTAAAACTCCATGTCTCTAAATTCCAAGAAAGGCTTCGATTTCTTGAGTCGCAGAAACAGACAAAACCGGATCATCAGAGTTTAACACCATCCTCTTTGAATCATCAGACCCAATCTTTGAACCCTAGACAGTTTTCGCTGTTTATGTATAACCATGGAGACAATACTCTGTCTCAGATCTCAGTCTCTGCATCAAATTTCAATCAGAATTATTTCTCAGCGTTACTTGAACAATCTGAGTTGAAGAATCAGTTAATGAAGCAGGATGGTTATGATCAGAATCAGAACATGAGGATGGGTGACATCACCAACAACAATTTTCAACTTCCTTACTTCTCAAAGAAAGAAGCGGTACAAGAATCTGTGAACTACTTTGGGATGAATCAGTTGATGCTGAAGGAGTTATATGGTTGTGATCAAAACATGTGTATGGGTAACATCAATAGCAACAGCTTTCAACATCCTTGCGTCTCAAAGGCACAACATTACTCGGCGGTAGAAGGATCTGTCAATAACCAACGACAATCTGAGTTAATGCAGCAGGAGCTTTGTGGTTATGAGCAGAACATGTGCTTTACCAACAACAATTTTCAAGTCTCAAACAAAGAAGCGGTTCAAGAATCTGTGACTAATTTTGGGTTGATGCAGCATGAACTTTATGGGTGTGATCAAAACATGTCTATGGGCAACATCATTAACAACAGCTTTCAACAGCGTCTCAAACACAGAACAAGAATCTGTGAATAA

>AT5G40220.1 | Symbols: AGL43 | AGAMOUS-like 43 | chr5:16078390-16079364 REVERSE LENGTH=975

ATGACTATGCGATCATCTTTACCTTCTTCCTCTTCCGCTTATTCACTCGCTTCAACAAGTTTGAGCAACAGACTTGAGACCATCTTCAAGAAAGCTTCAGAGCTTTGCACTCTCTGTGATATTGAAGCCTGCGTCATCTATTACGGACCAGACGGAGAACTCAAGACATGGCCTCCGGAGCGAGAGAAAGTGAGAGACATTGCTCTTAGGTATAGTCAATTAAACGAAGCATTGAGACGCAAGAAAAGCGTTAATCTTCATGGGTTCCTGAACAAGAAGAAGAAGAACAAGGGTTTGAAGAATACGGACAAGAAGAGGAAGACGAGTCTTAAGAAGGTGAATGTTTTAAAGTATCCACTCGCTGATCATTACCCTCCCGACCAAGTTTCTCCACTGATTCAGTCCTTGGAACTCCATGTCTCTAAATTCCACGAAAGGCTTGAATTTCTTGAGTCGCGGAAACAGAATGAGACACAACCGGATCACCACAGTTTAGCATCATCCTCTCTGAATCATCAGACCCAATCTTTGAACCCTAGCCAGTTCTCGCTGTTTATGTATAATCATGGAGACAATACTCTGTCTCAGATCCCAGTCTCTGCATCAAATTTCAATCAGGATTATTTCTCAGCGTTACTTGAACAATCTGAGTTGAAGAATCAGTTAATGAAGCAGGAGATTTGTGGTAATGATCAGAATCAGAACATGTGGATGGGTAACATCACCAACAACAATTTTCAACTTCCTTGCGTCTCAGTACAAGAATCTGTGAACAACTTTGGGTTGATGCACAAGGAGTTTTATGGTTGTGATCATAACATGTCTGTGGGTAACATCAATAGCAACAGCTGTGAACATCCTTGCGTCTCAAGCACACAACATTACTCGGCGGTAGAAGAATCTGTGAATAACCCCTGGTTGAATCAGTTGATGCAGAATGAACTTTATGGTTACGGTTATGCAGACTTTTGTTAA

>AT5G41200.1 | Symbols: AGL75 | AGAMOUS-like 75 | chr5:16490544-16491536 FORWARD LENGTH=993

ATGACTATGCGATCATCTTCACCTTCGTCCTCTTCTTCTTATTCACTCGCTTTCACAAGTTTGAGCAATAGGCTTGAGACCATCTTCAAGAAAGCTTCAGAGCTTTGCACTCTTTGTGATATCGAAGCCTGCGTTATCTATTACGGACCAGACGGAGAACTTAAGACATGGCCTAAGGAGAAAGAGAAAGTGAGAGACATTGCTCTTAGGTATAGTCTATTGAACGAAGCATTGAGACGCAAGAAAAGCGTTAATCTTCATGGGTTCCTGAACAAGAAGAAGAACAAGGGTTTGAAGAATCCGAACAAGAAGATGAAGACGAGTCTTAAGAACGTGAATATTTTAAAGTATCCACTCGCTGATCATTACCCTCCCGACCAAGTTTCTCCACTGATTCAGTCCTTGGAACTCCATGTCTCTAAATTCCAAGAAAGGCTTCGATTTCTTGAGTCGCAGAAACAGAATCAGACAAAACCGGATCATCAGAGTTTAACACCATCCTCTCTGAATCATTACACCCAATCTTTGAACCCTAGCCAGTTCTCGCTGTTTATGTATAACCATGGAGACAATACTCTGTCTCAGATCCCAGTCTCTGCATCAAATTTCAATCAGGATTATTTCTCAGCGTTACTTGAAGAATCTGAGTTGAAGAATCAGTTAATGAAGCCGGAGATTTGTGGTTATGATCAGAATCAGAACATGAGCATGGGTGACATCACAAACAACAAATTTCAAGATCCTTGCGTCTCAAACAAAGAAGCGGTACAAGAATCTGTCAACAACTTTGGGTTGAATCAATTGATGTACAAGGAGTTTTATGGTTGTGATCAAAACATGTCTATGGGTAACATCAATAGCAACAGCTTTCAAAATCCTTGCGTCTCAAACACACAACATTACTCGGCGGTAGAAGAATCTGTGAAGAACCCCTGGTTGAATCAGTTAATGCAGAATGAACTTTACGGTTACGGTTATGCAGGCTTTTGTTAA

>AT5G48670.1 | Symbols: FEM111, AGL80 | AGAMOUS-like 80 | chr5:19738825-19739790 REVERSE LENGTH=966

ATGACAAGAAAGAAAGTGAAACTTGCTTACATTTCCAACGATTCTTCAAGAAAAGCGACTTTCAAAAAAAGAAAGAAGGGTTTGATGAAGAAGGTACACGAACTCTCGACTCTTTGTGGCATCACTGCATGTGCGATCATCTACAGCCCGTATGATACCAACCCTGAAGTGTGGCCATCAAATTCCGGTGTGCAAAGGGTGGTTTCAGAATTTAGGACACTCCCAGAGATGGATCAACACAAAAAGATGGTAGATCAAGAGGGTTTTCTCAAACAAAGAATCGCGAAAGCGACAGAAACTTTGAGGAGACAAAGAAAGGATAGTAGGGAGCTAGAGATGACTGAAGTCATGTTCCAATGTTTGATTGGAAACATGGAGATGTTTCATTTGAATATTGTGGATCTTAATGATTTGGGTTATATGATTGAGCAATATCTTAAAGATGTTAATCGCAGGATTGAGATTTTACGAAATTCTGGTACGGAGATTGGTGAATCTTCTTCTGTTGCGGTAGCTGCTTCTGAAGGGAATATACCAATGCCGAATTTGGTCGCTACAACAGCTCCCACCACTACAATTTATGAGGTGGGTTCTTCTTCCTCATTTGCGGCTGTCGCGAATTTTGTTAATCCTATTGATCTTCAACAATTTCGTCATCCGGCGGCTCAACATGTTGGACTTAATGAGCAACCTCAAAATCTAAATCTGAATTTAAATCAGAACTATAATCAAAATCAAGAATGGTTTATGGAAATGATGAACCATCCTGAGCAAATGAGGTATCAGACAGAGCAAATGGGATATCAATTCATGGATGATAACCACCACAACCACATCCATCACCAGCCACAGGAGCACCAACATCAGATCCATGACGAATCTTCCAACGCTCTTGATGCCGCCAACTCAAGCAGCATCATCCCTGTTACTAGTTCAAGTATTACCAATAAAACATGGTTCCATTAG

>AT5G49420.1 | Symbols: AGL84 | agamous-like 84 | chr5:20035166-20036170 REVERSE LENGTH=1005

ATGGTGAAAAAAGGTGGCACGAAGAGGAAGATTGCGATTGAGACGATACAGAAGAGAGATTCCCTTAGGGTTACCTGCACCAAACGCCGTGAAGGTCTCTATAGCAAAGCCTCTCAGCTTTGTCTTCTCTCAGACGCACAGATCGCTATCTTAGCGACTCCTCCTTCTTCTGAATCCAACGTATCCTTCTACTCTTTCGGTCACTCCTCTGTTGATGCCGTTGTCTCTGCTTTTCTCTCCGGACAACGTCCTGTTCCGAAGGATAACAAAGAGACGAGGGAGGACGTTGGTATCTGTTTGACTCGCAATAATCTAGGGTTAGGTTTTTGGTGGAACGACGAGAGCCTTGCCAGATCGGAGAATCCTCAAGAGATTAGTGAAGCGATCGACTCTATGCGGACGCTGTTGAGGAATCTCAAGGAATTGCGTGCGGATGAAGCTTTAGCCTGTAATCAAGCTTTCGTTAATGATCGCGAGGACCTGAAGAACAACGACAAGTGCGATTTTGTTTCAGACCACGAAACTCATGATCAAACCCTAATTCTTCAGTCCGCTTCTCCAATTTGTTGCATCCCAGAAAATCTCAACGAGATTACTCAAGAGCCAAATCAAACCCTAAATATTCAGTCGAGTACTTCTGCAATTTGTTGCGTCCCTGATAATTCACCTGAAAATTTCAATGAGATCACTGAAGAACAAGATCAGATTCGCTCGATTTGTGAAACTTTTTGTGTTATGGATAACAACGCTGCTTTACCTGAAATGAACCTGGATTACGATCAAGACATTGGCTTCGACACGCCTTTTGAGAGCGCGCTAAATGATTGGTTTTCGGACAACACTACTCATCAGGAGATTAGTGCAAGTATTTTGAACGCTGTTGTTGATGATCAAGTCTCGGTGGATCTAACTCCGTTTTCTTATTTTCAGAGATGTTTGGATGGAGATAATCTACGATTCTCTGATTGTTTCAAAGACTTTGCAAACACTATTTCAGCACTTTGA

>AT5G49490.1 | Symbols: AGL83 | AGAMOUS-like 83 | chr5:20075328-20076185 FORWARD LENGTH=858

ATGAGATTCGTTCCTTATTTATACGAGATTGAGAGATTATGGCTTTCACTTGTCAATTATCTTTCTCCACGCAAAAACAAAAACCGTCGCTGTGGTGAAATAGATAAAATCAGAATGGTGAAAAAAGGAGGTACGAAGAGGAAGATTGCGATTGAGACGATACAGAAGAGCGATTACCTTAGGGTTACTTGCACCAAACGTCGTGAAGGTCTCTTTAGCAAAGCTTCTCAGCTTTGTCTTCTCTCCGATGCACAGATCGCTATCTTAGCGACTCCTCCTACTTCTGAATCCAACATCTCCTTCTACTCTTTCGGTCACTCCTCCGTTGATGCCGTTGTCTCTTCTTTCCTCTCCGGGCAACGTTGTGTTCCTCTTCAGGAGGATACCAAAGAGATGAGAGAGGACGTTGCTATTTGCTTGTCTCGTACAAATCTAGGGTTAGGGTTTTGGTGGAACAACGAGAGCCTCAACAAATCGGAGAATCCTCAAGAGATTAGTGACGCTATCAACTCTATGTTAACGCTCTTGAGTAATCTCAAGGAATTGAGTGGGGAGGAAGCTTTAGTTAACGATCACAAGGACTTGAAGAAGAATGAGAGGAGCGATGTTGTTTTACAACACGGAACTCAATATGAAACCCTAAATCCTAACTCCAACACTACTACAATTTGTTGCGTCCCTGATGAATTACCTGCCAATTCCAACGAGATTGTTGGAATCTCACCAAATCCTTTGATTATGCTAGAAAAGAAGAAATCACAAATTGAAGAAAAGTTTGAGAAGGAATGGCAAGTTTCAGTTACTCGAATTGAAAATGAAGCCACGAGTTCTTATGCAAAACGAAGAAGAAGTATATGA

>AT5G51860.1 | Symbols: AGL72 | AGAMOUS-like 72 | chr5:21081844-21084126 REVERSE LENGTH=636

ATGGTGAGAGGAAAGATCGAAATCAAGAAGATTGAGAACGTGACAAGTAGACAAGTCACGTTTTCGAAGCGAAGGAGTGGTCTCTTTAAGAAGGCTCATGAGCTTTCTGTTCTATGTGATGCTCAAGTGGCAGCTATGATCTTCTCTCAGAAAGGAAGATTATATGAATTCGCTAGCTCCGATATCAGGAACACGATAAAGCGATACGCTGAGTACAAGAGAGAGTATTTTGTTGCAGAAACTCATCCTATAGAGCAATACGTGCAGGGGCTAAAGAAGGAAATGGTGACAATGGTGAAAAAGATTGAAGTGCTTGAAGTCCATAACCGGAAGATGATGGGACAAAGTTTGGATTCTTGTTCGGTAAAAGAACTTTCAGAGATAGCCACACAGATAGAGAAAAGCCTTCATATGGTTAGATTAAGAAAGGCTAAGTTATATGAAGATGAGCTACAGAAACTAAAAGCCAAGGAGAGGGAACTCAAGGACGAGAGAGTCAGGCTTTCTCTAAAGAAAACAATTTATACTCACTTATGCCAGGTTGGAGAAAGACCAATGGGGATGCCGTCGGGAAGCAAAGAGAAAGAGGATGTTGAAACTGATCTATTTATTGGATTTCTGAAGAACCGACCATAA

>AT5G51870.1 | Symbols: AGL71 | AGAMOUS-like 71 | chr5:21085635-21087923 REVERSE LENGTH=624

ATGGTGAGAGGGAAGATCGAGATCAAGAAGATTGAGAACGTGACGAGCAGACAAGTCACGTTCTCGAAGCGAAGAAGCGGTCTATTTAAGAAGGCTCATGAGCTTTCGGTTCTATGCGATGCTCAAGTAGCCGCCATTGTCTTTTCTCAGAGCGGAAGATTACACGAATACTCTAGCTCCCAGATGGAGAAGATTATAGATAGATATGGCAAGTTTAGTAATGCCTTCTATGTGGCAGAGAGGCCTCAAGTAGAACGATACTTGCAGGAGCTGAAGATGGAAATTGATAGAATGGTGAAAAAGATTGATCTCCTTGAAGTTCATCACCGTAAGCTGTTGGGGCAAGGTTTGGATTCGTGTTCAGTGACAGAACTTCAAGAGATTGACACTCAAATTGAGAAAAGCCTTCGTATTGTCAGGTCAAGAAAGGCTGAGTTATATGCAGATCAACTGAAAAAGCTAAAAGAAAAGGAGAGGGAGCTCTTGAACGAGAGAAAAAGGCTGCTTGAAGAGGTAAATATGCACCACTCATCAAAGGGTAATACTGAAGGTGGTCACAGAACCAAACATTCATCAGAGGTTGAAACCGACCTATTTATCGGATTGCCCGTGACTCGGCTATAA

>AT5G55690.1 | Symbols: AGL47 | agamous-like 47 | chr5:22548790-22549623 REVERSE LENGTH=834

ATGGGTCGAAAGATGGTAAAGATGACGAGGATAACGAACGAGAAAACGAGGATAACGACTTACAAGAAGAGGAAAGCATGTTTGTATAAGAAAGCCAGTGAGTTCTCAACACTCTGCGGTGTGGACACTTGTGTCATTGTGTACGGCCCGAGCAGAGCAGGGGACGAAATGGTCATGGAGCCCGAGTTATGGCCAAAGGATGGGAGCAAAGTCCGTGAAATCTTAACCAAGTACAGAGACACTGCGTCAAGCAGCTGCACCAAGACATACACCGTGCAAGAATGCTTGGAGAAAAACAACACTAAGGTGGAGAAACCGACGATTGCGACAAAGTATCCTACATGGGACAAAAAGCTCGACCAGTGTTCTTTAAATGACCTCTATGCGGTTTTCATGGCAGTAGAAAACAAGATCCAAGAGGCTACGAATAGGAATCAGACATTTCCTGACACTAGTTGTTGGTCTAATGACCAACTTGGTTTATGCGGTTACAATCGGCAATGTTTTGAGCAGTATCAGTTGTTTCCTCTGCCTACTATGGATTACAACGGGCTCTCTTTCTTCCCTTTTAATAACCAGATGACCTCAAATACTGCGGAAGTGTCTTCCTTCTCGAATGTGACAGAGCCGATGATAGCGAACGGGCAAAGCTTGTTTTACGGGAGTTGTTCGGATGGTCCATATGGTCCGATGGTACAGAGGACAGCTTATATGGAGCCAATACATTGGGGTTTAGGAAACAGTATGTTCAACAATGTGAAGCAGTTCCAAGATTATCCCTTCAGGTTTGCACAAGTTAATGATTTGGAGGATTCAAGTAAACTTTCTATGTGA

>AT5G58890.1 | Symbols: AGL82 | AGAMOUS-like 82 | chr5:23780832-23781716 FORWARD LENGTH=885

ATGGTTCCGAAAGTGGTCGACCTACAAAGGATAGCGAACGATAAGACAAGGATAACAACTTACAAGAAGAGGAAAGCTAGTCTTTACAAGAAGGCACAAGAGTTCTCAACTCTCTGCGGCGTCGAGACATGTCTCATCGTCTACGGTCCCACGAAGGCTACCGATGTGGTGATTTCCGAGCCAGAGATATGGCCGAAGGACGAGACCAAAGTCAGGGCCATCATACGCAAGTACAAAGACACAGTGTCGACCAGCTGCAGGAAAGAAACCAACGTGGAGACTTTCGTCAACGATGTAGGGAAAGGAAACGAGGTGGTGACTAAAAAGAGAGTGAAGCGTGAGAATAAGTATTCTAGTTGGGAGGAGAAGCTAGACAAGTGTTCACGAGAGCAACTACATGGGATTTTCTGTGCCGTGGATAGCAAGTTAAATGAAGCTGTAACGAGACAGGAGCGTAGTATGTTTAGGGTTAATCATCAAGCCATGGACACACCATTCCCGCAGAATTTAATGGACCAACAATTCATGCCACAGTATTTTCATGAGCAGCCACAGTTTCAAGGCTTCCCTAATAATTTCAATAATATGGGTTTCTCGTTGATTTCACCTCATGATGGTCAGATTCAAATGGACCCAAATCTCATGGAGAAGTGGACCGACTTGGCTTTGACTCAAAGCTTGATGATGTCAAAGGGAAACGATGGTACTCAATTCATGCAGAGGCAAGAACAACCATACTATAATCGTGAACAGGTTGTATCGAGGTCTGCAGGTTTCAATGTTAACCCGTTTATGGGATATCAAGTCCCGTTTAATATTCCTAATTGGAGATTATCGGGAAATCAAGTTGAAAATTGGGAGCTTTCAGGGAAGAAAACGATATGA

>AT5G60440.1 | Symbols: AGL62 | AGAMOUS-like 62 | chr5:24306329-24307520 FORWARD LENGTH=900

ATGGTGAAAAAAAGCAAAGGTCGTCAAAAAATAGAGATGGTCAAAATGAAAAATGAAAGTAACCTTCAAGTTACTTTTTCAAAAAGAAGATCTGGACTTTTCAAAAAGGCTAGTGAGCTTTGCACACTTTGTGGTGCAGAAGTGGCCATAGTTGTGTTCTCACCTGGTCGAAAAGTCTTTTCTTTTGGTCATCCAAATGTTGATTCTGTAATTGATCGATTCATAAACAATAACCCTCTACCTCCTCACCAACACAACAACATGCAACTTAGAGAAACTCGTCGAAATTCGATTGTTCAGGATCTGAATAATCATCTTACTCAGGTGTTGAGTCAATTAGAAACAGAGAAAAAGAAGTACGACGAGTTAAAGAAAATAAGAGAAAAGACAAAAGCCCTTGGGAATTGGTGGGAAGATCCCGTTGAGGAACTTGCGTTATCTCAACTCGAGGGGTTCAAAGGTAATCTTGAAAATTTGAAGAAAGTAGTTACAGTCGAAGCTTCCAGATTTTTTCAGGCAAATGTTCCAAACTTCTATGTGGGAAGTTCTAGTAATAATGCTGCTTTTGGGATTGATGATGGTAGTCATATCAACCCTGATATGGATCTCTTTAGCCAAAGAAGAATGATGGACATAAATGCCTTCAACTACAACCAGAACCAAATTCACCCTAATCATGCATTACCACCCTTTGGAAACAATGCTTATGGTATTAATGAAGGGTTTGTTCCAGAATACAATGTGAACTTCAGACCAGAGTATAACCCAAACCAAAACCAAATCCAAAACCAAAATCAAGTTCAAATCCAAATCCAAAACCAGAGTTTTAAGAGAGAAAACATCTCTGAATATGAACATCATCATGGTTATCCTCCCCAGTCTAGATCTGATTACTATTAA

>AT5G60910.1 | Symbols: FUL, AGL8 | FRUITFULL, AGAMOUS-like 8 | chr5:24502736-24506013 REVERSE LENGTH=729

ATGGGAAGAGGTAGGGTTCAGCTGAAGAGGATAGAGAACAAGATCAATAGGCAAGTTACTTTCTCAAAGAGAAGGTCTGGTTTGCTCAAGAAAGCTCATGAGATCTCTGTTCTCTGCGATGCTGAGGTTGCTCTCATCGTCTTCTCTTCCAAAGGCAAACTCTTCGAATATTCCACCGACTCTTGCATGGAGAGGATACTTGAACGCTATGATCGCTATTTATATTCAGACAAACAACTTGTTGGCCGAGACGTTTCACAAAGTGAAAATTGGGTTCTAGAACATGCTAAGCTCAAGGCAAGAGTTGAGGTACTTGAGAAGAACAAAAGGAATTTTATGGGGGAAGATCTTGATTCGTTGAGCTTGAAGGAGCTCCAAAGCTTGGAGCATCAGCTCGATGCAGCTATCAAGAGCATTAGGTCAAGAAAGAACCAAGCTATGTTCGAATCCATATCTGCGCTCCAGAAGAAGGATAAAGCCTTGCAAGATCACAACAATTCGCTTCTCAAAAAGATTAAGGAGAGGGAGAAGAAAACGGGTCAGCAAGAAGGACAATTAGTCCAATGCTCCAACTCTTCTTCAGTTCTTCTGCCTCAATACTGCGTAACCTCCTCCAGAGATGGCTTTGTGGAGAGAGTTGGGGGAGAGAACGGTGGTGCATCGTCGTTGACGGAACCAAACTCTCTGCTTCCGGCTTGGATGTTACGTCCTACCACTACGAACGAGTAG

>AT5G62165.1 | Symbols: AGL42, FYF | AGAMOUS-like 42, FOREVER YOUNG FLOWER | chr5:24965075-24968437 FORWARD LENGTH=633

ATGGTTAGAGGAAAGATAGAGATGAAGAAAATAGAAAACGCAACGAGTAGACAAGTGACTTTCTCAAAAAGAAGAAATGGTTTGTTGAAGAAAGCTTATGAGCTCTCAGTACTCTGCGATGCTCAACTCTCTCTCATCATCTTCTCCCAGAGAGGAAGGCTTTATGAATTCTCTAGCTCTGATATGCAGAAGACGATCGAACGCTACCGCAAGTACACAAAAGATCATGAAACCAGCAATCACGACTCACAAATTCACTTGCAGCAATTGAAACAAGAAGCAAGCCACATGATAACAAAGATTGAACTCCTTGAGTTTCACAAGCGGAAGCTATTGGGACAAGGAATTGCTTCTTGTTCTCTAGAAGAGCTTCAAGAAATCGATAGTCAACTCCAAAGAAGTCTGGGAAAGGTCCGAGAAAGAAAGGCTCAATTGTTCAAGGAGCAGTTGGAGAAACTAAAAGCAAAGGAGAAACAATTGTTAGAAGAGAACGTCAAGTTACATCAAAAGAATGTTATTAATCCATGGAGAGGATCATCGACTGATCAGCAGCAAGAGAAATACAAAGTTATAGATTTGAATTTGGAAGTTGAAACTGACTTATTCATCGGTTTGCCAAATAGAAACTGCTAG

>AT5G65050.1 | Symbols: MAF2, AGL31 | AGAMOUS-like 31, MADS AFFECTING FLOWERING 2 | chr5:25982415-25986114 FORWARD LENGTH=549

ATGGGTAGAAAAAAAGTCGAGATCAAGCGAATCGAGAACAAAAGTAGTCGACAAGTCACTTTCTCCAAACGACGCAATGGTCTCATCGAGAAAGCTCGACAACTTTCAATTCTCTGTGAATCTTCCATCGCTGTTCTCGTCGTCTCCGGCTCCGGAAAACTCTACAAGTCTGCCTCCGGTGACAACATGTCAAAGATCATTGATCGTTACGAAATACATCATGCTGATGAACTTGAAGCCTTAGATCTTGCAGAAAAAACTCGGAATTATCTGCCACTCAAAGAGTTACTAGAAATAGTCCAAAGCAAGCTTGAAGAATCAAATGTCGATAATGCAAGTGTGGATACTTTAATTTCTCTGGAGGAACAGCTCGAGACTGCTCTGTCCGTAACTAGAGCTAGGAAGACAGAACTAATGATGGGGGAAGTGAAGTCCCTTCAAAAAACGGTGGGGAAGAAGACGTTTCTGGTTATAGAAGGTGACAGAGGAATGTCATGGGAAAATGGCTCCGGCAACAAAGTACGGGAGACTCTTCCGCTGCTCAAGTAA

>AT5G65060.1 | Symbols: AGL70, FCL3, MAF3 | AGAMOUS-like 70, MADS AFFECTING FLOWERING 3 | chr5:25987527-25991065 FORWARD LENGTH=591

ATGGGAAGAAGAAAAGTCGAGATCAAGCGAATCGAGAACAAAAGCAGTCGACAAGTCACTTTCTCCAAACGACGCAAAGGTCTCATCGAAAAAGCTCGACAACTTTCAATTCTCTGTGAATCTTCCATCGCTGTTGTCGCCGTCTCCGGTTCCGGAAAACTCTACGACTCTGCCTCCGGTGACAACATGTCAAAGATCATTGATCGTTATGAAATACATCATGCTGATGAACTTAAAGCCTTAGATCTTGCAGAAAAAATTCGGAATTATCTTCCACACAAGGAGTTACTAGAAATAGTCCAAAGCAAGCTTGAAGAATCAAATGTCGATAATGTAAGTGTAGATTCTCTAATATCTATGGAGGAACAGCTCGAGACTGCTCTGTCAGTAATTAGAGCTAAGAAGACAGAACTAATGATGGAGGATATGAAGTCACTTCAAGAAAGGGAGAAGTTGCTGATAGAAGAGAACCAGATTCTGGCTAGCCAGGTGGGGAAGAAGACGTTTCTGGTTATAGAAGGTGACAGAGGAATGTCACGGGAAAATGGCTCCGGCAACAAAGTACCGGAGACTCTTTCGCTGCTCAAGTAA

>AT5G65070.1 | Symbols: MAF4, FCL4, AGL69 | AGAMOUS-like 69, MADS AFFECTING FLOWERING 4 | chr5:25992310-25995930 FORWARD LENGTH=603

ATGGGAAGAAGAAAAGTAGAGATCAAACGAATTGAGAACAAAAGCTCTCGACAAGTTACTTTCTGTAAACGACGAAATGGTCTCATGGAGAAAGCTCGTCAACTCTCAATTCTTTGTGAATCCTCCGTCGCTCTTATCATCATCTCTGCCACCGGAAGACTCTACAGCTTCTCCTCAGGTGATAGCATGGCCAAGATCCTCAGTCGTTATGAATTAGAACAGGCTGATGATCTTAAAACCTTGGATCTAGAAGAAAAAACTCTTAATTATCTTTCGCACAAGGAGTTGCTAGAAACAATCCAATGCAAGATTGAAGAAGCGAAAAGCGATAATGTAAGTATAGATTGTCTAAAGTCCCTGGAAGAGCAGCTCAAGACTGCTCTGTCTGTAACTAGAGCTAGGAAGACAGAACTAATGATGGAGCTTGTGAAGACCCATCAAGAGAAGGAGAAGCTGCTGAGAGAGGAGAACCAGAGTTTGACTAACCAGCTTATAAAGATGGGGAAGATGAAGAAGTCTGTGGAAGCAGAGGATGCAAGAGCAATGTCACCGGAAAGTAGCTCTGACAACAAGCCACCGGAGACTCTCCTGCTTCTCAAGTAA

>AT5G65080.1 | Symbols: MAF5, AGL68 | AGAMOUS-like 68, MADS AFFECTING FLOWERING 5 | chr5:26000866-26002211 FORWARD LENGTH=354

GATCTTGAAGACAAAACTCAGGATTATCTTTCACACAAGGAGTTACTAGAAATAGTTCAAAGAAAGATTGAAGAAGCAAAAGGGGATAATGTAAGTATAGAATCTCTAATTTCCATGGAAGAGCAGCTCAAGAGTGCTCTGTCTGTAATTAGAGCTAGGAAGACAGAGTTATTGATGGAGCTTGTGAAGAACCTTCAGGATAAGGAGAAGTTGCTGAAAGAAAAGAACAAGGTTCTAGCTAGCGAGGTGGGGAAGCTGAAGAAAATTTTGGAAACAGGGGATGAAAGAGCAGTAATGTCACCGGAAAATAGCTCTGGCCACAGCCCACCGGAGACTCTCCCGCTTCTCAAGTAA

>AT5G65330.1 | Symbols: AGL78 | AGAMOUS-like 78 | chr5:26110326-26111351 FORWARD LENGTH=1026

ATGAAGCAAGCTTCTTCTTCTTCTTCGTCACGTAATTCAACAAGTTTAACAAATAGACTTAAAACCATCTTCAAGAAAGCTGAAGAGCTTTCGATTCTCTGTGCTATTGAGGTTTGCGTCATCTATTACGGACCAGACGGTGAACTGAGAACATGGCCTAAGGAGAGAGAGACAGTGAAAGACATGGCTTTGAGGTATAAAGAGGCCAGAAAACGCAAGAAGAGCCGCAATCTTCATGAGTTCCTCGAGAAGGAGAAGGACAAGGACAAGGGGAAGACGAATTTGAAGAAGAATTGGTATCCAAACTTTGATCATTACTCTCCTCAACAACTCTCTCAATTGATTCAGTCCTTAGAACGGACTCTTTCTACTCTGCAAGAAAGGCTTCGTATTGTTGAGGCGCAGAAACTACAGAACACAAACTTGGTGCATCAGAGTTTAACACCATCGTATCTGAACCAGACCCAACACTTGAATCCTAGCAAGTTCTCGCTGTTTATGTACAACCATGGAGATGCTACTCTCTCACAACTCCCACTGTCTGCTCCACACTCCAATCAACTCATCAATTACCAGAATCACTTGATGCAGCATGGTTTTGGTCAGAACATGTGTTCGGACAACATCACCAACAACAACTTTGAACATCCTGGCGTGTCAAACACACAAGACTACTCACCGTTACTTTCGGTACAAGCATCTGCAGTGAATAACTACGGGTTGAATAATCACTTGATGCAGCAACAGGATCAGCTTCATGGTTTTGATCAGAACATGTGTATGGTGAGTGAAATCATCAACAACAACAACGGTCTACAACATCCTAATCTCTCAAACACGGTTCCACATGAATTCTCTTCTGATTTCAACCAGAACCCTTATGGTAATGCGGTCGGTAACATTAGCTTCTCTCAAGACATGTTTTCAAGCTATGATGCGAGCAGTCTGCTACAGACATCTTCTCTGCCACCTCTCCACAACATTCCTAGCAGTTATTGTTTTCCTGGCAACTCAAGACTTCTCTGA

**#amino acid sequences of MADS-box genes in *Arabidopsis thaliana***

>AT1G01530.1 | Symbols: AGL28 | AGAMOUS-like 28 | chr1:192640-193662 REVERSE LENGTH=744

MARKNLGRRKIELVKMTNESNLQVTFSKRRSGLFKKGSELCTLCDAEIAIIVFSPSGKAYSFGHPNVNKLLDHSLGRVIRHNNTNFAESRTKLRIQMLNESLTEVMAEKEKEQETKQSIVQNERENKDAEKWWRNSPTELNLAQSTSMKCDLEALKKEVDEKVAQLHHRNLNFYVGSSSNVAAPAAVSGGNISTNHGFFDQNGNSTSAPTLPFGFNVMNRTPAGYNSYQLQNQEVKQVHPQYWARYY*

>AT1G17310.1 | Symbols: AGL100 | agamous-like 100 | chr1:5928014-5928667 REVERSE LENGTH=654

MKDLFMEGERETSSMTCLTPKDSVQSPNMLVRQPKKETTTQTPKTTRGRQKIEIKKIEEETKRQVTFSKRRRGLFKKSAELSVLTGAKIAVITFSKCDRIYRFGHVDALIDKYLRKSPVKLEGYSGDNAADEESRRPWWERPVESVPEEELEEYMAALSMLRENIGKKIVAMGNDRTVDMVPAWPINVMGWKPTMDMQKLENLTDGVNRCRVGQNGD*

>AT1G18750.1 | Symbols: AGL65 | AGAMOUS-like 65 | chr1:6467266-6469640 FORWARD LENGTH=1170

MGRVKLKIKRLESTSNRQVTYTKRKNGILKKAKELSILCDIDIVLLMFSPTGRATAFHGEHSCIEEVISKFAQLTPQERTKRKLESLEALKKTFKKLDHDVNIHDFLGARNQTIEGLSNQVAIYQAQLMECHRRLSCWTNIDRIENTEHLDLLEESLRKSIERIQIHKEHYRKNQLLPIECATTQFHSGIQLPMAMGGNSSMQEAHSMSWLPDNDHQQTILPGDSSFLPHREMDGSIPVYSSCFFESTKPEDQICSNPGQQFEQLEQQGNGCLGLQQLGEEYSYPTPFGTTLGMEEDQEKKIKSEMELNNLQQQQQQQQQQQQQDPSMYDPMANNNGGCFQIPHDQSMFVNDHHHHHHHHHQNWVPDSMFGQTSYNQVCVFTPPLELSR*

>AT1G22130.1 | Symbols: AGL104 | AGAMOUS-like 104 | chr1:7812387-7814259 REVERSE LENGTH=1008

MGRVKLEIKRIENTTNRQVTFSKRRNGLIKKAYELSILCDIDIALIMFSPSDRLSLFSGKTRIEDVFSRFINLPKQERESALYFPDQNRRPDIQNKECLLRILQQLKTENDIALQVTNPAAINSDVEELEHEVCRLQQQLQMAEEELRRYEPDPIRFTTMEEYEVSEKQLLDTLTHVVQRRDHLMSNHLSSYEASTMQPNIGGPFVNDVVEGWLPENGTNQTHLFDASAHSNQLRELSSAMYEPLLQGSSSSSNQNNMSECHVTNHNGEMFPEWAQAYSSSALFASMQQQHEGVGPSIEEMMPAQQSDIPGVTAETQVDHEVSDYETKVPQLSSQ*

>AT1G22590.2 | Symbols: AGL87 | AGAMOUS-like 87 | chr1:7983511-7984002 FORWARD LENGTH=492

MGRRKVTHQLISDNATRRVTFRKRKDGLLKKIYELTVLCGLPACAIIYSEYKDGPELWPNLNEVRSILNRLSELPVEKQTKYMMDQKDLMNKMIQDAEKKLEKEKMHTRAMKLGLMAGSNDLITDTDCSEELARAADVVDKKLKAIRERIKAVEAGAPIIKRD*

>AT1G24260.1 | Symbols: AGL9, SEP3 | SEPALLATA3, AGAMOUS-like 9 | chr1:8593790-8595862 REVERSE LENGTH=753

MGRGRVELKRIENKINRQVTFAKRRNGLLKKAYELSVLCDAEVALIIFSNRGKLYEFCSSSSMLRTLERYQKCNYGAPEPNVPSREALAELSSQQEYLKLKERYDALQRTQRNLLGEDLGPLSTKELESLERQLDSSLKQIRALRTQFMLDQLNDLQSKERMLTETNKTLRLRLADGYQMPLQLNPNQEEVDHYGRHHHQQQQHSQAFFQPLECEPILQIGYQGQQDGMGAGPSVNNYMLGWLPYDTNSI*

>AT1G26310.1 | Symbols: CAL1, AGL10, CAL | AGAMOUS-like 10, CAULIFLOWER | chr1:9100330-9103510 REVERSE LENGTH=768

MGRGRVELKRIENKINRQVTFSKRRTGLLKKAQEISVLCDAEVSLIVFSHKGKLFEYSSESCMEKVLERYERYSYAERQLIAPDSHVNAQTNWSMEYSRLKAKIELLERNQRHYLGEELEPMSLKDLQNLEQQLETALKHIRSRKNQLMNESLNHLQRKEKEIQEENSMLTKQIKERENILRTKQTQCEQLNRSVDDVPQPQPFQHPHLYMIAHQTSPFLNMGGLYQEEDQTAMRRNNLDLTLEPIYNYLGCYAA*

>AT1G28450.1 | Symbols: AGL58 | AGAMOUS-like 58 | chr1:10003966-10004523 FORWARD LENGTH=558

MNPKKTKGKQKINIKKIEKDEDRSVTLSKRLNAIYTMIIELSILCGVEVAFIGYSCSGKPYTFGSPSFQAVVERFLNGEASSSSSSSLQRSVKNAHKQAKIQELCKRYNRLVEELKVDEVKVKKAAALAETRAVNKDAWWKADPNDVKDHEKAKKMMEKYQELKEKLREEVALRIKRGHDENNNK*

>AT1G28460.1 | Symbols: AGL59 | AGAMOUS-like 59 | chr1:10006230-10006778 FORWARD LENGTH=549

MNPKKTKGKQKINIKKIEKDEGRSVTFSKRLNGIYTKISELSILCGVEVAFIGYSCSGKPYTFGSPSFQAVAERFLNGDASSSSSSSLVMNAHKQAKIQELCKKYNRLVEELKVDEVKVKKAAALAETRVVNKDVWWKVDPNDVKDHEKAKKMMEKYQELYDKLCEQAASRIKRGHDENNNK*

>AT1G29962.1 | Symbols: AGL64 | AGAMOUS-like 64 | chr1:10496730-10497287 FORWARD LENGTH=558

MKPKKTKGKQRINIKKIEKDEDRLVTLSKRRNGIYTKLSELSILCGAEVAFLGYSCSGKPYTFGSPSFQAVAERFLNGEASSSSSSSLQRSVMNAHQQAKIQELCKVYNRLVEEITVEEVKLKKTAALAEMMPMNEDAWWKVDPNDVKDREEVKKMMEKHQELYEKLCEEAASRIKRGHDENNNK*

>AT1G31140.1 | Symbols: GOA, AGL63 | AGAMOUS-like 63, GORDITA | chr1:11118031-11119673 FORWARD LENGTH=642

MRKGKRVIKKIEEKIKRQVTFAKRKKSLIKKAYELSVLCDVHLGLIIFSHSNRLYDFCSNSTSMENLIMRYQKEKEGQTTAEHSFHSCSDCVKTKESMMREIENLKLNLQLYDGHGLNLLTYDELLSFELHLESSLQHARARKSEFMHQQQQQQTDQKLKGKEKGQGSSWEQLMWQAERQMMTCQRQKDPAPANEGGVPFLRWGTTHRRSSPP*

>AT1G31630.1 | Symbols: AGL86 | AGAMOUS-like 86 | chr1:11318528-11319547 REVERSE LENGTH=1020

MRSKIKLSLIANKTSRRTTFRKRKGGITNKLHELTTLCGVKACAVISSPYENPVVWPSTEGVQEAVSMFMERPATEQSKLMMSHETYLQDKITKETKKLESLRRENRESQLRQFMFDCVEGKMSEHQYGARDLQDLSLYIDHYINQLNSSVMLLTNNGASSSSFPPPLHTSVAGAGAGAGAAPLVVAGAGAAPLAVAGAGASPLAVAGVGAAPLAVAGAGPPMAQNQYEPIQPYIPTAFSDNIQYQAPVDFNHQIQHGIYDNLSLDPNHQYPFQDDPFMEMLMEYPYEQVGYAAEHAHIPFMNGNYYNYHQPPTVGLTTTGHMPSNNATTTTTTNTTVV*

>AT1G31640.1 | Symbols: AGL92 | AGAMOUS-like 92 | chr1:11322692-11324176 REVERSE LENGTH=1395

MRTKTKLVLIPDRHFRRATFRKRNAGIRKKLHELTTLCDIKACAVIYSPFENPTVWPSTEGVQEVISEFMEKPATERSKTMMSHETFLRDQITKEQNKLESLRRENRETQLKHFMFDCVGGKMSEQQYGARDLQDLSLFTDQYLNQLNARKKFLTEYGESSSSVPPLFDVAGANPPVVADQAAVTVPPLFAVAGANLPVVADQAAVTVPPLFAVAGANLPVVADQAAVNVPTGFHNMNVNQNQYEPVQPYVPTGFSDHIQYQNMNFNQNQQEPVHYQALAVAGAGLPMTQNQYEPVHYQSLAVAGGGLPMSQLQYEPVQPYIPTVFSDNVQYQHMNLYQNQQEPVHYQALGVAGAGLPMNQNQYEPVQPYVPTGFSDHFQFENMNLNQNQQEPVQYQAPVDFNHQIQQGNYDMNLNQNMKHAHIPFMDGNYYNYHQPPTVGLTSTGHMPSTTTTTTNNNNNNNV*

>AT1G33070.1 | Symbols: no symbol available | no full name available | chr1:11982889-11985299 FORWARD LENGTH=498

MPMRKEGITKKLYELATLCDIKACAGKISEHQYGARDLQDLSFHIDHYINQLNSRVKILTNNGESSSSVPPLLHTSVAGAGAAPLPVAGADLPMDQNQYEPIQLYIPIGLSYHIQYQHEIYDNFKHAHILFMNGNYYNYHQTPTVGLTTTGHMPSNNNTTPTTDV*

>AT1G46408.1 | Symbols: AGL97 | AGAMOUS-like 97 | chr1:17232135-17232935 REVERSE LENGTH=801

MGGVKRKIAIEKIQNKNPRAVSFSKRRKGLYSKASELCLLSDAEIAIIATPVSSNSNAAFYSFGHSSVDNVVAAFLANQRPCDERFWWEDESLLKSENLEELREAMDSMSTMLRDLKELEKQRDHQTQTLIHQPCSARVCIQDYVTVNFDGFNTEEQTLAVSDNSNNNGLLGNLDECNEDFDDLDQIFDTVTNSEFLSVNLEMDDVTVNSEGNTEEQTLAVSDNSNNNGLLGNLDECNEDFDDLDQIIEYLTSSEALSMNLKMDDV*

>AT1G47760.1 | Symbols: AGL102 | AGAMOUS-like 102 | chr1:17572451-17573159 FORWARD LENGTH=555

MGRRKIEIKFIEDSIERKATFSRRRNGIFKKADELAKLCNVEIAVLVISPTNIPYTYGYPCFNDVVERIQNPSASSKLRSLMKELEQIKEFQEDLRKKQQRNLEKSNMKENVDLKLEDLVAFKAKLEAYQAGLKRKHVEMEDLSSPSILSKNTKNKMMRTEYSSGQSKGMYEFRAFGPGFLGTI*

>AT1G48150.1 | Symbols: AGL74 | agamous-like 74 | chr1:17785397-17786368 FORWARD LENGTH=972

MQSSNVTDCTMRKRGTKRKIEIEKRMTKQQRSVACSKRRPTLFSKAADLCLLSGANIAVFVTSPDENSDVVYSFSGYSHASEIVDCYLNNKSPPKTTINPESAKFWWEEPDLYRDCDDLSELRIIEDRLMRTKKHLMDYLEKKEKSHSVSKSDQNPNNDSGSSSSSSQIASDFGQNPSTLSPSSLKIVSFDQNSYSSLEPSSQVTTCFDQNPVFSVGSESSSDQSRYLVNEDSGFVDGLLCETEEENNGMRLPQETQTQPMFTEEDQSFWENLDVDDVFGLFKDDNNLEVPLQDHSSTNEDDELLIDISEYLSEEAMECPCFS*

>AT1G54760.1 | Symbols: AGL85 | AGAMOUS-like 85 | chr1:20433912-20434397 FORWARD LENGTH=486

MKTDWSHYLSVEMESTISNELSILCGAEVAFLGYSCSGKPYTFGSPSFQAVAERFLNREASSSLQRSVMNAHQQAKIQELCKVYNRMVEEAKTEEAKVKKAAALAETMPVDEDAWWKVDPKEVEDHEEAKKIMEKCEGLYEKLCNEAAARIQRGDAENNNK*

>AT1G59810.1 | Symbols: AGL50 | AGAMOUS-like 50 | chr1:22008604-22009455 FORWARD LENGTH=852

MAPRQKKPNKSDDDDDLRRKKQSFFKQRFPGFKKKASELSVLCGNSVGFICYGPDSDLHVWPQSQDHNPQALHEIVAKFNALSDERRKNHACDLNDFPHHLKGLSREELRKHLLHLDSQLLGVREQKIEILKKTLTGSSEKDGARVSENSAISDHKLKIEPHLKDILSEDHLIRVSDKKLGSCDVFDELAYVVRGSRNLNENVSKYESKDADNTGLDHLVTLGGDYLQEAAAELYQTYNLGNFCDDHVWDLEFASRLPLLHTFSDPLMTTNTCQTMSTDMISI*

>AT1G60040.1 | Symbols: AGL49 | AGAMOUS-like 49 | chr1:22119075-22119929 REVERSE LENGTH=855

MAPRQKKPNKSDDDDGDLHRKKQSFFKQRFPGFKKKASELSVLCGNSVGFICYGPDNDLHVWPQSQDHNPQALHEIVAKFNALSDERRKNHACDLNDFPHHLKGLSREELRKHLLHLDSQLLGVREQKIEILKKTLTGSSEKDGARVSENSAISDHKLKIEPNLTDILSEDHLIRVSDKKLGSCDVFDELAYVVRGSRNLNENVSNYESKDAAYTGMDHLGTFGGNYLQEAAAELYQTYNLGNFCDDHVWDLEFASRLPPLHTFSDPLMTTNTCQTMSSDMISI*

>AT1G60880.1 | Symbols: AGL56 | AGAMOUS-like-56 | chr1:22411575-22412180 FORWARD LENGTH=606

MGGKKTKIEIKKIINKPAKTVAFTKRREGLFRKASQLCLLSPATQIAILAAPMTSKSHASFYSFGHSSVDNVVSSLLYDHPPLTANQDNRSGLGFWWEDKRFDVSENVEELKEAVDAVSRMLNNVRCRLNDAVKSTQRDGGLEILHHQEEEVLQTRNDETKTNQTHEFEGGETSGSASWLENEDDILHFDDDFYTGIDPLF*

>AT1G60920.1 | Symbols: AGL55 | AGAMOUS-like 55 | chr1:22429692-22430267 REVERSE LENGTH=576

MGGTKRKIEMKRIEDKNVRAVAFTKRKSGLFHKASELCLLSPGTQIAILATPLSSHSHASFYSFGHSSVDHVVSSLLHNQHPSLPTNQDNRSGLGFWWEDQAFDRLENVDELKEAVDAVSRMLNNVRLRLDDAVKSNQRDGSLVIHQEDEEVLQLGYKDTNQITKLEGETSASASLLKNVVDNLHIDDRYY*

>AT1G65300.1 | Symbols: PHE2, AGL38 | PHERES2, AGAMOUS-like 38 | chr1:24254929-24255765 FORWARD LENGTH=837

MKRKMKLSLIENSVSRKTTFTKRKKGMTKKLTELVTLCGVEACAVVYSPFNSIPEAWPSREGVEDVVSKFMELSVLDRTKKMVDQETFISQRIAKEKEQLQKLRDENHNSQIRELMFGCLKGETNVYNLDGRDLQDLSLYIDKYLNGLTRRIEILIENGESSSSLPLPIVANAAAPVGFDGPMFQYHNQNQQKPVQFQYQALYDFYDQIPKKIHGFNMNMNKDSNQSMVLDLNQNLNDGEDEGIPCMDNNNYHPEIDCLATVTTAPTDVCAPNITNDL*

>AT1G65330.1 | Symbols: PHE1, AGL37 | AGAMOUS-like 37, PHERES1 | chr1:24266481-24267320 REVERSE LENGTH=840

MRGKMKLSFIENDSVRKTTFTKRKKGMLKKFNELVTLCGVDACAVIRSPYNSIQEPWPSREGVEEVMSKFMEFSVLDRTKKMVDQETFLRQRIAKETERLQKLRDENRNSQIRDLMFGCLKGEVDVSHLHGRDLLDLNVFLNKYLNGVIRRVEILKENGESSSSVPPPIGVAPTVVDASVPIGFDGRMIQDQNQNQQEPVQFQYQALYDFYDQIPKKLHDFNMKMNIDPNQSMNLDLNDGEDEGIPCMDNNNYHPEIDCLATVTTAPTDVCAPNIINDL*

>AT1G65360.1 | Symbols: AGL23 | AGAMOUS-like 23 | chr1:24281337-24282151 FORWARD LENGTH=681

MVKKTLGRRKVEIVKMTKESNLQVTFSKRKAGLFKKASEFCTLCDAKIAMIVFSPAGKVFSFGHPNVDVLLDHFRGCVVGHNNTNLDESYTKLHVQMLNKSYTEVKAEVEKEQKNKQSRAQNERENENAEEWWSKSPLELNLSQSTCMIRVLKDLKKIVDEKAIQLIHQTNPNFYVGSSSNAAAPATVSGGNISTNQGFFDQNGMTTNPTQTLLFGFDIMNRTPGV*

>AT1G69120.1 | Symbols: AP1, AtAP1, AGL7 | AGAMOUS-like 7, APETALA1 | chr1:25982576-25986102 REVERSE LENGTH=771

MGRGRVQLKRIENKINRQVTFSKRRAGLLKKAHEISVLCDAEVALVVFSHKGKLFEYSTDSCMEKILERYERYSYAERQLIAPESDVNTNWSMEYNRLKAKIELLERNQRHYLGEDLQAMSPKELQNLEQQLDTALKHIRTRKNQLMYESINELQKKEKAIQEQNSMLSKQIKEREKILRAQQEQWDQQNQGHNMPPPLPPQQHQIQHPYMLSHQPSPFLNMGGLYQEDDPMAMRRNDLELTLEPVYNCNLGCFAA*

>AT1G69540.1 | Symbols: AGL94 | AGAMOUS-like 94 | chr1:26145306-26147159 REVERSE LENGTH=1035

MGRVKLKIKKLQNMNGRQCTYTKRRHGIMKKAKELSILCDIDVVLLMFSPMGKASICIGKHSIGEVIAKFAQLSPQERAKRKLENLEALRKTFMKANHDIDISKFLDRISTPTVEVLSEKIRFLQTQLSDIHTRLSYWTDVDNIDSVDVLQQLEHSLRQSLAQIYGRKASMPQRQQQQLMSSQCKNQLQTEIDIDFGMEMEQQLENFSWVRTDENMNVPIEEEDPNLQLHHMYKDITCSASSALGNYSGLFSKSSDILQKLETGSIPGTSADPNQQFSNLSFLNDQKLKQLAEWNLLGSPADYYVSQILEASYKPQIGGKNNGASSETLPYVAVFDDPLYFWPN*

>AT1G71692.1 | Symbols: XAL1, AGL12 | XAANTAL1, AGAMOUS-like 12 | chr1:26952903-26954939 REVERSE LENGTH=636

MARGKIQLKRIENPVHRQVTFCKRRTGLLKKAKELSVLCDAEIGVVIFSPQGKLFELATKGTMEGMIDKYMKCTGGGRGSSSATFTAQEQLQPPNLDPKDEINVLKQEIEMLQKGISYMFGGGDGAMNLEELLLLEKHLEYWISQIRSAKMDVMLQEIQSLRNKEGVLKNTNKYLLEKIEENNNSILDANFAVMETNYSYPLTMPSEIFQF*

>AT1G72350.1 | Symbols: AGL60 | agamous-like 60 | chr1:27239273-27239947 REVERSE LENGTH=675

MEDGEASTITFLPTTEPKPLQNPNLLAKPKKETKQKKPKTTKGRQKIEIKEIMLETRRQVTFSKRRSGLFKKAAELSVLCGAQIGIITFSRCDRIYSFGNVNSLIDKYLRKAPVMLRSHPGGNVANGEEDNDGLMWWERAVESVPEEHMEEYKNALSVLRENLLTRIYQMSGDRTVENLPAFPNEMAMADWKLTNENLMARNDRGYGGNNGDLEFAFMPQNGRQ*

>AT1G77080.2 | Symbols: FLM, AGL27, MAF1 | FLOWERING LOCUS M, AGAMOUS-like 27, MADS AFFECTING FLOWERING 1 | chr1:28955679-28959845 FORWARD LENGTH=579

MGRRKIEIKRIENKSSRQVTFSKRRNGLIDKARQLSILCESSVAVVVVSASGKLYDSSSGDEIEALFKPEKPQCFELDLEEKIQNYLPHKELLETVQSKLEEPNVDNVSVDSLISLEEQLETALSVSRARKAELMMEYIESLKEKEKLLREENQVLASQMGKNTLLATDDERGMFPGSSSGNKIPETLPLLN*

>AT1G77950.1 | Symbols: AGL67 | AGAMOUS-like 67 | chr1:29307029-29309667 FORWARD LENGTH=759

MGRVKLELKRIEKSTNRQITFSKRKKGLIKKAYELSTLCDIDLALLMFSPSDRLCLFSGQTRIEDVLARYINLPDQERENAIVFPDQSKRQGIQNKEYLLRTLEKLKIEDDMALQINEPRPEATNSNVEELEQEVCRLQQQLQISEEELRKFEPDPMRLTSMEEIEACEANLINTLTRVVQRREHLLRKSCEAQSNQQSMDGILLNDIVEDWGPEPEPKQAHMIANSAHHSNQPSYDLLLRRSNSSSNQNPK*

>AT1G77980.1 | Symbols: AGL66 | AGAMOUS-like 66 | chr1:29315212-29317067 REVERSE LENGTH=999

MGRVKLEIKRIENTTNRQVTFSKRRNGLIKKAYELSILCDIDIALLMFSPSDRLSLFSGKTRIEDVFSRYINLSDQERENALVFPDQSRRPDFQSKEYLLRTLQQLKAENDIALQLTNPTAINSDVEELEHEVYKLQQQLLMAEEELRKYEPDPIRFTTMEEYETCEKQLMDTLTRVNQRREHILSQDQLSSYEASALQQQQSMGGPFGNDVVGGWLTENGPNEAHLFDASAHSAMYETLLQGSSSSSNQNNIMGESNVSNHNGDMFQEWAQAYNSTTAHNPSTLFPPMQHQHGLVVDPNIEEIEIPVMKKDAQADHEVSDYDIRMPQLSSQ*

>AT2G03060.1 | Symbols: AGL30 | AGAMOUS-like 30 | chr2:901614-903639 FORWARD LENGTH=1146

MGRVKLKIKKLENTNGRQSTFAKRKNGILKKANELSILCDIDIVLLMFSPTGKAAICCGTRSSMEEVIAKFSQVTPQERTKRKFESLENLKKTFQKLDHDVNIREFIASSNSTVEDLSTQARILQARISEIHGRLSYWTEPDKINNVEHLGQLEISIRQSLDQLRAHKEHFGQQQQAMQIENANFVKDWSTCSMQDGIQIPLEQQLQSMSWILNSNTTNIVTEEHNSIPQREVECSASSSFGSYPGYFGTGKSPEMTIPGQETSFLDELNTGQLKQDTSSQQQFTNNNNITAYNPNLHNDMNHHQTLPPPPLPLTLPHAQVYIPMNQREYHMNGFFEAPPPDSSAYNDNTNQTRFGSSSSSLPCSISMFDEYLFSQMQQPN*

>AT2G03710.1 | Symbols: SEP4, AGL3 | SEPALLATA 4, AGAMOUS-like 3 | chr2:1129622-1131628 FORWARD LENGTH=777

MGRGKVELKRIENKINRQVTFAKRRNGLLKKAYELSVLCDAEIALLIFSNRGKLYEFCSSPSGMARTVDKYRKHSYATMDPNQSAKDLQDKYQDYLKLKSRVEILQHSQRHLLGEELSEMDVNELEHLERQVDASLRQIRSTKARSMLDQLSDLKTKEEMLLETNRDLRRKLEDSDAALTQSFWGSSAAEQQQQHQQQQQGMSSYQSNPPIQEAGFFKPLQGNVALQMSSHYNHNPANATNSATTSQNVNGFFPGWMV*

>AT2G14210.1 | Symbols: ANR1, AtANR1, AGL44 | ARABIDOPSIS NITRATE REGULATED 1, AGAMOUS-like 44 | chr2:6018841-6023585 FORWARD LENGTH=705

MGRGKIVIRRIDNSTSRQVTFSKRRSGLLKKAKELSILCDAEVGVIIFSSTGKLYDYASNSSMKTIIERYNRVKEEQHQLLNHASEIKFWQREVASLQQQLQYLQECHRKLVGEELSGMNANDLQNLEDQLVTSLKGVRLKKDQLMTNEIRELNRKGQIIQKENHELQNIVDIMRKENIKLQKKVHGRTNAIEGNSSVDPISNGTTTYAPPQLQLIQLQPAPREKSIRLGLQLS*

>AT2G22540.1 | Symbols: AGL22, FAQ1, SVP | SHORT VEGETATIVE PHASE, Flowering Arabidopsis QTL1, AGAMOUS-like 22 | chr2:9580417-9583603 FORWARD LENGTH=723

MAREKIQIRKIDNATARQVTFSKRRRGLFKKAEELSVLCDADVALIIFSSTGKLFEFCSSSMKEVLERHNLQSKNLEKLDQPSLELQLVENSDHARMSKEIADKSHRLRQMRGEELQGLDIEELQQLEKALETGLTRVIETKSDKIMSEISELQKKGMQLMDENKRLRQQGTQLTEENERLGMQICNNVHAHGGAESENAAVYEEGQSSESITNAGNSTGAPVDSESSDTSLRLGLPYGG*

>AT2G22630.1 | Symbols: AGL17 | AGAMOUS-like 17 | chr2:9618372-9621641 FORWARD LENGTH=684

MGRGKIVIQKIDDSTSRQVTFSKRRKGLIKKAKELAILCDAEVCLIIFSNTDKLYDFASSSVKSTIERFNTAKMEEQELMNPASEVKFWQREAETLRQELHSLQENYRQLTGVELNGLSVKELQNIESQLEMSLRGIRMKREQILTNEIKELTRKRNLVHHENLELSRKVQRIHQENVELYKKAYGTSNTNGLGHHELVDAVYESHAQVRLQLSQPEQSHYKTSSNS*

>AT2G24840.1 | Symbols: DIA, AGL61 | DIANA, AGAMOUS-like 61 | chr2:10581082-10581876 FORWARD LENGTH=795

MYVTKYKNKLPTLQVDLIIMPHQTQACIYKQTLSLHQIFQQRKATKTLHTKKTMMSKKKESIGRQKIPMVKIKKESHRQVTFSKRRAGLFKKASELCTLCGAEIGIIVFSPAKKPFSFGHPSVESVLDRYVSRNNMSLAQSQQLQGSPAASCELNMQLTHILSEVEEEKKKGQAMEEMRKESVRRSMINWWEKPVEEMNMVQLQEMKYALEELRKTVVTNMASFNEAKDDVFGFLDNKVTVPPYVNMPSGPSNIYNFANGNGCF*

>AT2G26320.1 | Symbols: AGL33 | AGAMOUS-like 33 | chr2:11205389-11206287 REVERSE LENGTH=330

MKRTIKNKNKQIVKENMGRKKLKLKRIESLKERSSKFSKRKKGLFKKAEEVALLCDSDIMLIVVSPTEKPTVFNTRSRSFHTILERFCMLSLQEREERCDLSYFYIIIT*

>AT2G28700.1 | Symbols: AGL46 | AGAMOUS-like 46 | chr2:12317384-12318724 REVERSE LENGTH=990

MARKKLNLTYIFNDRMRKRSFKQRREGFLKKLNDLKVLCDVNACAVVYNPFNSNPDVWPSKSEVNNIIKKFEMLPETQKKVKSVNHEEFLNLYISKVEKQSKKLIVENKETCLKEVMFKCLGGNMGDFVMNDNDRLDLCKFIDHYLRNLYHHKNVTLNNPNFEIGESSSLMDMAPTATTGNMATTVVDEGMTPLLIAEGSSSSFLNSPLFNSPQLTNELQLIVSQNHRLENSLASNLFFSEGQDICIPDMNQSIIPSNQGAEHVDFLESNFLPNNNQEVYIPVMDQDEVYNPNQNHYENQQGFIDEMMKYAEKTSFPWMVENHCYNHNQ*

>AT2G34440.1 | Symbols: AGL29 | AGAMOUS-like 29 | chr2:14526950-14527468 FORWARD LENGTH=519

MGRRKIKMEMVQDMNTRQVTFSKRRTGLFKKASELATLCNAELGIVVFSPGGKPFSYGKPNLDSVAERFMREYDDSDSGDEEKSGNYRPKLKRLSERLDLLNQEVEAEKERGEKSQEKLESAGDERFKESIETLTLDELNEYKDRLQTVHGRIEGQVNHLQASSCLMLLSRK*

>AT2G40210.1 | Symbols: AGL48 | AGAMOUS-like 48 | chr2:16793213-16794328 REVERSE LENGTH=1116

MTRKKVKLVWIENDKSRATSLQKMRVGLLKKVKELTILCAVRAIVIIFSPDKVGPLVWPSPQATHGLLDEFFALPKSVQKKKESNVESYLKEKTHKFQEQLKKSKKKNKEHVIDELMMQLQSGREIADLNQSEMYALLSFSRDTILLCRKKLAFMQFPPLRDPPVFPFEIQVEEFKTTTNDGFVGGGQDNKRAGRTDEATRFINTDIFKQSKSYYFFDEWVFPPSPPKYEIPQQMENGNPNPKSYRLYQGSSSNGNPHLEMDPFRLQMMTSQGLAGSVSQPLQHHSMINNPTMAMNQPSQDPFDYMRSELGINEGININNSQFYMSNNTITANDGVRQEPYPNVTTAGENNGDATTSNTNMVWPGFNNHHF*

>AT2G42830.1 | Symbols: AGL5, SHP2 | AGAMOUS-like 5, SHATTERPROOF 2 | chr2:17820602-17823806 FORWARD LENGTH=741

MEGGASNEVAESSKKIGRGKIEIKRIENTTNRQVTFCKRRNGLLKKAYELSVLCDAEVALVIFSTRGRLYEYANNSVRGTIERYKKACSDAVNPPTITEANTQYYQQEASKLRRQIRDIQNLNRHILGESLGSLNFKELKNLESRLEKGISRVRSKKHEMLVAEIEYMQKREIELQNDNMYLRSKITERTGLQQQESSVIHQGTVYESGVTSSHQSGQYNRNYIAVNLLEPNQNSSNQDQPPLQLV*

>AT2G45650.1 | Symbols: AGL6, RSB1 | AGAMOUS-like 6, REDUCED SHOOT BRANCHING 1 | chr2:18804453-18806291 FORWARD LENGTH=759

MGRGRVEMKRIENKINRQVTFSKRRNGLLKKAYELSVLCDAEVALIIFSSRGKLYEFGSVGIESTIERYNRCYNCSLSNNKPEETTQSWCQEVTKLKSKYESLVRTNRNLLGEDLGEMGVKELQALERQLEAALTATRQRKTQVMMEEMEDLRKKERQLGDINKQLKIKFETEGHAFKTFQDLWANSAASVAGDPNNSEFPVEPSHPNVLDCNTEPFLQIGFQQHYYVQGEGSSVSKSNVAGETNFVQGWVL*

>AT2G45660.1 | Symbols: AGL20, SOC1, ATSOC1 | SUPPRESSOR OF OVEREXPRESSION OF CO 1, AGAMOUS-like 20 | chr2:18807799-18810193 REVERSE LENGTH=645

MVRGKTQMKRIENATSRQVTFSKRRNGLLKKAFELSVLCDAEVSLIIFSPKGKLYEFASSNMQDTIDRYLRHTKDRVSTKPVSEENMQHLKYEAANMMKKIEQLEASKRKLLGEGIGTCSIEELQQIEQQLEKSVKCIRARKTQVFKEQIEQLKQKEKALAAENEKLSEKWGSHESEVWSNKNQESTGRGDEESSPSSEVETQLFIGLPCSSRK*

>AT3G02310.1 | Symbols: AGL4, SEP2 | AGAMOUS-like 4, SEPALLATA 2 | chr3:464554-466687 REVERSE LENGTH=753

MGRGRVELKRIENKINRQVTFAKRRNGLLKKAYELSVLCDAEVSLIVFSNRGKLYEFCSTSNMLKTLERYQKCSYGSIEVNNKPAKELENSYREYLKLKGRYENLQRQQRNLLGEDLGPLNSKELEQLERQLDGSLKQVRCIKTQYMLDQLSDLQGKEHILLDANRALSMKLEDMIGVRHHHIGGGWEGGDQQNIAYGHPQAHSQGLYQSLECDPTLQIGYSHPVCSEQMAVTVQGQSQQGNGYIPGWML*

>AT3G04100.1 | Symbols: AGL57 | AGAMOUS-like 57 | chr3:1075299-1075922 FORWARD LENGTH=624

MSSTKQAKGRKTKGKQKIEMKKVENYGDRMITFSKRKTGIFKKMNELVAMCDVEVAFLIFSQPKKPYTFAHPSMKKVADRLKNPSRQEPLERDDTRPLVEAYKKRRLHDLVKKMEALEEELAMDLEKLKLLKESRNEKKLDKMWWNFPSEGLSAKELQQRYQAMLELRDNLCDNMAHLRLGKDCGGSSSVRVGRRVSGGVRLFDREA*

>AT3G05860.1 | Symbols: AGL45 | agamous-like 45 | chr3:1751406-1752355 REVERSE LENGTH=783

MTRKKLNLSYITNESMRKATFNKRKKGLVKKIHELSVLCGIEACAVIYSPFNSNPEVWPSNSEVKNVMENFEMLTKLEQEKKMVSHEGFIRQNISKTMESNNKKMIDNAERTMKEAMFQLLSGKGEKLNLTDRNREDLCKYIDQYLKELYHHKNKTINQSHIEPGESSGATNAMTPTSVVEPIISSIQRPNQNPNFNHLSHNQYQYQQQFGYPILVQDGIYNPSQIQNQHEEWLDDHMMNHSKEISHPLMDDNNFYYQQP*

>AT3G18650.1 | Symbols: AGL103 | AGAMOUS-like 103 | chr3:6417344-6418504 REVERSE LENGTH=1161

MASSSSSSLSFSTSKKNKTFFKKPNSAFSSSRATSLIKRQQTVFKKAKELSILCDIDVCVICYGSNGELKTWPEEREKVKAIARRYGELSETKRRKGSVDLHEFLEKMNKDDPEKEEKKKIKVRRVPKVKYPVWDPRFDNYSVEQLMGLVQSLERNLTRIQHRTCAVVEAQGQRRVQYTNMANQELMMANTMNQLQQHSNQVSMYLWNHGNGAFSQIPVSALASNQTQSLAPIPPELMIYPNSDAGNYSGSLGVQGTGINGLQNMNMLTYNNINSVNDFSKQFDQNSRAESYSSLLGVHEDGNNEFENPNMSSRNNFNVQDCAGLLGMQGAGTNGLQSMNMHDYSNNNSINSNGLSHQYVQFPTYNSQHQDRVFNLDQNGNNTRSL*

>AT3G30260.1 | Symbols: AGL79 | AGAMOUS-like 79 | chr3:11909119-11912880 FORWARD LENGTH=750

MGRGRVQLRRIENKIRRQVTFSKRRTGLVKKAQEISVLCDAEVALIVFSPKGKLFEYSAGSSMERILDRYERSAYAGQDIPTPNLDSQGECSTECSKLLRMIDVLQRSLRHLRGEEVDGLSIRDLQGVEMQLDTALKKTRSRKNQLMVESIAQLQKKEKELKELKKQLTKKAGEREDFQTQNLSHDLASLATPPFESPHELRRTISPPPPPLSSGDTSQRDGVGEVAAGTLIRRTNATLPHWMPQLTGE*

>AT3G54340.1 | Symbols: AP3, ATAP3 | APETALA 3 | chr3:20119428-20121087 REVERSE LENGTH=699

MARGKIQIKRIENQTNRQVTYSKRRNGLFKKAHELTVLCDARVSIIMFSSSNKLHEYISPNTTTKEIVDLYQTISDVDVWATQYERMQETKRKLLETNRNLRTQIKQRLGECLDELDIQELRRLEDEMENTFKLVRERKFKSLGNQIETTKKKNKSQQDIQKNLIHELELRAEDPHYGLVDNGGDYDSVLGYQIEGSRAYALRFHQNHHHYYPNHGLHAPSASDIITFHLLE*

>AT3G57230.1 | Symbols: AGL16 | AGAMOUS-like 16 | chr3:21177710-21180671 FORWARD LENGTH=723

MGRGKIAIKRINNSTSRQVTFSKRRNGLLKKAKELAILCDAEVGVIIFSSTGRLYDFSSSSMKSVIERYSDAKGETSSENDPASEIQFWQKEAAILKRQLHNLQENHRQMMGEELSGLSVEALQNLENQLELSLRGVRMKKDQMLIEEIQVLNREGNLVHQENLDLHKKVNLMHQQNMELHEKVSEVEGVKIANKNSLLTNGLDMRDTSNEHVHLQLSQPQHDHETHSKAIQLNYFSFIA*

>AT3G57390.1 | Symbols: AGL18 | AGAMOUS-like 18 | chr3:21233910-21235735 FORWARD LENGTH=771

MGRGRIEIKKIENINSRQVTFSKRRNGLIKKAKELSILCDAEVALIIFSSTGKIYDFSSVCMEQILSRYGYTTASTEHKQQREHQLLICASHGNEAVLRNDDSMKGELERLQLAIERLKGKELEGMSFPDLISLENQLNESLHSVKDQKTQILLNQIERSRIQEKKALEENQILRKQVEMLGRGSGPKVLNERPQDSSPEADPESSSSEEDENDNEEHHSDTSLQLGLSSTGYCTKRKKPKIELVCDNSGSQVASD*

>AT3G58780.1 | Symbols: SHP1, AGL1 | SHATTERPROOF 1, AGAMOUS-like 1 | chr3:21739150-21741766 FORWARD LENGTH=747

MEEGGSSHDAESSKKLGRGKIEIKRIENTTNRQVTFCKRRNGLLKKAYELSVLCDAEVALVIFSTRGRLYEYANNSVRGTIERYKKACSDAVNPPSVTEANTQYYQQEASKLRRQIRDIQNSNRHIVGESLGSLNFKELKNLEGRLEKGISRVRSKKNELLVAEIEYMQKREMELQHNNMYLRAKIAEGARLNPDQQESSVIQGTTVYESGVSSHDQSQHYNRNYIPVNLLEPNQQFSGQDQPPLQLV*

>AT3G61120.1 | Symbols: AGL13 | AGAMOUS-like 13 | chr3:22618414-22620466 REVERSE LENGTH=735

MGRGKVEVKRIENKITRQVTFSKRKSGLLKKAYELSVLCDAEVSLIIFSTGGKLYEFSNVGVGRTIERYYRCKDNLLDNDTLEDTQGLRQEVTKLKCKYESLLRTHRNLVGEDLEGMSIKELQTLERQLEGALSATRKQKTQVMMEQMEELRRKERELGDINNKLKLETEDHDFKGFQDLLLNPVLTAGCSTDFSLQSTHQNYISDCNLGYFLQIGFQQHYEQGEGSSVTKSNARSDAETNFVQ*

>AT3G66656.1 | Symbols: AGL91 | AGAMOUS-like 91 | chr3:2091262-2091798 REVERSE LENGTH=537

MGRRKIKMEKVQDTNTKQVTFSKRRLGLFKKASELATLCNAEVGIVVFSPGNKPYSFGKPNFDVIAERFKNEFEEEEEGDSCETSGYSRGNRARQEKKICKRLNSITEEAEAEKKHGEDLHKWLESAEQDKFNKPIEELTLEELKEFEAKIKKISCGIQSNISHMQASSSLMFLSNDN*

>AT4G02235.1 | Symbols: AGL51 | AGAMOUS-like 51 | chr4:980955-981711 FORWARD LENGTH=591

MKQSSFSSSSSSRNSTSLTNRLKTIFKKAEELSILCAIDVCVIYYGPDGELRTWPKERNTVKDMASRYKEATKRKKKRTLSTLQERLRIVESQKQQNKNLVHQSLTPSYLNQIQHLNPSNFSPYMYNHGDAATLSQLPLSASLSNQLQLPESLDAAWFWSEHVFGQHHQQQQLSTSWRVKHTRILTVSFGTSICSE*

>AT4G09960.1 | Symbols: STK, AGL11 | AGAMOUS-like 11, SEEDSTICK | chr4:6236713-6239409 REVERSE LENGTH=693

MGRGKIEIKRIENSTNRQVTFCKRRNGLLKKAYELSVLCDAEVALIVFSTRGRLYEYANNNIRSTIERYKKACSDSTNTSTVQEINAAYYQQESAKLRQQIQTIQNSNRNLMGDSLSSLSVKELKQVENRLEKAISRIRSKKHELLLVEIENAQKREIELDNENIYLRTKVAEVERYQQHHHQMVSGSEINAIEALASRNYFAHSIMTAGSGSGNGGSYSDPDKKILHLG*

>AT4G11250.1 | Symbols: AGL52 | AGAMOUS-like 52 | chr4:6849578-6850567 FORWARD LENGTH=990

MKQASSSSSCNPTSLTNRLKTIFKKAEELSILCAIDVCVIYYGPDGDLRTWPKDRETVKNMALRYKEDRKRKKCLNLHEFLEKEKVKDKDKYKGKTNYVKNPNWYPNFDHYSPQQLSQLIQSLERTLSTLQKRLRIVESQKKQNTNLVHQSLTPSYLNQTQHLDPSKFSLYMYNHGDATLSQLPLSASQSNQLINYQMQHGFGQNMCLDNITNNNNFQHPGVSNTQDYSPLLSANNYGLNNHLMQQQDQLHGFDQNLCMMSEIINNNNGLQHPNLSNTVPHEFPYGNTSFSQDMFSSYDGSSLLQTSSLPPLHNIPNSYCFSDNSRLLC*

>AT4G11880.1 | Symbols: AGL14, XAL2 | XAANTAL2, AGAMOUS-like 14 | chr4:7143512-7147108 FORWARD LENGTH=666

MVRGKTEMKRIENATSRQVTFSKRRNGLLKKAFELSVLCDAEVALIIFSPRGKLYEFSSSSSIPKTVERYQKRIQDLGSNHKRNDNSQQSKDETYGLARKIEHLEISTRKMMGEGLDASSIEELQQLENQLDRSLMKIRAKKYQLLREETEKLKEKERNLIAENKMLMEKCEMQGRGIIGRISSSSSTSELDIDDNEMEVVTDLFIGPPETRHFKKFPPSN*

>AT4G18960.1 | Symbols: AG | AGAMOUS | chr4:10383917-10388272 FORWARD LENGTH=759

TAYQSELGGDSSPLRKSGRGKIEIKRIENTTNRQVTFCKRRNGLLKKAYELSVLCDAEVALIVFSSRGRLYEYSNNSVKGTIERYKKAISDNSNTGSVAEINAQYYQQESAKLRQQIISIQNSNRQLMGETIGSMSPKELRNLEGRLERSITRIRSKKNELLFSEIDYMQKREVDLHNDNQILRAKIAENERNNPSISLMPGGSNYEQLMPPPQTQSQPFDSRNYFQVAALQPNNHHYSSAGRQDQTALQLV*

>AT4G22950.1 | Symbols: GL19, AGL19 | AGAMOUS-like 19 | chr4:12023946-12027421 REVERSE LENGTH=660

MVRGKTEMKRIENATSRQVTFSKRRNGLLKKAFELSVLCDAEVALVIFSPRSKLYEFSSSSIAATIERYQRRIKEIGNNHKRNDNSQQARDETSGLTKKIEQLEISKRKLLGEGIDACSIEELQQLENQLDRSLSRIRAKKYQLLREEIEKLKAEERNLVKENKDLKEKWLGMGTATIASSQSTLSSSEVNIDDNMEVETGLFIGPPETRQSKKFPPQN*

>AT4G24540.1 | Symbols: AGL24 | AGAMOUS-like 24 | chr4:12671160-12673645 REVERSE LENGTH=663

MAREKIRIKKIDNITARQVTFSKRRRGIFKKADELSVLCDADVALIIFSATGKLFEFSSSRMRDILGRYSLHASNINKLMDPPSTHLRLENCNLSRLSKEVEDKTKQLRKLRGEDLDGLNLEELQRLEKLLESGLSRVSEKKGECVMSQIFSLEKRGSELVDENKRLRDKLETLERAKLTTLKEALETESVTTNVSSYDSGTPLEDDSDTSLKLGLPSWE*

>AT4G36590.1 | Symbols: AGL40 | agamous-like 40 | chr4:17261146-17262189 REVERSE LENGTH=747

MVRSTKGRQKIEMKKMENESNLQVTFSKRRFGLFKKASELCTLSGAEILLIVFSPGGKVFSFGHPSVQELIHRFSNPNHNSAIVHHQNNNLQLVETRPDRNIQYLNNILTEVLANQEKEKQKRMVLDLLKESREQVGNWYEKDVKDLDMNETNQLISALQDVKKKLVREMSQYSQVNVSQNYFGQSSGVIGGGNVGIDLFDQRRNAFNYNPNMVFPNHTPPMFGYNNDGVLVPISNMNYMSSYNFNQS*

>AT4G37940.1 | Symbols: AGL21 | AGAMOUS-like 21 | chr4:17835695-17838621 REVERSE LENGTH=687

MGRGKIVIQRIDDSTSRQVTFSKRRKGLIKKAKELAILCDAEVGLIIFSSTGKLYDFASSSMKSVIDRYNKSKIEQQQLLNPASEVKFWQREAAVLRQELHALQENHRQMMGEQLNGLSVNELNSLENQIEISLRGIRMRKEQLLTQEIQELSQKRNLIHQENLDLSRKVQRIHQENVELYKKAYMANTNGFTHREVAVADDESHTQIRLQLSQPEHSDYDTPPRANE*

>AT5G04640.1 | Symbols: AGL99 | AGAMOUS-like 99 | chr5:1332825-1333793 FORWARD LENGTH=969

MGGVKRKISIELIEKKDSRAVAFSKRSRGLYSKASDLCLLSDAQIAIIATPVSSKSNVSFYTFGHSSVDNVVAAFLTNQRPREGLGLDYWWEDERLSKSEDLEELRDAMDSMSKMLKDLKDLQNQRDCEEDVKKKGVLHGTHQKQTFNPESCSVNFDGFNKNTEEFDLDEIFDYVSTAEALSMNLDMDDVSVVTTNQNPVSASETVEDRELVVHKNMDEDNIHVSDMDDKDTMLMISDKNNVLPENLDEFDQELDLDQLLDFETNYESLLKSCEMEDYASMVTTKQNLCSNPEAVEDGGLMIQKDLPEDNLCFSDYFSDLHC*

>AT5G06500.1 | Symbols: AGL96 | AGAMOUS-like 96 | chr5:1982444-1983172 FORWARD LENGTH=729

MARKKVRAAWIRDDRMRRASLKRRLTGLIKKVNELSILCDMRASVVVFNREEEQLTAWPSPEAANSLIDNFYSLTDHERTMKAVDPESYVQTVIEKIEKKRADTRKVITEFEMDELMFQVQNGRELADLSPTEADKLIPYADKKLMWLSKRMGSTGVDALRASNVASGSGGNGLNMMETGRSFYYVDKWVFVDPQVQNPCDVETHLPTMVSGLDLNMEPSDEDLGTYKGESSMAGGAEDDAE*

>AT5G10140.1 | Symbols: FLC, RSB6, AGL25, FLF | AGAMOUS-like 25, FLOWERING LOCUS F, REDUCED STEM BRANCHING 6, FLOWERING LOCUS C | chr5:3173724-3179339 REVERSE LENGTH=591

MGRKKLEIKRIENKSSRQVTFSKRRNGLIEKARQLSVLCDASVALLVVSASGKLYSFSSGDNLVKILDRYGKQHADDLKALDHQSKALNYGSHYELLELVDSKLVGSNVKNVSIDALVQLEEHLETALSVTRAKKTELMLKLVENLKEKEKMLKEENQVLASQMENNHHVGAEAEMEMSPAGQISDNLPVTLPLLN*

>AT5G13790.1 | Symbols: AGL15 | AGAMOUS-like 15 | chr5:4449128-4450802 REVERSE LENGTH=807

MGRGKIEIKRIENANSRQVTFSKRRSGLLKKARELSVLCDAEVAVIVFSKSGKLFEYSSTGMKQTLSRYGNHQSSSASKAEEDCAEVDILKDQLSKLQEKHLQLQGKGLNPLTFKELQSLEQQLYHALITVRERKERLLTNQLEESRLKEQRAELENETLRRQVQELRSFLPSFTHYVPSYIKCFAIDPKNALINHDSKCSLQNTDSDTTLQLGLPGEAHDRRTNEGERESPSSDSVTTNTSSETAERGDQSSLANSPPEAKRQRFSV*

>AT5G15800.1 | Symbols: AGL2, SEP1 | SEPALLATA1, AGAMOUS-like 2 | chr5:5151594-5153767 REVERSE LENGTH=756

MGRGRVELKRIENKINRQVTFAKRRNGLLKKAYELSVLCDAEVALIIFSNRGKLYEFCSSSNMLKTLDRYQKCSYGSIEVNNKPAKELENSYREYLKLKGRYENLQRQQRNLLGEDLGPLNSKELEQLERQLDGSLKQVRSIKTQYMLDQLSDLQNKEQMLLETNRALAMKLDDMIGVRSHHMGGGGGWEGGEQNVTYAHHQAQSQGLYQPLECNPTLQMGYDNPVCSEQITATTQAQAQQGNGYIPGWML*

>AT5G20240.1 | Symbols: PI | PISTILLATA | chr5:6829203-6831208 FORWARD LENGTH=627

MGRGKIEIKRIENANNRVVTFSKRRNGLVKKAKEITVLCDAKVALIIFASNGKMIDYCCPSMDLGAMLDQYQKLSGKKLWDAKHENLSNEIDRIKKENDSLQLELRHLKGEDIQSLNLKNLMAVEHAIEHGLDKVRDHQMEILISKRRNEKMMAEEQRQLTFQLQQQEMAIASNARGMMMRDHDGQFGYRVQPIQPNLQEKIMSLVID*

>AT5G23260.1 | Symbols: TT16, ABS, AGL32 | TRANSPARENT TESTA16, ARABIDOPSIS BSISTER, AGAMOUS-like 32 | chr5:7836294-7838340 FORWARD LENGTH=792

MNIEEEGATHKRKKREMGRGKIEIKKIENQTARQVTFSKRRTGLIKKTRELSILCDAHIGLIVFSATGKLSEFCSEQNRMPQLIDRYLHTNGLRLPDHHDDQEQLHHEMELLRRETCNLELRLRPFHGHDLASIPPNELDGLERQLEHSVLKVRERKQQLENLSRKRRMLEEDNNNMYRWLHEHRAAMEFQQAGIDTKPGEYQQFIEQLQCYKPGEYQQFLEQQQQQPNSVLQLATLPSEIDPTYNLQLAQPNLQNDPTAQND*

>AT5G26580.1 | Symbols: AGL34 | AGAMOUS-like-34 | chr5:9393065-9394102 REVERSE LENGTH=1038

MGMKKVKLSLIANEISRETSFMKRKNGIMKKLYELSTLCGVQACTLIYSPFIPVPEFLEMSPTARTRKMMNQETYLMERITKAKEQLQNLVGANQELQVRRFMFDCVEGKMSQYRYDAKDLQDLLSCINLYLDQLNGRIEILKEHGDSLPSVSPFPTRIGVEETGDESSSDSPILATTGVVDTPNATNPRVLVADTTHFLDANATAVTAPFGFSNHIQYKNMNMSQDLHRPFQHLVPTNFCDFFQNQNMNQVQYQAPPNDMFNQIQREFYNINLNQKSNQYMNQQQPFMNPMVEQHMSHVGGRESIPFMDGNYYNYNQLPVVDHGSTSYMPSTTGVYDPYFNNNL*

>AT5G26630.1 | Symbols: AGL35 | agamous-like 35 | chr5:9350815-9351471 FORWARD LENGTH=657

MTRQKVKMTFIENETARKSTFKKRKKGLLKKAQELGILCGVPIFAVVNSPYELNPEVWPSREAANQVVSQWKTMSVMDKTKKMVNQETFLQQRITKATESWKKLRKENKELEMKNIMFDCLSGKTLVSSIEKTELRDFGYVIEQQLKDVNRRIEILKRNNEPSSALVPVAAPTTSSVMPVVEMGSSSVGFYDKVRDQIQITLNMKQTTNDLDLNKKQW*

>AT5G26650.1 | Symbols: AGL36 | AGAMOUS-like 36 | chr5:9343785-9344885 FORWARD LENGTH=1101

MKKVKLSLIANERSRKTSFIKRKDGIFKKLHELSTLCGVQACALIYSPFIPVPESWPSREGAKKVASRFLEMPPTARTKKMMDQETYLMERITKAKEQLKNLAAENRELQVRRFMFDCVEGKMSQYHYDAKDLQDLQSCINLYLDQLNGRIESIKENGESLLSSVSPFPTRIGVDEIGDESFSDSPIHATTGVVDTLNATNPHVLTGDMTPFLDADATAVTASSRFFDHIPYENMNMSQNLHEPFQHLVPTNVCDFFQNQNMNQVQYQAPNNLFNQIQREFYNINLNLNLNLNSNQYLNQQQSFMNPMVEQHMNHVGGRESIPFVDGNCYNYHQLPSNQLPAVDHASTSYMPSTTGVYDPYINNNL*

>AT5G26880.1 | Symbols: AGL26 | AGAMOUS-like 26 | chr5:9457950-9459190 REVERSE LENGTH=792

MESCCRSVIASRTFHLRSSGRLFPSLSLTHLKGKLSLSINSFSSKIQSHALRGVGIGESDKKNPLPRGAGEGVKEDARSKLLHVVLVSPQIPGNTGCIARTCAASAVGLHLVGPLGFQVDDARVKRAGLDYWPFVVVKAHSSWAEFQEYFRLQEGEKRMIAFTKRGTRIHSDFSYRSGDYLLFGSETSGLPPEALSDCNHEPYGGGTLRIPMVETYVRCLNLSVSVGIALYEASRQLNYEQIECAPQGCVNGEEPLLTEDIFA*

>AT5G26950.1 | Symbols: AGL93 | AGAMOUS-like 93 | chr5:9483251-9484120 REVERSE LENGTH=870

MDSSMSTKKKTKLSVRNQTCFKKSSLSSSSTAKKTTNLSMREQTMFKKALELSTLCNIDVCVIYYGRDGKLIKTWPDDQSKVRDMAERFSRLHERERCKKRTNLSLFLRKKILDDTKLSEKVLEMEDSLESGLRVLQDKLLLLQPEKNQTEFGQTRAVSSTTNPLSPPPSLIEDHRHQQRTEPLMSGVSNTEQDLSTSSLSQNQSKFSVFLYNHDNCSFYQVPDSVSSFDSLTSTGLLGEQGSGLGSSFDLPMVFPPQMQTQTPLVPFDQFAPWNQAPSFADPMMFPYN*

>AT5G27050.1 | Symbols: AGL101 | AGAMOUS-like 101 | chr5:9520276-9520638 FORWARD LENGTH=363

MFKKALELSTLCNIEVCVIYYGRDGELFKTWPEDESKVRDMAERFTKLNERERRKKRTNLSLFLRKKILDDNKLSGKVLEMKDSLERGLRVLQDKLLLLQPENQTKSLTRSVSSLDYVFV*

>AT5G27070.1 | Symbols: AGL53 | AGAMOUS-like 53 | chr5:9527741-9528604 FORWARD LENGTH=864

MDSSMSTKKKTKLSVRNQTCFKKSSLSSSSTAKKTTNLSMREQTMFKKALELSTLCNIDVCVIYYGRDGKLIKTWPEDQSKVRDMAERFSRLHERERCKKRTNLSLFLRKKILDDTKLSEKVLEMEDSLESGLRVLQDKLLLLQPEKNQTEFGQTRAVSSTTNPLSPPPSLIEDHRHQQWTEPLMSGVSNTEQDLSTSSLSQNQSRISVFLYNHDNRSFYQVPDSVSSFDQSALLGEQGSGLGSNFDLPPMVFPPQMQTQTPLVPFDQFAAWNQAPSFADPMMFPYN*

>AT5G27090.1 | Symbols: AGL54 | AGAMOUS-like 54 | chr5:9531845-9532408 FORWARD LENGTH=564

MDSSTSTKKNTKLFVRNQTCFKKSSLSSSNAKKTTNLSMREQTMFKKALELSTLCDIEVCVIYYGRDGKLIKTWPEDQSKVRDMAERFSRLHERERCKKRTNLSLFLRKQILHDKKLSEKVLEMEDSLESGLRVLQDKLLLLQPEKNQTELGQSCAVYSTTYPLSSPSLIEDHQHQQQWTEPLSNTE*

>AT5G27130.1 | Symbols: AGL39 | AGAMOUS-like 39 | chr5:9546633-9547553 FORWARD LENGTH=921

MPSSDSTMMKKGTKRKIEIKKRETKEQRAVTCSKRRQTVFSKAADLCLISGANIAVFVTSPSDSSDVVYSFSGYSSAYEIADCYLNRKPPPKIVNPAGSKLGFWWEDPDLYHSCDDLSELSIIEDRLQRMKKHVMACLEKEEKSQLVSSFDQNPNSTCSLDVEDCDGSSYSQIASTFTPNSVNEYCSDQTFSSFHGDQNPNLSSPSFDQDCYSSLYQICGESSSQVASFDQNPSSEIQGFETEEEINQINLLLQETQTEANVNLDDEICFWNDLSNDDVFGLNSYFGLDNTNAMINFGDSDFRRHV*

>AT5G27580.1 | Symbols: AGL89 | AGAMOUS-like 89 | chr5:9736651-9737322 FORWARD LENGTH=672

MDSSMSTKKKTKLSVRNQTCFKKSSLSSSSTAKKTTNLSMREETMFKKALELSTLCDIEVCVIYYGRDGELIKTWPEDQSKVRDMAERFSKLHERERRKKRTNLSLFLRKKILDDNKLSEKVLEMKDSLESGLRVLQDKLLLLQPENQTELGQSRAVSSTTNPLSSPEDHHHQQWTEPLVTGVSNTEQDLSTSPLSNHQSKYSVFVYNHDSGSFYQVPDSICF*

>AT5G27810.1 | Symbols: no symbol available | no full name available | chr5:9855827-9856186 FORWARD LENGTH=360

MKKVHELSTLCGITSCAIIYSPYDTSHEVWPSNSGVQRVVSEFRTLPEMDQHKKMVDQEGFLKQRIAKPTENLRRQRKDNKELEMTEVMFRCLIGNMEMLKSESQSESTTMVYENDEPS*

>AT5G27944.1 | Symbols: no symbol available | no full name available | chr5:9975918-9976592 REVERSE LENGTH=675

MYSSMSTKKKTKLSVRNQTCFKKSSLSSSSTAKKTTNLSMREQTMFKKALELSTLCDIEVCVILYGRDGELIKTWPEDQSKVRDMAERFSRLHERERCKKRTNLSLFLRKKILDDNKLSEKVLEMEDSLESGLRVLQNKLLLLQPEKNQTKLGQSRAVSSTTNLLSSPENHHNQQWTEPLENGVSNTEQELSTSSLSQHQSKYSVFLYNHDNGSFYQVPDSICF*

>AT5G27960.1 | Symbols: AGL90 | AGAMOUS-like 90 | chr5:9991685-9992770 REVERSE LENGTH=963

MKKVKLSLIANERSRKTSFMKRKNGIFKKLHELSTLCGVQACALIYSPFIPVPESWPSREGAKKVASKFLEMPRTARTRKMMDQETHLMERITKAKEQLKNLAAENRELQVRRFMFDCVEGKMSQYRYDAKDLQDLLSCMNLYLDQLNGRIESIKENGESLLSSVSPFPTRIGVDEIGDESFSDSPIHSTTRVVDTPNATNPHVLAGDMTPFLDADANANMNQVQYQAPNNLFNQIQREFYNINLNLNLNLNSNQYLNQQQSFMNPMVEQHMNHVGGRESIPFVDRNYYNYNQLPAVDLASTSYMPSTTDVYDPYINNNL*

>AT5G37415.1 | Symbols: AGL105 | AGAMOUS-like 105 | chr5:14839047-14840301 REVERSE LENGTH=951

MRDPDIVQNTRSLILLRVLTHEKLIQSHRYFFVGWIHKNLSRFRRKIRRRFLHCDFSGFGFFPRRFLFGFVLEKMDHLQESFRGSELCILCDIEACVIYYGPDGELKTWPKEREKVEDIALRYSQLNEALRRKKSVTLYDFLNKKKDKTNLEKKAMITDNDDLKTCLKNVNVLKSPIADHYFNDQISQLIQSLEPHVSKVQERIRFVESQKHKETKLDHQSLASIYSLNQSLNPSQFTLFLYNHGDNTMSQIPNMFMNNNNFQHSFVSNTQDYSALQESVNNNYGLMPNVLCGYDQNLFTSDITNNNLLIDNSMYL*

>AT5G38620.1 | Symbols: AGL73 | agamous-like 73 | chr5:15463858-15464907 REVERSE LENGTH=1050

MVKGTKRKIAIETIQKRDSLRVTCTKRRKGLYSKASQLCLLSDAQIAILATPPSSESDVSFYSFGHSSVDAVVSAFLSGKRPVSAPKDNKETREDVGICLTRKNLGLGFWWNDESLVRSENPQEISEAIGSMWTLLSNLKELRADEACVNDHKDLKKNEKSDVHGTQDQTLIFQSASAVCCIPENLNDITQEPNQTLDIQSSSSAICCVPDKSPEIFNEITEEQDQILSICETFCVTDNNNNNNNNAALPEVNLYYNQDMAIDQLIDFNTPFESSIDDWFSDNTTHQETTSASILNDVGVDDQVSVDTNPFSYFQSLEDADLVFQRCLDGDNLRFSDCFNDFANTIAAV*

>AT5G38740.1 | Symbols: AGL77 | AGAMOUS-like 77 | chr5:15513025-15514305 REVERSE LENGTH=1281

MTTIRSSPSSSRCSNSSSSSSYSLASTSLSNRLETIFKKASELCTLCDIEACVIYYGPDGELKTWPKEREKVRDIALRFNQLNEALRHKKSVNLHGFLNKKKKNKGLKNPNKKKKTSLKNVNVLKYPLADHYSPDQVSQLTQSLELNVSKFQERLRFLESQKQNETKPDHQSLTSISSLNQSLNPSQFSLFMYNHGYNTLSQIPVSASNFNQDYISALLEQSELKSQIMKQEVCGYEQNMCMSNHGDATLSQIPFSASNFNQDFSANNNFQHSFVSNTQDYYSVQKSVNNNYGLKNQLMKHDLCGYEHNMCMSNHGDATFSQIPLSASNFNQDFSVSIQEESGLMQQELCGYDQNQNMSMGDITNNNFQVTCASVLESVNNFGLNQLMHKEFYGCHQNMSMGNINNNSFQHPWVSNADHTRRYKNL*

>AT5G39750.1 | Symbols: EMB3008, AGL81 | EMBRYO DEFECTIVE 3008, AGAMOUS-like 81 | chr5:15906875-15907942 FORWARD LENGTH=1068

MAIRSLPSSSRCSSSSSSSSYSLASTSLSNRLETIFKKASELCTLCDIEACVIYYGPDGELKTWPPEREKVEDIALRYSQLNEALRRKKSVTLYDFLNKKKDKTNLEKKAKITDNDDLKTCLKNVNILKYPLADHYSPDQVSQLIQSLEPHVSKVRERIRFVESQKHKETKPDHQSLASSSLNHQTQSLNPSQFSLFMYNHGDNTLSQIPVSASNFNQDYFSALLEQSELKSQIMKQDLCGYEQNMCMSNHGDATLSQIPLSASNLNQDFSALLQDESGLMQQELCGYDQNMFMNNNNFQHSFVSNTQDHSAPVVQESVNNNYGLMPHVPCGYDQNLFTSDITNNNLLINNSMFL*

>AT5G39810.1 | Symbols: AGL98 | AGAMOUS-like 98 | chr5:15937278-15938344 REVERSE LENGTH=990

MAIRSLPSSSGCSNSSSSSSYSLASTSLSNRLETIFRKASELCTLCDIEACVIYYGPDGELKTWPPEREKVEDIALRYSQLNEALRRKKSVTLYDFLNKKKNKTNLEKKAKIKDNDLKRLSLEPHVSKVRERIRFVESQKHKETKPDHQSLASSSLNNQTQSLNPSQFSLFMYNHGDNILSQIPVSASNFNQDYFSALLEQSELKSQIMKQEVCGYEQNMCMSNNGDATLSQIPLSASNFNQEFSALLQEESGLMQQELCNYDQNMFMNNNNFQHSFVSNTQDHSAPAVQESVNNNYGLMPHVPCGYDQNLFTSDITNNNLLIDNSMFL*

>AT5G40120.1 | Symbols: AGL76 | AGAMOUS-like 76 | chr5:16051879-16053036 FORWARD LENGTH=1158

MTMRSLPFSSSSYSLASTSLSNRLETIFKKASELCTLCDIEACVIYYGPDGELKTWPKEKEKVRDIALRYSQLNEALRSKKSVNLHGFLNKKKKKKKKGLKNPNNKRKTCLKKNVNVLKYPLADHYPPDQVSQLTQSLKLHVSKFQERLRFLESQKQTKPDHQSLTPSSLNHQTQSLNPRQFSLFMYNHGDNTLSQISVSASNFNQNYFSALLEQSELKNQLMKQDGYDQNQNMRMGDITNNNFQLPYFSKKEAVQESVNYFGMNQLMLKELYGCDQNMCMGNINSNSFQHPCVSKAQHYSAVEGSVNNQRQSELMQQELCGYEQNMCFTNNNFQVSNKEAVQESVTNFGLMQHELYGCDQNMSMGNIINNSFQQRLKHRTRICE*

>AT5G40220.1 | Symbols: AGL43 | AGAMOUS-like 43 | chr5:16078390-16079364 REVERSE LENGTH=975

MTMRSSLPSSSSAYSLASTSLSNRLETIFKKASELCTLCDIEACVIYYGPDGELKTWPPEREKVRDIALRYSQLNEALRRKKSVNLHGFLNKKKKNKGLKNTDKKRKTSLKKVNVLKYPLADHYPPDQVSPLIQSLELHVSKFHERLEFLESRKQNETQPDHHSLASSSLNHQTQSLNPSQFSLFMYNHGDNTLSQIPVSASNFNQDYFSALLEQSELKNQLMKQEICGNDQNQNMWMGNITNNNFQLPCVSVQESVNNFGLMHKEFYGCDHNMSVGNINSNSCEHPCVSSTQHYSAVEESVNNPWLNQLMQNELYGYGYADFC*

>AT5G41200.1 | Symbols: AGL75 | AGAMOUS-like 75 | chr5:16490544-16491536 FORWARD LENGTH=993

MTMRSSSPSSSSSYSLAFTSLSNRLETIFKKASELCTLCDIEACVIYYGPDGELKTWPKEKEKVRDIALRYSLLNEALRRKKSVNLHGFLNKKKNKGLKNPNKKMKTSLKNVNILKYPLADHYPPDQVSPLIQSLELHVSKFQERLRFLESQKQNQTKPDHQSLTPSSLNHYTQSLNPSQFSLFMYNHGDNTLSQIPVSASNFNQDYFSALLEESELKNQLMKPEICGYDQNQNMSMGDITNNKFQDPCVSNKEAVQESVNNFGLNQLMYKEFYGCDQNMSMGNINSNSFQNPCVSNTQHYSAVEESVKNPWLNQLMQNELYGYGYAGFC*

>AT5G48670.1 | Symbols: FEM111, AGL80 | AGAMOUS-like 80 | chr5:19738825-19739790 REVERSE LENGTH=966

MTRKKVKLAYISNDSSRKATFKKRKKGLMKKVHELSTLCGITACAIIYSPYDTNPEVWPSNSGVQRVVSEFRTLPEMDQHKKMVDQEGFLKQRIAKATETLRRQRKDSRELEMTEVMFQCLIGNMEMFHLNIVDLNDLGYMIEQYLKDVNRRIEILRNSGTEIGESSSVAVAASEGNIPMPNLVATTAPTTTIYEVGSSSSFAAVANFVNPIDLQQFRHPAAQHVGLNEQPQNLNLNLNQNYNQNQEWFMEMMNHPEQMRYQTEQMGYQFMDDNHHNHIHHQPQEHQHQIHDESSNALDAANSSSIIPVTSSSITNKTWFH*

>AT5G49420.1 | Symbols: AGL84 | agamous-like 84 | chr5:20035166-20036170 REVERSE LENGTH=1005

MVKKGGTKRKIAIETIQKRDSLRVTCTKRREGLYSKASQLCLLSDAQIAILATPPSSESNVSFYSFGHSSVDAVVSAFLSGQRPVPKDNKETREDVGICLTRNNLGLGFWWNDESLARSENPQEISEAIDSMRTLLRNLKELRADEALACNQAFVNDREDLKNNDKCDFVSDHETHDQTLILQSASPICCIPENLNEITQEPNQTLNIQSSTSAICCVPDNSPENFNEITEEQDQIRSICETFCVMDNNAALPEMNLDYDQDIGFDTPFESALNDWFSDNTTHQEISASILNAVVDDQVSVDLTPFSYFQRCLDGDNLRFSDCFKDFANTISAL*

>AT5G49490.1 | Symbols: AGL83 | AGAMOUS-like 83 | chr5:20075328-20076185 FORWARD LENGTH=858

MRFVPYLYEIERLWLSLVNYLSPRKNKNRRCGEIDKIRMVKKGGTKRKIAIETIQKSDYLRVTCTKRREGLFSKASQLCLLSDAQIAILATPPTSESNISFYSFGHSSVDAVVSSFLSGQRCVPLQEDTKEMREDVAICLSRTNLGLGFWWNNESLNKSENPQEISDAINSMLTLLSNLKELSGEEALVNDHKDLKKNERSDVVLQHGTQYETLNPNSNTTTICCVPDELPANSNEIVGISPNPLIMLEKKKSQIEEKFEKEWQVSVTRIENEATSSYAKRRRSI*

>AT5G51860.1 | Symbols: AGL72 | AGAMOUS-like 72 | chr5:21081844-21084126 REVERSE LENGTH=636

MVRGKIEIKKIENVTSRQVTFSKRRSGLFKKAHELSVLCDAQVAAMIFSQKGRLYEFASSDIRNTIKRYAEYKREYFVAETHPIEQYVQGLKKEMVTMVKKIEVLEVHNRKMMGQSLDSCSVKELSEIATQIEKSLHMVRLRKAKLYEDELQKLKAKERELKDERVRLSLKKTIYTHLCQVGERPMGMPSGSKEKEDVETDLFIGFLKNRP*

>AT5G51870.1 | Symbols: AGL71 | AGAMOUS-like 71 | chr5:21085635-21087923 REVERSE LENGTH=624

MVRGKIEIKKIENVTSRQVTFSKRRSGLFKKAHELSVLCDAQVAAIVFSQSGRLHEYSSSQMEKIIDRYGKFSNAFYVAERPQVERYLQELKMEIDRMVKKIDLLEVHHRKLLGQGLDSCSVTELQEIDTQIEKSLRIVRSRKAELYADQLKKLKEKERELLNERKRLLEEVNMHHSSKGNTEGGHRTKHSSEVETDLFIGLPVTRL*

>AT5G55690.1 | Symbols: AGL47 | agamous-like 47 | chr5:22548790-22549623 REVERSE LENGTH=834

MGRKMVKMTRITNEKTRITTYKKRKACLYKKASEFSTLCGVDTCVIVYGPSRAGDEMVMEPELWPKDGSKVREILTKYRDTASSSCTKTYTVQECLEKNNTKVEKPTIATKYPTWDKKLDQCSLNDLYAVFMAVENKIQEATNRNQTFPDTSCWSNDQLGLCGYNRQCFEQYQLFPLPTMDYNGLSFFPFNNQMTSNTAEVSSFSNVTEPMIANGQSLFYGSCSDGPYGPMVQRTAYMEPIHWGLGNSMFNNVKQFQDYPFRFAQVNDLEDSSKLSM*

>AT5G58890.1 | Symbols: AGL82 | AGAMOUS-like 82 | chr5:23780832-23781716 FORWARD LENGTH=885

MVPKVVDLQRIANDKTRITTYKKRKASLYKKAQEFSTLCGVETCLIVYGPTKATDVVISEPEIWPKDETKVRAIIRKYKDTVSTSCRKETNVETFVNDVGKGNEVVTKKRVKRENKYSSWEEKLDKCSREQLHGIFCAVDSKLNEAVTRQERSMFRVNHQAMDTPFPQNLMDQQFMPQYFHEQPQFQGFPNNFNNMGFSLISPHDGQIQMDPNLMEKWTDLALTQSLMMSKGNDGTQFMQRQEQPYYNREQVVSRSAGFNVNPFMGYQVPFNIPNWRLSGNQVENWELSGKKTI*

>AT5G60440.1 | Symbols: AGL62 | AGAMOUS-like 62 | chr5:24306329-24307520 FORWARD LENGTH=900

MVKKSKGRQKIEMVKMKNESNLQVTFSKRRSGLFKKASELCTLCGAEVAIVVFSPGRKVFSFGHPNVDSVIDRFINNNPLPPHQHNNMQLRETRRNSIVQDLNNHLTQVLSQLETEKKKYDELKKIREKTKALGNWWEDPVEELALSQLEGFKGNLENLKKVVTVEASRFFQANVPNFYVGSSSNNAAFGIDDGSHINPDMDLFSQRRMMDINAFNYNQNQIHPNHALPPFGNNAYGINEGFVPEYNVNFRPEYNPNQNQIQNQNQVQIQIQNQSFKRENISEYEHHHGYPPQSRSDYY*

>AT5G60910.1 | Symbols: FUL, AGL8 | FRUITFULL, AGAMOUS-like 8 | chr5:24502736-24506013 REVERSE LENGTH=729

MGRGRVQLKRIENKINRQVTFSKRRSGLLKKAHEISVLCDAEVALIVFSSKGKLFEYSTDSCMERILERYDRYLYSDKQLVGRDVSQSENWVLEHAKLKARVEVLEKNKRNFMGEDLDSLSLKELQSLEHQLDAAIKSIRSRKNQAMFESISALQKKDKALQDHNNSLLKKIKEREKKTGQQEGQLVQCSNSSSVLLPQYCVTSSRDGFVERVGGENGGASSLTEPNSLLPAWMLRPTTTNE*

>AT5G62165.1 | Symbols: AGL42, FYF | AGAMOUS-like 42, FOREVER YOUNG FLOWER | chr5:24965075-24968437 FORWARD LENGTH=633

MVRGKIEMKKIENATSRQVTFSKRRNGLLKKAYELSVLCDAQLSLIIFSQRGRLYEFSSSDMQKTIERYRKYTKDHETSNHDSQIHLQQLKQEASHMITKIELLEFHKRKLLGQGIASCSLEELQEIDSQLQRSLGKVRERKAQLFKEQLEKLKAKEKQLLEENVKLHQKNVINPWRGSSTDQQQEKYKVIDLNLEVETDLFIGLPNRNC*

>AT5G65050.1 | Symbols: MAF2, AGL31 | AGAMOUS-like 31, MADS AFFECTING FLOWERING 2 | chr5:25982415-25986114 FORWARD LENGTH=549

MGRKKVEIKRIENKSSRQVTFSKRRNGLIEKARQLSILCESSIAVLVVSGSGKLYKSASGDNMSKIIDRYEIHHADELEALDLAEKTRNYLPLKELLEIVQSKLEESNVDNASVDTLISLEEQLETALSVTRARKTELMMGEVKSLQKTVGKKTFLVIEGDRGMSWENGSGNKVRETLPLLK*

>AT5G65060.1 | Symbols: AGL70, FCL3, MAF3 | AGAMOUS-like 70, MADS AFFECTING FLOWERING 3 | chr5:25987527-25991065 FORWARD LENGTH=591

MGRRKVEIKRIENKSSRQVTFSKRRKGLIEKARQLSILCESSIAVVAVSGSGKLYDSASGDNMSKIIDRYEIHHADELKALDLAEKIRNYLPHKELLEIVQSKLEESNVDNVSVDSLISMEEQLETALSVIRAKKTELMMEDMKSLQEREKLLIEENQILASQVGKKTFLVIEGDRGMSRENGSGNKVPETLSLLK*

>AT5G65070.1 | Symbols: MAF4, FCL4, AGL69 | AGAMOUS-like 69, MADS AFFECTING FLOWERING 4 | chr5:25992310-25995930 FORWARD LENGTH=603

MGRRKVEIKRIENKSSRQVTFCKRRNGLMEKARQLSILCESSVALIIISATGRLYSFSSGDSMAKILSRYELEQADDLKTLDLEEKTLNYLSHKELLETIQCKIEEAKSDNVSIDCLKSLEEQLKTALSVTRARKTELMMELVKTHQEKEKLLREENQSLTNQLIKMGKMKKSVEAEDARAMSPESSSDNKPPETLLLLK*

>AT5G65080.1 | Symbols: MAF5, AGL68 | AGAMOUS-like 68, MADS AFFECTING FLOWERING 5 | chr5:26000866-26002211 FORWARD LENGTH=354

DLEDKTQDYLSHKELLEIVQRKIEEAKGDNVSIESLISMEEQLKSALSVIRARKTELLMELVKNLQDKEKLLKEKNKVLASEVGKLKKILETGDERAVMSPENSSGHSPPETLPLLK*

>AT5G65330.1 | Symbols: AGL78 | AGAMOUS-like 78 | chr5:26110326-26111351 FORWARD LENGTH=1026

MKQASSSSSSRNSTSLTNRLKTIFKKAEELSILCAIEVCVIYYGPDGELRTWPKERETVKDMALRYKEARKRKKSRNLHEFLEKEKDKDKGKTNLKKNWYPNFDHYSPQQLSQLIQSLERTLSTLQERLRIVEAQKLQNTNLVHQSLTPSYLNQTQHLNPSKFSLFMYNHGDATLSQLPLSAPHSNQLINYQNHLMQHGFGQNMCSDNITNNNFEHPGVSNTQDYSPLLSVQASAVNNYGLNNHLMQQQDQLHGFDQNMCMVSEIINNNNGLQHPNLSNTVPHEFSSDFNQNPYGNAVGNISFSQDMFSSYDASSLLQTSSLPPLHNIPSSYCFPGNSRLL*

**#nucleotide sequences of MADS-box genes in *Oryza sativa***

>LOC_Os03g11614.1 Oryza sativa subsp. japonica|MIKC_MADS|MIKC_MADS family protein

ATGGGGAGGGGGAAGGTGGAGCTGAAGCGGATCGAGAACAAGATCAGCCGGCAGGTGACGTTCGCCAAGCGCAGGAACGGCCTGCTCAAGAAGGCCTACGAGCTCTCCCTCCTCTGCGACGCCGAGGTCGCCCTCATCATCTTCTCCGGCCGCGGCCGCCTCTTCGAGTTCTCCAGCTCATCATGCATGTACAAAACCTTGGAGAGGTACCGCAGCTGCAACTACAACTCACAGGATGCAGCAGCTCCAGAAAACGAAATTAATTACCAAGAATACCTGAAGCTGAAAACAAGAGTTGAATTTCTTCAAACCACACAGAGAAATATTCTTGGTGAGGATTTGGGCCCACTAAGCATGAAGGAGCTGGAGCAGCTTGAGAACCAGATAGAAGTATCCCTCAAACAAATCAGGTCAAGAAAGAACCAAGCACTGCTTGATCAGCTGTTTGATCTGAAGAGCAAGGAGCAACAGCTGCAAGATCTCAACAAAGACTTGAGGAAAAAGTTACAGGAAACCAGTGCAGAGAATGTGCTCCATATGTCCTGGCAAGATGGTGGTGGGCACAGCGGTTCTAGCACTGTTCTTGCTGATCAGCCTCATCACCATCAGGGTCTTCTCCACCCTCACCCAGATCAGGGTGACCATTCCCTGCAGATTGGGTATCATCACCCTCATGCTCACCATCACCAGGCCTACATGGACCATCTGAGCAATGAAGCAGCAGACATGGTTGCTCATCACCCCAATGAACACATCCCATCCGGCTGGATATGA

>LOC_Os12g10540.1 Oryza sativa subsp. japonica|MIKC_MADS|MIKC_MADS family protein

ATGGGGAGGGGCAGGATTGAGATCAAGAGGATCGAGAACACGACAAGCCGCCAGGTGACCTTCTGCAAGCGCCGCAACGGACTTCTCAAGAAGGCGTATGAGCTCTCCGTCCTCTGCGATGCCGAGGTGGCTCTCATCGTCTTCTCCAGCCGTGGCCGCCTCTACGAGTACTCCAACAACAACAATGTGAAGGCTACAATTGACAGGTACAAGAAGGCGCATGCTTGTGGCTCAACTTCTGGTGCACCTCTCATAGAGGTCAATGCTCAGCAATACTACCAGCAGGAGTCTGCCAAACTGCGCCACCAGATTCAGATGCTGCAAAACACCAACAAGCACCTGGTTGGCGATAATGTGAGCAACCTGTCACTGAAGGAGCTGAAGCAACTTGAAAGCCGCCTGGAGAAAGGCATTTCAAAGATCAGAGCCAGGAAGAATGAACTGCTGGCTTCAGAGATCAATTACATGGCCAAAAGGGAGATTGAGCTTCAGAACGACAACATGGACCTCAGAACCAAGATTGCTGAGGAGGAGCAGCAGCTGCAGCAGGTGACGGTGGCCCGGTCGGCCGCCATGGAGCTGCAGGCTGCGGCGGCGGCGCAGCAGCAGCAGCAGAATCCGTTCGCGGTGGCGGCGGCGCAGCTGGACATGAAGTGCTTCTTCCCGTTGAACCTGTTCGAGGCGGCGGCGCAGGTGCAGGCCGTGGCGGCGCAGCGCCAGCAGATCATCCCCACCGAGCTCAACCTCGGCTACCACCACCACCTTGCCATTCCCGGCGCCGCCGCCGCCGACGCGCCGCCTCCTCACTTCTGA

>LOC_Os03g54160.1 Oryza sativa subsp. japonica|MIKC_MADS|MIKC_MADS family protein

ATGGGGCGGGGCAAGGTGCAGCTGAAGCGGATCGAGAACAAGATCAACCGGCAGGTGACCTTCTCCAAGCGCAGGTCGGGGCTGCTCAAGAAGGCGAATGAGATCTCCGTGCTCTGCGACGCCGAGGTCGCGCTCATCATCTTCTCCACCAAGGGCAAGCTCTACGAGTACGCCACCGACTCATGTATGGACAAAATCCTTGAACGTTATGAGCGCTACTCCTATGCAGAAAAGGTCCTTATTTCAGCTGAATCTGACACTCAGGGCAACTGGTGCCACGAATATAGGAAACTGAAGGCTAAGGTTGAGACAATACAGAAATGTCAAAAGCACCTCATGGGAGAGGATCTTGAATCTTTGAATCTCAAAGAGCTGCAGCAGCTGGAGCAGCAGCTGGAAAATTCGTTGAAACATATCAGATCCAGAAAGAGCCAACTAATGCTCGAGTCCATTAACGAGCTTCAACGGAAGGAAAAGTCACTGCAGGAGGAGAATAAGGTCCTACAGAAAGAAAACCCTTGCTCCTTCCTACAGCTGGTGGAGAAGCAGAAAGTCCAGAAGCAACAAGTGCAATGGGACCAGACACAACCTCAAACAAGTTCCTCATCATCCTCCTTCATGATGAGGGAAGCCCTTCCAACAACTAATATCAGTAACTACCCTGCAGCAGCTGGCGAAAGGATAGAGGATGTAGCAGCAGGGCAGCCACAGCATGTTCGCATTGGGCTGCCACCATGGATGCTGAGCCACATCAACGGCTAA

>LOC_Os07g01820.1 Oryza sativa subsp. japonica|MIKC_MADS|MIKC_MADS family protein

ATGGGGCGGGGGAAGGTGCAGCTGAAGCGGATAGAGAACAAGATCAACAGGCAGGTGACGTTCTCCAAGAGGAGGAATGGATTGCTGAAGAAGGCGCACGAGATCTCCGTCCTCTGCGACGCCGAGGTCGCCGCCATCGTCTTCTCCCCCAAGGGCAAGCTCTACGAGTACGCCACTGACTCCAGGATGGACAAAATCCTTGAACGTTATGAGCGCTATTCATATGCTGAAAAGGCTCTTATTTCAGCTGAATCCGAGAGTGAGATAACTCTTCCCCAGTTGACAACATGCACTGCATCCAGAAGTACTCATGGTATTTGTTTTCAGTACTGTTTGATGAGTAAAACTTTGGGAAATTGGTGCCATGAATACAGGAAACTTAAGGCAAAGATTGAGACCATACAAAAATGTCACAAACACCTCATGGGAGAGGATCTAGAATCCCTGAATCTCAAAGAACTCCAACAGCTAGAGCAGCAGCTGGAGAGTTCATTGAAGCACATAATATCAAGAAAGAGCCACCTTATGCTTGAGTCCATTTCCGAGCTGCAGAAAAAGGAGAGGTCACTGCAGGAGGAGAACAAGGCTCTGCAGAAGGAACTGGTGGAGAGGCAGAAGAATGTGAGGGGCCAGCAGCAAGTAGGGCAGTGGGACCAAACCCAGGTCCAGGCCCAGGCCCAAGCCCAACCCCAAGCCCAGACAAGCTCCTCCTCCTCCTCCATGCTGAGGGATCAGCAGGCACTTCTTCCACCACAAAATATCTGCTACCCGCCGGTGATGATGGGCGAGAGAAATGATGCGGCGGCGGCGGCGGCGGTGGCGGCGCAGGGCCAGGTGCAACTCCGCATCGGAGGTCTTCCGCCATGGATGCTGAGCCACCTCAATGCTTAA

>LOC_Os06g49840.1 Oryza sativa subsp. japonica|MIKC_MADS|MIKC_MADS family protein

ATGGGGAGGGGCAAGATCGAGATCAAGCGGATCGAGAACGCGACCAACAGGCAGGTGACCTACTCGAAGCGCCGCACGGGGATCATGAAGAAGGCCAGGGAGCTCACCGTGCTCTGCGACGCCCAGGTCGCCATCATCATGTTCTCCTCCACCGGCAAGTACCACGAGTTCTGCAGCCCTTCCACCGACATCAAGGGGATCTTTGACCGCTACCAGCAAGCCATCGGCACCAGCCTTTGGATCGAGCAGTATGAGAATATGCAGCGCACGCTGAGCCATCTCAAGGACATCAACCGCAACCTGCGCACCGAGATCAGGCAAAGGATGGGAGAAGATCTGGACGGGCTGGAGTTCGACGAGCTGCGCGGTCTTGAGCAAAATGTCGATGCCGCCCTCAAGGAGGTTCGCCACAGGAAGTATCATGTGATCACCACACAGACTGAAACCTACAAGAAAAAGGTGAAGCACTCCTACGAGGCGTACGAGACTCTGCAGCAGGAGCTGGGGTTGCGCGAGGAGCCGGCGTTCGGGTTCGTGGACAACACCGGCGGCGGGTGGGACGGCGGCGCCGGCGCCGGCGCGGCGGCGGACATGTTCGCCTTCCGCGTGGTGCCCAGCCAGCCCAACCTGCACGGCATGGCCTACGGCGGCAACCACGACCTGCGCCTCGGTTGA

>LOC_Os04g49150.1 Oryza sativa subsp. japonica|MIKC_MADS|MIKC_MADS family protein

ATGGATCGATCAGAGATGGGGAGGGGAAGGGTTGAGCTGAAGCGCATCGAGAACAAGATCAACCGGCAGGTCACCTTCTCCAAGCGCCGCAACGGCCTCCTCAAGAAGGCGTACGAGCTCTCCGTGCTCTGCGACGCGGAGGTGGCGCTCATCATCTTCTCCAGCCGTGGCAAGCTGTACGAATTCGGTAGCGCCGGAATAAACAAGACATTGGAAAAGTACAATAGTTGCTGTTACAACGCTCAAGGTTCAAATAGTGCTCTTGCTGGTGGTGAACATCAGAGCTGGTACCAAGAGATGTCAAGGCTCAAGACTAAGCTTGAATGTCTCCAACGCTCTCAGAGGCACATGCTTGGTGAAGATCTTGGACCATTGAGCATAAAGGAACTGCAGCAGCTGGAGAAGCAACTTGAGTACTCACTGTCACAGGCTCGACAACGAAAGACACAAATCATGATGGAGCAGGTCGACGATCTTCGCCGGAAGGAACGCCAGCTTGGAGAGCTCAATAAGCAACTGAAAAACAAGCTAGAAGCTGAAGCCGATAGCAGCAACTGCAGATCAGCCATCCAGGATTCCTGGGTCCATGGCACCGTCGTCAGTGGCGGCAGAGTGCTGAATGCTCAACCACCACCAGATATTGACTGTGAGCCTACTCTGCAAATTGGGTACTATCAATTTGTCCGTCCTGAGGCGGCCAATCCAAGAAGCAATGGAGGAGGAGGGGATCAGAACAACAACTTTGTGATGGGATGGCCCCTCTGA

>LOC_Os07g41370.1 Oryza sativa subsp. japonica|MIKC_MADS|MIKC_MADS family protein

ATGGGGAGAGGGCCGGTGCAGCTGCGGCGGATCGAGAACAAGATAAACAGGCAGGTGACCTTCTCCAAGCGGAGGAACGGGCTGCTGAAGAAGGCGCACGAGATCTCCGTGCTCTGTGACGCCGACGTCGCGCTCATCGTCTTCTCCACCAAGGGCAAGCTCTACGAGTTCTCCAGCCACTCCAGTATGGAAGGGATCCTTGAACGCTACCAGCGTTACTCGTTTGATGAAAGAGCCGTACTGGAGCCAAATACTGAGGACCAGGAAAACTGGGGTGATGAATATGGAATTTTGAAGTCCAAACTGGATGCACTTCAGAAGAGCCAAAGGCAACTCTTAGGTGAACAATTGGACACACTAACAATAAAAGAACTCCAGCAATTGGAACATCAACTGGAATATTCTCTGAAGCATATAAGATCAAAAAAGAATCAGCTTCTGTTTGAATCAATTTCTGAGCTTCAGAAGAAGGAAAAGTCACTTAAAAACCAGAATAATGTTCTGCAAAAGCTCATGGAGACAGAAAAGGAGAAAAACAATGCTATAATAAACACTAACCGGGAGGAGCAAAATGGAGCAACACCAAGCACATCATCACCAACACCAGTGACGGCTCCAGATCCCATCCCGACAACAAATAACAGTCAAAGCCAACCAAGAGGATCAGGGGAGTCAGAAGCTCAACCGTCTCCGGCACAAGCAGGCAACAGCAAGCTTCCGCCATGGATGCTCCGGACAAGTCACACATGA

>LOC_Os01g66030.1 Oryza sativa subsp. japonica|MIKC_MADS|MIKC_MADS family protein

ATGGGGCGCGGGAAGATCGAGATCAAGAGGATCGAGAACTCCACCAACCGCCAGGTGACCTTCTCCAAGCGCAGGAGCGGGATCCTCAAGAAGGCCCGCGAGATCAGCGTCCTGTGCGACGCCGAGGTCGGCGTCGTCATCTTCTCCAGCGCCGGCAAGCTCTACGACTACTGCTCCCCCAAGACCTCGCTATCAAGAATCTTGGAGAAGTACCAGACCAATTCCGGAAAGATACTGTGGGATGAGAAGCACAAGAGCCTTAGCGCGGAGATTGATCGAATCAAGAAAGAGAACGATAATATGCAGATTGAGCTCAGGCACTTGAAAGGTGAAGATCTAAACTCTCTGCAGCCCAAAGAGCTCATCATGATTGAGGAGGCACTTGACAATGGGATAGTGAACGTGAATGATAAACTGATGGACCACTGGGAAAGGCACGTGAGAACTGATAAGATGCTGGAAGACGAGAACAAGCTGCTGGCTTTTAAACTGCACCAGCAAGATATAGCGCTGAGCGGGAGCATGAGGGATCTTGAGCTTGGGTACCATCCAGACAGGGACTTTGCGGCCCAGATGCCGATCACCTTCCGCGTGCAGCCCAGCCACCCCAACCTGCAGGAGAACAATTAA

>LOC_Os12g31748.1 Oryza sativa subsp. japonica|MIKC_MADS|MIKC_MADS family protein

ATGGGGAGGGGGAAGGTGCAGGTGCGGCGGATCGAGAACGAGGTGAGCCGGCAGGTGACCTTCTCCAAGCGGCGGCCGGGGCTGCTCAAGAAGGCCCACGAGATCGCCGTCCTCTGCGACGTCGACGTCGCCGCCATCGTCTTCTCCGCCAAGGGCAACCTCTTCCACTACGCCTCCTCCCACACCACTATGGAGCGAATCCTTGAGAAGTATGACAGACATGAGTTATTATCTGAAGGAAATAATGTGATTGAAGAGTTCCCTGAGCTGGAGGGAAGCATGAGCTATGACCACATCAAGCTGAGGGGCAGGATTGAAGCTCTAAAAAAGAGCCAAAGGAATCTTATGGGGCAGGAACTTGACTCGCTGACACTGCAAGATATCCAGCAGCTTGAGAACCAGATAGACACTTCTCTGAATAACATAAGATCAAGAAAGGAAAAGTTGCTGATGGAGAAAAACACTATTCTGGAGAAGAAAATTACTGAACTGGAGACACTGCATACATGCATCAGGGCGTCACCCACTAAAGCTGCTGCTCCTCCTGCCTGCAATACTGCTGATGCATTTGTTCCCAACCTCAACATCTGCTGCGGCGATTCCGGCGAGCCGGAAACCGTGACGGCGCCACTTGGCTGGACCAGCAGCAACAATGGCTTGCCATGGTGGATGCTCCAGTCATCATCGAACGGCAAGAGCTAG

>LOC_Os01g66290.1 Oryza sativa subsp. japonica|MIKC_MADS|MIKC_MADS family protein

ATGGGGAGGGGGAAGATTGAGATAAAGAGAATCGAGAACAAGACGAGCCGTCAGGTGACCTTCTGCAAGCGCAGGAATGGGCTGCTGAAGAAGGCCTACGAGCTCGCCATACTCTGCGACGCTGAGATCGCACTCATCGTCTTCTCCAGCCGAGGACGCCTCTATGAGTTCTCCAATGTAAACAGCACAAGGTCAACAATCGAGAGGTACAAGAAAGCCTCTGCCAGCACTTCAGGATCCGCTCCAGTGATAGACGTCAATTCTCATCAATACTTTCAGCAAGAAGCAGCAAAAATGCGCCACCAGATACAGACCTTGCAGAATGCAAACAGGCACCTCATCGGTGAGTCCATTGGCAATATGACCGCAAAGGAGCTCAAGAGCCTTGAAAATCGGCTTGAAAAGGGCATCAGCCGAATTCGATCAAAGAAGCATGAGCTGCTGTTCTCGGAGATCGAATACATGCAGAAAAGGGAAGCAGATCTTCAGAACGAGAACATGTTCCTGAGAGCCAAGGTGGCAGAGGCCGAGCGAGCTGAACATGATGATCAGCAGGCGGCGGAGGACGACGAGATGGCGCCGGCGCCGGCGGTCGGCGGCGGGTCATCGTCGGGGACAGAGCTGGAGGCGCTGCCGGCGACGTTCGACACAAGGGAATACTACCAGCCGGCGCCGCCGGTGAGCATGCTGGCCGCCGCGGCGGCGGCGGCGGCGGCGCAGTACTCGTCGGACCACCACCAGACTGCTCTCCACCTCGGCTACTTCAAGGTCGACTCCGGCAAAGGCGGCCTCCTCTAG

>LOC_Os02g52340.1 Oryza sativa subsp. japonica|MIKC_MADS|MIKC_MADS family protein

ATGGCGCGGGAGAGGCGGGAGATAAAGAGGATAGAGAGCGCGGCGGCGCGGCAGGTCACCTTCTCCAAGCGCCGGCGCGGCCTGTTCAAGAAGGCCGAGGAGCTCTCCGTGCTCTGCGACGCCGACGTCGCGCTCATCGTCTTCTCCTCCACGGGGAAGCTATCCCACTTCGCAAGCTCCAGTATGAATGAAATCATCGACAAGTACAACACACATTCTAATAATCTGGGGAAAGCAGAACAGCCTTCGCTCGACTTGAATTTAGAACATAGCAAGTATGCACATTTGAATGAGCAACTTGCGGAAGCTAGTCTCCGGCTTAGACAAATGAGAGGTGAGGAGCTTGAGGGATTGAGCATTGATGAACTCCAGCAGCTAGAGAAGAACCTGGAAGCTGGTCTGCACAGGGTGATGCTGACAAAGGATCAACAATTTATGGAGCAGATCAGTGAACTCCAGCGGAAGAGTTCACAGCTGGCAGAGGAGAACATGCAACTCAGAAACCAGGTATCCCAGATATCACCAGCTGAGAAGCAAGTTGTTGATACTGAAAATTTTGTTACCGAAGGACAGTCCTCTGAATCTGTGATGACTGCATTGCATTCTGGAAGTTCACAGTCGCAGGATAATGATGATGGCTCGGATGTATCCCTGAAATTAGGGCTGCCTTGTGGTGCATGGAAGTAA

>LOC_Os08g33488.1 Oryza sativa subsp. japonica|MIKC_MADS|MIKC_MADS family protein

ATGGGGAGAGGGAAGATAGAGATAAAGAGGATCGACAACGCGACGAGCCGACAGGTGACATTCTCGAAGCGGCGGAGCGGGCTGTTCAAGAAGGCGAGGGAGCTCTCCATCCTCTGCGATGCCGAGGTCGGCCTCCTCGTCTTCTCCAGCACCAGCCGTCTCTATGACTTTGCCAGCTCCAGCATGAAATCCATAATTGAGAGATACAATGAGACGAAAGAAGATCCCCATCAAACCATGAACGCAAGTTCTGAGGCAAAGCTTTGGCAACAGGAGGCAGCAAGCTTGAGGCAGCAACTGCATAACTTGCAAGAATATCATCGACAGTTGTTGGGACAACAGCTTTCTGGTCTGGATGTAGAAGACTTGCAAAATTTGGAAAGTAAGCTGGAGATGAGCTTAAAAAATATCCGTCTGAGGAAGGACAATGTCATGATGGATCAAATTCAAGAATTAAGCAGGAAGGTTGTTACCACTTAG

>LOC_Os04g23910.1 Oryza sativa subsp. japonica|MIKC_MADS|MIKC_MADS family protein

ATGGGGAGAGGGAAGATTGCCATCAAGAGGATCGACAACACGATGAACCGGCAGGTGACCTTCTCGAAGCGGCGCGGCGGGCTGATGAAGAAGGCCCGGGAGCTGGCCATCCTCTGCGACGCCGACGTCGGCCTCATTGTCTTCTCCTGCACCGGCCGCCTCTACGACTTCTCCAGCTCAAGCATGAAATCAATAATAGAGCGGTACCAGGAGGCAGGAGAGGAGCATTGTCGGTTGCTGAACCCAATGTCAGAGGCTAAGTTTTGGCAGCGGGAGGTTACAACTTTGAGGCAGCAAGTGCAAAACTTACACCACAACAACAGGCAACTTTTGGGAGAGGAAATCTCCAACTTCACAGTTAGAGATCTGCAGCTTCTCCAGAACCAAGTTGAGATGAGCCTACATTCCATAAGAAATAAAAAGGATCAACTTTTGGCAGAGGAGATTCTAAAACTCAATGAAAAGGGGTCTCTTGTTCAAAAGGAGAACAGTGAACTTCGCAAGAAGTTCAACATTGCTCATCAACGCAACATAGAATTACACAAGAAGCTTAACTCTGGAGAAAGCACGTCAAGTGAGCAAGTTACCAGAAGCTCAAAGGATCCCGGAGAATCGAGTACACCCCGTGATTCACGTGTGTGTATTGACCTTGAATTGAGTCAAAAAGAAGTTGAAGATGAATAA

>LOC_Os08g02070.1 Oryza sativa subsp. japonica|MIKC_MADS|MIKC_MADS family protein

ATGGCGCGAGGCAAGGTGCAGCTCCGTCGCATCGAGAACCCGGTTCACCGTCAGGTCACCTTCTGCAAGCGCCGTGCCGGCCTGCTGAAGAAGGCCAGGGAGCTCTCCATCCTCTGCGAGGCCGACATCGGCATCATCATCTTCTCCGCCCACGGCAAGCTCTACGACCTCGCCACCACCGGAACCATGGAGGAGCTGATCGAGAGGTACAAGAGTGCTAGTGGCGAACAGGCCAACGCCTGCGGCGACCAGAGAATGGACCCAAAACAGGAGGCAATGGTGCTCAAACAAGAAATCAATCTACTGCAGAAGGGCCTGAGGTACATCTATGGGAACAGGGCAAATGAACACATGACTGTTGAAGAGCTGAATGCCCTAGAGAGGTACTTAGAGATATGGATGTACAACATTCGCTCCGCAAAGATGCAGATAATGATCCAAGAGATCCAAGCACTAAAGAGCAAGGAAGGCATGTTGAAAGCTGCTAACGAAATTCTCCAAGAAAAGATAGTAGAACAGAATGGTCTGATCGACGTAGGCATGATGGTAGCAGATCAACAGAATGGGCATTTTAGTACAGTCCCACTGTTAGAAGAGATCACTAACCCACTGACTATACTGAGTGGCTATTCTACTTGTAGGGGCTCGGAGATGGGCTATTCCTTCTAA

>LOC_Os02g36924.1 Oryza sativa subsp. japonica|MIKC_MADS|MIKC_MADS family protein

ATGGGGAGGGGGAAGATTGTGATCCGCCGGATCGACAACTCGACGAGCCGGCAGGTGACGTTCTCGAAGCGGAGGAACGGGATCTTCAAGAAGGCCAAGGAGCTGGCCATCCTCTGCGACGCCGAGGTCGGCCTCATGATCTTCTCCAGCACCGGCCGCCTCTACGAGTACTCCAGCACCAGCATGAAGTCAGTTATAGATCGGTATGGCAAGTCCAAGGATGAGCAGCAAGCCGTCGCAAATCCCAACTCGGAGCTTAAGTTTTGGCAAAGGGAGGCAGCAAGCTTGAGACAACAACTGCACAACTTGCAAGAAAATCATCGGCAGTTGATGGGCGAAGATCTATCTGGGCTGAATGTTAAGGAATTGCAATCTCTAGAGAATCAGCTGGAAATAAGTCTACGTAGTGTCCGTACAAAGAAGGACCACGTCTTGATTGATGAAATTCATGAACTGAATCGGAAGGGAAGTCTAGTTCACCAAGAAAACATGGAATTATACAAGAAGATCAGTTTAATTCGTCAAGAAAATGCTGAGTTATATAAGAAGATCTACGAGACTGAAGGACCAAGTGAAGTCAATCGGGATTCACCAACTCCTTACAATTTTGCAGTAATTGAAAAAACAAATGTTCCTGTGCAACTTGGACTCAGCACACTACCACAACATAGTGACGCCGAACAATCAACTGCTCCTAAGCTAGGGTTACAGTTGAATCCATGA

>LOC_Os02g07430.1 Oryza sativa subsp. japonica|MIKC_MADS|MIKC_MADS family protein

ATGGGGCGCGGCAAGATCGAGATCAAGAGGATCGAGAACGCGACGAACAGGCAGGTGACATTCTCGAAGAGGCGGGGAGGGCTACTGAAGAAGGCGAACGAGCTCGCCGTGCTCTGCGACGCCCGCGTCGGCGTCGTCATCTTCTCCAGCACCGGCAAGATGTTCGAGTACTGCAGCCCTACCTGCAGTTTGAGGGAACTCATCGAGCATTACCAGACCGTCACCAACACTCATTTCGAGGAGATCAACCACGATCAGCAAATATTTGTGGAGATGACTCGGATGAGGAACGAGATGGAGAAGCTGGACGGTGGCATCAGGAGGTTCACCGGCGACGACCTCTCCAACCTCACGCTCGCCGACATCAACGATCTCGAGCAGCAGCTCGAATTCTCCGTCACCAAAGTCCGTGCGAGAAAGCATCAGCTCCTGAACCAGCAGCTGGACAACCTTCGTCGCAAGGAGCACATCCTGGAAGACCAGAACAGCTTCCTGTGCCGCATGATCAACGAGAACCATCATCAGGCGGCGGTGGGCGGCGGCGACGTGAAGGCGATGGTGGAGATGGCGCCGGTGCTGTCGATGCTGACGGCGGCGCCGGCGTACTACGGCGAGGAGTCGTCGAGCACCGCGCTGCAGCTCACCCCGCCGCTGCACGCCGTCGACGCCGCCGCCGCCGCCGGGTTCCGGCTGCAGCCGACGCAGCCCAACCTGCAGGACCCCGGCTGCAGCAGCAGCAGCTTCCATGCCGCCGCCGCCGGCCACGGCCTGCAGCTGTGGTAA

>LOC_Os01g10504.1 Oryza sativa subsp. japonica|MIKC_MADS|MIKC_MADS family protein

ATGATGAACATGATGACCGATCTGAGCTGCGGGCCATCGTCGATGACGGAGCTGACCGCGGCAGCGGCGCCGGCTGGGTCAGGATCGTCGGCGGCGGTGGCGGCGGGGAGCAGCGAGAAGATGGGGAGGGGGAAGATCGAGATAAAGCGGATCGAGAACACGACGAACCGGCAGGTGACCTTCTGCAAGCGCCGCAATGGCCTCCTGAAGAAGGCGTACGAGCTGTCCGTCCTCTGCGACGCCGAGGTTGCCCTCATCGTCTTCTCCAGCCGCGGCCGCCTCTACGAGTACGCCAACAACAGTGTGAAATCCACCGTTGAGAGGTACAAGAAGGCAAACAGTGACACCTCCAACTCTGGCACAGTTGCAGAAGTCAATGCCCAGCACTACCAGCAGGAGTCCTCCAAACTGCGCCAACAAATCAGTAGCTTACAGAACGCAAACAGTAGGACCATAGTGGGGGATTCTATCAACACCATGAGCCTCAGGGACCTTAAACAGGTAGAGAACAGGCTGGAGAAAGGCATAGCTAAGATAAGGGCTAGAAAGAATGAGCTGTTATATGCTGAAGTTGAGTACATGCAGAAAAGGGAAGTTGAGCTGCAGAATGACAACATGTACCTGAGGAGCAAGGTTGTTGAGAATGAGAGGGGACAGCAGCCACTGAACATGATGGGGGCAGCATCAACAAGTGAATACGATCATATGGTTAATAACCCATATGATTCCAGGAACTTTCTTCAAGTGAACATCATGCAGCAGCCTCAGCATTACGCCCATCAGCTGCAGCCAACTACCCTTCAACTCGGCAGCCGGCCTTCAATTAGTTTTGGTGTAGACACCGTACGTACACACGTACGTTAG

>LOC_Os06g45650.1 Oryza sativa subsp. japonica|MIKC_MADS|MIKC_MADS family protein

ATGGGGCAAGGGAAGATCGAGATGAAGAGGATCGAGGACGCGACGAGGCGGCAGGTGACGTTCAGCAAGCGCAGGGCTGGGTTTCTCAAGAAGGCGAACGAGCTCGCCGTGCTGTGCGATGCGCAGGTCGGCGTCGTCGTCTTCTCCGACAAGGGCAAGCTCTTCGACTTCTGCAGCCCGCCGGTCATCTTGATGGAGCTGTTTCACCGTTATGAGATCACCACCAGAAACACTCGGCTTCAGGAGACAAACCGTGATGATGAGCAAATGGTCATGGAGATCACAAGGCTAAGGAATGAGATCGACCAGCTCGAAGCCAGTTTAAGGAGGCAAACTGGAGAAGACCTGTCATCTGTGTCCACAGTGGATGAGCTCAGCCAGCTGCAGCTGCAGCTTGAATCATCTCTCAGCAAAGTTCATGCGAGGAAGGATGAGCTCATGAGCCAGCAGCTGGAGGATATGCGTCGCATGCATCAGACCGTGCATGAGCAGAACAATTTCTTGTGCCGCATGGTAACTAAAATACTCCTACTAAATGTGATCACAATAATGATACTTGCTGCCATGATTTCTATCACTCATGCACGCATTGCCCTTGATGATTGTATGCAGTTGGGATATATTGTCATCAAGCAAGAAAATTCTTGGCGATTCTGGTTTATATGA

>LOC_Os04g52410.1 Oryza sativa subsp. japonica|MIKC_MADS|MIKC_MADS family protein

ATGGGGCGTGGGAGAGTAGAGCTCAAAAAGATTGAAAATCCGACAAACCGCCAAGTTACCTTCTCCAAGAGGAGGATGGGGCTGCTCAAGAAAGCAAATGAGCTGGCCATTCTTTGTGATGCACAAATTGGGGTGATTGTGTTCTCAGGCACTGGCAAGATGTACGAGTACTCCAGCCCCCCTTGGAGGATTGCAAATATCTTTGATAGATACCTGAAAGCCCCCAGCACCCGTTTTGAGGAGATGGATGTTCAGCAGAGAATCATCCAAGAGATGACAAGAATGAAGGATGAGAACAACAGGCTTAGGATCATCATGAGGCAGTATATGGGTGATGACTTGGCTTCACTGACTCTGCAAGATGTGAGTAATCTTGAGCAGCAGATTGAGTTTTCTCTGTACAAAGTTCGTCTAAGGAAGCAACAGCTACTTGATCAGCAGCTGCTTGAGATGCACAGCCGGGTATGTAACAAGAGGATATACCGGTTCAGTATTCTTCCCTACCTTATACACTATATTGAAATAATCAAGTTTTGA

>LOC_Os01g52680.1 Oryza sativa subsp. japonica|MIKC_MADS|MIKC_MADS family protein

ATGGGGAGGGGGCGCAGCGAGATAAAGAGGATAGAGAACCCCACGCAGCGGCAGTCCACCTTCTACAAGCGCAGGGACGGCCTGTTCAAGAAGGCCAGGGAGCTCGCCGTCCTCTGCGACGCCGACCTCCTCCTCCTCCTCTTCTCCGCCTCCGGCAAGCTCTACCACTTCCTCTCCCCCACCGTCCCCTCCGTGAGGGAGTTTGTCGAGAGGTACGAGGCCACCACGCACACCAAGGTTTGGGCAGATATCAGGCAGGAGAGGCGCGCCGAGCTGGAGAAGGTGGGCAGCATGTGCGACCTCCTGGAGAAACAGCTGAGGTTCATGACGGTGGACGACGGCGAGGAGTACACGGTGCCGTCGCTGGAGGCGCTGGAGCACAATCTGGAGGCCGCCATGCGCAAGGTGCGCTCCGAGAAGGACCGCAAGATCGGAGGCGAGATCTGCTACCTCCAGAACATTATTAGGGGGCGACAAGAGGAGCGGTACGGGCTGTGCGACAAGATTGCTCATGCACAGACTCTGAAGGATGTGGAATGTGGATCCACCTCACTAAGCAATGGCTTGGACCTTAAACTGGGGTTCAACTAG

>LOC_Os12g10520.1 Oryza sativa subsp. japonica|MIKC_MADS|MIKC_MADS family protein

ATGGTCAGAGGAAAGGTGCAAATGCGACGAATAGAGAACCCTGTCCACAGACAGGTAACCTTCTGTAAGCGCCGTGGGGGGCTCCTCAAGAAGGCCAGGGAACTATCAGTGCTTTGTGATGCTGATGTAGGTGTTATCATATTCTCCTCTCAGGGAAAACTCCATGAATTAGCTACTAATGGAAATATGCATAACTTGGTTGAAAGGTACCAGAGCAATGTAGCAGGTGGTCAAATGGAACCAGGAGCTCTACAAAGACAGCAGGTAGCAGAACAAGGGATTTTCTTGTTAAGGGAAGAAATAGATCTACTACAGAGGGGTCTTAGGTCTACATATGGAGGGGGAGCAGGGGAAATGACACTAGATAAATTGCATGCACTAGAGAAAGGTCTTGAACTATGGATTTACCAAATACGCACAACAAAGATGCAGATGATGCAACAAGAGATTCAATTTCTTAGAAACAAGGAAGGCATACTAAAAGAAGCAAATGAAATGCTTCAAGAGAAGGTGAAAGAACAGCAAAAGCTATACATGTCTTTGTTGGATCTCCATAGTCAACAACCAACACAACCAATGACCTACGGAAATCGCTTCTTCTCAATCTAG

>LOC_Os03g54170.1 Oryza sativa subsp. japonica|MIKC_MADS|MIKC_MADS family protein

ATGGGGCGAGGCAAGGTGGTGCTTCAGCGGATCGAGAACAAGATCAGCCGGCAGGTGACGTTCGCCAAGCGGCGGAACGGCCTGCTCAAGAAGGCCTACGAGCTCTCCATCCTCTGCGACGCCGAGGTCGCCCTCGTCCTCTTCTCCCACGCCGGCCGCCTCTACCAGTTCTCCTCCTCATCCAACATGCTTAAGACGCTTGAGAGATACCAGAGGTACATTTATGCTTCGCAAGATGCTGCCGCACCAACTAGCGATGAGATGCAGAACAACTATCAGGAATATGTGAACTTGAAGGCACATGTTGAGATTCTGCAACAATCACAAAGGAACCTTCTAGGTGAGGATTTAGCTCCACTGGCTACAAATGAACTTGAGCAGCTTGAGAGTCAAGTAGTCAGAACCTTGAAGCAAATCAGATCAAGAAAGACTCAGGTACTACTTGATGAACTCTGCGACCTAAAGAGAAAGGAACAAATGCTACAAGATGCAAACAGGGTCCTGAAAAGGAAGCTTGACGAGATCGACGTAGAGGCAGCTCCCCCACAGCCTCCATGGAACGGAAACTGCAGCAATGGCCATGGCGGCGGCGGCGGCGTGTTTTCCAGTGAGCCTCCCCAACCAGAGCACTTCTTCCAGGCCCTCGGGCTTCATGCCGTGGACGTGAACCAGCCGCCGGCGCCGCCACCGGGCGGTTATCCTCCTGAGTGGATGGCCTAG

>LOC_Os08g41960.1 Oryza sativa subsp. japonica|MIKC_MADS|MIKC_MADS family protein

ATGGAGGGAGGAGGGAGGAGGAGGAAGAGGGGGAAGGTGGAGCTGCGGCGGATAGAGGACCGGACGAGCCGGCAGGTGCGATTCTCGAAGCGGCGGAGCGGGCTGTTCAAGAAGGCGTACGAGCTGTCCGTGCTCTGCGACGCCCAGGTCGCCCTCCTCGTCTTCTCCCCCGCCGGCCGCCTCTACGAGTTCGCCTCTTCCACCTCCAGCATTGATACAATTTTTGGTCGGTATTGGGACCTTCTGGACACAACAATTGATCTCAATATTGAAGCAAGGGAATCTCGGGTTGATTGCAATATACAGCTTCGTCAGAAAGAGCGTTCAGATGACCCGGTGCCTAAGATAAACCACATTACTCAATGTGTGTTGGAATCAAATGTCAACGAGCTGAACATCGCTGAGCTAAGAGGTTTGGAGGAAGCGATGACTAATGCTTTGACAGTTGTTAAGAACAAACTGATGATGAAGGTGGCTAGTGTGCTCCCCCAAAGCGAGAAGAAGAGGAAGAGTTGCTCGATTTCAGAGCCAAGATCAGGAGTGAGCTCTTAA

>LOC_Os05g34940.1 Oryza sativa subsp. japonica|MIKC_MADS|MIKC_MADS family protein

ATGGGGCGCGGCAAGATCGAGATCAAGAGGATCGAGAACTCGACGAACCGGCAGGTGACGTTCTCGAAGCGCCGCGCCGGGATACTCAAGAAGGCCCGCGAGATCGGCGTGCTCTGCGACGCCGAGGTCGGCGTCGTCATCTTCTCCAGCGCCGGCAAGCTCTCCGACTACTGCACGCCCAAGACCACGTCCGTGTTTCCCCCGCTGTCAAGGATCTTGGAGAAGTACCAGACCAACTCCGGGAAGATACTCTGGGATGAGAAGCACAAGAGCCTCAGCGCAGAGATCGATCGTGTCAAGAAGGAGAACGACAACATGCAGATCGAGCTCAGGCATATGAAAGGGGAGGATCTGAACTCCCTGCAGCCCAAGGAGCTGATCGCGATCGAGGAGGCGCTCAACAACGGCCAGGCCAATCTGCGGGACAAGATGATGGACCACTGGAGGATGCATAAAAGGAATGAGAAGATGCTGGAGGACGAGCACAAGATGTTGGCTTTTAGGGTGCACCAGCAGGAGGTCGAGCTGAGCGGCGGCATAAGGGAGCTGGAGCTCGGCTACCACCACGACGACAGGGACTTCGCGGCCTCGATGCCGTTCACCTTCAGGGTGCAGCCCAGCCACCCCAACCTCCAGCAGGAGAAGTAG

>LOC_Os03g08754.1 Oryza sativa subsp. japonica|MIKC_MADS|MIKC_MADS family protein

ATGGCTGGCGGCGGCGGTGGCGGGGGAAGGGGGGAGGGGGAGGGGAGGGCGGCGACGGGGAAGAGGGAGAGGATAGCAATACGGAGGATCGACAACCTGGCGGCGAGGCAGGTGACCTTCTCGAAGCGGAGGAGGGGGCTGTTCAAGAAGGCCGAGGAGCTCTCCATCCTCTGCGACGCCGAGGTCGGGCTCGTCGTCTTCTCCGCCACCGGCAAGCTCTTCCAATTCGCCAGCACCAGCATGGAACAGATTATTGACCGGTACAACTCGCATTCCAAGACACTTCAGAGAGCAGAACCTTCTCAACTCGACTTACAAGGGGAGGACAGCAGTACTTGTGCCAGACTAAAGGAGGAGCTTGCAGAAACTAGCCTTAGGCTGAGGCAGATGAGAGGAGAGGAGCTGCACAGGCTGAATGTGGAACAGCTGCAGGAGCTAGAGAAGAGCCTCGAGTCCGGTCTAGGCTCTGTTCTCAAGACCAAGAGCAAGAAAATTCTGGATGAGATCGATGGACTGGAACGAAAGAGGATGCAATTGATAGAGGAGAATTTAAGGCTGAAGGAGCAACTGCAGGTGTCCAGGATGTCAAGAATGGAGGAGATGCAGCCTGGGCCTGATTCAGAAATCGTGTACGAGGAAGGGCAGTCGTCTGAATCTGTCACCAATGCTTCTTACCCGCGACCTCCACCCGACAACGACTACAGCTCTGATACATCTCTCAGGCTCGGGTTGTCACTCTTCAGCTCCAAGTGA

>LOC_Os06g06750.1 Oryza sativa subsp. japonica|MIKC_MADS|MIKC_MADS family protein

ATGGGGCGAGGGAAAGTAGAGCTGAAGCGGATCGAGAACAAGATAAGCCGGCAGGTGACGTTCGCGAAGAGGAGGAACGGGCTGCTGAAGAAGGCGTACGAGCTGTCCGTGCTCTGCGACGCCGAGGTCGCCCTCATCATCTTCTCCACCCGCGGCCGCCTCTTCGAGTTCTCCACCTCCTCCTGTATGTACAAGACACTGGAGCGATACCGCAGTTGCAACTACAACCTTAACTCATGTGAAGCATCTGCTGCACTGGAAACTGAACTAAGCAATTACCAAGAGTACTTAAAGTTAAAGACAAGAGTTGAGTTCCTACAAACAACTCAGAGAAATCTTCTTGGCGAGGACTTGGTTCCACTTAGCTTGAAGGAGCTCGAGCAACTTGAGAACCAGATCGAGATATCCCTCATGAATATCAGGTCATCAAAGAATCAACAGTTGCTTGATCAAGTATTTGAGCTCAAACGTAAGGAACAACAACTTCAAGATGCTAATAAAGACTTAAAAAGGAAGATACAAGAAACTAGTGGAGAAAATATGCTTCATATATCTTGCCAAGATGTAGGGCCCAGTGGCCATGCTAGTGAAGCTAACCAAGAGTTTCTCCATCATGCAATTTGTGACCCTTCCCTGCATATAGGGTATCAAGCTTACATGGATCACCTCAACCAATGA

>LOC_Os03g03100.1 Oryza sativa subsp. japonica|M-type_MADS|M-type_MADS family protein

ATGGTGCGGGGGAAGACGCAGATGAAGCGGATAGAGAACCCCACGAGCCGCCAGGTCACCTTCTCCAAGCGCCGCAACGGCCTGCTCAAGAAGGCCTTCGAGCTCTCCGTCCTCTGCGACGCCGAGGTCGCGCTCATCGTCTTCTCCCCGCGCGGCAAGCTCTACGAATTCGCCAGCGCCAGGAAAATTCGGCCTGAAAAGACGGCAAAAACTATATTTCCCCGTGTGGCGATTGAGTTGCCGTCTAAACAGTCACACTACTTCCATAAAGAAATTAGTTGTGAGACTGGCAAGGTTAGAAATTCTGAAAACATAGGCAACTTAGTACTGGCATTCCGGAGGGCAATATGA

>LOC_Os06g11330.1 Oryza sativa subsp. japonica|MIKC_MADS|MIKC_MADS family protein

ATGGCGAGGGAGAGGAGGGAGATACGGAGGATAGAGAGCGCGGCGGCGCGGCAGGTGACGTTCTCGAAGCGGCGGCGGGGGCTGTTCAAGAAGGCGGAGGAGCTGGCGGTGCTGTGCGACGCCGACGTCGCGCTCGTCGTCTTCTCCTCCACCGGCAAGCTCTCCCAGTTCGCCAGCTCCAATATGAACGAGATCATTGACAAGTATACTACACATTCAAAGAACCTGGGGAAAACAGATAAGCAGCCTTCTATTGATCTGAATATGAGAGGTGAGGAGCTTGAGGGATTGAGTGTGGAAGAGCTGCAGCAGATGGAAAAGAACCTCGAGGCAGGACTGCAGCGGGTGCTCTGTACAAAGGACCAGCAATTCATGCAAGAAATCAGTGAGCTCCAACGAAAGGGCATTCAGCTGGCAGAAGAGAATATGCGCCTCAGAGACCAAATGCCTCAGGTGCCTACTGCTGGCTTGGCGGTTCCTGATACTGAAAATGTTCTTACTGAAGATGGACAATCATCTGAATCTGTGATGACTGCATTAAATTCGGGAAGCTCGCAGGATAATGATGATGGTTCTGATATATCCCTGAAACTAGGGTGA

>LOC_Os10g39130.1 Oryza sativa subsp. japonica|MIKC_MADS|MIKC_MADS family protein

ATGGTGCGGGGGAGGACGGAGCTGAAGCGGATTGAGAACCCGACGAGCCGGCAGGTGACCTTCTCCAAGCGCCGGAATGGCCTCCTCAAGAAGGCGTTCGAGCTCTCCGTCCTCTGCGACGCCGAGGTCGCCCTCATCGTCTTCTCCCCCCGCGGCCGCCTCTACGAGTTCGCCAGCGCCCCCAGCCTACAGAAAACCATCGACCGCTATAAAGCATACACAAAGGATCATGTCAACAATAAGACAATTCAACAAGATATCCAGCAAGTCAAAGATGATACTTTAGGCTTGGCCAAGAAACTTGAAGCTCTTGATGAGTCCAGACGGAAAATATTGGGAGAAAATTTAGAAGGATTCTCTATTGAAGAACTGCGTGGTCTAGAAATGAAACTTGAGAAGAGCCTCCACAAGATAAGACTAAAGAAGACCGAGCTTCTGGAGCAGCAGATAGCCAAGCTGAAAGAGAAGGAGCGGACTTTGCTTAAAGACAACGAAAATTTACGCGGAAAGCATCGCAACCTTGAGGCTGCGGCGCTGGTGGCTAACCACATGACGACGACGACGGCGCCGGCGGCGTGGCCGCGGGACGTGCCTATGACGAGCAGCACAGCCGGCGCCGCCGACGCCATGGACGTGGAGACTGATCTGTACATTGGATTGCCCGGCACTGAGCGCTCCTCCAACCGGTCGGAGACAGGTTGA

>LOC_Os02g49840.1 Oryza sativa subsp. japonica|MIKC_MADS|MIKC_MADS family protein

ATGGGGAGGGGGAAGATAGTGATAAGGAGGATAGACAACTCGACGAGCAGGCAGGTGACGTTCTCGAAGCGTCGGAACGGGCTTCTGAAGAAGGCGAAGGAGCTATCCATCCTCTGCGATGCGGAGGTCGGCCTTGTCGTCTTCTCCAGCACCGGCAGGCTCTATGAGTTCTCCAGCACCAACATGAAAACTGTGATAGACCGGTATACCAACGCAAAGGAGGAGCTACTTGGCGGGAATGCAACTTCAGAAATTAAGATTTGGCAGAGGGAGGCAGCAAGCTTGAGGCAGCAACTGCACAACTTGCAAGAAAGCCACAAGCAACTGATGGGTGAGGAGCTTTCTGGCCTAGGTGTTAGAGACCTACAAGGTTTAGAGAATAGGCTTGAAATAAGTCTACGTAATATCAGAATGAGAAAGGACAATCTTTTGAAAAGTGAAATCGAGGAGTTACATGTGAAGGGAAGCCTAATTCACCAGGAAAACATCGAACTTTCTAGAAGCCTAAATGTCATGTCGCAACAAAAATTGGAACTGTATAACAAGCTTCAGGCCTGTGAACAGAGAGGTGCCACAGATGCAAATGAAAGTTCCAGCACTCCATACAGCTTTCGTATCATACAAAATGCTAATATGCCTCCTAGTCTTGAATTGAGCCAATCACAGCAAAGAGAAGGGGAGTGCAGCAAAACAGCTGCTCCAGAACTGGGACTTCATCTGCCTTAA

>LOC_Os05g11414.1 Oryza sativa subsp. japonica|MIKC_MADS|MIKC_MADS family protein

ATGCACATATACAAAGAGCAGGAGGCTGAACCATCCACTGGCCTGATGATGCCAGAGCCAGCACCTGTTGCTTCCCCCGGCTCCGGTGGCTCAGGTGGCTCAGGCTCGGTGGGAGCTGAGAAGATTGGGAGCAGGGGAAAGATTGAGATCAAGCGCATCGAGAACACGACGAACCGTCAAGTGACCTTCTGCAAGCGCCGCAGCGGGCTACTCAAGAAGGCGTATGAGCTCTCCGTGCTCTGCGATGCCGAGGTCGCCCTCGTCGTCTTCTCCAGCCGTGGCCGCCTCTACGAGTACTCCAACAACAGCGTGAAGGAAACTATTGAGAGGTACAAGAAAGCCAACAGTGACACCTCCAACGCCAGTACAGTTGCAGAGATCAATGCCCAGCACTACCAGCAGGAAGCTGCTAAGCTGAAGCAACAGATCACCAACCTGCAGAACTCCAACAGGACCCTAGTAGGTGATAATATCACCACCATGAACCACAGAGAACTTAAGCAGCTGGAAGGCAGACTGGACAAAGGCCTAGGAAAGATTAGAGCACGGAAGAACGAATTGCTGTGTGCTGAAATTGAGTATATGCAGAGAAGGGAAACGGAGCTGCAGAATGACAACATGTACTTAAAGAGCAAAGTTGCTGAGAGTGAAAGAGGACTGCAAACAGTGAACATGATGGGTTCAGCATCTACGAGCGAGTACGTGCAAAATATGATCCATTATGATCCAAGAAACTTCCTGCAATTCAACATCATGCATCAGCCTCAGTATTATCCTGAACAGGAGGACCGAAAGGCCTTTATGTCAGGTAAAAAATATTCTCAGTGTAACATAGTCAGAGTTCATAGTTCAACAAATGAAATTTGA

>LOC_Os06g23950.1 Oryza sativa subsp. japonica|M-type_MADS|M-type_MADS family protein

ATGGTTCGTGGGAAGACCGTCATCAGCAGGATCGAGAACACGACGAGCCGCCAGGTGACCTTCTCCAAGAGGAGGAGCGGGCTATTCAAGAAGGCCAAGGAGCTGGCCATCCTCTGTGATGCCCAGGTCGGCGTCCTGGTCTTCTCAAGCACTGGCCGTCTCTATGACTACTCCAACTCCAGTAATTCTCGCAATCTTACCTATGATCAGTTATTGGGGGATACCGTCTGTTAA

>LOC_Os02g45770.1 Oryza sativa subsp. japonica|MIKC_MADS|MIKC_MADS family protein

ATGGGGAGGGGAAGAGTTGAGCTGAAGCGCATCGAGAACAAGATCAACAGGCAGGTCACCTTCTCCAAGCGCCGCAACGGCCTCCTCAAGAAGGCCTACGAGCTGTCCGTTCTCTGCGACGCCGAGGTCGCGCTCATCATCTTCTCCAGCCGCGGCAAGCTCTACGAGTTCGGCAGCGCCGGCATAACAAAGACTTTAGAAAGGTACCAACATTGTTGCTACAATGCTCAAGATTCCAACAATGCACTTTCTGAAACCCAGAGTTGGTACCATGAAATGTCAAAGTTGAAAGCAAAATTTGAAGCTTTGCAGCGCACTCAAAGGCACTTGCTTGGGGAGGATCTTGGACCACTCAGCGTCAAAGAATTGCAGCAGCTGGAGAAACAGCTTGAATGTGCACTATCACAGGCGAGACAGAGAAAGACGCAACTGATGATGGAACAAGTGGAGGAACTTCGCAGAAAGGAGCGTCAGCTGGGTGAAATTAATAGGCAACTCAAGCACAAGCTCGAGGTTGAAGGTTCCACCAGCAACTACAGAGCCATGCAGCAAGCCTCCTGGGCTCAGGGCGCCGTGGTGGAGAATGGCGCCGCATACGTGCAGCCGCCGCCACACTCCGCGGCCATGGACTCTGAACCCACCTTGCAAATTGGGTATCCTCATCAATTTGTGCCTGCTGAAGCAAACACTATTCAGAGGAGCACTGCCCCTGCAGGTGCAGAGAACAACTTCATGCTGGGATGGGTTCTTTGA

>LOC_Os04g38770.1 Oryza sativa subsp. japonica|M-type_MADS|M-type_MADS family protein

ATGGGGAGGGGCAAGATAGTGATCCGGCGGATCGACAACTCGACGAGCCGGCAGGTGACGTTCTCGAAGCGGCGCAACGGGATCTTCAAGAAGGCCAAGGAGCTGGCCATCCTGTGCGACGCCGAGGTCGGCCTCGTCATCTTCTCCAGCACCGGCCGCCTCTACGAGTATGCCAGCACCAGCATGAAGTCAGTGATTGATCGATATGGGCGAGCTAAGGAGGAGCAGCAGCACGTCGCAAACCCCAACTCGGAGCTGAAGGAGTTTTGTAGCGTCTTCATATATATAACAGAGAACTAA

>LOC_Os08g38590.1 Oryza sativa subsp. japonica|M-type_MADS|M-type_MADS family protein

ATGGGGAGGGTGAAGCTGCCGATCAAGAGGATCGAGAACACGACGAACAGGCAGGTGACGTTCTCGAAGCGGCGGAACGGGCTGATCAAGAAGGCGTACGAGCTGTCCGTGCTCTGCGACATCGACGTCGCCCTCCTCATGTTCTCCCCCTCCGGCCGCCTCAGCCACTTCTCCGGCCGCCGCGGGGTGGAGGACGTGATTCTCCGGTACATGAACCTCTCGGAGCACGACAGGGGAGAAGCCATCCAGAATCGGGAGGAGATTCAGCAAGAGATATATTCTTCCCAGCAACAGCTGCAAATCACCGAGGACCGCCTTAGGATGTTCGAGCCAGATCCTGCGGCGTTCGGCACATCCAGCGAGGTCGACGGATGCGAGAAGTATCTCATGGAATTGCTGACCCGAGTCGTCGAAAGGAAGAACAACTTGTTGAGCAGTCACATGGCGCCGTTCGATGCAACGACAGCTGCGATGCAGGGTGCAGATGGGACGCAGATGTACGTGAGCCAGGCGGACGGGTTGGCCACGTTCGGTGGTGACGCTGCCATGTGGGGCCCAGATGGAGGCGCGGATCCTGGCCATCCGATGTTCAGTGCATCTGATCCGTTGATCTACCTCAGGGACCACGACGTGTACGACGCCAACTCACAGGTGGCCGGGCTGCACGGCGGCGACCCGTGCGCGGCCGGCGGCGCCGCGGCGGCGGCGGCCGCGGTGGGGTGCGTCGACGACGACGTCGCCGGAGGCCACGCCGCCGCCGCCGACGCGTGGAGGCAGGCGTACACCTGCACCGAGCTGCTCTCCACGCTCATCCCCACCACGCCGTTCCCCCTCATGCCGCATTGCCTGGGGCCGGAGGACCAGTACCTGTCGATGGAGCATGGCATGGTGGCGGCGGCGCAGGAGCCGGTGGAGGCGTCGACGGCGAGCTGCTCCTACGTTCCCAGCGACGAGAACTCCGGCACGCCGGTCATGGCGTACGACAGCAACCCGCCGCCGGCGAACATCGCCTGA

>LOC_Os06g11970.1 Oryza sativa subsp. japonica|M-type_MADS|M-type_MADS family protein

ATGGGACGGGTGAAGCTGCAGATCAAGAGGATCGAGAACATCCCGAACAGGCAGGTGACGTTCTCCAAGCGGCGAAACGGGCTGATCAAGAAGGCCTACGAGCTGTCCGTCCTCTGCGACATCGACATCGCCCTCCTCATGTTCTCCCCCTCCGGCCGCCTCAGCCACTTCTCCGGCCGCCGCAGGATCGAGGACGTGCTCACAAGGTACATCAATCTCCCAGAAAGTGACAGAGGAGGAACTATCCAAAACAGAGAGTATTTGATCAACATGCTCACCCAGCTCAAATGCGAGAGCGACGTCACCGAGGATCTCACTAACACGAGCAGTAAGGCGCCGGTTAACTCCAACATTGAGGAGCTTCAGCAAGAGATCAGGAGATGCCAGCACCAGATGCAGCTCACAGAGGAGCAATTGAGGATGTTTGAGCCGGACCCGGCCAGGTCTGCATCCATGGAGGACGTCGAGGCCAGCGAGAAGTTCATCGCCGGCATCCTCAGCCGCGTCGAGGAGAGGAAGAGGTATTTGCTGTGCAGCATGGGCTCGTTCGACGTGACGGCGTCAACATCTGCCATGCAGCATCTGTATCTACCGCAGCAGCATCAGCACGGGGACATCACTGATAATGGGTTTGGAAGCGATGAGGTGGCCTCGTGGGTCTCTGAGGGGATGCCGCCGACAACCTCGTCGGTGGCGTCGATATTCGCCGGAACTTCAGATTCCATGATGTCTTTTAGAGATCAAGCGGTGTACGACACGATGAGACAAGATGCATGTGTGGATCAGACAGTGGTCCCGGAGATGGGGATGTGCCATGTGGACCAGCAGAACCAGAGTGACGACTGGCAGGCGTACACGTCGGCGGAGTTCCTCAACGCGCTAATCCCGCCCACGCCGTTCCCTCTCGACGACGAGGATACGATGGGCCCGATGTTGGCGTCATCACCGCTATTGATGCCCGGCATCCATGACCAGCAGCCGCCGGTGGAGGATATGGCGACGGCGGGCTGCTCGCAAGCGCCGGCTAACGATGGCAACGGGTTATACGCAGCTGAGGACATTGCTCCGGTTAACGTTGGTTAA

>LOC_Os04g31804.1 Oryza sativa subsp. japonica|M-type_MADS|M-type_MADS family protein

ATGGGGAGGCGGGGGCGGGTGGTGCTGCGGCGGATCGAGGACCGGGTGCGGCGCGGGATCTGCTTCCGGAAGAGGCTCGCCGGGCTGGAGAAGAAGGTGGAGGAGCTCGCCGTGCTCTGCGACGCCCACGTCGGCTTCGTCGTCCTCTCCTGCTCCGGCGACGACGCCAACCCCCACCACTTCGCCGCGCCCGCCACTATTGAAAACATTGTGGAACGTTATGAGCACTCTCAAGCAGCACAAAAAGGAGTACATGGCAGGTGCATTCTGCAAAAGAGGAAGAGCAAGGATTTTCAAGTTCTCAAGGAAACAATAGATAAAGGACCAATCAATGATGACATGCGACCTATTGATGAAAAAGACATATCCACATTAAACATGGACCAGATTAGCCAAATTGAAATATTATTGGAAGATGAACTGAGGTGGACAAGGGCAAGAAAGGTGGTGGCAGATAGAATTGCCAGGTTGCAGAAAAAGGTACAGAAGAAACCGGCGACTGCTGAGACAGAGAGCAACTCCACTGAGATGCCCTCTGATCATGAGAAGAAACAGGTAGCAGGAGGAAGCCAGCAGAGCGCCGAGGAGGAGGAGGAGGAGATGGAGGTGGTGCTCAGGCACCGCCTGAGCCTGGGCACCGGCGACCGTGACGATGGTGGCGGTGGCGCGGCGGAGCAACGGCACCGGACGACGCCGCCGCCGGCGGTCGACCTCAACGTGCCGTGCCGGGACGCGGGTCAGCTGCAGTAG

>LOC_Os01g69850.1 Oryza sativa subsp. japonica|M-type_MADS|M-type_MADS family protein

ATGCCCCCCCCCCCCCCCCCCCACTCCATCGATCCCCTCCTCCTCCTCCCCCACTTCTCCCCACCACCACACCACCACACACACGCACGCACGCACGCACGCCTCCGTTTTCCCCCACCGACGAGAGGCGGAGAGGGAGGGGAGCTAGGGTTTTTCGGGGCGGGGATGGCGCGGAGGGGGAGAGTGCAGCTGAGGCGGATCGAGGACAAGGCGAGCCGGCAGGTGCGGTTCTCCAAGAGGAGGGCGGGGCTGTTCAAGAAGGCGTTCGAGCTCGCCCTGCTCTGCGACGTGGAGGTGGCGCTCCTCGTCTTCTCCCCCGTCGGCAAGCTCTACGAGTACTCCTCCTCCAGCATTGAAGGTACCTATGATCGCTATCAGCAATTCGCTGGAGCCAGGAGAGACCTGAACGAAGGAAGTACAAGCATCAACAGTGATGAAAATGCAAGTATACACTCCAGGCTTAGGGACATAACGGCCTGGTCTCTCCAAAACAATGCTGACGAGTCGGATGCTAATCAGCTAGAGAAACTGGAGAAACTGCTGACAAATGCTTTGAGGGATACGAAATCAAAGAAGATGTTGGCAAAACAAAATGGTGAAGGGAGTAGGAGCAGAGCAAACTCCAGTGGCTCTAGGGGGCAGGAGGAAGGAAGTGCATGA

>LOC_Os05g11380.1 Oryza sativa subsp. japonica|MIKC_MADS|MIKC_MADS family protein

ATGCACATATACAAAGAGCAGGAGGCTGAACCATCCACTGGCCTGATGATGCCAGAACCAGCACCTGCTGCTTCCCCCGGCTCCGGCAGCTCAGAGGGCTCAAGCATCGAGGACACAGCGGACCGTCAGGTCACCTTCTGCAAGCGCTGCAACGGGCTACTCAAGAAGGCGTATGAGCTCTCCATGCTCTGCGATGCCGAGGTTGCCCTCATCGTCTTCTCCAGCCGCGGCCGCCTCTACGAGTACTCCAACAACAGCGTGGAGGAAACTATTGAGAGGTACAAGAAAGCCAACAGTGACACCTCCAATACCAGTACAGTTGCAGAGATCAATGCCCAGCACTACCAGCAGGAGGCTGCTAAGCTGAAGCAACATATCACCTACCTGCAGAACTCCAACAGGTTCATTATTTCACTCAGCTGCTTAGATTTTTTTTTTTCAACTTGA

>LOC_Os11g43740.1 Oryza sativa subsp. japonica|M-type_MADS|M-type_MADS family protein

ATGGGGAGGGTCAAGCTCAAGATAAAGAAGCTGGAGAACAGCAGCGGCCGACATGTCACGTACTCGAAACGGAGGTCCGGGATCCTCAAGAAAGCCAAGGAGCTCTCCATTTTGTGCGACATCCCTCTCATCCTGCTGATGTTTTCACCCAACGACAAGCCCACGATTTGCGTTGGCGATCACAGCAGCATAGAGGATGTCATAACAAAGTATGCACAGCAAACTCCTCAGGAAAGGGCTAAAAGGAAGTTGGAGAGCTTAGAAGCCCTAAAGAAGACATTCAAGAAACTAGATCATGATGTCAATATTCAGGACTTTTTAGGCTCAGGGGGTCAGACTGTTGAGATGCTGATTAAGTCAATTGTTTGGCAGGAATTATCGAGCCATCTTGGTGCTTTGCAATGTCAAATGGCAGATGTCGAAAAGCGCCTCAGTTATTGGAGCGATCCCGAGAAGGTTGAGAATATTGACCATATAAGAGCGATGGAGCAATCTCTCAAAGAATCTCTGAATCGCATCCGGATTCATAAGGAGAACTTTGCAAAGCAGCATCTGATGAGCCTACAGTGTGCAGCTGCTCAGTTCCAGAACGACATGAAGCTCCCACTAGGACTAACAGGTGACCCGAACACTTCATCGTGGTTCCATGGCGGCGGCGGCGCAGAGGCGCAGCAGCCAATGATGCTACCTGAAGACCCCAGCTTGCTCCATCAGAGAGACATCGGGTGCTCAGCGAGCACGTCGTTGCAGAGCTACCCGGGCTACTTCAGCATGGGCAAGCAGTCCACCGACAACGCCGGCGGCGGCGAGCAGCACCACCACGCGGCGGTGCAGCAGCAGCCGGAGTTCAGCCAGGCCGACTGCCTGACGTCGCTGCAGCTCGGCGCGCAGTTCCCGTACCCGTCGGCGTTCGACAACGCCGGGCTGCTCAGCGACCGGCTGTTCGACAACGCGGCGGCGGCGGCGGCGGCGATGGACTTCGGCGGCCACTACGACCTGCCGAGGCCCGGCGACGAGGCCAGCTTCCAGAACTGGGCGTCGGCGGCCTGCGGCGCCACCATGTATGATCATCAGCAGCAGCAGCAGCAACAGCAGCAGCCTGCCCAACTCCCTGCAGCAGCAACTGTAGAAGCACCTTCATTCAATCATCCCTCACCACACCGGCAGCTCATGATTTGA

>LOC_Os08g41950.1 Oryza sativa subsp. japonica|MIKC_MADS|MIKC_MADS family protein

ATGGCAGAGAAAAAGAAGAAGAAGAAGAAGAAGAAGCCGCAATCACTCCTAGTCCTTACAAGCTGGAGATCGATCGGGATGGGGAGGGGTCGGGTGGAGCTGAAGAGGATCGAGAACAAGATCAACCGGCAGGTGACGTTCGCCAAGCGCAGGAATGGCCTGCTCAAGAAGGCGTACGAGCTCTCCGTCCTCTGCGACGCCGAGGTCGCCCTCATCATCTTCTCCAACCGCGGCAAGCTCTACGAGTTCTGCAGCACCCAGAGCATGACTAAAACGCTTGAGAAGTATCAGAAATGCAGTTACGCAGGACCCGAAACAGCTGTCCAAAATAGAGAAAGTGAGCAATTGAAAGCTAGCCGCAATGAATACCTCAAACTGAAGGCAAGGGTTGAAAATTTACAACGGACTCAAAGGCAATACTACAAATCTAAACATAGGCTGTGTTTAGTTCGGTCCAAAGTTTGGAATTTGGTTAAAATTAGAGACGATGTGACTGAAAAGTTGTGTATGTATGAAAGAAATTTGCTGGGTGAAGATCTTGATTCATTAGGCATAAAAGAGCTCGAGAGCCTAGAGAAGCAGCTTGATTCATCCCTGAAGCACGTCAGAACTACAAGGACAAAACATCTGGTTGACCAACTGACGGAGCTTCAGAGAAAGGAACAAATGGTTTCTGAAGCAAATAGATGCCTTAGGAGAAAACTGGAGGAAAGCAACCATGTTCGCGGGCAGCAAGTGTGGGAGCAGGGCTGCAACTTAATTGGCTATGAACGTCAGCCTGAAGTGCAGCAGCCTCTTCACGGCGGCAATGGGTTCTTCCATCCACTTGATGCTGCTGGTGAACCCACCCTTCAGATTGGGTACCCTGCAGAGCATCATGAGGCGATGAACAGTGCGTGCATGAACACCTACATGCCCCCATGGCTACCATGA

>LOC_Os05g23780.1 Oryza sativa subsp. japonica|M-type_MADS|M-type_MADS family protein

ATGGTGAAGGGAAAGGGAAGGGCTGGCAGGAAAAAGGTTGAGATCAAGCGCATCGAAAAAAAGGATGCACGTGACGTTTGCTTCTCGAAGCGTCGCCAAACCCTATTTAATAAAGCTGGTGAGCTTTCATTGCTATGTAATGCAAATATAGCTGCTGTTGTGATTTCTCCTGCTGGTAGGGGTTTCTCCTTTGCCCATCCCTCTGTTGATGATGTTGCTGACCGCTTGGCCTCCATGGCCATGGGCATTCCCAATAACCATTCCTTGGGTGGTGGGTACCATGATAGCGGTGAGGTGACAAACATAGCGCAACAGCAGAAAATTGAATATGTGGAGCTCCAAAAGTCATTGGAAAAGTCGGAAAAGAAGAAGAGGGTTCAGGAGGCAATGGAGAAGGAGAGGGCAGGACATCTTATGCAATCACTGACTTCTGAAGTTAATCTCCTGGGGCAGGATGAATTGGAGGAGCTGCACAACAAACTCTCAGCTCTACCTTACACTTCCATTGCAAAGTTCTACCAGGTACTGCAAGATGCAAAGGGTACTAGAATGCCATTGCCACAGCCACATATTGAAATAGCATGTCAATCACAATTTCTATTTGAAGAGCAGGCTGTCACACCCGCAAATGCTGATTTTCCGGGATCTAGCACATGA

>LOC_Os06g22760.1 Oryza sativa subsp. japonica|M-type_MADS|M-type_MADS family protein

ATGACAAAGCGGAAGATTGAGATCAAGCGCATCAAGAATGAGGAAGCACGCCAAGTGTGTTTCTCTAAGCGTCGTCCTAGTGTGTTCAAGAAGGCCAGCGAGCTCTATACCGTCTGTGGTGCAGAGGTTGCTATGCTTGTCAAATCTCCTGCTGGTAAATTTTTCTCCTTTGGTGCCCCCTCTGTCGGATTTGTTCTTAGCCGCTTCCATGCTACTACTACTTCTAGAAAACACTCCAGTATGGGTGTTACGATCCAACATGATAATAGTGCAACAATCAAACTACATGAGTTGAATCAACAACACATAGAGTTGCAAAACCAACTGCAAGCTCAAAATGAGAAAATGAAAGCGCTACAAGAGGTTGCTAAGAAGGAAAGTGGGGGAAAAGTGATGGGTTGGTTGAATAGCAAGGTTGAAGATATATGCCAAGAAGATTTGGAAGAGTTCAAGATGGTGCTTGAGTCTCTAAAGTACTTGACGAGGGGGATAATTAATCAACTCTTTCAGAATTATGCTATGTTCTCTAATATGATGCGTGTGCAGCATTGTGTGACCGCTTTGCCCAATCAACAATTCCTTCCGAGCAGTGAAGATGTCAAACCGATGATTCACCATGTTCCTAGTTCAAGTTATGGATGGAACACAAGCATAGATAGCAAACCTAATTCAAGTGATGCTCATGTTGTTGGGGCCAGGAGGTACTTTCCGAAGTGA

>LOC_Os03g14850.1 Oryza sativa subsp. japonica|M-type_MADS|M-type_MADS family protein

ATGGTGAAGTGTAGGGCACGCACGACTAGGAAAAAGATTGAGATCAAGCGCGGGGACAAGAAGGTACGAGATGCATGCTTTTCCAAGCGCCACACTACCATCTTCAACAAGGCCAATGAGCTCGCTATCCTCTGTGGCGTGATGGTTGCTGTTGTTTTTGTCTCTCCCAATGCCAATGGCGGGATTTTCTCATTTGGCTACCCATCTGTCAGCTCTGTGGCAAATCGCTTCCTCGCCAATGCTCCAAACAACACCAGTGTGAGTAGCTCAACCCAAAGCGGGAGGGATGTGGAAATCCGTGAACTAGAACGTGAAGAAAGAGAGTTGAAAGAGCATCTACAAGCTTCCACAGATCAGAATAAGCTTCTGCGGGAGGCAATAGCAGCAAGGGATGGAGGGCAATTGATGCTTCTCCTGCAGAGTGATTGTAGTGAGTTGGGACCAAAAGGTCTGGTGGCAGTTGTGCGGTTGTGGTTCCGCAATTCTCTTGTTGGCATTGGGCTTGATCGGTTGTGCTGGCTGCTGCAGCAAATACTCTTTGGTTTGGATTGGGGCTGA

>LOC_Os12g21850.1 Oryza sativa subsp. japonica|M-type_MADS|M-type_MADS family protein

ATGGTGCATCCGCTGGGGAGGACGAGTATGGGGCGGCAGCGCATCGAGATCCGCCGCATCGACAACAAGGAAAGGCGGCAGGTCACCTTCACCAAGCGGAGGGGCGGGCTGTTCAAGAAGGCGTCGGAGCTCGCCCTGCTCACCGGCGCGAGCGTCGCCGTCGTTGTCTTCTCCCCTGCCAAGCACGTCTACGCCTTCGGCCACCCGTCCGTCGACGCCGTTCTCCGCTCATACGCCTCCGTCCCCGGGGAGGCCGCCGCGGTCGCACCCGTCCCCGTCCACGGCGGCAGCGGCGGCGAGGACGTCGACCTCCTCGGGCTGAGGCTGGCGGCAGATGATACCGGGGCGCAGGTCGCGGCGGAGCATGCGCGGATGCGCGACGTCGCGGCGAGGATCGTGCAGGCGAAGGCCGGGAGGCGGTTCTGGTGGGAGGCCGACGTGGACGCGCTCGGGGAGGCCGAGTTGCTGGAGTTCTTCACGGCGCTCAAGAAGCTCAGGGACAACGTCGGCCGCCACGCCAACGCCCTGCTCGCCCCCCAGCCGCCGCCGCTTCCGCTGCAGCAGAAGCAGCGGCGCCGGCGGTAG

>LOC_Os12g21880.1 Oryza sativa subsp. japonica|M-type_MADS|M-type_MADS family protein

ATGCGTCCGCTCGGGAGGACTAGTAAGGGGAGGCAGCACATCGACAACAAGGAAAGGCGGCAGGTCACCTTCACCAAGCGGCGGGGCGGGCTGTTCAAGAAGGCGTCGGAGCTCGCCCTGCTCGCCGGCGCGAGCATCGCCGTCGTCGTCTTCTCCGAGACCAACCTTGCCTACGCCTTCGGTGACCCATCCGTCGACGCCGTCCTCCTCTCCTACGGCCCCGTCCCCGGGGAGGATGCTGAGCCCGCACCCGTCCACAGCGGCGGGTTAGGCAAGGACGTCGACCTCGAGATGCTGAGGCATAACAATTGTGCTTCTTCTCTGCAATTTGTTCTCGTCTCGCTTTCTTTCATCATGTAA

>LOC_Os06g30810.1 Oryza sativa subsp. japonica|M-type_MADS|M-type_MADS family protein

ATGGTGAGGTCGCGTGGGAGGCCGAGCCTGGGGAGGCAGAGGATCGAGATCCGCCGCATCGACAACAGCGGGCGGCGGCAGGTGACCTTCTCCAAGCGCCGCAACGGGCTCTTCAAGAAGGCGTCCGAGCTGTCCACGCTCTGCGGCGCCTCCGTCGCCGTCGTCGCCTTCTCCTCCGCCGGCAACGTCTTCGCCTTCGGTCAACCCACCGTCGACGCCGTCGTCCGCCGCTTCGACCCGCTCCACGCCGACGGCGCCGACCCCGCCCCCGCGGCCGTCGAAGACGGCGGCGGCGGCGGCGACGACGTCGTCGTCGCAGACCCCGAGGAGCTGGATGCGTTGAGGCGCGCGGAGGAGCAGACCAAGGCGCAGGTGGCGGCGGAGCAGGCGCGGATGCGCGACGTCGGGGACAAGGTCACGCAGGCCATGGCGGGGAGGGCCCTCTGGTGGGAGGCGGACGTCGAGGCGCTCGGGGAGGCGGAGCTGCCGGAGTTCGTCAGGGCGCTCGAGCGGCTCAGGGACAGCGTCCACCGCCACGCCAGCACGCTGGCCTCCACAGCCACGCCGCTGCCGCCGCCGCCGGAGCAGGAAGAAGAAGTTCCTGAGCTAGACGTCTCGGATTATTCCTTTTAG

>LOC_Os06g30830.1 Oryza sativa subsp. japonica|M-type_MADS|M-type_MADS family protein

ATGGGTCGGCCGCGAGGGGGGACTAGCAAGGGGAAGCAGAAGATCGAGATGTGCTGCATCGACGGCAAGGAGAAACGGCAGGTCACCTTCTCCAAGCGCCGCCGGGGCCTCTTCAAGAAGGCGTCCGAGATATCCACGCTCTCCGGCGCGTCCATCGCCATCGTCTCCTTCTCCAAGGCCGGCAACGTCTTCGCCTTCGGCTCGCCGAGCGTCGACGCCGTCCTCCGCCGCCACGTCGTGGCCGGGCCTTCCACCTCCACCTCCCACGCCCACGCCGGCGGCGACGTCTTCGCGGACGACGGCGGCGACAACCCCGAGGTGCTGAACGCGCTGAAGCGGGCGACGGACGAGGCCGCGGCGGAGGTGGCGGCGGAGGACGCGCGGCAGAGCGGCGTCGAGGGAAAGATCACGGAGGCCATGGCGGCGGGGAGGCGGCGGTTCTGGTGGGACGCCGCCAACGTCGAGGCGCTCGGGGAGGCGGAGCTGCCGGTGTTCGAAAGGGCGCTCCATAAGCTCAGGGGCGCCGTCGCACAGGGAGGCAACAATCCAATCCAGGAGAGATGGGATAGGATCCCAGCTCAAGGGAGCCCTGGTGGCAGCTACGGTTGGGAGGACCTAAGCCCTACAGGCAAGCTCTATAATCAACCCCCTTTTGCTTACCGGCTTCCAGTGATGCAGTCAATAGTTTCTGATCAGCTTGGAGTGGTCTGTGCCATGTTGCTGTGA

>LOC_Os09g02780.1 Oryza sativa subsp. japonica|M-type_MADS|M-type_MADS family protein

ATGGAGAGCGAGGAGGCACGGAAGGTGTGCTTCTCCAAGCGCCGTGCGGACCTCTTCAAGATGGCGAGCGAGCTGTCCGTTCATTTCAACGCCGACGTGGCGGCTGTCGTCTTCTCCCCCGCCGGCAACAGGGCCTACTCCATCGGCGACCCGTCGGTCATGGACCGCTTCCTGTCGTCGTTGCCGGCGCCGGCGCCGCCGGCGGAGACCGAGCCAGAGCCAGAGGTGGATTGGTCGGTGATGGAGGAGCTGAGCAGGCTGTGTGGCCAGCTGCAGGCCATGGTGGACGCGCACAAGGCGCGCCTGGAGAAGGCGGAGGAGAAGCTTCGCGAGAGCGGCGCGGCGGCGTGGATGATGGATCTGGAGGCGGAGGTGGGTCGCATGGCGCCGGAGGATGTGCTGGCCCTGGTGACTAAGCTGGCGGTGCTGCGGGACGGCGTGGCGGAGCGCGCCCACGAGATGCTGCGCGAGGCGCTGCTCGCTGTCGCTGCCCCGACCCCGACCACCCCCACCACCCCGCCGCCCGCGGGCTTCTGA

>LOC_Os09g02830.1 Oryza sativa subsp. japonica|M-type_MADS|M-type_MADS family protein

ATGGTGACGGCGGCAGCGGCGAGGAGGCGGCCGAGCCTGGGGCGGCAGAAGATCGAGATCCGGCGCATCGAGAGCGAGGAGGCACGGCAGGTGTGCTTCTCCAAGCGCCGTGCGGGCTTCTTCAAGAAGGCCAGCGAGCTGTCCATCCTGTGCAGCGCCGACGTGGCGGCCGTCGTCTTCTCCCCCGCCGGCAAGGCCTACTCCTTCGGCCACCCGTCGGTGGAGTTCCTCCTGGACCGCTTCCTCTCGTCGTCGTTGCCCGCGACGGCCGGGAAGGAGGAGGGGTCGTCGGTCTCGGTGGTGGCGGAGCTCAACAGGCAGTACGGCGAGCTGCGCGCGATGGTGGACGCCCACAAGGCGCGCCGGGAGAGGGCGGAGAAGACGATGGAGAAGCAGCGGCAGCGGCAGCCCGCGGCGTGGATGGATCCGGAGGCGGAGGTGGGTCGCATGGCGCCGGAGGAGCTGATGGCCTTAGGGACGAAGCTGGTGGCCGTGCAAGGTGGCGTGGCGGCGCGCGCGGACCAGATGCTGCGTGACGCGCTGCTTCTTGGCCGGAGGCCGAACACCACCACCACCACCACCACCAGGGCGCCGCCCGGCTTCTTCCACCTGCACCCGCACTTCTGA

>LOC_Os01g74440.1 Oryza sativa subsp. japonica|M-type_MADS|M-type_MADS family protein

ATGGCGATGCAGGTGGCGGCGCCGTCGCGGAGGAGGCCGAGCTTGGGGCGGCAGAAGATCGAGATCCGGCGCATCGAGAGCGAGGAGGCACGTCAGGTGTGCTTCTCCAAGCGCCGTGCCGGCTTCTTCAAGAAGGCCAGCGAGCTGTCCATTCTGTGCAGCGCCGACGTGGCCGCCGTCGTCTTCTCCCCCGCCGGCAAGGCCTACTCCTTCGGCCACCCCTCCGTGGAGTGCCTCCTGGAGCGATTCCTGCCGGACTCGTCGTCGGGAGCGGCGGCGCGGGTGCGGCGAGGAGCGAATAATAATGGTGGTGGTGGGATGGTCGGGGAGCTCAACAGGCAGTACGGCGAGCTGCGCGCGATGGTGGAGGCGCACAAGGCGCGGCGGGAGAGGGCGGACGAGAAGATAGAGATGGAGCGCGCGGCGGGGAGGTGGCTGCCCATGGACGCCGATGTGCGCCGCATGTCGCCGGAGGAGCTCATGGCTTTCGGGACGGGCCTCATGGCTGTGCAGGCTGCCGTCTCCGCGCGCGCCGACCAGATGCTGCGCGACGCGCTCCTCATTGGCCGCAGGCCGCCCACCACCACCACCGCCGGCTTCGGCTTCTTCCACATGCCACACTACTGA

>LOC_Os09g32948.1 Oryza sativa subsp. japonica|MIKC_MADS|MIKC_MADS family protein

ATGGGGAGAGGGAGGGTGGAGCTGAAGAGGATCGAGAACAAGATCAACAGGCAGGTGACGTTCGCGAAGCGGAGGAATGGGCTGCTCAAGAAGGCGTACGAGCTCTCCGTGCTCTGCGACGCCGAGGTCGCCCTCATCATCTTCTCCAACCGCGGCAAGCTCTACGAGTTCTGCAGCGGCCAAAGCATGACCAGAACTTTGGAAAGATACCAAAAATTCAGTTATGGTGGGCCAGATACTGCAATACAGAACAAGGAAAATGAGTTAGTGCAAAGCAGCCGCAATGAGTACCTCAAACTGAAGGCACGGGTGGAAAATTTACAGAGGACCCAAAGGAATCTTCTTGGTGAAGATCTTGGGACACTTGGCATAAAAGAGCTAGAGCAGCTTGAGAAACAACTTGATTCATCCTTGAGGCACATTAGATCCACAAGGACACAGCATATGCTTGATCAGCTCACTGATCTCCAGAGGAGGGAACAAATGTTGTGTGAAGCAAATAAGTGCCTCAGAAGAAAACTGGAGGAGAGCAACCAGTTGCATGGACAAGTGTGGGAGCACGGCGCCACCCTACTCGGCTACGAGCGGCAGTCGCCTCATGCCGTCCAGCAGGTGCCACCGCACGGTGGCAACGGATTCTTCCATTCCCTGGAAGCTGCCGCCGAGCCCACCTTGCAGATCGGGTTTACTCCAGAGCAGATGAACAACTCATGCGTGACTGCCTTCATGCCGACATGGCTACCCTGA

>LOC_Os02g06860.1 Oryza sativa subsp. japonica|M-type_MADS|M-type_MADS family protein

ATGGCGAAGGCGGCGGCGGAGATGGACGTCGGCGGCGGCGAGGGGAGGAGGTGGAAGCGGACGCGGGGGCGGCAGAGGATCGAGATGAAGCTGATCGAGAACAAGGAGGCGCGGCAGGTGTGCTTCTCCAAGCGCCGCGAGGGAGTCTTCAAGAAGGCCAGCGAGCTGTCCGTGCTCTGCGGCGCGCGCGTCGCCGTCGTCTTCTTCTCCCCCGCCGGGAGGCCGCACTGCTTCGGCCACCCCTCCGTCTCCGCCGTCGCCGACCGCTTCCTCCTCGGCCGCTCCCCCGCCGACGCCGCGGCGGCGGCGGCGGAGGAGGAGGAGGCGGTCGTGCGCGAGTTCAACCGCGTGGAGGAGCGCCTGAAGGACGCGCTCGGCGCGGCGGCGCGCAGGCGGGACGCGCTCGACGAGGCGGCGCGCGTGGCGGGCGTCTGGAACGACGCCGACGTGCGCCGCGCGGGGCTCGCCGACCTCGTGTCCATGCTCGCCGCGCTCCAGAGGGTCCAGGCCGAGGCGTCGGAGCGCGTCCACGACATCATCGTCGAGGAGGCGATGACACACTACACGGGCGCCGCCGCCGCCGCCGCCAACCTCATCGACTACCTCGACGCCGGCCCGTTAGTCTCTCACAGCCCCGGGAGCCACGACACCACGACGAAGTTGATCGGCGGCAACGCTGTCCATGCGCCGCCGTTGTCCTTCCCGCCGATGATAATGCCGCCGCCTCTCCCCCCGCAGTTCAGCCATGGATTTGGGTACACTGACCTCGCCGCCGGCTACGGCTACAACCTTGACCACGGCCATGGCGCCGCCTATGAAACGGAAGAATTTCACAACGCGGCAGCATGCGACTTCTTTTAA

>LOC_Os04g25870.1 Oryza sativa subsp. japonica|M-type_MADS|M-type_MADS family protein

ATGGCTCTTAGGAAGATGAAGCTTCAGCGGATTGTCATTGATGTGAAGCGGCAGGTGACATTCAAGAAGCGTCTCAATGGCTTGACGAAGAAGGTTAGTGAGTTCGCAACACTATTCCTCATGGTGTACGGTGAGGTTGAAGTGCAAGCAACGAAGGTTTGGCCGTTGGTGTGGAAGGCGACCAGAGTTCTCGAACACTTCAAGGCCATGCCGCAGCTAGACAGATACAAGAAGATGACTGACCTAGAGGACATCCTCAACGAGCAGGTCGACAAGCTCAAAGAGGAGTTGCACAAGGTGGGACGCGATGCTGATGAAAGCGACACCAAGCTAATCCTCGTTGAAGCCCTCAATGGCCACCGCCCGAGCCTCGAGGGGCTCACGATCGAGCAGATTACCAGCCTCGGATTGATGGCAAATGCACACCTCAAGATTGTCAACGACCGCCTCAAGAAGCTCCGTGAGCAAGGCCTCATACCAGCATCCTTGTTATTGTCTGGCACGGAGGTTCCAATCCAGAGGGAGGGATGGCTGATGGATGTTGCAAGGGGCATTGGCTCAATGGGGAACAATCGGTTCGGAGGTACCAGCGGAAGCGGCATTGCTGGATCCAATGGTGACATGGCTTAG

>LOC_Os03g37670.1 Oryza sativa subsp. japonica|M-type_MADS|M-type_MADS family protein

ATGTTCAAGAAGCGGCAAAAGAGCCTAATGAAGAAGGCAAGCGAGCTCTCGACGCTATATGGCGTGGATGCATGCGTTGTGATGTATGCTGAAGGCGAGGCGCAACCTATGATGGTGTGGCCATCGGTACCAGAGGCAAGGCGTGTGATCGAGCGCTTCAGGGCCTTGCCGCAGAAGGACCAATACGAGAACACGACTAACCTGGAGGGCTTCCTCAAGCAGCGCATCGCCAATCTCCAAGAGAAGGTGGATAAGGCAAAGCATGAGAACGATGAGCTCGAGACAAAGCTCCTCCTCCTCAACAGCCTTGACTGCTGCCTCCCTAGCCTTGTGGGCCTCACGGTCAAGCAGATTACTAGCCTCAACTCGATGGTAGAGGAACGCCTCAAGAAGCTCCGTGGGAATGGTCTCCTGGCGACACCAGTCCCAACCAGCTCGAAGAAGAAAATTATATGGGATGTCCGGTACAACCTCAAAGCGAGGTATGTCACGGATGATTCTGCCAAGAATGGTAGCCCTAGGTTGCGGTGTACCCCGGCAACCTCCTCCCAACGCCAGAGCTCGCCGGAATTCCGACATCGCCGTTGCCTCCCCTAG

>LOC_Os03g38610.1 Oryza sativa subsp. japonica|M-type_MADS|M-type_MADS family protein

ATGGCTCGCAACAAGGTGAAGCTGCAGCGGATCATCAATGATGCGAAGCGGCGGGCAACATTCAAGAAGCGGCTCAAGGGCTTGATGAAGAAGGCAAGCGAGCTGGCAACACTATGCAATGTGGACACATGCCTCATGGTGTATGGTGAGGGTGAGGCGCAAGCAACGGTGGTTTGGCCGTCGGAGTCGGAGGTGATGAGAGTTCTCGAGCGCTTCAAGACCTTGCCACAGCTGGACAAATATAAGAAGATGACCGACCTGGAGGGCTTCATCCAGGAGCGCATCAACAAGTTCCAAGAGCAGTTAGACAAGGTGAGGCGTGATGCCGATGAAAGTGAGACCAAGCTACTCCTCATTGAAGCTCTCGAAGGCCGCCGCCCAGGCCTTGAGGGGATCACCATTGAGCAACTTACCAGCCTTGGCTGGCTTGTAGATGCACGCCTCAACATCGTCAATGATCAACTCCAGAAGCTCCACGAGCAGGGCCTCCTACCAGCGTCTATTTCACTGCCAACCATGGGGGTACTGCCTTACACCACGGCGGGCTACACTGTTGCGCAAGAGGCACCAATCCAGAGGGGGGGATGGCTCATGGGTGTTGTAAGGGGCATTGGCTCATTGGGCTATAGTTTGTTCAGAGGTAGCGGCAGAAGCAACACTGCTGGACCCAGTGGTGACATGGTGCAACCTTTCAACATTGGCGCAGGATCCTCATTGGCCAACCAGGGTATTTCATTCCCTCCCAAGTAA

>LOC_Os01g18420.1 Oryza sativa subsp. japonica|M-type_MADS|M-type_MADS family protein

ATGGCTCGCAATAGGATCATCCTCAAGAAGGTCGCTAAGGATTCCACTCGGCGCCTCACATTCAAGAAGCGGCGCAGGGGCTTGATCAAGAAGGCCGGAGAGCTGGCCTCGTTGTGTGGTATTGGCGTGTGTGTGGTGGTGTATGGAGAGGGCGAGGTGAAGCCAGAGGTTTGGCCTTCTGCCCCCGAGGCACGAGCAATCCTCTCCCGCTTCAACTCGGCGCCGAACATTGATAGGTTCAAGAGGGTGACGAACCAGGAGCAGTACCTCCGAAAGCGCATCGCCAAGGCTCGGGAGCGCACGAGCAAGGCGGACGATGTCAACCGTGAGCGTGATGCTACCATCATGCTTTATGAGGCCGCCACCGATAAGCGCCCAGTGGCAGACCTCAATGTCCAGGAGCTCACCAACCTCGGATTGGTGATCAATGAGCGCATCAACCACCTGAAGGAACGCATTGAGCGCCTTGGAGGTGCGGCCCTCATGGCGCCACCGCCATCGACGCAACCGACGGAGGCATCATCATCCTTGCCACCGCTAGTGCCATACGCCAATGGTGCTGGCATGGAAGGGAACAAGAGGATGAAGGTGAGCACGCATCAGAAAGGCTGGTTCATTAACATGAGCACCATGACAGGTGACGCAGGGACCTCTGCCGACGTTGAAGGCAATACTGGTGTTGGCACCAGCGCTAGGGGTGACATGATGCATCTTTCCAACTAG

>LOC_Os01g18440.1 Oryza sativa subsp. japonica|M-type_MADS|M-type_MADS family protein

ATGGCTCGCAAGAAGATCGTCCTGGACCGCATCGCCAACGACGCGACGCGGCGGGCGACGTTCAAGAAGCGGCGGCGCGGGCTGCTGAAGAAGGCGAGCGAGCTGGCGACGCTGTGCGACGTGGACGCGTGCCTGGTGGTGTACGGGGAGGGGGACGCGGAGCCAGAGGTGTGGCCGTCGACGGAGGTGGCCATGAACGTGCTGCGGCAGTTCAGGGCGCTGCCGGAGATGGAGCAGTGCAAGAAGATGATGAACCAGGAGGACTTCCTCCGCCTCCGGATCGGCAAGCTCAAGGAGCAGCTGCGCAAGATGGACCGCGACAACCACGAGCGGGAGACGCTCATCCTCCTCCACGACGCCCTCCAGGGACGCCTCGGCACCTACGAGTCCCTCTCCGTCGAGCAGCTCACCAGCGTCGACTGCCTCGCCAGCGCCAGGCTCAAGGTCATCACCGACCGCCTCGTCGAGATCCGCGCCCCCAACGAGGACGGCCAGGTGCTCGTTCCCCCACCTCCTCCTCCCCCGCCGGCGCTCCCCGCTCCTCCTCCGCCTCCGGCGCCAATGCTTCCTCTCGCGCCGCCGCCGACTCATGTTACTCCGGCCATGCCGCTGTCTTCTATGCCGCCGCCGGCGTTCCACGGGATGAACCACCACCACCACCAGAACCACTTCATCAACCATGGCGGCAACGACCAGAACGCCTGGCTCATGAACGTCGCCAGGAACGGCGGCGACCTCGGCGCCCTCGTCTACAGCGCCTTCGCCAGCAGCTCCTCCTCCAACACCGGTGGCGCCGGCACCAGCGCCGCCGGCGCCGCCGCCCCGGGCCCTGACATGATGGATCTCGCCAACCCGGACATGCCGGGATTCGGGTGTCCCTGGGACGACGATTCTGCGGGGCCATCTTTCCCTCCCATGTAA

>LOC_Os07g04170.1 Oryza sativa subsp. japonica|M-type_MADS|M-type_MADS family protein

ATGGAGAGGGAATTCTGTGGAGAGATGGAGAGCCAAATTTGCCAATTTGGTACCTTTCTAGAAGTCAACAATCAGCATTCAGCAACTCCAAAAGGAAAAACCATTCCAGTTAAATGCACAGACTGCCTCCCAGGAGTGGCTTCCCGCTCGCTCACCGTAATCCCACTTGAAGTTCCACACGACGTTCTTCTTTCTGCAAGGGAAGCCAAAACTCAATACAATAGCACATTTGAACACAATCTCAGTGAGACCAACTTCGTAACTGGTACTTACAATGGAGCTCACCATGCCAATGTGAAGGATGTACCACCTGACATTTATGAGACAGAATTTAGGCATCAATTTCCAAGGATTGAAACTTCTTTTTCAATCATTGGAGCACATTTCCTGTCAAGTATGGTGAGGAGGGGTAGAAGGAAAGGCGTGAGGTACATCGAAGAGGATAGAGATCGCAGCCTCACCCTCAGCAAGAGGCGTGATGGGCTGTTCAAGCTTGCAAATGACCTCTCCCTTCTCACTGATGCAAGCGTCGCAATTTGCCTACATGACAGCAATAAGGCACAATTCTTTGGGGCACCATCGGTGAAACCTGTTGTCGATGCTTTTGTATCTGAGGCAGAGCCATTTGCCGATGAACAACTGAAGGCCAAGCTTACGTCAATGCAAAGTGAGCTGGTCCAGCTAGAGAATGAAGAAGAAGAGAAGGACAAGAAGACTGAAGAATCAATCCAGCGCTTCAAGGAGGCCCAAGAAGAGAGCTTAGGTATGGGTATGGCCAAGCATCTATTCTCCAGGCTAGAGGATCTCAGCCACGATGATATGCGTGAACTTTTAGATGTACTCTTGCCGCTCCAGCAAGATTTCAAGAAGCGGCTGCCTCCCCTACGTCGTGGCAGCAAGCTGCAAATTGGTGGATCAAGTGCGTGGGCACATCAGCAGCCCTCGTGTTCTCGCTTCTTGGCCTCACACCGTCCATTCACTCCATTACTTCCTGGAGGGACATCAGGAGTTCCGATGATACCTCCGCCTCCAGTGCCGGGCTCACCATGGTCTCAGATATTTCCGCTACGGCCACCACTGTTTCCTTCTCCAGAGTTAGTGCCATCCCAACAATTACCACCAGTATCACCGCCGCAGAACACAGTGGCACCTCCTCCAATGCATGCTCCATTGGTGCAACAGCCACTTACTAATCAATCATCGGCGGTTCCCCTGCTGACGCAATGGCAAATGCGTTTTGGGGATCAGCCACCCGCTGAAGTGCAAGCATGCACTCCCGTAGAGCAACCTCAGAATGACAATGCAGTCCATACCCCAACCTTCAGCGACAGTTTCCTTTTGGAGTTGTTGGCTGATGTCAGCGATGATGGCATTGCTACTGCTGAACCATTGTGTTCTCCTCCTATTGATGACCAGTTTTTGGCTGACATCGACTGGCTTGCTGAATTGGACACTATCGATGGAAATTTATAG

>LOC_Os01g11510.1 Oryza sativa subsp. japonica|M-type_MADS|M-type_MADS family protein

ATGCCGAGGAGGGCGAGGAGGACCGGCGCGGCGTACGTCGACGACGAGAGGGAGCGCGACATCACCTTCTTCAAGCGGCGGAACGGGCTGTTCAAGTGCGCCAGCGACCTCTCCATCCTCACCGGCGCTAGCGTCGCCGTGGTCATCGAGGACCAGAACCGCAGCAAGTTCCACGCGGTCGGGACGCCGACGGTGCAGGCCGTCGTCGACGCCGCCCTCTCAAGCGACGTGGAGGAGGCGGCGGCTGAGGCGCGGCCGGTCGCCGACGAGCAGCTGATGGAGAGGATCGCGCCGCTGGAGAGGGAGCTGGCTTGGCTGAAGGGTGAAGCTGCCGAGAAGGATGAGACGACGAAAGCATCCAAAGCTCGGTTCAAGATGGCCCAGAAGAAGGAGGAGAACGAAGAGGAGGGTGACACGAAGAAGAAGAAGCTCTTCTTCTCCAAGGCCGACAAGCTCAGCTCAGACGAGATGAATGAACTGTTAGCTGAGATGCTTGAGATCAAGAAGGAACTCAACGTGCGGCTACCCCCGCTACGTCGTCGCGGTGGCAAGCGGCCAATCCAGGGCTCTAGCGTGCCACCTCCACCGCCGCCGCCGCCACCACCGCCGCAGCCGGAGCAACAGCTGCAGCTGCCGCAATGGCCGAATCTGTCCGGTCCTCATAACCAGTTGCTTCCTGTGGCGCCGCCGCCATTTGTCGCTGATCAGCCACCTCCACCTCCACCGCCAGCAGCAGGAGGGTCTCTCTGGATCCCTGAGCTGCCTCCACCTCCGGTGGAGGGGTCGCCATGGGCTGGTCTGCTTCCGCTGCGACCACCACGGTTTGCCGGGATGGAGCCATCCTTTCTTGAATCACAGCAAGCTCCGCCTCCGGCACAGGTCAGCACACAGCTAGCACCACTACCTCTGATACGGGAGGAAGCTCCATTGCTGCAGGAGCCATTTCTGTTTGCTGATCAAGCACCAGTACTTGCTCCTCTGCCGGCGCCATTGCAGATGCCTGTGGCAGAAACTCACTTGCCACTGCAGGCTCCATTGCTGCAGGAGCCATTTCTATTCTCTGATCATGCACCGACGCTTGCTCCTCTGCCATCGCCATTGCAGATGCCTGTGGCTGAAACTCACCTGCCACTGCAAGTGCAGGCTCCGTTCATGCAAGAACCATTTCTGTTTTCTGATCAGGCACCAGTGCTTGCTCCTCCGCCGACGCCATTGCAGATGCCAGTGGAAGCTCACATGCCACTGGAGGCTCCATGGATCCAAGAACCATTTCTCATGCCTGATCAAGCACCGGTCCATGCTCCTCCACCAACGCCATTGCTGATGCCAGTGGGAGCTGATCACTTCCCACTCGAGGCTCCATTGTTCCAAGAATCACTTATAATGGCTGATCAGAAATCTGTGCATGCTCTTCCTCCACCTCCATTGCAGATGCCTTTGGAAGCTCACCTGCCACCTGCAGCACAAGAGTATAACCAAGATCTGGCCGTGCAGCAGCAACCCCAAGAGTATGAAAACTATGACTACATGTTTGAGAACGTGGGGCTCTCACAGGCACAGCCGGTGGCCGCTGGCGCCGGCGACGCAGGCTTTGCAGCTATAGGCAACGACGACAACCCGTTCGGCTACCAACAGTTGGTTGCCTCTCCTCTCTATGATGGACAGATCTACTTCGGCTCCGGGGTTGATAACATGGGCGTTCCTCCTGCTGGTGATTTTGGTGGTGTTCCTGAAGCTGCTTTGCCGGAAGTCGAGCATGCGTCTTCGTCCGGTTGGGGAAACAACATTACTGGTGATGCTGGTGCATGGTTCTGA

>LOC_Os01g23750.1 Oryza sativa subsp. japonica|M-type_MADS|M-type_MADS family protein

ATGGCCGGGAGGAAGGAGACCGTGATAAAAATGGCGAAGGAGCTCTCCGTGCTCTGCGACGTCCCCGTCGCGCTCGTCTGCGCCGTCGGCGGCGCGGTGGAGGTGTGGGAGTCGGAGGAGGGCGTCCTCGACCGGTACCGCGCCCTCCCGCCCGAGGTCCGCGCCACGCGCGCGCACACGCACCGGGGCTACCTCGAGCGGGAGCTCCGCGCGAGGAGAGCCAAGCTGGCCAAGGTCCGGGAGGAAGGCGCGTTCAAGTCGTGGGGGCGCGACGCGCTCAGCGGCATCATCACGGCGGAGGAGGCGCCGGCGCTGCTCGAGTCCATCGACGCTGCGATCGCCGCCGCGACCGCGAGGCAGGAGGCGCTTGCTCTACTGGACGGCGGCGGGCTCCATCTGCAGCACGTCCCGGCGAGCGCTTCCGACGCCGTCGCGCCTGTCGTCGGCGGCCACGGCGTCCAGGTCCAGTACATCGGCGGCAGCGGCGGCGGCGGCGGGAGCCAGCAGGAGATGACGCCGGCAGCCGACGGAGATGGCGCCCGCAACGCCGACCAGTACGACGACATCCTCCCGTGGGATGGCAACACCTTCGAGGCGCACAACGCCCACGTGATGCTGCCCGCGTGCGGCTTCCAATGCACCGGCGACTACCGCGTGGACATGGACGGCTACGTGTGGGGAGCCCCGGATGATGCCAACGCCTACCATGGCTGGCCCGATGAGGCAATGTGGTGCACCGACGAGTCGTGCTCGTGCAACGCCGCCACCGCCACCGCCGTGCCAGCCATGTATCATCCCCCCACCCTGGACACCGTCCACGGGAGCTTCCTCGCCGCGCCAGCTCAACCACTCGCCTTCAGCACCGGCGCCGACTTCATCAACGCGCCGAACGACTTTCTCACCGTGGGCGTCGGCGGCAGCTTCATCAACGTCGGCGACTACTCGGCGCAGAGCTTGGCCGACGAGTTCCACCACCTCAGCGACGCCACCAACCAGCTCGATCAGACGCACTACCCACCATTCGGTGGAACGGGCGGCGCCGAGCCCGGCGACACGCAGTCCCATAGCTGGGGGGACTACTACTTGGCGCAGAGCTCCGCCAATGAGTGCCAGCTGCTCGGCGTCGACGGCGGCGACATCCATCTCGATCAGACGCATTGCCTCGGTGGTGCGGGCGGCGCCGAGCCCGGCGACACGAAGTCCCACAATTGGGGTGGCTGA

>LOC_Os01g23760.1 Oryza sativa subsp. japonica|M-type_MADS|M-type_MADS family protein

ATGGCGCGACGAAAGATCCCGATAGGCCTCATCGCGCACCGGCAGAAGCGCGCCGCGACGTACGCGAAGAGGAAGGAGAGCCTGAGGAAGAAGGCGGAGGAGCTCTCCACGCTCTGCGGCGTCCGCGTCGCGTTCGTCTGCGCGGGCCCCGTCGTCCCCGGCGGCGGCGGCGGCGCGGCGGGGAAGGAGGAGGTGTGGGAGTCGGAGGAGGGCGTCCTCGCGGAGTACCGCGCCCTCCCGCCGGAGGCTCGCGCGCAGCACGCGCACCGGGTGTACCTCGAGGAGGAGGTCGGCAAGGAGAGGGCCAAGCTCGCCAGGGTGAGGCAGGACGGCGCGTTCCCGTCGTGGGACGCCGCGCTCGACGGCATCACGGCGGACGAGGCGCGGGCGCTGCTCGAGTCCATCGACGCGGCGAGGGCGGCCGCGAACGCGAGGCGGGAGGCGCTGGGTCTGCCGGACGACGGCAACGGCGTGGACGATGACGGCGGACTAGATCTGCAGCAGCAGCAGGAGCACGTCCCGCCGGGCGGTTCTGACGCCGTCGTCGTGCCTGTCGGCCATGGCGTCCTCCAATACACCGGCAGCGGCGGCGGCAACCAGATGCAGACGACGCCGGCCGCAGATGGCATCAACTGCGCCGACCTGTACGGCGCCGTTCCGTGGGATGACACCTTCCAGCCGCAGGTGATGCGCACCGGTGACCACTTCGTGCCCATGGACGGCTACCTGTGGCAAGCCCCGGGCAATGGCTGGCCCGATCTCGCCACGGGGTGCACCAACGAGTCGTGCTCGTGCAACGCCGCCGCCGCCGCAGCCGCCATGCCAGCCATGTATCCCCCCACCCTAGACACCGTCCACGGAAGCTTTCTCGCCGCGCCGGCTCAACCCATACCCATCGCATTCAGCACCAGCACCGACTTCATCGACGCGCCGAACGACTTCCTCACCATGGGCCTGTGTGGCGGCTTCACCAACGTCGGCGACTACTCGGCGGCGCAGCCTCAGAGCTCGGCCGATGGAGGATTCCAGCTCGGTGACACGTTCGCCGCCGAGCCCGGCGACACGCAATCCCAGAATTGGGGGAGCTTCATCAACGTCGTGAGCGACGACTCGGCGCAGTGCAACTGCAACGCCGCAATCCATCTTGATCAGATGTATTACCTATTCGGTGGCACGGGCGGCGGCGAGCCCAGCGACACGCAGTCCCGGCATTGGGGGAGCTGA

>LOC_Os01g23770.1 Oryza sativa subsp. japonica|M-type_MADS|M-type_MADS family protein

ATGCCGCGCGCGAAGACCCCGATGGGCCTCATCCCGTTCCCGAAGAAGCGCGCCGCGACGTTCGCGAGGAGGAAGGAGACCGTGATGAAAATGGCGAAGGAGCTCTCGGTGCTCTGCGACGCCCAAGTCGCGGTCCGCGCCAAGCGCTCGCTCACGCACCGGGAGTACCTCCGGGGGGAGCTCCGCAAGCAGAGGGCCAAGCTGGCCAAGGTCCGGGAGGAAGGCGCGTTCAAGCCGTGGGACGACGCGCTCGACGGCATCGCGGAGGAGGAGACGCGGAAGCTGCACAAGTACCTCAGCGACAAGATCGAGGCCGCGAGGGCGAGAATGGAGGCGATGGGTCTGCAGCTGGGCGACGTCGACGACAACGGCGTGAACGGCGACGACGGCGGCGGACTCGATCTGCAGCAGCACGTCCCGCCGAGCGCTTCCGACGCCAAGGAGTTCGAGTCCGTGCCTGTCGTCCATGGCGGCCAATACATCGGCAGCAGCAGCGGCGGCGGGGGCGGTGACATCCAGATGCAGACGACGCCGGCCGCAGATGGCATCAGCTTCGCCGAGCACTTTCTCGTCGCGCCGCGCGCTCAGCCCCTCGCCTTCAGCACCGCCGGCGCCGACTTCATCAACGCACCGAACAACTTCCTCACCACGGGCGTCAGCGTCAGCGACTACTCGGTGCAGAGCTCGGGCTACGGGATCGGAAACCAGATCGACAACGCCAAGCAACTCCTCTATCAGATGCAAATGCAATACCCAGTCGGTGGCACGGGTGGCGCCGAGCCCAGCAACACGCAGACGCAGTCCCCGGATTTGCGGAGCTTCAGCAACGTCGTCGGCGACTACTCGTACACGGCGGCGCAGAGCTCGGCCAACCGCCTCGATCAGATGCATCACCCAGTCGGTGGCCGTGGCAGCACGGGTGGCGCCGCCGCCGATCCCAGCGACACGCAGTCCAAGAATCGGGGGAGCTGA

>LOC_Os01g23780.1 Oryza sativa subsp. japonica|M-type_MADS|M-type_MADS family protein

ATGAAGATGGAGGAGGACGCCACGTACGGGAAGATGCAGGAGTCCCTGATGGAGGAGGCGAGGGAGCTCTCCATCCTCTGCGGCGTCGACGTCGCTCTCCTCTGTGCCGGCGGCCCCGGCACCGGAGACGGAGACGGCGGCGGTGCGGTGTCGACGGCGGAGGTGGCGGTGTGGGAGTCGGAGGAGGGCGTCCTCGCCTCGTACCGCGCCATCCTGCCTAGGCCGGAGGCGCACACGCTCCGAGAGTGCCTCGAGTTGAAGCTCGCCAGGGAGAGGGCCAAGCTCGCCATGCTACATCAGTACGGCGGAGGAGGCGCGGGCGCTGCTCGAGTCCATGGACGCGGCGATGGTGGCCGCGACCGCGAGGCGGGAGGCGCTGCGCTGGGTCTACTGGACGACGGCAACGCCGTGAACGGCGACGGCGGCGGACCCGATCTGCTGCACATCCCGATCCAGCCGGGCGCTCCCGACTCCGTCGTTGTGCCTGTCATCAGCCATGCTCAGCCCATCGCCTCCAGCACTGGCGCTGACTTCGCCGACGCGCCCAACGGCTTCCTCGCCATGGACGTCGGTGGCAGCCTCATCAAGTCGGCGACTACTCAGCGCAGTGCTCGGCCGATGGGTTCCAGCTCGGGGACGCCAACCATGGCCAACCTCGATCAGATACATTACCTAGTCGGTGGCTCGGCATTCTCCATAGATGCATTAGAAAGTCCTACACTCCTAGTTAGGTGCAAGACATTGCGAATTGCGATTCAGTTCATGTTGCCGTTGCGCCACGGCCGATTCCGAGCAAATGTACCGTCCAACCATCGACGTACGGAGACGAAATCTTAG

>LOC_Os01g67890.1 Oryza sativa subsp. japonica|M-type_MADS|M-type_MADS family protein

ATGGTCCGCACGCCCACCATAATCCCGCTACGGCCCAACTACAGCCCGCCGGGCGACGGTGCCGCATTCCGCCGCGAGCCCGCGACGTATTCCCGTTTCCCACCCGCCGCGGCTCGCAACGAATGTGATCTCACCTTCGGTCAGGAAAGGAAACGAGAGGGAATGAAAGGCAAGCCCCCGATATCAAGGAAAATCATTTATAATCACCGAGATTTTGTTTCCATGGGTGAAAGATTTGTTGCTCTTTGCTTTATATTTACGATGTCTCCATGCATTGATGCAACGCTATTCGTTCGCGTGGTTTTGGATTTCGTCCGGCGAGAGGAGACGAGACGAGACGAGATGCCTCGCACGAAGCTTGTGTTGAAGCTCATCGAGAACGAGAAGAAGCGGAAGGCCACGTTCAAGAATCGCCGGGACGGCCTCAAGCAGAAGGTGTCGCAGTTCGCGACGCTGTGCGGCGTCGAGGCCTTGCTCATCTGCGTCGCCCCCGCCGTGGCGGGCGGCGAGGTCACGACGTGGCCGCCCGACCGCGCCGCCGTGCTGGACCTCATCGCGAGGCTCCGGGCGACGCCGCCGGAGAAGATCCGGCAGCTGCACAACACCCAGTCCCAACTCCGGGACGATCTGGACAAGCAGCAGCGGTTGCTCCTCAAAGTCCAGAAGTGCGGCGCCGACGACGTGCTCACACCGTGGCACTGCAGCCTGTACGACCTCTCCCTCGACGGCCTCAATGCGCTCCACGACACCCTGTCCGAGACGCTGGACAGGGCGCACCGGCGGATAGCCGCGCTCGGCGGCGGCCACGGCCACGTCCACGACGACGCCGCGTCGTCCTCCGAGTTCTCGGTCCCCGCGCCCGCGCCGCACGCCGTGGCATTGCCGGACAACGCCTTCGACTTCCCGTTCGCCCCGTCGAACACCGGCCCCGTCGTCGGCGCCCACTACTTCTACCCTCTCCACGACACCCTGCCGCTGCCGCTGCCGCTGCCACAGCAGGTTCCCGGCCAGCATCCGCCGTGCATCGCCTACCAGATGCCGCCGCCGCCGTGCCTCGCCTATCAGATGCCGCCGCCGCCGCCGCCGTCGCTCGCCGCGGCGCCATTCGACCAGTGTATGAGCGCCACGGGCTTCATGGACAGCAACCCTTACGCCACCCACATCATGCACGGTGGCTCGACGGCTGCTGGCCTCCTCGACGATCATGGCCAGATCTTCTCCGCCGGCGCAGGGTACGACGACGACGACATTCTTGGGCACGGCTTCGGCTTCGCCGCCGGCACCGGGTACGATCTTGACCCGCGCATGGCCACCGCCGACGTTTGGCCGATGAACACGCTCAACAATATTCCAAACGATGGCGGCATCGGCTTCCAGCTGCAGAACGATCTGAAGTGGATGCTTCCAGGTGGTAGCAACGGGAGTAATTTGCAAGGCGGCTTTCAGATTTAG

>LOC_Os01g68560.1 Oryza sativa subsp. japonica|M-type_MADS|M-type_MADS family protein

ATGTCTCGCCGGAAGACATCCATAGCCCTCATCGCGAACCCGCAGACGCGCGCTACGACCTACAAGAAGCGGAAGGCGGGGCTGATCAAGAAGGCCGGGGAGCTCGCCACGCTGTGCGACATCCCCGTCGCCGTCGTGTGCGCCGGGCCCGACGGCGGCGCGCCGACCGTGTGGGTGTCGCCGGAGGGCGGCGACGCCATCGAGAGGTACCGCGCGCTGCCGGCGGAGAAGCGGGCGAGGCACACGCACGTGGCTTACCTCCAGGAGGAGCTCGACAAGGAGAGGGCCAAGCTCGCCAGGCTGCGGCAGAAGGGCCGCCCCGGCGAGCTCGACCCGCCGGACGCCGTGCTGGACGGGATGTCCCAGGACGAGCTGCAGCAGCTGCTCGCGTCCATCGACGCCACGCTGCTGGCTACGGCCAAGAGGCGGGAGGCGCTCGGGTTGCTGCCCGGCGCCGACGACGACGCGGACGGCGGCGGCCGGCGGCGCGACGCCGACGTCGCCGGAACCAACTCCGTCGGCGTACACGGCTACCAGCACCAGGAGGTGCATGCACCGGCGACATGTGACCCCTTCCATCCGTACAACGCCGGCGTGACGCTGATGCAGCCAGGGTACAACAACGCCCAGTACATGGGCGGCCATGGCGCCGTGGACATGAGCGGCTACCAGCTGCAGATGCAGATGCCGGGCAATGGCAGCAACAACCACAGCCGGCTCGCGTGGGGAGGCTTCCAGCCATGCAACGCCACCTTCGTCCAGCCCGTGTACGGCAACCTCCAATGCTGGTACAACAATGTCGTCGACGGCAACGGCGAGCCCTGCGACGCCATCGTGCCATCAGCTGGTGACCCCTACATGGACATCGCCGGCAACGACGTCTACGGCAACCAGATGCAGCCGGCACCGGCGGCCAACGGCGGGTGGCACGATCCGGGCACGTGGGGCTACGACGGCGGCGAGCCGTGCAAAGCCATCGTGCCATCATTTGGTGACCCCTACATGGGCATTGGCGTCTACGGCAACCAAATGCAGCCGGCGCCGGCGCCGGCGGCCAACGGCTGCTGGCACAATCCGGCCGGCACATGGGGCAACGACGGCGAGCCGTGCAACGCCATCGTGCCATCAGCTGGTCACCCCTACATCGACATCGAGTGCGACATCGACGGCAACTACATCGACACAACCGTGTTCGATTACCAAACCACCAGCACCAGCGACAACTTCATGGACGCGCCCGTCCAGTTCATCGCCACGGGCAGTGACGAGAGCATCGTCACGAACGTGGCCGGCTGCGACGAGACGGAGTTCTCCATTGACGATCTCCTCCAGTGCTCCGACGCCTCCCAGCATTCTTCCGGCCTCGAGGAGCTGCATTATCTCAGTGATCTGGCCGATGGCTTCGACTTTGGATGCAACTTCGACGTGCTATTGGACTGA

**#amino acid sequences of MADS-box genes in *Oryza sativa***

>LOC_Os03g11614.1 Oryza sativa subsp. japonica|MIKC_MADS|MIKC_MADS family protein

MGRGKVELKRIENKISRQVTFAKRRNGLLKKAYELSLLCDAEVALIIFSGRGRLFEFSSSSCMYKTLERYRSCNYNSQDAAAPENEINYQEYLKLKTRVEFLQTTQRNILGEDLGPLSMKELEQLENQIEVSLKQIRSRKNQALLDQLFDLKSKEQQLQDLNKDLRKKLQETSAENVLHMSWQDGGGHSGSSTVLADQPHHHQGLLHPHPDQGDHSLQIGYHHPHAHHHQAYMDHLSNEAADMVAHHPNEHIPSGWI*

>LOC_Os12g10540.1 Oryza sativa subsp. japonica|MIKC_MADS|MIKC_MADS family protein

MGRGRIEIKRIENTTSRQVTFCKRRNGLLKKAYELSVLCDAEVALIVFSSRGRLYEYSNNNNVKATIDRYKKAHACGSTSGAPLIEVNAQQYYQQESAKLRHQIQMLQNTNKHLVGDNVSNLSLKELKQLESRLEKGISKIRARKNELLASEINYMAKREIELQNDNMDLRTKIAEEEQQLQQVTVARSAAMELQAAAAAQQQQQNPFAVAAAQLDMKCFFPLNLFEAAAQVQAVAAQRQQIIPTELNLGYHHHLAIPGAAAADAPPPHF*

>LOC_Os03g54160.1 Oryza sativa subsp. japonica|MIKC_MADS|MIKC_MADS family protein

MGRGKVQLKRIENKINRQVTFSKRRSGLLKKANEISVLCDAEVALIIFSTKGKLYEYATDSCMDKILERYERYSYAEKVLISAESDTQGNWCHEYRKLKAKVETIQKCQKHLMGEDLESLNLKELQQLEQQLENSLKHIRSRKSQLMLESINELQRKEKSLQEENKVLQKENPCSFLQLVEKQKVQKQQVQWDQTQPQTSSSSSSFMMREALPTTNISNYPAAAGERIEDVAAGQPQHVRIGLPPWMLSHING*

>LOC_Os07g01820.1 Oryza sativa subsp. japonica|MIKC_MADS|MIKC_MADS family protein

MGRGKVQLKRIENKINRQVTFSKRRNGLLKKAHEISVLCDAEVAAIVFSPKGKLYEYATDSRMDKILERYERYSYAEKALISAESESEITLPQLTTCTASRSTHGICFQYCLMSKTLGNWCHEYRKLKAKIETIQKCHKHLMGEDLESLNLKELQQLEQQLESSLKHIISRKSHLMLESISELQKKERSLQEENKALQKELVERQKNVRGQQQVGQWDQTQVQAQAQAQPQAQTSSSSSSMLRDQQALLPPQNICYPPVMMGERNDAAAAAAVAAQGQVQLRIGGLPPWMLSHLNA*

>LOC_Os06g49840.1 Oryza sativa subsp. japonica|MIKC_MADS|MIKC_MADS family protein

MGRGKIEIKRIENATNRQVTYSKRRTGIMKKARELTVLCDAQVAIIMFSSTGKYHEFCSPSTDIKGIFDRYQQAIGTSLWIEQYENMQRTLSHLKDINRNLRTEIRQRMGEDLDGLEFDELRGLEQNVDAALKEVRHRKYHVITTQTETYKKKVKHSYEAYETLQQELGLREEPAFGFVDNTGGGWDGGAGAGAAADMFAFRVVPSQPNLHGMAYGGNHDLRLG*

>LOC_Os04g49150.1 Oryza sativa subsp. japonica|MIKC_MADS|MIKC_MADS family protein

MDRSEMGRGRVELKRIENKINRQVTFSKRRNGLLKKAYELSVLCDAEVALIIFSSRGKLYEFGSAGINKTLEKYNSCCYNAQGSNSALAGGEHQSWYQEMSRLKTKLECLQRSQRHMLGEDLGPLSIKELQQLEKQLEYSLSQARQRKTQIMMEQVDDLRRKERQLGELNKQLKNKLEAEADSSNCRSAIQDSWVHGTVVSGGRVLNAQPPPDIDCEPTLQIGYYQFVRPEAANPRSNGGGGDQNNNFVMGWPL*

>LOC_Os07g41370.1 Oryza sativa subsp. japonica|MIKC_MADS|MIKC_MADS family protein

MGRGPVQLRRIENKINRQVTFSKRRNGLLKKAHEISVLCDADVALIVFSTKGKLYEFSSHSSMEGILERYQRYSFDERAVLEPNTEDQENWGDEYGILKSKLDALQKSQRQLLGEQLDTLTIKELQQLEHQLEYSLKHIRSKKNQLLFESISELQKKEKSLKNQNNVLQKLMETEKEKNNAIINTNREEQNGATPSTSSPTPVTAPDPIPTTNNSQSQPRGSGESEAQPSPAQAGNSKLPPWMLRTSHT*

>LOC_Os01g66030.1 Oryza sativa subsp. japonica|MIKC_MADS|MIKC_MADS family protein

MGRGKIEIKRIENSTNRQVTFSKRRSGILKKAREISVLCDAEVGVVIFSSAGKLYDYCSPKTSLSRILEKYQTNSGKILWDEKHKSLSAEIDRIKKENDNMQIELRHLKGEDLNSLQPKELIMIEEALDNGIVNVNDKLMDHWERHVRTDKMLEDENKLLAFKLHQQDIALSGSMRDLELGYHPDRDFAAQMPITFRVQPSHPNLQENN*

>LOC_Os12g31748.1 Oryza sativa subsp. japonica|MIKC_MADS|MIKC_MADS family protein

MGRGKVQVRRIENEVSRQVTFSKRRPGLLKKAHEIAVLCDVDVAAIVFSAKGNLFHYASSHTTMERILEKYDRHELLSEGNNVIEEFPELEGSMSYDHIKLRGRIEALKKSQRNLMGQELDSLTLQDIQQLENQIDTSLNNIRSRKEKLLMEKNTILEKKITELETLHTCIRASPTKAAAPPACNTADAFVPNLNICCGDSGEPETVTAPLGWTSSNNGLPWWMLQSSSNGKS*

>LOC_Os01g66290.1 Oryza sativa subsp. japonica|MIKC_MADS|MIKC_MADS family protein

MGRGKIEIKRIENKTSRQVTFCKRRNGLLKKAYELAILCDAEIALIVFSSRGRLYEFSNVNSTRSTIERYKKASASTSGSAPVIDVNSHQYFQQEAAKMRHQIQTLQNANRHLIGESIGNMTAKELKSLENRLEKGISRIRSKKHELLFSEIEYMQKREADLQNENMFLRAKVAEAERAEHDDQQAAEDDEMAPAPAVGGGSSSGTELEALPATFDTREYYQPAPPVSMLAAAAAAAAAQYSSDHHQTALHLGYFKVDSGKGGLL*

>LOC_Os02g52340.1 Oryza sativa subsp. japonica|MIKC_MADS|MIKC_MADS family protein

MARERREIKRIESAAARQVTFSKRRRGLFKKAEELSVLCDADVALIVFSSTGKLSHFASSSMNEIIDKYNTHSNNLGKAEQPSLDLNLEHSKYAHLNEQLAEASLRLRQMRGEELEGLSIDELQQLEKNLEAGLHRVMLTKDQQFMEQISELQRKSSQLAEENMQLRNQVSQISPAEKQVVDTENFVTEGQSSESVMTALHSGSSQSQDNDDGSDVSLKLGLPCGAWK*

>LOC_Os08g33488.1 Oryza sativa subsp. japonica|MIKC_MADS|MIKC_MADS family protein

MGRGKIEIKRIDNATSRQVTFSKRRSGLFKKARELSILCDAEVGLLVFSSTSRLYDFASSSMKSIIERYNETKEDPHQTMNASSEAKLWQQEAASLRQQLHNLQEYHRQLLGQQLSGLDVEDLQNLESKLEMSLKNIRLRKDNVMMDQIQELSRKVVTT*

>LOC_Os04g23910.1 Oryza sativa subsp. japonica|MIKC_MADS|MIKC_MADS family protein

MGRGKIAIKRIDNTMNRQVTFSKRRGGLMKKARELAILCDADVGLIVFSCTGRLYDFSSSSMKSIIERYQEAGEEHCRLLNPMSEAKFWQREVTTLRQQVQNLHHNNRQLLGEEISNFTVRDLQLLQNQVEMSLHSIRNKKDQLLAEEILKLNEKGSLVQKENSELRKKFNIAHQRNIELHKKLNSGESTSSEQVTRSSKDPGESSTPRDSRVCIDLELSQKEVEDE*

>LOC_Os08g02070.1 Oryza sativa subsp. japonica|MIKC_MADS|MIKC_MADS family protein

MARGKVQLRRIENPVHRQVTFCKRRAGLLKKARELSILCEADIGIIIFSAHGKLYDLATTGTMEELIERYKSASGEQANACGDQRMDPKQEAMVLKQEINLLQKGLRYIYGNRANEHMTVEELNALERYLEIWMYNIRSAKMQIMIQEIQALKSKEGMLKAANEILQEKIVEQNGLIDVGMMVADQQNGHFSTVPLLEEITNPLTILSGYSTCRGSEMGYSF*

>LOC_Os02g36924.1 Oryza sativa subsp. japonica|MIKC_MADS|MIKC_MADS family protein

MGRGKIVIRRIDNSTSRQVTFSKRRNGIFKKAKELAILCDAEVGLMIFSSTGRLYEYSSTSMKSVIDRYGKSKDEQQAVANPNSELKFWQREAASLRQQLHNLQENHRQLMGEDLSGLNVKELQSLENQLEISLRSVRTKKDHVLIDEIHELNRKGSLVHQENMELYKKISLIRQENAELYKKIYETEGPSEVNRDSPTPYNFAVIEKTNVPVQLGLSTLPQHSDAEQSTAPKLGLQLNP*

>LOC_Os02g07430.1 Oryza sativa subsp. japonica|MIKC_MADS|MIKC_MADS family protein

MGRGKIEIKRIENATNRQVTFSKRRGGLLKKANELAVLCDARVGVVIFSSTGKMFEYCSPTCSLRELIEHYQTVTNTHFEEINHDQQIFVEMTRMRNEMEKLDGGIRRFTGDDLSNLTLADINDLEQQLEFSVTKVRARKHQLLNQQLDNLRRKEHILEDQNSFLCRMINENHHQAAVGGGDVKAMVEMAPVLSMLTAAPAYYGEESSSTALQLTPPLHAVDAAAAAGFRLQPTQPNLQDPGCSSSSFHAAAAGHGLQLW*

>LOC_Os01g10504.1 Oryza sativa subsp. japonica|MIKC_MADS|MIKC_MADS family protein

MMNMMTDLSCGPSSMTELTAAAAPAGSGSSAAVAAGSSEKMGRGKIEIKRIENTTNRQVTFCKRRNGLLKKAYELSVLCDAEVALIVFSSRGRLYEYANNSVKSTVERYKKANSDTSNSGTVAEVNAQHYQQESSKLRQQISSLQNANSRTIVGDSINTMSLRDLKQVENRLEKGIAKIRARKNELLYAEVEYMQKREVELQNDNMYLRSKVVENERGQQPLNMMGAASTSEYDHMVNNPYDSRNFLQVNIMQQPQHYAHQLQPTTLQLGSRPSISFGVDTVRTHVR*

>LOC_Os06g45650.1 Oryza sativa subsp. japonica|MIKC_MADS|MIKC_MADS family protein

MGQGKIEMKRIEDATRRQVTFSKRRAGFLKKANELAVLCDAQVGVVVFSDKGKLFDFCSPPVILMELFHRYEITTRNTRLQETNRDDEQMVMEITRLRNEIDQLEASLRRQTGEDLSSVSTVDELSQLQLQLESSLSKVHARKDELMSQQLEDMRRMHQTVHEQNNFLCRMVTKILLLNVITIMILAAMISITHARIALDDCMQLGYIVIKQENSWRFWFI*

>LOC_Os04g52410.1 Oryza sativa subsp. japonica|MIKC_MADS|MIKC_MADS family protein

MGRGRVELKKIENPTNRQVTFSKRRMGLLKKANELAILCDAQIGVIVFSGTGKMYEYSSPPWRIANIFDRYLKAPSTRFEEMDVQQRIIQEMTRMKDENNRLRIIMRQYMGDDLASLTLQDVSNLEQQIEFSLYKVRLRKQQLLDQQLLEMHSRVCNKRIYRFSILPYLIHYIEIIKF*

>LOC_Os01g52680.1 Oryza sativa subsp. japonica|MIKC_MADS|MIKC_MADS family protein

MGRGRSEIKRIENPTQRQSTFYKRRDGLFKKARELAVLCDADLLLLLFSASGKLYHFLSPTVPSVREFVERYEATTHTKVWADIRQERRAELEKVGSMCDLLEKQLRFMTVDDGEEYTVPSLEALEHNLEAAMRKVRSEKDRKIGGEICYLQNIIRGRQEERYGLCDKIAHAQTLKDVECGSTSLSNGLDLKLGFN*

>LOC_Os12g10520.1 Oryza sativa subsp. japonica|MIKC_MADS|MIKC_MADS family protein

MVRGKVQMRRIENPVHRQVTFCKRRGGLLKKARELSVLCDADVGVIIFSSQGKLHELATNGNMHNLVERYQSNVAGGQMEPGALQRQQVAEQGIFLLREEIDLLQRGLRSTYGGGAGEMTLDKLHALEKGLELWIYQIRTTKMQMMQQEIQFLRNKEGILKEANEMLQEKVKEQQKLYMSLLDLHSQQPTQPMTYGNRFFSI*

>LOC_Os03g54170.1 Oryza sativa subsp. japonica|MIKC_MADS|MIKC_MADS family protein

MGRGKVVLQRIENKISRQVTFAKRRNGLLKKAYELSILCDAEVALVLFSHAGRLYQFSSSSNMLKTLERYQRYIYASQDAAAPTSDEMQNNYQEYVNLKAHVEILQQSQRNLLGEDLAPLATNELEQLESQVVRTLKQIRSRKTQVLLDELCDLKRKEQMLQDANRVLKRKLDEIDVEAAPPQPPWNGNCSNGHGGGGGVFSSEPPQPEHFFQALGLHAVDVNQPPAPPPGGYPPEWMA*

>LOC_Os08g41960.1 Oryza sativa subsp. japonica|MIKC_MADS|MIKC_MADS family protein

MEGGGRRRKRGKVELRRIEDRTSRQVRFSKRRSGLFKKAYELSVLCDAQVALLVFSPAGRLYEFASSTSSIDTIFGRYWDLLDTTIDLNIEARESRVDCNIQLRQKERSDDPVPKINHITQCVLESNVNELNIAELRGLEEAMTNALTVVKNKLMMKVASVLPQSEKKRKSCSISEPRSGVSS*

>LOC_Os05g34940.1 Oryza sativa subsp. japonica|MIKC_MADS|MIKC_MADS family protein

MGRGKIEIKRIENSTNRQVTFSKRRAGILKKAREIGVLCDAEVGVVIFSSAGKLSDYCTPKTTSVFPPLSRILEKYQTNSGKILWDEKHKSLSAEIDRVKKENDNMQIELRHMKGEDLNSLQPKELIAIEEALNNGQANLRDKMMDHWRMHKRNEKMLEDEHKMLAFRVHQQEVELSGGIRELELGYHHDDRDFAASMPFTFRVQPSHPNLQQEK*

>LOC_Os03g08754.1 Oryza sativa subsp. japonica|MIKC_MADS|MIKC_MADS family protein

MAGGGGGGGRGEGEGRAATGKRERIAIRRIDNLAARQVTFSKRRRGLFKKAEELSILCDAEVGLVVFSATGKLFQFASTSMEQIIDRYNSHSKTLQRAEPSQLDLQGEDSSTCARLKEELAETSLRLRQMRGEELHRLNVEQLQELEKSLESGLGSVLKTKSKKILDEIDGLERKRMQLIEENLRLKEQLQVSRMSRMEEMQPGPDSEIVYEEGQSSESVTNASYPRPPPDNDYSSDTSLRLGLSLFSSK*

>LOC_Os06g06750.1 Oryza sativa subsp. japonica|MIKC_MADS|MIKC_MADS family protein

MGRGKVELKRIENKISRQVTFAKRRNGLLKKAYELSVLCDAEVALIIFSTRGRLFEFSTSSCMYKTLERYRSCNYNLNSCEASAALETELSNYQEYLKLKTRVEFLQTTQRNLLGEDLVPLSLKELEQLENQIEISLMNIRSSKNQQLLDQVFELKRKEQQLQDANKDLKRKIQETSGENMLHISCQDVGPSGHASEANQEFLHHAICDPSLHIGYQAYMDHLNQ*

>LOC_Os03g03100.1 Oryza sativa subsp. japonica|M-type_MADS|M-type_MADS family protein

MVRGKTQMKRIENPTSRQVTFSKRRNGLLKKAFELSVLCDAEVALIVFSPRGKLYEFASARKIRPEKTAKTIFPRVAIELPSKQSHYFHKEISCETGKVRNSENIGNLVLAFRRAI*

>LOC_Os06g11330.1 Oryza sativa subsp. japonica|MIKC_MADS|MIKC_MADS family protein

MARERREIRRIESAAARQVTFSKRRRGLFKKAEELAVLCDADVALVVFSSTGKLSQFASSNMNEIIDKYTTHSKNLGKTDKQPSIDLNMRGEELEGLSVEELQQMEKNLEAGLQRVLCTKDQQFMQEISELQRKGIQLAEENMRLRDQMPQVPTAGLAVPDTENVLTEDGQSSESVMTALNSGSSQDNDDGSDISLKLG*

>LOC_Os10g39130.1 Oryza sativa subsp. japonica|MIKC_MADS|MIKC_MADS family protein

MVRGRTELKRIENPTSRQVTFSKRRNGLLKKAFELSVLCDAEVALIVFSPRGRLYEFASAPSLQKTIDRYKAYTKDHVNNKTIQQDIQQVKDDTLGLAKKLEALDESRRKILGENLEGFSIEELRGLEMKLEKSLHKIRLKKTELLEQQIAKLKEKERTLLKDNENLRGKHRNLEAAALVANHMTTTTAPAAWPRDVPMTSSTAGAADAMDVETDLYIGLPGTERSSNRSETG*

>LOC_Os02g49840.1 Oryza sativa subsp. japonica|MIKC_MADS|MIKC_MADS family protein

MGRGKIVIRRIDNSTSRQVTFSKRRNGLLKKAKELSILCDAEVGLVVFSSTGRLYEFSSTNMKTVIDRYTNAKEELLGGNATSEIKIWQREAASLRQQLHNLQESHKQLMGEELSGLGVRDLQGLENRLEISLRNIRMRKDNLLKSEIEELHVKGSLIHQENIELSRSLNVMSQQKLELYNKLQACEQRGATDANESSSTPYSFRIIQNANMPPSLELSQSQQREGECSKTAAPELGLHLP*

>LOC_Os05g11414.1 Oryza sativa subsp. japonica|MIKC_MADS|MIKC_MADS family protein

MHIYKEQEAEPSTGLMMPEPAPVASPGSGGSGGSGSVGAEKIGSRGKIEIKRIENTTNRQVTFCKRRSGLLKKAYELSVLCDAEVALVVFSSRGRLYEYSNNSVKETIERYKKANSDTSNASTVAEINAQHYQQEAAKLKQQITNLQNSNRTLVGDNITTMNHRELKQLEGRLDKGLGKIRARKNELLCAEIEYMQRRETELQNDNMYLKSKVAESERGLQTVNMMGSASTSEYVQNMIHYDPRNFLQFNIMHQPQYYPEQEDRKAFMSGKKYSQCNIVRVHSSTNEI*

>LOC_Os06g23950.1 Oryza sativa subsp. japonica|M-type_MADS|M-type_MADS family protein

MVRGKTVISRIENTTSRQVTFSKRRSGLFKKAKELAILCDAQVGVLVFSSTGRLYDYSNSSNSRNLTYDQLLGDTVC*

>LOC_Os02g45770.1 Oryza sativa subsp. japonica|MIKC_MADS|MIKC_MADS family protein

MGRGRVELKRIENKINRQVTFSKRRNGLLKKAYELSVLCDAEVALIIFSSRGKLYEFGSAGITKTLERYQHCCYNAQDSNNALSETQSWYHEMSKLKAKFEALQRTQRHLLGEDLGPLSVKELQQLEKQLECALSQARQRKTQLMMEQVEELRRKERQLGEINRQLKHKLEVEGSTSNYRAMQQASWAQGAVVENGAAYVQPPPHSAAMDSEPTLQIGYPHQFVPAEANTIQRSTAPAGAENNFMLGWVL*

>LOC_Os04g38770.1 Oryza sativa subsp. japonica|M-type_MADS|M-type_MADS family protein

MGRGKIVIRRIDNSTSRQVTFSKRRNGIFKKAKELAILCDAEVGLVIFSSTGRLYEYASTSMKSVIDRYGRAKEEQQHVANPNSELKEFCSVFIYITEN*

>LOC_Os08g38590.1 Oryza sativa subsp. japonica|M-type_MADS|M-type_MADS family protein

MGRVKLPIKRIENTTNRQVTFSKRRNGLIKKAYELSVLCDIDVALLMFSPSGRLSHFSGRRGVEDVILRYMNLSEHDRGEAIQNREEIQQEIYSSQQQLQITEDRLRMFEPDPAAFGTSSEVDGCEKYLMELLTRVVERKNNLLSSHMAPFDATTAAMQGADGTQMYVSQADGLATFGGDAAMWGPDGGADPGHPMFSASDPLIYLRDHDVYDANSQVAGLHGGDPCAAGGAAAAAAAVGCVDDDVAGGHAAAADAWRQAYTCTELLSTLIPTTPFPLMPHCLGPEDQYLSMEHGMVAAAQEPVEASTASCSYVPSDENSGTPVMAYDSNPPPANIA*

>LOC_Os06g11970.1 Oryza sativa subsp. japonica|M-type_MADS|M-type_MADS family protein

MGRVKLQIKRIENIPNRQVTFSKRRNGLIKKAYELSVLCDIDIALLMFSPSGRLSHFSGRRRIEDVLTRYINLPESDRGGTIQNREYLINMLTQLKCESDVTEDLTNTSSKAPVNSNIEELQQEIRRCQHQMQLTEEQLRMFEPDPARSASMEDVEASEKFIAGILSRVEERKRYLLCSMGSFDVTASTSAMQHLYLPQQHQHGDITDNGFGSDEVASWVSEGMPPTTSSVASIFAGTSDSMMSFRDQAVYDTMRQDACVDQTVVPEMGMCHVDQQNQSDDWQAYTSAEFLNALIPPTPFPLDDEDTMGPMLASSPLLMPGIHDQQPPVEDMATAGCSQAPANDGNGLYAAEDIAPVNVG*

>LOC_Os04g31804.1 Oryza sativa subsp. japonica|M-type_MADS|M-type_MADS family protein

MGRRGRVVLRRIEDRVRRGICFRKRLAGLEKKVEELAVLCDAHVGFVVLSCSGDDANPHHFAAPATIENIVERYEHSQAAQKGVHGRCILQKRKSKDFQVLKETIDKGPINDDMRPIDEKDISTLNMDQISQIEILLEDELRWTRARKVVADRIARLQKKVQKKPATAETESNSTEMPSDHEKKQVAGGSQQSAEEEEEEMEVVLRHRLSLGTGDRDDGGGGAAEQRHRTTPPPAVDLNVPCRDAGQLQ*

>LOC_Os01g69850.1 Oryza sativa subsp. japonica|M-type_MADS|M-type_MADS family protein

MPPPPPPHSIDPLLLLPHFSPPPHHHTHARTHARLRFPPPTRGGEGGELGFFGAGMARRGRVQLRRIEDKASRQVRFSKRRAGLFKKAFELALLCDVEVALLVFSPVGKLYEYSSSSIEGTYDRYQQFAGARRDLNEGSTSINSDENASIHSRLRDITAWSLQNNADESDANQLEKLEKLLTNALRDTKSKKMLAKQNGEGSRSRANSSGSRGQEEGSA*

>LOC_Os05g11380.1 Oryza sativa subsp. japonica|MIKC_MADS|MIKC_MADS family protein

MHIYKEQEAEPSTGLMMPEPAPAASPGSGSSEGSSIEDTADRQVTFCKRCNGLLKKAYELSMLCDAEVALIVFSSRGRLYEYSNNSVEETIERYKKANSDTSNTSTVAEINAQHYQQEAAKLKQHITYLQNSNRFIISLSCLDFFFST*

>LOC_Os11g43740.1 Oryza sativa subsp. japonica|M-type_MADS|M-type_MADS family protein

MGRVKLKIKKLENSSGRHVTYSKRRSGILKKAKELSILCDIPLILLMFSPNDKPTICVGDHSSIEDVITKYAQQTPQERAKRKLESLEALKKTFKKLDHDVNIQDFLGSGGQTVEMLIKSIVWQELSSHLGALQCQMADVEKRLSYWSDPEKVENIDHIRAMEQSLKESLNRIRIHKENFAKQHLMSLQCAAAQFQNDMKLPLGLTGDPNTSSWFHGGGGAEAQQPMMLPEDPSLLHQRDIGCSASTSLQSYPGYFSMGKQSTDNAGGGEQHHHAAVQQQPEFSQADCLTSLQLGAQFPYPSAFDNAGLLSDRLFDNAAAAAAAMDFGGHYDLPRPGDEASFQNWASAACGATMYDHQQQQQQQQQPAQLPAAATVEAPSFNHPSPHRQLMI*

>LOC_Os08g41950.1 Oryza sativa subsp. japonica|MIKC_MADS|MIKC_MADS family protein

MAEKKKKKKKKKPQSLLVLTSWRSIGMGRGRVELKRIENKINRQVTFAKRRNGLLKKAYELSVLCDAEVALIIFSNRGKLYEFCSTQSMTKTLEKYQKCSYAGPETAVQNRESEQLKASRNEYLKLKARVENLQRTQRQYYKSKHRLCLVRSKVWNLVKIRDDVTEKLCMYERNLLGEDLDSLGIKELESLEKQLDSSLKHVRTTRTKHLVDQLTELQRKEQMVSEANRCLRRKLEESNHVRGQQVWEQGCNLIGYERQPEVQQPLHGGNGFFHPLDAAGEPTLQIGYPAEHHEAMNSACMNTYMPPWLP*

>LOC_Os05g23780.1 Oryza sativa subsp. japonica|M-type_MADS|M-type_MADS family protein

MVKGKGRAGRKKVEIKRIEKKDARDVCFSKRRQTLFNKAGELSLLCNANIAAVVISPAGRGFSFAHPSVDDVADRLASMAMGIPNNHSLGGGYHDSGEVTNIAQQQKIEYVELQKSLEKSEKKKRVQEAMEKERAGHLMQSLTSEVNLLGQDELEELHNKLSALPYTSIAKFYQVLQDAKGTRMPLPQPHIEIACQSQFLFEEQAVTPANADFPGSST*

>LOC_Os06g22760.1 Oryza sativa subsp. japonica|M-type_MADS|M-type_MADS family protein

MTKRKIEIKRIKNEEARQVCFSKRRPSVFKKASELYTVCGAEVAMLVKSPAGKFFSFGAPSVGFVLSRFHATTTSRKHSSMGVTIQHDNSATIKLHELNQQHIELQNQLQAQNEKMKALQEVAKKESGGKVMGWLNSKVEDICQEDLEEFKMVLESLKYLTRGIINQLFQNYAMFSNMMRVQHCVTALPNQQFLPSSEDVKPMIHHVPSSSYGWNTSIDSKPNSSDAHVVGARRYFPK*

>LOC_Os03g14850.1 Oryza sativa subsp. japonica|M-type_MADS|M-type_MADS family protein

MVKCRARTTRKKIEIKRGDKKVRDACFSKRHTTIFNKANELAILCGVMVAVVFVSPNANGGIFSFGYPSVSSVANRFLANAPNNTSVSSSTQSGRDVEIRELEREERELKEHLQASTDQNKLLREAIAARDGGQLMLLLQSDCSELGPKGLVAVVRLWFRNSLVGIGLDRLCWLLQQILFGLDWG*

>LOC_Os12g21850.1 Oryza sativa subsp. japonica|M-type_MADS|M-type_MADS family protein

MVHPLGRTSMGRQRIEIRRIDNKERRQVTFTKRRGGLFKKASELALLTGASVAVVVFSPAKHVYAFGHPSVDAVLRSYASVPGEAAAVAPVPVHGGSGGEDVDLLGLRLAADDTGAQVAAEHARMRDVAARIVQAKAGRRFWWEADVDALGEAELLEFFTALKKLRDNVGRHANALLAPQPPPLPLQQKQRRRR*

>LOC_Os12g21880.1 Oryza sativa subsp. japonica|M-type_MADS|M-type_MADS family protein

MRPLGRTSKGRQHIDNKERRQVTFTKRRGGLFKKASELALLAGASIAVVVFSETNLAYAFGDPSVDAVLLSYGPVPGEDAEPAPVHSGGLGKDVDLEMLRHNNCASSLQFVLVSLSFIM*

>LOC_Os06g30810.1 Oryza sativa subsp. japonica|M-type_MADS|M-type_MADS family protein

MVRSRGRPSLGRQRIEIRRIDNSGRRQVTFSKRRNGLFKKASELSTLCGASVAVVAFSSAGNVFAFGQPTVDAVVRRFDPLHADGADPAPAAVEDGGGGGDDVVVADPEELDALRRAEEQTKAQVAAEQARMRDVGDKVTQAMAGRALWWEADVEALGEAELPEFVRALERLRDSVHRHASTLASTATPLPPPPEQEEEVPELDVSDYSF*

>LOC_Os06g30830.1 Oryza sativa subsp. japonica|M-type_MADS|M-type_MADS family protein

MGRPRGGTSKGKQKIEMCCIDGKEKRQVTFSKRRRGLFKKASEISTLSGASIAIVSFSKAGNVFAFGSPSVDAVLRRHVVAGPSTSTSHAHAGGDVFADDGGDNPEVLNALKRATDEAAAEVAAEDARQSGVEGKITEAMAAGRRRFWWDAANVEALGEAELPVFERALHKLRGAVAQGGNNPIQERWDRIPAQGSPGGSYGWEDLSPTGKLYNQPPFAYRLPVMQSIVSDQLGVVCAMLL*

>LOC_Os09g02780.1 Oryza sativa subsp. japonica|M-type_MADS|M-type_MADS family protein

MESEEARKVCFSKRRADLFKMASELSVHFNADVAAVVFSPAGNRAYSIGDPSVMDRFLSSLPAPAPPAETEPEPEVDWSVMEELSRLCGQLQAMVDAHKARLEKAEEKLRESGAAAWMMDLEAEVGRMAPEDVLALVTKLAVLRDGVAERAHEMLREALLAVAAPTPTTPTTPPPAGF*

>LOC_Os09g02830.1 Oryza sativa subsp. japonica|M-type_MADS|M-type_MADS family protein

MVTAAAARRRPSLGRQKIEIRRIESEEARQVCFSKRRAGFFKKASELSILCSADVAAVVFSPAGKAYSFGHPSVEFLLDRFLSSSLPATAGKEEGSSVSVVAELNRQYGELRAMVDAHKARRERAEKTMEKQRQRQPAAWMDPEAEVGRMAPEELMALGTKLVAVQGGVAARADQMLRDALLLGRRPNTTTTTTTRAPPGFFHLHPHF*

>LOC_Os01g74440.1 Oryza sativa subsp. japonica|M-type_MADS|M-type_MADS family protein

MAMQVAAPSRRRPSLGRQKIEIRRIESEEARQVCFSKRRAGFFKKASELSILCSADVAAVVFSPAGKAYSFGHPSVECLLERFLPDSSSGAAARVRRGANNNGGGGMVGELNRQYGELRAMVEAHKARRERADEKIEMERAAGRWLPMDADVRRMSPEELMAFGTGLMAVQAAVSARADQMLRDALLIGRRPPTTTTAGFGFFHMPHY*

>LOC_Os09g32948.1 Oryza sativa subsp. japonica|MIKC_MADS|MIKC_MADS family protein

MGRGRVELKRIENKINRQVTFAKRRNGLLKKAYELSVLCDAEVALIIFSNRGKLYEFCSGQSMTRTLERYQKFSYGGPDTAIQNKENELVQSSRNEYLKLKARVENLQRTQRNLLGEDLGTLGIKELEQLEKQLDSSLRHIRSTRTQHMLDQLTDLQRREQMLCEANKCLRRKLEESNQLHGQVWEHGATLLGYERQSPHAVQQVPPHGGNGFFHSLEAAAEPTLQIGFTPEQMNNSCVTAFMPTWLP*

>LOC_Os02g06860.1 Oryza sativa subsp. japonica|M-type_MADS|M-type_MADS family protein

MAKAAAEMDVGGGEGRRWKRTRGRQRIEMKLIENKEARQVCFSKRREGVFKKASELSVLCGARVAVVFFSPAGRPHCFGHPSVSAVADRFLLGRSPADAAAAAAEEEEAVVREFNRVEERLKDALGAAARRRDALDEAARVAGVWNDADVRRAGLADLVSMLAALQRVQAEASERVHDIIVEEAMTHYTGAAAAAANLIDYLDAGPLVSHSPGSHDTTTKLIGGNAVHAPPLSFPPMIMPPPLPPQFSHGFGYTDLAAGYGYNLDHGHGAAYETEEFHNAAACDFF*

>LOC_Os04g25870.1 Oryza sativa subsp. japonica|M-type_MADS|M-type_MADS family protein

MALRKMKLQRIVIDVKRQVTFKKRLNGLTKKVSEFATLFLMVYGEVEVQATKVWPLVWKATRVLEHFKAMPQLDRYKKMTDLEDILNEQVDKLKEELHKVGRDADESDTKLILVEALNGHRPSLEGLTIEQITSLGLMANAHLKIVNDRLKKLREQGLIPASLLLSGTEVPIQREGWLMDVARGIGSMGNNRFGGTSGSGIAGSNGDMA*

>LOC_Os03g37670.1 Oryza sativa subsp. japonica|M-type_MADS|M-type_MADS family protein

MFKKRQKSLMKKASELSTLYGVDACVVMYAEGEAQPMMVWPSVPEARRVIERFRALPQKDQYENTTNLEGFLKQRIANLQEKVDKAKHENDELETKLLLLNSLDCCLPSLVGLTVKQITSLNSMVEERLKKLRGNGLLATPVPTSSKKKIIWDVRYNLKARYVTDDSAKNGSPRLRCTPATSSQRQSSPEFRHRRCLP*

>LOC_Os03g38610.1 Oryza sativa subsp. japonica|M-type_MADS|M-type_MADS family protein

MARNKVKLQRIINDAKRRATFKKRLKGLMKKASELATLCNVDTCLMVYGEGEAQATVVWPSESEVMRVLERFKTLPQLDKYKKMTDLEGFIQERINKFQEQLDKVRRDADESETKLLLIEALEGRRPGLEGITIEQLTSLGWLVDARLNIVNDQLQKLHEQGLLPASISLPTMGVLPYTTAGYTVAQEAPIQRGGWLMGVVRGIGSLGYSLFRGSGRSNTAGPSGDMVQPFNIGAGSSLANQGISFPPK*

>LOC_Os01g18420.1 Oryza sativa subsp. japonica|M-type_MADS|M-type_MADS family protein

MARNRIILKKVAKDSTRRLTFKKRRRGLIKKAGELASLCGIGVCVVVYGEGEVKPEVWPSAPEARAILSRFNSAPNIDRFKRVTNQEQYLRKRIAKARERTSKADDVNRERDATIMLYEAATDKRPVADLNVQELTNLGLVINERINHLKERIERLGGAALMAPPPSTQPTEASSSLPPLVPYANGAGMEGNKRMKVSTHQKGWFINMSTMTGDAGTSADVEGNTGVGTSARGDMMHLSN*

>LOC_Os01g18440.1 Oryza sativa subsp. japonica|M-type_MADS|M-type_MADS family protein

MARKKIVLDRIANDATRRATFKKRRRGLLKKASELATLCDVDACLVVYGEGDAEPEVWPSTEVAMNVLRQFRALPEMEQCKKMMNQEDFLRLRIGKLKEQLRKMDRDNHERETLILLHDALQGRLGTYESLSVEQLTSVDCLASARLKVITDRLVEIRAPNEDGQVLVPPPPPPPPALPAPPPPPAPMLPLAPPPTHVTPAMPLSSMPPPAFHGMNHHHHQNHFINHGGNDQNAWLMNVARNGGDLGALVYSAFASSSSSNTGGAGTSAAGAAAPGPDMMDLANPDMPGFGCPWDDDSAGPSFPPM*

>LOC_Os07g04170.1 Oryza sativa subsp. japonica|M-type_MADS|M-type_MADS family protein

MEREFCGEMESQICQFGTFLEVNNQHSATPKGKTIPVKCTDCLPGVASRSLTVIPLEVPHDVLLSAREAKTQYNSTFEHNLSETNFVTGTYNGAHHANVKDVPPDIYETEFRHQFPRIETSFSIIGAHFLSSMVRRGRRKGVRYIEEDRDRSLTLSKRRDGLFKLANDLSLLTDASVAICLHDSNKAQFFGAPSVKPVVDAFVSEAEPFADEQLKAKLTSMQSELVQLENEEEEKDKKTEESIQRFKEAQEESLGMGMAKHLFSRLEDLSHDDMRELLDVLLPLQQDFKKRLPPLRRGSKLQIGGSSAWAHQQPSCSRFLASHRPFTPLLPGGTSGVPMIPPPPVPGSPWSQIFPLRPPLFPSPELVPSQQLPPVSPPQNTVAPPPMHAPLVQQPLTNQSSAVPLLTQWQMRFGDQPPAEVQACTPVEQPQNDNAVHTPTFSDSFLLELLADVSDDGIATAEPLCSPPIDDQFLADIDWLAELDTIDGNL*

>LOC_Os01g11510.1 Oryza sativa subsp. japonica|M-type_MADS|M-type_MADS family protein

MPRRARRTGAAYVDDERERDITFFKRRNGLFKCASDLSILTGASVAVVIEDQNRSKFHAVGTPTVQAVVDAALSSDVEEAAAEARPVADEQLMERIAPLERELAWLKGEAAEKDETTKASKARFKMAQKKEENEEEGDTKKKKLFFSKADKLSSDEMNELLAEMLEIKKELNVRLPPLRRRGGKRPIQGSSVPPPPPPPPPPPQPEQQLQLPQWPNLSGPHNQLLPVAPPPFVADQPPPPPPPAAGGSLWIPELPPPPVEGSPWAGLLPLRPPRFAGMEPSFLESQQAPPPAQVSTQLAPLPLIREEAPLLQEPFLFADQAPVLAPLPAPLQMPVAETHLPLQAPLLQEPFLFSDHAPTLAPLPSPLQMPVAETHLPLQVQAPFMQEPFLFSDQAPVLAPPPTPLQMPVEAHMPLEAPWIQEPFLMPDQAPVHAPPPTPLLMPVGADHFPLEAPLFQESLIMADQKSVHALPPPPLQMPLEAHLPPAAQEYNQDLAVQQQPQEYENYDYMFENVGLSQAQPVAAGAGDAGFAAIGNDDNPFGYQQLVASPLYDGQIYFGSGVDNMGVPPAGDFGGVPEAALPEVEHASSSGWGNNITGDAGAWF*

>LOC_Os01g23750.1 Oryza sativa subsp. japonica|M-type_MADS|M-type_MADS family protein

MAGRKETVIKMAKELSVLCDVPVALVCAVGGAVEVWESEEGVLDRYRALPPEVRATRAHTHRGYLERELRARRAKLAKVREEGAFKSWGRDALSGIITAEEAPALLESIDAAIAAATARQEALALLDGGGLHLQHVPASASDAVAPVVGGHGVQVQYIGGSGGGGGSQQEMTPAADGDGARNADQYDDILPWDGNTFEAHNAHVMLPACGFQCTGDYRVDMDGYVWGAPDDANAYHGWPDEAMWCTDESCSCNAATATAVPAMYHPPTLDTVHGSFLAAPAQPLAFSTGADFINAPNDFLTVGVGGSFINVGDYSAQSLADEFHHLSDATNQLDQTHYPPFGGTGGAEPGDTQSHSWGDYYLAQSSANECQLLGVDGGDIHLDQTHCLGGAGGAEPGDTKSHNWGG*

>LOC_Os01g23760.1 Oryza sativa subsp. japonica|M-type_MADS|M-type_MADS family protein

MARRKIPIGLIAHRQKRAATYAKRKESLRKKAEELSTLCGVRVAFVCAGPVVPGGGGGAAGKEEVWESEEGVLAEYRALPPEARAQHAHRVYLEEEVGKERAKLARVRQDGAFPSWDAALDGITADEARALLESIDAARAAANARREALGLPDDGNGVDDDGGLDLQQQQEHVPPGGSDAVVVPVGHGVLQYTGSGGGNQMQTTPAADGINCADLYGAVPWDDTFQPQVMRTGDHFVPMDGYLWQAPGNGWPDLATGCTNESCSCNAAAAAAAMPAMYPPTLDTVHGSFLAAPAQPIPIAFSTSTDFIDAPNDFLTMGLCGGFTNVGDYSAAQPQSSADGGFQLGDTFAAEPGDTQSQNWGSFINVVSDDSAQCNCNAAIHLDQMYYLFGGTGGGEPSDTQSRHWGS*

>LOC_Os01g23770.1 Oryza sativa subsp. japonica|M-type_MADS|M-type_MADS family protein

MPRAKTPMGLIPFPKKRAATFARRKETVMKMAKELSVLCDAQVAVRAKRSLTHREYLRGELRKQRAKLAKVREEGAFKPWDDALDGIAEEETRKLHKYLSDKIEAARARMEAMGLQLGDVDDNGVNGDDGGGLDLQQHVPPSASDAKEFESVPVVHGGQYIGSSSGGGGGDIQMQTTPAADGISFAEHFLVAPRAQPLAFSTAGADFINAPNNFLTTGVSVSDYSVQSSGYGIGNQIDNAKQLLYQMQMQYPVGGTGGAEPSNTQTQSPDLRSFSNVVGDYSYTAAQSSANRLDQMHHPVGGRGSTGGAAADPSDTQSKNRGS*

>LOC_Os01g23780.1 Oryza sativa subsp. japonica|M-type_MADS|M-type_MADS family protein

MKMEEDATYGKMQESLMEEARELSILCGVDVALLCAGGPGTGDGDGGGAVSTAEVAVWESEEGVLASYRAILPRPEAHTLRECLELKLARERAKLAMLHQYGGGGAGAARVHGRGDGGRDREAGGAALGLLDDGNAVNGDGGGPDLLHIPIQPGAPDSVVVPVISHAQPIASSTGADFADAPNGFLAMDVGGSLIKSATTQRSARPMGSSSGTPTMANLDQIHYLVGGSAFSIDALESPTLLVRCKTLRIAIQFMLPLRHGRFRANVPSNHRRTETKS*

>LOC_Os01g67890.1 Oryza sativa subsp. japonica|M-type_MADS|M-type_MADS family protein

MVRTPTIIPLRPNYSPPGDGAAFRREPATYSRFPPAAARNECDLTFGQERKREGMKGKPPISRKIIYNHRDFVSMGERFVALCFIFTMSPCIDATLFVRVVLDFVRREETRRDEMPRTKLVLKLIENEKKRKATFKNRRDGLKQKVSQFATLCGVEALLICVAPAVAGGEVTTWPPDRAAVLDLIARLRATPPEKIRQLHNTQSQLRDDLDKQQRLLLKVQKCGADDVLTPWHCSLYDLSLDGLNALHDTLSETLDRAHRRIAALGGGHGHVHDDAASSSEFSVPAPAPHAVALPDNAFDFPFAPSNTGPVVGAHYFYPLHDTLPLPLPLPQQVPGQHPPCIAYQMPPPPCLAYQMPPPPPPSLAAAPFDQCMSATGFMDSNPYATHIMHGGSTAAGLLDDHGQIFSAGAGYDDDDILGHGFGFAAGTGYDLDPRMATADVWPMNTLNNIPNDGGIGFQLQNDLKWMLPGGSNGSNLQGGFQI*

>LOC_Os01g68560.1 Oryza sativa subsp. japonica|M-type_MADS|M-type_MADS family protein

MSRRKTSIALIANPQTRATTYKKRKAGLIKKAGELATLCDIPVAVVCAGPDGGAPTVWVSPEGGDAIERYRALPAEKRARHTHVAYLQEELDKERAKLARLRQKGRPGELDPPDAVLDGMSQDELQQLLASIDATLLATAKRREALGLLPGADDDADGGGRRRDADVAGTNSVGVHGYQHQEVHAPATCDPFHPYNAGVTLMQPGYNNAQYMGGHGAVDMSGYQLQMQMPGNGSNNHSRLAWGGFQPCNATFVQPVYGNLQCWYNNVVDGNGEPCDAIVPSAGDPYMDIAGNDVYGNQMQPAPAANGGWHDPGTWGYDGGEPCKAIVPSFGDPYMGIGVYGNQMQPAPAPAANGCWHNPAGTWGNDGEPCNAIVPSAGHPYIDIECDIDGNYIDTTVFDYQTTSTSDNFMDAPVQFIATGSDESIVTNVAGCDETEFSIDDLLQCSDASQHSSGLEELHYLSDLADGFDFGCNFDVLLD*

**#nucleotide sequences of MADS-box genes in *Solanum lycopersicum***

>Solyc01g087990.2.1 Solanum lycopersicum|MIKC_MADS|MIKC_MADS family protein

ATGGGTAGAGGAAAAATTGACATAAAATTGATTGAGAATCTAAATAATAGACAAGTAACATTTTCTAAGAGGCGTGCTGGCTTGTTGAAGAAAGCCGGGGAGCTTTCTGTTCTTTGTGATTCTGAAGTTGCTGTTATTATTTTCTCAAGTACTGGAAAGCTTTTTGAGTTTTCAAGTACTAGCATGAAACAGACGCTTTCCAGATACAACAAATGTGTAGCCTCGACAGATAATTCTGCCGTAGAAAAGAAGTCAGAGGACAACGAGCAGCCACAGTTGCAGCAGCAGACACATGTGCTGAAGCAGGAACAAAAAGAGGTGGACAGTCTTAAAGACGAACTCGCAAAGCTCAAGATGAAACAACAGCGGTTGTTAGGCAAGGACCTTAATGGTATGGGTTTGAATGAGCTACGGCTTCTTGAACATCAACTAAATGAAGGACTACTAGCCATAAAGGAGAGAAAGGAGGAATTGCTGATACAGCAACTAGAGTATTCTAGGAAACAGGAGGAGAGGTCTGCGCTGGAGTGTGAGACCTTACGTAGACAGGTAGAAGAGCTCCGAGGGTTATTTCCTTTAAGTGCTAGTTTACCGCCACCTTTTCTTGAATATGATCGCCCATTGGAAAAGAAGTATTCAATTTTAAAAGAGAGTAAGGAGAGTCTGGATTCCGACACTGCATGTGAAGATGGAGTAGATGATGAAGATTCCAACACAACTTTGCAATTGGGGCTTCCAACTATTTGTCGAAAGAGAAAGAGAACAGAGCAGGAATCTCCTTCAAGCAATTCAGAGAATCAAGTTGGCTCAAAGTGA

>Solyc01g093960.2.1 Solanum lycopersicum|MIKC_MADS|MIKC_MADS family protein

ATGGGGAGAGGGAGAGTGGAACTAAAGAGAATAGAGAACAAAATCAACCGTCAAGTGACATTTTCTAAGAGGAGGAATGGTTTGTTGAAGAAAGCTTATGAATTATCAGTGCTTTGTGAGGCTGAAGTTGCTCTCATCATCTTCTCTAGTCGTGGAAAGCTCTATGAGTTTGGTAGTGCAGGTATCACTAAAACCCTTGAGAGGTACCAACGTTGTTGCCTTAATCCTCAAGACAATTGTGGTGAAAGAGAAACACAGAGCTGGTACCAAGAGGTCTCTAAATTAAAGGCCAAGTTTGAAGCACTTCAACGAACTCAAAGGCACTTGCTTGGTGAAGATCTTGGAGCACTAAGTGTGAAGGAGTTGCAAAATCTTGAAAAACAACTTGAAGGTGCACTTGCACAAGCTAGACAAAGAAAGACACAAATAATGATGGAACAGATGGAGGAGCTTCGTAGAAAGGAGCGTCATCTTGGTGATGTGAACAAGCAGTTGAAGATTAAGGTTTCTCTTGAACTATCATCGTTTGAGGGTGAAGGACAAGGTGTTCCTTTTCCATGGAGTAATTGTAATGCATCTTTAGATGAAGCAGGAAGCAGCACCTTTCATGTCCACCATTCTCAATCAAATCACATGGACTGTGATTTACCTGATCCAGTTCTTCAAATAGGGTATCATCAGTATATGGCTGCAGATGGAGCCTCAGGGTCAAGGAACATGGCTGTTGAGAGTAACATTATCCATGGTTGGGGTCTTTAA

>Solyc01g105800.2.1 Solanum lycopersicum|MIKC_MADS|MIKC_MADS family protein

ATGGAACAAATAACTATTCAATTAAAAAGCTCCAACTCAAACTCGCATGAAGAAATACAATTACTCCTTTGTAACCAAAAATACCCTAATTACTACTTTTTCTCTTTCTATTGTAATAACATACCTTTTTTTTCTTTCTTCAGCTCACAACTTCAGATCTCTTCTCCATTTCTTGCAAATTTCAGTAAGGAAATGGTGAGACAAAAAATTCAGATCAAGAAGATAGACAATTTGACAGCAAGACAAGTGACATTTTCAAAGAGAAGAAGAGGGCTTTTCAAAAAAGCTCAAGAGCTTTCAACTCTTTGTGATGCTGATATTGGACTCATTGTTTTCTCTGCTACTGGAAAACTTTTTGAGTATTCAAGCTCCAGCATGATGCAACTGATTGAGAAGCACAAGATGCAGTCAGAAAGGGATGGTATGGATAATCCAGAACAACTGCATTCTTCTAACATTCTGAGCGAGAAGAAAACCCATGCAATGCTTAACAGGGACTTTGTGGAGAAGAATCGGGAGTTAAGGCAACTACATGGAGAAGAGCTGCAAGGACTTGGTTTGGATGAATTAATGAAATTGGAGAAATTAGTCGAGGGAGGAATAAGTCGTGTCCTCAAAATTAAGGGTGACAAGTTTATGAAAGAGATCAGTTCCCTCAAGAAAAAGGAAGCTAAACTCCAGGAAGAGAATTCACAGTTGAAAAAGCAATCACAAGCAAGATTGAATGAAGAAGGGCAAAATGTAATTGAGCAAGGACATTCAGCAGACTCCATCACGAACAATCGCAGCTTAGTCAATGACTCAGATACTAGTCTCAAGTTATGCTTGGCTTTTCCTTAG

>Solyc02g065730.1.1 Solanum lycopersicum|MIKC_MADS|MIKC_MADS family protein

ATGGGGCGGGGTAGGGTGGAGATGAAGCGTATCGAAAATAAAATAAGCAGACAAGTTACATTCTCAAAGAGACGATCCGGTTTGTTGAAGAAAACCAACGAGATCTCTGTGCTATGTGATGCTGAGGTGGCATTAATTGTTTTCTCTTCAAATGGAAAACTATTTGAGTACTCTACTCAATCAAGCATGGAAAATATATTGGAAAGATATGAAAATTACTCATACGAGGAGATGAACTTGAATACAACTTATAAGGAAAATTGGACTCTTGAGTACCCAAAGCTCATGGCAAGAGTTGAACTTCTGCAAAGAAATATAAGGCATTTTATGGGAGAAGATCTGGACGCCTTTAATCTGCGTGAATTTCGGGGTTTAGAGAAACAGCTCGATACAGCTCTAAAGCGAGTGCGATCTAAGAAGAACCAACTGATGCACGAGTCCATTTCCCAGCTGCAGAAAAAGGAAAAAGAACTGCAACAGCGAAACAACTTAATTTCTAACAAGCTTAAAGAAAATGAGAAGAAGCAAATTGTGCAAACAAATCCAGGCCAAAGCTCTACTATGACTTTCTTGCTACAATCTCCCACAGTCACTAACCAAACAATTGGCGGTCCTTCTCAAGCAACGGATCAAAGTCAAAATCGCGATGGTTACAATAGCTTGATGCCTCCATGGATGTTCCACCATGTCCACAACAAAGGATGA

>Solyc02g071730.2.1 Solanum lycopersicum|MIKC_MADS|MIKC_MADS family protein

ATGGACTTCCAAAGTGATCTAACCAGAGAGATCTCACCACAAAGGAAACTAGGAAGGGGGAAAATTGAGATCAAAAGGATCGAAAACACGACGAATCGACAAGTAACATTCTGCAAGAGGCGCAATGGTTTGCTTAAAAAGGCTTATGAATTGTCTGTGCTCTGTGATGCTGAGGTTGCTTTGGTTGTCTTCTCAAACAGAGGCAGACTCTATGAGTATGCCAACAACAGTGTGAAAGCAACAATCGAGAGGTACAAGAAAGCATGCTCAGATTCCTCAAACACTGGTTCAGTATCCGAGGCCAATGCTCAGTATTACCAGCAAGAAGCCTCCAAACTGCGCGCACAAATTGGAAATCTGATGAACCAAAACAGGAACATGATGGGTGAAGCTCTTGCTGGAATGAAACTCAAAGAACTGAAGAATCTGGAGCAAAGAATTGAAAAAGGGATTAGCAAAATCCGATCCAAAAAGAATGAGCTGTTGTTTGCTGAAATTGAGTATATGCAGAAGAGGGAAGTTGATTTACACAACAACAATCAGTACCTGAGAGCAAAGATTGCTGAAACTGAGAGAGCTCAGCATCAGCATCAGCAGATGAACTTGATGCCAGGGAGTTCATCAAACTATCATGAGCTTGTGCCTCCACCTCAGCAATTCGATACTCGAAACTATCTACAAGTTAATGGATTGCAAACCAACAACCATTACCCTAGACAAGACCAACCCCCTATTCAACTAGTCTAA

>Solyc02g084630.2.1 Solanum lycopersicum|MIKC_MADS|MIKC_MADS family protein

ATGGGCCGTGGAAAAATTGAGATCAAGAAGATTGAAAACTCGACAAACAGGCAGGTCACTTACTCCAAGAGAAGAAACGGTATTTTCAAGAAAGCTAAAGAACTTACTGTTCTTTGTGACGCTAAGATCTCTCTCATCATGCTATCAAGCACCAGGAAGTATCATGAGTACACAAGCCCAAACACTACGACAAAAAAGATGATTGATCAGTATCAGAGTGCACTTGGAGTTGATATCTGGAGCATTCACTACGAGAAAATGCAAGAAAACTTGAAGAGATTGAAAGAGATCAATAACAAGCTAAGAAGAGAGATAAGGCAGAGAACAGGGGAAGACATGAGCGGACTAAATTTGCAGGAACTATGTCACTTGCAGGAGAACATCACTGAATCTGTTGCTGAGATTCGTGAACGAAAGTACCACGTGATCAAGAATCAAACAGACACCTGCAAGAAGAAGGCGAGGAACTTAGAAGAGCAAAATGGAAACCTTGTACTTGACTTGGAAGCAAAATGTGAAGATCCAAAGTATGGTGTTGTGGAAAATGAGGGGCATTACCACTCTGCTGTGGCATTTGCGAATGGAGTACACAATCTTTATGCTTTTCGCCTACAACCATTGCACCCCAATCTTCAAAACGAAGGAGGATTTGGTTCTCGTGATCTACGTCTCTCCTGA

>Solyc02g089200.2.1 Solanum lycopersicum|MIKC_MADS|MIKC_MADS family protein

ATGGGTAGAGGAAGAGTTGAGCTGAAGAGGATAGAAAACAAGATAAATAGACAAGTCACTTTTGCAAAGAGGAGAAATGGATTGCTCAAAAAAGCTTATGAACTATCTGTGCTTTGTGATGCTGAAGTTGCTCTACTCGTTTTCTCTAATCGTGGAAAACTCTATGAATTCTGCAGCACAAACAATATGCTCAAAACACTTGATAGGTACCAAAAGTGCAGCTATGGAACATTGGAAGTCAATCGATCAATCAAAGATAATGAGCAAAGCAGCTATAGGGAATACTTGAAACTCAAAGCCAAATATGAGTCGCTGCAGCGATATCAAAGACACCTTCTTGGAGATGAGTTGGGGCCTCTGACTATAGATGATCTTGAGCATCTTGAAGTCCAACTAGATACTTCCCTCAAACACATTAGGTCCACCAGGACACAAATGATGCTTGATCAGCTTTCTGATCTTCAAACTAAGGAGAAATTGTGGAATGAGGCTAACAAGGTTCTTGAAAGAAAGATGGAAGAAATATATGCTGAAAACAACATGCAACAAGCATGGGGTGGTGGTGAGCAAAGTCTCAATTATGGTCAGCAGCAACATCCTCAATCTCAGGGTTTCTTCCAACCTCTAGAGTGCAACTCTTCCTTGCAAATTGGGTACGATCCAATAACAACTTCAAGCCAAATAACAGCAGTAACAAATGCCCAAAACGTGAATGGTATGATACCTGGTTGGATGCTGTGA

>Solyc02g089210.2.1 Solanum lycopersicum|MIKC_MADS|MIKC_MADS family protein

ATGGGAAGAGGTAGGGTAGAGTTGAAACGGATCGAGAACAAAATAAGCAGACAAGTAACATTCTCAAAGAGACGATCTGGATTATTGAAGAAAGCTAATGAGATCTCAGTATTATGTGATGCTGATGTTGCATTGATTGTGTTTTCTACCAAAGGCAAACTTTTCGAGTATTCCTCAAATGACTCAAGTATGGAAAGTATTCTTGAAAGATATGAAAGATGCTCATATGCAGAGAGACAGATGAATGCTAATGATTCTGATCCCAAGGAAAATTGGAGTGTGGAGTATCCGAAGCTCATGTCAAGAATTGAACTTTTACAAAGAAATATAAGGCATTACATGGGTCAGGATCTGGACCCTCTCAGTTTGCGTGAGCTCCAGAGTATAGAGCAACAGATTGATACTTCATTAAAGAGAATTAGAAGCAGGAAGAATCAACTGATGCACGAGTCCATTTCTGAGCTGCAGAAAAAGGAGAAAGCGCTCCAAGAACAAAACAACTTGATTACTAAGAAGCTAAAAGAAAATGAGAAGACACAACCCAACTCATCAGGCCAAAACTCAGCAACAGTTCATGTGTTTCCATCACATTCTCACCACCAACTTCCTAACCTTACAATTGGGGGGGCTTTTGGAGGAATGAACAGAGATGGATCGGGTCAGGCTCATCACTATCCGGGTTCAAATAACAATAATAATAATTCCTCATTGATACCGCCATGGATGCTCCGCCACGTCAGCAACGAAGGATGA

>Solyc02g091550.1.1 Solanum lycopersicum|MIKC_MADS|MIKC_MADS family protein

ATGGGAAGGGGAAAGATAGTGATCCGAAGGATCGATAACTCGACGAGTAGACAAGTTACGTTCTCAAAGAGAAGGAATGGATTGTTGAAGAAAGCTAAGGAGCTTGCGATTCTTTGTGATGCTGAAGTTGGATTGATTATTTTCTCTAGTACTGGGAAGCTCTATGAGTTTTCTAACACAAGCATGAAATCAGTTATTGAACGATACAACAAAACGAAGGACGATTGTCAGCAGTTGCACAATCCAGTTTCAGAACTCAAGTTGTGGCAGAGGGAGGCAGAGATTTTGCGGCAACAACTACAGGACCTGCAAGATAATCATCGGCAATTATTGGGAGAGGAGCTCAGTGGTTTGGGCGTAAAAGAACTAACTAATCTGGAAAATCAACTGGAAATGAGCTTAAAGGGCATCCGTATGAAAAAGGAGCAAATATTAAAGGACGAAATTCAAGAGCTAACTCGAAAGGGGAGCATTATACATCAAGAAAATATGGAACTCTACAAGAAGGTAAATCTCATTCGACAAGAAAATGCAGAATTGTATAAAAAGGCTTATGGTGCAAGAGATGCTAATGCAGTGAATGGAAACATCAACTATCCGTATCGCTTCACTGTGAGTAGAGAAGTTCAGGCACCCATCCATCTGCAGCTAAGCCAGCCTGAGCCACAATATTTTGAGATGCAAGCAGGAACATCTGATTCAAGGTAG

>Solyc03g006830.2.1 Solanum lycopersicum|MIKC_MADS|MIKC_MADS family protein

ATGAAAAGAATAGAAAATTCAACGAGCAGGCAAGTGACGTTTTCGAAAAGACGAAATGGACTTACGAAGAAAGCTTATGAATTATCAGTTCTTTGTGATGCTGAAGTTGCTTTCATTATTTTCTCACATAAAGGAAGACTTTATGAATTTGCTAGCTCCAACATGCAGAAGATAATTGAGAGATACCGAGGACGTGCAAGAGAAACAACGACGGTGGACAAAAGCACTGAACTCGAGCACTACATGGAGAACTTGAAGCATGAAACAGCTAATATGGCGAAGAAGATAGAGATCCTCGAAATTTCTAAACGGAAGCTAATGGGGCAAGGATTAGGGTCATGTTCAATGGATGAACTAGAAGACATTGACAGCCAACTGGAGAGGACCCTCAAAATTATCAGGGCTAGAAAGACTCAATTGTTCAAAGAGGAAATAGAAAGTCTAAAAGCAAAGGAGAGACTATTGCTCCAACAAAATGCAAGTTTACGTGAAAAGTGCGGGCTTAGGCCAATGCTATCAGAGTCAGCATCTGCACCCGAACCCATACCAGCACCACCATCAACACCACCAGCTCAATCAAAAGAAAGGGGAAATTGTAGCCAAAGTACAAAGAGTTGGGAAGTGGAGACTGAATTGTTTATTGGCCTTCCTCAAACGCGCTGCTTATAG

>Solyc03g019710.2.1 Solanum lycopersicum|MIKC_MADS|MIKC_MADS family protein

ATGGGGAGAGGAAAAGTTGAATTGAAGAGAATAGAGAATCAAACAAATAGGCAAGTTACCTTCTCCAAGAGAAGAAATGGTTTACTTAAAAAAGCTTATGAACTCTCTATTCTATGTGATGCTGAAGTTGCCCTTCTCCTTTTCTCTCCTTCTGGCAAAGCTTATCATTTCGCGAGCCACGACATCGAAAGGACTATTTTAAGGTACAAGAATGAAGTTGGATTGTCCAAAAATAGTGATCAAGGCCCCAGAGCTATGGAGGTTTGGAGAACTAAGATTGATGACATGACAAGAACAATACATGAACTTGAAGCTAGAGATAAGCATTTTGCTGGAGAAGAGTTATCAAATCTTGGTATGAAAGAATTGAAGCAGTTGGAGCGTCAACTCAGAGTTGGAGTTGAACGCATTCGATCTAAAAAGCATAAAATCCTTCATGAGGAGAACATCCATCTTCAAAAGCAAGTAAAATTATATGAAGTAGAAGGGAGCTCAAGGATTCTCGATACAAATCCAAGGATGAGGATTATATGA

>Solyc03g114830.2.1 Solanum lycopersicum|MIKC_MADS|MIKC_MADS family protein

ATGGGTAGAGGAAGAGTACAATTGAAGAGAATTGAGAACAAAATTAATCGTCAAGTTACTTTTTCAAAGAGGCGATCTGGTTTGCTTAAAAAAGCTCATGAGATCTCTGTGCTTTGCGATGCTGAAGTTGGACTCATTGTTTTCTCAACTAAAGGAAAACTCTTTGAGTATTCTACTGACTCTTGCATGGAAAGGATTCTTGAAAGGTATGAAAGGTACTCATATGCTGAAAGGCAGCTTAATGCTACTGATATTATAACCCCGGGTAGCTGGACTTTGGAACATGCTAAGCTTAAGGCCAGACTTGAGGTTTTGCAAAGAAACCAAAAGCATTATGCAGGAGAAGAGTTGGACACATTGAGTATGAAAGAGCTTCAGAATCTGGAACACCAGCTCGATTCTGCTCTTAAGCACATTCGCTCTAGAAAGAACCAATTGATGCATGAATCCATTTCTGAGCTTCAAAAGAAGGACAAGGCATTGCAAGAACAAAACAACAATCTTTCAAAGCAGGTTAAGGAAAGGGAGAAAGAGATGGCCCAACAGACTCCGTGGGAGCAACAGAGTCATGATCATCTCAATTCATCTTCGTTTGTTTTGCCACACCCCTTTAACAATCTTCACATAGGGGAAGCATACCCAAATGCAGGAGACAATGGAGAAGTAGAAGGATCATCGCGGCAACAACAACAAAACAGTGCTTCTGTGATGCCTCCATGGATGCTTCGCCATCTCAACGGTTAA

>Solyc03g114840.2.1 Solanum lycopersicum|MIKC_MADS|MIKC_MADS family protein

ATGGGAAGAGGAAGAGTTGAGCTTAAGAGAATAGAAAATAAAATAAATAGGCAAGTCACTTTTGCTAAGAGAAGAAATGGACTTCTTAAAAAAGCTTATGAACTTTCTGTTCTTTGTGATGCTGAAGTTGCCCTTATAATCTTCTCTAATAGGGGTAAACTCTATGAATTTTGCAGCACTTCAAGCATGGTGAAAACAATTGAAAAGTACCAACGTTGCAGCTATGCTACTTTGGAAGCCAACCAATCAGTTACTGATACTCAGAATAACTACCACGAATATCTGAGGCTAAAAGCTAGAGTTGAGCTCCTCCAACGATCTCAGAGAAACTTTCTTGGTGAAGATTTGGGCACGTTAAGCTCGAAGGACCTTGAGCAGCTTGAGAATCAATTAGAGTCTTCCTTAAAGCAAATCAGGTCAAGGAAGACACAATTCATGCTGGATCAGCTTGCAGATCTTCAACAAAAGGAGCAAATGCTTGCAGAATCTAATAGATTACTCCGTAGAAAGTTAGAAGAAAGTGTAGCTGGATTTCCACTTCGATTGTGTTGGGAAGATGGAGGTGATCATCAACTTATGCATCAACAAAATCGTCTCCCTAACACAGAGGGTTTCTTTCAGCCTCTTGGATTGCATTCTTCTTCTCCACATTTTGGGTACAATCCTGTTAATACAGATGAGGTGAATGCAGCGGCAACTGCACACAATATGAATGGATTTATTCATGGATGGATGCTTTAA

>Solyc04g005320.2.1 Solanum lycopersicum|MIKC_MADS|MIKC_MADS family protein

ATGGGAAGAGGTAAGGTAGAATTGAAGAGAATAGAAAATAAGATAAACAGGCAAGTTACTTTTGCTAAGAGAAGAAATGGATTACTCAAAAAAGCTTATGAGCTTTCTATTTTGTGTGAAGCTGAAGTTGCTCTTATCATTTTCTCTAATAGAGGCAAACTCTATGAATTTTGCAGTACCTCTAGTATGTCTGATACACTGGAGAGATACCATAGATGCAGCTATGGTGACCTTGAAACTGGCCAGTCTTCAAAGGATTCACAGAATAACTACCAAGAGTATATGAAGCTGAAAGCAAGAGTTGAAGTGCTACAACAGTCACAAAGGCATATACTTGGAGAGGACTTAGGACAATTAAACACAAAAGATTTGGAACAGCTTGAGCGTCAACTGGATTCATCTTTGAGGCTAATAAGATCAAGAAGGACACAAAACATGCTTGATCAACTTTCTGATCTTCAACAAAAGGAACAATCTCTTCTTGAAATCAACAGATCCTTGAAAACAAAGTTGGAAGAAAACTCTGTAGCACATTGGCATATCACTGGAGAGCAAAATGTACAATTCAGACAACAACCTGCTCAGTCAGAGGGGTTCTTTCAGCCTTTACAATGCAATACTAATATAGTGCCAAACAGGTACAATGTGGCTCCATTGGATAGTATAGAACCATCAACACAGAATGCTACTGGAATTTTACCAGGATGGATGCTTTGA

>Solyc04g078300.2.1 Solanum lycopersicum|MIKC_MADS|MIKC_MADS family protein

ATGGGGAGAGTAAAGCTTCAAATCAAGAAAATAGAGAATACAACAAATAGGCAAGTCACTTTCTCCAAAAGAAGAAATGGTCTTATCAAGAAAGCTTATGAACTTTCTGTACTTTGTGATGTTGATGTTGCTCTCATCATGTTCTCTCCCTCTGGTCGAGTTAGTACTTTCTCTGGAAATAAAAGCATTGAAGATATTATGGCACGTTATGTGAATCTTCCGGAGCATGATCGAGGAAGGCTACATAACCAAGAGCATCTCCAAAGGGCTATTGCTAAGCTAAAATGTGAAGCAGATCGAACTTATCAAGCACCAAGTAGCCCATCAAGTGTTGATTCCCACATAGAGGAATTTCAACAAGAAATTATTAGATACAAAACTCAAGTAGAAGACATGGAAAGGCGACTAAGAATGTATGAAGGTGGTTTTTGTGAAATTACCACAGTATGTGAGGCACAATATAGGGAAGAAATACTCCAAGAGACACTAAAACAAGTTCAAGCTCGCAAACAAGTATTGGAGGAAAATTATCACTCTCCACAAACACAAAACACTACACAGCCACAGATGGATTTTTCAGGACAAAATGTGAACATGGTGAATAATGTGGCAACAAGTGATGCAATTGCAAATAGTACATTTATGGATTGGGTTCCACACTCACAAAGAGATCCTCATGTCCAAATTCTCAATTTTCTTGATTCCAGTGGCCTTCTTCCCTTCAGAGATGAAGCTGATCAACACATGTTACCACCATCTTTAAATCAACTTCACGGTGTGAATGTCCCTGCTGGAACAGATCATTTGAGTTCAAATAGTCGATTTGATCAAAATAATCCGCCTCGTCCATCATCGTTCGATGGCATAATCGATGTCAATAACGCTCCGTGGCCACCGTTATATACAACAGGGGATGATCCATTTCCAGTGTCACAACCTAGAGAAAGAGCAATTCTTGAACTCTTCTTGTCTCAACTCACTCCAGTGAACCAAGATCACATATGA

>Solyc04g081000.2.1 Solanum lycopersicum|MIKC_MADS|MIKC_MADS family protein

ATGGCTCGTGGTAAGATCCAGATCAAGAAAATAGAAAACCAAACAAATAGACAAGTGACTTATTCAAAGAGAAGAAATGGGCTATTCAAGAAGGCTAATGAACTTACTGTTCTTTGTGATGCTAAAGTTTCAATTGTTATGATTTCTAGTACTGGAAAACTTCATGAGTTTATAAGTCCCTCTATCACGACCAAACAATTGTTCGATCTGTACCAGAAGACTATTGGAGTTGATATTTGGACTACTCACTATGAGAAAATGCAAGAGCAGCTAAGGAAGCTAAAGGATGTGAATAGGAATCTACGAAAAGAGATCAGACAGAGGATGGGAGAAAGCCTAAATGATCTGAACTATGAACAGTTGGAAGAACTCATGGAAAATGTGGACAATTCTCTGAAGCTTATTCGTGAAAGAAAGTTTAAGGTGATTGGCAATCAGATTGAAACTTACAGGAAAAAGGTTAGGAATGTGGAAGAAATAAATAGAAATCTCCTACTTGAATTTGATGCAAGACAAGAGGATCCATATGGTGGATTAGTTGAGCATGATGGAGACTACAATTCCGTGCTTGGATTTCCAACTGGAGGGCCTCGTATATTAGACTTACGCCTTCAACCCAACAACAATTATCATAATCATCTTCACAGTGGAGGTGGCTCTGATATTACTACTTTTGCTCTAGGTTGA

>Solyc05g012020.2.1 Solanum lycopersicum|MIKC_MADS|MIKC_MADS family protein

ATGGGTAGAGGGAAAGTAGAATTGAAGAGAATTGAGAACAAAATAAATAGACAAGTTACCTTTGCAAAGAGAAGAAATGGACTCCTAAAGAAAGCTTATGAACTTTCTATACTTTGTGATGCTGAAATTGCTCTTATTATTTTCTCTAGTCGTGGCAAGCTTTATGAATTTTGCAGCAATTCAAGTATGTCCAAGACATTGGAGAGATACCACAGATACAATTATGGTACACTTGAAGGAACCCAAACTTCATCAGATTCACAGAACAACTACCAAGAGTATTTGAAGCTTAAAACAAGAGTGGAAATGTTACAACAGTCTCAAAGGCATTTGCTAGGTGAGGATTTGGGACAATTGGGCACAAAAGACTTGGAACAGCTTGAACGTCAATTGGATTCATCATTGAGGCAAATTAGGTCAACAAAGACACAACACATTCTTGATCAACTTGCTGAACTTCAACAAAAGGAACAATCTCTTACTGAAATGAACAAATCTTTGAGAATAAAGTTGGAAGAACTTGGTGTTACCTTTCAAACATCATGGCATTGTGGTGAGCAAAGTGTACAATATAGACATGAACAGCCTTCTCATCATGAGGGATTTTTTCAACATGTAAATTGCAATAATACATTGCCTATAAGTTACGGATACGATAATGTACAACCCGAAAATGCAGCACCATCAACACATGATGCTACTGGAGTTGTACCTGGATGGATGCTTTGA

>Solyc05g015750.2.1 Solanum lycopersicum|MIKC_MADS|MIKC_MADS family protein

ATGGGAAGGGGTAGGGTTGAGCTAAAGAGAATAGAGAACAAGATCAACAGGCAAGTGACCTTTGCTAAGAGAAGAAATGGGCTTTTGAAGAAAGCTTATGAGCTTTCAGTTCTTTGTGATGCTGAGGTTGCTCTAATCATCTTCTCTAATAGGGGAAAACTCTATGAGTTCTGCAGTAGTTCTAGCATGCTAAAGACGTTGGAGAGGTACCAGAAGTGCAACTATGGAGCACCTGAACCGAATATATCAACACGAGAAGCACTGGAAATTAGTAGCCAGCAGGAGTACTTGAAGCTTAAAGGACGTTACGAAGCATTGCAGCGATCACAGAGGAATCTTCTTGGTGAAGATCTTGGTCCTTTGAACAGCAAAGAACTTGAATCACTTGAGAGACAACTTGATATGTCGCTCAAACAGATCAGATCAACTCGGACTCAGTTAATGTTGGATCAACTTACAGATTATCAGAGAAAGGAACATGCATTGAACGAAGCCAACAGAACCTTGAAACAAAGGTTGATGGAAGGAAGCCAACTAAATCTGCAGTGGCAGCCAAATGCACAAGATGTGGGCTATGGCCGGCAAACAACTCAAACTCAGGGCGATGGCTTCTTTCATCCTTTGGATTGTGAACCTACTTTGCAAATTGGGTATCAGAATGATCCAATAACAGTAGGAGGAGCAGGGCCTAGTGTGAATAACTACATGGCTGGCTGGTTGCCTTGA

>Solyc05g056620.1.1 Solanum lycopersicum|MIKC_MADS|MIKC_MADS family protein

ATGGGAAGAGGAAAAGTTGAATTAAGAAAAATAGAGAATAAAATAAATAGACAAGTAACATTTTCAAAGAGAAGAGGTGGATTAGTGAAAAAAGCTCATGAAATTTCAGTTTTATGTGATGCTGAAGTTGCTTTAATTGTTTTCTCTCAAAAGGGAAAAATCTTTGAGTATTCTTCTGATTCATGTATGGAACAAATTCTTGAACGATATGAAAGATACTCATATGCAGAGAGACGTTTGCTTGCAAATAATTCTGAATCACCGGTGCAGGAAAACTGGAGCTTGGAATATACTAAACTCAAGGCTAGGATTGATCTCCTTCAAAGGAACCACAAGCATTATATGGGAGAAGATCTTGATTCAATGAGCTTGAAGGACTTGCAAAACTTGGAACAACAGCTTGATTCTGCTCTTAAGCTAATTCGATCGAGAAAGAACCAACTCATGCATGAATCAATCTCTGAACTGCAGAAAAAGGAAAGAGCTATCCTAGAGGAGAATAACATGCTAACCAAGAAGATTAAGGAGAAGGATAAGATAGTAGAACAGCAAGGTGAATGGCACCAGCAAACTAATCAAGTTTCTACTTCAACATCTTTCCTCTTACAACCACATCAATGCCTAAATATGGGAGGTAATTACCAAGATGAAGTAGCAGAAGCAAGGAGGAATAATGAGCTTGACCTAAATCTTGATTCATTATATCCACTTTACAACATGAATAAACATCTATGA

>Solyc06g059970.2.1 Solanum lycopersicum|MIKC_MADS|MIKC_MADS family protein

ATGGGGAGAGGTAAAATAGAGATAAAGAGAATAGAAAACACAAACAACAGGCAAGTAACTTATTCAAAAAGAAGAAATGGTATAATAAAGAAAGCTAAAGAAATTACTGTTCTTTGTGAAGCTAAGGTTTCACTTATAATCTTTGCTAGTTCTGGAAAGATGCATGAATATTGTAGCCCTTCTACTACGATAAGTGATATGTTGGATGGTTATCAAAAAGCTTCTGGGAGGAGACTATGGGATGCTAAGCATGAGAATTTGAGTAATGAAATTGATAGAATCAAGAAAGAGAATGACAGTATGCAGGTTAAGCTCAGGCACCTCAAAGGAGAAGATATCAATCAACTTACCCATAAAGAGCTTATAATTATGGAAGAAGCCTTACAAAATGGACTTTCTAGTATCAGTGCCAAGCAGTCTGAAATCTTGAGGATGGTCAGGAAAAATGATCAAATTCTGGAGGAGGAAAATAAGCAACTTCAATATGCTTTGCACCAAAAGGAGATGGGAGCCATTGGTGGAAGTGGAAATATGAGAGGAATTCATGAAGAAGTGTATCATCAAAGAGAAAGGGATTATGAGTACCAAATGCCATTTGGCCTACGAGTTCAGCCAATGCAGCCAAATCTACATGAAAGAATGTAA

>Solyc06g069430.2.1 Solanum lycopersicum|MIKC_MADS|MIKC_MADS family protein

ATGGGAAGAGGAAGAGTCCAGTTGAAGCGAATAGAGAACAAAATTAACCGTCAAGTTACCTTCTCGAAACGTCGATCTGGTTTGCTGAAGAAAGCCCATGAGATCTCTGTGCTTTGTGATGCTGAGGTTGGTTTGATTGTTTTTTCTACTAAAGGAAAACTCTTTGAATATGCCAACGATTCCTGCATGGAGAGGATACTTGAAAGATATGAAAGATACTCATTTGCTGAGAAACAGCTTGTTCCTACTGATCATACCTCCCCGGTAAGCTGGACCCTTGAACATGCAAAACTTAAGGCCAGACTTGAGGTTCTGCAGAGGAACCAAAAGCATTATGTGGGAGAAGATTTGGAGTCCTTAAGTATGAAGGAACTTCAGAATCTGGAGCACCAGCTTGATTCAGCTCTTAAACACATTCGATCAAGAAAGAATCAATTGATGCATGAGTCCATTTCTGTGCTTCAAAAAAAGGACAGAGCATTGCAGGAGCAAAACAACCAGCTTTCGAAGAAGGTGAAGGAGAGGGAGAAAGAGGTGGCACAGCAAAATCAGTGGGAAATCAACTCATCTTCATTTGTTTTGCCACAACAACTGGACTCTCCTCACCTTGGGGAAGCATACCAGAGTACTAATGTAATAGATAATGGGGAAGTGGAAGGAGGTAGTTCTTCACAGCAGCAAGGTGCAGCTAATAATACTGTGATGCCACAATGGATGCTTCGTCATCTTAATAATTAA

>Solyc07g055920.2.1 Solanum lycopersicum|MIKC_MADS|MIKC_MADS family protein

ATGGTTTTTCCTATTAATCAGGAATTACTTGTCGATGAGTCGTCTTCTCAGTTGAGAAAAACAAGTGGAGGAACTGGTGGAGGAGGTAGAGGGAAGATTGAAATTAAAAGGATCGAAAATACGACAAATCGACAAGTTACGTTCTGCAAGCGTAGAAATGGGCTATTGAAAAAAGCTTATGAACTTTCTGTTCTTTGTGATGCTGAAGTTTCACTAATTGTATTTTCCAGCCGCGGCCGTCTCTATGAATATGCCAATAACAGTGTTAGGGCAACTATTGATAGGTACAAGAAACACCATGCTGATTCCACTAGTACTGGATCTGTTTCTGAAGCTAACACTCAGTACTACCAGCAAGAAGCATCCAAACTGCGACGACAAATTCGAGATATACAGACTTATAACAGGCAAATAGTTGGAGAGGCATTGGGCAGTTTAAGCCCTAGAGACCTCAAGAATTTGGAAGGGAAACTTGAAAAGGCCATTGGTAGAGTCCGTTCCAAAAAGAATGAATTGCTTTTCTCAGAAATAGAGCTCATGCAAAAGAGGGAGATTGAGCTGCAGAACGCCAACATGTATCTACGCATAGCAGAGGTAGAGAGAGCACAAGAGCAAATGAACTTGATGCCTGGAGGCGGAGGCGGAGGCGGAGGCGGAGGAGGAGGAGGATCTGATCATCAATACCATCATCAGCCAAATTACGAAGATGCTCGCAATAACTTCCTGCCTGTAAATCTCCTGGAACCAAATCCTCATTACTCTCGTCGCGACAATGGTGACCAAACTCCTCTCCAGCTTGTCTGA

>Solyc08g067230.2.1 Solanum lycopersicum|MIKC_MADS|MIKC_MADS family protein

ATGGGAAGAGGAAAGATAGAGATAAAGAGAATAGAAAACTCAAGCAATAGACAAGTAACATACTCAAAGAGAAGAAATGGGATCTTGAAAAAAGCTAAGGAAATTAGTGTTCTTTGTGATGCTCATGTTTCTGTTATCATTTTTGCTACTTCTGGAAAAATGCATGAATTCTCGTCTACTTCTTTGGTTGATATTTTGGATCAATACCACAAGCTTACTGGAAGAAGATTGTGGGATGCTAAGCATGAGAACTTGGACAATGAAATCAACAAAGTCAAGAAAGACAATGACAACATGCAAATAGAACTCAGGCACCTAAAGGGTGAAGATATATCATCTTTGAATTATAGAGAACTCATGATATTGGAAGATGCACTTGAAAATGGACTCACTGGTATCCGTGAAAAACAGAATGAGTTTATGAGGATGATGAGGAAAAAGACTCAAAATATGGAGCAGGAGCAAGATCAACTTAACTGTCAATTGAGACAACTAGAGATAGCAAGCATGAATAGGAACATGGGAGAAATAGGGGAAGTGTTTGAGCAGACAAGGGAGAATCATGATTATGGGCAAATGCCTTTTGCTTTCAGAGTCCAACCAATGCAGCCTAATTTGCACCAAAGGTTCTAA

>Solyc08g080100.2.1 Solanum lycopersicum|MIKC_MADS|MIKC_MADS family protein

ATGGTGAGAGGAAAAACTGAGTTGAAAAGAATTGAAAATGCAACAAGTAGACAAGTGACCTTCTCAAAAAGAAGAAGTGGGCTTCTAAAAAAAGCATTTGAGCTTTCAGTTTTATGTGATGCTGAAGTTGCTCTTATTGTTTTCTCTCCAAAAGGAAAGCTTTATGAGTTCTCAAGTTCCAGTACAAACAAGACAATAGAACGCTATCAGAAGAATGAGAAGAGCCTGGGACGACTCAACAGAAAATTAACTGATCAACTAACCACTGAGCATTTGAAAGAGGAAGTTGCAACCATGACTAGAAAGCTTGAATTTCTTGAAGACTCTAAGAGAAAACTTTTAGGACATGGTCTAGAATCTTCCACCTTTGATGAACTTCAAAAGGTAGAAGAACAGTTGGAAAAAAGTTTAAGCAACATTAGGGCAAGAAAGAATCTGTTATTCAAGGAACAGATTGCTCAACTGAAGGAAGAGGAAAAAATTCTATTGAAGGAAAATGTAGACTTGAAAAAAAAGTGTCAGGTGCTACCATTGACTTTAACACCAGTTCCTCTAGTAGAGAAAGATGTTGAAAGACAAATAATGGAAGTTGAGACAGAGCTCTTTATAGGACTTCCAGAGACTAGAAAAAGTTCATATTGTCCTAATTTAAACACACTACCTACATTACTTTAA

>Solyc10g080030.1.1 Solanum lycopersicum|MIKC_MADS|MIKC_MADS family protein

ATGGGAAGAGGGAAAATAGAGATGAAGAAAATTGAAAATATAAGTAGTAGACAAGTAACATTTTCAAAACGAAGAGCTGGACTTTTTAAGAAAGCTGAAGAATTGTCTGTTCTTTGTGATGCTGAAATTGGTGTTATTGTTTTCTCTAATACTGATAGGCTTTATAAATTTGCTAGCTCCAAATCCAGTATGGAAAAAATTGTGGAGAGATATAACAGTTCTTCACATTCATTTGAGCATCCTATGATCGAAAATGTGGTAGAGCCTGAATTGAATTCCTTGAAAGCTGAAGTTGCAAAATTACGAAAGGCTACCGGAAGGATGATGGGGAAAGAACTTGATGGCCTGGACTTCAAAGAGTTGCAGCAATTAGAGCATCAACTAACTGAAGGCATTTTATCTGTTAAGAATAAGAAGGAACAAGTACTATTGGAGCTACTTGAAAAATCAAATCTGCAGATTGAGGAGCTTGGCCACAAATCATGTAATCATTATCCTGAAAATTATGAAGCAGCAAGGAAAATTTCTGGTGGAAATACAACAGTAATTTGTGATTTTAAATCAGTTGAAGAAGAAAATTCAGACACTTCTTTGAGCTTAGGACTATCAGTTGCTACAAGTCAGAAGAAAAAAAATCCACAGATTGAATGCACTTCAAATGATTCTGAGAATCTAATGATTTTAGATTAA

>Solyc11g005120.1.1 Solanum lycopersicum|MIKC_MADS|MIKC_MADS family protein

ATGGGGAGAGGAAAGATAGAAGTGAAGAGAATTGAGAACAAAACAAGTAGACAAGTTACTTTCTCAAAGAGAAGAGCTGGACTTTTGAAGAAAACACATGAACTTTCTGTTCTTTGTGATGCTCAAATTGGACTCATCATTTTCTCAACCAAAGGCAAATTGTTTGAGTACACCACTCAACCTCACAGCATGGGTGAAATCATTAATAAGTATCTCCAAACTACTGGTGCCTCACTTCCAATTCATGATCATAGGGTGGAACAATATGATGAAATAACAAAAATGAAAAGAGAAACATTGAATCTTGAATTAAGTCTTCAAAGATACAAAGGTGATGAATTGAACTCAGCACAATATGATGAATTAAATGAACTTGAGAAGCAGCTTGAAAATTCTATTAACAAAATTAGAGCCAGAAAGCTTGAACTCTTGCAACAGCAGATGGAAAACCTGAAGAGAACAGAGAAAATGTTGGAGAAAGAAAATCATGATATGTGTCAGTGGTTGATGAAGTATGAAATGTACAAGCAACAGCCAGTAGCAATGATGGAGCAACAAGAAGAAGCAGCAATTACTGAACTGAATTTACTTGGAGAACAACCATTGTTGTCTCAATTTTCATTCTTTGGAGATCAACACCAACTTGGTACTACAAGTAATTCTTCAGCATATCACCTTCAAACTTCTCACCCTTTTACTCCCTCTACCTACGATTGA

>Solyc11g010570.1.1 Solanum lycopersicum|MIKC_MADS|MIKC_MADS family protein

ATGGCTAGAGAAAAAATTCAGATCAAGAAAATAGATAACTCCACAGCAAGACAAGTTACATTTTCAAAGAGGAGAAGAGGTTTATTCAAGAAAGCTGAAGAACTTTCTGTTCTCTGTGATGCTGATGTTGCTCTCATCATTTTCTCTTCTACTGGAAAATTATTTGACTATTCTAGCTCAAGCATGAAACAAATTCTTGAGAGGCGTGATTTGCATTCCAAAAATCTGGAAAAATTGGATCAACCATCACTTGAACTTCAGCTTGTAGAAAATAGCAACTACTCCAGATTAAGCAAGGAAATTTCCGAAAAAAGTCATCGATTAAGGCAAATGAGGGGAGAAGAACTTCAAGGACTAAATATTGAAGAGTTGCAACAATTGGAGAGATCTCTTGAAACTGGATTGAGCCGCGTCATAGAGAGAAAGGGTGATAAAATAATGAGAGAGATCAACCAACTCCAACAAAAGGGTATGCATCTAATGGAAGAAAATGAAAAATTAAGGCAACAGGTGATGGAGATATCTAATAATAATAATAATAATAATAATGGATATAGAGAGGCAGGAGTAGTAATATTTGAACCAGAAAATGGATTTAATAATAATAATAATGAAGATGGCCAATCATCTGAATCAGTAACAAATCCATGTAACTCAATTGATCCTCCTCCTCAAGATGATGATAGTTCTGATACTTCTCTCAAATTGGGGTTAGCTACCTTACTCAGGCTGAAGAGATCAAAAGCAAGGTGTGGCTATTTTTGTATGTTATTAGAAGAAGGAGAAAAAAAAAAGTAA

>Solyc11g028020.1.1 Solanum lycopersicum|MIKC_MADS|MIKC_MADS family protein

ATGGGTCGAGGAAAGATAGAGATAAAGAGGATTGAAAACAACACAAATCGACAGGTAACATTCTGCAAGAGAAGAAATGGATTACTGAAAAAGGCATATGAACTTTCAGTTCTATGTGATGCTGAGATTGCTCTCATTGTTTTCTCTACTCGTGGTCGACTCTATGAATACTCTAACAACAATGTAAAGGCAACTATAGAACGATACAAAAAGGCAACAGCAGAAACGTCTAGTGCGTACACTACTCAAGAGCTCAATGCACAGTTTTACCAACAAGAATCAAAGAAGTTGCGGCAACAGATACAAATGATGCAGAACACAAATAGGCATCTGGTGGGTGAAGGGCTGAGCTCTTTGAATGTGAGGGAACTGAAGCAATTGGAGAACAGACTTGAACGAGGCATTACAAGAATTAGGTCCAAAAAGCATGAAGCAATACTAGCTGAGACTGAGGATTTGCACAAGAGGGAAATTCAACTTGAACAGGAAAATGCATTCCTTAGATCAAAGATAGCAGAAAATGAGAGGTTGCAGGAACTAAGCATGATGCCATCTGGTGGGGAAGAGTATAATGCATTTCAACAATATTTAGCAAGAAATATGCTACAGCTCAACATGATGGAAACTGCACTACCATCTTATGATCCATTGTCTCCTGATCACAAAAGGTAA

>Solyc11g032100.1.1 Solanum lycopersicum|MIKC_MADS|MIKC_MADS family protein

ATGGCTCGTGGTAAGGTTCAAATGAAGAGGATAGAGAATCCAGTTCATCGACAAGTCACTTTCTGCAAACGTCGAGCAGGCCTTCTTAAGAAGGCCAAAGAGCTCTCCGTTTTGTGCGATGCTGAAATTGGTCTTTTCATTTTCTCCGCTCACGGAAAGCTCTATGAACTTGCTACTAAAGGAAGCATGCAAGGGCTGATTGAGAGGTACATCAAGTCAACCAAGGGAGTTGAGGTGGCTGAGGAAGCCAAAGATACACAACCTCTGGACCCAAAAGAGGAGATCAACATGCTGAAGAATGAGATTGACGTACTCCAGAAAGGCTTAAGCTACATGTATGGGGGAGGCGCAGGAACAATGACACTAGATGAACTTCATTCACTTGAAAAGTACCTTGAAATTTGGATGTATCATATTCGTTCAGCAAAGATGGATATCATGTTTCAAGAGATCCAACTGTTGAAGAATAAGGAAGGGATACTGGAAGCTGCAAACAAATATTTACAGGATAAGATAGATGAGCAATACACTGTGACTAACATGACCCAGAATTTGACTGACTTTCAATGCCCACTAACTGTACAAAATGAGATATTTCAGTTTTAA

>Solyc12g038510.1.1 Solanum lycopersicum|MIKC_MADS|MIKC_MADS family protein

ATGGGAAGAGGAAGAGTAGAACTAAAGAGAATAGAGAACAAAATAAACAGGCAAGTTACTTTTGCTAAGAGAAGAAATGGACTTCTTAAGAAAGCTTATGAGTTATCTATACTTTGTGATGCTGAAGTTGCTCTCATCATCTTCTCTAGCCGCGGAAAACTCTATGAGTTTTCAAGTGCTTCCAGCATGATGACAACACTTGAAAAGTATCAACAATGCAGTTACGCATCTTTGGACCCGATGTTACCGGTTAGTGATACTCAGATGAACTACAATGAGTATGTGAGGCTAAAAGCTAGAGTTGAGCTCCTTCAACGTTCTCAAAGACATATTCTTGGAGAGGATTTGGGCACACTAAACTCGAAAGAACTTGAGCAGCTTGAGCACCAATTGGATGCATCTTTGAAGAAAGTTAGATCAAAAAAGACTCAATCTATGCTGGATCAGCTGGCAGACCTTCAAGAAAAGGAGCAAATGCTGGAAGAAGCAAATAAACAACTAAAAAACAAGCTGGAAGAAAGTGCAGCTAGAATTCCACTTGGATTGTCATGGGGAAATAATGGAGGACAAACAATGGAATACAATCGACTCCCTCCACAAACTACTGCACAACCTTTCTTTCAACCTCTCCGTTTGAATTCTTCATCGCCTCAATTCGGATACAATCCAAATATGGGTGCAAATGATCATGAGGTTAATGCAGCAACAACTGCTCATAATATTAATGGATTTATTCCAGGGTGGATGCTCTAA

>Solyc12g056460.1.1 Solanum lycopersicum|MIKC_MADS|MIKC_MADS family protein

ATGGTGAGGGGAATAACTGAGATGAAAAGGATTGAAAATACAACAAGTAGACAAGTGACATTCTCAAAAAGAAGAGGTGGACTACTTAAGAAGGCATTTGAACTCTCAGTTCTTTGTGATGCTGAAGTTAGTCTCATCATATTTTCTCAAAAAGGAAAACTTTTTGAGTTTTCAAGTTCAAGTACTAACAAGACAATAGAACGTTATCAAAAGAATGACAAGAATTTGGGTCATGAAAATATATTACTTGAACAAACTACAGAGCATTTGAAGGGGGAGGTTATGAGCATGACTAGGAATCTGGAGGTTCTTGAAATCTCTAAAAGAAGGCTTTTAGGAGAAGATTTAGAGTCTTGTTCCATTGATGAACTTGAAAAGGTTGAAGGACAATTAGACCAAAGTTTAAGAAATATTAGGGCAAAAAAGAACCAGCTATTCAAGGAACAAATTTCTCTTCTTAAAGACGAGGAAAAAGTCCTAATGAATAAAAATGCAGAATTGCGGGAAAAGTATGAGGCTCGATCACTACCTTTATTTATTGATCGACGAGAAGATGAAAGTCCTCAAACACAAAATATGGAGGTAGATACACAACTATTTATTGGACTTCCTGAAAGATGA

>Solyc12g087830.1.1 Solanum lycopersicum|MIKC_MADS|MIKC_MADS family protein

ATGGGGCGAAGGAAGGTAGAAATTAAGCGAATTCAAGATAAAAATTGCAGGCAAGTTGCGTTCTGTAAACGGAGGAAGGGTTTATTGAAGAAAGCTAAAGAAATTTCTATTCTCTGCGATGTCGATGTTGCTGTTGTTATCATCTCAAATCGTGGTAGACTCCATGAATTCTCCAGCAATAACAGTATGACAGCGATGCTTCGACGATATGAAAGCCATGTTGGAGCAGAAAAAGAGATCAATGCAGAAATCCAGGTCGCAGAGGTGTCGGGGTTCACAACAATGGGAGAACTGCTACAAACAACAGAAAGGCAACTCGAGGAAACAAATGCTGATGGTCTCACTTTGACTGACCTTATCCATTTGGAAAACGAACTTCAAACTGCTCTAATACATCTCAGAGCTAGAAAGACACATTTGATGCTTGAATCTGCTAAGGTTCTTCATGAGAAGGAAAAACTGCTGCTAGAGGAAAAGAAACATCTGGAGGACAATATAGCTAGTATCAAGAAAAACACAAAAGTGAATGAAATGTCTGACCTTCCAGCACCCCACATGATTTGTGGACAACAAAAAGTTACCCTGAATTTCTTCTAG

>Solyc00g179240.1.1 Solanum lycopersicum|M-type_MADS|M-type_MADS family protein

ATGAGGCGTATAGAGAACGCCACAAGCAGACAAGTTACTTTCTCAAAGAGAAGAAATGGTTTGCTAAAAAAAGCTTTTGAGCTTTCTGTCTTATGTGATGCTGAAGTGGGATTGATTATATTTTCTCCAAGAGGAAAACTATATGAATTTGCTAGTTCAAGGNNN

>Solyc01g010300.1.1 Solanum lycopersicum|M-type_MADS|M-type_MADS family protein

ATGGCACAAACTAAAATTCTACGCCGGCAAAAGATTAAACGGAAAATGATAACATCTAAAGTGAAACGTATGGTACTTTTCAAAAAGCTATGGGCAAATTTTGTCAAGGAAGCACATGATCTTTATATGACAACGGGAGCACATGTTACTATTGTCGCTTTTTCCCCGACTGGCAAGGCATATGCTTATGATTCCTCCAACAATTTTGATACTATAGAGAGGTTCCTTAATGATTCAAAAGCTTCTGCAATTGAAGGCGGTCATTGA

>Solyc01g060300.1.1 Solanum lycopersicum|M-type_MADS|M-type_MADS family protein

ATGAGAAAACCTAATGGCCGCAAAAAAATTGAAATTGCGAAGATTCAAAATCAAACCAACTTGCAAGTAACATTATCAAAAAGACGTGCCGGCCTATTTAAAAAGGCAAGTGAGCTCTCGACTTTGTGTGGTGCTAATGTTGCTATTGTAGCTTTTTCTCCTAGCAACAAAGTATACGCATGTGGACACCCTTCCGTCGAGTCAATTGTGGATAAATTTATCGGAGAGAATCCTCCACCTGAAACTGATGATCCTAACCCCATCATTGTAATGACTGCTGACACAATTCCTAAGGGATCTCCATTTACGAGCATGCAAGAGTTTTCATTAGGAATAGACTTTGGAAAACTGTGTCAGATTACTTGTGTTCCACAGTTGATTGAGAGTTGCCCCAATTCGAGTATACTTCAGATTTGGACCGATTACCATCTTGGATCTACAAATGAACTAGCCCATCAGGCGTTATTGGACTTAGGAGGGTACCGACTCGTTATCCTTCCACTGCATAGAGCGGGAACTGTTCGTTGCTCTGTGAAACCAGCCTCACGCAGTCCGTTCCACTTTAGGAACCCATCAGCCCATTCCTTCGATTGGATAGCAAGGCCCAACTCTATGCTTGGACAGGGCAGGCCATAA

>Solyc01g060310.1.1 Solanum lycopersicum|M-type_MADS|M-type_MADS family protein

ATGAGAAAACCTAATGGCCGCAAAAAAATTGAAATTGCAAAGATTCAAAATCAAACCAACTTACAAGTAACATTCTCAAAAAGACGTGCCGGCCTATTTAAAAAGGCAAGTGAGCTCTCGACTTTGTGTGGTGCTAATGTAGCTATTGTATCTTTTTCTCCTAGCAACAAAGTATACGCATGTGGACACCCTTCCGTCGAGTCAATTGTGGATAAATTTATCGGAGAGAATACTCCACCTGAAACTGATGATCTTAACCCCATCATTGTAATGGGCTTAAGTTTCCTTGAGACCGATTACCATCTTGGACCTACAACTGAACTAGCTCAAGTATGTGTGCCCGCAGCACGACAGATGTCTGGTGCCCGCAGCATAATAGTCGTCTCACAATGTGTGCTTGAGAGGCTCATTGCAACGTGA

>Solyc01g066500.1.1 Solanum lycopersicum|M-type_MADS|M-type_MADS family protein

ATGACGAAAGGAACAGGGAAGAAGAAGATTGACATTAAAAAGCTCGCCAGTAATTCATCAAGAAAAGTCACATTCTCGAAAAGACGCACTGGTCTTTTCAAGAAAGCTGAAGAACTCGCAACCAAATTCGGCGCTCGAATTGCTCTGTTAGTCTTCTCGCCGGCCGGACGACTCTACACTTCCGGCGATGTTTCCATCTTCGAGAATAATGGGTTTCCTTCAAAACCCCATCTTTCTACTGAAACTGCGTCGAATTCATACGTAAAACCAAGTACTCTGCGTCTGAAAAATCTCAATGTTGAAGGATGTAGTGAAATTTTCTCGCCAGACGGCGTACATTATAGTGCCGGCGAGATTCCCATCGTCGAAGACAACTTGCAGAATAATGGGTTTTCTTCAAATTCTCATCTTTCTACTGATTTCAGTTCGAATTTTTATGAAGATTGGAGTACTCTCTGGCTGAAAAATTTCAAGGGAAATAATGAAATTTTCTCGCCGCCCGGCGGCTTTTACAACTCTGGTGAATTTCCCATTGTTGCGGACGAGTTGAAGAATAATGAGTTTCCTTCAAACTCCGATCTTTCTATTGAAATTTCATCGAATTCATATGCAAAACCAAGTACTCTGCATCTGAAAAATTTCAATGCTGAAGGATGTAGTGAAATTTTCTCGACAGACGGTAGATTTTACAGTTCCAACGAGTTTCCCATTATCAACGACACCTTGAAGAATAATGGGTTTTCTTCAAATTCCGATCTTTCTACTGATTTGGGGTCAAATTTGTATGAATATTGGAGTATGGAAAATTTTAATGTTGAAGAATGTAGCAGTATTGAAGAACTTATGTTATTGAAGGAGAAATTAGAAGAAAAGAGAGATGAAATTATTTTGAAGATGGAGGCTGAGTTTATTGATTCATTACTTGTTTAA

>Solyc01g066730.2.1 Solanum lycopersicum|M-type_MADS|M-type_MADS family protein

ATGGGTAAAGGGAAGCAGAAAATTGAAATTAAGAAAATCACTAAAGAATCAGCAAGAAAGGTGGCATTTTCGAAGAGACGCAAAGGACTTTTCAAGAAAGCTGTGCAACTTGAATCCAAGACTGGTGCTAAAGTTGCCATTCTTGTTTTCTCTTCCTCAGGAAAACCCTACACTTGTGGGGACGTGGAAACGCTTTGTGGTATTTCCGATTCATTCAATCTTCAAACGCATTCTGAGTCAAATAGTATTTGGGATTCATTCAATCTTCAAACGCATTCTGAGTCGAATGGTATTTGGGACTCATTCAATCTTGAAAGGCCTTGTGAGTCGAATGGTATGTGGGATTCATTCAATATAATTGAAGGGCCTTGTTCATCCTCTGGGAAGAATGGTATGTGGGATTCATTCAATCTTGAAAGGCCTTGTTCATCCTCTGGGCAGAGTGGTATGTGGGACTCATTCAATCTTGGAAGGCCTTGTGAGTCGAATGGTATGTGGGATTCATTCAATATAATTGAAGGGCCTTGTTCATCCTCTGGGCAGAATGGTATGTGGGATTCATTCAATCTTGAAAGGCCTTGTTCATCCTCTGGGCAGAGTGGTATGTGGGACTCATTCAATCTTGAAAGGCATTGTTCATCCTCAGGAATCCCATCTGGGTCGAATGGTATTTGGGATTCGTTCAATGTTGAAACGCATTGTTCATCCTCTGAGTCGTGTGGTATGTCTGATTCATTCAATGTTGAAACACATTGTTCATCCTCTGGGCAGAGTGGTACGTGGGATTCATTCAATGTTGAAGCATGTCATAACGTGAATGAACTCTTGCTGTTGAAGGCACACTTGGAAAGTACAAGAGAAAAGCTGCTGGAGTCTCAGTTTCTTGACTCTCTCTGGTCATGA

>Solyc01g097850.1.1 Solanum lycopersicum|M-type_MADS|M-type_MADS family protein

ATGGAGAATAAGAAGAGTAGAGGACGTCAAAAAATACCAATGAAAAAAATAGAAAAACTAGGTGACTTATATGCTAGCTTCTCAAAGCGCCGTTTGAGTTTGTATAAAAAAGCTAGCGATCTCGTTTTTGAATGTGATGTGGACATTGGAATGATATATTTTTCTCCTAAAGGTAATCCTTACTCATTTTTTCATCCAAATGTTAATACCGTTGTTTCTCGTTTTCAGAATCCTGATATGGAATTCAGTGAAAGTGATCTTCTGATCACGACTGATAACCAAGTAAAAGTGAAGGAACTCAAAAGCAGGCTTGATGAACTTGATATCATAGAAGACATTGCAATTGCTACGAAGAAATCATATGATGAAGTGATTAAAGCTAGAAAGAGAGGTTGGTGGGAGTCGATTGAGCAGCTTAATGAACCTCAAGTAACCAAGTTTGAAGCTTGGATGGATACTATTATTTTTAACATGCAGAATCGTTTGAATGAAATGGAAAATGGAGCTTCGTCCTCATAA

>Solyc01g098050.1.1 Solanum lycopersicum|M-type_MADS|M-type_MADS family protein

ATGGGCACAGGGAAGAAGAAAATAGAGATCGAGAAAATTATAAAGGAAACTTCTAGAATGGTTACATTCTCGAAAAGACGAAAAGGGCTTTTCAAAAAAGCTAAACAATTTGAATCCATGACGGGTTCTCGTGTTGCTTCAATTGTATTGTCACCCACAGGAAGGCCATATACTTGTGGAGACGTTGATTATGCTATAAGAACACATTTTTCTAATAGTGGTCGATGTATGAAATTATTAATAACAGACATCATGAATTCTCACGATTCTAATTCTAGTTCCAATGTTGTTGTTTATGGCGAAACTTCGAGATCGAAATTCTCTTCGGCTCCAAAGAAGAATAGTCTCCACAATTGGGTGAAGCGTATAGATGTTGAACAATGTCAAAATTTGAATTGGCTCTTAATGTTGAAACAACAATTGGAAGGGACAAAAGAAAAGATTGGTGAAGACGTTGAGTCATTTAAAGCTTTTTTTGTGTAA

>Solyc01g098060.1.1 Solanum lycopersicum|M-type_MADS|M-type_MADS family protein

ATGGGAACAAGGAAGAGGAGGACAGAAATCGAGAAACTCACAAAGCAAAGTGATAGATTGACGACATTTTCAAAAAGAAAGAAAGGAATTTTTAAAAAAGCTGAACTACTTGAATCCTTGACAAGTTCTCGTGTTACCTCCGTTGTTTTTTCACCTTCTGGCATTCCTTATACTTACGGAAATGTTAACTCTGTTATCAAAAAGCATTTTCCTAGCTGTAATCGATCAGAAATATCAACAACGGTAATGAATTCTCATCATGATGTTTCTGGCGAATCTTCGGGGTCAAAATCATTATCGATCCCAAAGGAGAATGGTCTCCGTCGTTGGGTGGAGGATATAGATGTGGAAGGGTGTCAAAATTTAAATCAACTGTTTATGTTGAAGGAGCAATTAGAAGGAACCAGAGAGAAAATTATTTCCAGTGATCCTGAGTCATTTGAAGCTTTGTTTATGTAG

>Solyc01g098070.1.1 Solanum lycopersicum|M-type_MADS|M-type_MADS family protein

ATGGGAACAGGAAAGAAGAAAATAGAGATTGAGAAAATCACAAAGCAAACTGCTAGAATGGTGGCATTTTCGAAAAGAAGGAAAGGACTTTTCAGAAAAGCTGAAGAACTTGAGTCCATGTCAAGTTCTCGTGTTACCTCTGTTGTTATTTCACCTTTTGGCAAGCCTTATACTTATGGAAATGTTAACTCTGTTATAAAGAAGTATTTTTCCATCTGTATCCGCCCAGAAATATCAACACCAGTGATGAATTCTCATCCTTCCTCTTCTAATGTTTCTGGTGAATCTTTGGGGTCAAAATCTTCATCGACCCCAAATGGAAATGCTCTCTGCAATTGGGTGGAAGGTATAGATGTCGAAGAATGTCAAAATCTGAACCAACTGTTAATGTTGAAGAAGCAATTGGAAGGAACTAGAGAGAAGATTGTTTCCAAAGAATCTGAATCATTTCAAGCTTTGTTTATTTAG

>Solyc01g102260.2.1 Solanum lycopersicum|M-type_MADS|M-type_MADS family protein

ATGACAAGAAAGAAAGTGAAATTAGCCTTCATCACTAATGACTCGGCAAGAAAAGCAACATTCAAGAAAAGGAAGAAGGGTCTGATGAAGAAGGTGAGTGAATTGAGCACCCTTTGTGGGATTGATGCTTGTGCTATTATTTATAGCCCTTATGACACTTCACCAGAAGTATGGCCAAACACCATGGGAGCTCAACGCGTGCTCGCCGAGTTTAAAAGGATGCCTGAGATGGAACAGAGCAAGAAAATGGTGAATCAAGAGAGTTTCATAAGGCAGAGGATAGCTAAAGCGAGCGAGCAATTGAAGAAACAGAGCAAGGAAAACAGAGAAAAGGAAATGACCGAAGTTATGTATCAAGGGTTGACTGGAAAAGGGCTTCAGAATTTGAATTTGGGAGATTTGAATGACCTTGGATGGGTAATTGATCAGAATTTGAAAGAGGTTTATAAGAGGATTGAGGCAGTTAAAAAGGGGGCTTCGACTTCCTCTTCTTCTTCTGTTGCTGCTGCTGCAGTTGCTGCGGCAGCAGTAGCATCGCAGGCAGTTGCTCCACCGATGGAGCAAAAGCCTGCTGTGGTGGAATTGGGATTGGATTCGATGCAGAGGACACAAACAGAGTGGTTTACTGATTGGATGAACAGCAACGCAAGTGATCAGCATATTGGTTATGGACATGCAGATGAGATGATTTTGCCCAATTTTAATGATAATCATAATGCTAATGTGTGGCCTAATAATTTCTATCCTTAA

>Solyc01g103550.1.1 Solanum lycopersicum|M-type_MADS|M-type_MADS family protein

ATGGGTCGTGCGAAATTGAAGATGGAATTAATAAGCAAGGAGAAATCAAGGAATGCAACGTTTAAGAAAAGGAAAGAAGGTTTATTGAAAAAGTTGTATGAATTCACTACACTTTGCAATGTGAATGGCCTTATGATAATGTATGGACCAAAACAAGGGAATGGATCAGAGTGTAGGCCTGAGATTTGGACAAATAGTAGTGGAAGTAGTAGTAGTACAAATAGTAAAAGCTTACAACAACAACAAGAAGAGATTGAGAATTTAATCGATGAGTATAAAAAGGAGAATAGTTTGCAATCTGGTAGTAGTAAAACCTTTGGATTGTCTGATTATTTCGTTGATCGAAACAAGAGGGTTGAGGAAGAGTTTATCAAGTTGAGAAAGATGAATATGGAGAAAAAATATCCATGTTGGCTAGAGTTTATGGATCAGTTATCTGAGTTTAAGTTGAGGGATTTTTTAACTTTGTTGGATGACAGAGTTGAAAATGTCAAGGCTAGGATTCATTTGCTTAAAGGGAATTTTAGTGGTTTGATGGGAGGAGAAATGATTGATTTGGGAGGAGGAAACCAATGGACTCATTACAATGACAATGTTATGGTCCAAGGAGGGGGGATGGAATATGGAGATTATAATCAATTGCAAGCTCCAATATATCATCAAGAAATGAGGATGGTGATGATGAATGAGAATGATTGGCCACAGTATAATAATGGTGCATCGTCGTCGTCCTCCGCGGGGAATGGGAGCAACAATATGATGTGTGCATTGATGAAATATGAAACTATGATGCCAAGTAATAATCACTTGGCATACTCACCCTATGTAGCTCCAACAATTCTGCAGCAAACTCCATGCATGATGATGCCACAGCATTCTTGGAGAGACAACGACAGAGATGACAAAGCTAAATTTTCACCTTATATGACCAAGTAA

>Solyc01g103870.1.1 Solanum lycopersicum|M-type_MADS|M-type_MADS family protein

ATGGCAGACGAAGACCAAAGCAAGAAGGGAGAAGAAGATCAAAGGAAGAAGAAAACAAACAATTCCAAATCTTATCAAGTGAGGAAAGAATGCATAAAGAGGAAATCAATGGAGCTTGCAACTCTCTGCGATATCAAGGTTTGTACGGTTATTACTGGTCCTAATAGGGAATTGCAAACTTGGCCTGACAATTTGAATGCCTGTAAGGAAGTTCTTGATATCTACTCTCAAAATTTGAAACCCGAAAAGAAACACAAGCAAGAAGACAAAGATCTCCCGACGCTAGTTGAATCAAAGCTTGCAGCAGTCAACAGGAGAATTTGTTTCTTGGAGAACAAGAATGTTGCTGATAAGGGAAAAGGAAAGAGAATTGAATGA

>Solyc01g106170.2.1 Solanum lycopersicum|M-type_MADS|M-type_MADS family protein

ATGATATTTCTAGACAGGTGTCAGCGAGTGAGGCTATTATGGTGTACTCGCGCGAGTAGGGGAAGTAAAATATCTGATTATTTTTTCACTAAACCAATATTTCTCCTCTCTCTCTCTCTCTCTATCTGTAGAATCAGTCTACTATCAAAATCTCTCAAATTATCTCGTTTAATTAAGGAGATGGGGAGAGGAAAAATAGTGATACAAAGGATTGATAATACAACAAGTAGACAAGTTACTTTCTCAAAGAGAAGAAATGGACTTCTCAAAAAGGCTAAAGAGCTTGCAATTCTTTGTGATGCTCAAGTTGGACTCATTATTTTCTCAAGCACTGGAAAACTCTATGAATTTGCCAATAACAGCATGAAGTCTACAATTGATAGATACAACAAGATAAAGGAGGAGAATAATAACATGAATCCCATGTCAGAGGTCAAGGTAACCTGA

>Solyc01g106700.2.1 Solanum lycopersicum|M-type_MADS|M-type_MADS family protein

ATGACCACCATTAACGCCGTCAAGAAAACTCAAGGACGGAGGAAAATTGCAATCAAACCGATAGACAATCAAAACAGTCGGCACGTTACTTTCTCCAAACGCCGTTTAGGACTTTTCAAAAAAGCTAGTGAACTTTGCATCTTAAGCGGTGCAGAAATTGCTATACTTGTACAGTCATTAAAACGACAGCGTCTCTTCACATTCGGTCATCCTAGCCCCGACGCCGTCATCGACCGTTACCTCACTGGAAAATCAGTATCTCCCGGTGACGGCGATCAATTCAACTTGCAACAGAGTAATCAGTATTATTCCCAGATTTGTAGAGATCTGGAATTAGAGAAGCAGAAGAAGGAGAATATTGAAGAATCGAAGATGGTTAATAATGGGGGATTTTGGTGGAACGAACCAATTGATGATATGGGGATTGAAGGACTTGAAGAATTTATGAGTGCTTTGGAAGAATTGAAGAAGAAAGTAACAATGAGAGCTGATGAATTGAGTATGATAAATGGGTCTTCTTCGAATTCGACCATGAAAATAGCAAGATTTGGGGCTGAAGATCAATATTTTAATGAATCAATCGATTATTGTTCTTCCATTGTTCCTTTTGATTTCAATCAACCAGGAGATAGACAATTCTGA

>Solyc01g106710.1.1 Solanum lycopersicum|M-type_MADS|M-type_MADS family protein

ATGGCAAAAAAACCTAGTATGGGTCGTCAAAAGATCAAAATTGCCAAGATAGAGGTCAAGAATCACCTCCAAGTTACCTTCTCAAAACGTCGTTCTGGTCTATTCAAGAAAGCTAGTGAACTATGCACGTTATGTGGTGTTGAAATAGCCATCATAGTTTTTTCTCCAGCGAGAAAAGTATTTTCTTTTGGTCACCCTAATGTTGAGTCTATTATCGATAGGTTCCTCACAAGAGCTAATTCTAATAATAATCCAATCGCGAATAATTCAATTCAACTTGTTGAGGCTCATCGAAATGCTAGTGTTCGTGGGCTCAATTTACAACTTACTCAAATTCTTGGTGAGGTTGAAATTGAGAAGAAAAGAGGAGAATCACTTGATCAAATGAGGAAAACTAGTCAAAGCCAATATTGGTGGGAAGCTCCTATTAATCAACTTGATTTGCAAGAACTTGAACAATTAAAGGATTCAATGGAGGTTTTGAAAAAAAATGTTACTAATCAAGCAAACAAGTTTATGGTTAATGAGACTCCTAATCCTTCTTTTTTTGGTGTTAATGCTAATGGGATTTTTGACAATTATGACATCAAGCCACCAAGAAACATGAATGCTTCAAATAATCTTCATAACCATAACCTTGGTTTTGATTCATCCACATTTTTCTAA

>Solyc01g106720.1.1 Solanum lycopersicum|M-type_MADS|M-type_MADS family protein

ATGGCAAAAAAAATGAGTAAAGGTAGGCAAAAGATCCAAATGACCAAAATGTCAAAAGAAAGTAACCTTCTTGTTACCTTCTCGAAACGTCGTTCTGGTCTTTTCAAAAAAGCTAGTGAACTTTGTACCCTTTGTGGTGTTGAAATAGCGATTGTTGTCTTTTCACCAGGTCATAAAGTCTTCTCTTTTGGTCATCCTAATGTTGATTCTATCGTAAATCGCTTCCTTACTCGTAACCCTACGAGTTCTTCTTCTACAACTTGTCAACTTGTTGAAGCTCATAGAAACGCAAATGTACGCGAATTGAACGCACAACTCACGGAGATTCTCAATCAATTAGAGTTTGAGAAAAAACGCGAGGTTGAAATCGAAAAAATTCAAAAGGGTAAAATTGGAAAGAATTGGTGGGAAGGTCCATTGAATGAACTTGAACATGGTGAATTGGAACAATTGAAATTGGGAATGGAAGAACTCAAGAAAAATGTTACAAAACAAATGCAAAAAATAATTTTTGAGGCTTCAAATGCACCTACATTTTTTCTTGGAGGATCATCTTCTTCTAATGGAGATGTCAAAAATATGAAGGGGCTTGGACTTTCTATGGCAACCAATGGTGGGCATACAAGTTTTCCATGA

>Solyc01g106730.1.1 Solanum lycopersicum|M-type_MADS|M-type_MADS family protein

ATGGCAAGAAGGGTTAGCAAGGGTCGTCAAAAGGTTGAGATGGTGAAAATGAAGAATGCAAGTAACTTACAAGTTACGTTTTCTAAGCGTCGAGCTGGTTTATTTAAAAAAGCTAGCGAACTTTGTACGTTATGTGGTGCTGAAATTGCCATAGTGGTATTTTCCCCTGGTGATAAAGTTTTCTCCTTTGGTCATCCCAATGTAGAAACGTTGGTGGATAGGTTCCTTGGGAGGGACCTCCCTTTACCAAATAACGATGTCCACAATCATCTAATCGTGGCTCATCGAGAAGCTGGTATTCGTGAGCTCAATACGAAGCTCATGAATCTCGAGGGGGTTCTCCATATGGAGAAAAAACGCGGAGAATCCCTACAAGAAATTAGGAGGAGAGCTAATGGTCAATGGTGGGAATCTCCTATAGAAGAACTTAACTTATTCCAACTTCAACACTTGAAGGAGGCCTTGGAAAATCTAGAGCAAAAGGTTGAAAAAGTGGCACATCAACAACAAATGCTAAATAATATTGCATTCCCATTCCGCACACTTGGAAGTGCTTTGACTCCTCCTAATTGTGCTAGGGAAACTTCTTCATATGGACTCAATGTTGGTGCACCCTTTGCTAATAGGGATATTGGATCTACTTCTTCCATAGTTCCCAATCACTAG

>Solyc02g032000.1.1 Solanum lycopersicum|M-type_MADS|M-type_MADS family protein

ATGGAAAATGAGACTAAAAAAGGTAAGAAAAAAATTGAAATGAAGCTTATCGAGTCTGAGAGAGCACGTGCTGTGAGTTTCTCAAAAAGAAAAAAAACTTTGTTTGAGGATGCAAAGAAGTTTGCAACTCAAACCGGTGCAGATGTTGCTGTGATGCTCTTTTCACCAGGTGGAAAACCATATTCCTGTGGTTCCACAAGCGTAGAAGATATAATTGAAAATTTTCTCAAAATGAAAGTGGCGGACCCCCGGCGTCATTATGCTGAGGGTGAATCAAAGGTTTTGAGGAATTGA

>Solyc03g007020.1.1 Solanum lycopersicum|M-type_MADS|M-type_MADS family protein

ATGGGATCCAAAATTCGTGGTCTAACCTTATCAAATCACGAGACATTTTCTCAAGTGTTCTTACCAATAAACTTATTTTTTAGAAAAAATATCTTACATCAAAAGTTGAGTCATGGAAAAAAAAAGACTTTAGGACGTCAAAAGATTTCAATGGTCAAAATAGAAAATGAAGATGCTCGATACACAACATTTTCAAAGCGTCGATCAACATTATATAAAAAAGCTAGTGAATTGGTTGGAAAATACGATGTTGATGTGGGGATAACATTATTTTCTCCGACTGATAAACCCTACTCTTTTTTTCACCCTACAGTTGATGTTGTTGTTGATCGTATTCTCAATCCAAATACACAGCCAAGTGAAGATAACAGCATCGCGATTGCAAGTTATCGAAACAAAGTGAAGGATCAAAAAGTTGAGCTTGACGAGCTTGATATCATAGAAAGAGGTATATCTAATTCGACATTTGGCACTAAGGAAACTAACATGAAAAATATATGGGAATCCTTTATGAAATTTAATGAAGATGAGGTGAATGAACTCGAGCTTTGGCTGAACTCTATTGATTTTGATTTGAAAAATTATTTATCGCAGCTGGAAAATAATGTTTCTTCCTCTACACAAGCACCTCCAAAAAATGTTGGTTAG

>Solyc03g034260.1.1 Solanum lycopersicum|M-type_MADS|M-type_MADS family protein

ATGGATAGCAAGAAGACAAGAGTGAGACAAACATTTCCAATTTCAAAAATAGAAAATCAACGTGTTAGCGATGTAACATTTTTCAACCGTCGCTCTAGTCTATACAGAATGGCAAACGAACTTGTTGATTTCTGTGATGTTGATATTGGAATAGTACTATTTTCACCATCAAACCACCCTTTCTCCTTTTTTCACCCAACAAGTGAAGCAGTCATTGAACGTTTTTTGAATCCCGATTCACAACTAAGTGAGAAAACTCGCCTAGATGCGGAGCAAGCACGAAATAAGGTGAATCAACTCAACAATCGCCTAGATGCTATGGAGAAAAGGATTGAGCAAATAGAAGAAATTGAACATGCTCAAACTCTACTTCAACTTAGCCAAACGGAAGAAAATGGCGAAAGAAGTAAGTGGAAATCGATTGATCAATTAAATGCAAATGAAATACCAACATTTGAAGCATGGTTAAGAACCACCGTCTCTAAAATGAATTATCGTTTGGAGAAGTTGGAAAATGAGGCTTCATCATTGAAAAATGCACGTGGAACTTCATAA

>Solyc03g062820.1.1 Solanum lycopersicum|M-type_MADS|M-type_MADS family protein

ATGGGCACAGGAAAGAAGAAAATAGAGATGGAGAAAATTACAAAGAAAAGTTGTAGAATGGTGACATTCTCCAAAAGAAGGAACGGACTTTTCAAGAAAAATGAAGAACTTGAATCCTTGACGGGTTCTCAAGTTTCCTCTGTTGTTCTTTCACCCGCTGGAAGGATTTATACTTACGGAGACGTTAACACTGCTATCAACATGCATTTTTCCAAAATTGATTGCATGAGACAATCAGATTCTGATGTTGTTGTTTCTAGTGGATCCTCAGAGTTGAGATCATCATCGAAAAGTCTCCGAGATTGGCTGGAGGATATAGATGTTGAACAGTGTCAAAATTTGAATCAACTTTTATTGTTGAAGGAGCAATTGGAAGGAACTAAAAAAAAGATTGTTTCCATTGAAGATTCTAAGTCCTTTCAAGCTTTGTTTATGTAG

>Solyc03g115910.1.1 Solanum lycopersicum|M-type_MADS|M-type_MADS family protein

ATGGCATCTTTTGCATGTGTTTTTTGTCCTCTAGAATTTCACATCAGCGAACCCCTTCCAGCGTTCAAACTTGGGTCTTGCCCGACAAGTAATCCCAAGTTCCAAAGTGCGCCGATATGTTTTTTCCATAGGATGGGAAGGGTAAGGCTAAGCGTGAAGCGATTGGAGAGTCATAGTAACAGGCAATCCACTTATTGCAAACGAAGATGTGGAATCCTGAAGAAAGCCCAGGAGATATCTGTGCTATGTGATATAGACATTATCCTTCTTTTGTTTTCCCCAACTGGAAAACCAACGCTATTTCAAGGCGGACAAAGCAATTTTGATGAGATAATTGCAAAATTTGCTCAATTGACTCCTCAAGAAAGGGCAAAAAGGAAGTTGGAGAGCCTTGAAGTAAGTCTGATATTCATCACCACTATGTGTTCTCCTCTTGCTATGCTTTGTTGTTACTTCATAATCCAAGTGAAAGTAATAACAATGCTAATGGTTACACTCCAGACATTGAGGAAGACTTTTAAGAAGCTTGACAACGATATAGGCGTGCCAGAGTTTCTAGATGCTAGTGATCCATCAGTTGAAGAATTGCATAGTCAAGTGAAACTTTTGCAATCTCGGCTTACTGATGTAGAAATGAGACTCAACTGGTGGAGCAATCCTGATAATATAAATAAAGTGGAAGATTTCGCGCTAATGGAGTGTGCACTAAGGGAGTCACTTAATGCAGTTCATGTTCGGAAGCTCCAGAAGACGACGCATTTGCATCTTTTGATGAACAGTGAACTAGATGGGAATACTCATCAGTGGCATCCAGAAAATAAAATCCTGCGCATGCCATTCCCTCAGACTCCAAACATTCTACCCCAGGAAAATATGGGATACTTTGGAGATAATTCAGTTGCTGAATCTTCTCATGTTCAGGGCTCTGGAGAAGTTGATCAAGCAAGACAGGACACAACTGCAATGCTAGATAACGGTGTCTTGAATGATCTAACTAGTATAGCATGCTTGAGACAGCAACTAAGTGAGCAGTACTCATACAACCCCAATGAGGATCTTGATCTGCTTGAAAGGAACATGTTGGATCCTCAGAGTGATGCCAATTTGAAAGGATATTTGATGGATTATGCATTTCAAAGAAACTTTAACCTAACAAGGTCTGTGGATTCAGTCAATTATAGTGCAGTCGATGCCGTTGCTGTTCCAGATTTTGATGAAAAATCATATGCGCAGCCAGCAACTTCCAGTGATTAG

>Solyc03g119680.1.1 Solanum lycopersicum|M-type_MADS|M-type_MADS family protein

ATGGAAAATGAGACCAAAAAAGGTAAGCAAAAGATTGAAATGAAGTTGATTGAATCCGAGAAAGCACGCACAGTGAGTTTCTCAAAAAGAAAAAGAACTTTGTTCCAAGATGTAGATAAGTTTGCTGCTCAGACCGGAGCAGATGTAGGTGTTATGCTCTTTTCACCAAGTGGAAAACCATATTCCCATGGTTCCACAAGTATAGAAGAAATCATCGATCAATATCTCAAAGTGAAACTAGAGGATCACCAACGTGATCATGCTGAGGGAAAAATGAACGGCTTTGAGGTATTGGGAGCTCTCCATAAAGAATTACAAGCATGGAACGAGAAAGAAAAAAATCGAAAACTAATGTATAAGATTATGCACTCTGGCTCAGAAGCACCTCCAGATAAACACATGGAGGAGCAGAAGTTGGCATTGAAGTTGAGGGTAGAGAAAATTAAAAAAGAAACACAAGCTGCTATTCTGGTTGAGCATCTAAAGTTTGATTTAAATGTTGCCCCTGAGCCAGAAGAAGATGAGAGTTCTTGA

>Solyc04g025030.1.1 Solanum lycopersicum|M-type_MADS|M-type_MADS family protein

ATGACAACAAGGATTAACAAGGGTCGTCAAAGGGTTGACATGGTGAAAATGAAGAATGCGAGGAATTTACAAGTTACATTTTCTACGCGTCTTGCTGGTCTGTTCAAAAAGTCTAATGAACTCTGTATGTTGTTTAATGCTGAAATTTTCATTGTGGTATTTTCACAGGGTGATAAAGGAGTTTTGTGCTTTGATCACCCTAGCGTGAACCCATTGGCAGAAGGGTTCTTTGAGTGGAACCTCCCTCAACCACATATTAATGTCCACAATCAACATATTGTGGCTCGTAAAGAAGGCGGTACTCGTGATTTGAGTACCAAGCTCATGAGCCTCGAGGCGATTCTCGAGAAAGAAAAAAATTGCGGACAAATCCTTATAGAAATTAGGAAGAGAGCTAATAGTCTATGA

>Solyc04g025050.1.1 Solanum lycopersicum|M-type_MADS|M-type_MADS family protein

ATGAAGAATACTAGAAACTTACGAGTTACATTTTCTAAGCATCGTGTTGGTTTATTCAAAAAGGCTAGTAAACTTTGTATGCTATGTGGTGCTGAAATTTCCATTGTGGTATTTTCCCCGAACGGTAAAGTTTTCTCCTTTGGTCACCTTAGCATGGACACGTTGATATAG

>Solyc04g025110.1.1 Solanum lycopersicum|M-type_MADS|M-type_MADS family protein

ATGGCAACAAGGATCAACAAGGGTTCTCAAAGGGTTGACATGGTGAAAATGAAGAATGCGAGGAACTTACAAGTTACGTTCTCTAAGCGTCTTGCTGGTCTGTTAAAAAAGGCTCTTGAACTTTGTATGTTGTGTGGTGCTAAAATTATCATTGTGGCATTTTCACCGAGCGATAATGGAGATTTCTCCTTTGGTCCCACTAGCATAAGCCCATCGGTGGAGAGGTTCCTTGGGAGGAAATTTCCACAACCAAATAATGATGTTCACAATCAACAAATCGTGGCTCTTATAGAAGGTGGTATTTGTGAGCTCAATACCAAGCTCAGGAACCTCGAGGGGATTCTTGAGATGGAAATAAATCGCGGACCATCCCTTGGAGAATTAGGAAGATAG

>Solyc04g025970.1.1 Solanum lycopersicum|M-type_MADS|M-type_MADS family protein

ATGGAGGGTAAGAAAAAAGCAGGCTGCCAAAGAATTCCACTAGAGAAAATAGAAAAAAAAGTTGCTCGATATGCCTCATTTTTTAAACGTCGTTTATGTTTGTATAAAAAAGCAAGTGAACTCATTCAAGAACGTGATGTCGATATTGGAGTATTTATTTCTTCTCAAACTGGTAAGCCGTATTCATTTGTTCATCAGACTGCTAATGTAGTCATCAATCATTTTAAAAGTCCCACAACAATAGATCTAGGTGCACAATTCGCTGGTGCAGAGGCACGCAACAATGTGATTCAAATGAACGATATGCTAAATGACTTTGATGCAAGGGAAAAAGTTACAAAGAACCATATATGA

>Solyc04g047870.1.1 Solanum lycopersicum|M-type_MADS|M-type_MADS family protein

ATGTCAAGAAGAATTTTCAAGGGTCGTCAGAGGGTTGACATGGTGAAAATAACGAATGGGAGAAACTTAGGAGTAACATTTTCTAAGCGACGTGCTGGTCTGTACAAAAAGGCTTGTGAACTTTGTATGCTATGTGGTGCTGAAATTGCCATTGTTATATTTTCTCCGGAGGGTAAAATTTTCTCCTTTGGTCACCCTAGCGTGGAAACGCTGGTGGAGAGGTTCCTTGGGAGGAACCTTCCTCCACCAAATAACGATGTCCACAATCAACAAATTGTGGCTCATAGAGAAGCTGGAATTCGTGAGCTCAATACAAGGCTCATGAACGTCGAGGGGGCTCTCCAGATGGAGAAAAATCGTGGAGAATCCCTTCAAGAAATTAGGAAGAAAGCTGATGGTGTATGGTGGCAATCTCCTATCAAAGAACTTAACTTGTTCCATCTTCAACACTTGAAGAGGGCATTGGAAATTCTAAAGCAAAAGGTTGTAAAAGAGGCACAGATGGTGAATAATAATGCATTCCCATTTCAGACATTAGGAAGTGCTTGGAGTCCTCCTAATTATACAAGCTAA

>Solyc04g056550.1.1 Solanum lycopersicum|M-type_MADS|M-type_MADS family protein

ATGGAGAGAAAGAAGACTAAAGGGCGTCAAAAGATACCAATGCAAAAAATAGAAAATAAGAATGCCCTGCTAACCACATTTTCAAAGCGTCGGAAGGGTTTGTTCAAAAAAGCTAGTGAAGTTGTTACAGAATGTGATGTTGACATTGGAATAATGATGATTTCACCTTCTGGTAAGCCACACTCATTTTTTCACCCTACTGCTGATGCAATTGTTTCTCGTTTTCAGAATCCTGATATGCAGCTAAGCGAAGGTATTCGTCTAGACGCGACTACTGCTCGAAATCGAGTGAATCAACTCAAAACCAGGCTCGAAGAACTTGATGCTATAGAAGATGCTCTGTTTGCTCAAACAATTTTTTATGACCAAATGGCAGAAACGCAACAAAAAAGTTCGTGGGAGTCAATTGAACAACTCGATGCAGATGAACTAATCATAAATGAAGCTTGGTTACGTGACACTAATTTCAAAATTTGCGATCGTTTGAGCCAATTAGAAATTGGAGCTTCATCCTCATTGGGATGTGAATTTTTGGAGTATGAAGTTTGA

>Solyc04g056740.1.1 Solanum lycopersicum|M-type_MADS|M-type_MADS family protein

ATGGAGAGAAAGAAGACTAAAGGACGTCAAAAGATACCAATAAAAAAAATAGAAAATGAGGATGCCCTCCTTACCACATTTTCAAAGCGTCGCGAGGGTTTGTACAAGAAAGCGAGTGAACTCGTTAGAGAATGCGACGTTGACATTGGAATAATGATGATCTCCCCTGCTGGTAAGCCACATTCATTTTTCCACCCTACTCTTGATGCAATTGTTACTCGTTTTCAGAATCCTGATATGCAGTTAAGCCAAGGTATTCTTCTAGACACGATCACTGCACGAAATAAAGTGAACGAACTCAAAAACAGGCTTGAAGAACTTGACGTTGTAGAAGATGCTACGATTGCTCAAACAACTTTTTATGACCAAATGGCTGAAATAAGACAAAAAGGTTGGTGGGAATCAATTGAACAGCTCAATGCAGATGAAGTGACCATATTTAATGCTTGGTTGAGTGACACTTGTTCCAAAATGTGCCATCGTCTAAAACAATTAGAAAATGGAGCTTCATCATCATTGGGACGTGGATCTTTTGGAGTGTGA

>Solyc04g064860.1.1 Solanum lycopersicum|M-type_MADS|M-type_MADS family protein

ATGAATATTAATAACAATACCAACGCCGCCGCCAACTCCGCCGCCGTCGCCGTCAAGAAAACTCAAGGGCGGAGGAAAATAGCAATTAAACCAATAGCAAATCAAAACAGTAGGCACGTTACCTTTAGCAAACGCCGTTTAGGTCTCTTCAAAAAAGCTAGCGAACTTTGCATTTTAACCGGCGCTGAAATCGCTATTATGGTTCAGTCGCTAAAACGACAGCGTTTGTTCACTTTTGGTCATCCGAGCGCCGACGCCGTCATTAACCGGTACCTTACCGGAAAATCAGAGGAACAAAAACCGGCGGTCGATGATCAGTTGAACTATGTGCAACAGAGCAATGAATATTATTCTCAGATCTGTAGAGAATTGGAGTTGGAGAAGAAGGTAAAGGAGGAGATTGTTATTGATGAATCGAAGATGGTGAATGGTGGTAGTAGTAGTAATAATGGAGGAGGAGGATTTTGGTGGAATGAATCGATTGATGAAATGGGAATTGAAGAACTTGAGAAATTTATGTTTGCATTGGAAGAATTGAAGAAGAAAGTAAATATGAGATGTGATGAATTGAGTATGATAAATGGTTCTTCTTCAATGGCGGCGGCGGCGACGGCGTCTACTTCAAGTATGAATCAAGCTATTGATTATTGTGCTTCCATTGTTCCTTTTGATTTCAATTACCCAGGAAATGCCCAATTTTGA

>Solyc04g076680.2.1 Solanum lycopersicum|M-type_MADS|M-type_MADS family protein

ATGGGGAGAGGTAAGATAGTGATAAGGAGGATCGATAATTCGACGAGCAGGCAAGTGACGTTCTCGAAGAGGAGAAATGGATTGTTGAAGAAGGCGAAGGAGCTAGCGATTCTGTGCGATGCGGAGGCCGGAGTTATTATCTTCTCCAGTACTGGAAAACTCTATGAATATTCAAACACCAGTAAAAAATAA

>Solyc05g013370.1.1 Solanum lycopersicum|M-type_MADS|M-type_MADS family protein

ATGGAGAAGAAGAATAAGGGCAACATGCTAAATTACAAGAAGAAAAAAGAAACTATCAAGAAAAAAACTAGAGAGCTTTCAATCCTCTGTGACGTTAAAGCTTGTGTCATTCTTGTTGATCCTAATGGAAAAGTCGATACATGGCCTGAAAATCCCACTGATTTCAACCCCATTATTCAATCCTACAAAGAAAATCTCTGTCACGGCAAGAGAAAACGAATTGACGATGATGGGTGTTTTGAGAAAAAGTCGAAAAAGAATCATGCTCTGTTTTGCGATGATGATGAAAATCAGTGGCTTAATGATGTGTTTAGAGAGTCGAATGAAAGTTTGTTGGTGAAATTGAATTCGAAATTAGAAGCTGTGGATAGAAGAATTGAATTCCTGAAGATGATGAATTATGGGAATGGGGTTGTTGGAGGATCAAGTTCAAGTGCGAAAGAAAGTTTACTTGCTAATCAAGAAACCCATAATCGACTTGAAAACAGCAACGCGTATAATCAAGAAACAGAAATTGCTATGGCGGCTGAGTTTTGGGTGATTGGTGGTGATGAATCTGCTAATGATCGGGGCAAAGAAATTGATTTTCTGAGAGATAATGCGACAGTAAACAATTTGAATAATGTGCAGAATTTTGGGTATGATGATCATCTTTGGCCAGTCATCGCTGCTTCTGAATTTTCGACTTGTATTAATTAA

>Solyc05g015730.1.1 Solanum lycopersicum|M-type_MADS|M-type_MADS family protein

ATGGGGAGGAAGAAAGTGGAAATAAAGCGAATTGAAGATAAGAGCAGTAGGCAAGCAACTTTCTCCAAACGGAGAAATGGACTCATGAAGAAAGCTAAACAGCTCTCTGTTCTCTGCGATGTTGATGTCGCCGTCCTCGTCTTCTCCAGTCGCGGACGCCTCTTTGAATTCTCCAGTACCAACAGGTTCTTCTTTTATTTATTTTTGGCGAATAGTCTGCTAGATCCTTATATTGTATTGTGA

>Solyc05g051830.2.1 Solanum lycopersicum|M-type_MADS|M-type_MADS family protein

ATGGGAAGAAGTAAATTGCCATTGTTGAAGATTGAGAGTTTGACCAATAGACAAGTCACTTTTAGCAAGAGAAGAAATGGAATTCTCAAAAAAGTTTATGAATTATCTGTTTTATGTGATGTAGATGTTGGTATTATAATGTTCTCTCCTTCTGGTCGTCTCACACATTATTCGCGTAAAAGAAGAATTGAAGATATTCTTTCTGAGCTCATTAGTCTCCCAGATAGTGAAAGAGGATTCTACATCAACAATAAAGAGTCTGTACTTTGGAACTTAAGGAAGATTGAAATTGAAGATAAATTTTGCGATATTGAAAGGATAAATCCTGCATACGTCAACGCAAATGACACAACAAAGAAAATTCAAGATGAAATCAATGGCTTGCATTGTAAACTCGATGAGGCTGAGGGATTATTAAGAATATTTGAACCAGATACACAAAGGATTACATCACTCCATGAGCTTGATTTATGTGAAAAACGTCTTCAAGTTGCATTAAATCAAGTTAGACAAAGAATGGAACAACTCTCTAGCAATAATACACCAAGTTATGAAGATAATATGGCGCAAATAAATGAACTTCTTCAACACATAGACAACACACAAGTTCATGAGAAACCTCCTTATGACTTATGGTTAGAGCTTGAAGATTATAATCATGAGAACAACAATATTAATAGTCCTCTCTATACTGCCTCAGAAACATCTTCAATTTCTCAAAGTTCTATGAACCTTCCATCCTCAACTACTTATGATACAATGTCCCAAACAAGTCTAAGTGGTGAGACTTATCAAAATAATAACAACTTTAAGCAATCACAACATTCAACAAGGACCTTACCAAATCTCACTTTACAAACTTCATTCAAATTTGCCAAGCCTGAAATGTCCCAAACAAGTATTGAAGGAAGTTTTAGTTGCCTCACTGATGAAAATTTGAAGAAATCAATATGTTCAAATAGGGTCTTCCCAGCTATCACTCCATTACAAACTTCATTCTCTTTTGCCAAGGCTGAAATGGAAACTCCAACCTCAGCATTAAGACCATTGGCACCATATCTACAGGCTGAAGCAACAACATCTTCTTGTACTAATCAAGAAGGGAACAATGAAATGTCTTGGTTTCAGCCCAAAGTGAAAAAATCCAAGCAATATCATTCAATTGACTAA

>Solyc06g033820.1.1 Solanum lycopersicum|M-type_MADS|M-type_MADS family protein

ATGGATCGCAATAAGATAATGAGGAAGAAAATTGAGGATCCAGTATCTCGTCAACAGTTCTACTTAAAGTGCAAGGATATCATTGTTAAGAAGTCAGATGAGTTGGGGTTTTTATGTAACTCAAATATTGCACTGTTAATGGTTTCTCAAAATGGTGAAGTGACCAGCTATTCTAGAGGAGAAAGTTTTGAGGATATTATGGTCAAAGCAATGAACCAGCCTGTTCAACTGAATCGACGATCCATTCCAAATCCAGATGAAGAGCATTTGATGCAGAGTCTTGTGCAATCAAAATCTGAAAGAGGAATGATTGAAAAGATTGCTATGTATGACACTCTACTTTTCTCGAGTTCTTTTATTTTGATCTATTTCTTTGTTGTGGTACTTTTCTACATGATTGAAAAATTCTTGTTATTTTTGTTGGGTGTTTTTACGATGCTGATGATTGGACAACGTGCTTCAGTGTTGAGTTCTTATGAACCACAAGTGGAGAATATCAACACAACGGAAGAAGCAGATGCATATAAGGAATATATTCTGGGTGCTATTGAACGGGTTCAACGATCCAAAGTGAGCATATCTAAAAGCTAA

>Solyc06g033830.1.1 Solanum lycopersicum|M-type_MADS|M-type_MADS family protein

ATGGGTCGCAATAAGGTTGTAATGAAGAAAATTGAGGATCCAGAATCGCGTAAACACTTCTACTCAAAGCGCAAGGATGGTCTTGTTAAGAAGTCGAATGAGTTGGGAGTTGTATGTCACACAAATATTGCATTGTTAATGTTTTCTCCAACTGGTGAAGTGACGACCTATTCTCGCGCAAAAAGTTCTTATGAACCACAAGTGGAGAACATCAACAATGTTGAAGAAGCTGATGCATATAAGGGGTATCTTCTGGGTGCTATGGAACGGGTTCAACGATCCAAAGAACCATTAGGGGATGAAAGATTTGATGAACTAAATTGGATCACAATGGAGAGGTAG

>Solyc06g035570.1.1 Solanum lycopersicum|M-type_MADS|M-type_MADS family protein

ATGAAAAGGATTGAGAATGCAACCAGCCGGCAAGTGACTTTTTCTAAACGGAGAAATGGAGTTATAAAGAAAGCTTATGAACTATCCGTTCTTTGTGATGCTCAAGTTGCTCTTATTATTTTCTCTAACAAAGGAAGACTCTTTCAATTCTCAAGCTCCTGGTAA

>Solyc06g048380.1.1 Solanum lycopersicum|M-type_MADS|M-type_MADS family protein

ATGGATCGCAATAAGATTATGATCAAGATAATTGAGGATCCAATATCCCGTCAACAGTTCTACTCAAAATGTAAGGATAGCATTGTTAAGAAGTCAAATGAGTTGGGGCTTTTGTGTGACACAAATATTGCACTGTTAATGGTCTCTCCAAATGGTGAAGTGACCAGCTGTTCTGGAGGAGAAAGTTTTGAGGATATTATGAGCAATGCAATGAACCAATTTGATGAACTAAATCGACATTCTTACGAACCACAAGCGGAGAATATCAACACAGTCGAAGAAGCTGATGCATATGAGCAGTATCTTCTGGGTGCTATAGGACGGATTCAACTATCCAAAGCAAAATTCTTGGATAATCAGGAATTTCTGAAGAGAAATGAAAATGTCGCGGAACCAGTAAGGGATGGAAGACCTGAGAGAAACATATTGGATCACAATGGATGTAGCTGCAGCAGAAATTATGTTATATTAGTTTATATATTAAAACTTAGTACAATGAATAACTTGCACCAAGCAAGCAAGCAAGATTAA

>Solyc06g054680.1.1 Solanum lycopersicum|M-type_MADS|M-type_MADS family protein

ATGGAGAGAAAAAAAACTAAAGGACGTCAAAAGATACCAATGAAAAAAATTGAAAATAAGGATTCGATGTTTGCTTCATTTACAAAGCGTCGTGAGGGTTTGTATAAAAAATCTAGCAAACTCGCTACAGAATACAATGTTGACATTGGAATAATGATGATTTCTGCTACTGGTAAGCCTCATTCCTTTTTTCACCCAACATTTGATGCAGTTATTTCTCGTTTTCAGAATCATGATATGCAGTTTGGTGAAAGGACAAATCTAGAGGCAAATGATGCTAGAAATGAAGTGAATCAACTCAAAACTAGGCTTGAAGAACTTGATGTCAGAGAAGACATTGCGATTGCTAAGAAAAATTCTTATGAACAAATGGAAGAAACAAGACAAAAAGGTTGGTGGGAGTCGACTGAGCAGCTCAATGCAGATGAAGTGTTCATATTTGAAACTTGGTTGAATGAAACTAGTTCTAACTTACACCATCGTTTAAATCAATTAGAAATTGAAGCTTCATCCTCAATGAGACATGAATCTTTTGGAGTGTGA

>Solyc06g059780.1.1 Solanum lycopersicum|M-type_MADS|M-type_MADS family protein

ATGGAGAGAAAAAAAACTAAAGGACGTCAAAAGATACCAATGAAAAAAATTGAAAATAAGGATTCGATGTTTGCTTCATTTACAAAGCGTCGTGAGGGTTTGTATAGAAAAGCTAGCGAACTCGCTACAGAATACAATGTTGACATTGGAATAATGATGATTTCCTCTACTGGTAAGCCTCATTCCTTTTTTCACCCAACATTTGATGCAGTTATTTCTCGTTTTCAGAATCCTGATATGCAGTTTGGTGAAAGCACAAATCTAGAGGCAAATGCTGCTAGAAATGAAGTGAATCAACTCAAAACTAAGCTTGAAGAACTTGATGTCAGAGAAGACATTGCAATTGCTAAGAAAAATTCTTATGAACAAATGGAAGAAACAAGACAAAAAGATTGGTGGGAGTCGACTGAGCAGCTCAATGCAGATCAAGTGTTCATATTTGAAACTTGGTTGAATGAAACTAGTTCTAACTTACACCATCGTTTAAATCAATTAGAAATTGAAGCTTCATCCTCAATGAGACATGAATCTTTTGGAGTGTGA

>Solyc06g071300.1.1 Solanum lycopersicum|M-type_MADS|M-type_MADS family protein

ATGGCAGAGGAAGATGAAATCAAGAGGAGAACGAACAATCCCAAGTCTTATCAAGTGAGGAAAGAATGCATAAAAAGGAAATCGATGGAGCTTGCGACTCTCTGCGATATCAAGGTTTGTACGGTTATTACTGGTCCTAATGGGGAACTGCAAACTTGGCCTGACGATTTCGATGCCTGTAAGCAAGTTCTCGATCTCTACTCTCAAAATTTGAAACCCGAAAAGAAGTACAAGGAATCATCGACACCAGAGCCAGAGCCAGAGCCAGAACCAGAACGAGGCGAAGAACAAGGAGAGAAAGATCTCCTGACGCTAGTTGAATCAACGCTTGCTGCTGTCAATAGGAGAATTTGTATATTGGAGAATAAGGGGAAAAGAAAGAGAATCGAGTGA

>Solyc07g052700.2.1 Solanum lycopersicum|M-type_MADS|M-type_MADS family protein

ATGAAGAAAATTGAGGATTCAACATCCCGTAAACAGTTCTATTCAAATCGCAAGGATAGCATTGTGAAGAAGTCAAATGAGCTGGCGGTTGTATGTGGTACAGATGTGGGGTTGTTGATGTTTTCTCCATCTGGTCAGCTGACTACCTATTCTAGCAAAGAAAGTATTGAGGACATCATGATCGAAGCTATGAACAAGTCTGTGAATCCGCGACCCATACCAAATCTAAATGAACAGCTTTTGATGCAGAGTCTCAAACAGTCAAAATCTGAAGGCCAAATGGTTGGAAAAATAGCTATTGCTGAGGCTCATGAGAAGAAGCTTAATGAGCTCAAAGAAACACTAAGGGAGGCACAACAGAAAATAAGGTATTGCAATCCGCAAGTGGAGAATATCAGCTCAGTCCAAGAAGCTGAAGCATATGAGCAGTTCCTTAGGAGTAATATGGAACAGATTCAACAATCAAAAGCAAAACTCTTAGGTGTCCAAGGATTAGTCCATAGAAATGAATATCCTGCGGTCAACACAGAGGATACGGCTGCTGCAGGAACCAGTAGTGGATGGATGTTTTGA

>Solyc09g061950.1.1 Solanum lycopersicum|M-type_MADS|M-type_MADS family protein

ATGGAGAATGAGAACAAAAAAGGCAAGCAAAAAATTGAAATGAAGTTGATTGAGAATGAGAGAGCACGTATGGTGAGTTTCTCAAAAAGAAAAAAAACTTTGTTCGAGGATGCACATAAGTTTGCAACTCGGACTGGAGCAGATGTTGGTGTTATGCTATTTTCACCAAGTGGAAAACCATATTCTAATGATTCCGCAACCATAGCAGATATAATTGATAGATTTCTCAAAGTGAAACAGGAGGATCACAAACGTGATTATGCTGAGGGAGAATCAAATGGTTTCGAGGCATTGAAAGATCTCCATAAAGAATTACAAGCATGGAACGACAAAGAGAAAAAACGAAAACTAATGCATAAGATTATGCACCCTAGCTTAGAAATACCTTCAGATAAACACATGGAGGAGCAGAAGCTAGCATTGAAGTTGAAGGTAGAGAAATTTAAAAATGAAATACAAAGTGCTATTACGACTGAGCATCTAAAGTTTGACCTAAATGTCGTTCCTGATCCAGAAGAGTAG

>Solyc10g012180.1.1 Solanum lycopersicum|M-type_MADS|M-type_MADS family protein

ATGGAATCAAAAAATTCCAATGTTGATTCAGCAGTTGCAAAAGATAAACGTGGTGAGGGTGGTAAAATCTCAAAAATGCAAAAAGCATTGTTCAAAAAAGCAAGCGACCTATCCATATTGTGTGGAATTCAGGTCGCCATCATAATTCTCTTCATTAACCGTCAACCAATTGTATTTGGAAAGCCTGATGCAGAATCGGTTATTAACCAGTTCATTGAGGCCAACCATCCAACCGCACCTCGATTTTATATGAAGATGAAAAAGAAGGAAGAAGAAAATAAGGAGAAAGGAAAATCCATTGAAGATGATATACAATCACAAGATTTTGAGTCTCCTTATTTGGGAAGTCTTTTAAAGTTGTACGAAGGGCTCACAGAATTTGAAAATCAATTGACTAAAGAGATTGATCTCACACAATTGAATCAAGAGATTGAGAAGCATGAAGATCCAAAATTAATGAATGTGGCTAGTTCTTCGACTTTACCAACTAATTTTAGTCCATAG

>Solyc10g012200.1.1 Solanum lycopersicum|M-type_MADS|M-type_MADS family protein

ATGGAATCAAAGAATTCCAATGTTGATTCAGCAGTTGCAAAAGATAAACGTGCTGAGGGTGGTAAAATCTTAAAGATGCAAAAAGCTTTGTTCAAAAAAGCAAGCGACCTTTCAATTTTGTGTGGTATTCAGGTCGCCATCATCATCCTCTTCATCAACCGTCAACCAATTGTATTTGGGAAGCCTGATGCAGAATTGGTTATTCACCAATTTATTGAGGCCAACCATCCAACCGCACCTCGATTTTATATGAAGATGAAAAAGAAGGAAGAAGAAAATAAGAAGAAAGGAAAATCCGTTGAAGATGATATACAATCACAAGATTTTGAGTCTCCTTATTTGGAAAGTCTTTTAAAGTTGTACGAAGGGCTCAGAGAATTTGAAAATCAATTGACTAAAGAGATGGATCTCAAACAATTGAATCAAGAGATTGAAAAGCACAAAGATCCAAAATTAATGAATGTGGCTAGTTCTTCGACTTTACCAACTAATTTTAATCTATAG

>Solyc10g012380.1.1 Solanum lycopersicum|M-type_MADS|M-type_MADS family protein

ATGCAGAAATCACTTTTTAAGAGAGCAAACGACCTTGCTATCTTATGTGGAATCCACATAGCTATCCTAATTTTCTCAGTCGGCCGTCAACCAATTTTTTTTGGAATGCCCGATGTTGAAACGGTTGTCCAAAAATTCATGGAGGCTAACCATCCAACCGCACCTCGATTTTATATGAAGATAAAAAAGACTGAAGAAGAAAACAAGGAGAAAGGAAAATCTGTTGAAGATAATATCGCACATCGGCAACTAGAAGATTTCGAGTCTCCTTATTTGGGAAGTCTTCTGAAGTTGTACCAAGGGCTCACAGAATTTGAAGATCTATTGAATAAGGAGATCGATCCCACACAATTGAATCAAGAGATCGAAAAACATGAAGATCCAAAAATAGTATCTAAAATGAATGTGGCTAGTTCTTCGTATTTACCAACTGATATGCTTAGTCCTTAA

>Solyc10g012390.1.1 Solanum lycopersicum|M-type_MADS|M-type_MADS family protein

ATGCAGAAATCACTTTTTAAGAGAGCAAACGACCTTGCTATCTTATGTGGAATCCACATAGCTATCCTAATTTTCTCAGTCGGCCGTCAACCAATTTTTTTTGGAATGCCCGATGTTGAAACGGTTGTCCAAAAATTCATGGAGGCTAACCATCCAACCGCACCTCGATTTTATATGAAGATAAAAAAGACTGAAGAAGAAAACAAGGAGAAAGGAAAATCTGTTGAAGATAATATCGCACATCGGCAACTAGAAGATTTCGAGTCTCCTTATTTGGGAAGTCTTCTGAAGTTGTACCAAGGGCTCACAGAATTTGAAGATCTATTGAATAAGGAGATCGATCCCACACAATTGAATCAAGAGATCGAAAAACATGAAGATCCAAAAATAGTATCTAAAATGAATGTGGCTAGTTCTTCGTATTTACCAACTGATATGCTTAGTCCTTAA

>Solyc10g017640.1.1 Solanum lycopersicum|M-type_MADS|M-type_MADS family protein

ATGGTGAGAGGGAAAACTGAAATGAGGCGTATCGAAAACGCGACAAGCAGGCAAGTCACTTTCTCAAAGAGAAGAAATGGATTGTTAAAAAAAGCCTTTGAGCTTTCTGTTCTTTGTGATGCTCAAGTTGGATTAGTTATTCTTTCTCCTAGAGACAAACTTTATGAATTTTCAACCTCAAGGTAA

>Solyc10g018070.1.1 Solanum lycopersicum|M-type_MADS|M-type_MADS family protein

ATGGAGCATAAGAAGACAGCAGGACGCCAAAAAATTTCATTGGCAAAAATAGAGAATGAATCTGCTAGACTCACCAAATTCTCTAAACGTCGTTCTGGCTTATACAAAAAAGCTTGTGAACTTGTTAGAGAATGTGATGTAGATCTTGGAATTGTTATGTCTTCACTGAAAGGTATACCTTATTCCTTTAGTAGTCCAACCTCTAATGTGGTCATTGATCGTTTTATAAATCCTACAGCAAATTTAAGTTCAAGTGACCGTCTTGTTGCTGTAGAAACACGCAAAAAACTAGAAAATGGAGCTTCATCCTCCTCACAAATTCCATCAGATGATGCAAATATCTCACCAAATGTCTTCTAG

>Solyc10g018080.1.1 Solanum lycopersicum|M-type_MADS|M-type_MADS family protein

ATGGAGCATAAGAAGACAGCAGGATGCCAAAAAACTTCATTGGCGAAAATAGAGAATGAATCTGCTCGACTCACCACATTCTCTAAACGACGTTCTGGCTTATACAAAATAGCTTGTGAACTTGTTAGAGAATGTGATGTAGATCTTGGAATTGTTATGTCTTCACCGAAAGGTATACCTTATTCCTTTAGTAGTCAAACCTCTAATGTGGTCATTAATCATTTTATAAATCCTACAGCAAAATTAAGTTCAAGTGACCACCTTGTTGCTTCAGAAGCACGCAAAAGAGTAAGTCAATTTAATGATATTTTAAACGAATTGGACGAAAGAGAAAAAATTGCAAATGAAAAGTTAGACCAAATGAATGAGGCTAGAGATCTAGGTTGGTGGGAGTCCATAGATCGATTGAATGTTCATGATGTAATGAAGTTGGAAGCATGGCTGAATTTACATTGA

>Solyc10g018110.1.1 Solanum lycopersicum|M-type_MADS|M-type_MADS family protein

ATGGAGCATAAGAAGACGGCAGGACGCCAAAAAATTTCATTGGCGAAAATAGAGAATGAATCTGCTCGACTCACCACATTCTCTAAACGACGTTCTGGCTTATACAAAAAAGCTTGTGAACTTGTTAGAGAATGTGATGTCGCTCTTGGAATTGTTATGTCTTCACCGAAAGGTATACCTTATTCCTTTAGTAGTCCAACCTCTAATGTGGTCATTGATCGTTTTATAAATCCTACAGCAAATTTAAGTTCAAGTGACCGCCTTGTTGCTGCAGAAGCACGCAAAAAAGTAAGTCAATTTTATGATATTCTAAACGAATTGGACGAAAGAGAAAAAATTGCAAATGAAAAATTAGATCGAATGAATGAGGCTAGAGATCTAGGTTGGTGGGAGTCCATAGATCAATTGAATGTACGTGATGTAAAGAAGTTGGAAGCATGGCTTATATCTGGTGAATTTAAATTGAACGAACATTTGGAGCAGCTTGAAAATGGAGCTTCATCCTCCTCACAGATTCCATCAGATGATGCAAATATCTCACCAAATGTCTTCTAG

>Solyc10g050900.1.1 Solanum lycopersicum|M-type_MADS|M-type_MADS family protein

ATGGAGAATAAGAAGACTAAAGGACGTCAAAAGATACCAATGAAAAAAATAGAAAATGAAAAGGCCTTGTTAAGTTCATTTTCAAAGCGCCGTAATGGTTTGTTCAAAGCAGCGAATAATCTCGTAAAAAAATTTGATGTAGACATTGGAATAATAGTGTTTTCTCCTACTGGTAAGCCTCATTCATATTTTCATCCTACAGTTGATGCAGTCATTTCTCGTTTTCAGAATCCTGATATGCAGTTAAGTGATGAAACTCACCTGGCCATGATTTTTGCTCGAAATTCGGTGAATCAACTCGAAAAAAAGCTTGAAGAACTTGACATCCAAGAAAAAATTGAAATTGATCGAACAAATTATCTTGACCAAATGACAGAAACTAGACAGAAAGGTTGGTGGGAATCAATTGAGCAACTTAATGAAGATGAAGTGTCCAAGTTTGAAGAATGGTTGAATGTTGCTAGTTTTACTATGCACTACCGTTTGAACCAATCAATTGTGTCATCCTAA

>Solyc10g050940.1.1 Solanum lycopersicum|M-type_MADS|M-type_MADS family protein

ATGGAGAATAAAAAGGCTAAAGGACGTCAAAAGATACCAATGAAAAAAATGAAAAATGAAAAGGCCTTGTTAAGTTCATTTTCAAAGCGCCGTGATGGTTTGTTCAAAGTAGCGAATAATCTCGTAAAAGAATTTGATGTTGACATTGGAATAATAGTGTTTGCTCCTACTGGTAAGCCTTATTCATTTTTTCACCCTACAGCTGATGCAGTCATTTCTCGTTTTCAGAATCCTGATACGCAGTTAAGTGATGAAACTCACCTGGCCATGGTTTTTGCTCAAAATTCGGTGAATCAACTCGAAAAAAAGCTTGAAGAACTTGACATCAAAGAAAAAATTGAAAGTGATCGAACAAATTATCTTGACCAAATGACAGAAACTAGACAGAAAGGTTGGTGGGAATCGATTGAGCAACTTAATGAAGATGAAGTGTCCAAGTTTGAAGCATGGTTGAATGTTGCTAGTTTTACTATGCACTACCGTTTGAACCAATCAATTGTTTCATCCTAA

>Solyc10g050950.1.1 Solanum lycopersicum|M-type_MADS|M-type_MADS family protein

ATGGAGAATAAGAAGATTAAAGGACGTCAAAAGATACCAATGAAAAAAATAGAAAATGAAAAAGCCTTGTTAAGTTCATTTTCAAAGCGTCGTAATGGTTTGTTCAAAGCAACGAATAATCTCATAAAAGAATTTGATGTTGACATTGGAATAATAGTGTTTTCTCCTACTAGTAAGCCTCATTCATTTTTTCACCCTACAACTGATGCAGTCATTTCTCGTTTTCAGAATCCTGATATGCAGTTAAGTGATGAAACTCACCTGGCCACAGTTTTTGCTCGAAATTCGGTGAATCAACTCGAAAAAAAGCTTGAAGAACTTGACATCAAAGAAAAAATTGGAAGTGATCGAACAAATTATCTTGACCAAATGACAGAAACTAGACAGAAAGGTTGGTGGGAATCAATTGAGCAACTTAATGAAGATGAAGTGTCCAAGTTGGAAGCATGGTTGGATGTTGCTAGTTTACTATGCACTACCGTTTAA

>Solyc11g020320.1.1 Solanum lycopersicum|M-type_MADS|M-type_MADS family protein

ATGGGAAGATCAAAAATCAAAATGGAACTTATTGAAGATCACAAGAAGAGGAAGTCTACTTTGGTAAATCGAAAAGCTGGCTTAGTCAAGAAAATCTCAGAACTCTCAATACTTTGTGACATTAAAGCAAGCATGATCATATATGAAGGAAATTATAATTATCAAATTTGGCCAAATGATTCAAATGACGTTCAAGACCTAATAAATCTCTACAAAAATCAGTCACAGGATGGGCGGACTAAGAGGGGTAAGACCTTGTCCAACTTCTTCAAAAATGATGAAAAGAAAAACAATGAGTTCAAAGTAGAAAAGTATCCTACTTGGGATTCAAGATTTGGCTATTTATCTCAAATAGAATTACAAAATCTTGTTGGTGTTGTTGAGAAAAGGATTGAGAAGGCAAAGGAAAAAATCGAATTGTTGAAGAGCATGAATGATCAAGATCCAAATATTGGAAGTTCCTCGTTTTCTCATCAACAACAAATATGGAACAACAACAACTTGATGAACCAAACTACTCAATGGCCTTTTTCTTATGTGAATCCTTTTACTCACTACGACAACTTCTTTCAAGCAAATATTCCAATTAGTGGTGCAAATTCAATGGGCGCGGGAATGGATGATGGACTTCTTACTATCGATGATTATCAATTTAACAACACAGATTATTCTACGATGATTGAGACAGAAAATTGTTTGGTGAAGAATGGAATTGGTTCAAGTTCAATAATGCATCCTATGATGAATAATGGAATTGATTCAAGTTCAACAATGGAGTACCCTTTCATATACAATGGCTATACTCATATGCCTTATGGATTTCAGTAA

>Solyc11g020620.1.1 Solanum lycopersicum|M-type_MADS|M-type_MADS family protein

ATGGGAAGATCAAAAATCAAGGTGGAACTAATTCAAGATGACAAGAAGAGGATGAAAACTTTAGTGACACGAAAGGCTGGCTTGTTCAAGAAAATTTCAGAACTCTCTATACTTTGCGATATCAAAGCATGCATGCTCATATATGATGAAGGAAATAATAATAATTGTGAAATGTGGCCGAATGATCCAAATGAGCTCATAAATCTATACAAAAATCAACCATTTGAAGGACCAACCAAAAGGGGTAAAACATTGTCCGGAGATGAGATCAAAGTAGAAAAGGATCCGGATTCAAGATTTGACTATTTCGAGAATAATGAGAAGAAAAAGGCTGATGCGATCCAAGTAGAAAAGTATCCTACTTGGGATTCAAGATTTGACTATTTATCTCAAAAAGAATTGCAAAATCTTGCTGGTGTTCTTGAGAATAGGATGGAGAATGCAAAAGGAAGAATAGAATTGTTGAAGAGCATGAATGGAAGTTGCTCACTTTCTCATCAACAACAAATATGGGACTATAACAACTTGATGAACCAAACTACTACTCTGTCTAACAACAACTTGTTTCAATCAAATATTCCAATTAGTAGTACTGTGAATTCAATGGGAGCAGGGATGGATTATCACTTTTGTAATGCTAATTATTGTAAGATGAATGAGTCTGAAAACTGGTATGGAGTTGGTTCAAGTTTGACAATGATGCAGCATAATATGGCAAATAATGGAATTGGTTCAAGTTCAACAATATTGCATCTTATGGGGGATGATTATGGGATTATTGATTCAAGTTCAACAATGATGCTGCCTATGGGGAATAACGAAATTGGTTCAAGTTCGACAATGCAGCAGCCTATGTATCAGTACCCTTTCATGTACAATGGCTCTACCCATGTCATTGGTTCAAGTTCGACAATGCAGCCTCCTATGGGTCAGTACCCTTTCACAAACAATGACGACTCTACTGGGATATTGGTTCAAGTTCAACAATGCAGCCTATGGGGAATAATAATGAGATTGGTTCAACTTTGA

>Solyc11g020660.1.1 Solanum lycopersicum|M-type_MADS|M-type_MADS family protein

ATGAACACTTCTGTAAAACGGAAGGCTAGCTTAGTCAAGAAAATTTCAGAACTCTCAATACTTTGCGACATCAAAGCCTGCATGATCATATATGATGAAGGAAATAGTAATTGTGAAATGTGGCCAAATGAAGTTAAAGAGCTAATAAATGTCTACAAAGATCAACCATTTGAAGGACGAACCAAAAGGGGTAAGACATTGTCCAACTATTTCAAAGATGAGATCAAAGTAGAAAAGGATCTGGATTCAAGATTTGACTATTTCGAGAATGATGAAAAGAAAAAGGCTGATGCAATCAATGTAGAAAAGTATCCGACTTGGGATTCAAGATTTGACTATTTATCTCAAAAAGAAATACAAAATCTTGTTGGTGTTATCTCAAAAAGGATGGAGAATGCAAAAGGAAGAATAGAATTGTTGAAGAGCATGAATGGAAGTTGCTCACTTTCTCACCGACAACAAATATGGGACTACAACAACTTGATGAACCAAACATCTCCATGGCCTATTTCTGATCATGTGAATTCTTTCAACAACTTTTTTCAATCAAATATTGGTGTGAATTCAATGATGGAGTCAGAAAACTGGTTGGCAAATGATGAAATTGGTTCAAGTTTGGCAATGCATCCTATGGGGAATAATTATGGGATTATTGATTCAAGTACGACAATGATGCAGCCTATGGGGAATAACGAAATTGGTTCAAGTTCAACAATGCAGCAGCCTATGTTTCAGTACCCTTTCATGTACAATGGCTCTACCCATGTCATTGGTTCAAGTTCGACAATGCAGCCTCCTATGGGTCAGTACCCTTTCACAAACAATGACGACTCTACTGGGGATATTGGTTCAAGTTCAATGCAGCCTATCGGGAATAATAATGAGATTGGACCCATGGACAATGACTCTACTTATGATTCCTGTACATTTTATTATTGA

>Solyc11g069770.1.1 Solanum lycopersicum|M-type_MADS|M-type_MADS family protein

ATGGGAAGAGATTTAATCATGGCAAGGCTTGGTCGCAGAAGAATCCGCATTGAAAAGATAGAAAACAAAAAGAAGAGGAGTGTCACATTCTCTAAAAGGCGTTATGGCCTTTTTAAGAAAGAAAGTGAGTTATCAATGCTTTGCGACAGTCCAAATGCCACGGTAGTTTTTTCCCCTGACGACGAAACTTGTGTTTACTCGATTGGGTACCCTTGTGTCAATTCAGTTCTGGATAAGTTTATGGACGTGAATCCTCCACAAAACCTTGATGACACTGGCTCCCTCAGTGTACATCGTCGAAATGCTATTAGAGAGGGGGTGTTGGCTCTAATGGAGATTGAAGAAGAGTTTGAAGAAGAGAAAAAACGCGAAAAAAGCCTAGACACGGGAATTTCATATGAAAATTTGAATTCCTCTGTGTATCAGAATTTCATCGAAAAAATTGAGATCGGCTATATGGAAGCGGAACAGCTAGCTATCGAACTAAAGGAGCGTAACGTACCATTTCCCTACAGTACCTTTGGAGATGCCTTAGCTCCTAAATAA

>Solyc12g005210.1.1 Solanum lycopersicum|M-type_MADS|M-type_MADS family protein

ATGACTATGAAGGGGGTTAGGAGCACTAGGAATGTAGATCCTAGAATGAAAAAATTCATTTTAGATAAAAGAATAGAAGCACTATTTAAGCAAGCAAATGACTTGTCAATTCTATGTGATATTGAAGTTGGTGTAATTGTTTTTGGTCCAGGAGAAAACAATGCTGTTGTTTGGCCATCTCTAGCACAGGCTAGTGATAGAGTGAAAAACTATTTAGCTAGGCATCGAGACTGCAAAGGTAAAGAAGTTGTCAACGTACGTCAATGA

>Solyc12g016150.1.1 Solanum lycopersicum|M-type_MADS|M-type_MADS family protein

ATGGCTACAAAAAGGTTGAGAGATACTGCGAACTACAATGAAAATGTGAGAAATTCCATCTTAGATAAAAGAGTAACAAGTTTGTTCAAGAAAGCAGAAGAGCTATCTATTGTGTGTGACGTAGAAGTTGCCATAATTATTTTTAGGCCAGGAAAAATCCAACCCATTACTTGGAAATCCCCAAGTCTGGCTCAGGATGTCTTGACGAGGTATTTAAGTTTTATTGAATTCAAAAGGCTGTCAAAGTTGGTCACACATGAAGACTATCTTCAAAAGAAAGTTGATAAGAAAGAAGAACAAATTAGTAAATTAGAGAAAATGAATGAGATGGAAGAATCAATCAATGATCCGTGGTTTATTCAGACTATAGCTACATTAGGGGATGTGAGCGGTGTAGAGTCTGCACAAAAAGAAGGCAAAGGCGTCAATGTTGAAGATGATGGACATTCCAAGGACCTCGATTGA

>Solyc12g016170.1.1 Solanum lycopersicum|M-type_MADS|M-type_MADS family protein

ATGGCTATAAAAAGGCTTAGAAATACTAGGAACTACAGTGAAAATGTGAGAAATTCCATCTTAGATAGAAGAGAAAAAAGTTTGTTCAAGAAAGCAGAAGAGCTTTCTATTTTGTGTGATGTGGAAGTTGCCATAGTTATTTTTAGGCCAGGAAAAATCCAACCCATCACTTGGAAATCCGCAAGTCTGGCTCAGGATGTCTTGACAAGGTATTTAGGTTTCATTGAGTTCAAAAGACTTAATAAGTTGGTCACACATGAAGACTATCTTCAAAAGAAAATTGATAAGAAAGAAGAACAAATTAGCAAATTAGAGAAAATGAATGAGGCGAAGGAAATGGAAATCCTCTTCAACCAACTAGTGGAGGGAAAAAGTATTAATGAACTTGATGCTAGAGAAATGAAAGGCTTGTTAAAGGTGTTTGCTGCAAAAATGGCTAAACTAGATGAAAGAAAGAAAGAACTTAACCAGACTCCAAATCCTCCATCAAACAAAGAAAACATTACTTTATCAGGAAGTCCAATGGAAGAATCATTCAATGGCCCGTGGTTTATTCAGACTATAGCTACGTTAGGGGATGGAAGTGATATAGAGTTTTCACCAAAAGAAGGCAATGGCGTCAATGTTGAAGATGATGGACATTCCAAGGACCTCGATTGA

>Solyc12g016180.1.1 Solanum lycopersicum|M-type_MADS|M-type_MADS family protein

ATGGCTATAAAAAGGCTTAGAAATACTAGGAACTACAGTGAAAATGTGAGAAATTCCATCTTAGATAGAAGAGAAAGAAGTTTGTTCAAGAAAGCAGAAGAGCTTTCTATTTTGTGTGATGTGGAAGTTGCCATTGTTATTTTTAGGCCAGGAAAAATCCAACCCATCACTTGGAAATCCGCAAGTCTGGATCAGGATGTCTTGACAAGGTATTTAGGTTTCATTGAGTTCAAAAGACTTAATAAGTTGGTCACACATGAATACTATCTTCAAAAGAAAATTGATAAGAAAGAAGAACAAATTAGCAAATTAGAGAAAATGAATGAGGCGAAGGAAATGGAAATCCTCTTCAACCAACTAGTGGAGGGAAAAAGTATTAATGAACTTGATGCTAGAGAAATGAAAGGCTTGTTAAAGGTGTTTGCTGCAAAAATGGCTAAACTAGATGAAAGAAAGAAAGAACTTAACCAGACTCCAAATCCTCCATCCAACAAAGAAAACATTACTTTATCAGGAAGTCCAATGGAAGAATCATTCAATGGCCCGTGGTTTATTCAGACTATAGCTACGTTAGGGGATGGAAGTGATATAGAGTTTTCACCAAAAGAAGGCAATGGCGTCAATGTTGAAGATGATGGACATTCCAAGGACCTCGATTGA

>Solyc12g017300.1.1 Solanum lycopersicum|M-type_MADS|M-type_MADS family protein

ATGGCTACAAAAAGGCTCAGAGACAGTAGGAATTACAGTGAAAATGTGAGAAATTCCATCTTAGATAGAAGAGAAATAAGTTTGTTCAAGAAGGCAGAAGAGCTTTCTATTTTGTGTGACGTAGAAGCTGCCATAATTATTTTTAGGCCAGGAAAAATTCAACCCATTGCTTGGAAATCCGCAAGTCTGGCTCAGGATGTCTTGACAAGGTATTTAAGTTTTCTTGAGTTCAAAAGGCTCGATAAGTTGGTCACACATGAAGACTATCTTCAAAAGTTAGTTGATAAGAAAGAAGAACAAATTACCAAATTACAGAAAATGAATGAGATGGAAGAGTCATTCAATGACCCCTGGTTTATTCAGTCTATAGCTACATTAGGGGATGGAAGTGGTATAGAGTCTACACCAAAAGAAGGCAATGGCGTCAATGTTGAATATGATGGACATTCAAAGGACCTCGATTGA

>Solyc12g087820.1.1 Solanum lycopersicum|M-type_MADS|M-type_MADS family protein

ATGGGGCGAAGGAAGGTAGAAATTAAGCGAATTCAAGATAAAAATTGCAGGCAAGTTGCGTTCTGTAAACGGAGGAAAGGTTTATTGAAGAAAGCTAAAGAAATTTCCGTTCTCTGCGATGTCGATGTTGCTGTTGTTATCATCTCAAATCGAGGCAGGCTCCATGAATTCTCCAGCAATAACAGGACTAGCACACCATGTTGGCTTGATAAAATCTCCCCAATGCATCAGGTTGAATTGATAACTCTAGGAAGCTCCACTTTAGGTGGCACAATGTGTGCATTACTTCAAGTTACTCGTCGTGCTTTGCCTACATTCTAG

>Solyc12g088080.1.1 Solanum lycopersicum|M-type_MADS|M-type_MADS family protein

ATGGGGAGAGGAAAGATATTGATAAGGAGGATCGATAATTCAACGAGCAGACAAGTGACGTTTTCGAAGAGGAGGAATGGATTGTTGAAGAAGGCGAAGGAGTTAGCGATTTTGTGTGATGCGGAGGTTGGAGTCATCATCTTCTCCAGTACTAGCAAGCTCTATGACTATGCAAACACCAGGTTTTCCCCCCCTATTTCTCACACTTTTTTTGCCTGTCAGATGCTTGAACTGCAATTTATATTTAATTTTCTGGAAAAAAACTTTTTGCGTGTGTTTTCATTCGTCTATTAG

**#amino acid sequences of MADS-box genes in *Solanum lycopersicum***

>Solyc01g087990.2.1|Solanum_lycopersicum|MIKC_MADS|Solyc01g087990.2.1

MGRGKIDIKLIENLNNRQVTFSKRRAGLLKKAGELSVLCDSEVAVIIFSSTGKLFEFSSTSMKQTLSRYNKCVASTDNSAVEKKSEDNEQPQLQQQTHVLKQEQKEVDSLKDELAKLKMKQQRLLGKDLNGMGLNELRLLEHQLNEGLLAIKERKEELLIQQLEYSRKQEERSALECETLRRQVEELRGLFPLSASLPPPFLEYDRPLEKKYSILKESKESLDSDTACEDGVDDEDSNTTLQLGLPTICRKRKRTEQESPSSNSENQVGSK*

>Solyc01g093960.2.1|Solanum_lycopersicum|MIKC_MADS|Solyc01g093960.2.1

MGRGRVELKRIENKINRQVTFSKRRNGLLKKAYELSVLCEAEVALIIFSSRGKLYEFGSAGITKTLERYQRCCLNPQDNCGERETQSWYQEVSKLKAKFEALQRTQRHLLGEDLGALSVKELQNLEKQLEGALAQARQRKTQIMMEQMEELRRKERHLGDVNKQLKIKVSLELSSFEGEGQGVPFPWSNCNASLDEAGSSTFHVHHSQSNHMDCDLPDPVLQIGYHQYMAADGASGSRNMAVESNIIHGWGL*

>Solyc01g105800.2.1|Solanum_lycopersicum|MIKC_MADS|Solyc01g105800.2.1

MEQITIQLKSSNSNSHEEIQLLLCNQKYPNYYFFSFYCNNIPFFSFFSSQLQISSPFLANFSKEMVRQKIQIKKIDNLTARQVTFSKRRRGLFKKAQELSTLCDADIGLIVFSATGKLFEYSSSSMMQLIEKHKMQSERDGMDNPEQLHSSNILSEKKTHAMLNRDFVEKNRELRQLHGEELQGLGLDELMKLEKLVEGGISRVLKIKGDKFMKEISSLKKKEAKLQEENSQLKKQSQARLNEEGQNVIEQGHSADSITNNRSLVNDSDTSLKLCLAFP*

>Solyc02g065730.1.1|Solanum_lycopersicum|MIKC_MADS|Solyc02g065730.1.1

MGRGRVEMKRIENKISRQVTFSKRRSGLLKKTNEISVLCDAEVALIVFSSNGKLFEYSTQSSMENILERYENYSYEEMNLNTTYKENWTLEYPKLMARVELLQRNIRHFMGEDLDAFNLREFRGLEKQLDTALKRVRSKKNQLMHESISQLQKKEKELQQRNNLISNKLKENEKKQIVQTNPGQSSTMTFLLQSPTVTNQTIGGPSQATDQSQNRDGYNSLMPPWMFHHVHNKG*

>Solyc02g071730.2.1|Solanum_lycopersicum|MIKC_MADS|Solyc02g071730.2.1

MDFQSDLTREISPQRKLGRGKIEIKRIENTTNRQVTFCKRRNGLLKKAYELSVLCDAEVALVVFSNRGRLYEYANNSVKATIERYKKACSDSSNTGSVSEANAQYYQQEASKLRAQIGNLMNQNRNMMGEALAGMKLKELKNLEQRIEKGISKIRSKKNELLFAEIEYMQKREVDLHNNNQYLRAKIAETERAQHQHQQMNLMPGSSSNYHELVPPPQQFDTRNYLQVNGLQTNNHYPRQDQPPIQLV*

>Solyc02g084630.2.1|Solanum_lycopersicum|MIKC_MADS|Solyc02g084630.2.1

MGRGKIEIKKIENSTNRQVTYSKRRNGIFKKAKELTVLCDAKISLIMLSSTRKYHEYTSPNTTTKKMIDQYQSALGVDIWSIHYEKMQENLKRLKEINNKLRREIRQRTGEDMSGLNLQELCHLQENITESVAEIRERKYHVIKNQTDTCKKKARNLEEQNGNLVLDLEAKCEDPKYGVVENEGHYHSAVAFANGVHNLYAFRLQPLHPNLQNEGGFGSRDLRLS*

>Solyc02g089200.2.1|Solanum_lycopersicum|MIKC_MADS|Solyc02g089200.2.1

MGRGRVELKRIENKINRQVTFAKRRNGLLKKAYELSVLCDAEVALLVFSNRGKLYEFCSTNNMLKTLDRYQKCSYGTLEVNRSIKDNEQSSYREYLKLKAKYESLQRYQRHLLGDELGPLTIDDLEHLEVQLDTSLKHIRSTRTQMMLDQLSDLQTKEKLWNEANKVLERKMEEIYAENNMQQAWGGGEQSLNYGQQQHPQSQGFFQPLECNSSLQIGYDPITTSSQITAVTNAQNVNGMIPGWML*

>Solyc02g089210.2.1|Solanum_lycopersicum|MIKC_MADS|Solyc02g089210.2.1

MGRGRVELKRIENKISRQVTFSKRRSGLLKKANEISVLCDADVALIVFSTKGKLFEYSSNDSSMESILERYERCSYAERQMNANDSDPKENWSVEYPKLMSRIELLQRNIRHYMGQDLDPLSLRELQSIEQQIDTSLKRIRSRKNQLMHESISELQKKEKALQEQNNLITKKLKENEKTQPNSSGQNSATVHVFPSHSHHQLPNLTIGGAFGGMNRDGSGQAHHYPGSNNNNNNSSLIPPWMLRHVSNEG*

>Solyc02g091550.1.1|Solanum_lycopersicum|MIKC_MADS|Solyc02g091550.1.1

MGRGKIVIRRIDNSTSRQVTFSKRRNGLLKKAKELAILCDAEVGLIIFSSTGKLYEFSNTSMKSVIERYNKTKDDCQQLHNPVSELKLWQREAEILRQQLQDLQDNHRQLLGEELSGLGVKELTNLENQLEMSLKGIRMKKEQILKDEIQELTRKGSIIHQENMELYKKVNLIRQENAELYKKAYGARDANAVNGNINYPYRFTVSREVQAPIHLQLSQPEPQYFEMQAGTSDSR*

>Solyc03g006830.2.1|Solanum_lycopersicum|MIKC_MADS|Solyc03g006830.2.1

MKRIENSTSRQVTFSKRRNGLTKKAYELSVLCDAEVAFIIFSHKGRLYEFASSNMQKIIERYRGRARETTTVDKSTELEHYMENLKHETANMAKKIEILEISKRKLMGQGLGSCSMDELEDIDSQLERTLKIIRARKTQLFKEEIESLKAKERLLLQQNASLREKCGLRPMLSESASAPEPIPAPPSTPPAQSKERGNCSQSTKSWEVETELFIGLPQTRCL*

>Solyc03g019710.2.1|Solanum_lycopersicum|MIKC_MADS|Solyc03g019710.2.1

MGRGKVELKRIENQTNRQVTFSKRRNGLLKKAYELSILCDAEVALLLFSPSGKAYHFASHDIERTILRYKNEVGLSKNSDQGPRAMEVWRTKIDDMTRTIHELEARDKHFAGEELSNLGMKELKQLERQLRVGVERIRSKKHKILHEENIHLQKQVKLYEVEGSSRILDTNPRMRII*

>Solyc03g114830.2.1|Solanum_lycopersicum|MIKC_MADS|Solyc03g114830.2.1

MGRGRVQLKRIENKINRQVTFSKRRSGLLKKAHEISVLCDAEVGLIVFSTKGKLFEYSTDSCMERILERYERYSYAERQLNATDIITPGSWTLEHAKLKARLEVLQRNQKHYAGEELDTLSMKELQNLEHQLDSALKHIRSRKNQLMHESISELQKKDKALQEQNNNLSKQVKEREKEMAQQTPWEQQSHDHLNSSSFVLPHPFNNLHIGEAYPNAGDNGEVEGSSRQQQQNSASVMPPWMLRHLNG*

>Solyc03g114840.2.1|Solanum_lycopersicum|MIKC_MADS|Solyc03g114840.2.1

MGRGRVELKRIENKINRQVTFAKRRNGLLKKAYELSVLCDAEVALIIFSNRGKLYEFCSTSSMVKTIEKYQRCSYATLEANQSVTDTQNNYHEYLRLKARVELLQRSQRNFLGEDLGTLSSKDLEQLENQLESSLKQIRSRKTQFMLDQLADLQQKEQMLAESNRLLRRKLEESVAGFPLRLCWEDGGDHQLMHQQNRLPNTEGFFQPLGLHSSSPHFGYNPVNTDEVNAAATAHNMNGFIHGWML*

>Solyc04g005320.2.1|Solanum_lycopersicum|MIKC_MADS|Solyc04g005320.2.1

MGRGKVELKRIENKINRQVTFAKRRNGLLKKAYELSILCEAEVALIIFSNRGKLYEFCSTSSMSDTLERYHRCSYGDLETGQSSKDSQNNYQEYMKLKARVEVLQQSQRHILGEDLGQLNTKDLEQLERQLDSSLRLIRSRRTQNMLDQLSDLQQKEQSLLEINRSLKTKLEENSVAHWHITGEQNVQFRQQPAQSEGFFQPLQCNTNIVPNRYNVAPLDSIEPSTQNATGILPGWML*

>Solyc04g078300.2.1|Solanum_lycopersicum|MIKC_MADS|Solyc04g078300.2.1

MGRVKLQIKKIENTTNRQVTFSKRRNGLIKKAYELSVLCDVDVALIMFSPSGRVSTFSGNKSIEDIMARYVNLPEHDRGRLHNQEHLQRAIAKLKCEADRTYQAPSSPSSVDSHIEEFQQEIIRYKTQVEDMERRLRMYEGGFCEITTVCEAQYREEILQETLKQVQARKQVLEENYHSPQTQNTTQPQMDFSGQNVNMVNNVATSDAIANSTFMDWVPHSQRDPHVQILNFLDSSGLLPFRDEADQHMLPPSLNQLHGVNVPAGTDHLSSNSRFDQNNPPRPSSFDGIIDVNNAPWPPLYTTGDDPFPVSQPRERAILELFLSQLTPVNQDHI*

>Solyc04g081000.2.1|Solanum_lycopersicum|MIKC_MADS|Solyc04g081000.2.1

MARGKIQIKKIENQTNRQVTYSKRRNGLFKKANELTVLCDAKVSIVMISSTGKLHEFISPSITTKQLFDLYQKTIGVDIWTTHYEKMQEQLRKLKDVNRNLRKEIRQRMGESLNDLNYEQLEELMENVDNSLKLIRERKFKVIGNQIETYRKKVRNVEEINRNLLLEFDARQEDPYGGLVEHDGDYNSVLGFPTGGPRILDLRLQPNNNYHNHLHSGGGSDITTFALG*

>Solyc05g012020.2.1|Solanum_lycopersicum|MIKC_MADS|Solyc05g012020.2.1

MGRGKVELKRIENKINRQVTFAKRRNGLLKKAYELSILCDAEIALIIFSSRGKLYEFCSNSSMSKTLERYHRYNYGTLEGTQTSSDSQNNYQEYLKLKTRVEMLQQSQRHLLGEDLGQLGTKDLEQLERQLDSSLRQIRSTKTQHILDQLAELQQKEQSLTEMNKSLRIKLEELGVTFQTSWHCGEQSVQYRHEQPSHHEGFFQHVNCNNTLPISYGYDNVQPENAAPSTHDATGVVPGWML*

>Solyc05g015750.2.1|Solanum_lycopersicum|MIKC_MADS|Solyc05g015750.2.1

MGRGRVELKRIENKINRQVTFAKRRNGLLKKAYELSVLCDAEVALIIFSNRGKLYEFCSSSSMLKTLERYQKCNYGAPEPNISTREALEISSQQEYLKLKGRYEALQRSQRNLLGEDLGPLNSKELESLERQLDMSLKQIRSTRTQLMLDQLTDYQRKEHALNEANRTLKQRLMEGSQLNLQWQPNAQDVGYGRQTTQTQGDGFFHPLDCEPTLQIGYQNDPITVGGAGPSVNNYMAGWLP*

>Solyc05g056620.1.1|Solanum_lycopersicum|MIKC_MADS|Solyc05g056620.1.1

MGRGKVELRKIENKINRQVTFSKRRGGLVKKAHEISVLCDAEVALIVFSQKGKIFEYSSDSCMEQILERYERYSYAERRLLANNSESPVQENWSLEYTKLKARIDLLQRNHKHYMGEDLDSMSLKDLQNLEQQLDSALKLIRSRKNQLMHESISELQKKERAILEENNMLTKKIKEKDKIVEQQGEWHQQTNQVSTSTSFLLQPHQCLNMGGNYQDEVAEARRNNELDLNLDSLYPLYNMNKHL*

>Solyc06g059970.2.1|Solanum_lycopersicum|MIKC_MADS|Solyc06g059970.2.1

MGRGKIEIKRIENTNNRQVTYSKRRNGIIKKAKEITVLCEAKVSLIIFASSGKMHEYCSPSTTISDMLDGYQKASGRRLWDAKHENLSNEIDRIKKENDSMQVKLRHLKGEDINQLTHKELIIMEEALQNGLSSISAKQSEILRMVRKNDQILEEENKQLQYALHQKEMGAIGGSGNMRGIHEEVYHQRERDYEYQMPFGLRVQPMQPNLHERM*

>Solyc06g069430.2.1|Solanum_lycopersicum|MIKC_MADS|Solyc06g069430.2.1

MGRGRVQLKRIENKINRQVTFSKRRSGLLKKAHEISVLCDAEVGLIVFSTKGKLFEYANDSCMERILERYERYSFAEKQLVPTDHTSPVSWTLEHAKLKARLEVLQRNQKHYVGEDLESLSMKELQNLEHQLDSALKHIRSRKNQLMHESISVLQKKDRALQEQNNQLSKKVKEREKEVAQQNQWEINSSSFVLPQQLDSPHLGEAYQSTNVIDNGEVEGGSSSQQQGAANNTVMPQWMLRHLNN*

>Solyc07g055920.2.1|Solanum_lycopersicum|MIKC_MADS|Solyc07g055920.2.1

MVFPINQELLVDESSSQLRKTSGGTGGGGRGKIEIKRIENTTNRQVTFCKRRNGLLKKAYELSVLCDAEVSLIVFSSRGRLYEYANNSVRATIDRYKKHHADSTSTGSVSEANTQYYQQEASKLRRQIRDIQTYNRQIVGEALGSLSPRDLKNLEGKLEKAIGRVRSKKNELLFSEIELMQKREIELQNANMYLRIAEVERAQEQMNLMPGGGGGGGGGGGGGSDHQYHHQPNYEDARNNFLPVNLLEPNPHYSRRDNGDQTPLQLV*

>Solyc08g067230.2.1|Solanum_lycopersicum|MIKC_MADS|Solyc08g067230.2.1

MGRGKIEIKRIENSSNRQVTYSKRRNGILKKAKEISVLCDAHVSVIIFATSGKMHEFSSTSLVDILDQYHKLTGRRLWDAKHENLDNEINKVKKDNDNMQIELRHLKGEDISSLNYRELMILEDALENGLTGIREKQNEFMRMMRKKTQNMEQEQDQLNCQLRQLEIASMNRNMGEIGEVFEQTRENHDYGQMPFAFRVQPMQPNLHQRF*

>Solyc08g080100.2.1|Solanum_lycopersicum|MIKC_MADS|Solyc08g080100.2.1

MVRGKTELKRIENATSRQVTFSKRRSGLLKKAFELSVLCDAEVALIVFSPKGKLYEFSSSSTNKTIERYQKNEKSLGRLNRKLTDQLTTEHLKEEVATMTRKLEFLEDSKRKLLGHGLESSTFDELQKVEEQLEKSLSNIRARKNLLFKEQIAQLKEEEKILLKENVDLKKKCQVLPLTLTPVPLVEKDVERQIMEVETELFIGLPETRKSSYCPNLNTLPTLL*

>Solyc10g080030.1.1|Solanum_lycopersicum|MIKC_MADS|Solyc10g080030.1.1

MGRGKIEMKKIENISSRQVTFSKRRAGLFKKAEELSVLCDAEIGVIVFSNTDRLYKFASSKSSMEKIVERYNSSSHSFEHPMIENVVEPELNSLKAEVAKLRKATGRMMGKELDGLDFKELQQLEHQLTEGILSVKNKKEQVLLELLEKSNLQIEELGHKSCNHYPENYEAARKISGGNTTVICDFKSVEEENSDTSLSLGLSVATSQKKKNPQIECTSNDSENLMILD*

>Solyc11g005120.1.1|Solanum_lycopersicum|MIKC_MADS|Solyc11g005120.1.1

MGRGKIEVKRIENKTSRQVTFSKRRAGLLKKTHELSVLCDAQIGLIIFSTKGKLFEYTTQPHSMGEIINKYLQTTGASLPIHDHRVEQYDEITKMKRETLNLELSLQRYKGDELNSAQYDELNELEKQLENSINKIRARKLELLQQQMENLKRTEKMLEKENHDMCQWLMKYEMYKQQPVAMMEQQEEAAITELNLLGEQPLLSQFSFFGDQHQLGTTSNSSAYHLQTSHPFTPSTYD*

>Solyc11g010570.1.1|Solanum_lycopersicum|MIKC_MADS|Solyc11g010570.1.1

MAREKIQIKKIDNSTARQVTFSKRRRGLFKKAEELSVLCDADVALIIFSSTGKLFDYSSSSMKQILERRDLHSKNLEKLDQPSLELQLVENSNYSRLSKEISEKSHRLRQMRGEELQGLNIEELQQLERSLETGLSRVIERKGDKIMREINQLQQKGMHLMEENEKLRQQVMEISNNNNNNNNGYREAGVVIFEPENGFNNNNNEDGQSSESVTNPCNSIDPPPQDDDSSDTSLKLGLATLLRLKRSKARCGYFCMLLEEGEKKK*

>Solyc11g028020.1.1|Solanum_lycopersicum|MIKC_MADS|Solyc11g028020.1.1

MGRGKIEIKRIENNTNRQVTFCKRRNGLLKKAYELSVLCDAEIALIVFSTRGRLYEYSNNNVKATIERYKKATAETSSAYTTQELNAQFYQQESKKLRQQIQMMQNTNRHLVGEGLSSLNVRELKQLENRLERGITRIRSKKHEAILAETEDLHKREIQLEQENAFLRSKIAENERLQELSMMPSGGEEYNAFQQYLARNMLQLNMMETALPSYDPLSPDHKR*

>Solyc11g032100.1.1|Solanum_lycopersicum|MIKC_MADS|Solyc11g032100.1.1

MARGKVQMKRIENPVHRQVTFCKRRAGLLKKAKELSVLCDAEIGLFIFSAHGKLYELATKGSMQGLIERYIKSTKGVEVAEEAKDTQPLDPKEEINMLKNEIDVLQKGLSYMYGGGAGTMTLDELHSLEKYLEIWMYHIRSAKMDIMFQEIQLLKNKEGILEAANKYLQDKIDEQYTVTNMTQNLTDFQCPLTVQNEIFQF*

>Solyc12g038510.1.1|Solanum_lycopersicum|MIKC_MADS|Solyc12g038510.1.1

MGRGRVELKRIENKINRQVTFAKRRNGLLKKAYELSILCDAEVALIIFSSRGKLYEFSSASSMMTTLEKYQQCSYASLDPMLPVSDTQMNYNEYVRLKARVELLQRSQRHILGEDLGTLNSKELEQLEHQLDASLKKVRSKKTQSMLDQLADLQEKEQMLEEANKQLKNKLEESAARIPLGLSWGNNGGQTMEYNRLPPQTTAQPFFQPLRLNSSSPQFGYNPNMGANDHEVNAATTAHNINGFIPGWML*

>Solyc12g056460.1.1|Solanum_lycopersicum|MIKC_MADS|Solyc12g056460.1.1

MVRGITEMKRIENTTSRQVTFSKRRGGLLKKAFELSVLCDAEVSLIIFSQKGKLFEFSSSSTNKTIERYQKNDKNLGHENILLEQTTEHLKGEVMSMTRNLEVLEISKRRLLGEDLESCSIDELEKVEGQLDQSLRNIRAKKNQLFKEQISLLKDEEKVLMNKNAELREKYEARSLPLFIDRREDESPQTQNMEVDTQLFIGLPER*

>Solyc12g087830.1.1|Solanum_lycopersicum|MIKC_MADS|Solyc12g087830.1.1

MGRRKVEIKRIQDKNCRQVAFCKRRKGLLKKAKEISILCDVDVAVVIISNRGRLHEFSSNNSMTAMLRRYESHVGAEKEINAEIQVAEVSGFTTMGELLQTTERQLEETNADGLTLTDLIHLENELQTALIHLRARKTHLMLESAKVLHEKEKLLLEEKKHLEDNIASIKKNTKVNEMSDLPAPHMICGQQKVTLNFF*

>Solyc00g179240.1.1|Solanum_lycopersicum|M-type_MADS|Solyc00g179240.1.1

MRRIENATSRQVTFSKRRNGLLKKAFELSVLCDAEVGLIIFSPRGKLYEFASSRX

>Solyc01g010300.1.1|Solanum_lycopersicum|M-type_MADS|Solyc01g010300.1.1

MAQTKILRRQKIKRKMITSKVKRMVLFKKLWANFVKEAHDLYMTTGAHVTIVAFSPTGKAYAYDSSNNFDTIERFLNDSKASAIEGGH*

>Solyc01g060300.1.1|Solanum_lycopersicum|M-type_MADS|Solyc01g060300.1.1

MRKPNGRKKIEIAKIQNQTNLQVTLSKRRAGLFKKASELSTLCGANVAIVAFSPSNKVYACGHPSVESIVDKFIGENPPPETDDPNPIIVMTADTIPKGSPFTSMQEFSLGIDFGKLCQITCVPQLIESCPNSSILQIWTDYHLGSTNELAHQALLDLGGYRLVILPLHRAGTVRCSVKPASRSPFHFRNPSAHSFDWIARPNSMLGQGRP*

>Solyc01g060310.1.1|Solanum_lycopersicum|M-type_MADS|Solyc01g060310.1.1

MRKPNGRKKIEIAKIQNQTNLQVTFSKRRAGLFKKASELSTLCGANVAIVSFSPSNKVYACGHPSVESIVDKFIGENTPPETDDLNPIIVMGLSFLETDYHLGPTTELAQVCVPAARQMSGARSIIVVSQCVLERLIAT*

>Solyc01g066500.1.1|Solanum_lycopersicum|M-type_MADS|Solyc01g066500.1.1

MTKGTGKKKIDIKKLASNSSRKVTFSKRRTGLFKKAEELATKFGARIALLVFSPAGRLYTSGDVSIFENNGFPSKPHLSTETASNSYVKPSTLRLKNLNVEGCSEIFSPDGVHYSAGEIPIVEDNLQNNGFSSNSHLSTDFSSNFYEDWSTLWLKNFKGNNEIFSPPGGFYNSGEFPIVADELKNNEFPSNSDLSIEISSNSYAKPSTLHLKNFNAEGCSEIFSTDGRFYSSNEFPIINDTLKNNGFSSNSDLSTDLGSNLYEYWSMENFNVEECSSIEELMLLKEKLEEKRDEIILKMEAEFIDSLLV*

>Solyc01g066730.2.1|Solanum_lycopersicum|M-type_MADS|Solyc01g066730.2.1

MGKGKQKIEIKKITKESARKVAFSKRRKGLFKKAVQLESKTGAKVAILVFSSSGKPYTCGDVETLCGISDSFNLQTHSESNSIWDSFNLQTHSESNGIWDSFNLERPCESNGMWDSFNIIEGPCSSSGKNGMWDSFNLERPCSSSGQSGMWDSFNLGRPCESNGMWDSFNIIEGPCSSSGQNGMWDSFNLERPCSSSGQSGMWDSFNLERHCSSSGIPSGSNGIWDSFNVETHCSSSESCGMSDSFNVETHCSSSGQSGTWDSFNVEACHNVNELLLLKAHLESTREKLLESQFLDSLWS*

>Solyc01g097850.1.1|Solanum_lycopersicum|M-type_MADS|Solyc01g097850.1.1

MENKKSRGRQKIPMKKIEKLGDLYASFSKRRLSLYKKASDLVFECDVDIGMIYFSPKGNPYSFFHPNVNTVVSRFQNPDMEFSESDLLITTDNQVKVKELKSRLDELDIIEDIAIATKKSYDEVIKARKRGWWESIEQLNEPQVTKFEAWMDTIIFNMQNRLNEMENGASSS*

>Solyc01g098050.1.1|Solanum_lycopersicum|M-type_MADS|Solyc01g098050.1.1

MGTGKKKIEIEKIIKETSRMVTFSKRRKGLFKKAKQFESMTGSRVASIVLSPTGRPYTCGDVDYAIRTHFSNSGRCMKLLITDIMNSHDSNSSSNVVVYGETSRSKFSSAPKKNSLHNWVKRIDVEQCQNLNWLLMLKQQLEGTKEKIGEDVESFKAFFV*

>Solyc01g098060.1.1|Solanum_lycopersicum|M-type_MADS|Solyc01g098060.1.1

MGTRKRRTEIEKLTKQSDRLTTFSKRKKGIFKKAELLESLTSSRVTSVVFSPSGIPYTYGNVNSVIKKHFPSCNRSEISTTVMNSHHDVSGESSGSKSLSIPKENGLRRWVEDIDVEGCQNLNQLFMLKEQLEGTREKIISSDPESFEALFM*

>Solyc01g098070.1.1|Solanum_lycopersicum|M-type_MADS|Solyc01g098070.1.1

MGTGKKKIEIEKITKQTARMVAFSKRRKGLFRKAEELESMSSSRVTSVVISPFGKPYTYGNVNSVIKKYFSICIRPEISTPVMNSHPSSSNVSGESLGSKSSSTPNGNALCNWVEGIDVEECQNLNQLLMLKKQLEGTREKIVSKESESFQALFI*

>Solyc01g102260.2.1|Solanum_lycopersicum|M-type_MADS|Solyc01g102260.2.1

MTRKKVKLAFITNDSARKATFKKRKKGLMKKVSELSTLCGIDACAIIYSPYDTSPEVWPNTMGAQRVLAEFKRMPEMEQSKKMVNQESFIRQRIAKASEQLKKQSKENREKEMTEVMYQGLTGKGLQNLNLGDLNDLGWVIDQNLKEVYKRIEAVKKGASTSSSSSVAAAAVAAAAVASQAVAPPMEQKPAVVELGLDSMQRTQTEWFTDWMNSNASDQHIGYGHADEMILPNFNDNHNANVWPNNFYP*

>Solyc01g103550.1.1|Solanum_lycopersicum|M-type_MADS|Solyc01g103550.1.1

MGRAKLKMELISKEKSRNATFKKRKEGLLKKLYEFTTLCNVNGLMIMYGPKQGNGSECRPEIWTNSSGSSSSTNSKSLQQQQEEIENLIDEYKKENSLQSGSSKTFGLSDYFVDRNKRVEEEFIKLRKMNMEKKYPCWLEFMDQLSEFKLRDFLTLLDDRVENVKARIHLLKGNFSGLMGGEMIDLGGGNQWTHYNDNVMVQGGGMEYGDYNQLQAPIYHQEMRMVMMNENDWPQYNNGASSSSSAGNGSNNMMCALMKYETMMPSNNHLAYSPYVAPTILQQTPCMMMPQHSWRDNDRDDKAKFSPYMTK*

>Solyc01g103870.1.1|Solanum_lycopersicum|M-type_MADS|Solyc01g103870.1.1

MADEDQSKKGEEDQRKKKTNNSKSYQVRKECIKRKSMELATLCDIKVCTVITGPNRELQTWPDNLNACKEVLDIYSQNLKPEKKHKQEDKDLPTLVESKLAAVNRRICFLENKNVADKGKGKRIE*

>Solyc01g106170.2.1|Solanum_lycopersicum|M-type_MADS|Solyc01g106170.2.1

MIFLDRCQRVRLLWCTRASRGSKISDYFFTKPIFLLSLSLSICRISLLSKSLKLSRLIKEMGRGKIVIQRIDNTTSRQVTFSKRRNGLLKKAKELAILCDAQVGLIIFSSTGKLYEFANNSMKSTIDRYNKIKEENNNMNPMSEVKVT*

>Solyc01g106700.2.1|Solanum_lycopersicum|M-type_MADS|Solyc01g106700.2.1

MTTINAVKKTQGRRKIAIKPIDNQNSRHVTFSKRRLGLFKKASELCILSGAEIAILVQSLKRQRLFTFGHPSPDAVIDRYLTGKSVSPGDGDQFNLQQSNQYYSQICRDLELEKQKKENIEESKMVNNGGFWWNEPIDDMGIEGLEEFMSALEELKKKVTMRADELSMINGSSSNSTMKIARFGAEDQYFNESIDYCSSIVPFDFNQPGDRQF*

>Solyc01g106710.1.1|Solanum_lycopersicum|M-type_MADS|Solyc01g106710.1.1

MAKKPSMGRQKIKIAKIEVKNHLQVTFSKRRSGLFKKASELCTLCGVEIAIIVFSPARKVFSFGHPNVESIIDRFLTRANSNNNPIANNSIQLVEAHRNASVRGLNLQLTQILGEVEIEKKRGESLDQMRKTSQSQYWWEAPINQLDLQELEQLKDSMEVLKKNVTNQANKFMVNETPNPSFFGVNANGIFDNYDIKPPRNMNASNNLHNHNLGFDSSTFF*

>Solyc01g106720.1.1|Solanum_lycopersicum|M-type_MADS|Solyc01g106720.1.1

MAKKMSKGRQKIQMTKMSKESNLLVTFSKRRSGLFKKASELCTLCGVEIAIVVFSPGHKVFSFGHPNVDSIVNRFLTRNPTSSSSTTCQLVEAHRNANVRELNAQLTEILNQLEFEKKREVEIEKIQKGKIGKNWWEGPLNELEHGELEQLKLGMEELKKNVTKQMQKIIFEASNAPTFFLGGSSSSNGDVKNMKGLGLSMATNGGHTSFP*

>Solyc01g106730.1.1|Solanum_lycopersicum|M-type_MADS|Solyc01g106730.1.1

MARRVSKGRQKVEMVKMKNASNLQVTFSKRRAGLFKKASELCTLCGAEIAIVVFSPGDKVFSFGHPNVETLVDRFLGRDLPLPNNDVHNHLIVAHREAGIRELNTKLMNLEGVLHMEKKRGESLQEIRRRANGQWWESPIEELNLFQLQHLKEALENLEQKVEKVAHQQQMLNNIAFPFRTLGSALTPPNCARETSSYGLNVGAPFANRDIGSTSSIVPNH*

>Solyc02g032000.1.1|Solanum_lycopersicum|M-type_MADS|Solyc02g032000.1.1

MENETKKGKKKIEMKLIESERARAVSFSKRKKTLFEDAKKFATQTGADVAVMLFSPGGKPYSCGSTSVEDIIENFLKMKVADPRRHYAEGESKVLRN*

>Solyc03g007020.1.1|Solanum_lycopersicum|M-type_MADS|Solyc03g007020.1.1

MGSKIRGLTLSNHETFSQVFLPINLFFRKNILHQKLSHGKKKTLGRQKISMVKIENEDARYTTFSKRRSTLYKKASELVGKYDVDVGITLFSPTDKPYSFFHPTVDVVVDRILNPNTQPSEDNSIAIASYRNKVKDQKVELDELDIIERGISNSTFGTKETNMKNIWESFMKFNEDEVNELELWLNSIDFDLKNYLSQLENNVSSSTQAPPKNVG*

>Solyc03g034260.1.1|Solanum_lycopersicum|M-type_MADS|Solyc03g034260.1.1

MDSKKTRVRQTFPISKIENQRVSDVTFFNRRSSLYRMANELVDFCDVDIGIVLFSPSNHPFSFFHPTSEAVIERFLNPDSQLSEKTRLDAEQARNKVNQLNNRLDAMEKRIEQIEEIEHAQTLLQLSQTEENGERSKWKSIDQLNANEIPTFEAWLRTTVSKMNYRLEKLENEASSLKNARGTS*

>Solyc03g062820.1.1|Solanum_lycopersicum|M-type_MADS|Solyc03g062820.1.1

MGTGKKKIEMEKITKKSCRMVTFSKRRNGLFKKNEELESLTGSQVSSVVLSPAGRIYTYGDVNTAINMHFSKIDCMRQSDSDVVVSSGSSELRSSSKSLRDWLEDIDVEQCQNLNQLLLLKEQLEGTKKKIVSIEDSKSFQALFM*

>Solyc03g115910.1.1|Solanum_lycopersicum|M-type_MADS|Solyc03g115910.1.1

MASFACVFCPLEFHISEPLPAFKLGSCPTSNPKFQSAPICFFHRMGRVRLSVKRLESHSNRQSTYCKRRCGILKKAQEISVLCDIDIILLLFSPTGKPTLFQGGQSNFDEIIAKFAQLTPQERAKRKLESLEVSLIFITTMCSPLAMLCCYFIIQVKVITMLMVTLQTLRKTFKKLDNDIGVPEFLDASDPSVEELHSQVKLLQSRLTDVEMRLNWWSNPDNINKVEDFALMECALRESLNAVHVRKLQKTTHLHLLMNSELDGNTHQWHPENKILRMPFPQTPNILPQENMGYFGDNSVAESSHVQGSGEVDQARQDTTAMLDNGVLNDLTSIACLRQQLSEQYSYNPNEDLDLLERNMLDPQSDANLKGYLMDYAFQRNFNLTRSVDSVNYSAVDAVAVPDFDEKSYAQPATSSD*

>Solyc03g119680.1.1|Solanum_lycopersicum|M-type_MADS|Solyc03g119680.1.1

MENETKKGKQKIEMKLIESEKARTVSFSKRKRTLFQDVDKFAAQTGADVGVMLFSPSGKPYSHGSTSIEEIIDQYLKVKLEDHQRDHAEGKMNGFEVLGALHKELQAWNEKEKNRKLMYKIMHSGSEAPPDKHMEEQKLALKLRVEKIKKETQAAILVEHLKFDLNVAPEPEEDESS*

>Solyc04g025030.1.1|Solanum_lycopersicum|M-type_MADS|Solyc04g025030.1.1

MTTRINKGRQRVDMVKMKNARNLQVTFSTRLAGLFKKSNELCMLFNAEIFIVVFSQGDKGVLCFDHPSVNPLAEGFFEWNLPQPHINVHNQHIVARKEGGTRDLSTKLMSLEAILEKEKNCGQILIEIRKRANSL*

>Solyc04g025050.1.1|Solanum_lycopersicum|M-type_MADS|Solyc04g025050.1.1

MKNTRNLRVTFSKHRVGLFKKASKLCMLCGAEISIVVFSPNGKVFSFGHLSMDTLI*

>Solyc04g025110.1.1|Solanum_lycopersicum|M-type_MADS|Solyc04g025110.1.1

MATRINKGSQRVDMVKMKNARNLQVTFSKRLAGLLKKALELCMLCGAKIIIVAFSPSDNGDFSFGPTSISPSVERFLGRKFPQPNNDVHNQQIVALIEGGICELNTKLRNLEGILEMEINRGPSLGELGR*

>Solyc04g025970.1.1|Solanum_lycopersicum|M-type_MADS|Solyc04g025970.1.1

MEGKKKAGCQRIPLEKIEKKVARYASFFKRRLCLYKKASELIQERDVDIGVFISSQTGKPYSFVHQTANVVINHFKSPTTIDLGAQFAGAEARNNVIQMNDMLNDFDAREKVTKNHI*

>Solyc04g047870.1.1|Solanum_lycopersicum|M-type_MADS|Solyc04g047870.1.1

MSRRIFKGRQRVDMVKITNGRNLGVTFSKRRAGLYKKACELCMLCGAEIAIVIFSPEGKIFSFGHPSVETLVERFLGRNLPPPNNDVHNQQIVAHREAGIRELNTRLMNVEGALQMEKNRGESLQEIRKKADGVWWQSPIKELNLFHLQHLKRALEILKQKVVKEAQMVNNNAFPFQTLGSAWSPPNYTS*

>Solyc04g056550.1.1|Solanum_lycopersicum|M-type_MADS|Solyc04g056550.1.1

MERKKTKGRQKIPMQKIENKNALLTTFSKRRKGLFKKASEVVTECDVDIGIMMISPSGKPHSFFHPTADAIVSRFQNPDMQLSEGIRLDATTARNRVNQLKTRLEELDAIEDALFAQTIFYDQMAETQQKSSWESIEQLDADELIINEAWLRDTNFKICDRLSQLEIGASSSLGCEFLEYEV*

>Solyc04g056740.1.1|Solanum_lycopersicum|M-type_MADS|Solyc04g056740.1.1

MERKKTKGRQKIPIKKIENEDALLTTFSKRREGLYKKASELVRECDVDIGIMMISPAGKPHSFFHPTLDAIVTRFQNPDMQLSQGILLDTITARNKVNELKNRLEELDVVEDATIAQTTFYDQMAEIRQKGWWESIEQLNADEVTIFNAWLSDTCSKMCHRLKQLENGASSSLGRGSFGV*

>Solyc04g064860.1.1|Solanum_lycopersicum|M-type_MADS|Solyc04g064860.1.1

MNINNNTNAAANSAAVAVKKTQGRRKIAIKPIANQNSRHVTFSKRRLGLFKKASELCILTGAEIAIMVQSLKRQRLFTFGHPSADAVINRYLTGKSEEQKPAVDDQLNYVQQSNEYYSQICRELELEKKVKEEIVIDESKMVNGGSSSNNGGGGFWWNESIDEMGIEELEKFMFALEELKKKVNMRCDELSMINGSSSMAAAATASTSSMNQAIDYCASIVPFDFNYPGNAQF*

>Solyc04g076680.2.1|Solanum_lycopersicum|M-type_MADS|Solyc04g076680.2.1

MGRGKIVIRRIDNSTSRQVTFSKRRNGLLKKAKELAILCDAEAGVIIFSSTGKLYEYSNTSKK*

>Solyc05g013370.1.1|Solanum_lycopersicum|M-type_MADS|Solyc05g013370.1.1

MEKKNKGNMLNYKKKKETIKKKTRELSILCDVKACVILVDPNGKVDTWPENPTDFNPIIQSYKENLCHGKRKRIDDDGCFEKKSKKNHALFCDDDENQWLNDVFRESNESLLVKLNSKLEAVDRRIEFLKMMNYGNGVVGGSSSSAKESLLANQETHNRLENSNAYNQETEIAMAAEFWVIGGDESANDRGKEIDFLRDNATVNNLNNVQNFGYDDHLWPVIAASEFSTCIN*

>Solyc05g015730.1.1|Solanum_lycopersicum|M-type_MADS|Solyc05g015730.1.1

MGRKKVEIKRIEDKSSRQATFSKRRNGLMKKAKQLSVLCDVDVAVLVFSSRGRLFEFSSTNRFFFYLFLANSLLDPYIVL*

>Solyc05g051830.2.1|Solanum_lycopersicum|M-type_MADS|Solyc05g051830.2.1

MGRSKLPLLKIESLTNRQVTFSKRRNGILKKVYELSVLCDVDVGIIMFSPSGRLTHYSRKRRIEDILSELISLPDSERGFYINNKESVLWNLRKIEIEDKFCDIERINPAYVNANDTTKKIQDEINGLHCKLDEAEGLLRIFEPDTQRITSLHELDLCEKRLQVALNQVRQRMEQLSSNNTPSYEDNMAQINELLQHIDNTQVHEKPPYDLWLELEDYNHENNNINSPLYTASETSSISQSSMNLPSSTTYDTMSQTSLSGETYQNNNNFKQSQHSTRTLPNLTLQTSFKFAKPEMSQTSIEGSFSCLTDENLKKSICSNRVFPAITPLQTSFSFAKAEMETPTSALRPLAPYLQAEATTSSCTNQEGNNEMSWFQPKVKKSKQYHSID*

>Solyc06g033820.1.1|Solanum_lycopersicum|M-type_MADS|Solyc06g033820.1.1

MDRNKIMRKKIEDPVSRQQFYLKCKDIIVKKSDELGFLCNSNIALLMVSQNGEVTSYSRGESFEDIMVKAMNQPVQLNRRSIPNPDEEHLMQSLVQSKSERGMIEKIAMYDTLLFSSSFILIYFFVVVLFYMIEKFLLFLLGVFTMLMIGQRASVLSSYEPQVENINTTEEADAYKEYILGAIERVQRSKVSISKS*

>Solyc06g033830.1.1|Solanum_lycopersicum|M-type_MADS|Solyc06g033830.1.1

MGRNKVVMKKIEDPESRKHFYSKRKDGLVKKSNELGVVCHTNIALLMFSPTGEVTTYSRAKSSYEPQVENINNVEEADAYKGYLLGAMERVQRSKEPLGDERFDELNWITMER*

>Solyc06g035570.1.1|Solanum_lycopersicum|M-type_MADS|Solyc06g035570.1.1

MKRIENATSRQVTFSKRRNGVIKKAYELSVLCDAQVALIIFSNKGRLFQFSSSW*

>Solyc06g048380.1.1|Solanum_lycopersicum|M-type_MADS|Solyc06g048380.1.1

MDRNKIMIKIIEDPISRQQFYSKCKDSIVKKSNELGLLCDTNIALLMVSPNGEVTSCSGGESFEDIMSNAMNQFDELNRHSYEPQAENINTVEEADAYEQYLLGAIGRIQLSKAKFLDNQEFLKRNENVAEPVRDGRPERNILDHNGCSCSRNYVILVYILKLSTMNNLHQASKQD*

>Solyc06g054680.1.1|Solanum_lycopersicum|M-type_MADS|Solyc06g054680.1.1

MERKKTKGRQKIPMKKIENKDSMFASFTKRREGLYKKSSKLATEYNVDIGIMMISATGKPHSFFHPTFDAVISRFQNHDMQFGERTNLEANDARNEVNQLKTRLEELDVREDIAIAKKNSYEQMEETRQKGWWESTEQLNADEVFIFETWLNETSSNLHHRLNQLEIEASSSMRHESFGV*

>Solyc06g059780.1.1|Solanum_lycopersicum|M-type_MADS|Solyc06g059780.1.1

MERKKTKGRQKIPMKKIENKDSMFASFTKRREGLYRKASELATEYNVDIGIMMISSTGKPHSFFHPTFDAVISRFQNPDMQFGESTNLEANAARNEVNQLKTKLEELDVREDIAIAKKNSYEQMEETRQKDWWESTEQLNADQVFIFETWLNETSSNLHHRLNQLEIEASSSMRHESFGV*

>Solyc06g071300.1.1|Solanum_lycopersicum|M-type_MADS|Solyc06g071300.1.1

MAEEDEIKRRTNNPKSYQVRKECIKRKSMELATLCDIKVCTVITGPNGELQTWPDDFDACKQVLDLYSQNLKPEKKYKESSTPEPEPEPEPERGEEQGEKDLLTLVESTLAAVNRRICILENKGKRKRIE*

>Solyc07g052700.2.1|Solanum_lycopersicum|M-type_MADS|Solyc07g052700.2.1

MKKIEDSTSRKQFYSNRKDSIVKKSNELAVVCGTDVGLLMFSPSGQLTTYSSKESIEDIMIEAMNKSVNPRPIPNLNEQLLMQSLKQSKSEGQMVGKIAIAEAHEKKLNELKETLREAQQKIRYCNPQVENISSVQEAEAYEQFLRSNMEQIQQSKAKLLGVQGLVHRNEYPAVNTEDTAAAGTSSGWMF*

>Solyc09g061950.1.1|Solanum_lycopersicum|M-type_MADS|Solyc09g061950.1.1

MENENKKGKQKIEMKLIENERARMVSFSKRKKTLFEDAHKFATRTGADVGVMLFSPSGKPYSNDSATIADIIDRFLKVKQEDHKRDYAEGESNGFEALKDLHKELQAWNDKEKKRKLMHKIMHPSLEIPSDKHMEEQKLALKLKVEKFKNEIQSAITTEHLKFDLNVVPDPEE*

>Solyc10g012180.1.1|Solanum_lycopersicum|M-type_MADS|Solyc10g012180.1.1

MESKNSNVDSAVAKDKRGEGGKISKMQKALFKKASDLSILCGIQVAIIILFINRQPIVFGKPDAESVINQFIEANHPTAPRFYMKMKKKEEENKEKGKSIEDDIQSQDFESPYLGSLLKLYEGLTEFENQLTKEIDLTQLNQEIEKHEDPKLMNVASSSTLPTNFSP*

>Solyc10g012200.1.1|Solanum_lycopersicum|M-type_MADS|Solyc10g012200.1.1

MESKNSNVDSAVAKDKRAEGGKILKMQKALFKKASDLSILCGIQVAIIILFINRQPIVFGKPDAELVIHQFIEANHPTAPRFYMKMKKKEEENKKKGKSVEDDIQSQDFESPYLESLLKLYEGLREFENQLTKEMDLKQLNQEIEKHKDPKLMNVASSSTLPTNFNL*

>Solyc10g012380.1.1|Solanum_lycopersicum|M-type_MADS|Solyc10g012380.1.1

MQKSLFKRANDLAILCGIHIAILIFSVGRQPIFFGMPDVETVVQKFMEANHPTAPRFYMKIKKTEEENKEKGKSVEDNIAHRQLEDFESPYLGSLLKLYQGLTEFEDLLNKEIDPTQLNQEIEKHEDPKIVSKMNVASSSYLPTDMLSP*

>Solyc10g012390.1.1|Solanum_lycopersicum|M-type_MADS|Solyc10g012390.1.1

MQKSLFKRANDLAILCGIHIAILIFSVGRQPIFFGMPDVETVVQKFMEANHPTAPRFYMKIKKTEEENKEKGKSVEDNIAHRQLEDFESPYLGSLLKLYQGLTEFEDLLNKEIDPTQLNQEIEKHEDPKIVSKMNVASSSYLPTDMLSP*

>Solyc10g017640.1.1|Solanum_lycopersicum|M-type_MADS|Solyc10g017640.1.1

MVRGKTEMRRIENATSRQVTFSKRRNGLLKKAFELSVLCDAQVGLVILSPRDKLYEFSTSR*

>Solyc10g018070.1.1|Solanum_lycopersicum|M-type_MADS|Solyc10g018070.1.1

MEHKKTAGRQKISLAKIENESARLTKFSKRRSGLYKKACELVRECDVDLGIVMSSLKGIPYSFSSPTSNVVIDRFINPTANLSSSDRLVAVETRKKLENGASSSSQIPSDDANISPNVF*

>Solyc10g018080.1.1|Solanum_lycopersicum|M-type_MADS|Solyc10g018080.1.1

MEHKKTAGCQKTSLAKIENESARLTTFSKRRSGLYKIACELVRECDVDLGIVMSSPKGIPYSFSSQTSNVVINHFINPTAKLSSSDHLVASEARKRVSQFNDILNELDEREKIANEKLDQMNEARDLGWWESIDRLNVHDVMKLEAWLNLH*

>Solyc10g018110.1.1|Solanum_lycopersicum|M-type_MADS|Solyc10g018110.1.1

MEHKKTAGRQKISLAKIENESARLTTFSKRRSGLYKKACELVRECDVALGIVMSSPKGIPYSFSSPTSNVVIDRFINPTANLSSSDRLVAAEARKKVSQFYDILNELDEREKIANEKLDRMNEARDLGWWESIDQLNVRDVKKLEAWLISGEFKLNEHLEQLENGASSSSQIPSDDANISPNVF*

>Solyc10g050900.1.1|Solanum_lycopersicum|M-type_MADS|Solyc10g050900.1.1

MENKKTKGRQKIPMKKIENEKALLSSFSKRRNGLFKAANNLVKKFDVDIGIIVFSPTGKPHSYFHPTVDAVISRFQNPDMQLSDETHLAMIFARNSVNQLEKKLEELDIQEKIEIDRTNYLDQMTETRQKGWWESIEQLNEDEVSKFEEWLNVASFTMHYRLNQSIVSS*

>Solyc10g050940.1.1|Solanum_lycopersicum|M-type_MADS|Solyc10g050940.1.1

MENKKAKGRQKIPMKKMKNEKALLSSFSKRRDGLFKVANNLVKEFDVDIGIIVFAPTGKPYSFFHPTADAVISRFQNPDTQLSDETHLAMVFAQNSVNQLEKKLEELDIKEKIESDRTNYLDQMTETRQKGWWESIEQLNEDEVSKFEAWLNVASFTMHYRLNQSIVSS*

>Solyc10g050950.1.1|Solanum_lycopersicum|M-type_MADS|Solyc10g050950.1.1

MENKKIKGRQKIPMKKIENEKALLSSFSKRRNGLFKATNNLIKEFDVDIGIIVFSPTSKPHSFFHPTTDAVISRFQNPDMQLSDETHLATVFARNSVNQLEKKLEELDIKEKIGSDRTNYLDQMTETRQKGWWESIEQLNEDEVSKLEAWLDVASLLCTTV*

>Solyc11g020320.1.1|Solanum_lycopersicum|M-type_MADS|Solyc11g020320.1.1

MGRSKIKMELIEDHKKRKSTLVNRKAGLVKKISELSILCDIKASMIIYEGNYNYQIWPNDSNDVQDLINLYKNQSQDGRTKRGKTLSNFFKNDEKKNNEFKVEKYPTWDSRFGYLSQIELQNLVGVVEKRIEKAKEKIELLKSMNDQDPNIGSSSFSHQQQIWNNNNLMNQTTQWPFSYVNPFTHYDNFFQANIPISGANSMGAGMDDGLLTIDDYQFNNTDYSTMIETENCLVKNGIGSSSIMHPMMNNGIDSSSTMEYPFIYNGYTHMPYGFQ*

>Solyc11g020620.1.1|Solanum_lycopersicum|M-type_MADS|Solyc11g020620.1.1

MGRSKIKVELIQDDKKRMKTLVTRKAGLFKKISELSILCDIKACMLIYDEGNNNNCEMWPNDPNELINLYKNQPFEGPTKRGKTLSGDEIKVEKDPDSRFDYFENNEKKKADAIQVEKYPTWDSRFDYLSQKELQNLAGVLENRMENAKGRIELLKSMNGSCSLSHQQQIWDYNNLMNQTTTLSNNNLFQSNIPISSTVNSMGAGMDYHFCNANYCKMNESENWYGVGSSLTMMQHNMANNGIGSSSTILHLMGDDYGIIDSSSTMMLPMGNNEIGSSSTMQQPMYQYPFMYNGSTHVIGSSSTMQPPMGQYPFTNNDDSTGILVQVQQCSLWGIIMRLVQL*

>Solyc11g020660.1.1|Solanum_lycopersicum|M-type_MADS|Solyc11g020660.1.1

MNTSVKRKASLVKKISELSILCDIKACMIIYDEGNSNCEMWPNEVKELINVYKDQPFEGRTKRGKTLSNYFKDEIKVEKDLDSRFDYFENDEKKKADAINVEKYPTWDSRFDYLSQKEIQNLVGVISKRMENAKGRIELLKSMNGSCSLSHRQQIWDYNNLMNQTSPWPISDHVNSFNNFFQSNIGVNSMMESENWLANDEIGSSLAMHPMGNNYGIIDSSTTMMQPMGNNEIGSSSTMQQPMFQYPFMYNGSTHVIGSSSTMQPPMGQYPFTNNDDSTGDIGSSSMQPIGNNNEIGPMDNDSTYDSCTFYY*

>Solyc11g069770.1.1|Solanum_lycopersicum|M-type_MADS|Solyc11g069770.1.1

MGRDLIMARLGRRRIRIEKIENKKKRSVTFSKRRYGLFKKESELSMLCDSPNATVVFSPDDETCVYSIGYPCVNSVLDKFMDVNPPQNLDDTGSLSVHRRNAIREGVLALMEIEEEFEEEKKREKSLDTGISYENLNSSVYQNFIEKIEIGYMEAEQLAIELKERNVPFPYSTFGDALAPK*

>Solyc12g005210.1.1|Solanum_lycopersicum|M-type_MADS|Solyc12g005210.1.1

MTMKGVRSTRNVDPRMKKFILDKRIEALFKQANDLSILCDIEVGVIVFGPGENNAVVWPSLAQASDRVKNYLARHRDCKGKEVVNVRQ*

>Solyc12g016150.1.1|Solanum_lycopersicum|M-type_MADS|Solyc12g016150.1.1

MATKRLRDTANYNENVRNSILDKRVTSLFKKAEELSIVCDVEVAIIIFRPGKIQPITWKSPSLAQDVLTRYLSFIEFKRLSKLVTHEDYLQKKVDKKEEQISKLEKMNEMEESINDPWFIQTIATLGDVSGVESAQKEGKGVNVEDDGHSKDLD*

>Solyc12g016170.1.1|Solanum_lycopersicum|M-type_MADS|Solyc12g016170.1.1

MAIKRLRNTRNYSENVRNSILDRREKSLFKKAEELSILCDVEVAIVIFRPGKIQPITWKSASLAQDVLTRYLGFIEFKRLNKLVTHEDYLQKKIDKKEEQISKLEKMNEAKEMEILFNQLVEGKSINELDAREMKGLLKVFAAKMAKLDERKKELNQTPNPPSNKENITLSGSPMEESFNGPWFIQTIATLGDGSDIEFSPKEGNGVNVEDDGHSKDLD*

>Solyc12g016180.1.1|Solanum_lycopersicum|M-type_MADS|Solyc12g016180.1.1

MAIKRLRNTRNYSENVRNSILDRRERSLFKKAEELSILCDVEVAIVIFRPGKIQPITWKSASLDQDVLTRYLGFIEFKRLNKLVTHEYYLQKKIDKKEEQISKLEKMNEAKEMEILFNQLVEGKSINELDAREMKGLLKVFAAKMAKLDERKKELNQTPNPPSNKENITLSGSPMEESFNGPWFIQTIATLGDGSDIEFSPKEGNGVNVEDDGHSKDLD*

>Solyc12g017300.1.1|Solanum_lycopersicum|M-type_MADS|Solyc12g017300.1.1

MATKRLRDSRNYSENVRNSILDRREISLFKKAEELSILCDVEAAIIIFRPGKIQPIAWKSASLAQDVLTRYLSFLEFKRLDKLVTHEDYLQKLVDKKEEQITKLQKMNEMEESFNDPWFIQSIATLGDGSGIESTPKEGNGVNVEYDGHSKDLD*

>Solyc12g087820.1.1|Solanum_lycopersicum|M-type_MADS|Solyc12g087820.1.1

MGRRKVEIKRIQDKNCRQVAFCKRRKGLLKKAKEISVLCDVDVAVVIISNRGRLHEFSSNNRTSTPCWLDKISPMHQVELITLGSSTLGGTMCALLQVTRRALPTF*

>Solyc12g088080.1.1|Solanum_lycopersicum|M-type_MADS|Solyc12g088080.1.1

MGRGKILIRRIDNSTSRQVTFSKRRNGLLKKAKELAILCDAEVGVIIFSSTSKLYDYANTRFSPPISHTFFACQMLELQFIFNFLEKNFLRVFSFVY*
